# New insights into the evolution of host specificity of three *Penicillium* species and the pathogenicity of *P. Italicum* involving the infection of Valencia orange (*Citrus sinensis*)

**DOI:** 10.1080/21505594.2020.1773038

**Published:** 2020-06-11

**Authors:** Liang Gong, Yongfeng Liu, Yehui Xiong, Taotao Li, Chunxiao Yin, Juanni Zhao, Jialin Yu, Qi Yin, Vijai Kumar Gupta, Yueming Jiang, Xuewu Duan

**Affiliations:** a Key Laboratory of Plant Resource Conservation and Sustainable Utilization/Guangdong Provincial Key Laboratory of Applied Botany, South China Botanical Garden, Chinese Academy of Sciences, Guangzhou, China; b Center of Economic Botany, Core Botanical Gardens, Chinese Academy of Sciences, Guangzhou, China; c Key Laboratory of Post-Harvest Handling of Fruits, Ministry of Agriculture, Guangzhou, China; d BGI PathoGenesis Pharmaceutical Technology Co., Ltd, BGI-Shenzhen, Shenzhen, China; e Innovation Center for Structural Biology, Tsinghua-Peking Joint Center for Life Sciences, School of Life Sciences, Tsinghua University, Beijing, China; f Department of Chemistry and Biotechnology, ERA Chair of Green Chemistry, School of Science, Tallinn University of Technology, Tallinn, Estonia

**Keywords:** *Penicillium* species, genomics, genome comparison, evolution of host specificity, *p. italicum*, transcriptomics, metabolomics, pathogenicity

## Abstract

Blue and green molds, the common phenotypes of post-harvest diseases in fruits, are mainly caused by* Penicillium *fungal species, including* P. italicum, P. digitatum,* and *P. expansum*. We sequenced and assembled the genome of a *P. italicum *strain, which contains 31,034,623 bp with 361 scaffolds and 627 contigs. The mechanisms underlying the evolution of host specificity among the analyzed *Penicillium *species were associated with the expansion of protein families, genome restructuring, horizontal gene transfer, and positive selection pressure. A dual-transcriptome analysis following the infection of Valencia orange *(Citrus sinensis)* by *P. italicum *resulted in the annotation of 9,307 *P. italicum* genes and 24,591 Valencia orange genes. The pathogenicity of* P. italicum *may be due to the activation of effectors, including 51 small secreted cysteine-rich proteins, 110 carbohydrate-active enzymes, and 12 G protein-coupled receptors. Additionally, 211 metabolites related to the interactions between* P. italicum *and Valencia orange were identified by gas chromatography-time of flight mass spectrography, three of which were further confirmed by ultra-high performance liquid chromatography triple quadrupole mass spectrometry. A metabolomics analysis indicated that *P. italicum *pathogenicity is associated with the sphingolipid and salicylic acid signaling pathways. Moreover, a correlation analysis between the metabolite contents and gene expression levels suggested that* P. italicum *induces carbohydrate metabolism in Valencia orange fruits as part of its infection strategy. This study provides useful information regarding the genomic determinants that drive the evolution of host specificity in* Penicillium *species and clarifies the host-plant specificity during the infection of Valencia orange by *P. italicum*.

**IMPORTANCE:**

P. italicum GL_Gan1, a local strain in Guangzhou, China, was sequenced. Comparison of the genome of *P. italicum* GL_Gan1 with other pathogenic *Penicillium* species, *P. digitatum* and *P. expansum*, revealed that the expansion of protein families, genome restructuring, HGT, and positive selection pressure were related to the host range expansion of the analyzed *Penicillium* species. Moreover, gene gains or losses might be associated with the speciation of these *Penicillium* species. In addition, the molecular basis of host-plant specificity during the infection of Valencia orange (*Citrus sinensis*) by *P. italicum* was also elucidated by transcriptomic and metabolomics analysis. The data presented herein may be useful for further elucidating the molecular basis of the evolution of host specificity of *Penicillium* species and for illustrating the host-plant specificity during the infection of Valencia orange by *P. italicum*.

## Introduction

Host specificity refers to the ability of an organism to colonize another organism [1]. Some plant pathogens have a broad host range, whereas others can only colonize specific plant species or families. Even within the same genus, there are considerable differences in the host range among species, which may be related to pathogen evolution. During the evolution of plant pathogens, host shifts may occur to broaden or limit the host range [2]. In extreme cases, a pathogen evolves to colonize a new host that is phylogenetically distant from the original hosts (i.e., host jump), possibly resulting in the emergence of new fungal diseases [3,4]. Therefore, elucidating the molecular basis of the evolution of host specificity in fungal plant pathogens is critical for developing strategies to decrease the yield losses of economically valuable crops.

The genus *Penicillium* comprises ascomycetous fungal species that are widely distributed, with important implications for natural environments as well as in the food and drug industries. Some members of this genus produce penicillin, an antibiotic, whereas other species are used to make cheese. However, some *Penicillium* species are among the most important plant pathogens responsible for postharvest diseases. For example, *Penicillium italicum, Penicillium digitatum*, and *Penicillium expansum* cause losses of up to 10% of harvested crops. Of these species, *P. italicum* and *P. digitatum* exclusively infect citrus fruits, but *P. expansum* can infect diverse fruit and vegetable crops, such as apple, pear, peach, strawberry, and tomato, but not citrus fruits [5]. The genomes of *P. italicum, P. digitatum*, and *P. expansum* strains have been sequenced [6–9]. Additionally, Li et al. [8] and Ballester et al. [7] focused on the relationship between secondary metabolism and infections in *P. expansum*. However, the pathogenicity and evolution of host specificity in *Penicillium* species have remained largely uncharacterized.

The evolution of host specificity of fungi is mediated by a complex process involving multiple genes and mechanisms. A multidisciplinary approach may unveil new molecular determinants that drive the evolution of host specificity. In this study, we sequenced the genome of a *P. italicum* strain and then applied comparative genomics, dual-transcriptomics, and metabolomics analyzes to uncover the genomic determinants associated with the evolution of *Penicillium* species and elucidate the molecular mechanism underlying the host-plant specificity during the infection of *Citrus sinensis* by *P. italicum*. The results presented herein provide the molecular basis for clarifying the genomic determinants that drive the evolution of host specificity of *Penicillium* species and the host-plant specificity during the infection of Valencia orange by *P. italicum*.

**Figure 1.**
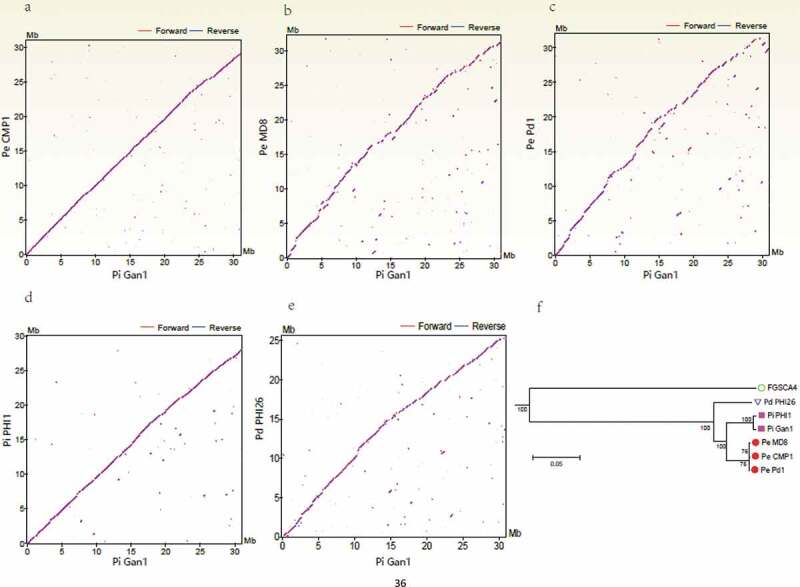
Comparison of the *Penicillium italicum, P. digitatum*, and *P. expansum* genomes. (a – e) Dot-plot analysis of the pairwise alignments of the *P. italicum* GL_Gan1 genome and the genomes of the other analyzed *Penicillium* species. (f) A phylogenetic tree revealing the relationships among the following *Penicillium* species and strains: *P. italicum* (Gan1 and PHI1), *P. expansum* (CMP1, MD8, and Pd1), and *P. digitatum* (PHI26).

## Results and Discussion

### *Penicillium italicum* genome sequencing and annotation

An Illumina HiSeq 2000 sequencing platform was used to generate 1,815 Mb of raw data for the *P. italicum* strain GL_Gan1 genome. The assembled genome contained 31,034,623 bp, with an average GC content of 45.83%, including 361 scaffolds ranging from 1,002 bp to 994,651 bp and 627 contigs ranging from 213 bp to 867,867 bp. The N50 values of the scaffolds and contigs were 316.59 kb and 196.54 kb, respectively (Table 1), which were considerably higher than those of the released genomes of *P. italicum* B3 and *P. italicum* PITC [7,8]. Moreover, the *P. italicum* GL_Gan1 genome comprised 9,447 genes, which consisted of 15,524,911 bp, including 13,960,971 bp of exons and 1,563,940 bp of introns. The repeat sequence contained 3,791,084 bp. The predicted genes included 7,136 core genes and 85 species-specific genes (Dataset S1), which represented 75.54% and 0.90% of the total number of genes, respectively. The genes were functionally annotated with the Gene ontology (GO), Kyoto Encyclopedia of Genes and Genomes (KEGG), and Clusters of Orthologous Groups (COG) databases (Fig. S1). The top matches for the GO, KEGG, and COG databases included 3,350 (35.46%), 1,172 (12.41%), and 1,447 (15.31%) genes, respectively, and the most abundant assigned terms were ‘metabolic process’, ‘xenobiotic bio-degradation and metabolism’, and “general function prediction only”, respectively (Fig. S1).

**Table 1.** *Penicillium italicum* GL_Gan1 genome properties compared with those of other sequenced *P. italicum* strains.

Genome specifics		P. italicum GL_Gan1	P. italicum PHI1/PITC	P. italicum B3
Source		Decayed mandarin, Guangzhou, china	Decayed mandarin, Spain	Decayed mandarin, Wuhan, China
Assembly statistics				
	Genome size (Mb)	31.03	32.08	28.99
	Total number of contigs	627	1792	4873
	Total number of scaffolds (>1Kb)	361	788	640
	Average base coverage (X)	58	421	193
	N50 contig (Kb)	196.54	55.51	161.33
	N50 scaffold (Kb)	316.59	122.13	205.92
	GC content (%)	45.83	47.45	47.03
Coding sequence				
	Average gene density (gens/Kb)	0.30	0.31	0.32
	Protein – coding genes	9447	9995	9369
	Mean gene length (bp)	1643	1589	1648
	Exons/gene	3	3	3
	Mean exon size (bp)	483	482	490
	Mean intron size (bp)	80	80	82
	Min protein length (aa)	35	29	50
	Max protein length (aa)	6490	7287	7288
	Repetitive elements (%)	12.22	-	1.80
	NCBI accession	LWEC00000000	JQGA00000000.1	JMDK00000000

Horizontal gene transfer (HGT) is a major evolutionary factor that enables organisms to quickly acquire new genes that enhance environmental adaptation and survival. In this study, we identified 383 transferred genes in the *P. italicum* GL_Gan1 genome (**Dataset S1**). Regarding the functional annotation with the COG database (Dataset S1), the top three categories were secondary metabolite biosynthesis, transport, and catabolism (49 proteins), amino acid transport and metabolism (48 proteins), and energy production and conversion (44 proteins). Similarly, the top categories for the functional annotation with the GO database (**Dataset S1**) were metabolic process (294 genes) and catalytic activity (291 genes). Interestingly, the most enriched KEGG pathways (**Dataset S1**) were xenobiotics biodegradation and metabolism (137 genes), amino acid metabolism (104 genes), and carbohydrate metabolism (97 genes). These results suggested that genes associated with metabolic processes and/or products are prone to be horizontally transferred [10]. Moreover, GL_Gan1_GLEAN_10000555 encodes a polyketide synthase (PKS) with two active domains (MDR and Qor). This PKS is a core enzyme responsible for catalyzing the first reaction during the biosynthesis of secondary metabolites (SMs) [11]. We constructed a phylogenetic tree based on PKS sequences from *Aspergillaceae*, Bacteria, *Glomerellaceae, Nectriaceae, Clavicipitaceae*, and so on. There were three major clades, *Polyporaceae* and bacteria, *Aspergillaceae* and others. Surprisingly, when PKS from seven Bacteria genes and seven *Penicillium* genes were constructed a phylogenetic tress, it was found that *Paenibacillus mucilaginosus* gene was clustered with *Penicillium* genes (Fig. S2). *Paenibacillus mucilaginosus* is able to promote crop growth and widely used as microbial fertilizer in China [12]. Possibly, horizontal gene transfer occurred for *P. italicum* PKS gene from *Paenibacillus mucilaginosus*, however, further experiment is required to verify the hyposthesis. A similar HGT was reported for the phytohormone biosynthetic genes in *Fusarium* species [13].

Plants interact with pathogens through microbial effectors and host immune receptors. The successful colonization of a plant pathogen is the result of the interaction between pathogen effectors and host immune receptors. Pathogen effectors repress the host immune response to facilitate pathogen growth and colonization, which are critical for the pathogenicity of filamentous fungi. Sanchez-Vallet et al. proposed that some small-size peptides for secretion, cell wall-degrading enzymes, protease inhibitors, interactors with the ubiquitin-proteasome system, and disruptors of the hormone signaling pathway can act as effectors [14]. It has been reported that some fungal effector proteins identified are relatively small protein containing fewer than 200 amino acids and more than 2% cysteines, i.e. small secreted cysteine-rich proteins (SSCPs), constituting a common source of fungal effectors [15]. In the present study, a total of 444 putative effector genes were identified in the *P. italicum* GL_Gan1 genome, of which 51 genes encoding SSCPs (Dataset S1) possibly play roles in trigger resistance or susceptibility in citrus fruit [16].

Plant cell wall is a complex matrix of polysaccharides, including pectin, hemicellulose and cellulose, which provides mechanical support for cells and forms a barrier against fungal invasion. Plant pathogens are equipped with multiple carbohydrate-active enzymes (CAZymes), including glycoside hydrolases (GHs), glycosyl transferases (GTs), polysaccharide lyases (PLs), carbohydrate esterases (CEs) and carbohydrate-binding modules (CBMs), which facilitate fungal invasion via degradation or modification of plant cell walls [17,18]. *P. italicum* is a necrotrophic pathogen and only infects citrus fruit through peel injuries in the field, the packing house, or during commercialization chains [6]. Initiative infection of *P. italicum* is usually seen only after about 3 d of incubation at room temperature, with water-soaked and soft peel. Therefore, hydrolytic enzymes produced by *P. italicum* appear responsible for the maceration of the tissue during disease development [19]. In the present study, 1,290 genes encoding CAZymes were identified in the *P. italicum* genome, including 18 PLs, 383 GTs, 623 GHs, 141 CEs, and 274 CBMs, which was much more than that observed in the other published and annotated *P. italicum* genome [8]. The number of CAZymes in *P. italicum* was similar to that of the necrotrophic fungi *Fusarium oxysporum* (approximately 1200), but much more than those of saprotrophic fungi, such as *Chaetomium thermophilum var. thermophilum* DSM 1495 and *Neurospora crassa* OR74A (less than 600) [17]. We also noticed that GHs were the most abundant CAZymes in *P. italicum* genome, of which some subgroups, including GH76, GH78, GH92, GH3, GH16, GH51, GH55, GH43, GH5, GH2, GH17, GH71, were involved in degradation of cell wall polysaccharides (www.cazy.org). The distribution of CAZymes in *P. italicum* was similar with other necrotrophic fungi, such as *Fusarium oxysporum, Fusarium verticillioides, Nectria haematococca, Alternaria brassicicola, Parastagonospora nodorum*, and *Corynespora cassiicola* [17].

### Comparative genomics analysis reveals the evolution of *Penicillium* species

To elucidate the evolution of host specificity and the pathogenicity of *Penicillium* species, the genomes of two *P. italicum* strains (GL_Gan1 and PHI1/PITC), one *P. digitatum* strain (PHI26), and three *P. expansum* strains (CMP1, Pd1, and MD8) were compared. A genome wide analysis of synteny showed that Pe_CMP1/Pi_Gan1 (Figure 1a) and Pi_PHI1/Pi_Gan1 (Figure 1d) had high levels of synteny, followed by Pd_PHI26/Pi_Gan1 (Figure 1e). A total of 7136 single-copy core genes were used to constructed phylogenetic tree (figure 1f), with *Aspergillus nidulans*_FGSCA4 as an outgroup species, to further elucidate the evolutionary relationships among *P. italicum, P. expansum*, and *P. digitatum*. The results showed that *P. italicum* Gan1 and PHI1 were cluster into one group, while *P. expansum* CMP1, Pd1, and MD8 were cluster into another group. Moreover, the phylogenetic distance between *P. italicum* Gan1 and PHI1 was shorter than those between PHI26 and *P. expansum* CMP1, Pd1, and MD8. The inconsistence between synteny and phylogenetic tree might be related to the application of genome and single-copy core genes for comparative analysis, respectively. However, the comparative analysis of core genome showed the close relationship between *P. italicum* and *P. expansum*. Our results were consistent with the species tree reconstructed using 524 single-copy genes present in seven *Penicillium* species [7] and 2,134 single-copy genes present in eight *Penicillium* species [20].

The comparative genomics analysis demonstrated the evolutionary plasticity of these *Penicillium* genomes. The core genomes comprised 7,690 genes (*P. digitatum*), 8,375 genes (mean) (*P. italicum*), and 9,342 genes (mean) (*P. expansum*) (Table 2), suggesting that lineage-specific gene expansion was essential for *P. expansum* evolution and its adaptations to diverse hosts. Similar results were reported by Yoshida et al. [21] who proposed that gene gains and losses are two of the primary contributors to the genome shift essential for species evolution.

**Table 2.** Core and strain-specific genes in the *Penicillium italicum, P. digitatum*, and *P. expansum* genomes.

Species	Core	Specific
*P. expansum* CMP1	9212	5
*P. expansum* MD8	9417	19
*P. expansum* Pd1	9397	15
*P.italicum* Gan1	7136	85
*P.italicum* PHI1	8393	467
*P. digitatum* PHI26	7690	456

The expansion of protein families associated with fungal pathogenicity was speculated to force the speciation of these *Penicillium* species. Table 3 presents the differences in the protein content among *Penicillium* species. 6,309 and 6851 protein families were detected in *P. digitatum* and *P. italicum*, respectively, whereas 7,640 protein families were detected in *P. expansum*, implying that the expansion of protein families has occurred from *P. digitatum* and *P. italicum* to *P. expansum*, which might be related to the fungal pathogenicity of the three *Penicillium* species.

**Table 3.** Comparison of protein family number associated with fungal pathogenicity among *Penicillium* species.

Type of protein	*P. italicum* GL_Gan1	*P. italicum* PHI1/PITC		Mean No. of *P. italicum*	*P. digitatum* _PHI26	*P. expansum* _CMP1	*P. expansum* _Pd1	*P.expansum* _MD8	Mean No. of *P. expansum*
Total proteins	9,447	9,995		9,721	9,133	10,663	11,023	11,060	10,915
Protein families	6,815	6,887		6,851	6,309	7,411	7,640	7,640	7,564
Secreted proteins	444	457		451	377	576	601	607	595
SSCPs	51	62		57	61	70	70	69	70
PHI proteins	1,728	1,728		1,728	1,503	2,155	2,242	2,245	2,214
CAZyme	1,290	1,306		1,298	1,162	1,616	1,686	1,676	1,659
CE8/CE12	2/6	2/5		7.5	3/5	3/12	3/13	3/13	15.7
GH28/GH78/GH95/GH105	17/56/3/3	20/57/3/3		81	33/36/3/1	24/93/3/1	24/96/3/1	25/95/3/1	123
PL1/PL3/PL4	6/1/9	6/1/10		16.5	6/1/6	6/1/7	6/1/8	6/1/8	15
GPCR	12	11		12	8	9	9	9	9
P450	30	28		29	12	41	44	44	43
SM cluster	42		43	43	32	63	65	67	65

SSCPs, small secreted cysteine-rich protein; PHI proteins, pathogen-host interaction proteins; CAZyme, carbohydrate active enzyme; GH, glycoside hydrolase; PL, Polysaccharide lyases; P450, Cytochromes P450; GPCR, G protein-coupled receptor; SM clusters, secondary metabolite biosynthetic gene cluster

Pathogen-host interaction (PHI) are crucial for initiating the infections of a host by a pathogen, which is regulated by pathogenesis-related secreted proteins, such as CAZymes and SSCPs [22]. PHI-base database (http://www.phibase.org/) is to provide expertly curated molecular and biological information on genes proven to affect the outcome of pathogen-host interactions. In the present study, searching against the PHI database identified 1728 putative PHI genes in the *P. italicum* GL_Gan1 genome which were homologous genes involved in pathogenicity in other fungi. A comparison of the number of PHI genes revealed that *P. digitatum* (average 1,503) has fewer PHI genes than *P. expansum* (average 2,214) and *P. italicum* (average 1,728) (Table 3). Furthermore, *P. italicum, P. digitatum* and *P. expansum* shared common subgroups (399) with differential numbers of PHI genes in some subgroups. Moreover, each species had its own specific PHI subgroups (Dataset S2). Possibly, the differential compositions and number in PHI genes among three *Penicillium* species were related to the different host ranges.

**Figure 2.**
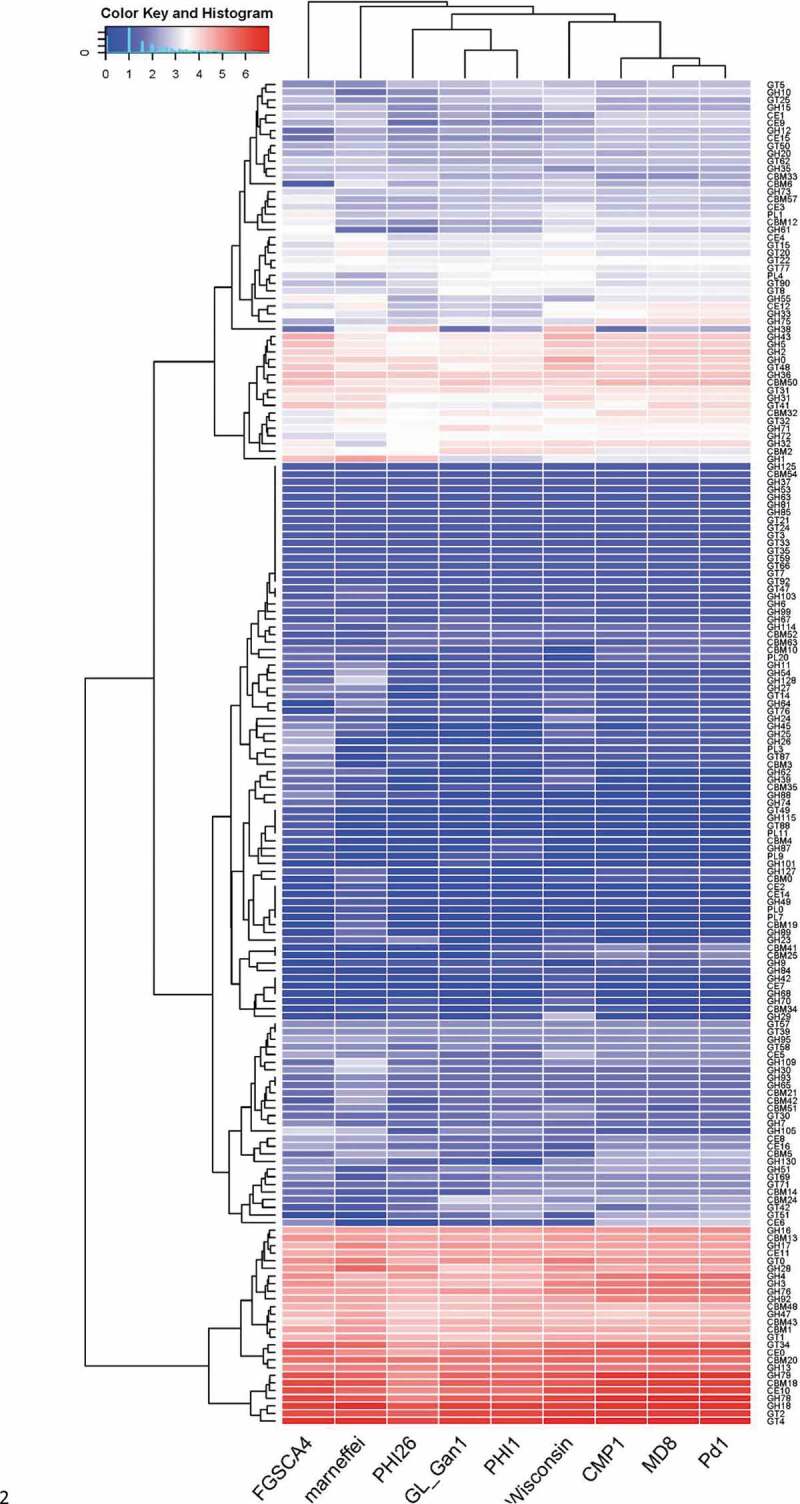
Heat map of CAZyme genes reveals the evolutionary relationships among *Penicillium italicum, P. digitatum*, and *P. expansum*. Several other fungi, *Aspergillus nidulans_*FGSCA4, *Penicillium rubens* Wisconsin 54–1255 and *Penicillium marneffei*, also were included in the heat map.

CAZymens play important role in the invasion of pathogens on host plants by degrading cell wall polysaccharides, such as cellulose, hemicellulose and pectin [17]. In pathogens, GTs are mainly involved in carbohydrate assembly, while GHs, CEs and PLs are cell wall degradation-related enzymes [23]. Among *P. italicum, P. expansum*, and *P. digitatum, P. expansum* has the most CAZymes, followed by *P. italicum* (Table 3). We constructed a heat map for the different CAZyme subgroups to further elucidate the evolutionary relationships among *P. italicum, P. expansum*, and *P. digitatum*. There were no obvious differences in numbers in most of the CAZyme subgroups among the three analyzed species. However, significant differences were detected for the subgroups involving GH76, GH78, GH79, GH92, GH3, GH16, GH51, GH55, GH32, GH75, GH43, GH5, GH2, CE10, CE9, CE15, GT34, CBM18, CBM13, CBM12, CBM50 (Figure 2), with *P. expansum* harboring more of these families than *P. italicum* and *P. digitatum*. GH92 s and GH76 s are exo-acting α-mannosidases and endo-acting α-mannanases, respectively [24,25], while GH3s and GH43 s are xylan degradation-related enzymes (www.cazy.org), which could together play important roles in degrading hemicellulose and softening host tissues. The majority of GH16, GH51, GH55 and GH5 CAZymes are endoglucanase, lichenase/endo-β-1,3–1,4-glucanase, endo-β-1,4-glucanase, xyloglucanase, endo-β-1,4-xylanase, glucan 1,3-β-glucosidase, glucan endo-1,3-β-D-glucosidase, endo-β-1,4-xylanase, β-glucosidase (www.cazy.org), which are important enzymes involving in the degradation of cellulose and hemicellulose. GH78 s are rhamnogalacturonan α-L-rhamnohydrolases that could also participate in host cell wall degradation [23]. Lipase is an emerging research field on virulence mechanisms in fungi. CE10 s belong to lipases which hydrolyze carboxyl ester bonds on triacylglycerols, and may act against host morpho-anatomical barriers (wax and cuticle) and be involved in fungal pathogenesis [17,23]. CE15 s are 4-O-methyl-glucuronoyl methylesterase (Glucuronoyl esterase), which is considered to disconnect hemicellulose from lignin through the hydrolysis of the ester bond between 4-O-methyl-D-glucuronic acid residues of glucuronoxylans and aromatic alcohols of lignin [26]. The greater amount of cell wall polysaccharide degradation-related enzymes in *P. expansum* might be beneficial for degradation of cell walls and also implied greater pathogenicity.

Small secreted cysteine-rich proteins (SSCPs) are important fungal effectors that trigger resistance or susceptibility in specific host plants [15]. These proteins can recognize the products of plant *R* genes and are related to disease symptom development and fungal pathogenicity. The *P. expansum* genome carries an average of 70 SSCP genes, whereas the *P. italicum* (average 57) and *P. digitatum* (average 61) genomes consist of fewer SSCP genes.

G protein-coupled receptors (GPCRs) are seven-transmembrane domain-containing receptors that transduce extracellular signals and activate intracellular responses [27]. They have important functions related to fungal growth and survival. The number of GPCRs varies depending on the fungal species. For example, the *Sporisorium scitamineum* genome encodes only six GPCRs, whereas the *Magnaporthe grisea* genome includes 61 GPCR genes [28,29]. *Penicillium* species have a few GPCRs, and no GPCR gene expansion was detected during evolution.

Cytochrome P450 s (P450 s) belong to a superfamily of mono-oxygenases, which are distributed in a wide range of organisms. The diversity in the number of P450 genes and families among organisms reflects an evolutionary driving force toward speciation [30]. Compared with the number of P450 genes in *P. italicum* (average 29) and *P. digitatum* (average 12), a marked gene expansion occurred in *P. expansum* (average 43).

Secondary metabolites are necessary for fungi to produce a variety of chemical compounds, including toxins and antibiotics, which contribute to fungal virulence, host specificity, and adaptations to ecological niches [31]. The specialist *P. digitatum* has fewer SM biosynthetic gene clusters (average 32) than *P. italicum* (average 43) and *P. expansum* (average 65). Recently Wu et al. (2019) reported the similar composition of SM gene clusters in different *Penicillium* species, but further functional characterization of all these genes are required to be exactly identified by RNA interference and/or gene deletion studies [32].

Therefore, protein-family expansions in these *Penicillium* lineages were possibly associated with the evolutionary processes in these species. It seems that *P. italicum* is a transitional species in the *Penicillium* lineage that developed during the evolution of *P. digitatum* to *P. expansum*. Thus, *P. expansum* may be a newer species than *P. italicum*. This is consistent with the principle of speciation that suggests the newly evolved *P. expansum* should have a broader host range than the older related species. Additionally, our data are consistent with those of previous studies on seven *Metarhizium* species and three *Fusarium* species, which indicated that the genome and protein families expanded considerably in the transitional and generalist species [33,34]. Alternatively, these *Penicillium* species may have undergone a parallel evolution, but the divergence of *P. italicum* and *P. digitatum* occurred because of gene losses, particularly the loss of genes associated with virulence and metabolic products, resulting in major changes to *P. italicum* and *P. digitatum* that enabled them to infect new hosts. Accordingly, these two species may have been exposed to extreme external pressures that led to a rapid evolution of effectors for their colonization of a new host species. Similarly, a loss of genes associated with virulence and toxin biosynthesis in host-specific strains of *Metarhizium* species resulted in the divergence or rapid evolution of specialist *Metarhizium* species [35]. Moreover, gene losses rather than gains occurred in *Melanopsichium pennsylvanicum*, which expanded its host range from monocot to dicot hosts [36].

Genome restructuring may have contributed to the evolution of host specificity. Transposable elements (TE) cause gene mutations and the evolution of genomes [37]. We detected considerably more TEs in *P. digitatum* and *P. italicum* than in *P. expansum* (Table S1), implying that *P. expansum* has fewer mutations than *P. digitatum* and *P. italicum* after the reinforcement of reproductive isolation. Moreover, the plasticity of the *P. digitatum* and *P. italicum* genomes was greater than that of the *P. expansum* genome.

Using Ka/Ks > 1 and P < 0.01 as criteria, we detected 17 genes under positive selection pressure among the examined *Penicillium* species. Positively selected genes, most of which are related to fungal virulence (Dataset S1), were frequently detected between *P. italicum* and *P. expansum*, rather than in *P. italicum* itself or between *P. italicum* and *P. digitatum*. These results imply that these genes may have contributed to the divergence of the host specificity between *P. italicum* and *P. expansum*, thereby providing important evidence of the evolutionary processes of these *Penicillium* species as well as the adaptation of *P. expansum* to various hosts.

### Dual-transcriptome analysis of *P. italicum* GL_Gan1 and Valencia orange during colonization

**Figure 3.**
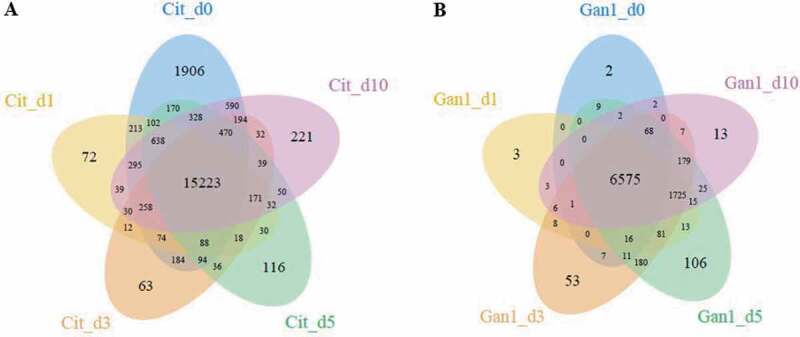
Venn diagram presenting the number of genes expressed in Valencia orange (a) and *P. italicum* (b) on days 0, 1, 3, 5, and 10 after the inoculation.

To explore the mechanism underlying the colonization and pathogenicity of *P. italicum* on *Citrus sinensis*, the transcriptome profiles of Valencia orange and *P. italicum* GL_Gan1 during the colonization were analyzed. We constructed RNA-sequencing (RNA-seq) libraries for the inoculated Valencia orange at 0, 1, 3, 5, and 10 days post-inoculation (dpi). The libraries were sequenced with the Illumina RNA-seq technology, and more than 20 M reads were generated for each library (Table S2). Additionally, 0.61%–57.27% of the clean reads were mapped to the *P. italicum* genome, in which 6,840–9,145 expressed genes were detected. Moreover, 12.07%–82.82% of the clean reads were mapped to the *C. sinensis* genome, in which 17,610–21,896 expressed genes were detected (Table S2). Furthermore, 6575 and 15,223 genes were expressed at all stages in *P. italicum* and Valencia orange, respectively (Figure 3). Compared with the expression levels at 0 dpi, 228, 414, 376, and 336 genes in *P. italicum* and 301, 1,314, 1,955, and 479 genes in Valencia orange were differentially expressed at 1, 3, 5, and 10 dpi, respectively.

**Figure 4.**
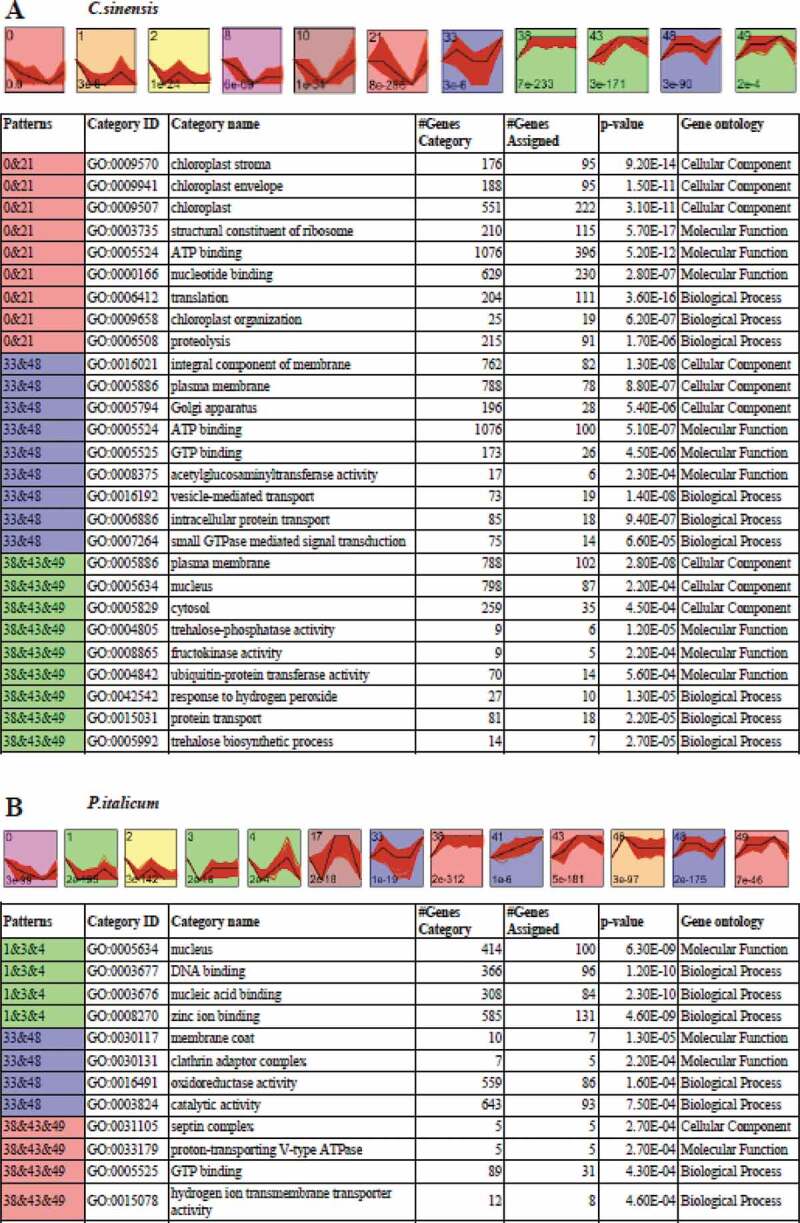
STEM clusters of gene expression profiles of *P. italicum* and *C. sinensis* during the colonization of *P. italicum* in *C. sinensis*. (a) Top panel: eleven representative temporal gene expression patterns were identified in *C. sinensis*; Colored backgrounds were used to differentiate different possible model profiles recognized by STEM, with the number of clustered genes indicated in the top left corner, the bottom left presents the p-value of number of genes assigned versus expected. Bottom panel: GO enrichment of three clusters of expression patterns genes category with top three of ontology, genes category, genes assigned and the significance (p-value<0.001) were listed. (b) Top panel: thirteen representative temporal gene expression patterns were identified in *P. italicum*; Colored backgrounds were used to differentiate different possible model profiles recognized by STEM, with the number of clustered genes indicated in the top left corner, the bottom left presents the p-value of number of genes assigned versus expected. Bottom panel: GO enrichment of three clusters of expression patterns genes category with top three of ontology, genes category, genes assigned and the significance (p-value<0.001) were listed.

A short time-series expression miner (STEM) clustering analysis of gene expression during the infection of Valencia orange by *P. italicum* revealed that the fruit responses to *P. italicum* included cell wall and membrane degradation, accelerated polysaccharide metabolism and energy transformation, and increased metabolite production (Figure 4a). The genes associated with cellular components that exhibited the same temporal expression patterns were clustered together. Among these genes, the expression levels of the genes associated with the chloroplast, including stroma (95 assigned genes), envelope (95 assigned genes), thylakoid (17 assigned genes), and organization (19 assigned genes), were significantly down-regulated, suggesting that the chloroplast of the infected Valencia orange may have degraded or was otherwise adversely affected. Similarly, the expression levels of many genes related to protein synthesis, including the structural constituents of ribosomes (115 assigned genes) and translation (111 assigned genes), were down-regulated in the inoculated Valencia orange, indicating the infection impeded protein synthesis (Figure 4a). Additionally, glucosyltransferases and the related metabolism may be activated in Valencia orange. The expression levels of five of nine genes associated with fructokinase activity were up-regulated. It is possible that the growth of *P. italicum* exhibits a preference for glucose rather than fructose as an energy source. The impact of fungal pathogen on plant host transcriptome have widely revealed the altered gene expression patterns involved in defense and stress responses, cell wall structure, chloroplast biogenesis, carbon metabolism and transportation, and biosynthesis of secondary metabolites [38,39].

**Figure 5.**
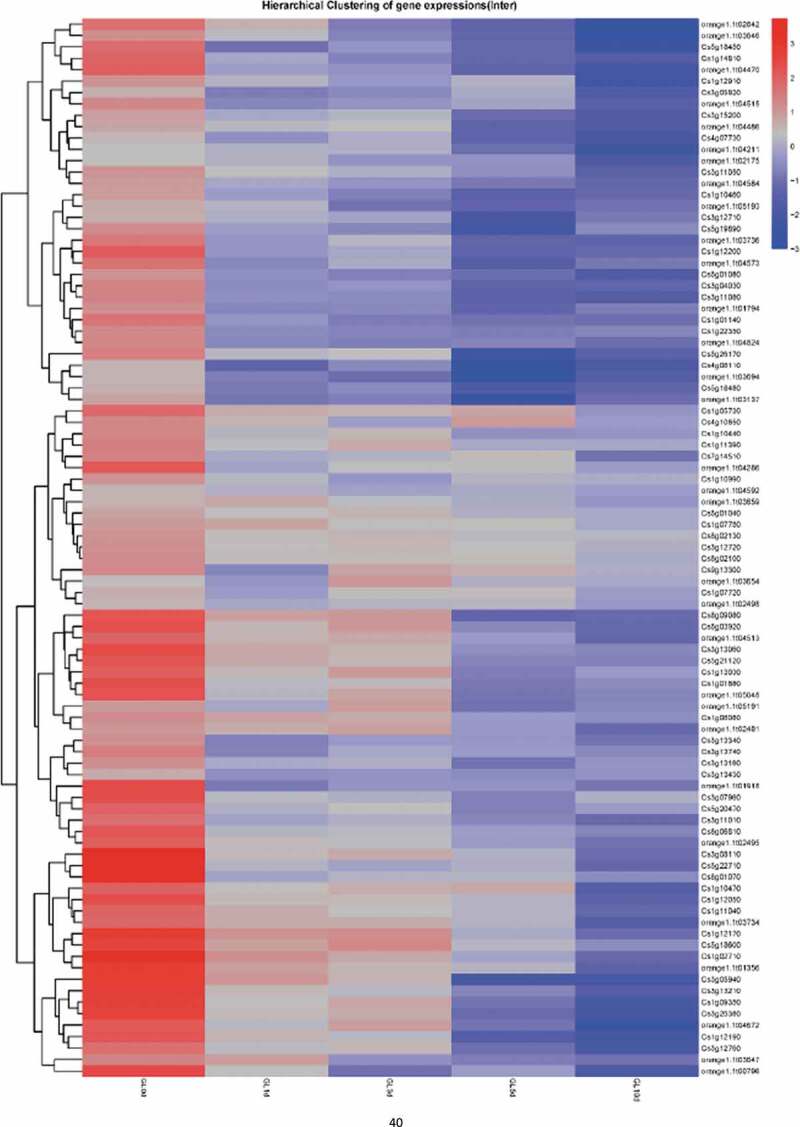
Heat map for the expression profiles of Valencia orange *R* genes during the infection by *P. italicum*. Blue and red bars represent low and high expression levels based on the number of transcripts, respectively.

**Figure 6.**
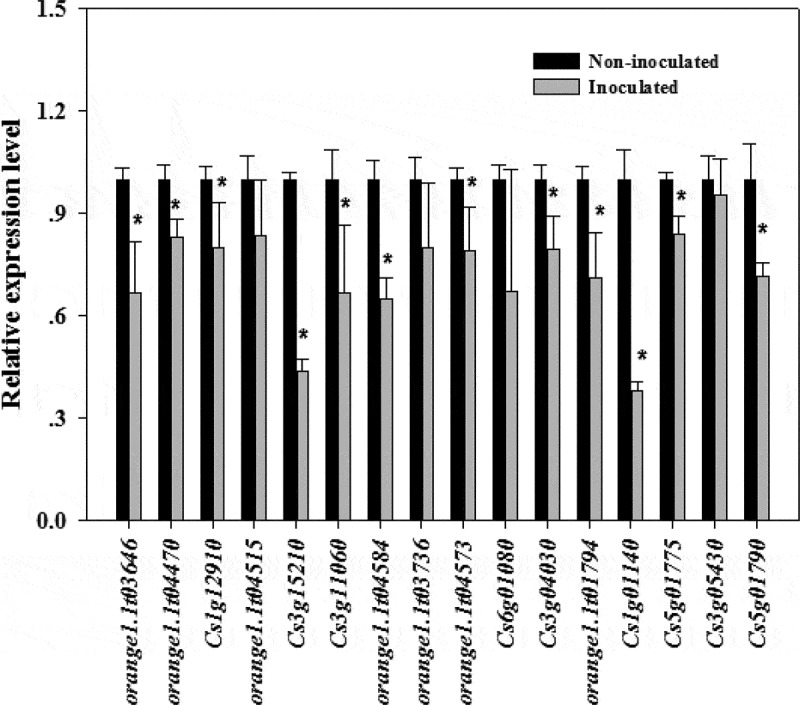
Comparison of the expression levels of *R* genes in *P. italicum*-infected and healthy peel of Valencia orange at 3 dpi. * indicates significant difference (p < 0.05).

Plants have evolved sophisticated strategies to escape various pathogen attacks. Plant resistance genes (*R* genes) are important for resisting pathogen infections. These genes are structurally conserved in vertebrates and plants, with the following typical domains: NBS, LRR, TIR, and CC. A total of 636 *R* genes were identified in the Valencia orange genome (Table S3). The expression of most of these genes was down-regulated during infection (Figure 5). We randomly selected 16 down-regulated *R* genes from the transcriptome data and compared their expression in non-inoculated and inoculated Valencia orange. Of the 16 genes, the expression of 12 was significantly lower in the inoculated Valencia orange than in the non-inoculated fruit at 3 dpi (Figure 6). These results indicated that the *P. italicum* infection adversely affected *R* gene-based immunity. Houterman et al. [40] reported that *Fusarium oxysporum* secretes an effector, Avr1, which can induce and suppress *R* gene-based immunity in infected tomato plants. This effector activates the disease resistance of tomato plants via *R* genes (*I* or *I-1*), but suppresses the protective effects of two other *R* genes, *I-2* and *I-3*.

Our STEM clustering analysis also confirmed that *P. italicum* exhibited accelerated growth and development, as the expression levels of all five genes associated with a cell division control-related protein were up-regulated during infection. Similarly, many genes related to ATP hydrolysis also exhibited up-regulated expression (Figure 4b). Therefore, *P. italicum* may be very actively absorbing nutrients, leading to accelerated growth.

**Figure 7.**
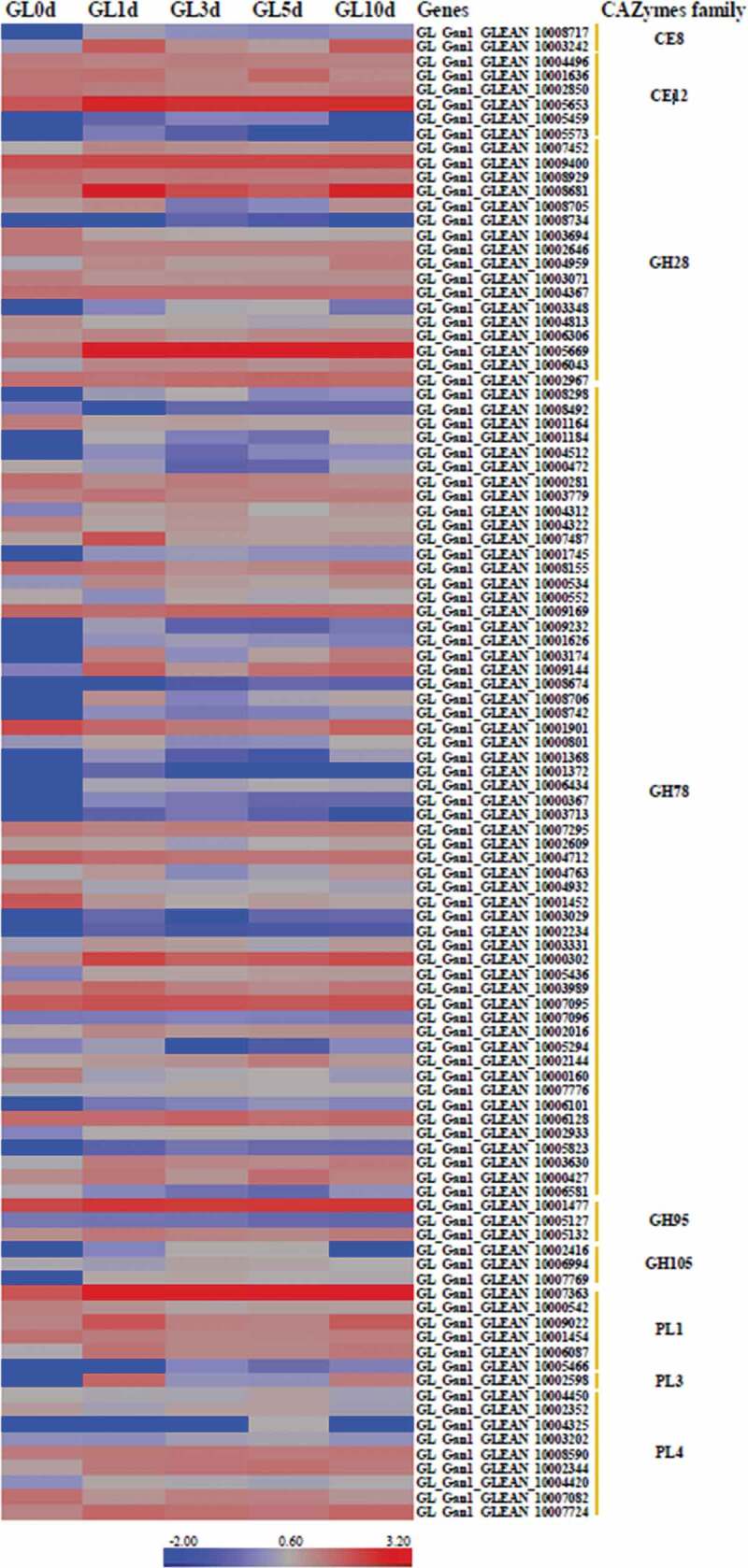
CAZyme genes and their expression levels during the infection of Valencia orange by *P. italicum* GL_Gan1.

*P. italicum* secretes many proteins and substances that enable it to infect and colonize *Citrus sinensis*, such as CAZymes, SSCPs, GPCR, and SMs. The CAZymes degrade plant cell walls to create an entry point in the plant host. In the present study, 110 genes encoding CAZymes were differentially expressed in *P. italicum* during infection, including genes belonging to the GH28, GH78, GH95, GH105, CE8, CE12, PL1, PL3, and PL4 families. These CAZymes help degrade the pectin, hemicellulose, and cellulose in plant cell walls. Specifically, the expression levels of several genes encoding CAZymes, including GL_Ganl_CLEAN_10003242, GL_Ganl_CLEAN_10005653, GL_Ganl_CLEAN_10005681, GL_Ganl_CLEAN _10005669, GL_Ganl_CLEAN_10000302, and GL_Ganl_CLEAN_10007363, were substantially up-regulated in *P. italicum* during the infection of Valencia orange (Figure 7). Thus, these genes may be critical for the infection. Ballester et al. [7] reported that a large number of gene encoding cell wall-degrading enzymes including polygalacturonase, pectate lyase, glycosyl hydrolase and xylanase, are obviously up-regulated in *P. expansum* when infecting apple fruit at 2–3 dpi. The SSCPs represent a common source of fungal effectors [41]. In the current study, 51 genes encoding SSCPs were differentially expressed in *P. italicum* during infection (Dataset S3). The expression of a number of SSCP genes was down-regulated in *P. italicum* during infection. Additionally, 15 genes encoding SSCPs were not expressed at 0 dpi, but were expressed at 1 or 3 dpi. Moreover, the expression levels of four SSCP genes (GL_Gan1_GLEAN_10002725, GL_Gan1_GLEAN_10004237, GL_Gan1_GLEAN _10005638, and GL_Gan1_GLEAN_10001595) increased considerably during the early infection stage, implying these SSCPs influence *P. italicum* pathogenicity. Similarly, Bradshaw et al. [42] examined the gene expression dynamics of *Dothistroma septosporum* when it invades its host, and revealed the up-regulated expression of genes mainly encoding SSCPs, CAZymes, and SMs in three infection cycle stages. Alkan et al. [43] simultaneously analyzed the transcriptomes of *Colletotrichum gloeosporioides* and tomato fruits during their interaction, and determined that many SSCP genes are expressed in different stages. Overall, the RNA-seq results highlighted the arsenal of putative pathogenicity factors and identified specific candidates that should be functionally analyzed to further characterize their effect on *P. italicum* pathogenicity.

### Metabolomics analysis of non-infected and infected Valencia orange by *P. italicum*

We used GC-TOF-MS to analyze the metabolome of the cuticle tissues in non-infected and infected Valencia orange fruits at 3 dpi. A total of 592 metabolites were identified and quantified, of which 207 metabolites had the similarity over 700, including sugars, polyols, organic acids, amino acids, and SMs (Table S4). The abundance of 51 metabolites was significantly different between the inoculated and non-inoculated Valencia orange fruits (Table 4). Most of the differentially accumulated metabolites are related to carbohydrate metabolism, amino acid metabolism, fatty acid metabolism, secondary substance metabolism, and nucleic acid metabolism.

**Table 4.** Identification of differentially accumulated metabolites (p < 0.05) between non-infected and infected Valencia orange fruits.

Metabolite name	RT^a^	Mass	Similarity^b^	VIP^c^	P-value	Fold change^d^
Hydroxylamine	7.95	133	742.8	1.07	2.71E-05	2.757
Benzoic acid	9.95	179	964.3	2.31	8.75E-08	2.016
Ethanolamine	10.1	86	916.9	1.8	8.28E-05	0.909
Glycine	10.6	174	949.8	1.09	0.0002	1.157
Phosphate	10.15	205	809.6	3.96	0.034	0.112
Glycerol	10.16	117	844.3	7.43	2.43E-11	0.041
2-Deoxyerythritol	10.37	117	908.7	1.23	0.005	0.102
Threonine	10.48	117	892.5	1.26	8.82E-05	1.966
Succinic acid	10.74	56	900.1	1.32	0.002	0.269
Citraconic acid	11.2	147	931.6	2	0.003	1.59
serine	11.3	204	911.4	1.13	0.001	0.664
3-hydroxy-L-proline	12.4	82	294.3	1.92	2.13E-06	0.522
Aspartic acid	12.21	160	909.9	2.34	0.000315	1.792
3-hydroxy-L-proline	12.37	82	294.3	1.45	0.018	1.392
L-Malic acid	13	66	811.3	2.48	0.000144	1.43E+08
Salicylic acid	13.29	159	250	1.47	0.002	0.481
Oxoproline	13.4	156	850.9	4.81	0.003	0.23
4-aminobutyric acid	13.5	304	823.8	2.1	7.13E-10	0.732
Threonic acid	13.81	292	925.1	2.12	1.17E-15	0.238
Digitoxose	14.4	204	552.5	1.64	0.007	1.532
4-Hydroxyphenyl ethanol	14.04	179	596.4	2.34	2.38E-07	0.001
Lyxose	14.91	307	915.7	1.73	0.00012	3.741
Xylose	15.16	217	714.9	2.9	1.13E-07	0.267
Acetol	15.62	172	450.6	1.01	1.53E-16	0.035
D-Arabitol	15.87	205	649.4	1.3	3.51E-07	0.344
D-(glycerol 1-phosphate)	16.13	299	634.9	2.03	6.91E-11	0.086
Glucose-1-phosphate	16.18	232	864.4	1.12	1.38E-05	0.643
4-hydroxy-3-Methoxybenzoic acid	16.28	297	551.9	1.1	7.28E-14	0.154
2-Deoxy-D-galactose	16.54	437	602.1	2.22	0.033	0.077
Alpha-D-glucosamine 1-phosphate	16.94	334	409.6	1.88	1.92E-08	9.75E-09
Glucose	17.9	235	671.2	1.58	0.004	2.57E-08
D-Talose	17.83	305	333.7	1.44	0.003	0.23
Sedoheptulose	18	204	688.8	4.17	7.81E-09	1.338
D-galacturonic acid	18.04	333	632	3.28	0.034	0.081
Conduritol β epoxide	18.19	265	714	1.28	6.43E-07	0.822
mucic acid	18.93	333	850.8	1.33	1.28E-18	0.200
Palmitic acid	19.03	117	919	2.85	1.92E-06	0.172
Ferulic acid	19.5	338	846.7	1.27	3.43E-07	2.595
Oleic acid	20.56	117	762.8	1.09	5.79E-07	0.023
Linolenic acid	20.58	79	563.2	1.04	2.94E-06	0.019
Stearic acid	20.8	117	919.3	1.09	0.001	0.403
Purine riboside	22.09	105	455.5	3.6	7.77E-06	0.241
D-erythro-sphingosine	22.44	204	635.6	1.05	0.002	0.314
Cytidine-monophosphate	22.62	105	404.8	3.95	1.81E-06	0.322
Inosine	23.4	233	591.6	1.23	0.027	8.256
Lactose	24.56	204	740.9	3.37	3.36E-10	0.558
Trehalose	24.64	191	935.7	1.79	4.57E-07	3.446
Melibiose	25.52	361	873.2	1.1	8.83E-06	0.193
Galactinol	26	204	802.2	2.15	1.05E-10	11.838
Naringin	28.78	361	646.5	3.53	1.62E-10	0.317
1-Kestose	28.89	169	757	1.09	0.044	2.611

^a^RT, Retention time.

^b^Similarity, the mass spectral similarity in comparison with authentic standards.

^c^VIP, Variable importance in the projection.

^d^Fold change, the mean value of peak area obtained from non-infected *C. sinensis*/the mean value of peak area obtained from infected *C. sinensis* at 3 days post-inoculation.

**Figure 8.**
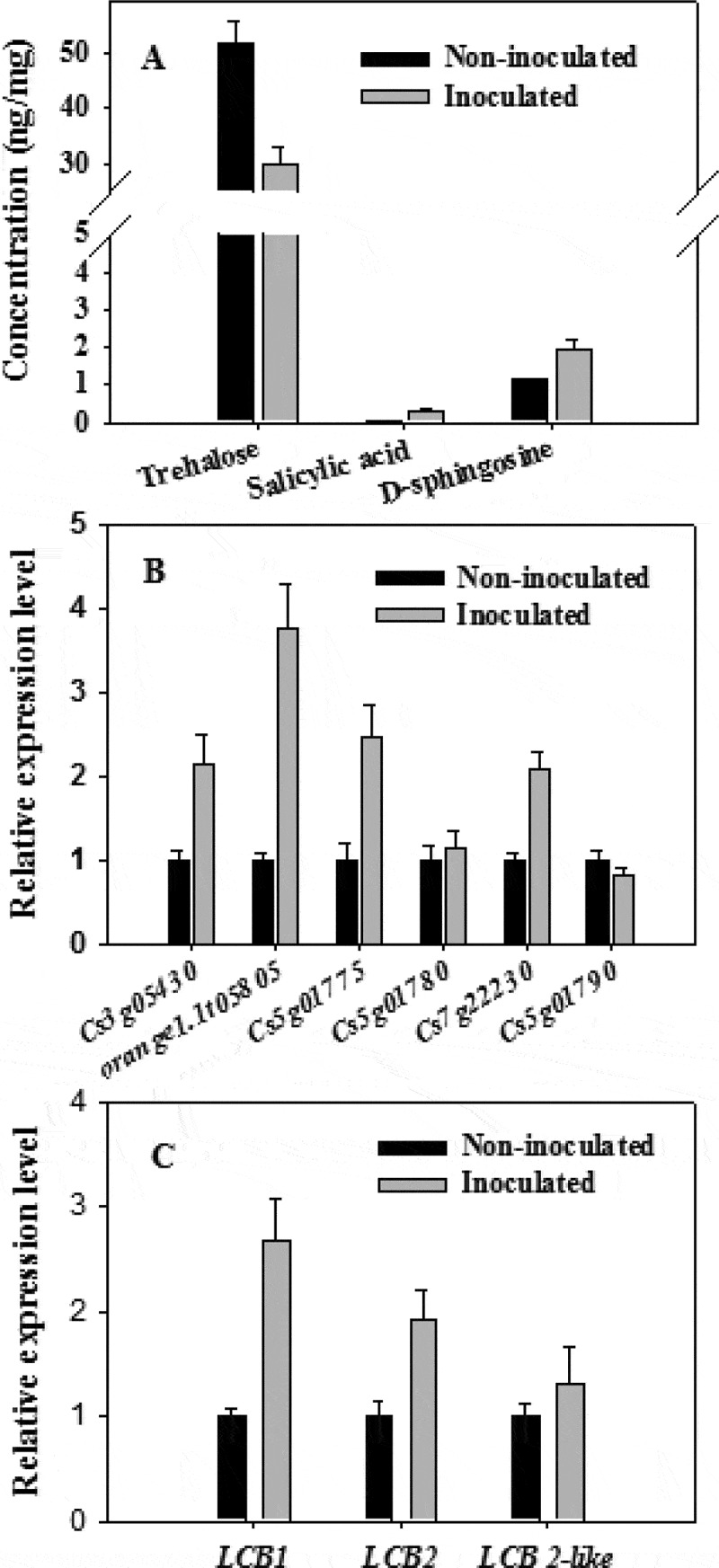
(a) Comparison of the concentrations of specific chemicals (trehalose, salicylic acid, and D-sphingosine) between the *P. italicum*-infected and healthy cuticles of Valencia orange based on a UPLC-QQQ-MS analysis. Expression of six genes encoding trehalase (b) and three genes encoding serine palmitoyl transferase (c) in non-inoculated and inoculated Valencia orange fruits at 3 dpi.

Trehalose is a widely distributed non-reducing disaccharide that has important effects on plant growth and development as well as responses to abiotic stresses [44]. There is emerging evidence that trehalose is also involved in plant responses to pathogens. For example, exogenous trehalose acts as an elicitor to induce the resistance of wheat to powdery mildew disease [45]. Zhang et al. [46] applied virus-induced gene silencing to reveal the involvement of several putative trehalose-6-phosphate synthase/phosphatase genes in the resistance of tomato plants to *Botrytis cinerea* and *Pseudomonas syringae*. In the present study, the trehalose concentration of non-infected Valencia orange was 3.45 times higher than that in infected fruits at 3 dpi (Table 4). The decreased accumulation of trehalose in infected Valencia orange was also confirmed by ultra-high performance liquid chromatography and triple quadrupole mass spectrometry (UPLC-QQQ-MS) (Figure 8a). The Valencia orange genome carries six genes (*Cs3g05430, orange1.1t05805, Cs5g01775, Cs5g01780, Cs7g22230*, and *Cs5g01790*) encoding trehalase, which is the enzyme responsible for the degradation of trehalose [47]. Of these genes, *Cs3g05430, orange1.1t05805, Cs5g01775* and *Cs7g22230* were more highly expressed in the infected Valencia orange than in the non-infected fruit at 3 dpi, whereas there were no significant differences in the expression of *Cs5g01780* and *Cs5g01790* (Figure 8b). In addition, we identified six trehalose-phosphate synthase genes (GL_Gan1_GLEAN_10003645, GL_Gan1_GLEAN_ 10,003,647, GL_Gan1_GLEAN_10007719, GL_Gan1_GLEAN_10006325, GL_Gan1_GLEAN _10009252, GL_Gan1_GLEAN_10007138) and one trehalase gene (GL_Gan1_GLEAN_10009387) in *P. italicum* GL_Gan1. Among these genes, expression of GL_Gan1_GLEAN_10003645 were significantly down-regulated at 3 dpi compared with at 0 dpi, while expression of other genes showed no significant difference between at 0 dpi and at 3 dpi. The up-regulated expression of trehalase genes in Valencia orange and the down-regulated expression of trehalose-phosphate synthase in *P. italicum* was consistent with the decreased accumulation of trehalose in the infected Valencia orange fruit. Therefore, during the infection of Valencia orange, *P. italicum* induces the expression of trehalose degradation-related genes, which accelerates trehalose degradation and decreases disease resistance.

Salicylic acid is a key plant hormone that mediates host responses to microbial pathogens. A UPLC-QQQ-MS analysis indicated the salicylic acid concentration was 0.27 ng/mg in inoculated fruits and 0.055 ng/mg in non-inoculated fruits at 3 dpi, implying that a *P. italicum* infection induces salicylic acid biosynthesis in Valencia orange. Sphingosine is an important regulator of SA accumulation in plant cells [48]. Additionally, D-sphingosine levels in non-inoculated Valencia orange was significantly lower than that in inoculated Valencia orange (Table 4), which was consistent with the UPLC-QQQ-MS data (Figure 8a). Serine palmitoyl transferase, a heterodimer composed of LCB1 and LCB2 subunits, catalyzes the first reaction of the biosynthesis of LCB (sphingoid long-chain base) [48]. The *LCB1, LCB2*, and *LCB2-like* genes were expressed more highly in inoculated Valencia orange than in non-inoculated Valencia orange (Figure 8c). This explains the high D-sphingosine content in the inoculated Valencia orange. These results suggest that the infection of Valencia orange by *P. italicum* involves the signaling pathways associated with sphingolipid metabolism and SA accumulation.

The abundance of several disease resistance-related metabolites, including galactinol, ferulic acid, and benzoic acid, significantly decreased in the inoculated Valencia orange at 3 dpi, which likely contributed to the susceptibility of the fruits to a *P. italicum* infection [49,50].

**Figure 9.**
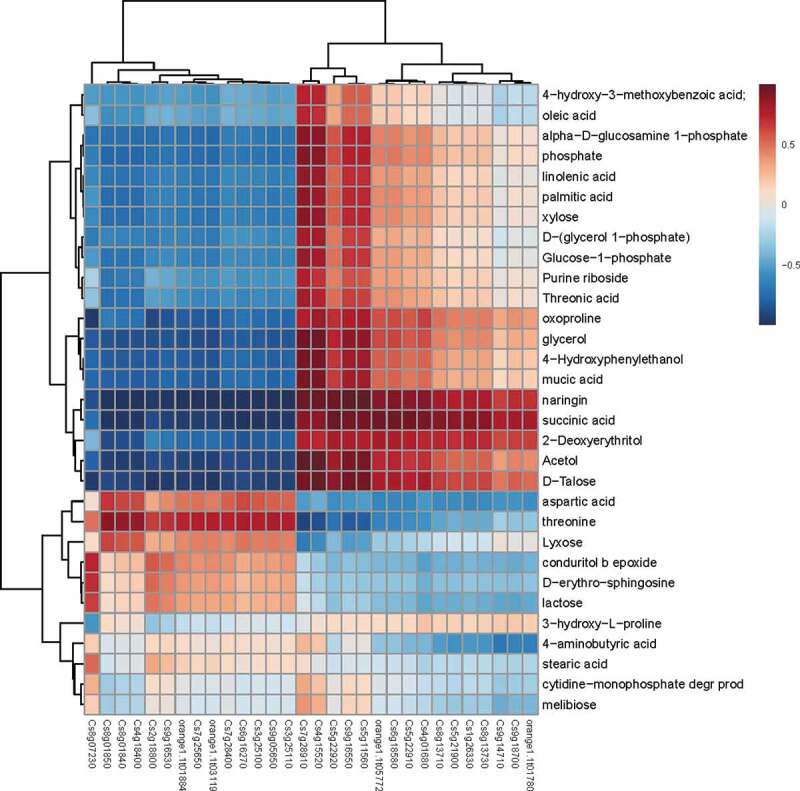
Heat map analysis of metabolite contents and gene expression profiles. Correlation of metabolite content (vertical axis) and gene expression (horizontal axis) between *P. italicum*-infected and healthy cuticles of Valencia orange at 3 dpi. Red and blue represent positive and negative correlations, respectively.

An analysis of the correlation between metabolite accumulation and gene expression clarified the role of primary or secondary metabolism in the disease resistance of Valencia orange or the pathogenicity of *P. italicum*. The expression levels of several carbohydrate metabolism-related genes, including *Cs7g28910* (UDP-apiose/xylose synthase), *Cs4g15520* (hexokinase), *Cs5g11560* (UDP-glucose 6-dehydrogenase), *Cs5g22920* (fructokinase), and *Cs9g16550* (fructokinase), were positively correlated with the accumulation of metabolites (Figure 9), indicating that carbohydrate metabolism was related to the infection by *P. italicum*.

### Conclusion

We sequenced the genome of *P. italicum* GL_Gan1, which is a local strain in Guangzhou, China. A comparative genomics analysis among three *Penicillium* species, including *P. italicum, P. digitatum*, and *P. expansum*, suggested that the host range expansion from *P. italicum* and *P. digitatum* to *P. expansum* might be related to the expansion of protein families, genome restructuring, HGT, and positive selection pressure. In addition, we explored the molecular basis underlying the pathogenicity of *P. italicum* to Valencia Orange (*Citrus sinensis*). Diverse strategies mediating the pathogenicity of this fungus were revealed, including (1) the activation of effectors, such as CAZymes, SSCPs; (2) the suppression of host *R* genes; (3) the regulation of defense responses-related metabolites in host, such as trehalose and salicylic acid. However, in the present study, we have not compared diseased tissue to non-diseased tissue or fungal transcripts in pathogenic vs nonpathogenic interactions from a transcriptome perspective. More transcriptome analysis are necessary to elucidate the pathogen-host interaction. The data presented herein may be useful for further elucidating the molecular basis underlying the evolution of the host specificity of *Penicillium* species and for illustrating the the pathogenicity of *P. italicum* to Valencia Orange.

## Materials and Methods

### Fungal strain

*Penicillium italicum* GL_**Gan1** was first isolated in Guangzhou, China from infected sweet orange exhibiting typical blue mold symptoms. The strain was purified as a monospore and stored at −80°C prior to use.

### Fruit inoculation

The *P. italicum* GL_Gan1 conidiospores were suspended in sterile water containing 0.05% (v/v) Tween 80. The spore concentration in the suspension was adjusted to 1 × 10^7^ spores/ml with a hemocytometer under an optical microscope (Olympus, Japan). Valencia orange (*C. sinensis*) was purchased from a local market and sterilized with 75% ethanol. Five cross-like wounds (width: 0.5–1; depth: 1–2 cm) were made at the base of the Valencia orange with sterilized tips. The wounds were then inoculated with 10 µl spore suspension, after which the fruits were incubated in a controlled-environment room (26 ± 1°C, natural photoperiod, and 70%–80% humidity). Infected Valencia orange peel tissues (flavedo and albedo) were collected at 0, 1, 3, 5, and 10 dpi, and then immediately frozen in liquid nitrogen for the subsequent RNA-seq and quantitative real-time PCR (qPCR) analyses. After inoculation, the tissues was immediately collected as the sample at 0 dpi.

### Extraction of DNA and RNA

*Penicillium italicum* GL_Gan1 was cultured on potato dextrose agar for 7 days, after which spores were harvested. Genomic DNA was extracted from conidiospores (80 mg) with the HiPure Fungal DNA kit (Magen, Guangzhou, China). High-quality DNA was submitted to BGI-Shenzhen (Shenzhen, China) for genome sequencing. Total RNA was extracted from the inoculated Valencia orange peel tissue with TRIzol® reagent according to the manufacturer’s instructions (Invitrogen, China).

### Genome sequencing and assembly

The *P. italicum* GL_Gan1 genomic DNA was used to construct 500-bp sequencing libraries. Sequencing data comprising 1,815 Mb (125-bp paired-end) were generated with the Illumina HiSeq™ 2000 system at BGI-Shenzhen (Shenzhen, China). To ensure the accuracy of the assembly, reads with 25 low-quality (≤ Q2) bases, 10% Ns, or a 15-bp overlap between the adapter, and duplicated reads were eliminated. The short reads were assembled with SOAPdenovo 1.05, with the assembly optimized with the key parameter K = 63.

### Gene prediction and annotation

Homology-based and *de novo* methods were used to predict the genes in the assembled genome. For the homology-based prediction, genes encoding *P. digitatum* Pd1 proteins were mapped onto the assembled genome with Genewise [51]. Regarding the *de novo* prediction, the Augustus program [52] was used, with appropriate parameters. Data from these complementary analyses were merged to produce a non-redundant reference gene set using GLEAN (http://glean-gene.sourceforge.net/). Repeat sequences were identified with Repeat Masker (version 3.3.0) and Repbase (version 18.05), with the following parameters: – nolow – no_is – norna-s – engine wublast – parallel 1. Repeat Protein Mask was also used with the following parameters: – noLowSimple – pvalue = 1e-4 (http://www.repeatmasker.org/). Tandem repeats in DNA sequences were detected with Tandem Repeat Finder (version 4.0.4), with the following parameters: 2 7 7 80 10 50 Period_size – d – h seqfile (http://tandem.bu.edu/trf/trf.html). The protein-encoding genes were annotated via BLASTP searches of the SwissProt (release: 2012_06), GO (release: 1.419), COG (release: 20,090,331), KEGG (release: 59), and NR (20,150,222) databases, with a threshold e-value ≤ 1e-5.

The CAZyme database (http://www.cazy.org/) was used to identify proteins involved in carbohydrate metabolism. Pathogenicity- and virulence-related genes were identified using the PHI-base database (http://www.phibase.org/). We identified PKS and NRPS with SMURF [53]. Cytochrome P450 s were identified with the Cytochrome P450 database (http://drnelson.uthsc.edu/CytochromeP450.html). The SSCPs of *P. italicum* GL_Gan1 were analyzed as described by Que et al. [28]. The GPCR sequences were evaluated regarding their seven transmembrane regions with Phobius [54] and the default settings of TMHMM 2.0. The *R* genes were identified as previously described [44]. The STEM program (version 1.3.9) [55] was used to compare and visualize the RNA-seq data.

### Comparative genomics analysis

Protein sequences for *P. italicum* GL_Gan1, *P. expansum* CMP1, *P. expansum* Pd1, *P. expansum* MD8, *P. italicum* PHI1, *P. digitatum* PHI26, and *A. nidulans* FGSCA4 were aligned and redundant sequences were eliminated with BLAST and SOLAR (https://surfsara.nl/systems/lisa/software/solar). Genes were clustered with TreeFam and Hcluster_sg [56], with -w 10 -s 0.34 -m 500 -b 0.1 -C category genes_file. After clustering, multiple protein sequences were aligned for each gene family with MUSCLE [57], and the protein alignments were converted to CDS alignments.

The single-copy core genes of the above-mentioned six *Penicillium* species/strains and *A. nidulans* (outgroup species) were identified with Hcluster_sg [56]. Multiple sequences were then aligned with MUSCLE [57]. The phyml subprogram of TreeBeST (http://treesoft.sourceforge.net/treebest.shtml) was used to construct a phylogenetic tree, with default parameters and 1,000 bootstrap replicates. The core genome and pan-genome of the six *Penicillium* species/strains were obtained based on a previous analysis of a *Yersinia pestis* population [58]. Additionally, Ka and Ks were estimated using an established method [59]. Horizontal gene transfer was analyzed according to a published procedure [60]. Protein sequences that were shorter than 150 amino acids were excluded. The BLASTP algorithm was used for similarity searches involving the bacterial and viral protein databases of NR (1e-5, identity > 40%). Moreover, phylogenetic trees with fewer than 10 species were excluded. Multiple sequences were then aligned with MUSCLE [57] MEGA v6.06 (https://www.megasoftware.net/) was used to construct Neighbor-joining tree with default parameters and 2,000 bootstrap replicates. With which the iTOL v4.0 (https://itol.embl.de) was employed to improve the display, annotation and management of phylogenetic trees.

### RNA sequencing

Two independent biological replicates of inoculated Valencia orange fruits were used to construct 10 short-fragment libraries with the Illumina TruSeq RNA library construction kit (version 2) (Illumina, CA, USA) for an RNA-seq analysis. After low-quality raw reads were discarded, the remaining high-quality reads were aligned to the *P. italicum* GL_Gan1 and Valencia orange gene sequences with SOAP2 [61]. The RNA-seq data were analyzed and gene expression levels (as FPKM) were calculated with the RSEM software package [62]. The NOIseq method was applied to screen for differentially expressed genes between two groups [63]. An FDR ≤ 0.001 and an absolute value of the log_2_ ratio ≥ 1 were used as the criteria for identifying differentially expressed genes.

### qPCR analysis

The PrimeScript™ RT reagent Kit with gDNA Eraser (Takara, Otsu, Japan) was used to synthesize cDNA. Gene-specific primer pairs (Table S5) were designed with the Primer Express software (Applied Biosystems, Foster City, CA, USA). *G3PDH* and *Actin* were used as reference gene. The qPCR analysis was completed with three biological replicates. The output data were generated with the Sequence Detector program (version 1.3.1) (Applied Biosystems) and then analyzed according to the 2^−ΔΔCt^ method [64].

### Metabolomics analysis

Valencia orange peel tissues infected with *P. italicum* were collected at 3 dpi for a GC-TOF-MS analysis as described by Dunn et al. [65]. Metabolites were extracted from 1.0 g citrus samples with a methanol:chloroform (3:1) solution, with 100 μl adonitol (0.2 mg/ml) as an internal standard. After derivatization, the extracts were added with fatty acid methyl esters (C8-C24) as retention index markers derivatized and then were transferred to a 7890 gas chromatograph system (Agilent Technologies, USA) connected to a Pegasus HT time-of-flight mass spectrometer (LECO Corporation, USA). Additionally, a DB-5 MS capillary column was coated with cross-linked 5% diphenyl/95% dimethyl polysiloxane (30 m × 250 μm inner diameter, 0.25 μm film thickness; J&W Scientific, Folsom, CA, USA). The Chroma TOF 4.3X software (LECO, St. Joseph, USA) and LECO-Fiehn Rtx5 database were used for the raw peak extraction, baselines correction, deconvolution analysis and peak identification. Both of retention time index (RT) and mass spectral similarity were considered in metabolites identification. If the similarity is >700, we deem the metabolite identification is reliable. If the similarity is < 200, the compound name is defined as “analyte”, and the similarity between 200 and 700, the compound name was considered as a putative annotation.

Principal component analysis (PCA) and orthogonal projections to latent structures-discriminant analysis (OPLS-DA) were used to display the similarity and difference of the origin data with the SIMCA-P 13.0 software package (Umetrics, Umea, Sweden).

To refine this analysis, the first principal component of variable importance projection (VIP) was obtained. The VIP values exceeding 1.0 were first selected as changed metabolites. In step 2, the remaining variables were then assessed by Student’s T test (T-test), P > 0.05, variables were discarded between two comparison groups.

Of the differentially accumulated metabolites, we quantified trehalose, salicylic acid, and D-sphingosine by UPLC-QQQ-MS. The standard chemicals were purchased from Sigma Chemical Co. (St. Louis, USA) and prepared as 100 µg/ml solutions. A 500-mg citrus peel sample was prepared and analyzed with the 6460 triple quadrupole LC/MS system with an electrospray ionization source (Torrance, CA, USA) as described by Chen et al. [66]. Samples were examined with six repetitions. Additionally, the Pearson correlation coefficient was used to assess the relationship between the metabolite changes and gene expression levels. Furthermore, a heat map was prepared with the R program and the PathPod mapping system as described by Hsu et al. [67].

### Statistical analysis

Data are presented herein as the mean value of three biological replicates ± standard deviation. The significance of any differences among samples was calculated with SPSS (version 7.5) (SPSS, Inc., Chicago, IL, USA).

## Supplementary Material

Supplemental Material

## Data Availability

The draft *P. italicum* GL_Gan1 genome sequence was deposited in the GenBank database (accession number LWEC00000000). The transcriptome sequence was deposited in the GenBank database (accession number SRP073474).
