# Supplementary material for: New insights into the evolution of host specificity of three Penicillium species and the pathogenicity of P. Italicum involving the infection of Valencia orange (Citrus sinensis)
Source: Virulence. 2020 Jun 11;11(1):748–68. doi: 10.1080/21505594.2020.1773038 (PMC7549954; doi:10.1080/21505594.2020.1773038)
Supplement: Supplemental Material [file KVIR_A_1773038_SM2584.zip › Dataset S1.docx]

**Dataset S1** Species-specific genes (A), horizontal gene transfer (B), effectors (C), genes under positive selection pressure (PSP) (D) in the *P. italicum* GL-Gan1 genome; Functional annotation with GO (E), KEGG (F) and COG (G) for HGT in the *P. italicum* GL-Gan1 genome

(**A**) Species-specific genes

| Species specific unigene_ID | Swissprot | Interpro | nr |
| --- | --- | --- | --- |
| GL_Gan1_GLEAN_10000001 | INSH5_ECOLI Transposase insH for insertion sequence element IS5Y OS=Escherichia coli (strain K12) GN=insH5 PE=3 SV=1 | IPR002559; Transposase, IS4-like IPR008490; Transposase InsH, N-terminal | transposase [Pseudomonas stutzeri] |
| GL_Gan1_GLEAN_10000024 | NA | NA | NA |
| GL_Gan1_GLEAN_10000036 | NA | NA | NA |
| GL_Gan1_GLEAN_10000072 | NA | NA | NA |
| GL_Gan1_GLEAN_10000074 | NA | NA | NA |
| GL_Gan1_GLEAN_10000076 | NA | NA | NA |
| GL_Gan1_GLEAN_10000077 | NA | NA | NA |
| GL_Gan1_GLEAN_10000092 | NA | NA | unnamed protein product [Penicillium roqueforti FM164] |
| GL_Gan1_GLEAN_10000105 | NA | NA | NA |
| GL_Gan1_GLEAN_10000131 | NA | NA | conserved hypothetical protein [Aspergillus fumigatus A1163] |
| GL_Gan1_GLEAN_10000143 | NA | NA | conserved hypothetical protein [Histoplasma capsulatum NAm1] |
| GL_Gan1_GLEAN_10000240 | NA | NA | NA |
| GL_Gan1_GLEAN_10000241 | NA | NA | NA |
| GL_Gan1_GLEAN_10000250 | NA | NA | NA |
| GL_Gan1_GLEAN_10000266 | NA | NA | NA |
| GL_Gan1_GLEAN_10000436 | NA | NA | NA |
| GL_Gan1_GLEAN_10000548 | NA | NA | NA |
| GL_Gan1_GLEAN_10000671 | NA | NA | NA |
| GL_Gan1_GLEAN_10000721 | NA | NA | NA |
| GL_Gan1_GLEAN_10000894 | NA | NA | unnamed protein product [Penicillium roqueforti FM164] |
| GL_Gan1_GLEAN_10001144 | NA | NA | NA |
| GL_Gan1_GLEAN_10001408 | NA | NA | NA |
| GL_Gan1_GLEAN_10001463 | NA | IPR003014; PAN-1 domain | unnamed protein product [Penicillium roqueforti FM164] |
| GL_Gan1_GLEAN_10001585 | NA | NA | hypothetical protein TEQG_08624 [Trichophyton equinum CBS 127.97] |
| GL_Gan1_GLEAN_10001834 | NA | NA | NA |
| GL_Gan1_GLEAN_10001878 | NA | NA | NA |
| GL_Gan1_GLEAN_10002055 | NA | NA | NA |
| GL_Gan1_GLEAN_10002214 | NA | NA | NA |
| GL_Gan1_GLEAN_10002215 | NA | NA | hypothetical protein NFIA_092510 [Neosartorya fischeri NRRL 181] |
| GL_Gan1_GLEAN_10002220 | NA | NA | hypothetical protein NFIA_030960 [Neosartorya fischeri NRRL 181] |
| GL_Gan1_GLEAN_10002333 | NA | NA | NA |
| GL_Gan1_GLEAN_10002515 | NA | NA | NA |
| GL_Gan1_GLEAN_10002799 | NA | NA | NA |
| GL_Gan1_GLEAN_10002969 | NA | NA | NA |
| GL_Gan1_GLEAN_10003054 | NA | NA | NA |
| GL_Gan1_GLEAN_10003078 | NA | NA | unnamed protein product [Penicillium roqueforti FM164] |
| GL_Gan1_GLEAN_10003179 | NA | NA | Glycoside hydrolase, family 71 [Penicillium roqueforti FM164] |
| GL_Gan1_GLEAN_10003337 | NA | NA | NA |
| GL_Gan1_GLEAN_10003368 | NA | NA | NA |
| GL_Gan1_GLEAN_10003404 | NA | NA | NA |
| GL_Gan1_GLEAN_10003419 | NA | NA | NA |
| GL_Gan1_GLEAN_10003740 | NA | NA | NA |
| GL_Gan1_GLEAN_10003789 | NA | NA | hypothetical protein GMDG_06752 [Pseudogymnoascus destructans 20631-21] |
| GL_Gan1_GLEAN_10004046 | NA | NA | NA |
| GL_Gan1_GLEAN_10004076 | NA | NA | NA |
| GL_Gan1_GLEAN_10004088 | NA | NA | hypothetical protein TEQG_08624 [Trichophyton equinum CBS 127.97] |
| GL_Gan1_GLEAN_10004106 | NA | NA | NA |
| GL_Gan1_GLEAN_10004135 | NA | NA | NA |
| GL_Gan1_GLEAN_10004445 | NA | NA | NA |
| GL_Gan1_GLEAN_10004857 | NA | NA | NA |
| GL_Gan1_GLEAN_10004947 | NA | NA | NA |
| GL_Gan1_GLEAN_10004973 | NA | NA | hypothetical protein PDIP_11230 [Penicillium digitatum Pd1] |
| GL_Gan1_GLEAN_10005298 | NA | NA | unnamed protein product [Penicillium roqueforti FM164] |
| GL_Gan1_GLEAN_10005544 | NA | NA | hypothetical protein TEQG_08624 [Trichophyton equinum CBS 127.97] |
| GL_Gan1_GLEAN_10005600 | NA | NA | NA |
| GL_Gan1_GLEAN_10005766 | NA | NA | NA |
| GL_Gan1_GLEAN_10005999 | NA | NA | NA |
| GL_Gan1_GLEAN_10006048 | NA | NA | NA |
| GL_Gan1_GLEAN_10006142 | CMTD1_MOUSE Catechol O-methyltransferase domain-containing protein 1 OS=Mus musculus GN=Comtd1 PE=2 SV=1 | IPR002935; O-methyltransferase, family 3 | hypothetical protein GLRG_11986 [Colletotrichum graminicola M1.001] |
| GL_Gan1_GLEAN_10006385 | NA | NA | NA |
| GL_Gan1_GLEAN_10006466 | NA | NA | NA |
| GL_Gan1_GLEAN_10006524 | NA | NA | hypothetical protein BA78_8864 [Aspergillus fumigatus var. RP-2014] |
| GL_Gan1_GLEAN_10006821 | NA | NA | NA |
| GL_Gan1_GLEAN_10006858 | NA | NA | NA |
| GL_Gan1_GLEAN_10007109 | NA | NA | unnamed protein product [Penicillium roqueforti FM164] |
| GL_Gan1_GLEAN_10007150 | NA | NA | NA |
| GL_Gan1_GLEAN_10007217 | NA | NA | NA |
| GL_Gan1_GLEAN_10007287 | NA | NA | NA |
| GL_Gan1_GLEAN_10008031 | NA | NA | NA |
| GL_Gan1_GLEAN_10008158 | NA | NA | NA |
| GL_Gan1_GLEAN_10008182 | NA | NA | NA |
| GL_Gan1_GLEAN_10008302 | NA | NA | NA |
| GL_Gan1_GLEAN_10008354 | NA | NA | NA |
| GL_Gan1_GLEAN_10008488 | NA | NA | NA |
| GL_Gan1_GLEAN_10008500 | NA | NA | NA |
| GL_Gan1_GLEAN_10008517 | NA | NA | unnamed protein product [Penicillium roqueforti FM164] |
| GL_Gan1_GLEAN_10008579 | NA | NA | unnamed protein product [Penicillium roqueforti FM164] |
| GL_Gan1_GLEAN_10008635 | NA | NA | NA |
| GL_Gan1_GLEAN_10008735 | NA | NA | NA |
| GL_Gan1_GLEAN_10008755 | EGH_DROME Beta-1,4-mannosyltransferase egh OS=Drosophila melanogaster GN=egh PE=2 SV=1 | NA | hypothetical protein AKAW_03962 [Aspergillus kawachii IFO 4308] |
| GL_Gan1_GLEAN_10008764 | NA | NA | hypothetical protein TEQG_08624 [Trichophyton equinum CBS 127.97] |
| GL_Gan1_GLEAN_10008927 | NA | NA | NA |
| GL_Gan1_GLEAN_10009122 | NA | NA | NA |
| GL_Gan1_GLEAN_10009126 | NA | NA | NA |
| GL_Gan1_GLEAN_10009131 | NA | NA | NA |

(B) Horizontal gene transfer

| gene_id | cog | nr |
| --- | --- | --- |
| GL_Gan1_GLEAN_10003397 | COG0636 F0F1-type ATP synthase, subunit c/Archaeal/vacuolar-type H+-ATPase, subunit K C Energy production and conversion ; | Putative ATP synthase protein 9 [Penicillium digitatum Pd1] |
| GL_Gan1_GLEAN_10000587 | COG1028 Dehydrogenases with different specificities (related to short-chain alcohol dehydrogenases) IQR Lipid transport and metabolism ; Secondary metabolites biosynthesis, transport and catabolism ; General function prediction only ; | Short-chain dehydrogenase/reductase SDR [Penicillium italicum] |
| GL_Gan1_GLEAN_10000590 | COG1028 Dehydrogenases with different specificities (related to short-chain alcohol dehydrogenases) IQR Lipid transport and metabolism ; Secondary metabolites biosynthesis, transport and catabolism ; General function prediction only ; | Short-chain dehydrogenase/reductase SDR [Penicillium italicum] |
| GL_Gan1_GLEAN_10000712 | COG1473 Metal-dependent amidase/aminoacylase/carboxypeptidase R General function prediction only ; | Amidohydrolase [Penicillium italicum] |
| GL_Gan1_GLEAN_10000719 | COG0111 Phosphoglycerate dehydrogenase and related dehydrogenases HE Coenzyme transport and metabolism ; Amino acid transport and metabolism ; | D-isomer specific 2-hydroxyacid dehydrogenase, NAD-binding [Penicillium italicum] |
| GL_Gan1_GLEAN_10001354 | NA | ER membrane protein Wsc4, putative [Penicillium digitatum PHI26] |
| GL_Gan1_GLEAN_10001363 | COG0596 Predicted hydrolases or acyltransferases (alpha/beta hydrolase superfamily) R General function prediction only ; | hypothetical protein PITC_052060 [Penicillium italicum] |
| GL_Gan1_GLEAN_10008272 | COG0788 Formyltetrahydrofolate hydrolase F Nucleotide transport and metabolism ; | Formyl transferase, N-terminal [Penicillium italicum] |
| GL_Gan1_GLEAN_10008313 | NA | Galactose oxidase/kelch, beta-propeller [Penicillium italicum] |
| GL_Gan1_GLEAN_10008328 | COG1335 Amidases related to nicotinamidase Q Secondary metabolites biosynthesis, transport and catabolism ; | hypothetical protein PITC_097020 [Penicillium italicum] |
| GL_Gan1_GLEAN_10008336 | COG0366 Glycosidases G Carbohydrate transport and metabolism ; | Glycoside hydrolase, superfamily [Penicillium italicum] |
| GL_Gan1_GLEAN_10008341 | COG2021 Homoserine acetyltransferase E Amino acid transport and metabolism ; | Homoserine acetyltransferase [Penicillium italicum] |
| GL_Gan1_GLEAN_10008349 | NA | hypothetical protein PITC_097230 [Penicillium italicum] |
| GL_Gan1_GLEAN_10008352 | COG1794 Aspartate racemase M Cell wall/membrane/envelope biogenesis ; | Aspartate racemase [Penicillium italicum] |
| GL_Gan1_GLEAN_10008391 | COG3511 Phospholipase C M Cell wall/membrane/envelope biogenesis ; | Phosphoesterase [Penicillium italicum] |
| GL_Gan1_GLEAN_10008444 | COG2055 Malate/L-lactate dehydrogenases C Energy production and conversion ; | Malate/L-lactate dehydrogenase [Penicillium italicum] |
| GL_Gan1_GLEAN_10008450 | COG0006 Xaa-Pro aminopeptidase E Amino acid transport and metabolism ; | hypothetical protein PITC_047180 [Penicillium italicum] |
| GL_Gan1_GLEAN_10008464 | COG0154 Asp-tRNAAsn/Glu-tRNAGln amidotransferase A subunit and related amidases J Translation, ribosomal structure and biogenesis ; | Amidase [Penicillium italicum] |
| GL_Gan1_GLEAN_10008491 | COG0673 Predicted dehydrogenases and related proteins R General function prediction only ; | Oxidoreductase, N-terminal [Penicillium italicum] |
| GL_Gan1_GLEAN_10008511 | COG0652 Peptidyl-prolyl cis-trans isomerase (rotamase) - cyclophilin family O Posttranslational modification, protein turnover, chaperones ; | Cyclophilin-type peptidyl-prolyl cis-trans isomerase [Penicillium italicum] |
| GL_Gan1_GLEAN_10008512 | COG4106 Trans-aconitate methyltransferase R General function prediction only ; | Trans-aconitate 2-methyltransferase, C-terminal [Penicillium italicum] |
| GL_Gan1_GLEAN_10004441 | COG0625 Glutathione S-transferase O Posttranslational modification, protein turnover, chaperones ; | Glutathione S-transferase/chloride channel, C-terminal [Penicillium italicum] |
| GL_Gan1_GLEAN_10004477 | COG2140 Thermophilic glucose-6-phosphate isomerase and related metalloenzymes GR Carbohydrate transport and metabolism ; General function prediction only ; | Bicupin, oxalate decarboxylase/oxidase [Penicillium italicum] |
| GL_Gan1_GLEAN_10004524 | NA | Concanavalin A-like lectin/glucanases superfamily [Penicillium italicum] |
| GL_Gan1_GLEAN_10004535 | COG1028 Dehydrogenases with different specificities (related to short-chain alcohol dehydrogenases) IQR Lipid transport and metabolism ; Secondary metabolites biosynthesis, transport and catabolism ; General function prediction only ; | Short-chain dehydrogenase/reductase SDR [Penicillium expansum] |
| GL_Gan1_GLEAN_10004540 | COG2140 Thermophilic glucose-6-phosphate isomerase and related metalloenzymes GR Carbohydrate transport and metabolism ; General function prediction only ; | Bicupin, oxalate decarboxylase/oxidase [Penicillium italicum] |
| GL_Gan1_GLEAN_10004544 | COG1472 Beta-glucosidase-related glycosidases G Carbohydrate transport and metabolism ; | Glycoside hydrolase, superfamily [Penicillium italicum] |
| GL_Gan1_GLEAN_10004985 | COG1028 Dehydrogenases with different specificities (related to short-chain alcohol dehydrogenases) IQR Lipid transport and metabolism ; Secondary metabolites biosynthesis, transport and catabolism ; General function prediction only ; | Glucose/ribitol dehydrogenase [Penicillium italicum] |
| GL_Gan1_GLEAN_10005006 | COG0131 Imidazoleglycerol-phosphate dehydratase E Amino acid transport and metabolism ; | Imidazoleglycerol-phosphate dehydratase [Penicillium italicum] |
| GL_Gan1_GLEAN_10001522 | COG0654 2-polyprenyl-6-methoxyphenol hydroxylase and related FAD-dependent oxidoreductases HC Coenzyme transport and metabolism ; Energy production and conversion ; | Monooxygenase, FAD-binding [Penicillium italicum] |
| GL_Gan1_GLEAN_10001523 | COG3485 Protocatechuate 3,4-dioxygenase beta subunit Q Secondary metabolites biosynthesis, transport and catabolism ; | Catechol dioxygenase, N-terminal [Penicillium italicum] |
| GL_Gan1_GLEAN_10001527 | COG4818 Predicted membrane protein S Function unknown ; | Tic20-like protein [Penicillium italicum] |
| GL_Gan1_GLEAN_10001543 | COG1250 3-hydroxyacyl-CoA dehydrogenase I Lipid transport and metabolism ; | Dehydrogenase, multihelical [Penicillium italicum] |
| GL_Gan1_GLEAN_10003767 | COG0161 Adenosylmethionine-8-amino-7-oxononanoate aminotransferase H Coenzyme transport and metabolism ; | Pyridoxal phosphate-dependent transferase, major region, subdomain 2 [Penicillium italicum] |
| GL_Gan1_GLEAN_10003768 | COG0451 Nucleoside-diphosphate-sugar epimerases MG Cell wall/membrane/envelope biogenesis ; Carbohydrate transport and metabolism ; | NAD-dependent epimerase/dehydratase [Penicillium italicum] |
| GL_Gan1_GLEAN_10003782 | COG4341 Predicted HD phosphohydrolase R General function prediction only ; | hypothetical protein PITC_045530 [Penicillium italicum] |
| GL_Gan1_GLEAN_10003816 | COG1051 ADP-ribose pyrophosphatase F Nucleotide transport and metabolism ; | NUDIX hydrolase [Penicillium italicum] |
| GL_Gan1_GLEAN_10003820 | COG0666 FOG: Ankyrin repeat R General function prediction only ; | hypothetical protein PEXP_093450 [Penicillium expansum] |
| GL_Gan1_GLEAN_10003824 | COG0435 Predicted glutathione S-transferase O Posttranslational modification, protein turnover, chaperones ; | Omega family [Penicillium italicum] |
| GL_Gan1_GLEAN_10002881 | COG2008 Threonine aldolase E Amino acid transport and metabolism ; | Aromatic amino acid beta-eliminating lyase/threonine aldolase [Penicillium italicum] |
| GL_Gan1_GLEAN_10002884 | COG0288 Carbonic anhydrase P Inorganic ion transport and metabolism ; | Carbonic anhydrase [Penicillium italicum] |
| GL_Gan1_GLEAN_10002913 | COG1171 Threonine dehydratase E Amino acid transport and metabolism ; | Peptidase M20 [Penicillium italicum] |
| GL_Gan1_GLEAN_10002372 | COG1062 Zn-dependent alcohol dehydrogenases, class III C Energy production and conversion ; | Alcohol dehydrogenase superfamily, zinc-type [Penicillium italicum] |
| GL_Gan1_GLEAN_10004267 | COG0179 2-keto-4-pentenoate hydratase/2-oxohepta-3-ene-1,7-dioic acid hydratase (catechol pathway) Q Secondary metabolites biosynthesis, transport and catabolism ; | Fumarylacetoacetase, C-terminal-like protein [Penicillium italicum] |
| GL_Gan1_GLEAN_10004270 | COG0638 20S proteasome, alpha and beta subunits O Posttranslational modification, protein turnover, chaperones ; | Proteasome B-type subunit [Penicillium italicum] |
| GL_Gan1_GLEAN_10004323 | COG1003 Glycine cleavage system protein P (pyridoxal-binding), C-terminal domain E Amino acid transport and metabolism ; | Pyridoxal phosphate-dependent transferase, major region, subdomain 1 [Penicillium italicum] |
| GL_Gan1_GLEAN_10007346 | COG1680 Beta-lactamase class C and other penicillin binding proteins V Defense mechanisms ; | Beta-lactamase-like protein [Penicillium italicum] |
| GL_Gan1_GLEAN_10007350 | COG0491 Zn-dependent hydrolases, including glyoxylases R General function prediction only ; | hypothetical protein PITC_034030 [Penicillium italicum] |
| GL_Gan1_GLEAN_10007353 | COG4833 Predicted glycosyl hydrolase G Carbohydrate transport and metabolism ; | Six-hairpin glycosidase [Penicillium italicum] |
| GL_Gan1_GLEAN_10007371 | COG2084 3-hydroxyisobutyrate dehydrogenase and related beta-hydroxyacid dehydrogenases I Lipid transport and metabolism ; | Dehydrogenase, multihelical [Penicillium italicum] |
| GL_Gan1_GLEAN_10007404 | COG1028 Dehydrogenases with different specificities (related to short-chain alcohol dehydrogenases) IQR Lipid transport and metabolism ; Secondary metabolites biosynthesis, transport and catabolism ; General function prediction only ; | Glucose/ribitol dehydrogenase [Penicillium italicum] |
| GL_Gan1_GLEAN_10007413 | COG1012 NAD-dependent aldehyde dehydrogenases C Energy production and conversion ; | Aldehyde dehydrogenase, C-terminal [Penicillium italicum] |
| GL_Gan1_GLEAN_10007420 | COG0451 Nucleoside-diphosphate-sugar epimerases MG Cell wall/membrane/envelope biogenesis ; Carbohydrate transport and metabolism ; | NAD-dependent epimerase/dehydratase [Penicillium italicum] |
| GL_Gan1_GLEAN_10007437 | COG0625 Glutathione S-transferase O Posttranslational modification, protein turnover, chaperones ; | Glutathione S-transferase/chloride channel, C-terminal [Penicillium expansum] |
| GL_Gan1_GLEAN_10007449 | COG3384 Uncharacterized conserved protein S Function unknown ; | Extradiol ring-cleavage dioxygenase, class III enzyme, subunit B [Penicillium italicum] |
| GL_Gan1_GLEAN_10007460 | COG1960 Acyl-CoA dehydrogenases I Lipid transport and metabolism ; | Acyl-CoA dehydrogenase, N-terminal [Penicillium italicum] |
| GL_Gan1_GLEAN_10000944 | COG1012 NAD-dependent aldehyde dehydrogenases C Energy production and conversion ; | Aldehyde dehydrogenase, N-terminal [Penicillium italicum] |
| GL_Gan1_GLEAN_10006658 | COG0431 Predicted flavoprotein R General function prediction only ; | hypothetical protein PEX2_106140 [Penicillium expansum] |
| GL_Gan1_GLEAN_10006668 | COG2072 Predicted flavoprotein involved in K+ transport P Inorganic ion transport and metabolism ; | hypothetical protein PITC_026600 [Penicillium italicum] |
| GL_Gan1_GLEAN_10006743 | NA | hypothetical protein PDIG_62220 [Penicillium digitatum PHI26] |
| GL_Gan1_GLEAN_10006767 | COG2421 Predicted acetamidase/formamidase C Energy production and conversion ; | Acetamidase/Formamidase [Penicillium italicum] |
| GL_Gan1_GLEAN_10006780 | COG1335 Amidases related to nicotinamidase Q Secondary metabolites biosynthesis, transport and catabolism ; | hypothetical protein PITC_092360 [Penicillium italicum] |
| GL_Gan1_GLEAN_10006784 | COG0652 Peptidyl-prolyl cis-trans isomerase (rotamase) - cyclophilin family O Posttranslational modification, protein turnover, chaperones ; | Cyclophilin-type peptidyl-prolyl cis-trans isomerase [Penicillium italicum] |
| GL_Gan1_GLEAN_10006791 | COG1028 Dehydrogenases with different specificities (related to short-chain alcohol dehydrogenases) IQR Lipid transport and metabolism ; Secondary metabolites biosynthesis, transport and catabolism ; General function prediction only ; | Short-chain dehydrogenase/reductase SDR [Penicillium expansum] |
| GL_Gan1_GLEAN_10006802 | COG3752 Predicted membrane protein S Function unknown ; | Protein of unknown function DUF1295 [Penicillium italicum] |
| GL_Gan1_GLEAN_10006811 | COG1454 Alcohol dehydrogenase, class IV C Energy production and conversion ; | Alcohol dehydrogenase, iron-type [Penicillium italicum] |
| GL_Gan1_GLEAN_10006812 | COG1454 Alcohol dehydrogenase, class IV C Energy production and conversion ; | Alcohol dehydrogenase, iron-type [Penicillium italicum] |
| GL_Gan1_GLEAN_10001750 | COG1075 Predicted acetyltransferases and hydrolases with the alpha/beta hydrolase fold R General function prediction only ; | hypothetical protein PITC_079810 [Penicillium italicum] |
| GL_Gan1_GLEAN_10001754 | COG0235 Ribulose-5-phosphate 4-epimerase and related epimerases and aldolases G Carbohydrate transport and metabolism ; | Pc16g11350 [Penicillium rubens Wisconsin 54-1255] |
| GL_Gan1_GLEAN_10001755 | NA | hypothetical protein PITC_079770 [Penicillium italicum] |
| GL_Gan1_GLEAN_10001758 | COG1335 Amidases related to nicotinamidase Q Secondary metabolites biosynthesis, transport and catabolism ; | hypothetical protein PITC_079740 [Penicillium italicum] |
| GL_Gan1_GLEAN_10001777 | COG0071 Molecular chaperone (small heat shock protein) O Posttranslational modification, protein turnover, chaperones ; | HSP20-like chaperone [Penicillium italicum] |
| GL_Gan1_GLEAN_10008044 | COG0666 FOG: Ankyrin repeat R General function prediction only ; | hypothetical protein PITC_025290 [Penicillium italicum] |
| GL_Gan1_GLEAN_10008052 | COG0666 FOG: Ankyrin repeat R General function prediction only ; | hypothetical protein PITC_025360 [Penicillium italicum] |
| GL_Gan1_GLEAN_10008068 | COG1472 Beta-glucosidase-related glycosidases G Carbohydrate transport and metabolism ; | Glycoside hydrolase, superfamily [Penicillium italicum] |
| GL_Gan1_GLEAN_10008146 | COG0160 4-aminobutyrate aminotransferase and related aminotransferases E Amino acid transport and metabolism ; | Pyridoxal phosphate-dependent transferase, major region, subdomain 2 [Penicillium italicum] |
| GL_Gan1_GLEAN_10008147 | NA | hypothetical protein PITC_032740 [Penicillium italicum] |
| GL_Gan1_GLEAN_10008151 | COG0021 Transketolase G Carbohydrate transport and metabolism ; | Transketolase, N-terminal [Penicillium italicum] |
| GL_Gan1_GLEAN_10008172 | COG2833 Uncharacterized protein conserved in bacteria S Function unknown ; | Protein of unknown function DUF455 [Penicillium italicum] |
| GL_Gan1_GLEAN_10008175 | COG1335 Amidases related to nicotinamidase Q Secondary metabolites biosynthesis, transport and catabolism ; | hypothetical protein PITC_033040 [Penicillium italicum] |
| GL_Gan1_GLEAN_10008180 | COG0106 Phosphoribosylformimino-5-aminoimidazole carboxamide ribonucleotide (ProFAR) isomerase E Amino acid transport and metabolism ; | Aldolase-type TIM barrel [Penicillium italicum] |
| GL_Gan1_GLEAN_10008261 | COG1027 Aspartate ammonia-lyase E Amino acid transport and metabolism ; | Fumarase C, C-terminal [Penicillium italicum] |
| GL_Gan1_GLEAN_10000547 | COG0667 Predicted oxidoreductases (related to aryl-alcohol dehydrogenases) C Energy production and conversion ; | Aldo/keto reductase [Penicillium expansum] |
| GL_Gan1_GLEAN_10000553 | COG0596 Predicted hydrolases or acyltransferases (alpha/beta hydrolase superfamily) R General function prediction only ; | hypothetical protein PITC_037270 [Penicillium italicum] |
| GL_Gan1_GLEAN_10000555 | COG0604 NADPH:quinone reductase and related Zn-dependent oxidoreductases CR Energy production and conversion ; General function prediction only ; | Polyketide synthase, enoylreductase [Penicillium italicum] |
| GL_Gan1_GLEAN_10000560 | COG2515 1-aminocyclopropane-1-carboxylate deaminase E Amino acid transport and metabolism ; | 1-aminocyclopropane-1-carboxylate deaminase [Penicillium expansum] |
| GL_Gan1_GLEAN_10001825 | COG3560 Predicted oxidoreductase related to nitroreductase R General function prediction only ; | hypothetical protein PITC_054850 [Penicillium italicum] |
| GL_Gan1_GLEAN_10009160 | COG0133 Tryptophan synthase beta chain E Amino acid transport and metabolism ; | Tryptophan synthase, beta chain [Penicillium italicum] |
| GL_Gan1_GLEAN_10009166 | COG2220 Predicted Zn-dependent hydrolases of the beta-lactamase fold R General function prediction only ; | hypothetical protein PITC_085030 [Penicillium italicum] |
| GL_Gan1_GLEAN_10009169 | COG3119 Arylsulfatase A and related enzymes P Inorganic ion transport and metabolism ; | Sulfatase [Penicillium italicum] |
| GL_Gan1_GLEAN_10009177 | COG2141 Coenzyme F420-dependent N5,N10-methylene tetrahydromethanopterin reductase and related flavin-dependent oxidoreductases C Energy production and conversion ; | Nitrilotriacetate monooxygenase component A/pristinamycin IIA synthase subunit A [Penicillium expansum] |
| GL_Gan1_GLEAN_10009180 | COG2141 Coenzyme F420-dependent N5,N10-methylene tetrahydromethanopterin reductase and related flavin-dependent oxidoreductases C Energy production and conversion ; | Nitrilotriacetate monooxygenase component A/pristinamycin IIA synthase subunit A [Penicillium italicum] |
| GL_Gan1_GLEAN_10009198 | COG1028 Dehydrogenases with different specificities (related to short-chain alcohol dehydrogenases) IQR Lipid transport and metabolism ; Secondary metabolites biosynthesis, transport and catabolism ; General function prediction only ; | Glucose/ribitol dehydrogenase [Penicillium italicum] |
| GL_Gan1_GLEAN_10009203 | COG0604 NADPH:quinone reductase and related Zn-dependent oxidoreductases CR Energy production and conversion ; General function prediction only ; | Alcohol dehydrogenase superfamily, zinc-type [Penicillium italicum] |
| GL_Gan1_GLEAN_10009259 | COG0218 Predicted GTPase R General function prediction only ; | GTP-binding protein, ribosome biogenesis, YsxC [Penicillium italicum] |
| GL_Gan1_GLEAN_10009271 | COG0108 3,4-dihydroxy-2-butanone 4-phosphate synthase H Coenzyme transport and metabolism ; | 3,4-dihydroxy-2-butanone 4-phosphate synthase, RibB [Penicillium italicum] |
| GL_Gan1_GLEAN_10009371 | COG1062 Zn-dependent alcohol dehydrogenases, class III C Energy production and conversion ; | Alcohol dehydrogenase superfamily, zinc-type [Penicillium italicum] |
| GL_Gan1_GLEAN_10009378 | COG4122 Predicted O-methyltransferase R General function prediction only ; | O-methyltransferase, family 3 [Penicillium italicum] |
| GL_Gan1_GLEAN_10009388 | COG1028 Dehydrogenases with different specificities (related to short-chain alcohol dehydrogenases) IQR Lipid transport and metabolism ; Secondary metabolites biosynthesis, transport and catabolism ; General function prediction only ; | Short-chain dehydrogenase/reductase SDR [Penicillium italicum] |
| GL_Gan1_GLEAN_10009399 | COG3277 RNA-binding protein involved in rRNA processing J Translation, ribosomal structure and biogenesis ; | Translation elongation/initiation factor/Ribosomal, beta-barrel [Penicillium italicum] |
| GL_Gan1_GLEAN_10001641 | COG1012 NAD-dependent aldehyde dehydrogenases C Energy production and conversion ; | Aldehyde dehydrogenase, N-terminal [Penicillium italicum] |
| GL_Gan1_GLEAN_10001684 | COG1501 Alpha-glucosidases, family 31 of glycosyl hydrolases G Carbohydrate transport and metabolism ; | Glycoside hydrolase, family 31 [Penicillium expansum] |
| GL_Gan1_GLEAN_10003172 | COG2175 Probable taurine catabolism dioxygenase Q Secondary metabolites biosynthesis, transport and catabolism ; | Taurine catabolism dioxygenase TauD/TfdA [Penicillium italicum] |
| GL_Gan1_GLEAN_10003193 | COG0318 Acyl-CoA synthetases (AMP-forming)/AMP-acid ligases II IQ Lipid transport and metabolism ; Secondary metabolites biosynthesis, transport and catabolism ; | AMP-dependent synthetase/ligase [Penicillium italicum] |
| GL_Gan1_GLEAN_10003223 | COG0452 Phosphopantothenoylcysteine synthetase/decarboxylase H Coenzyme transport and metabolism ; | Flavoprotein [Penicillium italicum] |
| GL_Gan1_GLEAN_10008907 | COG0274 Deoxyribose-phosphate aldolase F Nucleotide transport and metabolism ; | Aldolase-type TIM barrel [Penicillium italicum] |
| GL_Gan1_GLEAN_10008911 | COG0560 Phosphoserine phosphatase E Amino acid transport and metabolism ; | Phosphoserine phosphatase, domain 2 [Penicillium italicum] |
| GL_Gan1_GLEAN_10008920 | COG0693 Putative intracellular protease/amidase R General function prediction only ; | ThiJ/PfpI [Penicillium italicum] |
| GL_Gan1_GLEAN_10008959 | COG2130 Putative NADP-dependent oxidoreductases R General function prediction only ; | Alcohol dehydrogenase, C-terminal [Penicillium expansum] |
| GL_Gan1_GLEAN_10008969 | NA | Protein of unknown function DUF1917 [Penicillium expansum] |
| GL_Gan1_GLEAN_10008986 | NA | hypothetical protein PITC_002330 [Penicillium italicum] |
| GL_Gan1_GLEAN_10008994 | COG2303 Choline dehydrogenase and related flavoproteins E Amino acid transport and metabolism ; | glucose oxidase, partial [Penicillium expansum] |
| GL_Gan1_GLEAN_10009000 | COG0252 L-asparaginase/archaeal Glu-tRNAGln amidotransferase subunit D EJ Amino acid transport and metabolism ; Translation, ribosomal structure and biogenesis ; | L-asparaginase, type II [Penicillium italicum] |
| GL_Gan1_GLEAN_10009001 | COG3476 Tryptophan-rich sensory protein (mitochondrial benzodiazepine receptor homolog) T Signal transduction mechanisms ; | TspO/MBR-related protein [Penicillium italicum] |
| GL_Gan1_GLEAN_10009012 | COG0388 Predicted amidohydrolase R General function prediction only ; | Carbon-nitrogen hydrolase [Penicillium italicum] |
| GL_Gan1_GLEAN_10009029 | COG0252 L-asparaginase/archaeal Glu-tRNAGln amidotransferase subunit D EJ Amino acid transport and metabolism ; Translation, ribosomal structure and biogenesis ; | L-asparaginase, type II [Penicillium italicum] |
| GL_Gan1_GLEAN_10009036 | COG2421 Predicted acetamidase/formamidase C Energy production and conversion ; | Acetamidase/Formamidase [Penicillium italicum] |
| GL_Gan1_GLEAN_10009128 | COG1028 Dehydrogenases with different specificities (related to short-chain alcohol dehydrogenases) IQR Lipid transport and metabolism ; Secondary metabolites biosynthesis, transport and catabolism ; General function prediction only ; | Short-chain dehydrogenase/reductase SDR [Penicillium italicum] |
| GL_Gan1_GLEAN_10009139 | COG1087 UDP-glucose 4-epimerase M Cell wall/membrane/envelope biogenesis ; | NAD-dependent epimerase/dehydratase [Penicillium italicum] |
| GL_Gan1_GLEAN_10009158 | COG5383 Uncharacterized protein conserved in bacteria S Function unknown ; | protein of unknown function DUF1338 [Penicillium italicum] |
| GL_Gan1_GLEAN_10006831 | COG1028 Dehydrogenases with different specificities (related to short-chain alcohol dehydrogenases) IQR Lipid transport and metabolism ; Secondary metabolites biosynthesis, transport and catabolism ; General function prediction only ; | Short-chain dehydrogenase/reductase SDR [Penicillium italicum] |
| GL_Gan1_GLEAN_10006885 | COG1304 L-lactate dehydrogenase (FMN-dependent) and related alpha-hydroxy acid dehydrogenases C Energy production and conversion ; | Aldolase-type TIM barrel [Penicillium italicum] |
| GL_Gan1_GLEAN_10006923 | COG1028 Dehydrogenases with different specificities (related to short-chain alcohol dehydrogenases) IQR Lipid transport and metabolism ; Secondary metabolites biosynthesis, transport and catabolism ; General function prediction only ; | Glucose/ribitol dehydrogenase [Penicillium italicum] |
| GL_Gan1_GLEAN_10008523 | COG0673 Predicted dehydrogenases and related proteins R General function prediction only ; | Oxidoreductase, N-terminal [Penicillium italicum] |
| GL_Gan1_GLEAN_10008565 | COG0666 FOG: Ankyrin repeat R General function prediction only ; | hypothetical protein PITC_020550 [Penicillium italicum] |
| GL_Gan1_GLEAN_10008567 | COG1670 Acetyltransferases, including N-acetylases of ribosomal proteins J Translation, ribosomal structure and biogenesis ; | Acyl-CoA N-acyltransferase [Penicillium italicum] |
| GL_Gan1_GLEAN_10008645 | COG0596 Predicted hydrolases or acyltransferases (alpha/beta hydrolase superfamily) R General function prediction only ; | hypothetical protein PITC_080860 [Penicillium italicum] |
| GL_Gan1_GLEAN_10008675 | COG0454 Histone acetyltransferase HPA2 and related acetyltransferases KR Transcription ; General function prediction only ; | Acyl-CoA N-acyltransferase [Penicillium italicum] |
| GL_Gan1_GLEAN_10008704 | COG3119 Arylsulfatase A and related enzymes P Inorganic ion transport and metabolism ; | Alkaline phosphatase-like, alpha/beta/alpha [Penicillium italicum] |
| GL_Gan1_GLEAN_10008709 | COG0314 Molybdopterin converting factor, large subunit H Coenzyme transport and metabolism ; | Glycoside hydrolase, superfamily [Penicillium italicum] |
| GL_Gan1_GLEAN_10008712 | COG0654 2-polyprenyl-6-methoxyphenol hydroxylase and related FAD-dependent oxidoreductases HC Coenzyme transport and metabolism ; Energy production and conversion ; | Monooxygenase, FAD-binding [Penicillium italicum] |
| GL_Gan1_GLEAN_10008719 | COG1237 Metal-dependent hydrolases of the beta-lactamase superfamily II R General function prediction only ; | hypothetical protein PITC_081560 [Penicillium italicum] |
| GL_Gan1_GLEAN_10008741 | COG0596 Predicted hydrolases or acyltransferases (alpha/beta hydrolase superfamily) R General function prediction only ; | Alpha/beta hydrolase fold-1 [Penicillium expansum] |
| GL_Gan1_GLEAN_10008802 | COG0461 Orotate phosphoribosyltransferase F Nucleotide transport and metabolism ; | Orotate phosphoribosyltransferase [Penicillium italicum] |
| GL_Gan1_GLEAN_10008808 | COG0318 Acyl-CoA synthetases (AMP-forming)/AMP-acid ligases II IQ Lipid transport and metabolism ; Secondary metabolites biosynthesis, transport and catabolism ; | AMP-dependent synthetase/ligase [Penicillium italicum] |
| GL_Gan1_GLEAN_10008811 | COG1680 Beta-lactamase class C and other penicillin binding proteins V Defense mechanisms ; | Peptidase S12, aminopeptidase DmpB, domain C [Penicillium italicum] |
| GL_Gan1_GLEAN_10005877 | COG2319 FOG: WD40 repeat R General function prediction only ; | Peptidase S9A/B/C, oligopeptidase, N-terminal beta-propeller [Penicillium italicum] |
| GL_Gan1_GLEAN_10005895 | COG1250 3-hydroxyacyl-CoA dehydrogenase I Lipid transport and metabolism ; | Dehydrogenase, multihelical [Penicillium italicum] |
| GL_Gan1_GLEAN_10005899 | COG1304 L-lactate dehydrogenase (FMN-dependent) and related alpha-hydroxy acid dehydrogenases C Energy production and conversion ; | Aldolase-type TIM barrel [Penicillium italicum] |
| GL_Gan1_GLEAN_10005915 | NA | protein of unknown function DUF1771 [Penicillium italicum] |
| GL_Gan1_GLEAN_10005925 | COG0604 NADPH:quinone reductase and related Zn-dependent oxidoreductases CR Energy production and conversion ; General function prediction only ; | Alcohol dehydrogenase, C-terminal [Penicillium italicum] |
| GL_Gan1_GLEAN_10005934 | COG3938 Proline racemase E Amino acid transport and metabolism ; | Proline racemase [Penicillium italicum] |
| GL_Gan1_GLEAN_10005965 | COG0753 Catalase P Inorganic ion transport and metabolism ; | Catalase, mono-functional, heme-containing [Penicillium expansum] |
| GL_Gan1_GLEAN_10005979 | COG0833 Amino acid transporters E Amino acid transport and metabolism ; | Amino acid/polyamine transporter I [Penicillium italicum] |
| GL_Gan1_GLEAN_10001912 | NA | Glycoside hydrolase, superfamily [Penicillium italicum] |
| GL_Gan1_GLEAN_10001913 | COG2377 Predicted molecular chaperone distantly related to HSP70-fold metalloproteases O Posttranslational modification, protein turnover, chaperones ; | Anhydro-N-acetylmuramic acid kinase [Penicillium italicum] |
| GL_Gan1_GLEAN_10001919 | COG0794 Predicted sugar phosphate isomerase involved in capsule formation M Cell wall/membrane/envelope biogenesis ; | Sugar isomerase (SIS) [Penicillium italicum] |
| GL_Gan1_GLEAN_10001932 | COG1764 Predicted redox protein, regulator of disulfide bond formation O Posttranslational modification, protein turnover, chaperones ; | Peroxiredoxin, OsmC-like protein [Penicillium italicum] |
| GL_Gan1_GLEAN_10000851 | COG0698 Ribose 5-phosphate isomerase RpiB G Carbohydrate transport and metabolism ; | Pc22g21440 [Penicillium rubens Wisconsin 54-1255] |
| GL_Gan1_GLEAN_10000855 | COG0473 Isocitrate/isopropylmalate dehydrogenase CE Energy production and conversion ; Amino acid transport and metabolism ; | Isocitrate/isopropylmalate dehydrogenase [Penicillium roqueforti FM164] |
| GL_Gan1_GLEAN_10000874 | COG0500 SAM-dependent methyltransferases QR Secondary metabolites biosynthesis, transport and catabolism ; General function prediction only ; | Methyltransferase type 11 [Penicillium italicum] |
| GL_Gan1_GLEAN_10000875 | COG3957 Phosphoketolase G Carbohydrate transport and metabolism ; | Xylulose 5-phosphate/Fructose 6-phosphate phosphoketolase, N-terminal [Penicillium italicum] |
| GL_Gan1_GLEAN_10000011 | COG5640 Secreted trypsin-like serine protease O Posttranslational modification, protein turnover, chaperones ; | Peptidase S1A, chymotrypsin-type [Penicillium italicum] |
| GL_Gan1_GLEAN_10003844 | COG5564 Predicted TIM-barrel enzyme, possibly a dioxygenase R General function prediction only ; | Aldolase-type TIM barrel [Penicillium italicum] |
| GL_Gan1_GLEAN_10003868 | NA | Endonuclease/exonuclease/phosphatase [Penicillium italicum] |
| GL_Gan1_GLEAN_10003873 | COG1011 Predicted hydrolase (HAD superfamily) R General function prediction only ; | Haloacid dehalogenase/epoxide hydrolase [Penicillium italicum] |
| GL_Gan1_GLEAN_10003879 | COG3486 Lysine/ornithine N-monooxygenase Q Secondary metabolites biosynthesis, transport and catabolism ; | FAD-dependent pyridine nucleotide-disulfide oxidoreductase [Penicillium italicum] |
| GL_Gan1_GLEAN_10002062 | NA | Protein of unknown function DUF3468 [Penicillium italicum] |
| GL_Gan1_GLEAN_10002065 | COG1064 Zn-dependent alcohol dehydrogenases R General function prediction only ; | Alcohol dehydrogenase superfamily, zinc-type [Penicillium italicum] |
| GL_Gan1_GLEAN_10002066 | COG4702 Uncharacterized conserved protein S Function unknown ; | protein of unknown function DUF336 [Penicillium italicum] |
| GL_Gan1_GLEAN_10002088 | NA | Lipase, secreted [Penicillium italicum] |
| GL_Gan1_GLEAN_10000814 | COG0665 Glycine/D-amino acid oxidases (deaminating) E Amino acid transport and metabolism ; | D-amino-acid oxidase [Penicillium italicum] |
| GL_Gan1_GLEAN_10000815 | COG0388 Predicted amidohydrolase R General function prediction only ; | Carbon-nitrogen hydrolase [Penicillium italicum] |
| GL_Gan1_GLEAN_10000826 | COG0665 Glycine/D-amino acid oxidases (deaminating) E Amino acid transport and metabolism ; | FAD dependent oxidoreductase [Penicillium italicum] |
| GL_Gan1_GLEAN_10001371 | COG2141 Coenzyme F420-dependent N5,N10-methylene tetrahydromethanopterin reductase and related flavin-dependent oxidoreductases C Energy production and conversion ; | Nitrilotriacetate monooxygenase component A/pristinamycin IIA synthase subunit A [Penicillium italicum] |
| GL_Gan1_GLEAN_10001381 | COG0028 Thiamine pyrophosphate-requiring enzymes [acetolactate synthase, pyruvate dehydrogenase (cytochrome), glyoxylate carboligase, phosphonopyruvate decarboxylase] EH Amino acid transport and metabolism ; Coenzyme transport and metabolism ; | Thiamine pyrophosphate enzyme, C-terminal TPP-binding [Penicillium italicum] |
| GL_Gan1_GLEAN_10001386 | COG0413 Ketopantoate hydroxymethyltransferase H Coenzyme transport and metabolism ; | Pyruvate/Phosphoenolpyruvate kinase [Penicillium italicum] |
| GL_Gan1_GLEAN_10000187 | COG0057 Glyceraldehyde-3-phosphate dehydrogenase/erythrose-4-phosphate dehydrogenase G Carbohydrate transport and metabolism ; | Glyceraldehyde/Erythrose phosphate dehydrogenase family [Penicillium italicum] |
| GL_Gan1_GLEAN_10000197 | COG0491 Zn-dependent hydrolases, including glyoxylases R General function prediction only ; | hypothetical protein PITC_049990 [Penicillium italicum] |
| GL_Gan1_GLEAN_10000198 | COG1621 Beta-fructosidases (levanase/invertase) G Carbohydrate transport and metabolism ; | Glycoside hydrolase, family 32 [Penicillium italicum] |
| GL_Gan1_GLEAN_10002670 | COG1028 Dehydrogenases with different specificities (related to short-chain alcohol dehydrogenases) IQR Lipid transport and metabolism ; Secondary metabolites biosynthesis, transport and catabolism ; General function prediction only ; | Glucose/ribitol dehydrogenase [Penicillium italicum] |
| GL_Gan1_GLEAN_10002677 | COG2202 FOG: PAS/PAC domain T Signal transduction mechanisms ; | PAS-associated, C-terminal [Penicillium italicum] |
| GL_Gan1_GLEAN_10002679 | COG2021 Homoserine acetyltransferase E Amino acid transport and metabolism ; | hypothetical protein PITC_000570 [Penicillium italicum] |
| GL_Gan1_GLEAN_10002688 | COG0045 Succinyl-CoA synthetase, beta subunit C Energy production and conversion ; | ATP-grasp fold, subdomain 2 [Penicillium expansum] |
| GL_Gan1_GLEAN_10002483 | COG5053 Translation initiation factor 4E (eIF-4E) J Translation, ribosomal structure and biogenesis ; | Translation Initiation factor eIF- 4e [Penicillium italicum] |
| GL_Gan1_GLEAN_10002516 | COG0688 Phosphatidylserine decarboxylase I Lipid transport and metabolism ; | Phophatidylserine decarboxylase [Penicillium italicum] |
| GL_Gan1_GLEAN_10002529 | COG1902 NADH:flavin oxidoreductases, Old Yellow Enzyme family C Energy production and conversion ; | Aldolase-type TIM barrel [Penicillium italicum] |
| GL_Gan1_GLEAN_10004181 | COG2320 Uncharacterized conserved protein S Function unknown ; | Uncharacterized protein family UPF0157 [Penicillium italicum] |
| GL_Gan1_GLEAN_10004216 | COG0500 SAM-dependent methyltransferases QR Secondary metabolites biosynthesis, transport and catabolism ; General function prediction only ; | hypothetical protein PITC_012310 [Penicillium italicum] |
| GL_Gan1_GLEAN_10006374 | COG0346 Lactoylglutathione lyase and related lyases E Amino acid transport and metabolism ; | hypothetical protein PEXP_003290 [Penicillium expansum] |
| GL_Gan1_GLEAN_10006469 | NA | hypothetical protein PITC_062780 [Penicillium italicum] |
| GL_Gan1_GLEAN_10003073 | COG1472 Beta-glucosidase-related glycosidases G Carbohydrate transport and metabolism ; | Glycoside hydrolase, superfamily [Penicillium italicum] |
| GL_Gan1_GLEAN_10003089 | COG0604 NADPH:quinone reductase and related Zn-dependent oxidoreductases CR Energy production and conversion ; General function prediction only ; | Polyketide synthase, enoylreductase [Penicillium italicum] |
| GL_Gan1_GLEAN_10003096 | COG0494 NTP pyrophosphohydrolases including oxidative damage repair enzymes LR Replication, recombination and repair ; General function prediction only ; | Nudix/MutT family protein [Penicillium digitatum PHI26] |
| GL_Gan1_GLEAN_10003120 | COG2873 O-acetylhomoserine sulfhydrylase E Amino acid transport and metabolism ; | Pyridoxal phosphate-dependent transferase, major region, subdomain 2 [Penicillium italicum] |
| GL_Gan1_GLEAN_10003159 | NA | Protein of unknown function DUF284, transmembrane eukaryotic [Penicillium italicum] |
| GL_Gan1_GLEAN_10000357 | NA | hypothetical protein PITC_076050 [Penicillium italicum] |
| GL_Gan1_GLEAN_10003691 | COG1028 Dehydrogenases with different specificities (related to short-chain alcohol dehydrogenases) IQR Lipid transport and metabolism ; Secondary metabolites biosynthesis, transport and catabolism ; General function prediction only ; | Short-chain dehydrogenase/reductase SDR [Penicillium italicum] |
| GL_Gan1_GLEAN_10003693 | COG4948 L-alanine-DL-glutamate epimerase and related enzymes of enolase superfamily MR Cell wall/membrane/envelope biogenesis ; General function prediction only ; | Mandelate racemase/muconate lactonizing enzyme, C-terminal [Penicillium italicum] |
| GL_Gan1_GLEAN_10007808 | COG4305 Endoglucanase C-terminal domain/subunit and related proteins G Carbohydrate transport and metabolism ; | Barwin-related endoglucanase [Penicillium italicum] |
| GL_Gan1_GLEAN_10007812 | COG0214 Pyridoxine biosynthesis enzyme H Coenzyme transport and metabolism ; | Vitamin B6 biosynthesis protein [Penicillium expansum] |
| GL_Gan1_GLEAN_10007894 | COG1028 Dehydrogenases with different specificities (related to short-chain alcohol dehydrogenases) IQR Lipid transport and metabolism ; Secondary metabolites biosynthesis, transport and catabolism ; General function prediction only ; | Glucose/ribitol dehydrogenase [Penicillium italicum] |
| GL_Gan1_GLEAN_10007895 | COG2234 Predicted aminopeptidases R General function prediction only ; | Peptidase M28 [Penicillium italicum] |
| GL_Gan1_GLEAN_10007905 | COG1960 Acyl-CoA dehydrogenases I Lipid transport and metabolism ; | Acyl-CoA dehydrogenase, N-terminal [Penicillium italicum] |
| GL_Gan1_GLEAN_10007935 | COG1028 Dehydrogenases with different specificities (related to short-chain alcohol dehydrogenases) IQR Lipid transport and metabolism ; Secondary metabolites biosynthesis, transport and catabolism ; General function prediction only ; | Short-chain dehydrogenase/reductase SDR [Penicillium italicum] |
| GL_Gan1_GLEAN_10007937 | COG0667 Predicted oxidoreductases (related to aryl-alcohol dehydrogenases) C Energy production and conversion ; | Potassium channel, voltage-dependent, beta subunit, KCNAB-like protein [Penicillium italicum] |
| GL_Gan1_GLEAN_10007958 | COG2957 Peptidylarginine deiminase and related enzymes E Amino acid transport and metabolism ; | Peptidyl-arginine deiminase, Porphyromonas-type [Penicillium italicum] |
| GL_Gan1_GLEAN_10007982 | COG2086 Electron transfer flavoprotein, beta subunit C Energy production and conversion ; | Electron transfer flavoprotein, alpha/beta-subunit, N-terminal [Penicillium italicum] |
| GL_Gan1_GLEAN_10001193 | COG0451 Nucleoside-diphosphate-sugar epimerases MG Cell wall/membrane/envelope biogenesis ; Carbohydrate transport and metabolism ; | hypothetical protein PEX1_039450 [Penicillium expansum] |
| GL_Gan1_GLEAN_10001234 | COG1472 Beta-glucosidase-related glycosidases G Carbohydrate transport and metabolism ; | Glycoside hydrolase, superfamily [Penicillium italicum] |
| GL_Gan1_GLEAN_10001235 | COG0625 Glutathione S-transferase O Posttranslational modification, protein turnover, chaperones ; | Glutathione S-transferase, N-terminal [Penicillium italicum] |
| GL_Gan1_GLEAN_10000111 | COG3791 Uncharacterized conserved protein S Function unknown ; | Glutathione-dependent formaldehyde-activating enzyme [Penicillium italicum] |
| GL_Gan1_GLEAN_10000087 | COG1028 Dehydrogenases with different specificities (related to short-chain alcohol dehydrogenases) IQR Lipid transport and metabolism ; Secondary metabolites biosynthesis, transport and catabolism ; General function prediction only ; | Short-chain dehydrogenase/reductase SDR [Penicillium italicum] |
| GL_Gan1_GLEAN_10001061 | COG1670 Acetyltransferases, including N-acetylases of ribosomal proteins J Translation, ribosomal structure and biogenesis ; | Acyl-CoA N-acyltransferase [Penicillium italicum] |
| GL_Gan1_GLEAN_10001948 | COG2957 Peptidylarginine deiminase and related enzymes E Amino acid transport and metabolism ; | Peptidyl-arginine deiminase, Porphyromonas-type [Penicillium italicum] |
| GL_Gan1_GLEAN_10001965 | COG1053 Succinate dehydrogenase/fumarate reductase, flavoprotein subunit C Energy production and conversion ; | Pc12g12200 [Penicillium rubens Wisconsin 54-1255] |
| GL_Gan1_GLEAN_10001984 | COG3665 Uncharacterized conserved protein S Function unknown ; | protein of unknown function DUF1989 [Penicillium italicum] |
| GL_Gan1_GLEAN_10001987 | COG2079 Uncharacterized protein involved in propionate catabolism R General function prediction only ; | MmgE/PrpD [Penicillium italicum] |
| GL_Gan1_GLEAN_10007175 | COG0604 NADPH:quinone reductase and related Zn-dependent oxidoreductases CR Energy production and conversion ; General function prediction only ; | Alcohol dehydrogenase superfamily, zinc-type [Penicillium italicum] |
| GL_Gan1_GLEAN_10007189 | NA | hypothetical protein PITC_027110 [Penicillium italicum] |
| GL_Gan1_GLEAN_10007233 | COG3250 Beta-galactosidase/beta-glucuronidase G Carbohydrate transport and metabolism ; | Glycoside hydrolase, family 2, N-terminal [Penicillium italicum] |
| GL_Gan1_GLEAN_10007234 | COG0388 Predicted amidohydrolase R General function prediction only ; | Carbon-nitrogen hydrolase [Penicillium italicum] |
| GL_Gan1_GLEAN_10007241 | COG0129 Dihydroxyacid dehydratase/phosphogluconate dehydratase EG Amino acid transport and metabolism ; Carbohydrate transport and metabolism ; | Dihydroxy-acid dehydratase [Penicillium italicum] |
| GL_Gan1_GLEAN_10002633 | COG0457 FOG: TPR repeat R General function prediction only ; | Tetratricopeptide-like helical [Penicillium italicum] |
| GL_Gan1_GLEAN_10002658 | COG3265 Gluconate kinase G Carbohydrate transport and metabolism ; | Shikimate kinase [Penicillium italicum] |
| GL_Gan1_GLEAN_10001601 | COG0596 Predicted hydrolases or acyltransferases (alpha/beta hydrolase superfamily) R General function prediction only ; | Alpha/beta hydrolase fold-1 [Penicillium italicum] |
| GL_Gan1_GLEAN_10000723 | COG3507 Beta-xylosidase G Carbohydrate transport and metabolism ; | Glycoside hydrolase, family 43 [Penicillium italicum] |
| GL_Gan1_GLEAN_10000730 | NA | hypothetical protein PITC_021640 [Penicillium italicum] |
| GL_Gan1_GLEAN_10000732 | COG5077 Ubiquitin carboxyl-terminal hydrolase O Posttranslational modification, protein turnover, chaperones ; | Zinc finger, RING-type [Penicillium expansum] |
| GL_Gan1_GLEAN_10000737 | COG1028 Dehydrogenases with different specificities (related to short-chain alcohol dehydrogenases) IQR Lipid transport and metabolism ; Secondary metabolites biosynthesis, transport and catabolism ; General function prediction only ; | Glucose/ribitol dehydrogenase [Penicillium italicum] |
| GL_Gan1_GLEAN_10000751 | COG0656 Aldo/keto reductases, related to diketogulonate reductase R General function prediction only ; | Aldo/keto reductase [Penicillium italicum] |
| GL_Gan1_GLEAN_10004667 | COG1028 Dehydrogenases with different specificities (related to short-chain alcohol dehydrogenases) IQR Lipid transport and metabolism ; Secondary metabolites biosynthesis, transport and catabolism ; General function prediction only ; | Short-chain dehydrogenase/reductase SDR [Penicillium italicum] |
| GL_Gan1_GLEAN_10004774 | COG0035 Uracil phosphoribosyltransferase F Nucleotide transport and metabolism ; | hypothetical protein PITC_083080 [Penicillium italicum] |
| GL_Gan1_GLEAN_10004884 | COG0717 Deoxycytidine deaminase F Nucleotide transport and metabolism ; | DeoxyUTP pyrophosphatase [Penicillium expansum] |
| GL_Gan1_GLEAN_10004906 | COG3345 Alpha-galactosidase G Carbohydrate transport and metabolism ; | Aldolase-type TIM barrel [Penicillium italicum] |
| GL_Gan1_GLEAN_10002306 | COG0657 Esterase/lipase I Lipid transport and metabolism ; | Alpha/beta hydrolase fold-3 [Penicillium italicum] |
| GL_Gan1_GLEAN_10002334 | COG0288 Carbonic anhydrase P Inorganic ion transport and metabolism ; | Carbonic anhydrase [Penicillium italicum] |
| GL_Gan1_GLEAN_10002416 | COG4225 Predicted unsaturated glucuronyl hydrolase involved in regulation of bacterial surface properties, and related proteins R General function prediction only ; | TPA: cell wall glycosyl hydrolase YteR, putative (AFU_orthologue; AFUA_2G14630) [Aspergillus nidulans FGSC A4] |
| GL_Gan1_GLEAN_10002449 | COG1231 Monoamine oxidase E Amino acid transport and metabolism ; | Flavin amine oxidase [Penicillium italicum] |
| GL_Gan1_GLEAN_10002458 | COG0693 Putative intracellular protease/amidase R General function prediction only ; | Pc20g03290 [Penicillium rubens Wisconsin 54-1255] |
| GL_Gan1_GLEAN_10004036 | COG1028 Dehydrogenases with different specificities (related to short-chain alcohol dehydrogenases) IQR Lipid transport and metabolism ; Secondary metabolites biosynthesis, transport and catabolism ; General function prediction only ; | Glucose/ribitol dehydrogenase [Penicillium italicum] |
| GL_Gan1_GLEAN_10004039 | COG1028 Dehydrogenases with different specificities (related to short-chain alcohol dehydrogenases) IQR Lipid transport and metabolism ; Secondary metabolites biosynthesis, transport and catabolism ; General function prediction only ; | Short-chain dehydrogenase/reductase SDR [Penicillium italicum] |
| GL_Gan1_GLEAN_10004070 | COG0154 Asp-tRNAAsn/Glu-tRNAGln amidotransferase A subunit and related amidases J Translation, ribosomal structure and biogenesis ; | Amidase [Penicillium italicum] |
| GL_Gan1_GLEAN_10004096 | COG0625 Glutathione S-transferase O Posttranslational modification, protein turnover, chaperones ; | Glutathione S-transferase/chloride channel, C-terminal [Penicillium italicum] |
| GL_Gan1_GLEAN_10004105 | COG3250 Beta-galactosidase/beta-glucuronidase G Carbohydrate transport and metabolism ; | Glycoside hydrolase, family 2, N-terminal [Penicillium italicum] |
| GL_Gan1_GLEAN_10002729 | COG0043 3-polyprenyl-4-hydroxybenzoate decarboxylase and related decarboxylases H Coenzyme transport and metabolism ; | Carboxylyase-like protein [Penicillium italicum] |
| GL_Gan1_GLEAN_10002730 | COG0163 3-polyprenyl-4-hydroxybenzoate decarboxylase H Coenzyme transport and metabolism ; | Phenylacrylic acid decarboxylase [Penicillium italicum] |
| GL_Gan1_GLEAN_10002732 | COG1053 Succinate dehydrogenase/fumarate reductase, flavoprotein subunit C Energy production and conversion ; | hypothetical protein PITC_068790 [Penicillium italicum] |
| GL_Gan1_GLEAN_10002739 | COG1434 Uncharacterized conserved protein S Function unknown ; | protein of unknown function DUF218 [Penicillium italicum] |
| GL_Gan1_GLEAN_10002758 | COG0388 Predicted amidohydrolase R General function prediction only ; | Carbon-nitrogen hydrolase [Penicillium italicum] |
| GL_Gan1_GLEAN_10001487 | COG1250 3-hydroxyacyl-CoA dehydrogenase I Lipid transport and metabolism ; | Dehydrogenase, multihelical [Penicillium expansum] |
| GL_Gan1_GLEAN_10001498 | COG1028 Dehydrogenases with different specificities (related to short-chain alcohol dehydrogenases) IQR Lipid transport and metabolism ; Secondary metabolites biosynthesis, transport and catabolism ; General function prediction only ; | Short-chain dehydrogenase/reductase SDR [Penicillium italicum] |
| GL_Gan1_GLEAN_10003006 | COG0604 NADPH:quinone reductase and related Zn-dependent oxidoreductases CR Energy production and conversion ; General function prediction only ; | Polyketide synthase, enoylreductase [Penicillium italicum] |
| GL_Gan1_GLEAN_10002233 | COG3491 Isopenicillin N synthase and related dioxygenases R General function prediction only ; | Major facilitator superfamily domain, general substrate transporter [Penicillium italicum] |
| GL_Gan1_GLEAN_10004376 | COG0161 Adenosylmethionine-8-amino-7-oxononanoate aminotransferase H Coenzyme transport and metabolism ; | Pyridoxal phosphate-dependent transferase, major region, subdomain 2 [Penicillium italicum] |
| GL_Gan1_GLEAN_10004380 | COG0154 Asp-tRNAAsn/Glu-tRNAGln amidotransferase A subunit and related amidases J Translation, ribosomal structure and biogenesis ; | Amidase [Penicillium expansum] |
| GL_Gan1_GLEAN_10004402 | COG0726 Predicted xylanase/chitin deacetylase G Carbohydrate transport and metabolism ; | Glycoside hydrolase/deacetylase, beta/alpha-barrel [Penicillium italicum] |
| GL_Gan1_GLEAN_10004406 | COG0625 Glutathione S-transferase O Posttranslational modification, protein turnover, chaperones ; | Glutathione S-transferase/chloride channel, C-terminal [Penicillium italicum] |
| GL_Gan1_GLEAN_10004407 | COG1052 Lactate dehydrogenase and related dehydrogenases CHR Energy production and conversion ; Coenzyme transport and metabolism ; General function prediction only ; | D-isomer specific 2-hydroxyacid dehydrogenase, NAD-binding [Penicillium italicum] |
| GL_Gan1_GLEAN_10004431 | COG3836 2,4-dihydroxyhept-2-ene-1,7-dioic acid aldolase G Carbohydrate transport and metabolism ; | Pyruvate/Phosphoenolpyruvate kinase [Penicillium italicum] |
| GL_Gan1_GLEAN_10001243 | COG0757 3-dehydroquinate dehydratase II E Amino acid transport and metabolism ; | Dehydroquinase, class II [Penicillium italicum] |
| GL_Gan1_GLEAN_10001249 | COG0673 Predicted dehydrogenases and related proteins R General function prediction only ; | Oxidoreductase, N-terminal [Penicillium expansum] |
| GL_Gan1_GLEAN_10003320 | COG2072 Predicted flavoprotein involved in K+ transport P Inorganic ion transport and metabolism ; | hypothetical protein PITC_002890 [Penicillium italicum] |
| GL_Gan1_GLEAN_10003348 | NA | Glycoside hydrolase, family 28 [Penicillium italicum] |
| GL_Gan1_GLEAN_10001055 | COG0515 Serine/threonine protein kinase RTKL General function prediction only ; Signal transduction mechanisms ; Transcription ; Replication, recombination and repair ; | hypothetical protein PITC_029360 [Penicillium italicum] |
| GL_Gan1_GLEAN_10000298 | COG1028 Dehydrogenases with different specificities (related to short-chain alcohol dehydrogenases) IQR Lipid transport and metabolism ; Secondary metabolites biosynthesis, transport and catabolism ; General function prediction only ; | short-chain dehydrogenase, putative [Neosartorya fischeri NRRL 181] |
| GL_Gan1_GLEAN_10004811 | COG1335 Amidases related to nicotinamidase Q Secondary metabolites biosynthesis, transport and catabolism ; | hypothetical protein PEX1_087760 [Penicillium expansum] |
| GL_Gan1_GLEAN_10004832 | COG3321 Polyketide synthase modules and related proteins Q Secondary metabolites biosynthesis, transport and catabolism ; | Acyl transferase/acyl hydrolase/lysophospholipase [Penicillium italicum] |
| GL_Gan1_GLEAN_10004833 | COG0382 4-hydroxybenzoate polyprenyltransferase and related prenyltransferases H Coenzyme transport and metabolism ; | UbiA prenyltransferase family [Penicillium italicum] |
| GL_Gan1_GLEAN_10004834 | COG2159 Predicted metal-dependent hydrolase of the TIM-barrel fold R General function prediction only ; | Cytochrome P450 [Penicillium italicum] |
| GL_Gan1_GLEAN_10004844 | NA | hypothetical protein PITC_009070 [Penicillium italicum] |
| GL_Gan1_GLEAN_10004846 | COG2072 Predicted flavoprotein involved in K+ transport P Inorganic ion transport and metabolism ; | hypothetical protein PITC_021720 [Penicillium italicum] |
| GL_Gan1_GLEAN_10006153 | NA | FERM/acyl-CoA-binding protein, 3-helical bundle [Penicillium italicum] |
| GL_Gan1_GLEAN_10006159 | COG0702 Predicted nucleoside-diphosphate-sugar epimerases MG Cell wall/membrane/envelope biogenesis ; Carbohydrate transport and metabolism ; | hypothetical protein PITC_056920 [Penicillium italicum] |
| GL_Gan1_GLEAN_10006165 | NA | Nucleoporin Nup54 [Penicillium italicum] |
| GL_Gan1_GLEAN_10006181 | COG0604 NADPH:quinone reductase and related Zn-dependent oxidoreductases CR Energy production and conversion ; General function prediction only ; | Polyketide synthase, enoylreductase [Penicillium italicum] |
| GL_Gan1_GLEAN_10006185 | COG2072 Predicted flavoprotein involved in K+ transport P Inorganic ion transport and metabolism ; | hypothetical protein PITC_057180 [Penicillium italicum] |
| GL_Gan1_GLEAN_10006192 | COG0001 Glutamate-1-semialdehyde aminotransferase H Coenzyme transport and metabolism ; | Tetrapyrrole biosynthesis, glutamate-1-semialdehyde aminotransferase [Penicillium italicum] |
| GL_Gan1_GLEAN_10006204 | COG1331 Highly conserved protein containing a thioredoxin domain O Posttranslational modification, protein turnover, chaperones ; | Six-hairpin glycosidase [Penicillium italicum] |
| GL_Gan1_GLEAN_10006272 | COG0451 Nucleoside-diphosphate-sugar epimerases MG Cell wall/membrane/envelope biogenesis ; Carbohydrate transport and metabolism ; | NAD-dependent epimerase/dehydratase [Penicillium italicum] |
| GL_Gan1_GLEAN_10006308 | NA | Molybdenum cofactor biosynthesis protein F [Penicillium italicum] |
| GL_Gan1_GLEAN_10002847 | COG0753 Catalase P Inorganic ion transport and metabolism ; | Catalase, mono-functional, heme-containing [Penicillium italicum] |
| GL_Gan1_GLEAN_10005325 | COG5147 Myb superfamily proteins, including transcription factors and mRNA splicing factors KAD Transcription ; RNA processing and modification ; Cell cycle control, cell division, chromosome partitioning ; | hypothetical protein PITC_046280 [Penicillium italicum] |
| GL_Gan1_GLEAN_10005339 | COG0484 DnaJ-class molecular chaperone with C-terminal Zn finger domain O Posttranslational modification, protein turnover, chaperones ; | Heat shock protein DnaJ, N-terminal [Penicillium italicum] |
| GL_Gan1_GLEAN_10005349 | COG0654 2-polyprenyl-6-methoxyphenol hydroxylase and related FAD-dependent oxidoreductases HC Coenzyme transport and metabolism ; Energy production and conversion ; | Monooxygenase, FAD-binding [Penicillium italicum] |
| GL_Gan1_GLEAN_10005367 | COG3376 High-affinity nickel permease P Inorganic ion transport and metabolism ; | Nickel/cobalt transporter, high-affinity [Penicillium italicum] |
| GL_Gan1_GLEAN_10005379 | COG1335 Amidases related to nicotinamidase Q Secondary metabolites biosynthesis, transport and catabolism ; | hypothetical protein PITC_035170 [Penicillium italicum] |
| GL_Gan1_GLEAN_10005384 | COG2303 Choline dehydrogenase and related flavoproteins E Amino acid transport and metabolism ; | Glucose-methanol-choline oxidoreductase, N-terminal [Penicillium italicum] |
| GL_Gan1_GLEAN_10005407 | NA | hypothetical protein PITC_035450 [Penicillium italicum] |
| GL_Gan1_GLEAN_10005414 | COG0404 Glycine cleavage system T protein (aminomethyltransferase) E Amino acid transport and metabolism ; | Glycine cleavage T-protein, N-terminal [Penicillium italicum] |
| GL_Gan1_GLEAN_10005418 | COG0146 N-methylhydantoinase B/acetone carboxylase, alpha subunit EQ Amino acid transport and metabolism ; Secondary metabolites biosynthesis, transport and catabolism ; | Hydantoinase B/oxoprolinase [Penicillium italicum] |
| GL_Gan1_GLEAN_10005419 | COG2273 Beta-glucanase/Beta-glucan synthetase G Carbohydrate transport and metabolism ; | Concanavalin A-like lectin/glucanase, subgroup [Penicillium italicum] |
| GL_Gan1_GLEAN_10005427 | COG0110 Acetyltransferase (isoleucine patch superfamily) R General function prediction only ; | Maltose/galactoside acetyltransferase [Penicillium expansum] |
| GL_Gan1_GLEAN_10005434 | COG0284 Orotidine-5'-phosphate decarboxylase F Nucleotide transport and metabolism ; | Aldolase-type TIM barrel [Penicillium italicum] |
| GL_Gan1_GLEAN_10005576 | COG0149 Triosephosphate isomerase G Carbohydrate transport and metabolism ; | Aldolase-type TIM barrel [Penicillium italicum] |
| GL_Gan1_GLEAN_10005601 | NA | hypothetical protein PITC_004320 [Penicillium italicum] |
| GL_Gan1_GLEAN_10005603 | COG2141 Coenzyme F420-dependent N5,N10-methylene tetrahydromethanopterin reductase and related flavin-dependent oxidoreductases C Energy production and conversion ; | Nitrilotriacetate monooxygenase component A/pristinamycin IIA synthase subunit A [Penicillium italicum] |
| GL_Gan1_GLEAN_10005628 | COG0246 Mannitol-1-phosphate/altronate dehydrogenases G Carbohydrate transport and metabolism ; | Mannitol dehydrogenase, C-terminal [Penicillium italicum] |
| GL_Gan1_GLEAN_10005639 | COG0044 Dihydroorotase and related cyclic amidohydrolases F Nucleotide transport and metabolism ; | Hydantoinase/dihydropyrimidinase [Penicillium italicum] |
| GL_Gan1_GLEAN_10005651 | COG0316 Uncharacterized conserved protein S Function unknown ; | FeS cluster insertion protein [Penicillium italicum] |
| GL_Gan1_GLEAN_10005074 | COG2272 Carboxylesterase type B I Lipid transport and metabolism ; | Carboxylesterase, type B [Penicillium italicum] |
| GL_Gan1_GLEAN_10005127 | NA | Alpha-L-fucosidase [Penicillium italicum] |
| GL_Gan1_GLEAN_10005157 | COG0596 Predicted hydrolases or acyltransferases (alpha/beta hydrolase superfamily) R General function prediction only ; | Alpha/beta hydrolase fold-1 [Penicillium italicum] |
| GL_Gan1_GLEAN_10005171 | COG2351 Transthyretin-like protein R General function prediction only ; | Hydroxyisourate hydrolase [Penicillium italicum] |
| GL_Gan1_GLEAN_10000842 | COG1816 Adenosine deaminase F Nucleotide transport and metabolism ; | Adenosine/adenine deaminase [Penicillium italicum] |
| GL_Gan1_GLEAN_10000849 | COG2303 Choline dehydrogenase and related flavoproteins E Amino acid transport and metabolism ; | Glucose-methanol-choline oxidoreductase [Penicillium italicum] |
| GL_Gan1_GLEAN_10003940 | COG1960 Acyl-CoA dehydrogenases I Lipid transport and metabolism ; | Acyl-CoA dehydrogenase, N-terminal [Penicillium italicum] |
| GL_Gan1_GLEAN_10003979 | COG1670 Acetyltransferases, including N-acetylases of ribosomal proteins J Translation, ribosomal structure and biogenesis ; | Acyl-CoA N-acyltransferase [Penicillium italicum] |
| GL_Gan1_GLEAN_10007042 | NA | Keratin-associated protein 4-3 [Cricetulus griseus] |
| GL_Gan1_GLEAN_10007060 | COG2312 Erythromycin esterase homolog R General function prediction only ; | Erythromycin esterase [Penicillium italicum] |
| GL_Gan1_GLEAN_10007061 | COG0129 Dihydroxyacid dehydratase/phosphogluconate dehydratase EG Amino acid transport and metabolism ; Carbohydrate transport and metabolism ; | Dihydroxy-acid/6-phosphogluconate dehydratase [Penicillium expansum] |
| GL_Gan1_GLEAN_10007076 | COG3938 Proline racemase E Amino acid transport and metabolism ; | Proline racemase [Penicillium italicum] |
| GL_Gan1_GLEAN_10007077 | NA | Esterase, SGNH hydrolase-type, subgroup [Penicillium italicum] |
| GL_Gan1_GLEAN_10007083 | COG2214 DnaJ-class molecular chaperone O Posttranslational modification, protein turnover, chaperones ; | Heat shock protein DnaJ, N-terminal [Penicillium italicum] |
| GL_Gan1_GLEAN_10007097 | COG1454 Alcohol dehydrogenase, class IV C Energy production and conversion ; | Alcohol dehydrogenase, iron-type [Penicillium roqueforti FM164] |
| GL_Gan1_GLEAN_10000071 | COG2072 Predicted flavoprotein involved in K+ transport P Inorganic ion transport and metabolism ; | hypothetical protein PITC_056190 [Penicillium italicum] |
| GL_Gan1_GLEAN_10000181 | COG3491 Isopenicillin N synthase and related dioxygenases R General function prediction only ; | Oxoglutarate/iron-dependent dioxygenase [Penicillium italicum] |
| GL_Gan1_GLEAN_10000183 | COG1231 Monoamine oxidase E Amino acid transport and metabolism ; | Flavin amine oxidase [Penicillium italicum] |
| GL_Gan1_GLEAN_10002027 | COG0520 Selenocysteine lyase E Amino acid transport and metabolism ; | Pyridoxal phosphate-dependent transferase, major region, subdomain 2 [Penicillium italicum] |
| GL_Gan1_GLEAN_10003240 | COG0666 FOG: Ankyrin repeat R General function prediction only ; | similar to ankyrin repeat domain-containing protein [Botrytis cinerea T4] |
| GL_Gan1_GLEAN_10003252 | COG0604 NADPH:quinone reductase and related Zn-dependent oxidoreductases CR Energy production and conversion ; General function prediction only ; | Polyketide synthase, enoylreductase [Penicillium italicum] |
| GL_Gan1_GLEAN_10003265 | NA | Glycoside hydrolase, family 43 [Penicillium italicum] |
| GL_Gan1_GLEAN_10001131 | COG3491 Isopenicillin N synthase and related dioxygenases R General function prediction only ; | Oxoglutarate/iron-dependent dioxygenase [Penicillium italicum] |
| GL_Gan1_GLEAN_10005199 | COG0656 Aldo/keto reductases, related to diketogulonate reductase R General function prediction only ; | Aldo/keto reductase subgroup [Penicillium italicum] |
| GL_Gan1_GLEAN_10005252 | COG2391 Predicted transporter component R General function prediction only ; | protein of unknown function DUF4341 [Penicillium italicum] |
| GL_Gan1_GLEAN_10005267 | COG0823 Periplasmic component of the Tol biopolymer transport system U Intracellular trafficking, secretion, and vesicular transport ; | WD40-like Beta Propeller [Penicillium italicum] |
| GL_Gan1_GLEAN_10002134 | COG2828 Uncharacterized protein conserved in bacteria S Function unknown ; | PrpF protein [Penicillium italicum] |
| GL_Gan1_GLEAN_10002143 | COG1816 Adenosine deaminase F Nucleotide transport and metabolism ; | Adenosine/adenine deaminase [Penicillium italicum] |
| GL_Gan1_GLEAN_10002172 | COG0657 Esterase/lipase I Lipid transport and metabolism ; | Alpha/beta hydrolase fold-3 [Penicillium italicum] |
| GL_Gan1_GLEAN_10000001 | COG3039 Transposase and inactivated derivatives, IS5 family L Replication, recombination and repair ; | transposase [Pseudomonas stutzeri] |
| GL_Gan1_GLEAN_10004562 | NA | Concanavalin A-like lectin/glucanase, subgroup [Penicillium italicum] |
| GL_Gan1_GLEAN_10004596 | COG0491 Zn-dependent hydrolases, including glyoxylases R General function prediction only ; | hypothetical protein PITC_010830 [Penicillium italicum] |
| GL_Gan1_GLEAN_10004607 | COG3534 Alpha-L-arabinofuranosidase G Carbohydrate transport and metabolism ; | Glycoside hydrolase, superfamily [Penicillium italicum] |
| GL_Gan1_GLEAN_10004610 | COG2141 Coenzyme F420-dependent N5,N10-methylene tetrahydromethanopterin reductase and related flavin-dependent oxidoreductases C Energy production and conversion ; | hypothetical protein PITC_021760 [Penicillium italicum] |
| GL_Gan1_GLEAN_10004626 | COG0366 Glycosidases G Carbohydrate transport and metabolism ; | Glycoside hydrolase, superfamily [Penicillium italicum] |
| GL_Gan1_GLEAN_10004632 | COG1020 Non-ribosomal peptide synthetase modules and related proteins Q Secondary metabolites biosynthesis, transport and catabolism ; | Male sterility, NAD-binding [Penicillium italicum] |
| GL_Gan1_GLEAN_10003464 | NA | Protein of unknown function DUF1682 [Penicillium italicum] |
| GL_Gan1_GLEAN_10003466 | COG3560 Predicted oxidoreductase related to nitroreductase R General function prediction only ; | hypothetical protein PITC_044890 [Penicillium italicum] |
| GL_Gan1_GLEAN_10003467 | COG1028 Dehydrogenases with different specificities (related to short-chain alcohol dehydrogenases) IQR Lipid transport and metabolism ; Secondary metabolites biosynthesis, transport and catabolism ; General function prediction only ; | Short-chain dehydrogenase/reductase SDR [Penicillium italicum] |
| GL_Gan1_GLEAN_10003471 | COG1051 ADP-ribose pyrophosphatase F Nucleotide transport and metabolism ; | NUDIX hydrolase [Penicillium italicum] |
| GL_Gan1_GLEAN_10003473 | COG0328 Ribonuclease HI L Replication, recombination and repair ; | hypothetical protein PITC_044830 [Penicillium italicum] |
| GL_Gan1_GLEAN_10003517 | COG0494 NTP pyrophosphohydrolases including oxidative damage repair enzymes LR Replication, recombination and repair ; General function prediction only ; | hypothetical protein PITC_080370 [Penicillium italicum] |
| GL_Gan1_GLEAN_10003523 | COG0129 Dihydroxyacid dehydratase/phosphogluconate dehydratase EG Amino acid transport and metabolism ; Carbohydrate transport and metabolism ; | Dihydroxy-acid/6-phosphogluconate dehydratase [Penicillium italicum] |
| GL_Gan1_GLEAN_10000161 | COG1228 Imidazolonepropionase and related amidohydrolases Q Secondary metabolites biosynthesis, transport and catabolism ; | Amidohydrolase 1 [Penicillium italicum] |
| GL_Gan1_GLEAN_10002235 | COG0604 NADPH:quinone reductase and related Zn-dependent oxidoreductases CR Energy production and conversion ; General function prediction only ; | Polyketide synthase, enoylreductase [Penicillium italicum] |
| GL_Gan1_GLEAN_10002241 | COG1004 Predicted UDP-glucose 6-dehydrogenase M Cell wall/membrane/envelope biogenesis ; | Cytochrome c1, transmembrane anchor, C-terminal [Penicillium italicum] |
| GL_Gan1_GLEAN_10002261 | COG0724 RNA-binding proteins (RRM domain) R General function prediction only ; | Nucleotide-binding, alpha-beta plait [Penicillium italicum] |
| GL_Gan1_GLEAN_10002284 | COG1610 Uncharacterized conserved protein S Function unknown ; | Pc20g01310 [Penicillium rubens Wisconsin 54-1255] |
| GL_Gan1_GLEAN_10007665 | COG0509 Glycine cleavage system H protein (lipoate-binding) E Amino acid transport and metabolism ; | Glycine cleavage H-protein, subgroup [Penicillium italicum] |
| GL_Gan1_GLEAN_10007701 | COG1018 Flavodoxin reductases (ferredoxin-NADPH reductases) family 1 C Energy production and conversion ; | Globin [Penicillium italicum] |
| GL_Gan1_GLEAN_10007712 | COG0673 Predicted dehydrogenases and related proteins R General function prediction only ; | Oxidoreductase, N-terminal [Penicillium italicum] |
| GL_Gan1_GLEAN_10007714 | COG0500 SAM-dependent methyltransferases QR Secondary metabolites biosynthesis, transport and catabolism ; General function prediction only ; | hypothetical protein PITC_047870 [Penicillium italicum] |
| GL_Gan1_GLEAN_10007718 | COG0833 Amino acid transporters E Amino acid transport and metabolism ; | Amino acid/polyamine transporter I [Penicillium expansum] |
| GL_Gan1_GLEAN_10007730 | COG3145 Alkylated DNA repair protein L Replication, recombination and repair ; | Oxoglutarate/iron-dependent dioxygenase [Penicillium italicum] |
| GL_Gan1_GLEAN_10007733 | COG0591 Na+/proline symporter ER Amino acid transport and metabolism ; General function prediction only ; | Sodium/solute symporter [Penicillium expansum] |
| GL_Gan1_GLEAN_10007752 | COG0454 Histone acetyltransferase HPA2 and related acetyltransferases KR Transcription ; General function prediction only ; | Acyl-CoA N-acyltransferase [Penicillium italicum] |
| GL_Gan1_GLEAN_10007759 | COG0654 2-polyprenyl-6-methoxyphenol hydroxylase and related FAD-dependent oxidoreductases HC Coenzyme transport and metabolism ; Energy production and conversion ; | Monooxygenase, FAD-binding [Penicillium expansum] |
| GL_Gan1_GLEAN_10007760 | COG0376 Catalase (peroxidase I) P Inorganic ion transport and metabolism ; | Catalase-peroxidase heme [Penicillium italicum] |
| GL_Gan1_GLEAN_10007763 | COG0345 Pyrroline-5-carboxylate reductase E Amino acid transport and metabolism ; | Pyrroline-5-carboxylate reductase [Penicillium digitatum Pd1] |
| GL_Gan1_GLEAN_10007769 | COG4225 Predicted unsaturated glucuronyl hydrolase involved in regulation of bacterial surface properties, and related proteins R General function prediction only ; | Six-hairpin glycosidase [Penicillium italicum] |
| GL_Gan1_GLEAN_10006018 | NA | hypothetical protein PITC_008720 [Penicillium italicum] |
| GL_Gan1_GLEAN_10006049 | COG0596 Predicted hydrolases or acyltransferases (alpha/beta hydrolase superfamily) R General function prediction only ; | hypothetical protein PITC_055380 [Penicillium italicum] |
| GL_Gan1_GLEAN_10006095 | COG0125 Thymidylate kinase F Nucleotide transport and metabolism ; | Thymidylate kinase [Penicillium expansum] |
| GL_Gan1_GLEAN_10006125 | COG3957 Phosphoketolase G Carbohydrate transport and metabolism ; | Transketolase, C-terminal/Pyruvate-ferredoxin oxidoreductase, domain II [Penicillium italicum] |
| GL_Gan1_GLEAN_10006136 | COG0654 2-polyprenyl-6-methoxyphenol hydroxylase and related FAD-dependent oxidoreductases HC Coenzyme transport and metabolism ; Energy production and conversion ; | Monooxygenase, FAD-binding [Penicillium italicum] |
| GL_Gan1_GLEAN_10006142 | COG4122 Predicted O-methyltransferase R General function prediction only ; | hypothetical protein GLRG_11986 [Colletotrichum graminicola M1.001] |
| GL_Gan1_GLEAN_10000898 | COG3491 Isopenicillin N synthase and related dioxygenases R General function prediction only ; | Oxoglutarate/iron-dependent dioxygenase [Penicillium italicum] |
| GL_Gan1_GLEAN_10002588 | COG3693 Beta-1,4-xylanase G Carbohydrate transport and metabolism ; | Glycoside hydrolase, superfamily [Penicillium italicum] |
| GL_Gan1_GLEAN_10005462 | COG3616 Predicted amino acid aldolase or racemase E Amino acid transport and metabolism ; | Alanine racemase, N-terminal [Penicillium italicum] |
| GL_Gan1_GLEAN_10005521 | COG0119 Isopropylmalate/homocitrate/citramalate synthases E Amino acid transport and metabolism ; | Pyruvate carboxyltransferase [Penicillium italicum] |
| GL_Gan1_GLEAN_10005547 | COG0489 ATPases involved in chromosome partitioning D Cell cycle control, cell division, chromosome partitioning ; | ATPase-like, ParA/MinD [Penicillium italicum] |
| GL_Gan1_GLEAN_10005564 | COG0111 Phosphoglycerate dehydrogenase and related dehydrogenases HE Coenzyme transport and metabolism ; Amino acid transport and metabolism ; | Acyl-CoA N-acyltransferase [Penicillium italicum] |
| GL_Gan1_GLEAN_10005568 | COG0642 Signal transduction histidine kinase T Signal transduction mechanisms ; | CheY-like superfamily [Penicillium italicum] |
| GL_Gan1_GLEAN_10005787 | COG1028 Dehydrogenases with different specificities (related to short-chain alcohol dehydrogenases) IQR Lipid transport and metabolism ; Secondary metabolites biosynthesis, transport and catabolism ; General function prediction only ; | Short-chain dehydrogenase/reductase SDR [Penicillium italicum] |
| GL_Gan1_GLEAN_10005806 | COG3119 Arylsulfatase A and related enzymes P Inorganic ion transport and metabolism ; | Alkaline phosphatase-like, alpha/beta/alpha [Penicillium italicum] |
| GL_Gan1_GLEAN_10005821 | COG1670 Acetyltransferases, including N-acetylases of ribosomal proteins J Translation, ribosomal structure and biogenesis ; | Acyl-CoA N-acyltransferase [Penicillium italicum] |
| GL_Gan1_GLEAN_10005842 | COG2130 Putative NADP-dependent oxidoreductases R General function prediction only ; | Alcohol dehydrogenase, C-terminal [Penicillium italicum] |
| GL_Gan1_GLEAN_10003550 | COG1028 Dehydrogenases with different specificities (related to short-chain alcohol dehydrogenases) IQR Lipid transport and metabolism ; Secondary metabolites biosynthesis, transport and catabolism ; General function prediction only ; | Short-chain dehydrogenase/reductase SDR [Penicillium italicum] |
| GL_Gan1_GLEAN_10003576 | COG2110 Predicted phosphatase homologous to the C-terminal domain of histone macroH2A1 R General function prediction only ; | Appr-1-p processing [Penicillium italicum] |
| GL_Gan1_GLEAN_10003591 | COG0693 Putative intracellular protease/amidase R General function prediction only ; | ThiJ/PfpI [Penicillium italicum] |
| GL_Gan1_GLEAN_10003594 | COG0666 FOG: Ankyrin repeat R General function prediction only ; | hypothetical protein PITC_039420 [Penicillium italicum] |
| GL_Gan1_GLEAN_10003623 | COG1171 Threonine dehydratase E Amino acid transport and metabolism ; | Tryptophan synthase beta subunit-like PLP-dependent enzymes superfamily [Penicillium italicum] |
| GL_Gan1_GLEAN_10000339 | COG3250 Beta-galactosidase/beta-glucuronidase G Carbohydrate transport and metabolism ; | Glycoside hydrolase, family 2, N-terminal [Penicillium italicum] |
| GL_Gan1_GLEAN_10000483 | COG0625 Glutathione S-transferase O Posttranslational modification, protein turnover, chaperones ; | Glutathione S-transferase/chloride channel, C-terminal [Penicillium italicum] |
| GL_Gan1_GLEAN_10000420 | COG4948 L-alanine-DL-glutamate epimerase and related enzymes of enolase superfamily MR Cell wall/membrane/envelope biogenesis ; General function prediction only ; | Mandelate racemase/muconate lactonizing enzyme, C-terminal [Penicillium italicum] |
| GL_Gan1_GLEAN_10006492 | COG2319 FOG: WD40 repeat R General function prediction only ; | G-protein, beta subunit [Penicillium italicum] |
| GL_Gan1_GLEAN_10006566 | COG1501 Alpha-glucosidases, family 31 of glycosyl hydrolases G Carbohydrate transport and metabolism ; | Glycoside hydrolase, family 31 [Penicillium italicum] |
| GL_Gan1_GLEAN_10006575 | COG0246 Mannitol-1-phosphate/altronate dehydrogenases G Carbohydrate transport and metabolism ; | Mannitol dehydrogenase, C-terminal [Penicillium italicum] |
| GL_Gan1_GLEAN_10006585 | COG1250 3-hydroxyacyl-CoA dehydrogenase I Lipid transport and metabolism ; | Dehydrogenase, multihelical [Penicillium italicum] |
| GL_Gan1_GLEAN_10006617 | COG2303 Choline dehydrogenase and related flavoproteins E Amino acid transport and metabolism ; | Glucose-methanol-choline oxidoreductase [Penicillium italicum] |
| GL_Gan1_GLEAN_10006618 | COG1012 NAD-dependent aldehyde dehydrogenases C Energy production and conversion ; | Aldehyde dehydrogenase, N-terminal [Penicillium italicum] |
| GL_Gan1_GLEAN_10001125 | COG4948 L-alanine-DL-glutamate epimerase and related enzymes of enolase superfamily MR Cell wall/membrane/envelope biogenesis ; General function prediction only ; | Mandelate racemase/muconate lactonizing enzyme [Penicillium italicum] |
| GL_Gan1_GLEAN_10001002 | COG0451 Nucleoside-diphosphate-sugar epimerases MG Cell wall/membrane/envelope biogenesis ; Carbohydrate transport and metabolism ; | NAD-dependent epimerase/dehydratase [Penicillium italicum] |

(**C**) Effectors

| Gene_ID | amid acid length(aa) | Cys number | Cys (%) |
| --- | --- | --- | --- |
| GL_Gan1_GLEAN_10005734 | 367 | 4 | 1.09% |
| GL_Gan1_GLEAN_10003032 | 581 | 10 | 1.72% |
| GL_Gan1_GLEAN_10003924 | 188 | 0 | 0% |
| GL_Gan1_GLEAN_10006506 | 601 | 7 | 1.16% |
| GL_Gan1_GLEAN_10006319 | 514 | 7 | 1.36% |
| GL_Gan1_GLEAN_10001756 | 202 | 4 | 1.98% |
| GL_Gan1_GLEAN_10006884 | 491 | 6 | 1.22% |
| GL_Gan1_GLEAN_10008812 | 573 | 2 | 0.35% |
| GL_Gan1_GLEAN_10007096 | 680 | 4 | 0.59% |
| GL_Gan1_GLEAN_10004316 | 291 | 6 | 2.06% |
| GL_Gan1_GLEAN_10001661 | 171 | 9 | 5.26% |
| GL_Gan1_GLEAN_10008861 | 207 | 8 | 3.86% |
| GL_Gan1_GLEAN_10003868 | 482 | 1 | 0.21% |
| GL_Gan1_GLEAN_10000362 | 732 | 9 | 1.23% |
| GL_Gan1_GLEAN_10006737 | 836 | 1 | 0.12% |
| GL_Gan1_GLEAN_10008868 | 338 | 2 | 0.59% |
| GL_Gan1_GLEAN_10008115 | 610 | 5 | 0.82% |
| GL_Gan1_GLEAN_10000943 | 820 | 6 | 0.73% |
| GL_Gan1_GLEAN_10004771 | 103 | 0 | 0% |
| GL_Gan1_GLEAN_10005870 | 792 | 10 | 1.26% |
| GL_Gan1_GLEAN_10004590 | 270 | 12 | 4.44% |
| GL_Gan1_GLEAN_10000998 | 1008 | 6 | 0.60% |
| GL_Gan1_GLEAN_10003283 | 372 | 2 | 0.54% |
| GL_Gan1_GLEAN_10001327 | 871 | 13 | 1.49% |
| GL_Gan1_GLEAN_10003888 | 313 | 1 | 0.32% |
| GL_Gan1_GLEAN_10006841 | 616 | 16 | 2.60% |
| GL_Gan1_GLEAN_10002167 | 146 | 8 | 5.48% |
| GL_Gan1_GLEAN_10006918 | 415 | 6 | 1.45% |
| GL_Gan1_GLEAN_10002846 | 1074 | 7 | 0.65% |
| GL_Gan1_GLEAN_10007781 | 95 | 6 | 6.32% |
| GL_Gan1_GLEAN_10007704 | 166 | 0 | 0% |
| GL_Gan1_GLEAN_10008751 | 688 | 7 | 1.02% |
| GL_Gan1_GLEAN_10009245 | 195 | 6 | 3.08% |
| GL_Gan1_GLEAN_10005158 | 645 | 8 | 1.24% |
| GL_Gan1_GLEAN_10005347 | 331 | 6 | 1.81% |
| GL_Gan1_GLEAN_10007658 | 279 | 6 | 2.15% |
| GL_Gan1_GLEAN_10003245 | 355 | 1 | 0.28% |
| GL_Gan1_GLEAN_10008673 | 536 | 2 | 0.37% |
| GL_Gan1_GLEAN_10007230 | 269 | 1 | 0.37% |
| GL_Gan1_GLEAN_10007245 | 720 | 13 | 1.81% |
| GL_Gan1_GLEAN_10006582 | 497 | 8 | 1.61% |
| GL_Gan1_GLEAN_10005249 | 204 | 2 | 0.98% |
| GL_Gan1_GLEAN_10006586 | 410 | 4 | 0.98% |
| GL_Gan1_GLEAN_10007974 | 142 | 1 | 0.70% |
| GL_Gan1_GLEAN_10001506 | 169 | 6 | 3.55% |
| GL_Gan1_GLEAN_10001304 | 293 | 0 | 0% |
| GL_Gan1_GLEAN_10005541 | 660 | 5 | 0.76% |
| GL_Gan1_GLEAN_10000636 | 176 | 2 | 1.14% |
| GL_Gan1_GLEAN_10008288 | 181 | 8 | 4.42% |
| GL_Gan1_GLEAN_10002847 | 731 | 1 | 0.14% |
| GL_Gan1_GLEAN_10006232 | 362 | 2 | 0.55% |
| GL_Gan1_GLEAN_10006190 | 364 | 4 | 1.10% |
| GL_Gan1_GLEAN_10008482 | 512 | 3 | 0.59% |
| GL_Gan1_GLEAN_10003121 | 87 | 6 | 6.90% |
| GL_Gan1_GLEAN_10004540 | 461 | 1 | 0.22% |
| GL_Gan1_GLEAN_10004061 | 202 | 6 | 2.97% |
| GL_Gan1_GLEAN_10005673 | 108 | 0 | 0% |
| GL_Gan1_GLEAN_10007808 | 444 | 7 | 1.58% |
| GL_Gan1_GLEAN_10005931 | 201 | 1 | 0.50% |
| GL_Gan1_GLEAN_10008012 | 1130 | 41 | 3.63% |
| GL_Gan1_GLEAN_10001856 | 132 | 1 | 0.76% |
| GL_Gan1_GLEAN_10008849 | 633 | 8 | 1.26% |
| GL_Gan1_GLEAN_10008328 | 237 | 2 | 0.84% |
| GL_Gan1_GLEAN_10004842 | 567 | 4 | 0.71% |
| GL_Gan1_GLEAN_10000458 | 517 | 28 | 5.42% |
| GL_Gan1_GLEAN_10001832 | 401 | 3 | 0.75% |
| GL_Gan1_GLEAN_10000964 | 515 | 6 | 1.17% |
| GL_Gan1_GLEAN_10000551 | 401 | 5 | 1.25% |
| GL_Gan1_GLEAN_10006429 | 768 | 11 | 1.43% |
| GL_Gan1_GLEAN_10008974 | 594 | 7 | 1.18% |
| GL_Gan1_GLEAN_10005253 | 399 | 2 | 0.50% |
| GL_Gan1_GLEAN_10005543 | 698 | 6 | 0.86% |
| GL_Gan1_GLEAN_10004261 | 373 | 11 | 2.95% |
| GL_Gan1_GLEAN_10002138 | 311 | 5 | 1.61% |
| GL_Gan1_GLEAN_10006686 | 483 | 9 | 1.86% |
| GL_Gan1_GLEAN_10001600 | 586 | 3 | 0.51% |
| GL_Gan1_GLEAN_10004301 | 591 | 3 | 0.51% |
| GL_Gan1_GLEAN_10002439 | 455 | 8 | 1.76% |
| GL_Gan1_GLEAN_10006054 | 670 | 5 | 0.75% |
| GL_Gan1_GLEAN_10005007 | 214 | 5 | 2.34% |
| GL_Gan1_GLEAN_10005195 | 68 | 1 | 1.47% |
| GL_Gan1_GLEAN_10006787 | 358 | 4 | 1.12% |
| GL_Gan1_GLEAN_10003533 | 259 | 0 | 0% |
| GL_Gan1_GLEAN_10004237 | 183 | 10 | 5.46% |
| GL_Gan1_GLEAN_10008841 | 491 | 4 | 0.81% |
| GL_Gan1_GLEAN_10001123 | 247 | 0 | 0% |
| GL_Gan1_GLEAN_10004419 | 140 | 8 | 5.71% |
| GL_Gan1_GLEAN_10006743 | 547 | 8 | 1.46% |
| GL_Gan1_GLEAN_10009053 | 544 | 3 | 0.55% |
| GL_Gan1_GLEAN_10006713 | 446 | 10 | 2.24% |
| GL_Gan1_GLEAN_10008278 | 73 | 1 | 1.37% |
| GL_Gan1_GLEAN_10003597 | 325 | 4 | 1.23% |
| GL_Gan1_GLEAN_10009000 | 366 | 2 | 0.55% |
| GL_Gan1_GLEAN_10006263 | 296 | 4 | 1.35% |
| GL_Gan1_GLEAN_10001191 | 811 | 3 | 0.37% |
| GL_Gan1_GLEAN_10003334 | 519 | 13 | 2.50% |
| GL_Gan1_GLEAN_10009049 | 180 | 5 | 2.78% |
| GL_Gan1_GLEAN_10003229 | 264 | 5 | 1.89% |
| GL_Gan1_GLEAN_10001149 | 518 | 10 | 1.93% |
| GL_Gan1_GLEAN_10001774 | 687 | 5 | 0.73% |
| GL_Gan1_GLEAN_10004244 | 399 | 4 | 1.00% |
| GL_Gan1_GLEAN_10001179 | 392 | 9 | 2.30% |
| GL_Gan1_GLEAN_10002671 | 187 | 13 | 6.95% |
| GL_Gan1_GLEAN_10003314 | 361 | 0 | 0% |
| GL_Gan1_GLEAN_10006505 | 195 | 2 | 1.03% |
| GL_Gan1_GLEAN_10007659 | 256 | 12 | 4.69% |
| GL_Gan1_GLEAN_10006486 | 895 | 17 | 1.90% |
| GL_Gan1_GLEAN_10000397 | 289 | 7 | 2.42% |
| GL_Gan1_GLEAN_10006574 | 544 | 12 | 2.21% |
| GL_Gan1_GLEAN_10007845 | 202 | 3 | 1.49% |
| GL_Gan1_GLEAN_10004479 | 758 | 5 | 0.66% |
| GL_Gan1_GLEAN_10001649 | 200 | 10 | 5% |
| GL_Gan1_GLEAN_10008855 | 117 | 8 | 6.84% |
| GL_Gan1_GLEAN_10006973 | 521 | 7 | 1.34% |
| GL_Gan1_GLEAN_10001345 | 266 | 5 | 1.88% |
| GL_Gan1_GLEAN_10006770 | 131 | 2 | 1.53% |
| GL_Gan1_GLEAN_10002000 | 129 | 6 | 4.65% |
| GL_Gan1_GLEAN_10005311 | 196 | 3 | 1.53% |
| GL_Gan1_GLEAN_10004627 | 272 | 0 | 0% |
| GL_Gan1_GLEAN_10008291 | 257 | 10 | 3.89% |
| GL_Gan1_GLEAN_10001851 | 865 | 6 | 0.69% |
| GL_Gan1_GLEAN_10001829 | 499 | 7 | 1.40% |
| GL_Gan1_GLEAN_10001750 | 275 | 8 | 2.91% |
| GL_Gan1_GLEAN_10000592 | 194 | 4 | 2.06% |
| GL_Gan1_GLEAN_10004823 | 529 | 14 | 2.65% |
| GL_Gan1_GLEAN_10002215 | 87 | 5 | 5.75% |
| GL_Gan1_GLEAN_10003669 | 567 | 17 | 3.00% |
| GL_Gan1_GLEAN_10001103 | 489 | 3 | 0.61% |
| GL_Gan1_GLEAN_10007786 | 895 | 4 | 0.45% |
| GL_Gan1_GLEAN_10006856 | 564 | 6 | 1.06% |
| GL_Gan1_GLEAN_10004477 | 454 | 0 | 0% |
| GL_Gan1_GLEAN_10004311 | 333 | 4 | 1.20% |
| GL_Gan1_GLEAN_10002568 | 538 | 5 | 0.93% |
| GL_Gan1_GLEAN_10000274 | 512 | 6 | 1.17% |
| GL_Gan1_GLEAN_10009051 | 294 | 29 | 9.86% |
| GL_Gan1_GLEAN_10009379 | 494 | 5 | 1.01% |
| GL_Gan1_GLEAN_10000830 | 463 | 13 | 2.81% |
| GL_Gan1_GLEAN_10007639 | 336 | 12 | 3.57% |
| GL_Gan1_GLEAN_10005122 | 470 | 7 | 1.49% |
| GL_Gan1_GLEAN_10002124 | 512 | 1 | 0.20% |
| GL_Gan1_GLEAN_10004442 | 635 | 2 | 0.31% |
| GL_Gan1_GLEAN_10003448 | 200 | 4 | 2% |
| GL_Gan1_GLEAN_10007495 | 190 | 4 | 2.11% |
| GL_Gan1_GLEAN_10002588 | 400 | 9 | 2.25% |
| GL_Gan1_GLEAN_10005410 | 344 | 2 | 0.58% |
| GL_Gan1_GLEAN_10000535 | 706 | 4 | 0.57% |
| GL_Gan1_GLEAN_10002513 | 733 | 12 | 1.64% |
| GL_Gan1_GLEAN_10002459 | 525 | 8 | 1.52% |
| GL_Gan1_GLEAN_10008591 | 856 | 1 | 0.12% |
| GL_Gan1_GLEAN_10008863 | 571 | 3 | 0.53% |
| GL_Gan1_GLEAN_10001595 | 121 | 6 | 4.96% |
| GL_Gan1_GLEAN_10002301 | 667 | 8 | 1.20% |
| GL_Gan1_GLEAN_10004982 | 67 | 1 | 1.49% |
| GL_Gan1_GLEAN_10000576 | 554 | 11 | 1.99% |
| GL_Gan1_GLEAN_10005455 | 225 | 8 | 3.56% |
| GL_Gan1_GLEAN_10004808 | 287 | 2 | 0.70% |
| GL_Gan1_GLEAN_10005352 | 708 | 0 | 0% |
| GL_Gan1_GLEAN_10002949 | 387 | 0 | 0% |
| GL_Gan1_GLEAN_10006964 | 669 | 10 | 1.49% |
| GL_Gan1_GLEAN_10004164 | 279 | 0 | 0% |
| GL_Gan1_GLEAN_10007864 | 561 | 9 | 1.60% |
| GL_Gan1_GLEAN_10007364 | 584 | 5 | 0.86% |
| GL_Gan1_GLEAN_10004602 | 391 | 8 | 2.05% |
| GL_Gan1_GLEAN_10006834 | 314 | 3 | 0.96% |
| GL_Gan1_GLEAN_10005345 | 372 | 8 | 2.15% |
| GL_Gan1_GLEAN_10009022 | 373 | 6 | 1.61% |
| GL_Gan1_GLEAN_10003426 | 344 | 14 | 4.07% |
| GL_Gan1_GLEAN_10007452 | 435 | 11 | 2.53% |
| GL_Gan1_GLEAN_10008559 | 252 | 2 | 0.79% |
| GL_Gan1_GLEAN_10003476 | 378 | 6 | 1.59% |
| GL_Gan1_GLEAN_10002901 | 666 | 5 | 0.75% |
| GL_Gan1_GLEAN_10006844 | 301 | 6 | 1.99% |
| GL_Gan1_GLEAN_10000206 | 176 | 4 | 2.27% |
| GL_Gan1_GLEAN_10008076 | 137 | 2 | 1.46% |
| GL_Gan1_GLEAN_10006861 | 399 | 5 | 1.25% |
| GL_Gan1_GLEAN_10006076 | 531 | 7 | 1.32% |
| GL_Gan1_GLEAN_10008986 | 489 | 7 | 1.43% |
| GL_Gan1_GLEAN_10003327 | 214 | 2 | 0.93% |
| GL_Gan1_GLEAN_10004365 | 476 | 2 | 0.42% |
| GL_Gan1_GLEAN_10005074 | 614 | 10 | 1.63% |
| GL_Gan1_GLEAN_10008113 | 123 | 6 | 4.88% |
| GL_Gan1_GLEAN_10001178 | 545 | 3 | 0.55% |
| GL_Gan1_GLEAN_10003742 | 501 | 10 | 2.00% |
| GL_Gan1_GLEAN_10008068 | 781 | 3 | 0.38% |
| GL_Gan1_GLEAN_10004856 | 131 | 5 | 3.82% |
| GL_Gan1_GLEAN_10003197 | 483 | 7 | 1.45% |
| GL_Gan1_GLEAN_10005963 | 270 | 4 | 1.48% |
| GL_Gan1_GLEAN_10002565 | 264 | 8 | 3.03% |
| GL_Gan1_GLEAN_10002628 | 255 | 14 | 5.49% |
| GL_Gan1_GLEAN_10007353 | 379 | 7 | 1.85% |
| GL_Gan1_GLEAN_10004739 | 155 | 5 | 3.23% |
| GL_Gan1_GLEAN_10002834 | 407 | 3 | 0.74% |
| GL_Gan1_GLEAN_10009234 | 151 | 6 | 3.97% |
| GL_Gan1_GLEAN_10000645 | 644 | 8 | 1.24% |
| GL_Gan1_GLEAN_10008145 | 690 | 3 | 0.43% |
| GL_Gan1_GLEAN_10005900 | 329 | 19 | 5.78% |
| GL_Gan1_GLEAN_10000952 | 329 | 6 | 1.82% |
| GL_Gan1_GLEAN_10008747 | 367 | 8 | 2.18% |
| GL_Gan1_GLEAN_10005421 | 933 | 12 | 1.29% |
| GL_Gan1_GLEAN_10007268 | 534 | 5 | 0.94% |
| GL_Gan1_GLEAN_10006306 | 453 | 12 | 2.65% |
| GL_Gan1_GLEAN_10004956 | 458 | 21 | 4.59% |
| GL_Gan1_GLEAN_10002983 | 453 | 5 | 1.10% |
| GL_Gan1_GLEAN_10002283 | 226 | 3 | 1.33% |
| GL_Gan1_GLEAN_10001375 | 300 | 10 | 3.33% |
| GL_Gan1_GLEAN_10005997 | 357 | 5 | 1.40% |
| GL_Gan1_GLEAN_10001971 | 94 | 0 | 0% |
| GL_Gan1_GLEAN_10007657 | 1522 | 44 | 2.89% |
| GL_Gan1_GLEAN_10007087 | 351 | 18 | 5.13% |
| GL_Gan1_GLEAN_10006385 | 69 | 0 | 0% |
| GL_Gan1_GLEAN_10009267 | 265 | 3 | 1.13% |
| GL_Gan1_GLEAN_10008854 | 121 | 8 | 6.61% |
| GL_Gan1_GLEAN_10004841 | 659 | 10 | 1.52% |
| GL_Gan1_GLEAN_10004562 | 506 | 9 | 1.78% |
| GL_Gan1_GLEAN_10006274 | 452 | 7 | 1.55% |
| GL_Gan1_GLEAN_10007067 | 266 | 3 | 1.13% |
| GL_Gan1_GLEAN_10000630 | 147 | 4 | 2.72% |
| GL_Gan1_GLEAN_10007481 | 1138 | 13 | 1.14% |
| GL_Gan1_GLEAN_10006075 | 169 | 8 | 4.73% |
| GL_Gan1_GLEAN_10005081 | 203 | 2 | 0.99% |
| GL_Gan1_GLEAN_10005342 | 486 | 6 | 1.23% |
| GL_Gan1_GLEAN_10001462 | 1068 | 42 | 3.93% |
| GL_Gan1_GLEAN_10008711 | 462 | 4 | 0.87% |
| GL_Gan1_GLEAN_10003839 | 456 | 4 | 0.88% |
| GL_Gan1_GLEAN_10003037 | 202 | 0 | 0% |
| GL_Gan1_GLEAN_10006641 | 363 | 3 | 0.83% |
| GL_Gan1_GLEAN_10003719 | 396 | 3 | 0.76% |
| GL_Gan1_GLEAN_10008173 | 171 | 2 | 1.17% |
| GL_Gan1_GLEAN_10008927 | 223 | 4 | 1.79% |
| GL_Gan1_GLEAN_10006909 | 533 | 8 | 1.50% |
| GL_Gan1_GLEAN_10005403 | 339 | 4 | 1.18% |
| GL_Gan1_GLEAN_10007362 | 352 | 2 | 0.57% |
| GL_Gan1_GLEAN_10009094 | 263 | 6 | 2.28% |
| GL_Gan1_GLEAN_10007129 | 471 | 2 | 0.42% |
| GL_Gan1_GLEAN_10006002 | 209 | 1 | 0.48% |
| GL_Gan1_GLEAN_10001733 | 870 | 8 | 0.92% |
| GL_Gan1_GLEAN_10006271 | 1167 | 28 | 2.40% |
| GL_Gan1_GLEAN_10004737 | 815 | 4 | 0.49% |
| GL_Gan1_GLEAN_10008513 | 291 | 6 | 2.06% |
| GL_Gan1_GLEAN_10004037 | 509 | 5 | 0.98% |
| GL_Gan1_GLEAN_10002110 | 336 | 2 | 0.60% |
| GL_Gan1_GLEAN_10002497 | 497 | 6 | 1.21% |
| GL_Gan1_GLEAN_10000305 | 440 | 10 | 2.27% |
| GL_Gan1_GLEAN_10006541 | 242 | 6 | 2.48% |
| GL_Gan1_GLEAN_10008143 | 1082 | 12 | 1.11% |
| GL_Gan1_GLEAN_10007771 | 348 | 8 | 2.30% |
| GL_Gan1_GLEAN_10004702 | 245 | 0 | 0% |
| GL_Gan1_GLEAN_10002598 | 241 | 10 | 4.15% |
| GL_Gan1_GLEAN_10008168 | 222 | 0 | 0% |
| GL_Gan1_GLEAN_10004524 | 216 | 2 | 0.93% |
| GL_Gan1_GLEAN_10002696 | 307 | 6 | 1.95% |
| GL_Gan1_GLEAN_10002224 | 163 | 3 | 1.84% |
| GL_Gan1_GLEAN_10004110 | 658 | 6 | 0.91% |
| GL_Gan1_GLEAN_10003465 | 701 | 9 | 1.28% |
| GL_Gan1_GLEAN_10002698 | 247 | 4 | 1.62% |
| GL_Gan1_GLEAN_10004277 | 114 | 4 | 3.51% |
| GL_Gan1_GLEAN_10004364 | 907 | 7 | 0.77% |
| GL_Gan1_GLEAN_10002720 | 604 | 3 | 0.50% |
| GL_Gan1_GLEAN_10001841 | 216 | 3 | 1.39% |
| GL_Gan1_GLEAN_10005669 | 368 | 8 | 2.17% |
| GL_Gan1_GLEAN_10001796 | 243 | 0 | 0% |
| GL_Gan1_GLEAN_10005201 | 713 | 7 | 0.98% |
| GL_Gan1_GLEAN_10008420 | 97 | 8 | 8.25% |
| GL_Gan1_GLEAN_10007985 | 116 | 2 | 1.72% |
| GL_Gan1_GLEAN_10003660 | 233 | 2 | 0.86% |
| GL_Gan1_GLEAN_10005267 | 677 | 4 | 0.59% |
| GL_Gan1_GLEAN_10000933 | 289 | 7 | 2.42% |
| GL_Gan1_GLEAN_10008779 | 152 | 1 | 0.66% |
| GL_Gan1_GLEAN_10000124 | 514 | 6 | 1.17% |
| GL_Gan1_GLEAN_10005179 | 399 | 4 | 1.00% |
| GL_Gan1_GLEAN_10009054 | 325 | 0 | 0% |
| GL_Gan1_GLEAN_10005483 | 147 | 4 | 2.72% |
| GL_Gan1_GLEAN_10003649 | 199 | 10 | 5.03% |
| GL_Gan1_GLEAN_10003381 | 230 | 10 | 4.35% |
| GL_Gan1_GLEAN_10003743 | 995 | 8 | 0.80% |
| GL_Gan1_GLEAN_10009205 | 705 | 19 | 2.70% |
| GL_Gan1_GLEAN_10003578 | 553 | 6 | 1.08% |
| GL_Gan1_GLEAN_10006004 | 557 | 8 | 1.44% |
| GL_Gan1_GLEAN_10001010 | 524 | 5 | 0.95% |
| GL_Gan1_GLEAN_10007310 | 191 | 0 | 0% |
| GL_Gan1_GLEAN_10004108 | 139 | 2 | 1.44% |
| GL_Gan1_GLEAN_10002077 | 318 | 4 | 1.26% |
| GL_Gan1_GLEAN_10008576 | 955 | 41 | 4.29% |
| GL_Gan1_GLEAN_10008392 | 155 | 2 | 1.29% |
| GL_Gan1_GLEAN_10008645 | 292 | 1 | 0.34% |
| GL_Gan1_GLEAN_10003430 | 534 | 4 | 0.75% |
| GL_Gan1_GLEAN_10000643 | 97 | 7 | 7.22% |
| GL_Gan1_GLEAN_10005638 | 191 | 6 | 3.14% |
| GL_Gan1_GLEAN_10009029 | 379 | 3 | 0.79% |
| GL_Gan1_GLEAN_10007412 | 547 | 3 | 0.55% |
| GL_Gan1_GLEAN_10007717 | 129 | 4 | 3.10% |
| GL_Gan1_GLEAN_10003772 | 410 | 0 | 0% |
| GL_Gan1_GLEAN_10000753 | 406 | 7 | 1.72% |
| GL_Gan1_GLEAN_10004398 | 585 | 12 | 2.05% |
| GL_Gan1_GLEAN_10007051 | 575 | 0 | 0% |
| GL_Gan1_GLEAN_10003135 | 98 | 0 | 0% |
| GL_Gan1_GLEAN_10008748 | 353 | 0 | 0% |
| GL_Gan1_GLEAN_10008273 | 269 | 4 | 1.49% |
| GL_Gan1_GLEAN_10007785 | 754 | 14 | 1.86% |
| GL_Gan1_GLEAN_10007235 | 416 | 6 | 1.44% |
| GL_Gan1_GLEAN_10006912 | 440 | 2 | 0.45% |
| GL_Gan1_GLEAN_10006163 | 567 | 3 | 0.53% |
| GL_Gan1_GLEAN_10008681 | 374 | 9 | 2.41% |
| GL_Gan1_GLEAN_10004813 | 437 | 9 | 2.06% |
| GL_Gan1_GLEAN_10004511 | 332 | 8 | 2.41% |
| GL_Gan1_GLEAN_10002725 | 95 | 10 | 10.53% |
| GL_Gan1_GLEAN_10002449 | 477 | 2 | 0.42% |
| GL_Gan1_GLEAN_10000577 | 392 | 11 | 2.81% |
| GL_Gan1_GLEAN_10001004 | 252 | 5 | 1.98% |
| GL_Gan1_GLEAN_10004713 | 562 | 8 | 1.42% |
| GL_Gan1_GLEAN_10005569 | 592 | 7 | 1.18% |
| GL_Gan1_GLEAN_10008816 | 320 | 3 | 0.94% |
| GL_Gan1_GLEAN_10008308 | 357 | 7 | 1.96% |
| GL_Gan1_GLEAN_10004066 | 179 | 7 | 3.91% |
| GL_Gan1_GLEAN_10009176 | 427 | 3 | 0.70% |
| GL_Gan1_GLEAN_10007961 | 156 | 8 | 5.13% |
| GL_Gan1_GLEAN_10002454 | 379 | 18 | 4.75% |
| GL_Gan1_GLEAN_10008153 | 398 | 4 | 1.01% |
| GL_Gan1_GLEAN_10008092 | 164 | 8 | 4.88% |
| GL_Gan1_GLEAN_10003025 | 375 | 3 | 0.80% |
| GL_Gan1_GLEAN_10003282 | 495 | 9 | 1.82% |
| GL_Gan1_GLEAN_10008360 | 398 | 3 | 0.75% |
| GL_Gan1_GLEAN_10009212 | 364 | 19 | 5.22% |
| GL_Gan1_GLEAN_10004421 | 221 | 17 | 7.69% |
| GL_Gan1_GLEAN_10006807 | 454 | 18 | 3.96% |
| GL_Gan1_GLEAN_10007135 | 489 | 5 | 1.02% |
| GL_Gan1_GLEAN_10005696 | 463 | 0 | 0% |
| GL_Gan1_GLEAN_10008398 | 952 | 9 | 0.95% |
| GL_Gan1_GLEAN_10007453 | 386 | 3 | 0.78% |
| GL_Gan1_GLEAN_10008271 | 535 | 22 | 4.11% |
| GL_Gan1_GLEAN_10007443 | 241 | 4 | 1.66% |
| GL_Gan1_GLEAN_10006205 | 338 | 5 | 1.48% |
| GL_Gan1_GLEAN_10006894 | 91 | 10 | 10.99% |
| GL_Gan1_GLEAN_10008152 | 487 | 4 | 0.82% |
| GL_Gan1_GLEAN_10002243 | 398 | 0 | 0% |
| GL_Gan1_GLEAN_10001126 | 210 | 5 | 2.38% |
| GL_Gan1_GLEAN_10007632 | 244 | 5 | 2.05% |
| GL_Gan1_GLEAN_10003692 | 153 | 8 | 5.23% |
| GL_Gan1_GLEAN_10008090 | 655 | 26 | 3.97% |
| GL_Gan1_GLEAN_10008204 | 530 | 2 | 0.38% |
| GL_Gan1_GLEAN_10008206 | 121 | 5 | 4.13% |
| GL_Gan1_GLEAN_10002903 | 103 | 6 | 5.83% |
| GL_Gan1_GLEAN_10009206 | 223 | 4 | 1.79% |
| GL_Gan1_GLEAN_10006005 | 273 | 0 | 0% |
| GL_Gan1_GLEAN_10000591 | 350 | 5 | 1.43% |
| GL_Gan1_GLEAN_10005087 | 180 | 0 | 0% |
| GL_Gan1_GLEAN_10002521 | 614 | 7 | 1.14% |
| GL_Gan1_GLEAN_10004491 | 420 | 4 | 0.95% |
| GL_Gan1_GLEAN_10003985 | 385 | 3 | 0.78% |
| GL_Gan1_GLEAN_10006659 | 138 | 2 | 1.45% |
| GL_Gan1_GLEAN_10005681 | 209 | 4 | 1.91% |
| GL_Gan1_GLEAN_10008807 | 174 | 4 | 2.30% |
| GL_Gan1_GLEAN_10008563 | 150 | 0 | 0% |
| GL_Gan1_GLEAN_10004872 | 393 | 10 | 2.54% |
| GL_Gan1_GLEAN_10005676 | 118 | 10 | 8.47% |
| GL_Gan1_GLEAN_10007696 | 601 | 7 | 1.16% |
| GL_Gan1_GLEAN_10000550 | 546 | 4 | 0.73% |
| GL_Gan1_GLEAN_10000449 | 1460 | 5 | 0.34% |
| GL_Gan1_GLEAN_10007363 | 377 | 6 | 1.59% |
| GL_Gan1_GLEAN_10003363 | 259 | 2 | 0.77% |
| GL_Gan1_GLEAN_10006739 | 297 | 8 | 2.69% |
| GL_Gan1_GLEAN_10004906 | 747 | 2 | 0.27% |
| GL_Gan1_GLEAN_10007598 | 182 | 5 | 2.75% |
| GL_Gan1_GLEAN_10001117 | 591 | 10 | 1.69% |
| GL_Gan1_GLEAN_10003250 | 296 | 4 | 1.35% |
| GL_Gan1_GLEAN_10004469 | 250 | 3 | 1.20% |
| GL_Gan1_GLEAN_10004623 | 124 | 6 | 4.84% |
| GL_Gan1_GLEAN_10001405 | 533 | 12 | 2.25% |
| GL_Gan1_GLEAN_10007684 | 407 | 3 | 0.74% |
| GL_Gan1_GLEAN_10005083 | 316 | 4 | 1.27% |
| GL_Gan1_GLEAN_10007773 | 294 | 7 | 2.38% |
| GL_Gan1_GLEAN_10001826 | 280 | 9 | 3.21% |
| GL_Gan1_GLEAN_10008349 | 927 | 3 | 0.32% |
| GL_Gan1_GLEAN_10007676 | 136 | 6 | 4.41% |
| GL_Gan1_GLEAN_10000303 | 279 | 7 | 2.51% |
| GL_Gan1_GLEAN_10006011 | 496 | 3 | 0.60% |
| GL_Gan1_GLEAN_10004317 | 276 | 6 | 2.17% |
| GL_Gan1_GLEAN_10004327 | 482 | 12 | 2.49% |
| GL_Gan1_GLEAN_10000654 | 517 | 7 | 1.35% |
| GL_Gan1_GLEAN_10009425 | 395 | 7 | 1.77% |
| GL_Gan1_GLEAN_10001138 | 270 | 4 | 1.48% |
| GL_Gan1_GLEAN_10003958 | 584 | 6 | 1.03% |
| GL_Gan1_GLEAN_10007989 | 337 | 1 | 0.30% |
| GL_Gan1_GLEAN_10000684 | 770 | 6 | 0.78% |
| GL_Gan1_GLEAN_10003341 | 559 | 10 | 1.79% |
| GL_Gan1_GLEAN_10002806 | 520 | 13 | 2.50% |
| GL_Gan1_GLEAN_10005176 | 269 | 5 | 1.86% |
| GL_Gan1_GLEAN_10000740 | 590 | 4 | 0.68% |
| GL_Gan1_GLEAN_10008299 | 1013 | 8 | 0.79% |
| GL_Gan1_GLEAN_10006848 | 180 | 4 | 2.22% |
| GL_Gan1_GLEAN_10002063 | 683 | 7 | 1.02% |
| GL_Gan1_GLEAN_10007995 | 849 | 14 | 1.65% |
| GL_Gan1_GLEAN_10004533 | 109 | 8 | 7.34% |
| GL_Gan1_GLEAN_10001568 | 292 | 6 | 2.05% |
| GL_Gan1_GLEAN_10002904 | 243 | 3 | 1.23% |
| GL_Gan1_GLEAN_10002088 | 469 | 4 | 0.85% |
| GL_Gan1_GLEAN_10008085 | 254 | 0 | 0% |
| GL_Gan1_GLEAN_10004507 | 261 | 0 | 0% |
| GL_Gan1_GLEAN_10007699 | 162 | 0 | 0% |
| GL_Gan1_GLEAN_10001463 | 137 | 7 | 5.11% |
| GL_Gan1_GLEAN_10004801 | 391 | 4 | 1.02% |
| GL_Gan1_GLEAN_10001860 | 437 | 6 | 1.37% |
| GL_Gan1_GLEAN_10002695 | 737 | 7 | 0.95% |
| GL_Gan1_GLEAN_10006579 | 464 | 0 | 0% |
| GL_Gan1_GLEAN_10006162 | 572 | 7 | 1.22% |
| GL_Gan1_GLEAN_10005246 | 255 | 4 | 1.57% |
| GL_Gan1_GLEAN_10001180 | 984 | 39 | 3.96% |
| GL_Gan1_GLEAN_10006552 | 408 | 4 | 0.98% |
| GL_Gan1_GLEAN_10004928 | 97 | 6 | 6.19% |
| GL_Gan1_GLEAN_10005589 | 604 | 21 | 3.48% |
| GL_Gan1_GLEAN_10002833 | 255 | 3 | 1.18% |
| GL_Gan1_GLEAN_10000153 | 1062 | 57 | 5.37% |
| GL_Gan1_GLEAN_10006810 | 503 | 4 | 0.80% |
| GL_Gan1_GLEAN_10004520 | 582 | 12 | 2.06% |
| GL_Gan1_GLEAN_10006745 | 515 | 7 | 1.36% |
| GL_Gan1_GLEAN_10004859 | 170 | 5 | 2.94% |
| GL_Gan1_GLEAN_10005370 | 521 | 5 | 0.96% |
| GL_Gan1_GLEAN_10002046 | 176 | 5 | 2.84% |
| GL_Gan1_GLEAN_10008736 | 535 | 25 | 4.67% |
| GL_Gan1_GLEAN_10008705 | 489 | 4 | 0.82% |
| GL_Gan1_GLEAN_10008929 | 541 | 6 | 1.11% |
| GL_Gan1_GLEAN_10002706 | 595 | 6 | 1.01% |
| GL_Gan1_GLEAN_10000542 | 324 | 2 | 0.62% |
| GL_Gan1_GLEAN_10004464 | 220 | 4 | 1.82% |
| GL_Gan1_GLEAN_10000878 | 246 | 2 | 0.81% |
| GL_Gan1_GLEAN_10006922 | 433 | 0 | 0% |
| GL_Gan1_GLEAN_10007772 | 240 | 5 | 2.08% |
| GL_Gan1_GLEAN_10004360 | 238 | 3 | 1.26% |
| GL_Gan1_GLEAN_10004909 | 217 | 8 | 3.69% |
| GL_Gan1_GLEAN_10001795 | 346 | 20 | 5.78% |
| GL_Gan1_GLEAN_10006060 | 579 | 9 | 1.55% |
| GL_Gan1_GLEAN_10003033 | 267 | 3 | 1.12% |
| GL_Gan1_GLEAN_10008717 | 388 | 5 | 1.29% |
| GL_Gan1_GLEAN_10000245 | 512 | 5 | 0.98% |
| GL_Gan1_GLEAN_10003763 | 1256 | 33 | 2.63% |
| GL_Gan1_GLEAN_10007001 | 352 | 3 | 0.85% |
| GL_Gan1_GLEAN_10007210 | 213 | 0 | 0% |
| GL_Gan1_GLEAN_10004433 | 656 | 7 | 1.07% |
| GL_Gan1_GLEAN_10005962 | 363 | 20 | 5.51% |
| GL_Gan1_GLEAN_10000159 | 592 | 1 | 0.17% |
| GL_Gan1_GLEAN_10005071 | 625 | 4 | 0.64% |
| GL_Gan1_GLEAN_10003053 | 237 | 10 | 4.22% |
| GL_Gan1_GLEAN_10007233 | 644 | 5 | 0.78% |
| GL_Gan1_GLEAN_10008847 | 429 | 10 | 2.33% |

**(D)** Genes under positive selection pressure (PSP)

| Sequence | GO Annotation | Ka/Ks | P-Value(Fisher) |
| --- | --- | --- | --- |
| GL_Gan1_GLEAN_10000302_GL_Gan1&GL_Gan1_GLEAN_10000427_GL_Gan1 | CAZy | 1.67282 | 7.49E-05 |
| GL_Gan1_GLEAN_10000348_GL_Gan1&PDIG_27770_PHI26 | CAZy | 2.18884 | 0.001866 |
| GL_Gan1_GLEAN_10001372_GL_Gan1&PEX1_006850_CMP1 | CAZy | 2.07769 | 0.007521 |
| GL_Gan1_GLEAN_10001372_GL_Gan1&PEX2_015350_MD8 | CAZy | 2.07769 | 0.007521 |
| GL_Gan1_GLEAN_10003174_GL_Gan1&PEX1_006850_CMP1 | CAZy | 3.52969 | 0.000157 |
| GL_Gan1_GLEAN_10003174_GL_Gan1&PEX2_015350_MD8 | CAZy | 3.52969 | 0.000157 |
| GL_Gan1_GLEAN_10003333_GL_Gan1&PDIG_14160_PHI26 | Protein of unknown function | 1.89459 | 0.000212 |
| GL_Gan1_GLEAN_10003667_GL_Gan1&PEX1_019270_CMP1 | Protein of unknown function | 2.10202 | 4.85E-05 |
| GL_Gan1_GLEAN_10003667_GL_Gan1&PEX2_109680_MD8 | Protein of unknown function | 2.10202 | 4.85E-05 |
| GL_Gan1_GLEAN_10003667_GL_Gan1&PEXP_028930_Pd1 | Protein of unknown function | 2.0875 | 6.63E-05 |
| GL_Gan1_GLEAN_10004029_GL_Gan1&PITC_052640_PHI1 | PHI | 10.9066 | 0.002329 |
| GL_Gan1_GLEAN_10004714_GL_Gan1&PEX1_044020_CMP1 | P450 | 1.36452 | 0.004129 |
| GL_Gan1_GLEAN_10004714_GL_Gan1&PEX2_017620_MD8 | P450 | 1.36452 | 0.004129 |
| GL_Gan1_GLEAN_10004932_GL_Gan1&GL_Gan1_GLEAN_10008674_GL_Gan1 | CAZy | 1.33679 | 0.00222 |
| GL_Gan1_GLEAN_10007042_GL_Gan1&PITC_095290_PHI1 | Keratin-associated protein | 2.97559 | 4.45E-12 |
| GL_Gan1_GLEAN_10008289_GL_Gan1&GL_Gan1_GLEAN_10008290_GL_Gan1 | Craniofacial development protein | 2.25867 | 0.003899 |
| GL_Gan1_GLEAN_10008554_GL_Gan1&PDIG_14610_PHI26 | PHI | 1.50026 | 0.009833 |

**(E)** Annotation with GO for HGT

| Ontology | Class | number_of_GL_Gan1 | | genes_of_GL_Gan1 |
| --- | --- | --- | --- | --- |
| biological_process | biological adhesion | 1 | GL_Gan1_GLEAN_10008313 | |
|  | biological regulation | 4 | GL_Gan1_GLEAN_10005568;GL_Gan1_GLEAN_10002062;GL_Gan1_GLEAN_10008690;GL_Gan1_GLEAN_10002677 | |
|  | carbon utilization | 2 | GL_Gan1_GLEAN_10002334;GL_Gan1_GLEAN_10002884 | |
|  | cellular component organization or biogenesis | 3 | GL_Gan1_GLEAN_10009259;GL_Gan1_GLEAN_10009399;GL_Gan1_GLEAN_10005651 | |
|  | cellular process | 76 | GL_Gan1_GLEAN_10005434;GL_Gan1_GLEAN_10005568;GL_Gan1_GLEAN_10001987;GL_Gan1_GLEAN_10007371;GL_Gan1_GLEAN_10008272;GL_Gan1_GLEAN_10008450;GL_Gan1_GLEAN_10002483;GL_Gan1_GLEAN_10001603;GL_Gan1_GLEAN_10005367;GL_Gan1_GLEAN_10005979;GL_Gan1_GLEAN_10007718;GL_Gan1_GLEAN_10007733;GL_Gan1_GLEAN_10007937;GL_Gan1_GLEAN_10002062;GL_Gan1_GLEAN_10003397;GL_Gan1_GLEAN_10001523;GL_Gan1_GLEAN_10000732;GL_Gan1_GLEAN_10008911;GL_Gan1_GLEAN_10009000;GL_Gan1_GLEAN_10009029;GL_Gan1_GLEAN_10000001;GL_Gan1_GLEAN_10002729;GL_Gan1_GLEAN_10008261;GL_Gan1_GLEAN_10006095;GL_Gan1_GLEAN_10008907;GL_Gan1_GLEAN_10009259;GL_Gan1_GLEAN_10009271;GL_Gan1_GLEAN_10000420;GL_Gan1_GLEAN_10001125;GL_Gan1_GLEAN_10008690;GL_Gan1_GLEAN_10007763;GL_Gan1_GLEAN_10002677;GL_Gan1_GLEAN_10007665;GL_Gan1_GLEAN_10001487;GL_Gan1_GLEAN_10001543;GL_Gan1_GLEAN_10005895;GL_Gan1_GLEAN_10006585;GL_Gan1_GLEAN_10009399;GL_Gan1_GLEAN_10004431;GL_Gan1_GLEAN_10007449;GL_Gan1_GLEAN_10002881;GL_Gan1_GLEAN_10003120;GL_Gan1_GLEAN_10008352;GL_Gan1_GLEAN_10004323;GL_Gan1_GLEAN_10005006;GL_Gan1_GLEAN_10008180;GL_Gan1_GLEAN_10004270;GL_Gan1_GLEAN_10006192;GL_Gan1_GLEAN_10004884;GL_Gan1_GLEAN_10008802;GL_Gan1_GLEAN_10007241;GL_Gan1_GLEAN_10008709;GL_Gan1_GLEAN_10009160;GL_Gan1_GLEAN_10000111;GL_Gan1_GLEAN_10001002;GL_Gan1_GLEAN_10003768;GL_Gan1_GLEAN_10006272;GL_Gan1_GLEAN_10007420;GL_Gan1_GLEAN_10009139;GL_Gan1_GLEAN_10001386;GL_Gan1_GLEAN_10007413;GL_Gan1_GLEAN_10005414;GL_Gan1_GLEAN_10000483;GL_Gan1_GLEAN_10004441;GL_Gan1_GLEAN_10002516;GL_Gan1_GLEAN_10000842;GL_Gan1_GLEAN_10002143;GL_Gan1_GLEAN_10004774;GL_Gan1_GLEAN_10005651;GL_Gan1_GLEAN_10005601;GL_Gan1_GLEAN_10008313;GL_Gan1_GLEAN_10005339;GL_Gan1_GLEAN_10006784;GL_Gan1_GLEAN_10007083;GL_Gan1_GLEAN_10008511;GL_Gan1_GLEAN_10007812 | |
|  | establishment of localization | 12 | GL_Gan1_GLEAN_10001603;GL_Gan1_GLEAN_10005367;GL_Gan1_GLEAN_10005979;GL_Gan1_GLEAN_10007718;GL_Gan1_GLEAN_10007733;GL_Gan1_GLEAN_10007937;GL_Gan1_GLEAN_10003397;GL_Gan1_GLEAN_10002334;GL_Gan1_GLEAN_10002884;GL_Gan1_GLEAN_10007701;GL_Gan1_GLEAN_10005601;GL_Gan1_GLEAN_10005171 | |
|  | localization | 12 | GL_Gan1_GLEAN_10001603;GL_Gan1_GLEAN_10005367;GL_Gan1_GLEAN_10005979;GL_Gan1_GLEAN_10007718;GL_Gan1_GLEAN_10007733;GL_Gan1_GLEAN_10007937;GL_Gan1_GLEAN_10003397;GL_Gan1_GLEAN_10002334;GL_Gan1_GLEAN_10002884;GL_Gan1_GLEAN_10007701;GL_Gan1_GLEAN_10005601;GL_Gan1_GLEAN_10005171 | |
|  | metabolic process | 239 | GL_Gan1_GLEAN_10000087;GL_Gan1_GLEAN_10000111;GL_Gan1_GLEAN_10000298;GL_Gan1_GLEAN_10000420;GL_Gan1_GLEAN_10000560;GL_Gan1_GLEAN_10000587;GL_Gan1_GLEAN_10000590;GL_Gan1_GLEAN_10000712;GL_Gan1_GLEAN_10000737;GL_Gan1_GLEAN_10000874;GL_Gan1_GLEAN_10000875;GL_Gan1_GLEAN_10000944;GL_Gan1_GLEAN_10001125;GL_Gan1_GLEAN_10001249;GL_Gan1_GLEAN_10001498;GL_Gan1_GLEAN_10001522;GL_Gan1_GLEAN_10001569;GL_Gan1_GLEAN_10001641;GL_Gan1_GLEAN_10001758;GL_Gan1_GLEAN_10002027;GL_Gan1_GLEAN_10002172;GL_Gan1_GLEAN_10002306;GL_Gan1_GLEAN_10002670;GL_Gan1_GLEAN_10002688;GL_Gan1_GLEAN_10002913;GL_Gan1_GLEAN_10003193;GL_Gan1_GLEAN_10003467;GL_Gan1_GLEAN_10003523;GL_Gan1_GLEAN_10003550;GL_Gan1_GLEAN_10003623;GL_Gan1_GLEAN_10003691;GL_Gan1_GLEAN_10003693;GL_Gan1_GLEAN_10003873;GL_Gan1_GLEAN_10003940;GL_Gan1_GLEAN_10004036;GL_Gan1_GLEAN_10004039;GL_Gan1_GLEAN_10004216;GL_Gan1_GLEAN_10004217;GL_Gan1_GLEAN_10004267;GL_Gan1_GLEAN_10004407;GL_Gan1_GLEAN_10004535;GL_Gan1_GLEAN_10004632;GL_Gan1_GLEAN_10004644;GL_Gan1_GLEAN_10004667;GL_Gan1_GLEAN_10004811;GL_Gan1_GLEAN_10004832;GL_Gan1_GLEAN_10004834;GL_Gan1_GLEAN_10005349;GL_Gan1_GLEAN_10005379;GL_Gan1_GLEAN_10005434;GL_Gan1_GLEAN_10005564;GL_Gan1_GLEAN_10005576;GL_Gan1_GLEAN_10005628;GL_Gan1_GLEAN_10005787;GL_Gan1_GLEAN_10005806;GL_Gan1_GLEAN_10006125;GL_Gan1_GLEAN_10006136;GL_Gan1_GLEAN_10006575;GL_Gan1_GLEAN_10006618;GL_Gan1_GLEAN_10006767;GL_Gan1_GLEAN_10006780;GL_Gan1_GLEAN_10006791;GL_Gan1_GLEAN_10006831;GL_Gan1_GLEAN_10006923;GL_Gan1_GLEAN_10007061;GL_Gan1_GLEAN_10007241;GL_Gan1_GLEAN_10007404;GL_Gan1_GLEAN_10007413;GL_Gan1_GLEAN_10007460;GL_Gan1_GLEAN_10007759;GL_Gan1_GLEAN_10007812;GL_Gan1_GLEAN_10007905;GL_Gan1_GLEAN_10007935;GL_Gan1_GLEAN_10008151;GL_Gan1_GLEAN_10008175;GL_Gan1_GLEAN_10008180;GL_Gan1_GLEAN_10008272;GL_Gan1_GLEAN_10008328;GL_Gan1_GLEAN_10008352;GL_Gan1_GLEAN_10008444;GL_Gan1_GLEAN_10008491;GL_Gan1_GLEAN_10008512;GL_Gan1_GLEAN_10008704;GL_Gan1_GLEAN_10008712;GL_Gan1_GLEAN_10008808;GL_Gan1_GLEAN_10008911;GL_Gan1_GLEAN_10009036;GL_Gan1_GLEAN_10009097;GL_Gan1_GLEAN_10009128;GL_Gan1_GLEAN_10009160;GL_Gan1_GLEAN_10009169;GL_Gan1_GLEAN_10009198;GL_Gan1_GLEAN_10009388;GL_Gan1_GLEAN_10001987;GL_Gan1_GLEAN_10007371;GL_Gan1_GLEAN_10009139;GL_Gan1_GLEAN_10002483;GL_Gan1_GLEAN_10002062;GL_Gan1_GLEAN_10005568;GL_Gan1_GLEAN_10003397;GL_Gan1_GLEAN_10001523;GL_Gan1_GLEAN_10000187;GL_Gan1_GLEAN_10000732;GL_Gan1_GLEAN_10009000;GL_Gan1_GLEAN_10009029;GL_Gan1_GLEAN_10000001;GL_Gan1_GLEAN_10002729;GL_Gan1_GLEAN_10002088;GL_Gan1_GLEAN_10008261;GL_Gan1_GLEAN_10006095;GL_Gan1_GLEAN_10008907;GL_Gan1_GLEAN_10004562;GL_Gan1_GLEAN_10009271;GL_Gan1_GLEAN_10001913;GL_Gan1_GLEAN_10004607;GL_Gan1_GLEAN_10007763;GL_Gan1_GLEAN_10007665;GL_Gan1_GLEAN_10001487;GL_Gan1_GLEAN_10001543;GL_Gan1_GLEAN_10005895;GL_Gan1_GLEAN_10006585;GL_Gan1_GLEAN_10009399;GL_Gan1_GLEAN_10004431;GL_Gan1_GLEAN_10007449;GL_Gan1_GLEAN_10002881;GL_Gan1_GLEAN_10003120;GL_Gan1_GLEAN_10004323;GL_Gan1_GLEAN_10000181;GL_Gan1_GLEAN_10000183;GL_Gan1_GLEAN_10000555;GL_Gan1_GLEAN_10000719;GL_Gan1_GLEAN_10000814;GL_Gan1_GLEAN_10000826;GL_Gan1_GLEAN_10000849;GL_Gan1_GLEAN_10000855;GL_Gan1_GLEAN_10000898;GL_Gan1_GLEAN_10001131;GL_Gan1_GLEAN_10001371;GL_Gan1_GLEAN_10001449;GL_Gan1_GLEAN_10001965;GL_Gan1_GLEAN_10002065;GL_Gan1_GLEAN_10002233;GL_Gan1_GLEAN_10002235;GL_Gan1_GLEAN_10002241;GL_Gan1_GLEAN_10002372;GL_Gan1_GLEAN_10002449;GL_Gan1_GLEAN_10002529;GL_Gan1_GLEAN_10002732;GL_Gan1_GLEAN_10002847;GL_Gan1_GLEAN_10002995;GL_Gan1_GLEAN_10003006;GL_Gan1_GLEAN_10003089;GL_Gan1_GLEAN_10003172;GL_Gan1_GLEAN_10003252;GL_Gan1_GLEAN_10003879;GL_Gan1_GLEAN_10004610;GL_Gan1_GLEAN_10005199;GL_Gan1_GLEAN_10005384;GL_Gan1_GLEAN_10005414;GL_Gan1_GLEAN_10005603;GL_Gan1_GLEAN_10005842;GL_Gan1_GLEAN_10005899;GL_Gan1_GLEAN_10005925;GL_Gan1_GLEAN_10005965;GL_Gan1_GLEAN_10006181;GL_Gan1_GLEAN_10006617;GL_Gan1_GLEAN_10006811;GL_Gan1_GLEAN_10006812;GL_Gan1_GLEAN_10006885;GL_Gan1_GLEAN_10007097;GL_Gan1_GLEAN_10007175;GL_Gan1_GLEAN_10007701;GL_Gan1_GLEAN_10007730;GL_Gan1_GLEAN_10007760;GL_Gan1_GLEAN_10008172;GL_Gan1_GLEAN_10008363;GL_Gan1_GLEAN_10008959;GL_Gan1_GLEAN_10008994;GL_Gan1_GLEAN_10009177;GL_Gan1_GLEAN_10009180;GL_Gan1_GLEAN_10009203;GL_Gan1_GLEAN_10009371;GL_Gan1_GLEAN_10005006;GL_Gan1_GLEAN_10004270;GL_Gan1_GLEAN_10006192;GL_Gan1_GLEAN_10002689;GL_Gan1_GLEAN_10008174;GL_Gan1_GLEAN_10004884;GL_Gan1_GLEAN_10008802;GL_Gan1_GLEAN_10002334;GL_Gan1_GLEAN_10002884;GL_Gan1_GLEAN_10008709;GL_Gan1_GLEAN_10000011;GL_Gan1_GLEAN_10007895;GL_Gan1_GLEAN_10008741;GL_Gan1_GLEAN_10001002;GL_Gan1_GLEAN_10003768;GL_Gan1_GLEAN_10006272;GL_Gan1_GLEAN_10007420;GL_Gan1_GLEAN_10001386;GL_Gan1_GLEAN_10000483;GL_Gan1_GLEAN_10004441;GL_Gan1_GLEAN_10000815;GL_Gan1_GLEAN_10002758;GL_Gan1_GLEAN_10007234;GL_Gan1_GLEAN_10009012;GL_Gan1_GLEAN_10002516;GL_Gan1_GLEAN_10000842;GL_Gan1_GLEAN_10002143;GL_Gan1_GLEAN_10004774;GL_Gan1_GLEAN_10000198;GL_Gan1_GLEAN_10000339;GL_Gan1_GLEAN_10000723;GL_Gan1_GLEAN_10000851;GL_Gan1_GLEAN_10001234;GL_Gan1_GLEAN_10001684;GL_Gan1_GLEAN_10001919;GL_Gan1_GLEAN_10002588;GL_Gan1_GLEAN_10002658;GL_Gan1_GLEAN_10003073;GL_Gan1_GLEAN_10003265;GL_Gan1_GLEAN_10003348;GL_Gan1_GLEAN_10004105;GL_Gan1_GLEAN_10004402;GL_Gan1_GLEAN_10004524;GL_Gan1_GLEAN_10004544;GL_Gan1_GLEAN_10004626;GL_Gan1_GLEAN_10004906;GL_Gan1_GLEAN_10005127;GL_Gan1_GLEAN_10005419;GL_Gan1_GLEAN_10005559;GL_Gan1_GLEAN_10006566;GL_Gan1_GLEAN_10007233;GL_Gan1_GLEAN_10008068;GL_Gan1_GLEAN_10008336;GL_Gan1_GLEAN_10005339;GL_Gan1_GLEAN_10006784;GL_Gan1_GLEAN_10007083;GL_Gan1_GLEAN_10008511 | |
|  | regulation of biological process | 4 | GL_Gan1_GLEAN_10005568;GL_Gan1_GLEAN_10002062;GL_Gan1_GLEAN_10008690;GL_Gan1_GLEAN_10002677 | |
|  | response to stimulus | 10 | GL_Gan1_GLEAN_10005568;GL_Gan1_GLEAN_10001932;GL_Gan1_GLEAN_10002677;GL_Gan1_GLEAN_10007060;GL_Gan1_GLEAN_10002334;GL_Gan1_GLEAN_10002884;GL_Gan1_GLEAN_10002847;GL_Gan1_GLEAN_10005965;GL_Gan1_GLEAN_10007760;GL_Gan1_GLEAN_10002633 | |
|  | signaling | 2 | GL_Gan1_GLEAN_10005568;GL_Gan1_GLEAN_10002677 | |
| cellular_component | cell | 13 | GL_Gan1_GLEAN_10002729;GL_Gan1_GLEAN_10000483;GL_Gan1_GLEAN_10002483;GL_Gan1_GLEAN_10004441;GL_Gan1_GLEAN_10005414;GL_Gan1_GLEAN_10008341;GL_Gan1_GLEAN_10008907;GL_Gan1_GLEAN_10007665;GL_Gan1_GLEAN_10003397;GL_Gan1_GLEAN_10004270;GL_Gan1_GLEAN_10009399;GL_Gan1_GLEAN_10000357;GL_Gan1_GLEAN_10002062 | |
|  | cell part | 13 | GL_Gan1_GLEAN_10002729;GL_Gan1_GLEAN_10000483;GL_Gan1_GLEAN_10002483;GL_Gan1_GLEAN_10004441;GL_Gan1_GLEAN_10005414;GL_Gan1_GLEAN_10008341;GL_Gan1_GLEAN_10008907;GL_Gan1_GLEAN_10007665;GL_Gan1_GLEAN_10003397;GL_Gan1_GLEAN_10004270;GL_Gan1_GLEAN_10009399;GL_Gan1_GLEAN_10000357;GL_Gan1_GLEAN_10002062 | |
|  | extracellular region | 1 | GL_Gan1_GLEAN_10002588 | |
|  | macromolecular complex | 5 | GL_Gan1_GLEAN_10007665;GL_Gan1_GLEAN_10003397;GL_Gan1_GLEAN_10004105;GL_Gan1_GLEAN_10004270;GL_Gan1_GLEAN_10009399 | |
|  | membrane | 11 | GL_Gan1_GLEAN_10002729;GL_Gan1_GLEAN_10005568;GL_Gan1_GLEAN_10005979;GL_Gan1_GLEAN_10007718;GL_Gan1_GLEAN_10007733;GL_Gan1_GLEAN_10007895;GL_Gan1_GLEAN_10001603;GL_Gan1_GLEAN_10004833;GL_Gan1_GLEAN_10005367;GL_Gan1_GLEAN_10009001;GL_Gan1_GLEAN_10003397 | |
|  | membrane part | 7 | GL_Gan1_GLEAN_10001603;GL_Gan1_GLEAN_10004833;GL_Gan1_GLEAN_10005367;GL_Gan1_GLEAN_10005979;GL_Gan1_GLEAN_10007718;GL_Gan1_GLEAN_10009001;GL_Gan1_GLEAN_10003397 | |
|  | membrane-enclosed lumen | 1 | GL_Gan1_GLEAN_10009399 | |
|  | organelle | 3 | GL_Gan1_GLEAN_10009399;GL_Gan1_GLEAN_10000357;GL_Gan1_GLEAN_10002062 | |
|  | organelle part | 1 | GL_Gan1_GLEAN_10009399 | |
| molecular_function | antioxidant activity | 3 | GL_Gan1_GLEAN_10007760;GL_Gan1_GLEAN_10002847;GL_Gan1_GLEAN_10005965 | |
|  | binding | 165 | GL_Gan1_GLEAN_10001185;GL_Gan1_GLEAN_10002633;GL_Gan1_GLEAN_10003055;GL_Gan1_GLEAN_10004832;GL_Gan1_GLEAN_10006153;GL_Gan1_GLEAN_10008487;GL_Gan1_GLEAN_10007701;GL_Gan1_GLEAN_10000849;GL_Gan1_GLEAN_10001449;GL_Gan1_GLEAN_10003940;GL_Gan1_GLEAN_10005384;GL_Gan1_GLEAN_10006617;GL_Gan1_GLEAN_10007460;GL_Gan1_GLEAN_10007905;GL_Gan1_GLEAN_10008994;GL_Gan1_GLEAN_10000357;GL_Gan1_GLEAN_10002261;GL_Gan1_GLEAN_10003473;GL_Gan1_GLEAN_10002847;GL_Gan1_GLEAN_10003523;GL_Gan1_GLEAN_10005965;GL_Gan1_GLEAN_10007760;GL_Gan1_GLEAN_10000483;GL_Gan1_GLEAN_10000550;GL_Gan1_GLEAN_10000730;GL_Gan1_GLEAN_10000732;GL_Gan1_GLEAN_10001235;GL_Gan1_GLEAN_10002597;GL_Gan1_GLEAN_10003240;GL_Gan1_GLEAN_10003594;GL_Gan1_GLEAN_10003820;GL_Gan1_GLEAN_10004096;GL_Gan1_GLEAN_10004406;GL_Gan1_GLEAN_10005267;GL_Gan1_GLEAN_10005325;GL_Gan1_GLEAN_10005877;GL_Gan1_GLEAN_10006492;GL_Gan1_GLEAN_10007437;GL_Gan1_GLEAN_10008044;GL_Gan1_GLEAN_10008052;GL_Gan1_GLEAN_10008313;GL_Gan1_GLEAN_10008565;GL_Gan1_GLEAN_10000181;GL_Gan1_GLEAN_10001523;GL_Gan1_GLEAN_10002233;GL_Gan1_GLEAN_10000187;GL_Gan1_GLEAN_10006272;GL_Gan1_GLEAN_10000087;GL_Gan1_GLEAN_10000298;GL_Gan1_GLEAN_10000555;GL_Gan1_GLEAN_10000587;GL_Gan1_GLEAN_10000590;GL_Gan1_GLEAN_10000719;GL_Gan1_GLEAN_10000737;GL_Gan1_GLEAN_10000814;GL_Gan1_GLEAN_10001002;GL_Gan1_GLEAN_10001193;GL_Gan1_GLEAN_10001249;GL_Gan1_GLEAN_10001487;GL_Gan1_GLEAN_10001498;GL_Gan1_GLEAN_10001543;GL_Gan1_GLEAN_10002065;GL_Gan1_GLEAN_10002235;GL_Gan1_GLEAN_10002241;GL_Gan1_GLEAN_10002372;GL_Gan1_GLEAN_10002670;GL_Gan1_GLEAN_10003006;GL_Gan1_GLEAN_10003089;GL_Gan1_GLEAN_10003252;GL_Gan1_GLEAN_10003467;GL_Gan1_GLEAN_10003550;GL_Gan1_GLEAN_10003691;GL_Gan1_GLEAN_10003768;GL_Gan1_GLEAN_10004036;GL_Gan1_GLEAN_10004039;GL_Gan1_GLEAN_10004407;GL_Gan1_GLEAN_10004535;GL_Gan1_GLEAN_10004632;GL_Gan1_GLEAN_10004667;GL_Gan1_GLEAN_10004985;GL_Gan1_GLEAN_10005564;GL_Gan1_GLEAN_10005568;GL_Gan1_GLEAN_10005628;GL_Gan1_GLEAN_10005787;GL_Gan1_GLEAN_10005842;GL_Gan1_GLEAN_10005895;GL_Gan1_GLEAN_10005925;GL_Gan1_GLEAN_10006159;GL_Gan1_GLEAN_10006181;GL_Gan1_GLEAN_10006575;GL_Gan1_GLEAN_10006585;GL_Gan1_GLEAN_10006791;GL_Gan1_GLEAN_10006831;GL_Gan1_GLEAN_10006923;GL_Gan1_GLEAN_10007371;GL_Gan1_GLEAN_10007404;GL_Gan1_GLEAN_10007420;GL_Gan1_GLEAN_10007712;GL_Gan1_GLEAN_10007763;GL_Gan1_GLEAN_10007894;GL_Gan1_GLEAN_10007935;GL_Gan1_GLEAN_10008491;GL_Gan1_GLEAN_10008523;GL_Gan1_GLEAN_10008959;GL_Gan1_GLEAN_10009128;GL_Gan1_GLEAN_10009139;GL_Gan1_GLEAN_10009198;GL_Gan1_GLEAN_10009371;GL_Gan1_GLEAN_10009388;GL_Gan1_GLEAN_10000339;GL_Gan1_GLEAN_10002588;GL_Gan1_GLEAN_10004105;GL_Gan1_GLEAN_10004607;GL_Gan1_GLEAN_10004626;GL_Gan1_GLEAN_10007233;GL_Gan1_GLEAN_10008336;GL_Gan1_GLEAN_10005651;GL_Gan1_GLEAN_10000855;GL_Gan1_GLEAN_10007895;GL_Gan1_GLEAN_10002483;GL_Gan1_GLEAN_10001913;GL_Gan1_GLEAN_10002658;GL_Gan1_GLEAN_10002688;GL_Gan1_GLEAN_10004774;GL_Gan1_GLEAN_10005547;GL_Gan1_GLEAN_10006095;GL_Gan1_GLEAN_10005339;GL_Gan1_GLEAN_10007083;GL_Gan1_GLEAN_10000111;GL_Gan1_GLEAN_10002062;GL_Gan1_GLEAN_10002334;GL_Gan1_GLEAN_10002884;GL_Gan1_GLEAN_10007175;GL_Gan1_GLEAN_10009203;GL_Gan1_GLEAN_10008690;GL_Gan1_GLEAN_10002529;GL_Gan1_GLEAN_10002995;GL_Gan1_GLEAN_10005899;GL_Gan1_GLEAN_10006885;GL_Gan1_GLEAN_10001754;GL_Gan1_GLEAN_10005367;GL_Gan1_GLEAN_10006811;GL_Gan1_GLEAN_10006812;GL_Gan1_GLEAN_10007097;GL_Gan1_GLEAN_10008172;GL_Gan1_GLEAN_10001684;GL_Gan1_GLEAN_10006566;GL_Gan1_GLEAN_10007449;GL_Gan1_GLEAN_10009259;GL_Gan1_GLEAN_10000001;GL_Gan1_GLEAN_10000560;GL_Gan1_GLEAN_10002027;GL_Gan1_GLEAN_10002881;GL_Gan1_GLEAN_10002913;GL_Gan1_GLEAN_10003120;GL_Gan1_GLEAN_10003623;GL_Gan1_GLEAN_10003767;GL_Gan1_GLEAN_10004323;GL_Gan1_GLEAN_10004376;GL_Gan1_GLEAN_10006192;GL_Gan1_GLEAN_10008146;GL_Gan1_GLEAN_10009160;GL_Gan1_GLEAN_10001381;GL_Gan1_GLEAN_10008272;GL_Gan1_GLEAN_10009399 | |
|  | biological regulation | 4 | GL_Gan1_GLEAN_10005568;GL_Gan1_GLEAN_10000357;GL_Gan1_GLEAN_10002062;GL_Gan1_GLEAN_10002677 | |
|  | catalytic activity | 291 | GL_Gan1_GLEAN_10000842;GL_Gan1_GLEAN_10002143;GL_Gan1_GLEAN_10007413;GL_Gan1_GLEAN_10009271;GL_Gan1_GLEAN_10000874;GL_Gan1_GLEAN_10004216;GL_Gan1_GLEAN_10008512;GL_Gan1_GLEAN_10004832;GL_Gan1_GLEAN_10005427;GL_Gan1_GLEAN_10005568;GL_Gan1_GLEAN_10008352;GL_Gan1_GLEAN_10000011;GL_Gan1_GLEAN_10000281;GL_Gan1_GLEAN_10000339;GL_Gan1_GLEAN_10000420;GL_Gan1_GLEAN_10000483;GL_Gan1_GLEAN_10000560;GL_Gan1_GLEAN_10000875;GL_Gan1_GLEAN_10001002;GL_Gan1_GLEAN_10001125;GL_Gan1_GLEAN_10001363;GL_Gan1_GLEAN_10001381;GL_Gan1_GLEAN_10001386;GL_Gan1_GLEAN_10001523;GL_Gan1_GLEAN_10001684;GL_Gan1_GLEAN_10001758;GL_Gan1_GLEAN_10001901;GL_Gan1_GLEAN_10002027;GL_Gan1_GLEAN_10002416;GL_Gan1_GLEAN_10002529;GL_Gan1_GLEAN_10002588;GL_Gan1_GLEAN_10002688;GL_Gan1_GLEAN_10002730;GL_Gan1_GLEAN_10002881;GL_Gan1_GLEAN_10002913;GL_Gan1_GLEAN_10003120;GL_Gan1_GLEAN_10003193;GL_Gan1_GLEAN_10003223;GL_Gan1_GLEAN_10003348;GL_Gan1_GLEAN_10003523;GL_Gan1_GLEAN_10003623;GL_Gan1_GLEAN_10003693;GL_Gan1_GLEAN_10003767;GL_Gan1_GLEAN_10003768;GL_Gan1_GLEAN_10003844;GL_Gan1_GLEAN_10004022;GL_Gan1_GLEAN_10004105;GL_Gan1_GLEAN_10004267;GL_Gan1_GLEAN_10004323;GL_Gan1_GLEAN_10004376;GL_Gan1_GLEAN_10004402;GL_Gan1_GLEAN_10004431;GL_Gan1_GLEAN_10004441;GL_Gan1_GLEAN_10004607;GL_Gan1_GLEAN_10004626;GL_Gan1_GLEAN_10004632;GL_Gan1_GLEAN_10004811;GL_Gan1_GLEAN_10004834;GL_Gan1_GLEAN_10004906;GL_Gan1_GLEAN_10005127;GL_Gan1_GLEAN_10005379;GL_Gan1_GLEAN_10005418;GL_Gan1_GLEAN_10005434;GL_Gan1_GLEAN_10005521;GL_Gan1_GLEAN_10005559;GL_Gan1_GLEAN_10005576;GL_Gan1_GLEAN_10005628;GL_Gan1_GLEAN_10005806;GL_Gan1_GLEAN_10005899;GL_Gan1_GLEAN_10006125;GL_Gan1_GLEAN_10006192;GL_Gan1_GLEAN_10006204;GL_Gan1_GLEAN_10006272;GL_Gan1_GLEAN_10006566;GL_Gan1_GLEAN_10006575;GL_Gan1_GLEAN_10006780;GL_Gan1_GLEAN_10006885;GL_Gan1_GLEAN_10007061;GL_Gan1_GLEAN_10007096;GL_Gan1_GLEAN_10007233;GL_Gan1_GLEAN_10007241;GL_Gan1_GLEAN_10007353;GL_Gan1_GLEAN_10007420;GL_Gan1_GLEAN_10007769;GL_Gan1_GLEAN_10007812;GL_Gan1_GLEAN_10008146;GL_Gan1_GLEAN_10008151;GL_Gan1_GLEAN_10008175;GL_Gan1_GLEAN_10008180;GL_Gan1_GLEAN_10008261;GL_Gan1_GLEAN_10008328;GL_Gan1_GLEAN_10008336;GL_Gan1_GLEAN_10008704;GL_Gan1_GLEAN_10008808;GL_Gan1_GLEAN_10008864;GL_Gan1_GLEAN_10008907;GL_Gan1_GLEAN_10009139;GL_Gan1_GLEAN_10009160;GL_Gan1_GLEAN_10009169;GL_Gan1_GLEAN_10001978;GL_Gan1_GLEAN_10006142;GL_Gan1_GLEAN_10009378;GL_Gan1_GLEAN_10003940;GL_Gan1_GLEAN_10007460;GL_Gan1_GLEAN_10007905;GL_Gan1_GLEAN_10000555;GL_Gan1_GLEAN_10002235;GL_Gan1_GLEAN_10002372;GL_Gan1_GLEAN_10003006;GL_Gan1_GLEAN_10003089;GL_Gan1_GLEAN_10003252;GL_Gan1_GLEAN_10005842;GL_Gan1_GLEAN_10006181;GL_Gan1_GLEAN_10008959;GL_Gan1_GLEAN_10009203;GL_Gan1_GLEAN_10009371;GL_Gan1_GLEAN_10004833;GL_Gan1_GLEAN_10005414;GL_Gan1_GLEAN_10000111;GL_Gan1_GLEAN_10001569;GL_Gan1_GLEAN_10004217;GL_Gan1_GLEAN_10004644;GL_Gan1_GLEAN_10009097;GL_Gan1_GLEAN_10006784;GL_Gan1_GLEAN_10008511;GL_Gan1_GLEAN_10000181;GL_Gan1_GLEAN_10000898;GL_Gan1_GLEAN_10001131;GL_Gan1_GLEAN_10002233;GL_Gan1_GLEAN_10007730;GL_Gan1_GLEAN_10000161;GL_Gan1_GLEAN_10000815;GL_Gan1_GLEAN_10002758;GL_Gan1_GLEAN_10005639;GL_Gan1_GLEAN_10007234;GL_Gan1_GLEAN_10009012;GL_Gan1_GLEAN_10004270;GL_Gan1_GLEAN_10008272;GL_Gan1_GLEAN_10007371;GL_Gan1_GLEAN_10000001;GL_Gan1_GLEAN_10000719;GL_Gan1_GLEAN_10000855;GL_Gan1_GLEAN_10001487;GL_Gan1_GLEAN_10001543;GL_Gan1_GLEAN_10002241;GL_Gan1_GLEAN_10004407;GL_Gan1_GLEAN_10005564;GL_Gan1_GLEAN_10005895;GL_Gan1_GLEAN_10006585;GL_Gan1_GLEAN_10007763;GL_Gan1_GLEAN_10000198;GL_Gan1_GLEAN_10000723;GL_Gan1_GLEAN_10001234;GL_Gan1_GLEAN_10003073;GL_Gan1_GLEAN_10003265;GL_Gan1_GLEAN_10004524;GL_Gan1_GLEAN_10004544;GL_Gan1_GLEAN_10005419;GL_Gan1_GLEAN_10008068;GL_Gan1_GLEAN_10000814;GL_Gan1_GLEAN_10007895;GL_Gan1_GLEAN_10008741;GL_Gan1_GLEAN_10001243;GL_Gan1_GLEAN_10002284;GL_Gan1_GLEAN_10004070;GL_Gan1_GLEAN_10004380;GL_Gan1_GLEAN_10008464;GL_Gan1_GLEAN_10006767;GL_Gan1_GLEAN_10009036;GL_Gan1_GLEAN_10002658;GL_Gan1_GLEAN_10000849;GL_Gan1_GLEAN_10005384;GL_Gan1_GLEAN_10006617;GL_Gan1_GLEAN_10008994;GL_Gan1_GLEAN_10001061;GL_Gan1_GLEAN_10002050;GL_Gan1_GLEAN_10003837;GL_Gan1_GLEAN_10003979;GL_Gan1_GLEAN_10005226;GL_Gan1_GLEAN_10005821;GL_Gan1_GLEAN_10006243;GL_Gan1_GLEAN_10007752;GL_Gan1_GLEAN_10008567;GL_Gan1_GLEAN_10008675;GL_Gan1_GLEAN_10008911;GL_Gan1_GLEAN_10002516;GL_Gan1_GLEAN_10001371;GL_Gan1_GLEAN_10005603;GL_Gan1_GLEAN_10009177;GL_Gan1_GLEAN_10009180;GL_Gan1_GLEAN_10000187;GL_Gan1_GLEAN_10000944;GL_Gan1_GLEAN_10001641;GL_Gan1_GLEAN_10006618;GL_Gan1_GLEAN_10009399;GL_Gan1_GLEAN_10004610;GL_Gan1_GLEAN_10002729;GL_Gan1_GLEAN_10000197;GL_Gan1_GLEAN_10000342;GL_Gan1_GLEAN_10000712;GL_Gan1_GLEAN_10002172;GL_Gan1_GLEAN_10002306;GL_Gan1_GLEAN_10002689;GL_Gan1_GLEAN_10003096;GL_Gan1_GLEAN_10003471;GL_Gan1_GLEAN_10003517;GL_Gan1_GLEAN_10003816;GL_Gan1_GLEAN_10003873;GL_Gan1_GLEAN_10004596;GL_Gan1_GLEAN_10004884;GL_Gan1_GLEAN_10007077;GL_Gan1_GLEAN_10007350;GL_Gan1_GLEAN_10008719;GL_Gan1_GLEAN_10008341;GL_Gan1_GLEAN_10002088;GL_Gan1_GLEAN_10006095;GL_Gan1_GLEAN_10008811;GL_Gan1_GLEAN_10005934;GL_Gan1_GLEAN_10007076;GL_Gan1_GLEAN_10008802;GL_Gan1_GLEAN_10000087;GL_Gan1_GLEAN_10000183;GL_Gan1_GLEAN_10000298;GL_Gan1_GLEAN_10000587;GL_Gan1_GLEAN_10000590;GL_Gan1_GLEAN_10000737;GL_Gan1_GLEAN_10000826;GL_Gan1_GLEAN_10001249;GL_Gan1_GLEAN_10001498;GL_Gan1_GLEAN_10001522;GL_Gan1_GLEAN_10001825;GL_Gan1_GLEAN_10001965;GL_Gan1_GLEAN_10002065;GL_Gan1_GLEAN_10002449;GL_Gan1_GLEAN_10002670;GL_Gan1_GLEAN_10002732;GL_Gan1_GLEAN_10002995;GL_Gan1_GLEAN_10003172;GL_Gan1_GLEAN_10003466;GL_Gan1_GLEAN_10003467;GL_Gan1_GLEAN_10003550;GL_Gan1_GLEAN_10003691;GL_Gan1_GLEAN_10004036;GL_Gan1_GLEAN_10004039;GL_Gan1_GLEAN_10004535;GL_Gan1_GLEAN_10004667;GL_Gan1_GLEAN_10005199;GL_Gan1_GLEAN_10005349;GL_Gan1_GLEAN_10005787;GL_Gan1_GLEAN_10005925;GL_Gan1_GLEAN_10006136;GL_Gan1_GLEAN_10006791;GL_Gan1_GLEAN_10006811;GL_Gan1_GLEAN_10006812;GL_Gan1_GLEAN_10006831;GL_Gan1_GLEAN_10006923;GL_Gan1_GLEAN_10007097;GL_Gan1_GLEAN_10007175;GL_Gan1_GLEAN_10007404;GL_Gan1_GLEAN_10007449;GL_Gan1_GLEAN_10007701;GL_Gan1_GLEAN_10007712;GL_Gan1_GLEAN_10007759;GL_Gan1_GLEAN_10007935;GL_Gan1_GLEAN_10008172;GL_Gan1_GLEAN_10008363;GL_Gan1_GLEAN_10008444;GL_Gan1_GLEAN_10008491;GL_Gan1_GLEAN_10008523;GL_Gan1_GLEAN_10008712;GL_Gan1_GLEAN_10009128;GL_Gan1_GLEAN_10009198;GL_Gan1_GLEAN_10009388;GL_Gan1_GLEAN_10001913;GL_Gan1_GLEAN_10004774;GL_Gan1_GLEAN_10005006;GL_Gan1_GLEAN_10004562;GL_Gan1_GLEAN_10002334;GL_Gan1_GLEAN_10002884;GL_Gan1_GLEAN_10007760;GL_Gan1_GLEAN_10008391;GL_Gan1_GLEAN_10000732;GL_Gan1_GLEAN_10001987;GL_Gan1_GLEAN_10009000;GL_Gan1_GLEAN_10009029;GL_Gan1_GLEAN_10000851;GL_Gan1_GLEAN_10001449;GL_Gan1_GLEAN_10002677;GL_Gan1_GLEAN_10002847;GL_Gan1_GLEAN_10005965;GL_Gan1_GLEAN_10003473 | |
|  | cellular process | 17 | GL_Gan1_GLEAN_10005568;GL_Gan1_GLEAN_10006784;GL_Gan1_GLEAN_10008511;GL_Gan1_GLEAN_10000357;GL_Gan1_GLEAN_10002658;GL_Gan1_GLEAN_10008911;GL_Gan1_GLEAN_10002483;GL_Gan1_GLEAN_10002062;GL_Gan1_GLEAN_10006095;GL_Gan1_GLEAN_10004632;GL_Gan1_GLEAN_10004832;GL_Gan1_GLEAN_10009160;GL_Gan1_GLEAN_10005979;GL_Gan1_GLEAN_10007718;GL_Gan1_GLEAN_10003397;GL_Gan1_GLEAN_10002677;GL_Gan1_GLEAN_10003473 | |
|  | electron carrier activity | 4 | GL_Gan1_GLEAN_10001965;GL_Gan1_GLEAN_10002732;GL_Gan1_GLEAN_10003523;GL_Gan1_GLEAN_10007982 | |
|  | establishment of localization | 4 | GL_Gan1_GLEAN_10007733;GL_Gan1_GLEAN_10005979;GL_Gan1_GLEAN_10007718;GL_Gan1_GLEAN_10003397 | |
|  | localization | 4 | GL_Gan1_GLEAN_10007733;GL_Gan1_GLEAN_10005979;GL_Gan1_GLEAN_10007718;GL_Gan1_GLEAN_10003397 | |
|  | metabolic process | 294 | GL_Gan1_GLEAN_10000842;GL_Gan1_GLEAN_10002143;GL_Gan1_GLEAN_10007413;GL_Gan1_GLEAN_10009271;GL_Gan1_GLEAN_10000874;GL_Gan1_GLEAN_10004216;GL_Gan1_GLEAN_10008512;GL_Gan1_GLEAN_10004832;GL_Gan1_GLEAN_10005427;GL_Gan1_GLEAN_10005568;GL_Gan1_GLEAN_10008352;GL_Gan1_GLEAN_10000011;GL_Gan1_GLEAN_10000281;GL_Gan1_GLEAN_10000339;GL_Gan1_GLEAN_10000420;GL_Gan1_GLEAN_10000483;GL_Gan1_GLEAN_10000560;GL_Gan1_GLEAN_10000875;GL_Gan1_GLEAN_10001002;GL_Gan1_GLEAN_10001125;GL_Gan1_GLEAN_10001363;GL_Gan1_GLEAN_10001381;GL_Gan1_GLEAN_10001386;GL_Gan1_GLEAN_10001523;GL_Gan1_GLEAN_10001684;GL_Gan1_GLEAN_10001758;GL_Gan1_GLEAN_10001901;GL_Gan1_GLEAN_10002027;GL_Gan1_GLEAN_10002416;GL_Gan1_GLEAN_10002529;GL_Gan1_GLEAN_10002588;GL_Gan1_GLEAN_10002688;GL_Gan1_GLEAN_10002730;GL_Gan1_GLEAN_10002881;GL_Gan1_GLEAN_10002913;GL_Gan1_GLEAN_10003120;GL_Gan1_GLEAN_10003193;GL_Gan1_GLEAN_10003223;GL_Gan1_GLEAN_10003348;GL_Gan1_GLEAN_10003523;GL_Gan1_GLEAN_10003623;GL_Gan1_GLEAN_10003693;GL_Gan1_GLEAN_10003767;GL_Gan1_GLEAN_10003768;GL_Gan1_GLEAN_10003844;GL_Gan1_GLEAN_10004022;GL_Gan1_GLEAN_10004105;GL_Gan1_GLEAN_10004267;GL_Gan1_GLEAN_10004323;GL_Gan1_GLEAN_10004376;GL_Gan1_GLEAN_10004402;GL_Gan1_GLEAN_10004431;GL_Gan1_GLEAN_10004441;GL_Gan1_GLEAN_10004607;GL_Gan1_GLEAN_10004626;GL_Gan1_GLEAN_10004632;GL_Gan1_GLEAN_10004811;GL_Gan1_GLEAN_10004834;GL_Gan1_GLEAN_10004906;GL_Gan1_GLEAN_10005127;GL_Gan1_GLEAN_10005379;GL_Gan1_GLEAN_10005418;GL_Gan1_GLEAN_10005434;GL_Gan1_GLEAN_10005521;GL_Gan1_GLEAN_10005559;GL_Gan1_GLEAN_10005576;GL_Gan1_GLEAN_10005628;GL_Gan1_GLEAN_10005806;GL_Gan1_GLEAN_10005899;GL_Gan1_GLEAN_10006125;GL_Gan1_GLEAN_10006192;GL_Gan1_GLEAN_10006204;GL_Gan1_GLEAN_10006272;GL_Gan1_GLEAN_10006566;GL_Gan1_GLEAN_10006575;GL_Gan1_GLEAN_10006780;GL_Gan1_GLEAN_10006885;GL_Gan1_GLEAN_10007061;GL_Gan1_GLEAN_10007096;GL_Gan1_GLEAN_10007233;GL_Gan1_GLEAN_10007241;GL_Gan1_GLEAN_10007353;GL_Gan1_GLEAN_10007420;GL_Gan1_GLEAN_10007769;GL_Gan1_GLEAN_10007812;GL_Gan1_GLEAN_10008146;GL_Gan1_GLEAN_10008151;GL_Gan1_GLEAN_10008175;GL_Gan1_GLEAN_10008180;GL_Gan1_GLEAN_10008261;GL_Gan1_GLEAN_10008328;GL_Gan1_GLEAN_10008336;GL_Gan1_GLEAN_10008704;GL_Gan1_GLEAN_10008808;GL_Gan1_GLEAN_10008864;GL_Gan1_GLEAN_10008907;GL_Gan1_GLEAN_10009139;GL_Gan1_GLEAN_10009160;GL_Gan1_GLEAN_10009169;GL_Gan1_GLEAN_10001978;GL_Gan1_GLEAN_10006142;GL_Gan1_GLEAN_10009378;GL_Gan1_GLEAN_10003940;GL_Gan1_GLEAN_10007460;GL_Gan1_GLEAN_10007905;GL_Gan1_GLEAN_10000555;GL_Gan1_GLEAN_10002235;GL_Gan1_GLEAN_10002372;GL_Gan1_GLEAN_10003006;GL_Gan1_GLEAN_10003089;GL_Gan1_GLEAN_10003252;GL_Gan1_GLEAN_10005842;GL_Gan1_GLEAN_10006181;GL_Gan1_GLEAN_10008959;GL_Gan1_GLEAN_10009203;GL_Gan1_GLEAN_10009371;GL_Gan1_GLEAN_10004833;GL_Gan1_GLEAN_10005414;GL_Gan1_GLEAN_10000111;GL_Gan1_GLEAN_10001569;GL_Gan1_GLEAN_10004217;GL_Gan1_GLEAN_10004644;GL_Gan1_GLEAN_10009097;GL_Gan1_GLEAN_10006784;GL_Gan1_GLEAN_10008511;GL_Gan1_GLEAN_10000181;GL_Gan1_GLEAN_10000898;GL_Gan1_GLEAN_10001131;GL_Gan1_GLEAN_10002233;GL_Gan1_GLEAN_10007730;GL_Gan1_GLEAN_10000161;GL_Gan1_GLEAN_10000815;GL_Gan1_GLEAN_10002758;GL_Gan1_GLEAN_10005639;GL_Gan1_GLEAN_10007234;GL_Gan1_GLEAN_10009012;GL_Gan1_GLEAN_10000357;GL_Gan1_GLEAN_10004270;GL_Gan1_GLEAN_10008272;GL_Gan1_GLEAN_10007371;GL_Gan1_GLEAN_10000001;GL_Gan1_GLEAN_10000719;GL_Gan1_GLEAN_10000855;GL_Gan1_GLEAN_10001487;GL_Gan1_GLEAN_10001543;GL_Gan1_GLEAN_10002241;GL_Gan1_GLEAN_10004407;GL_Gan1_GLEAN_10005564;GL_Gan1_GLEAN_10005895;GL_Gan1_GLEAN_10006585;GL_Gan1_GLEAN_10007763;GL_Gan1_GLEAN_10000198;GL_Gan1_GLEAN_10000723;GL_Gan1_GLEAN_10001234;GL_Gan1_GLEAN_10003073;GL_Gan1_GLEAN_10003265;GL_Gan1_GLEAN_10004524;GL_Gan1_GLEAN_10004544;GL_Gan1_GLEAN_10005419;GL_Gan1_GLEAN_10008068;GL_Gan1_GLEAN_10000814;GL_Gan1_GLEAN_10007895;GL_Gan1_GLEAN_10008741;GL_Gan1_GLEAN_10001243;GL_Gan1_GLEAN_10002284;GL_Gan1_GLEAN_10004070;GL_Gan1_GLEAN_10004380;GL_Gan1_GLEAN_10008464;GL_Gan1_GLEAN_10006767;GL_Gan1_GLEAN_10009036;GL_Gan1_GLEAN_10002658;GL_Gan1_GLEAN_10000849;GL_Gan1_GLEAN_10005384;GL_Gan1_GLEAN_10006617;GL_Gan1_GLEAN_10008994;GL_Gan1_GLEAN_10001061;GL_Gan1_GLEAN_10002050;GL_Gan1_GLEAN_10003837;GL_Gan1_GLEAN_10003979;GL_Gan1_GLEAN_10005226;GL_Gan1_GLEAN_10005821;GL_Gan1_GLEAN_10006243;GL_Gan1_GLEAN_10007752;GL_Gan1_GLEAN_10008567;GL_Gan1_GLEAN_10008675;GL_Gan1_GLEAN_10008911;GL_Gan1_GLEAN_10002483;GL_Gan1_GLEAN_10002516;GL_Gan1_GLEAN_10001371;GL_Gan1_GLEAN_10005603;GL_Gan1_GLEAN_10009177;GL_Gan1_GLEAN_10009180;GL_Gan1_GLEAN_10000187;GL_Gan1_GLEAN_10000944;GL_Gan1_GLEAN_10001641;GL_Gan1_GLEAN_10006618;GL_Gan1_GLEAN_10009399;GL_Gan1_GLEAN_10004610;GL_Gan1_GLEAN_10002729;GL_Gan1_GLEAN_10000197;GL_Gan1_GLEAN_10000342;GL_Gan1_GLEAN_10000712;GL_Gan1_GLEAN_10002172;GL_Gan1_GLEAN_10002306;GL_Gan1_GLEAN_10002689;GL_Gan1_GLEAN_10003096;GL_Gan1_GLEAN_10003471;GL_Gan1_GLEAN_10003517;GL_Gan1_GLEAN_10003816;GL_Gan1_GLEAN_10003873;GL_Gan1_GLEAN_10004596;GL_Gan1_GLEAN_10004884;GL_Gan1_GLEAN_10007077;GL_Gan1_GLEAN_10007350;GL_Gan1_GLEAN_10008719;GL_Gan1_GLEAN_10008341;GL_Gan1_GLEAN_10002062;GL_Gan1_GLEAN_10002088;GL_Gan1_GLEAN_10006095;GL_Gan1_GLEAN_10008811;GL_Gan1_GLEAN_10005934;GL_Gan1_GLEAN_10007076;GL_Gan1_GLEAN_10008802;GL_Gan1_GLEAN_10000087;GL_Gan1_GLEAN_10000183;GL_Gan1_GLEAN_10000298;GL_Gan1_GLEAN_10000587;GL_Gan1_GLEAN_10000590;GL_Gan1_GLEAN_10000737;GL_Gan1_GLEAN_10000826;GL_Gan1_GLEAN_10001249;GL_Gan1_GLEAN_10001498;GL_Gan1_GLEAN_10001522;GL_Gan1_GLEAN_10001825;GL_Gan1_GLEAN_10001965;GL_Gan1_GLEAN_10002065;GL_Gan1_GLEAN_10002449;GL_Gan1_GLEAN_10002670;GL_Gan1_GLEAN_10002732;GL_Gan1_GLEAN_10002995;GL_Gan1_GLEAN_10003172;GL_Gan1_GLEAN_10003466;GL_Gan1_GLEAN_10003467;GL_Gan1_GLEAN_10003550;GL_Gan1_GLEAN_10003691;GL_Gan1_GLEAN_10004036;GL_Gan1_GLEAN_10004039;GL_Gan1_GLEAN_10004535;GL_Gan1_GLEAN_10004667;GL_Gan1_GLEAN_10005199;GL_Gan1_GLEAN_10005349;GL_Gan1_GLEAN_10005787;GL_Gan1_GLEAN_10005925;GL_Gan1_GLEAN_10006136;GL_Gan1_GLEAN_10006791;GL_Gan1_GLEAN_10006811;GL_Gan1_GLEAN_10006812;GL_Gan1_GLEAN_10006831;GL_Gan1_GLEAN_10006923;GL_Gan1_GLEAN_10007097;GL_Gan1_GLEAN_10007175;GL_Gan1_GLEAN_10007404;GL_Gan1_GLEAN_10007449;GL_Gan1_GLEAN_10007701;GL_Gan1_GLEAN_10007712;GL_Gan1_GLEAN_10007759;GL_Gan1_GLEAN_10007935;GL_Gan1_GLEAN_10008172;GL_Gan1_GLEAN_10008363;GL_Gan1_GLEAN_10008444;GL_Gan1_GLEAN_10008491;GL_Gan1_GLEAN_10008523;GL_Gan1_GLEAN_10008712;GL_Gan1_GLEAN_10009128;GL_Gan1_GLEAN_10009198;GL_Gan1_GLEAN_10009388;GL_Gan1_GLEAN_10001913;GL_Gan1_GLEAN_10004774;GL_Gan1_GLEAN_10005006;GL_Gan1_GLEAN_10004562;GL_Gan1_GLEAN_10002334;GL_Gan1_GLEAN_10002884;GL_Gan1_GLEAN_10007760;GL_Gan1_GLEAN_10008391;GL_Gan1_GLEAN_10000732;GL_Gan1_GLEAN_10001987;GL_Gan1_GLEAN_10009000;GL_Gan1_GLEAN_10009029;GL_Gan1_GLEAN_10000851;GL_Gan1_GLEAN_10001449;GL_Gan1_GLEAN_10002677;GL_Gan1_GLEAN_10002847;GL_Gan1_GLEAN_10005965;GL_Gan1_GLEAN_10003473 | |
|  | molecular transducer activity | 2 | GL_Gan1_GLEAN_10005568;GL_Gan1_GLEAN_10002677 | |
|  | nucleic acid binding transcription factor activity | 1 | GL_Gan1_GLEAN_10002062 | |
|  | nutrient reservoir activity | 2 | GL_Gan1_GLEAN_10004477;GL_Gan1_GLEAN_10004540 | |
|  | protein binding transcription factor activity | 1 | GL_Gan1_GLEAN_10000357 | |
|  | receptor activity | 2 | GL_Gan1_GLEAN_10002677;GL_Gan1_GLEAN_10005568 | |
|  | regulation of biological process | 4 | GL_Gan1_GLEAN_10005568;GL_Gan1_GLEAN_10000357;GL_Gan1_GLEAN_10002062;GL_Gan1_GLEAN_10002677 | |
|  | response to stimulus | 2 | GL_Gan1_GLEAN_10005568;GL_Gan1_GLEAN_10002677 | |
|  | signaling | 2 | GL_Gan1_GLEAN_10005568;GL_Gan1_GLEAN_10002677 | |
|  | structural molecule activity | 1 | GL_Gan1_GLEAN_10005651 | |
|  | transporter activity | 4 | GL_Gan1_GLEAN_10007733;GL_Gan1_GLEAN_10005979;GL_Gan1_GLEAN_10007718;GL_Gan1_GLEAN_10003397 | |

**(F)** Annotation with KEGG for HGT

| First Level | Second Level | Gene of GL_Gan1.KEGG.catalog |
| --- | --- | --- |
| Cellular Processes | Cell Motility | 5 |
| Cellular Processes | Transport and Catabolism | 7 |
| Environmental Information Processing | Membrane Transport | 2 |
| Environmental Information Processing | Signal Transduction | 3 |
| Environmental Information Processing | Signaling Molecules and Interaction | 4 |
| Genetic Information Processing | Folding, Sorting and Degradation | 9 |
| Genetic Information Processing | Replication and Repair | 8 |
| Genetic Information Processing | Transcription | 12 |
| Genetic Information Processing | Translation | 9 |
| Human Diseases | Immune System Diseases | 2 |
| Human Diseases | Infectious Diseases | 3 |
| Human Diseases | Neurodegenerative Diseases | 9 |
| Metabolism | Amino Acid Metabolism | 104 |
| Metabolism | Biosynthesis of Other Secondary Metabolites | 14 |
| Metabolism | Carbohydrate Metabolism | 97 |
| Metabolism | Energy Metabolism | 25 |
| Metabolism | Enzyme Families | 10 |
| Metabolism | Glycan Biosynthesis and Metabolism | 9 |
| Metabolism | Lipid Metabolism | 60 |
| Metabolism | Metabolism of Cofactors and Vitamins | 23 |
| Metabolism | Metabolism of Other Amino Acids | 22 |
| Metabolism | Metabolism of Terpenoids and Polyketides | 24 |
| Metabolism | Nucleotide Metabolism | 9 |
| Metabolism | Xenobiotics Biodegradation and Metabolism | 137 |
| Organismal Systems | Circulatory System | 1 |
| Organismal Systems | Digestive System | 6 |
| Organismal Systems | Endocrine System | 2 |

**(G)** Annotation with COG for HGT

| function code | function | number | gene |
| --- | --- | --- | --- |
| A | RNA processing and modification | 1 | GL_Gan1_GLEAN_10005325; |
| C | Energy production and conversion | 44 | GL_Gan1_GLEAN_10003397;GL_Gan1_GLEAN_10008444;GL_Gan1_GLEAN_10001522;GL_Gan1_GLEAN_10002372;GL_Gan1_GLEAN_10007413;GL_Gan1_GLEAN_10000944;GL_Gan1_GLEAN_10006767;GL_Gan1_GLEAN_10006811;GL_Gan1_GLEAN_10006812;GL_Gan1_GLEAN_10000547;GL_Gan1_GLEAN_10000555;GL_Gan1_GLEAN_10009177;GL_Gan1_GLEAN_10009180;GL_Gan1_GLEAN_10009203;GL_Gan1_GLEAN_10009371;GL_Gan1_GLEAN_10001641;GL_Gan1_GLEAN_10009036;GL_Gan1_GLEAN_10006885;GL_Gan1_GLEAN_10008712;GL_Gan1_GLEAN_10005899;GL_Gan1_GLEAN_10005925;GL_Gan1_GLEAN_10000855;GL_Gan1_GLEAN_10001371;GL_Gan1_GLEAN_10002688;GL_Gan1_GLEAN_10002529;GL_Gan1_GLEAN_10003089;GL_Gan1_GLEAN_10007937;GL_Gan1_GLEAN_10007982;GL_Gan1_GLEAN_10001965;GL_Gan1_GLEAN_10007175;GL_Gan1_GLEAN_10002732;GL_Gan1_GLEAN_10003006;GL_Gan1_GLEAN_10004407;GL_Gan1_GLEAN_10006181;GL_Gan1_GLEAN_10005349;GL_Gan1_GLEAN_10005603;GL_Gan1_GLEAN_10007097;GL_Gan1_GLEAN_10003252;GL_Gan1_GLEAN_10004610;GL_Gan1_GLEAN_10002235;GL_Gan1_GLEAN_10007701;GL_Gan1_GLEAN_10007759;GL_Gan1_GLEAN_10006136;GL_Gan1_GLEAN_10006618; |
| D | Cell cycle control, cell division, chromosome partitioning | 2 | GL_Gan1_GLEAN_10005325;GL_Gan1_GLEAN_10005547; |
| E | Amino acid transport and metabolism | 48 | GL_Gan1_GLEAN_10000719;GL_Gan1_GLEAN_10008341;GL_Gan1_GLEAN_10008450;GL_Gan1_GLEAN_10005006;GL_Gan1_GLEAN_10002881;GL_Gan1_GLEAN_10002913;GL_Gan1_GLEAN_10004323;GL_Gan1_GLEAN_10008146;GL_Gan1_GLEAN_10008180;GL_Gan1_GLEAN_10008261;GL_Gan1_GLEAN_10000560;GL_Gan1_GLEAN_10009160;GL_Gan1_GLEAN_10008911;GL_Gan1_GLEAN_10008994;GL_Gan1_GLEAN_10009000;GL_Gan1_GLEAN_10009029;GL_Gan1_GLEAN_10005934;GL_Gan1_GLEAN_10005979;GL_Gan1_GLEAN_10000855;GL_Gan1_GLEAN_10000814;GL_Gan1_GLEAN_10000826;GL_Gan1_GLEAN_10001381;GL_Gan1_GLEAN_10002679;GL_Gan1_GLEAN_10006374;GL_Gan1_GLEAN_10003120;GL_Gan1_GLEAN_10007958;GL_Gan1_GLEAN_10001948;GL_Gan1_GLEAN_10007241;GL_Gan1_GLEAN_10002449;GL_Gan1_GLEAN_10001243;GL_Gan1_GLEAN_10005384;GL_Gan1_GLEAN_10005414;GL_Gan1_GLEAN_10005418;GL_Gan1_GLEAN_10000849;GL_Gan1_GLEAN_10007061;GL_Gan1_GLEAN_10007076;GL_Gan1_GLEAN_10000183;GL_Gan1_GLEAN_10002027;GL_Gan1_GLEAN_10003523;GL_Gan1_GLEAN_10007665;GL_Gan1_GLEAN_10007718;GL_Gan1_GLEAN_10007733;GL_Gan1_GLEAN_10007763;GL_Gan1_GLEAN_10005462;GL_Gan1_GLEAN_10005521;GL_Gan1_GLEAN_10005564;GL_Gan1_GLEAN_10003623;GL_Gan1_GLEAN_10006617; |
| F | Nucleotide transport and metabolism | 12 | GL_Gan1_GLEAN_10008272;GL_Gan1_GLEAN_10003816;GL_Gan1_GLEAN_10008907;GL_Gan1_GLEAN_10008802;GL_Gan1_GLEAN_10004774;GL_Gan1_GLEAN_10004884;GL_Gan1_GLEAN_10005434;GL_Gan1_GLEAN_10005639;GL_Gan1_GLEAN_10000842;GL_Gan1_GLEAN_10002143;GL_Gan1_GLEAN_10003471;GL_Gan1_GLEAN_10006095; |
| G | Carbohydrate transport and metabolism | 43 | GL_Gan1_GLEAN_10008336;GL_Gan1_GLEAN_10004477;GL_Gan1_GLEAN_10004540;GL_Gan1_GLEAN_10004544;GL_Gan1_GLEAN_10003768;GL_Gan1_GLEAN_10007353;GL_Gan1_GLEAN_10007420;GL_Gan1_GLEAN_10001754;GL_Gan1_GLEAN_10008068;GL_Gan1_GLEAN_10008151;GL_Gan1_GLEAN_10001684;GL_Gan1_GLEAN_10000851;GL_Gan1_GLEAN_10000875;GL_Gan1_GLEAN_10000187;GL_Gan1_GLEAN_10000198;GL_Gan1_GLEAN_10003073;GL_Gan1_GLEAN_10007808;GL_Gan1_GLEAN_10001193;GL_Gan1_GLEAN_10001234;GL_Gan1_GLEAN_10007233;GL_Gan1_GLEAN_10007241;GL_Gan1_GLEAN_10002658;GL_Gan1_GLEAN_10000723;GL_Gan1_GLEAN_10004906;GL_Gan1_GLEAN_10004105;GL_Gan1_GLEAN_10004402;GL_Gan1_GLEAN_10004431;GL_Gan1_GLEAN_10006159;GL_Gan1_GLEAN_10006272;GL_Gan1_GLEAN_10005419;GL_Gan1_GLEAN_10005576;GL_Gan1_GLEAN_10005628;GL_Gan1_GLEAN_10007061;GL_Gan1_GLEAN_10004607;GL_Gan1_GLEAN_10004626;GL_Gan1_GLEAN_10003523;GL_Gan1_GLEAN_10006125;GL_Gan1_GLEAN_10002588;GL_Gan1_GLEAN_10005559;GL_Gan1_GLEAN_10000339;GL_Gan1_GLEAN_10006566;GL_Gan1_GLEAN_10006575;GL_Gan1_GLEAN_10001002; |
| H | Coenzyme transport and metabolism | 20 | GL_Gan1_GLEAN_10000719;GL_Gan1_GLEAN_10001522;GL_Gan1_GLEAN_10003767;GL_Gan1_GLEAN_10009271;GL_Gan1_GLEAN_10003223;GL_Gan1_GLEAN_10008709;GL_Gan1_GLEAN_10008712;GL_Gan1_GLEAN_10001381;GL_Gan1_GLEAN_10001386;GL_Gan1_GLEAN_10007812;GL_Gan1_GLEAN_10002729;GL_Gan1_GLEAN_10002730;GL_Gan1_GLEAN_10004376;GL_Gan1_GLEAN_10004407;GL_Gan1_GLEAN_10004833;GL_Gan1_GLEAN_10006192;GL_Gan1_GLEAN_10005349;GL_Gan1_GLEAN_10007759;GL_Gan1_GLEAN_10006136;GL_Gan1_GLEAN_10005564; |
| I | Lipid transport and metabolism | 39 | GL_Gan1_GLEAN_10000587;GL_Gan1_GLEAN_10000590;GL_Gan1_GLEAN_10004535;GL_Gan1_GLEAN_10004985;GL_Gan1_GLEAN_10001543;GL_Gan1_GLEAN_10007371;GL_Gan1_GLEAN_10007404;GL_Gan1_GLEAN_10007460;GL_Gan1_GLEAN_10006791;GL_Gan1_GLEAN_10009198;GL_Gan1_GLEAN_10009388;GL_Gan1_GLEAN_10003193;GL_Gan1_GLEAN_10009128;GL_Gan1_GLEAN_10006831;GL_Gan1_GLEAN_10006923;GL_Gan1_GLEAN_10008808;GL_Gan1_GLEAN_10005895;GL_Gan1_GLEAN_10002670;GL_Gan1_GLEAN_10002516;GL_Gan1_GLEAN_10003691;GL_Gan1_GLEAN_10007894;GL_Gan1_GLEAN_10007905;GL_Gan1_GLEAN_10007935;GL_Gan1_GLEAN_10000087;GL_Gan1_GLEAN_10000737;GL_Gan1_GLEAN_10004667;GL_Gan1_GLEAN_10002306;GL_Gan1_GLEAN_10004036;GL_Gan1_GLEAN_10004039;GL_Gan1_GLEAN_10001487;GL_Gan1_GLEAN_10001498;GL_Gan1_GLEAN_10000298;GL_Gan1_GLEAN_10005074;GL_Gan1_GLEAN_10003940;GL_Gan1_GLEAN_10002172;GL_Gan1_GLEAN_10003467;GL_Gan1_GLEAN_10005787;GL_Gan1_GLEAN_10003550;GL_Gan1_GLEAN_10006585; |
| J | Translation, ribosomal structure and biogenesis | 12 | GL_Gan1_GLEAN_10008464;GL_Gan1_GLEAN_10009399;GL_Gan1_GLEAN_10009000;GL_Gan1_GLEAN_10009029;GL_Gan1_GLEAN_10008567;GL_Gan1_GLEAN_10002483;GL_Gan1_GLEAN_10001061;GL_Gan1_GLEAN_10004070;GL_Gan1_GLEAN_10004380;GL_Gan1_GLEAN_10003979;GL_Gan1_GLEAN_10002050;GL_Gan1_GLEAN_10005821; |
| K | Transcription | 8 | GL_Gan1_GLEAN_10008894;GL_Gan1_GLEAN_10008675;GL_Gan1_GLEAN_10003837;GL_Gan1_GLEAN_10001055;GL_Gan1_GLEAN_10006243;GL_Gan1_GLEAN_10005325;GL_Gan1_GLEAN_10005226;GL_Gan1_GLEAN_10007752; |
| L | Replication, recombination and repair | 6 | GL_Gan1_GLEAN_10003096;GL_Gan1_GLEAN_10001055;GL_Gan1_GLEAN_10000001;GL_Gan1_GLEAN_10003473;GL_Gan1_GLEAN_10003517;GL_Gan1_GLEAN_10007730; |
| M | Cell wall/membrane/envelope biogenesis | 14 | GL_Gan1_GLEAN_10008352;GL_Gan1_GLEAN_10008391;GL_Gan1_GLEAN_10003768;GL_Gan1_GLEAN_10007420;GL_Gan1_GLEAN_10009139;GL_Gan1_GLEAN_10001919;GL_Gan1_GLEAN_10003693;GL_Gan1_GLEAN_10001193;GL_Gan1_GLEAN_10006159;GL_Gan1_GLEAN_10006272;GL_Gan1_GLEAN_10002241;GL_Gan1_GLEAN_10000420;GL_Gan1_GLEAN_10001125;GL_Gan1_GLEAN_10001002; |
| O | Posttranslational modification, protein turnover, chaperones | 20 | GL_Gan1_GLEAN_10008511;GL_Gan1_GLEAN_10004441;GL_Gan1_GLEAN_10003824;GL_Gan1_GLEAN_10004270;GL_Gan1_GLEAN_10007437;GL_Gan1_GLEAN_10006784;GL_Gan1_GLEAN_10001777;GL_Gan1_GLEAN_10008690;GL_Gan1_GLEAN_10001913;GL_Gan1_GLEAN_10001932;GL_Gan1_GLEAN_10000011;GL_Gan1_GLEAN_10001235;GL_Gan1_GLEAN_10002597;GL_Gan1_GLEAN_10000732;GL_Gan1_GLEAN_10004096;GL_Gan1_GLEAN_10004406;GL_Gan1_GLEAN_10006204;GL_Gan1_GLEAN_10005339;GL_Gan1_GLEAN_10007083;GL_Gan1_GLEAN_10000483; |
| P | Inorganic ion transport and metabolism | 16 | GL_Gan1_GLEAN_10001187;GL_Gan1_GLEAN_10002884;GL_Gan1_GLEAN_10006668;GL_Gan1_GLEAN_10009169;GL_Gan1_GLEAN_10008704;GL_Gan1_GLEAN_10005965;GL_Gan1_GLEAN_10001449;GL_Gan1_GLEAN_10002334;GL_Gan1_GLEAN_10003320;GL_Gan1_GLEAN_10004846;GL_Gan1_GLEAN_10006185;GL_Gan1_GLEAN_10002847;GL_Gan1_GLEAN_10005367;GL_Gan1_GLEAN_10000071;GL_Gan1_GLEAN_10007760;GL_Gan1_GLEAN_10005806; |
| Q | Secondary metabolites biosynthesis, transport and catabolism | 49 | GL_Gan1_GLEAN_10000587;GL_Gan1_GLEAN_10000590;GL_Gan1_GLEAN_10008328;GL_Gan1_GLEAN_10004535;GL_Gan1_GLEAN_10004985;GL_Gan1_GLEAN_10001523;GL_Gan1_GLEAN_10004267;GL_Gan1_GLEAN_10004319;GL_Gan1_GLEAN_10007404;GL_Gan1_GLEAN_10006780;GL_Gan1_GLEAN_10006791;GL_Gan1_GLEAN_10001758;GL_Gan1_GLEAN_10008175;GL_Gan1_GLEAN_10009198;GL_Gan1_GLEAN_10009388;GL_Gan1_GLEAN_10003172;GL_Gan1_GLEAN_10003193;GL_Gan1_GLEAN_10003201;GL_Gan1_GLEAN_10009128;GL_Gan1_GLEAN_10006831;GL_Gan1_GLEAN_10006923;GL_Gan1_GLEAN_10006982;GL_Gan1_GLEAN_10008808;GL_Gan1_GLEAN_10000874;GL_Gan1_GLEAN_10003879;GL_Gan1_GLEAN_10002670;GL_Gan1_GLEAN_10004216;GL_Gan1_GLEAN_10003691;GL_Gan1_GLEAN_10007894;GL_Gan1_GLEAN_10007935;GL_Gan1_GLEAN_10000087;GL_Gan1_GLEAN_10000737;GL_Gan1_GLEAN_10004667;GL_Gan1_GLEAN_10004036;GL_Gan1_GLEAN_10004039;GL_Gan1_GLEAN_10001498;GL_Gan1_GLEAN_10003062;GL_Gan1_GLEAN_10000298;GL_Gan1_GLEAN_10004811;GL_Gan1_GLEAN_10004832;GL_Gan1_GLEAN_10005379;GL_Gan1_GLEAN_10005418;GL_Gan1_GLEAN_10004632;GL_Gan1_GLEAN_10003467;GL_Gan1_GLEAN_10000161;GL_Gan1_GLEAN_10007714;GL_Gan1_GLEAN_10005787;GL_Gan1_GLEAN_10003550;GL_Gan1_GLEAN_10006520; |
| R | General function prediction only | 124 | GL_Gan1_GLEAN_10000587;GL_Gan1_GLEAN_10000590;GL_Gan1_GLEAN_10000712;GL_Gan1_GLEAN_10001363;GL_Gan1_GLEAN_10008491;GL_Gan1_GLEAN_10008512;GL_Gan1_GLEAN_10004477;GL_Gan1_GLEAN_10004535;GL_Gan1_GLEAN_10004540;GL_Gan1_GLEAN_10004985;GL_Gan1_GLEAN_10003782;GL_Gan1_GLEAN_10003820;GL_Gan1_GLEAN_10007350;GL_Gan1_GLEAN_10007404;GL_Gan1_GLEAN_10006658;GL_Gan1_GLEAN_10006791;GL_Gan1_GLEAN_10001750;GL_Gan1_GLEAN_10008044;GL_Gan1_GLEAN_10008052;GL_Gan1_GLEAN_10000553;GL_Gan1_GLEAN_10000555;GL_Gan1_GLEAN_10001825;GL_Gan1_GLEAN_10009166;GL_Gan1_GLEAN_10009198;GL_Gan1_GLEAN_10009203;GL_Gan1_GLEAN_10009259;GL_Gan1_GLEAN_10009297;GL_Gan1_GLEAN_10009378;GL_Gan1_GLEAN_10009388;GL_Gan1_GLEAN_10003201;GL_Gan1_GLEAN_10003209;GL_Gan1_GLEAN_10008920;GL_Gan1_GLEAN_10008959;GL_Gan1_GLEAN_10009012;GL_Gan1_GLEAN_10009128;GL_Gan1_GLEAN_10006831;GL_Gan1_GLEAN_10006923;GL_Gan1_GLEAN_10008523;GL_Gan1_GLEAN_10008565;GL_Gan1_GLEAN_10008645;GL_Gan1_GLEAN_10008675;GL_Gan1_GLEAN_10008719;GL_Gan1_GLEAN_10008741;GL_Gan1_GLEAN_10005877;GL_Gan1_GLEAN_10005925;GL_Gan1_GLEAN_10000874;GL_Gan1_GLEAN_10003837;GL_Gan1_GLEAN_10003844;GL_Gan1_GLEAN_10003873;GL_Gan1_GLEAN_10002065;GL_Gan1_GLEAN_10002096;GL_Gan1_GLEAN_10000815;GL_Gan1_GLEAN_10000197;GL_Gan1_GLEAN_10002670;GL_Gan1_GLEAN_10004216;GL_Gan1_GLEAN_10003089;GL_Gan1_GLEAN_10003096;GL_Gan1_GLEAN_10003691;GL_Gan1_GLEAN_10003693;GL_Gan1_GLEAN_10007894;GL_Gan1_GLEAN_10007895;GL_Gan1_GLEAN_10007935;GL_Gan1_GLEAN_10000087;GL_Gan1_GLEAN_10001978;GL_Gan1_GLEAN_10001987;GL_Gan1_GLEAN_10007175;GL_Gan1_GLEAN_10007234;GL_Gan1_GLEAN_10002633;GL_Gan1_GLEAN_10001601;GL_Gan1_GLEAN_10000737;GL_Gan1_GLEAN_10000751;GL_Gan1_GLEAN_10004667;GL_Gan1_GLEAN_10002416;GL_Gan1_GLEAN_10002458;GL_Gan1_GLEAN_10004036;GL_Gan1_GLEAN_10004039;GL_Gan1_GLEAN_10002758;GL_Gan1_GLEAN_10001498;GL_Gan1_GLEAN_10003006;GL_Gan1_GLEAN_10002233;GL_Gan1_GLEAN_10004407;GL_Gan1_GLEAN_10001249;GL_Gan1_GLEAN_10001055;GL_Gan1_GLEAN_10000298;GL_Gan1_GLEAN_10004834;GL_Gan1_GLEAN_10006181;GL_Gan1_GLEAN_10006243;GL_Gan1_GLEAN_10006261;GL_Gan1_GLEAN_10005427;GL_Gan1_GLEAN_10005157;GL_Gan1_GLEAN_10005171;GL_Gan1_GLEAN_10004022;GL_Gan1_GLEAN_10007060;GL_Gan1_GLEAN_10000181;GL_Gan1_GLEAN_10003240;GL_Gan1_GLEAN_10003252;GL_Gan1_GLEAN_10001131;GL_Gan1_GLEAN_10005199;GL_Gan1_GLEAN_10005226;GL_Gan1_GLEAN_10005252;GL_Gan1_GLEAN_10004596;GL_Gan1_GLEAN_10003466;GL_Gan1_GLEAN_10003467;GL_Gan1_GLEAN_10003517;GL_Gan1_GLEAN_10002235;GL_Gan1_GLEAN_10002261;GL_Gan1_GLEAN_10007712;GL_Gan1_GLEAN_10007714;GL_Gan1_GLEAN_10007733;GL_Gan1_GLEAN_10007752;GL_Gan1_GLEAN_10007769;GL_Gan1_GLEAN_10006049;GL_Gan1_GLEAN_10006142;GL_Gan1_GLEAN_10000898;GL_Gan1_GLEAN_10002995;GL_Gan1_GLEAN_10005787;GL_Gan1_GLEAN_10005842;GL_Gan1_GLEAN_10003550;GL_Gan1_GLEAN_10003576;GL_Gan1_GLEAN_10003591;GL_Gan1_GLEAN_10003594;GL_Gan1_GLEAN_10000420;GL_Gan1_GLEAN_10006492;GL_Gan1_GLEAN_10001125; |
| S | Function unknown | 28 | GL_Gan1_GLEAN_10008264;GL_Gan1_GLEAN_10008363;GL_Gan1_GLEAN_10001527;GL_Gan1_GLEAN_10007449;GL_Gan1_GLEAN_10006802;GL_Gan1_GLEAN_10008172;GL_Gan1_GLEAN_10009097;GL_Gan1_GLEAN_10009137;GL_Gan1_GLEAN_10009158;GL_Gan1_GLEAN_10002066;GL_Gan1_GLEAN_10001370;GL_Gan1_GLEAN_10004181;GL_Gan1_GLEAN_10004217;GL_Gan1_GLEAN_10000111;GL_Gan1_GLEAN_10001984;GL_Gan1_GLEAN_10001569;GL_Gan1_GLEAN_10002314;GL_Gan1_GLEAN_10002317;GL_Gan1_GLEAN_10002739;GL_Gan1_GLEAN_10004383;GL_Gan1_GLEAN_10005651;GL_Gan1_GLEAN_10005110;GL_Gan1_GLEAN_10002134;GL_Gan1_GLEAN_10004644;GL_Gan1_GLEAN_10002284;GL_Gan1_GLEAN_10007632;GL_Gan1_GLEAN_10002924;GL_Gan1_GLEAN_10006521; |
| T | Signal transduction mechanisms | 4 | GL_Gan1_GLEAN_10009001;GL_Gan1_GLEAN_10002677;GL_Gan1_GLEAN_10001055;GL_Gan1_GLEAN_10005568; |
| U | Intracellular trafficking, secretion, and vesicular transport | 1 | GL_Gan1_GLEAN_10005267; |
| V | Defense mechanisms | 3 | GL_Gan1_GLEAN_10007346;GL_Gan1_GLEAN_10008811;GL_Gan1_GLEAN_10007052; |
