# Supplementary material for: New insights into the evolution of host specificity of three Penicillium species and the pathogenicity of P. Italicum involving the infection of Valencia orange (Citrus sinensis)
Source: Virulence. 2020 Jun 11;11(1):748–68. doi: 10.1080/21505594.2020.1773038 (PMC7549954; doi:10.1080/21505594.2020.1773038)
Supplement: Supplemental Material [file KVIR_A_1773038_SM2584.zip › Dataset S2.docx]

**Dataset** **S2** Common (A) or specific (B) PHI gene categories and gene numbers among three Penicillin species

**(A)** Common PHI gene categories and numbers

| subgroup | CMP1 | Gan1 | MD8 | Pd1 | PHI1 | PHI26 |
| --- | --- | --- | --- | --- | --- | --- |
| PHI:100 | 3 | 2 | 3 | 3 | 3 | 5 |
| PHI:1005 | 1 | 2 | 2 | 2 | 2 | 2 |
| PHI:1006 | 2 | 1 | 2 | 2 | 1 | 1 |
| PHI:1008 | 2 | 1 | 1 | 1 | 2 | 1 |
| PHI:101 | 3 | 4 | 3 | 3 | 4 | 3 |
| PHI:1018 | 19 | 15 | 19 | 19 | 14 | 15 |
| PHI:1021 | 38 | 30 | 41 | 42 | 31 | 18 |
| PHI:1022 | 12 | 10 | 11 | 12 | 8 | 7 |
| PHI:1023 | 2 | 2 | 2 | 2 | 1 | 1 |
| PHI:1025 | 1 | 1 | 1 | 1 | 1 | 1 |
| PHI:1026 | 2 | 2 | 2 | 2 | 2 | 2 |
| PHI:1028 | 3 | 1 | 3 | 3 | 1 | 1 |
| PHI:1030 | 4 | 3 | 4 | 4 | 4 | 4 |
| PHI:1032 | 1 | 1 | 1 | 1 | 1 | 1 |
| PHI:1034 | 1 | 1 | 1 | 1 | 1 | 1 |
| PHI:1037 | 1 | 2 | 1 | 1 | 1 | 1 |
| PHI:1039 | 1 | 2 | 1 | 1 | 1 | 1 |
| PHI:104 | 1 | 1 | 1 | 1 | 1 | 1 |
| PHI:1046 | 19 | 13 | 21 | 19 | 12 | 8 |
| PHI:1047 | 10 | 5 | 10 | 10 | 6 | 3 |
| PHI:1048 | 2 | 1 | 2 | 2 | 1 | 1 |
| PHI:105 | 1 | 1 | 1 | 2 | 1 | 1 |
| PHI:1050 | 4 | 6 | 4 | 4 | 2 | 3 |
| PHI:1051 | 19 | 8 | 21 | 21 | 10 | 9 |
| PHI:1052 | 1 | 2 | 1 | 1 | 2 | 2 |
| PHI:1056 | 1 | 2 | 1 | 1 | 1 | 2 |
| PHI:1057 | 2 | 2 | 2 | 2 | 3 | 2 |
| PHI:1058 | 4 | 5 | 4 | 4 | 6 | 3 |
| PHI:106 | 3 | 2 | 5 | 5 | 3 | 2 |
| PHI:1063 | 2 | 2 | 2 | 2 | 2 | 2 |
| PHI:1064 | 1 | 1 | 1 | 1 | 1 | 1 |
| PHI:1070 | 2 | 2 | 2 | 2 | 2 | 2 |
| PHI:1071 | 6 | 5 | 6 | 6 | 5 | 5 |
| PHI:1074 | 1 | 1 | 1 | 1 | 1 | 1 |
| PHI:1075 | 4 | 3 | 4 | 4 | 4 | 2 |
| PHI:1082 | 3 | 2 | 3 | 3 | 2 | 2 |
| PHI:1083 | 5 | 6 | 5 | 5 | 4 | 4 |
| PHI:1085 | 7 | 4 | 7 | 7 | 4 | 5 |
| PHI:109 | 4 | 3 | 3 | 3 | 3 | 2 |
| PHI:1100 | 35 | 26 | 37 | 35 | 26 | 19 |
| PHI:112 | 6 | 2 | 8 | 7 | 2 | 5 |
| PHI:113 | 2 | 3 | 2 | 2 | 3 | 2 |
| PHI:115 | 6 | 4 | 6 | 6 | 4 | 6 |
| PHI:12 | 12 | 7 | 12 | 12 | 7 | 9 |
| PHI:121 | 1 | 1 | 1 | 1 | 1 | 1 |
| PHI:124 | 1 | 1 | 1 | 1 | 1 | 1 |
| PHI:133 | 14 | 8 | 13 | 13 | 10 | 9 |
| PHI:134 | 2 | 1 | 3 | 3 | 1 | 1 |
| PHI:135 | 1 | 1 | 1 | 1 | 1 | 1 |
| PHI:139 | 5 | 5 | 5 | 5 | 4 | 4 |
| PHI:141 | 3 | 2 | 3 | 3 | 3 | 1 |
| PHI:143 | 6 | 4 | 5 | 5 | 5 | 4 |
| PHI:144 | 11 | 10 | 12 | 12 | 10 | 5 |
| PHI:146 | 1 | 1 | 1 | 1 | 1 | 1 |
| PHI:149 | 1 | 4 | 2 | 2 | 3 | 1 |
| PHI:153 | 6 | 6 | 6 | 6 | 8 | 3 |
| PHI:157 | 6 | 3 | 6 | 6 | 4 | 3 |
| PHI:158 | 3 | 3 | 3 | 3 | 3 | 3 |
| PHI:159 | 2 | 2 | 2 | 2 | 2 | 2 |
| PHI:160 | 13 | 3 | 13 | 15 | 3 | 6 |
| PHI:165 | 1 | 2 | 1 | 1 | 2 | 1 |
| PHI:167 | 5 | 6 | 5 | 6 | 6 | 4 |
| PHI:169 | 20 | 16 | 21 | 23 | 15 | 10 |
| PHI:172 | 3 | 4 | 3 | 3 | 4 | 5 |
| PHI:174 | 1 | 1 | 1 | 1 | 1 | 1 |
| PHI:177 | 3 | 2 | 3 | 3 | 2 | 2 |
| PHI:178 | 4 | 4 | 4 | 4 | 4 | 4 |
| PHI:180 | 1 | 1 | 1 | 1 | 1 | 1 |
| PHI:182 | 1 | 1 | 1 | 1 | 1 | 1 |
| PHI:184 | 2 | 2 | 2 | 2 | 2 | 2 |
| PHI:186 | 1 | 1 | 1 | 1 | 1 | 1 |
| PHI:187 | 8 | 6 | 8 | 8 | 6 | 5 |
| PHI:188 | 6 | 8 | 6 | 6 | 9 | 10 |
| PHI:189 | 1 | 1 | 1 | 1 | 1 | 1 |
| PHI:19 | 2 | 2 | 2 | 2 | 2 | 2 |
| PHI:190 | 1 | 1 | 1 | 1 | 1 | 1 |
| PHI:191 | 8 | 5 | 8 | 7 | 6 | 5 |
| PHI:194 | 1 | 1 | 1 | 1 | 2 | 1 |
| PHI:195 | 4 | 3 | 4 | 4 | 3 | 4 |
| PHI:197 | 3 | 1 | 3 | 3 | 2 | 1 |
| PHI:199 | 4 | 3 | 4 | 5 | 3 | 2 |
| PHI:200 | 2 | 1 | 2 | 2 | 1 | 1 |
| PHI:202 | 7 | 5 | 8 | 7 | 4 | 7 |
| PHI:206 | 1 | 1 | 1 | 1 | 1 | 1 |
| PHI:207 | 1 | 1 | 1 | 1 | 1 | 1 |
| PHI:208 | 5 | 4 | 5 | 5 | 4 | 5 |
| PHI:211 | 47 | 53 | 52 | 52 | 48 | 45 |
| PHI:213 | 4 | 6 | 5 | 5 | 6 | 5 |
| PHI:215 | 1 | 1 | 1 | 1 | 1 | 1 |
| PHI:216 | 1 | 1 | 1 | 1 | 1 | 1 |
| PHI:218 | 1 | 1 | 1 | 1 | 1 | 1 |
| PHI:219 | 4 | 4 | 4 | 4 | 4 | 4 |
| PHI:22 | 4 | 3 | 4 | 4 | 3 | 4 |
| PHI:220 | 2 | 2 | 2 | 2 | 3 | 2 |
| PHI:222 | 4 | 3 | 4 | 4 | 3 | 3 |
| PHI:226 | 15 | 15 | 15 | 15 | 15 | 17 |
| PHI:228 | 1 | 1 | 1 | 1 | 1 | 1 |
| PHI:231 | 2 | 2 | 2 | 2 | 2 | 2 |
| PHI:233 | 16 | 11 | 19 | 18 | 10 | 2 |
| PHI:235 | 1 | 1 | 1 | 1 | 1 | 1 |
| PHI:236 | 1 | 1 | 1 | 1 | 1 | 1 |
| PHI:24 | 2 | 1 | 6 | 5 | 1 | 2 |
| PHI:240 | 1 | 2 | 1 | 1 | 1 | 2 |
| PHI:242 | 3 | 3 | 3 | 3 | 4 | 3 |
| PHI:243 | 8 | 3 | 8 | 8 | 3 | 2 |
| PHI:244 | 5 | 3 | 5 | 5 | 3 | 3 |
| PHI:246 | 3 | 5 | 3 | 3 | 5 | 2 |
| PHI:249 | 5 | 4 | 5 | 5 | 4 | 3 |
| PHI:250 | 1 | 1 | 1 | 1 | 1 | 1 |
| PHI:251 | 1 | 1 | 1 | 1 | 1 | 1 |
| PHI:252 | 3 | 4 | 4 | 4 | 5 | 4 |
| PHI:253 | 2 | 2 | 2 | 2 | 2 | 2 |
| PHI:254 | 3 | 4 | 3 | 3 | 4 | 4 |
| PHI:255 | 12 | 12 | 11 | 11 | 12 | 3 |
| PHI:259 | 3 | 3 | 3 | 3 | 3 | 3 |
| PHI:26 | 19 | 10 | 21 | 20 | 11 | 4 |
| PHI:260 | 2 | 2 | 2 | 2 | 2 | 2 |
| PHI:261 | 4 | 2 | 4 | 4 | 2 | 4 |
| PHI:262 | 2 | 2 | 2 | 2 | 2 | 2 |
| PHI:265 | 1 | 2 | 2 | 2 | 2 | 2 |
| PHI:266 | 1 | 1 | 1 | 1 | 1 | 1 |
| PHI:267 | 28 | 18 | 27 | 26 | 18 | 13 |
| PHI:268 | 4 | 6 | 4 | 4 | 2 | 3 |
| PHI:269 | 1 | 1 | 1 | 1 | 2 | 2 |
| PHI:27 | 1 | 1 | 1 | 1 | 1 | 1 |
| PHI:270 | 4 | 3 | 4 | 4 | 3 | 4 |
| PHI:273 | 1 | 1 | 1 | 1 | 1 | 1 |
| PHI:274 | 3 | 3 | 3 | 3 | 3 | 1 |
| PHI:275 | 1 | 2 | 1 | 1 | 2 | 1 |
| PHI:277 | 1 | 2 | 2 | 2 | 2 | 3 |
| PHI:279 | 1 | 1 | 1 | 1 | 1 | 1 |
| PHI:280 | 4 | 5 | 4 | 4 | 4 | 5 |
| PHI:281 | 4 | 4 | 4 | 4 | 4 | 4 |
| PHI:282 | 1 | 1 | 1 | 1 | 1 | 2 |
| PHI:283 | 2 | 2 | 2 | 2 | 2 | 2 |
| PHI:285 | 2 | 1 | 2 | 2 | 2 | 2 |
| PHI:286 | 3 | 3 | 3 | 3 | 3 | 3 |
| PHI:287 | 1 | 1 | 1 | 1 | 1 | 1 |
| PHI:290 | 2 | 1 | 2 | 2 | 1 | 2 |
| PHI:292 | 1 | 2 | 1 | 1 | 1 | 1 |
| PHI:294 | 4 | 3 | 4 | 4 | 3 | 2 |
| PHI:296 | 3 | 4 | 2 | 3 | 3 | 2 |
| PHI:3 | 2 | 2 | 2 | 2 | 2 | 1 |
| PHI:300 | 3 | 2 | 3 | 3 | 2 | 1 |
| PHI:301 | 3 | 2 | 3 | 3 | 2 | 3 |
| PHI:303 | 1 | 1 | 1 | 1 | 1 | 1 |
| PHI:304 | 1 | 1 | 1 | 1 | 1 | 1 |
| PHI:307 | 1 | 1 | 1 | 1 | 1 | 1 |
| PHI:31 | 1 | 1 | 1 | 1 | 1 | 1 |
| PHI:310 | 3 | 3 | 3 | 3 | 2 | 2 |
| PHI:316 | 4 | 7 | 4 | 4 | 5 | 6 |
| PHI:317 | 1 | 1 | 1 | 1 | 1 | 1 |
| PHI:319 | 7 | 7 | 8 | 8 | 8 | 7 |
| PHI:320 | 4 | 6 | 6 | 6 | 5 | 4 |
| PHI:323 | 3 | 3 | 3 | 3 | 3 | 2 |
| PHI:325 | 14 | 11 | 19 | 16 | 11 | 11 |
| PHI:326 | 2 | 2 | 2 | 2 | 2 | 2 |
| PHI:33 | 4 | 3 | 4 | 4 | 3 | 3 |
| PHI:331 | 5 | 5 | 5 | 5 | 4 | 5 |
| PHI:334 | 6 | 6 | 6 | 6 | 7 | 8 |
| PHI:335 | 1 | 1 | 1 | 1 | 1 | 1 |
| PHI:336 | 1 | 1 | 1 | 1 | 1 | 1 |
| PHI:337 | 2 | 2 | 2 | 2 | 2 | 2 |
| PHI:338 | 2 | 2 | 2 | 2 | 2 | 2 |
| PHI:339 | 13 | 15 | 15 | 15 | 15 | 13 |
| PHI:340 | 1 | 1 | 1 | 1 | 1 | 1 |
| PHI:341 | 4 | 4 | 4 | 4 | 4 | 3 |
| PHI:342 | 3 | 6 | 2 | 2 | 8 | 4 |
| PHI:343 | 1 | 1 | 1 | 1 | 1 | 1 |
| PHI:344 | 3 | 3 | 3 | 3 | 4 | 4 |
| PHI:346 | 6 | 7 | 6 | 6 | 7 | 7 |
| PHI:348 | 1 | 1 | 1 | 1 | 1 | 1 |
| PHI:35 | 1 | 1 | 1 | 1 | 1 | 1 |
| PHI:350 | 1 | 1 | 1 | 1 | 1 | 1 |
| PHI:354 | 1 | 1 | 1 | 1 | 1 | 1 |
| PHI:355 | 3 | 3 | 3 | 3 | 3 | 4 |
| PHI:356 | 3 | 3 | 3 | 3 | 3 | 2 |
| PHI:358 | 8 | 3 | 8 | 8 | 3 | 6 |
| PHI:36 | 1 | 2 | 1 | 1 | 2 | 1 |
| PHI:360 | 1 | 1 | 1 | 1 | 1 | 1 |
| PHI:361 | 1 | 1 | 1 | 1 | 1 | 1 |
| PHI:362 | 4 | 4 | 4 | 4 | 4 | 4 |
| PHI:364 | 4 | 3 | 4 | 4 | 3 | 3 |
| PHI:365 | 1 | 1 | 1 | 1 | 1 | 1 |
| PHI:367 | 1 | 1 | 1 | 1 | 1 | 1 |
| PHI:368 | 1 | 1 | 1 | 1 | 1 | 1 |
| PHI:371 | 1 | 1 | 1 | 1 | 1 | 1 |
| PHI:372 | 1 | 1 | 1 | 1 | 1 | 1 |
| PHI:373 | 3 | 3 | 3 | 3 | 3 | 3 |
| PHI:375 | 1 | 1 | 1 | 1 | 1 | 1 |
| PHI:376 | 2 | 2 | 2 | 2 | 2 | 2 |
| PHI:378 | 5 | 5 | 5 | 5 | 6 | 6 |
| PHI:380 | 3 | 3 | 3 | 3 | 2 | 3 |
| PHI:382 | 2 | 3 | 2 | 2 | 3 | 3 |
| PHI:384 | 3 | 3 | 3 | 3 | 3 | 2 |
| PHI:386 | 1 | 2 | 2 | 2 | 2 | 2 |
| PHI:389 | 2 | 2 | 2 | 2 | 2 | 1 |
| PHI:390 | 1 | 1 | 1 | 1 | 1 | 1 |
| PHI:391 | 4 | 3 | 2 | 4 | 3 | 1 |
| PHI:392 | 2 | 2 | 2 | 2 | 2 | 2 |
| PHI:393 | 1 | 2 | 2 | 2 | 2 | 2 |
| PHI:394 | 2 | 2 | 2 | 2 | 2 | 1 |
| PHI:397 | 5 | 5 | 5 | 5 | 5 | 2 |
| PHI:398 | 1 | 1 | 1 | 1 | 1 | 1 |
| PHI:399 | 1 | 1 | 1 | 1 | 1 | 1 |
| PHI:40 | 2 | 2 | 2 | 2 | 2 | 1 |
| PHI:401 | 1 | 1 | 1 | 1 | 1 | 1 |
| PHI:404 | 21 | 14 | 21 | 21 | 14 | 10 |
| PHI:405 | 4 | 4 | 4 | 4 | 4 | 4 |
| PHI:406 | 3 | 2 | 3 | 3 | 3 | 4 |
| PHI:409 | 1 | 1 | 1 | 1 | 1 | 1 |
| PHI:412 | 3 | 5 | 4 | 4 | 4 | 4 |
| PHI:413 | 21 | 16 | 22 | 22 | 16 | 12 |
| PHI:414 | 2 | 2 | 2 | 2 | 2 | 2 |
| PHI:417 | 2 | 1 | 2 | 2 | 1 | 2 |
| PHI:418 | 3 | 4 | 3 | 3 | 5 | 4 |
| PHI:419 | 22 | 9 | 23 | 21 | 9 | 8 |
| PHI:420 | 1 | 1 | 1 | 1 | 1 | 1 |
| PHI:423 | 26 | 26 | 26 | 26 | 26 | 26 |
| PHI:424 | 1 | 1 | 1 | 1 | 1 | 1 |
| PHI:427 | 2 | 2 | 2 | 2 | 3 | 1 |
| PHI:428 | 1 | 1 | 1 | 1 | 1 | 1 |
| PHI:430 | 1 | 1 | 1 | 1 | 1 | 1 |
| PHI:431 | 2 | 2 | 2 | 2 | 2 | 2 |
| PHI:432 | 5 | 5 | 5 | 5 | 4 | 4 |
| PHI:434 | 2 | 2 | 2 | 2 | 2 | 2 |
| PHI:435 | 1 | 1 | 1 | 1 | 1 | 1 |
| PHI:436 | 1 | 1 | 1 | 1 | 1 | 1 |
| PHI:438 | 91 | 54 | 91 | 93 | 56 | 31 |
| PHI:439 | 1 | 1 | 1 | 1 | 1 | 1 |
| PHI:44 | 2 | 1 | 2 | 2 | 2 | 2 |
| PHI:440 | 14 | 13 | 15 | 15 | 14 | 13 |
| PHI:441 | 40 | 29 | 43 | 43 | 30 | 28 |
| PHI:442 | 1 | 1 | 1 | 1 | 1 | 1 |
| PHI:443 | 6 | 7 | 7 | 7 | 7 | 4 |
| PHI:444 | 1 | 1 | 1 | 1 | 1 | 1 |
| PHI:445 | 1 | 1 | 1 | 1 | 1 | 1 |
| PHI:446 | 1 | 1 | 1 | 1 | 1 | 1 |
| PHI:447 | 2 | 3 | 2 | 2 | 3 | 2 |
| PHI:451 | 1 | 1 | 1 | 1 | 1 | 1 |
| PHI:454 | 1 | 1 | 1 | 1 | 1 | 1 |
| PHI:455 | 2 | 2 | 2 | 2 | 2 | 2 |
| PHI:463 | 1 | 1 | 1 | 1 | 1 | 1 |
| PHI:464 | 1 | 1 | 1 | 1 | 1 | 1 |
| PHI:465 | 10 | 9 | 10 | 10 | 9 | 9 |
| PHI:469 | 2 | 1 | 2 | 2 | 1 | 2 |
| PHI:470 | 2 | 3 | 2 | 2 | 3 | 1 |
| PHI:474 | 2 | 4 | 2 | 2 | 3 | 2 |
| PHI:475 | 1 | 1 | 1 | 1 | 2 | 1 |
| PHI:479 | 1 | 1 | 1 | 1 | 1 | 1 |
| PHI:481 | 1 | 1 | 1 | 1 | 1 | 1 |
| PHI:482 | 14 | 11 | 15 | 16 | 12 | 13 |
| PHI:485 | 2 | 1 | 2 | 2 | 1 | 1 |
| PHI:486 | 1 | 1 | 1 | 1 | 1 | 1 |
| PHI:488 | 2 | 2 | 2 | 2 | 2 | 2 |
| PHI:489 | 1 | 1 | 1 | 1 | 1 | 1 |
| PHI:491 | 1 | 2 | 1 | 1 | 2 | 1 |
| PHI:493 | 2 | 2 | 2 | 2 | 2 | 2 |
| PHI:494 | 1 | 1 | 1 | 1 | 1 | 1 |
| PHI:496 | 1 | 1 | 1 | 1 | 1 | 1 |
| PHI:497 | 1 | 6 | 1 | 1 | 4 | 1 |
| PHI:500 | 7 | 3 | 7 | 7 | 2 | 3 |
| PHI:501 | 1 | 1 | 1 | 1 | 1 | 1 |
| PHI:502 | 2 | 1 | 2 | 2 | 2 | 2 |
| PHI:503 | 1 | 1 | 1 | 1 | 1 | 1 |
| PHI:504 | 7 | 6 | 7 | 7 | 6 | 5 |
| PHI:505 | 1 | 1 | 1 | 1 | 1 | 1 |
| PHI:506 | 2 | 2 | 2 | 2 | 2 | 2 |
| PHI:508 | 7 | 8 | 8 | 8 | 7 | 4 |
| PHI:509 | 10 | 8 | 11 | 11 | 8 | 7 |
| PHI:510 | 21 | 13 | 22 | 22 | 16 | 6 |
| PHI:511 | 38 | 30 | 40 | 39 | 30 | 27 |
| PHI:512 | 1 | 1 | 1 | 1 | 1 | 1 |
| PHI:513 | 15 | 8 | 15 | 15 | 9 | 8 |
| PHI:518 | 3 | 4 | 3 | 3 | 5 | 3 |
| PHI:520 | 1 | 1 | 1 | 1 | 1 | 1 |
| PHI:522 | 1 | 1 | 1 | 1 | 1 | 1 |
| PHI:524 | 1 | 1 | 1 | 1 | 1 | 1 |
| PHI:528 | 2 | 2 | 2 | 2 | 3 | 3 |
| PHI:538 | 106 | 60 | 111 | 110 | 62 | 55 |
| PHI:541 | 31 | 16 | 33 | 33 | 15 | 13 |
| PHI:542 | 1 | 1 | 1 | 1 | 1 | 1 |
| PHI:543 | 4 | 4 | 4 | 4 | 4 | 4 |
| PHI:544 | 64 | 32 | 66 | 65 | 33 | 21 |
| PHI:546 | 1 | 1 | 1 | 1 | 1 | 1 |
| PHI:547 | 4 | 2 | 4 | 4 | 2 | 3 |
| PHI:548 | 4 | 4 | 4 | 4 | 4 | 4 |
| PHI:55 | 26 | 20 | 27 | 27 | 19 | 18 |
| PHI:550 | 1 | 1 | 1 | 1 | 2 | 1 |
| PHI:552 | 4 | 2 | 4 | 4 | 2 | 4 |
| PHI:553 | 4 | 5 | 4 | 4 | 4 | 3 |
| PHI:564 | 2 | 2 | 2 | 2 | 2 | 2 |
| PHI:565 | 1 | 1 | 1 | 1 | 1 | 1 |
| PHI:566 | 2 | 2 | 2 | 2 | 2 | 2 |
| PHI:57 | 1 | 1 | 1 | 1 | 1 | 1 |
| PHI:577 | 5 | 2 | 4 | 4 | 2 | 3 |
| PHI:58 | 2 | 1 | 2 | 2 | 1 | 1 |
| PHI:587 | 5 | 5 | 5 | 5 | 5 | 4 |
| PHI:59 | 48 | 26 | 48 | 48 | 24 | 19 |
| PHI:591 | 1 | 1 | 1 | 1 | 1 | 1 |
| PHI:593 | 2 | 2 | 3 | 2 | 2 | 2 |
| PHI:594 | 2 | 2 | 2 | 2 | 2 | 2 |
| PHI:595 | 5 | 4 | 5 | 4 | 4 | 4 |
| PHI:598 | 7 | 7 | 8 | 8 | 7 | 8 |
| PHI:599 | 2 | 1 | 2 | 2 | 1 | 1 |
| PHI:600 | 1 | 1 | 1 | 1 | 1 | 1 |
| PHI:61 | 2 | 2 | 2 | 2 | 2 | 2 |
| PHI:612 | 2 | 2 | 2 | 2 | 3 | 1 |
| PHI:616 | 1 | 1 | 1 | 1 | 1 | 1 |
| PHI:624 | 1 | 2 | 1 | 1 | 1 | 2 |
| PHI:645 | 3 | 2 | 3 | 3 | 3 | 2 |
| PHI:65 | 2 | 2 | 2 | 2 | 1 | 2 |
| PHI:650 | 1 | 1 | 1 | 1 | 2 | 1 |
| PHI:651 | 1 | 1 | 1 | 1 | 1 | 1 |
| PHI:667 | 1 | 1 | 1 | 1 | 1 | 1 |
| PHI:668 | 12 | 5 | 12 | 12 | 5 | 4 |
| PHI:67 | 1 | 4 | 1 | 1 | 5 | 1 |
| PHI:673 | 1 | 1 | 1 | 1 | 1 | 1 |
| PHI:674 | 1 | 1 | 1 | 1 | 1 | 1 |
| PHI:675 | 2 | 2 | 2 | 2 | 2 | 2 |
| PHI:69 | 1 | 1 | 1 | 1 | 1 | 1 |
| PHI:693 | 1 | 1 | 1 | 1 | 1 | 1 |
| PHI:695 | 1 | 1 | 1 | 1 | 1 | 1 |
| PHI:697 | 6 | 5 | 6 | 6 | 6 | 5 |
| PHI:714 | 10 | 3 | 10 | 10 | 3 | 2 |
| PHI:716 | 15 | 11 | 16 | 16 | 10 | 5 |
| PHI:731 | 2 | 1 | 2 | 2 | 1 | 1 |
| PHI:734 | 56 | 43 | 56 | 54 | 40 | 30 |
| PHI:737 | 18 | 14 | 19 | 18 | 17 | 17 |
| PHI:741 | 1 | 1 | 1 | 1 | 1 | 1 |
| PHI:748 | 12 | 6 | 12 | 13 | 6 | 7 |
| PHI:777 | 2 | 2 | 2 | 2 | 2 | 2 |
| PHI:781 | 2 | 5 | 4 | 4 | 5 | 4 |
| PHI:783 | 1 | 1 | 1 | 1 | 1 | 1 |
| PHI:784 | 36 | 24 | 37 | 37 | 25 | 15 |
| PHI:785 | 1 | 1 | 1 | 1 | 1 | 1 |
| PHI:789 | 3 | 2 | 3 | 3 | 2 | 2 |
| PHI:791 | 1 | 1 | 1 | 1 | 1 | 1 |
| PHI:792 | 2 | 2 | 2 | 2 | 2 | 2 |
| PHI:795 | 1 | 1 | 1 | 1 | 1 | 1 |
| PHI:796 | 2 | 2 | 2 | 2 | 2 | 2 |
| PHI:797 | 2 | 2 | 2 | 2 | 2 | 2 |
| PHI:799 | 3 | 3 | 3 | 3 | 3 | 3 |
| PHI:80 | 1 | 1 | 1 | 1 | 1 | 1 |
| PHI:800 | 1 | 2 | 1 | 1 | 2 | 1 |
| PHI:801 | 1 | 1 | 1 | 1 | 1 | 1 |
| PHI:802 | 1 | 2 | 2 | 2 | 2 | 2 |
| PHI:803 | 2 | 2 | 2 | 2 | 2 | 2 |
| PHI:804 | 1 | 1 | 1 | 1 | 1 | 1 |
| PHI:805 | 1 | 1 | 1 | 1 | 1 | 1 |
| PHI:806 | 1 | 1 | 1 | 1 | 1 | 1 |
| PHI:807 | 1 | 2 | 1 | 1 | 1 | 1 |
| PHI:808 | 1 | 1 | 1 | 1 | 1 | 1 |
| PHI:81 | 4 | 4 | 4 | 4 | 4 | 5 |
| PHI:810 | 1 | 1 | 1 | 1 | 1 | 1 |
| PHI:811 | 2 | 2 | 2 | 2 | 2 | 2 |
| PHI:812 | 31 | 20 | 31 | 31 | 17 | 12 |
| PHI:815 | 1 | 1 | 1 | 1 | 1 | 1 |
| PHI:816 | 3 | 3 | 3 | 3 | 3 | 3 |
| PHI:817 | 4 | 3 | 5 | 6 | 5 | 1 |
| PHI:820 | 1 | 1 | 1 | 1 | 1 | 2 |
| PHI:822 | 1 | 1 | 1 | 1 | 1 | 1 |
| PHI:823 | 2 | 3 | 3 | 3 | 4 | 2 |
| PHI:830 | 1 | 2 | 1 | 1 | 1 | 1 |
| PHI:831 | 1 | 1 | 1 | 1 | 1 | 1 |
| PHI:838 | 2 | 2 | 2 | 2 | 2 | 2 |
| PHI:84 | 1 | 1 | 1 | 1 | 1 | 2 |
| PHI:843 | 2 | 2 | 2 | 3 | 1 | 3 |
| PHI:846 | 5 | 2 | 5 | 5 | 3 | 4 |
| PHI:853 | 3 | 1 | 3 | 3 | 2 | 3 |
| PHI:854 | 3 | 2 | 3 | 3 | 2 | 2 |
| PHI:856 | 1 | 1 | 1 | 1 | 1 | 1 |
| PHI:857 | 1 | 1 | 1 | 1 | 1 | 1 |
| PHI:858 | 1 | 1 | 1 | 1 | 1 | 1 |
| PHI:860 | 4 | 4 | 6 | 7 | 4 | 4 |
| PHI:862 | 2 | 2 | 2 | 2 | 2 | 2 |
| PHI:864 | 7 | 6 | 6 | 6 | 6 | 6 |
| PHI:867 | 4 | 4 | 5 | 5 | 4 | 4 |
| PHI:872 | 2 | 2 | 2 | 2 | 2 | 3 |
| PHI:873 | 1 | 1 | 1 | 1 | 1 | 1 |
| PHI:875 | 1 | 1 | 1 | 1 | 1 | 1 |
| PHI:877 | 2 | 1 | 2 | 2 | 1 | 1 |
| PHI:881 | 39 | 21 | 39 | 40 | 21 | 16 |
| PHI:882 | 3 | 5 | 4 | 4 | 5 | 5 |
| PHI:887 | 1 | 1 | 1 | 1 | 1 | 1 |
| PHI:888 | 1 | 1 | 1 | 1 | 1 | 1 |
| PHI:889 | 7 | 9 | 8 | 9 | 5 | 8 |
| PHI:89 | 3 | 2 | 3 | 3 | 2 | 2 |
| PHI:890 | 3 | 1 | 3 | 3 | 2 | 3 |
| PHI:891 | 2 | 3 | 2 | 2 | 2 | 1 |
| PHI:893 | 3 | 3 | 3 | 3 | 3 | 3 |
| PHI:901 | 7 | 6 | 7 | 7 | 6 | 4 |
| PHI:903 | 4 | 4 | 4 | 4 | 4 | 3 |
| PHI:911 | 3 | 3 | 3 | 3 | 3 | 3 |
| PHI:922 | 21 | 16 | 22 | 21 | 16 | 14 |
| PHI:923 | 3 | 3 | 3 | 3 | 3 | 3 |
| PHI:96 | 5 | 2 | 4 | 4 | 2 | 2 |
| PHI:97 | 3 | 2 | 3 | 3 | 2 | 2 |
| PHI:981 | 9 | 12 | 11 | 11 | 12 | 12 |
| Total | 2091 | 1663 | 2168 | 2164 | 1666 | 1434 |

**(B)** Specific PHI gene categories and numbers

| subgrouph | CMP1 | subgroup | Gan1 | subgroup | MD8 | subgroup | Pd1 | subgroup | PHI1 | subgroup | PHI26 |
| --- | --- | --- | --- | --- | --- | --- | --- | --- | --- | --- | --- |
| PHI:100 | 3 | PHI:100 | 2 | PHI:100 | 3 | PHI:100 | 3 | PHI:100 | 3 | PHI:100 | 5 |
| PHI:1005 | 1 | PHI:1005 | 2 | PHI:1005 | 2 | PHI:1005 | 2 | PHI:1005 | 2 | PHI:1005 | 2 |
| PHI:1006 | 2 | PHI:1006 | 1 | PHI:1006 | 2 | PHI:1006 | 2 | PHI:1006 | 1 | PHI:1006 | 1 |
| PHI:1008 | 2 | PHI:1008 | 1 | PHI:1008 | 1 | PHI:1008 | 1 | PHI:1008 | 2 | PHI:1008 | 1 |
| PHI:101 | 3 | PHI:101 | 4 | PHI:1009 | 1 | PHI:1009 | 1 | PHI:101 | 4 | PHI:101 | 3 |
| PHI:1018 | 19 | PHI:1018 | 15 | PHI:101 | 3 | PHI:101 | 3 | PHI:1018 | 14 | PHI:1010 | 1 |
| PHI:1021 | 38 | PHI:1021 | 30 | PHI:1018 | 19 | PHI:1018 | 19 | PHI:1021 | 31 | PHI:1018 | 15 |
| PHI:1022 | 12 | PHI:1022 | 10 | PHI:1021 | 41 | PHI:1021 | 42 | PHI:1022 | 8 | PHI:1021 | 18 |
| PHI:1023 | 2 | PHI:1023 | 2 | PHI:1022 | 11 | PHI:1022 | 12 | PHI:1023 | 1 | PHI:1022 | 7 |
| PHI:1025 | 1 | PHI:1025 | 1 | PHI:1023 | 2 | PHI:1023 | 2 | PHI:1025 | 1 | PHI:1023 | 1 |
| PHI:1026 | 2 | PHI:1026 | 2 | PHI:1025 | 1 | PHI:1025 | 1 | PHI:1026 | 2 | PHI:1025 | 1 |
| PHI:1027 | 1 | PHI:1028 | 1 | PHI:1026 | 2 | PHI:1026 | 2 | PHI:1028 | 1 | PHI:1026 | 2 |
| PHI:1028 | 3 | PHI:1029 | 1 | PHI:1027 | 1 | PHI:1027 | 1 | PHI:1029 | 1 | PHI:1027 | 1 |
| PHI:1030 | 4 | PHI:1030 | 3 | PHI:1028 | 3 | PHI:1028 | 3 | PHI:1030 | 4 | PHI:1028 | 1 |
| PHI:1032 | 1 | PHI:1031 | 1 | PHI:1030 | 4 | PHI:1030 | 4 | PHI:1031 | 1 | PHI:1029 | 2 |
| PHI:1034 | 1 | PHI:1032 | 1 | PHI:1032 | 1 | PHI:1032 | 1 | PHI:1032 | 1 | PHI:1030 | 4 |
| PHI:1037 | 1 | PHI:1034 | 1 | PHI:1034 | 1 | PHI:1034 | 1 | PHI:1034 | 1 | PHI:1032 | 1 |
| PHI:1039 | 1 | PHI:1035 | 1 | PHI:1037 | 1 | PHI:1037 | 1 | PHI:1035 | 2 | PHI:1034 | 1 |
| PHI:104 | 1 | PHI:1037 | 2 | PHI:1039 | 1 | PHI:1039 | 1 | PHI:1037 | 1 | PHI:1037 | 1 |
| PHI:1046 | 19 | PHI:1039 | 2 | PHI:104 | 1 | PHI:104 | 1 | PHI:1039 | 1 | PHI:1039 | 1 |
| PHI:1047 | 10 | PHI:104 | 1 | PHI:1046 | 21 | PHI:1046 | 19 | PHI:104 | 1 | PHI:104 | 1 |
| PHI:1048 | 2 | PHI:1046 | 13 | PHI:1047 | 10 | PHI:1047 | 10 | PHI:1046 | 12 | PHI:1046 | 8 |
| PHI:1049 | 1 | PHI:1047 | 5 | PHI:1048 | 2 | PHI:1048 | 2 | PHI:1047 | 6 | PHI:1047 | 3 |
| PHI:105 | 1 | PHI:1048 | 1 | PHI:105 | 1 | PHI:105 | 2 | PHI:1048 | 1 | PHI:1048 | 1 |
| PHI:1050 | 4 | PHI:1049 | 3 | PHI:1050 | 4 | PHI:1050 | 4 | PHI:1049 | 4 | PHI:1049 | 1 |
| PHI:1051 | 19 | PHI:105 | 1 | PHI:1051 | 21 | PHI:1051 | 21 | PHI:105 | 1 | PHI:105 | 1 |
| PHI:1052 | 1 | PHI:1050 | 6 | PHI:1052 | 1 | PHI:1052 | 1 | PHI:1050 | 2 | PHI:1050 | 3 |
| PHI:1056 | 1 | PHI:1051 | 8 | PHI:1056 | 1 | PHI:1056 | 1 | PHI:1051 | 10 | PHI:1051 | 9 |
| PHI:1057 | 2 | PHI:1052 | 2 | PHI:1057 | 2 | PHI:1057 | 2 | PHI:1052 | 2 | PHI:1052 | 2 |
| PHI:1058 | 4 | PHI:1056 | 2 | PHI:1058 | 4 | PHI:1058 | 4 | PHI:1056 | 1 | PHI:1056 | 2 |
| PHI:106 | 3 | PHI:1057 | 2 | PHI:106 | 5 | PHI:106 | 5 | PHI:1057 | 3 | PHI:1057 | 2 |
| PHI:1063 | 2 | PHI:1058 | 5 | PHI:1061 | 1 | PHI:1061 | 1 | PHI:1058 | 6 | PHI:1058 | 3 |
| PHI:1064 | 1 | PHI:106 | 2 | PHI:1063 | 2 | PHI:1063 | 2 | PHI:106 | 3 | PHI:106 | 2 |
| PHI:1070 | 2 | PHI:1061 | 1 | PHI:1064 | 1 | PHI:1064 | 1 | PHI:1061 | 1 | PHI:1061 | 1 |
| PHI:1071 | 6 | PHI:1063 | 2 | PHI:1070 | 2 | PHI:1070 | 2 | PHI:1063 | 2 | PHI:1063 | 2 |
| PHI:1072 | 1 | PHI:1064 | 1 | PHI:1071 | 6 | PHI:1071 | 6 | PHI:1064 | 1 | PHI:1064 | 1 |
| PHI:1074 | 1 | PHI:1070 | 2 | PHI:1072 | 1 | PHI:1072 | 1 | PHI:1070 | 2 | PHI:1070 | 2 |
| PHI:1075 | 4 | PHI:1071 | 5 | PHI:1074 | 1 | PHI:1074 | 1 | PHI:1071 | 5 | PHI:1071 | 5 |
| PHI:1082 | 3 | PHI:1072 | 1 | PHI:1075 | 4 | PHI:1075 | 4 | PHI:1072 | 1 | PHI:1073 | 1 |
| PHI:1083 | 5 | PHI:1073 | 1 | PHI:1082 | 3 | PHI:1082 | 3 | PHI:1074 | 1 | PHI:1074 | 1 |
| PHI:1085 | 7 | PHI:1074 | 1 | PHI:1083 | 5 | PHI:1083 | 5 | PHI:1075 | 4 | PHI:1075 | 2 |
| PHI:109 | 4 | PHI:1075 | 3 | PHI:1085 | 7 | PHI:1085 | 7 | PHI:1082 | 2 | PHI:1076 | 1 |
| PHI:1100 | 35 | PHI:1076 | 1 | PHI:109 | 3 | PHI:109 | 3 | PHI:1083 | 4 | PHI:1082 | 2 |
| PHI:112 | 6 | PHI:1082 | 2 | PHI:1100 | 37 | PHI:1100 | 35 | PHI:1085 | 4 | PHI:1083 | 4 |
| PHI:113 | 2 | PHI:1083 | 6 | PHI:112 | 8 | PHI:112 | 7 | PHI:109 | 3 | PHI:1085 | 5 |
| PHI:115 | 6 | PHI:1085 | 4 | PHI:113 | 2 | PHI:113 | 2 | PHI:1100 | 26 | PHI:109 | 2 |
| PHI:12 | 12 | PHI:109 | 3 | PHI:115 | 6 | PHI:115 | 6 | PHI:112 | 2 | PHI:1100 | 19 |
| PHI:121 | 1 | PHI:1100 | 26 | PHI:12 | 12 | PHI:12 | 12 | PHI:113 | 3 | PHI:112 | 5 |
| PHI:124 | 1 | PHI:112 | 2 | PHI:121 | 1 | PHI:121 | 1 | PHI:115 | 4 | PHI:113 | 2 |
| PHI:127 | 1 | PHI:113 | 3 | PHI:124 | 1 | PHI:124 | 1 | PHI:12 | 7 | PHI:115 | 6 |
| PHI:131 | 2 | PHI:115 | 4 | PHI:126 | 2 | PHI:126 | 2 | PHI:121 | 1 | PHI:12 | 9 |
| PHI:133 | 14 | PHI:12 | 7 | PHI:131 | 2 | PHI:131 | 2 | PHI:124 | 1 | PHI:121 | 1 |
| PHI:134 | 2 | PHI:121 | 1 | PHI:133 | 13 | PHI:133 | 13 | PHI:126 | 1 | PHI:124 | 1 |
| PHI:135 | 1 | PHI:124 | 1 | PHI:134 | 3 | PHI:134 | 3 | PHI:131 | 2 | PHI:127 | 1 |
| PHI:139 | 5 | PHI:126 | 1 | PHI:135 | 1 | PHI:135 | 1 | PHI:133 | 10 | PHI:133 | 9 |
| PHI:140 | 1 | PHI:131 | 2 | PHI:139 | 5 | PHI:138 | 1 | PHI:134 | 1 | PHI:134 | 1 |
| PHI:141 | 3 | PHI:133 | 8 | PHI:140 | 1 | PHI:139 | 5 | PHI:135 | 1 | PHI:135 | 1 |
| PHI:143 | 6 | PHI:134 | 1 | PHI:141 | 3 | PHI:140 | 1 | PHI:136 | 2 | PHI:136 | 3 |
| PHI:144 | 11 | PHI:135 | 1 | PHI:143 | 5 | PHI:141 | 3 | PHI:138 | 1 | PHI:138 | 1 |
| PHI:146 | 1 | PHI:138 | 2 | PHI:144 | 12 | PHI:143 | 5 | PHI:139 | 4 | PHI:139 | 4 |
| PHI:149 | 1 | PHI:139 | 5 | PHI:146 | 1 | PHI:144 | 12 | PHI:141 | 3 | PHI:141 | 1 |
| PHI:153 | 6 | PHI:141 | 2 | PHI:149 | 2 | PHI:146 | 1 | PHI:143 | 5 | PHI:143 | 4 |
| PHI:156 | 1 | PHI:143 | 4 | PHI:153 | 6 | PHI:149 | 2 | PHI:144 | 10 | PHI:144 | 5 |
| PHI:157 | 6 | PHI:144 | 10 | PHI:156 | 1 | PHI:153 | 6 | PHI:146 | 1 | PHI:146 | 1 |
| PHI:158 | 3 | PHI:146 | 1 | PHI:157 | 6 | PHI:156 | 1 | PHI:149 | 3 | PHI:149 | 1 |
| PHI:159 | 2 | PHI:149 | 4 | PHI:158 | 3 | PHI:157 | 6 | PHI:153 | 8 | PHI:153 | 3 |
| PHI:160 | 13 | PHI:153 | 6 | PHI:159 | 2 | PHI:158 | 3 | PHI:157 | 4 | PHI:156 | 1 |
| PHI:165 | 1 | PHI:156 | 2 | PHI:160 | 13 | PHI:159 | 2 | PHI:158 | 3 | PHI:157 | 3 |
| PHI:167 | 5 | PHI:157 | 3 | PHI:165 | 1 | PHI:160 | 15 | PHI:159 | 2 | PHI:158 | 3 |
| PHI:169 | 20 | PHI:158 | 3 | PHI:167 | 5 | PHI:165 | 1 | PHI:160 | 3 | PHI:159 | 2 |
| PHI:17 | 1 | PHI:159 | 2 | PHI:169 | 21 | PHI:167 | 6 | PHI:165 | 2 | PHI:160 | 6 |
| PHI:172 | 3 | PHI:160 | 3 | PHI:17 | 1 | PHI:169 | 23 | PHI:167 | 6 | PHI:165 | 1 |
| PHI:174 | 1 | PHI:165 | 2 | PHI:172 | 3 | PHI:17 | 1 | PHI:169 | 15 | PHI:167 | 4 |
| PHI:177 | 3 | PHI:167 | 6 | PHI:174 | 1 | PHI:172 | 3 | PHI:17 | 1 | PHI:169 | 10 |
| PHI:178 | 4 | PHI:169 | 16 | PHI:177 | 3 | PHI:174 | 1 | PHI:172 | 4 | PHI:172 | 5 |
| PHI:18 | 1 | PHI:17 | 2 | PHI:178 | 4 | PHI:177 | 3 | PHI:174 | 1 | PHI:174 | 1 |
| PHI:180 | 1 | PHI:172 | 4 | PHI:18 | 1 | PHI:178 | 4 | PHI:177 | 2 | PHI:177 | 2 |
| PHI:182 | 1 | PHI:174 | 1 | PHI:180 | 1 | PHI:18 | 1 | PHI:178 | 4 | PHI:178 | 4 |
| PHI:184 | 2 | PHI:177 | 2 | PHI:182 | 1 | PHI:180 | 1 | PHI:180 | 1 | PHI:180 | 1 |
| PHI:186 | 1 | PHI:178 | 4 | PHI:184 | 2 | PHI:182 | 1 | PHI:182 | 1 | PHI:182 | 1 |
| PHI:187 | 8 | PHI:180 | 1 | PHI:186 | 1 | PHI:184 | 2 | PHI:184 | 2 | PHI:184 | 2 |
| PHI:188 | 6 | PHI:182 | 1 | PHI:187 | 8 | PHI:186 | 1 | PHI:186 | 1 | PHI:186 | 1 |
| PHI:189 | 1 | PHI:184 | 2 | PHI:188 | 6 | PHI:187 | 8 | PHI:187 | 6 | PHI:187 | 5 |
| PHI:19 | 2 | PHI:186 | 1 | PHI:189 | 1 | PHI:188 | 6 | PHI:188 | 9 | PHI:188 | 10 |
| PHI:190 | 1 | PHI:187 | 6 | PHI:19 | 2 | PHI:189 | 1 | PHI:189 | 1 | PHI:189 | 1 |
| PHI:191 | 8 | PHI:188 | 8 | PHI:190 | 1 | PHI:19 | 2 | PHI:19 | 2 | PHI:19 | 2 |
| PHI:194 | 1 | PHI:189 | 1 | PHI:191 | 8 | PHI:190 | 1 | PHI:190 | 1 | PHI:190 | 1 |
| PHI:195 | 4 | PHI:19 | 2 | PHI:194 | 1 | PHI:191 | 7 | PHI:191 | 6 | PHI:191 | 5 |
| PHI:197 | 3 | PHI:190 | 1 | PHI:195 | 4 | PHI:194 | 1 | PHI:194 | 2 | PHI:194 | 1 |
| PHI:199 | 4 | PHI:191 | 5 | PHI:197 | 3 | PHI:195 | 4 | PHI:195 | 3 | PHI:195 | 4 |
| PHI:200 | 2 | PHI:194 | 1 | PHI:199 | 4 | PHI:197 | 3 | PHI:197 | 2 | PHI:197 | 1 |
| PHI:201 | 2 | PHI:195 | 3 | PHI:200 | 2 | PHI:199 | 5 | PHI:199 | 3 | PHI:199 | 2 |
| PHI:202 | 7 | PHI:197 | 1 | PHI:201 | 2 | PHI:200 | 2 | PHI:200 | 1 | PHI:200 | 1 |
| PHI:206 | 1 | PHI:199 | 3 | PHI:202 | 8 | PHI:201 | 2 | PHI:202 | 4 | PHI:202 | 7 |
| PHI:207 | 1 | PHI:200 | 1 | PHI:206 | 1 | PHI:202 | 7 | PHI:206 | 1 | PHI:206 | 1 |
| PHI:208 | 5 | PHI:202 | 5 | PHI:207 | 1 | PHI:206 | 1 | PHI:207 | 1 | PHI:207 | 1 |
| PHI:210 | 1 | PHI:206 | 1 | PHI:208 | 5 | PHI:207 | 1 | PHI:208 | 4 | PHI:208 | 5 |
| PHI:211 | 47 | PHI:207 | 1 | PHI:210 | 1 | PHI:208 | 5 | PHI:209 | 1 | PHI:209 | 1 |
| PHI:212 | 1 | PHI:208 | 4 | PHI:211 | 52 | PHI:210 | 1 | PHI:211 | 48 | PHI:210 | 1 |
| PHI:213 | 4 | PHI:209 | 1 | PHI:212 | 1 | PHI:211 | 52 | PHI:213 | 6 | PHI:211 | 45 |
| PHI:215 | 1 | PHI:210 | 1 | PHI:213 | 5 | PHI:213 | 5 | PHI:215 | 1 | PHI:213 | 5 |
| PHI:216 | 1 | PHI:211 | 53 | PHI:215 | 1 | PHI:215 | 1 | PHI:216 | 1 | PHI:215 | 1 |
| PHI:218 | 1 | PHI:213 | 6 | PHI:216 | 1 | PHI:216 | 1 | PHI:217 | 1 | PHI:216 | 1 |
| PHI:219 | 4 | PHI:215 | 1 | PHI:218 | 1 | PHI:218 | 1 | PHI:218 | 1 | PHI:218 | 1 |
| PHI:22 | 4 | PHI:216 | 1 | PHI:219 | 4 | PHI:219 | 4 | PHI:219 | 4 | PHI:219 | 4 |
| PHI:220 | 2 | PHI:217 | 1 | PHI:22 | 4 | PHI:22 | 4 | PHI:22 | 3 | PHI:22 | 4 |
| PHI:222 | 4 | PHI:218 | 1 | PHI:220 | 2 | PHI:220 | 2 | PHI:220 | 3 | PHI:220 | 2 |
| PHI:226 | 15 | PHI:219 | 4 | PHI:222 | 4 | PHI:222 | 4 | PHI:222 | 3 | PHI:222 | 3 |
| PHI:228 | 1 | PHI:22 | 3 | PHI:226 | 15 | PHI:226 | 15 | PHI:226 | 15 | PHI:226 | 17 |
| PHI:231 | 2 | PHI:220 | 2 | PHI:228 | 1 | PHI:228 | 1 | PHI:227 | 1 | PHI:228 | 1 |
| PHI:233 | 16 | PHI:222 | 3 | PHI:231 | 2 | PHI:231 | 2 | PHI:228 | 1 | PHI:231 | 2 |
| PHI:235 | 1 | PHI:226 | 15 | PHI:233 | 19 | PHI:233 | 18 | PHI:231 | 2 | PHI:233 | 2 |
| PHI:236 | 1 | PHI:228 | 1 | PHI:234 | 1 | PHI:235 | 1 | PHI:233 | 10 | PHI:235 | 1 |
| PHI:238 | 1 | PHI:231 | 2 | PHI:235 | 1 | PHI:236 | 1 | PHI:235 | 1 | PHI:236 | 1 |
| PHI:24 | 2 | PHI:232 | 1 | PHI:236 | 1 | PHI:238 | 1 | PHI:236 | 1 | PHI:24 | 2 |
| PHI:240 | 1 | PHI:233 | 11 | PHI:238 | 1 | PHI:24 | 5 | PHI:24 | 1 | PHI:240 | 2 |
| PHI:242 | 3 | PHI:235 | 1 | PHI:24 | 6 | PHI:240 | 1 | PHI:240 | 1 | PHI:242 | 3 |
| PHI:243 | 8 | PHI:236 | 1 | PHI:240 | 1 | PHI:241 | 1 | PHI:241 | 2 | PHI:243 | 2 |
| PHI:244 | 5 | PHI:24 | 1 | PHI:241 | 1 | PHI:242 | 3 | PHI:242 | 4 | PHI:244 | 3 |
| PHI:246 | 3 | PHI:240 | 2 | PHI:242 | 3 | PHI:243 | 8 | PHI:243 | 3 | PHI:246 | 2 |
| PHI:249 | 5 | PHI:241 | 2 | PHI:243 | 8 | PHI:244 | 5 | PHI:244 | 3 | PHI:249 | 3 |
| PHI:250 | 1 | PHI:242 | 3 | PHI:244 | 5 | PHI:246 | 3 | PHI:246 | 5 | PHI:250 | 1 |
| PHI:251 | 1 | PHI:243 | 3 | PHI:246 | 3 | PHI:249 | 5 | PHI:249 | 4 | PHI:251 | 1 |
| PHI:252 | 3 | PHI:244 | 3 | PHI:249 | 5 | PHI:250 | 1 | PHI:250 | 1 | PHI:252 | 4 |
| PHI:253 | 2 | PHI:246 | 5 | PHI:250 | 1 | PHI:251 | 1 | PHI:251 | 1 | PHI:253 | 2 |
| PHI:254 | 3 | PHI:249 | 4 | PHI:251 | 1 | PHI:252 | 4 | PHI:252 | 5 | PHI:254 | 4 |
| PHI:255 | 12 | PHI:250 | 1 | PHI:252 | 4 | PHI:253 | 2 | PHI:253 | 2 | PHI:255 | 3 |
| PHI:258 | 3 | PHI:251 | 1 | PHI:253 | 2 | PHI:254 | 3 | PHI:254 | 4 | PHI:259 | 3 |
| PHI:259 | 3 | PHI:252 | 4 | PHI:254 | 3 | PHI:255 | 11 | PHI:255 | 12 | PHI:26 | 4 |
| PHI:26 | 19 | PHI:253 | 2 | PHI:255 | 11 | PHI:258 | 4 | PHI:259 | 3 | PHI:260 | 2 |
| PHI:260 | 2 | PHI:254 | 4 | PHI:258 | 4 | PHI:259 | 3 | PHI:26 | 11 | PHI:261 | 4 |
| PHI:261 | 4 | PHI:255 | 12 | PHI:259 | 3 | PHI:26 | 20 | PHI:260 | 2 | PHI:262 | 2 |
| PHI:262 | 2 | PHI:258 | 1 | PHI:26 | 21 | PHI:260 | 2 | PHI:261 | 2 | PHI:263 | 1 |
| PHI:265 | 1 | PHI:259 | 3 | PHI:260 | 2 | PHI:261 | 4 | PHI:262 | 2 | PHI:265 | 2 |
| PHI:266 | 1 | PHI:26 | 10 | PHI:261 | 4 | PHI:262 | 2 | PHI:265 | 2 | PHI:266 | 1 |
| PHI:267 | 28 | PHI:260 | 2 | PHI:262 | 2 | PHI:265 | 2 | PHI:266 | 1 | PHI:267 | 13 |
| PHI:268 | 4 | PHI:261 | 2 | PHI:265 | 2 | PHI:266 | 1 | PHI:267 | 18 | PHI:268 | 3 |
| PHI:269 | 1 | PHI:262 | 2 | PHI:266 | 1 | PHI:267 | 26 | PHI:268 | 2 | PHI:269 | 2 |
| PHI:27 | 1 | PHI:265 | 2 | PHI:267 | 27 | PHI:268 | 4 | PHI:269 | 2 | PHI:27 | 1 |
| PHI:270 | 4 | PHI:266 | 1 | PHI:268 | 4 | PHI:269 | 1 | PHI:27 | 1 | PHI:270 | 4 |
| PHI:273 | 1 | PHI:267 | 18 | PHI:269 | 1 | PHI:27 | 1 | PHI:270 | 3 | PHI:273 | 1 |
| PHI:274 | 3 | PHI:268 | 6 | PHI:27 | 1 | PHI:270 | 4 | PHI:273 | 1 | PHI:274 | 1 |
| PHI:275 | 1 | PHI:269 | 1 | PHI:270 | 4 | PHI:273 | 1 | PHI:274 | 3 | PHI:275 | 1 |
| PHI:277 | 1 | PHI:27 | 1 | PHI:273 | 1 | PHI:274 | 3 | PHI:275 | 2 | PHI:277 | 3 |
| PHI:279 | 1 | PHI:270 | 3 | PHI:274 | 3 | PHI:275 | 1 | PHI:277 | 2 | PHI:279 | 1 |
| PHI:280 | 4 | PHI:273 | 1 | PHI:275 | 1 | PHI:277 | 2 | PHI:279 | 1 | PHI:280 | 5 |
| PHI:281 | 4 | PHI:274 | 3 | PHI:277 | 2 | PHI:279 | 1 | PHI:280 | 4 | PHI:281 | 4 |
| PHI:282 | 1 | PHI:275 | 2 | PHI:279 | 1 | PHI:280 | 4 | PHI:281 | 4 | PHI:282 | 2 |
| PHI:283 | 2 | PHI:277 | 2 | PHI:280 | 4 | PHI:281 | 4 | PHI:282 | 1 | PHI:283 | 2 |
| PHI:285 | 2 | PHI:279 | 1 | PHI:281 | 4 | PHI:282 | 1 | PHI:283 | 2 | PHI:285 | 2 |
| PHI:286 | 3 | PHI:280 | 5 | PHI:282 | 1 | PHI:283 | 2 | PHI:285 | 2 | PHI:286 | 3 |
| PHI:287 | 1 | PHI:281 | 4 | PHI:283 | 2 | PHI:285 | 2 | PHI:286 | 3 | PHI:287 | 1 |
| PHI:290 | 2 | PHI:282 | 1 | PHI:285 | 2 | PHI:286 | 3 | PHI:287 | 1 | PHI:290 | 2 |
| PHI:292 | 1 | PHI:283 | 2 | PHI:286 | 3 | PHI:287 | 1 | PHI:290 | 1 | PHI:292 | 1 |
| PHI:294 | 4 | PHI:285 | 1 | PHI:287 | 1 | PHI:290 | 2 | PHI:292 | 1 | PHI:294 | 2 |
| PHI:296 | 3 | PHI:286 | 3 | PHI:290 | 2 | PHI:292 | 1 | PHI:294 | 3 | PHI:296 | 2 |
| PHI:3 | 2 | PHI:287 | 1 | PHI:292 | 1 | PHI:294 | 4 | PHI:296 | 3 | PHI:3 | 1 |
| PHI:300 | 3 | PHI:290 | 1 | PHI:294 | 4 | PHI:296 | 3 | PHI:3 | 2 | PHI:300 | 1 |
| PHI:301 | 3 | PHI:292 | 2 | PHI:296 | 2 | PHI:3 | 2 | PHI:300 | 2 | PHI:301 | 3 |
| PHI:303 | 1 | PHI:294 | 3 | PHI:3 | 2 | PHI:300 | 3 | PHI:301 | 2 | PHI:303 | 1 |
| PHI:304 | 1 | PHI:296 | 4 | PHI:300 | 3 | PHI:301 | 3 | PHI:303 | 1 | PHI:304 | 1 |
| PHI:305 | 1 | PHI:3 | 2 | PHI:301 | 3 | PHI:303 | 1 | PHI:304 | 1 | PHI:306 | 1 |
| PHI:307 | 1 | PHI:300 | 2 | PHI:303 | 1 | PHI:304 | 1 | PHI:305 | 1 | PHI:307 | 1 |
| PHI:31 | 1 | PHI:301 | 2 | PHI:304 | 1 | PHI:305 | 1 | PHI:307 | 1 | PHI:31 | 1 |
| PHI:310 | 3 | PHI:303 | 1 | PHI:305 | 1 | PHI:307 | 1 | PHI:308 | 1 | PHI:310 | 2 |
| PHI:311 | 1 | PHI:304 | 1 | PHI:307 | 1 | PHI:31 | 1 | PHI:31 | 1 | PHI:316 | 6 |
| PHI:315 | 2 | PHI:305 | 1 | PHI:31 | 1 | PHI:310 | 3 | PHI:310 | 2 | PHI:317 | 1 |
| PHI:316 | 4 | PHI:307 | 1 | PHI:310 | 3 | PHI:311 | 1 | PHI:311 | 1 | PHI:319 | 7 |
| PHI:317 | 1 | PHI:31 | 1 | PHI:311 | 1 | PHI:315 | 2 | PHI:315 | 1 | PHI:320 | 4 |
| PHI:319 | 7 | PHI:310 | 3 | PHI:315 | 2 | PHI:316 | 4 | PHI:316 | 5 | PHI:323 | 2 |
| PHI:320 | 4 | PHI:311 | 2 | PHI:316 | 4 | PHI:317 | 1 | PHI:317 | 1 | PHI:325 | 11 |
| PHI:323 | 3 | PHI:315 | 1 | PHI:317 | 1 | PHI:319 | 8 | PHI:319 | 8 | PHI:326 | 2 |
| PHI:325 | 14 | PHI:316 | 7 | PHI:319 | 8 | PHI:320 | 6 | PHI:320 | 5 | PHI:33 | 3 |
| PHI:326 | 2 | PHI:317 | 1 | PHI:320 | 6 | PHI:323 | 3 | PHI:323 | 3 | PHI:330 | 1 |
| PHI:33 | 4 | PHI:319 | 7 | PHI:323 | 3 | PHI:325 | 16 | PHI:325 | 11 | PHI:331 | 5 |
| PHI:331 | 5 | PHI:320 | 6 | PHI:325 | 19 | PHI:326 | 2 | PHI:326 | 2 | PHI:334 | 8 |
| PHI:332 | 1 | PHI:323 | 3 | PHI:326 | 2 | PHI:33 | 4 | PHI:33 | 3 | PHI:335 | 1 |
| PHI:334 | 6 | PHI:325 | 11 | PHI:33 | 4 | PHI:330 | 1 | PHI:330 | 1 | PHI:336 | 1 |
| PHI:335 | 1 | PHI:326 | 2 | PHI:330 | 1 | PHI:331 | 5 | PHI:331 | 4 | PHI:337 | 2 |
| PHI:336 | 1 | PHI:33 | 3 | PHI:331 | 5 | PHI:332 | 1 | PHI:334 | 7 | PHI:338 | 2 |
| PHI:337 | 2 | PHI:330 | 1 | PHI:332 | 1 | PHI:334 | 6 | PHI:335 | 1 | PHI:339 | 13 |
| PHI:338 | 2 | PHI:331 | 5 | PHI:334 | 6 | PHI:335 | 1 | PHI:336 | 1 | PHI:340 | 1 |
| PHI:339 | 13 | PHI:334 | 6 | PHI:335 | 1 | PHI:336 | 1 | PHI:337 | 2 | PHI:341 | 3 |
| PHI:340 | 1 | PHI:335 | 1 | PHI:336 | 1 | PHI:337 | 2 | PHI:338 | 2 | PHI:342 | 4 |
| PHI:341 | 4 | PHI:336 | 1 | PHI:337 | 2 | PHI:338 | 2 | PHI:339 | 15 | PHI:343 | 1 |
| PHI:342 | 3 | PHI:337 | 2 | PHI:338 | 2 | PHI:339 | 15 | PHI:340 | 1 | PHI:344 | 4 |
| PHI:343 | 1 | PHI:338 | 2 | PHI:339 | 15 | PHI:340 | 1 | PHI:341 | 4 | PHI:346 | 7 |
| PHI:344 | 3 | PHI:339 | 15 | PHI:340 | 1 | PHI:341 | 4 | PHI:342 | 8 | PHI:348 | 1 |
| PHI:346 | 6 | PHI:340 | 1 | PHI:341 | 4 | PHI:342 | 2 | PHI:343 | 1 | PHI:35 | 1 |
| PHI:348 | 1 | PHI:341 | 4 | PHI:342 | 2 | PHI:343 | 1 | PHI:344 | 4 | PHI:350 | 1 |
| PHI:35 | 1 | PHI:342 | 6 | PHI:343 | 1 | PHI:344 | 3 | PHI:346 | 7 | PHI:354 | 1 |
| PHI:350 | 1 | PHI:343 | 1 | PHI:344 | 3 | PHI:346 | 6 | PHI:348 | 1 | PHI:355 | 4 |
| PHI:352 | 2 | PHI:344 | 3 | PHI:346 | 6 | PHI:348 | 1 | PHI:35 | 1 | PHI:356 | 2 |
| PHI:354 | 1 | PHI:346 | 7 | PHI:348 | 1 | PHI:35 | 1 | PHI:350 | 1 | PHI:358 | 6 |
| PHI:355 | 3 | PHI:348 | 1 | PHI:35 | 1 | PHI:350 | 1 | PHI:352 | 1 | PHI:36 | 1 |
| PHI:356 | 3 | PHI:35 | 1 | PHI:350 | 1 | PHI:352 | 2 | PHI:354 | 1 | PHI:360 | 1 |
| PHI:358 | 8 | PHI:350 | 1 | PHI:352 | 2 | PHI:354 | 1 | PHI:355 | 3 | PHI:361 | 1 |
| PHI:36 | 1 | PHI:352 | 1 | PHI:354 | 1 | PHI:355 | 3 | PHI:356 | 3 | PHI:362 | 4 |
| PHI:360 | 1 | PHI:354 | 1 | PHI:355 | 3 | PHI:356 | 3 | PHI:358 | 3 | PHI:364 | 3 |
| PHI:361 | 1 | PHI:355 | 3 | PHI:356 | 3 | PHI:358 | 8 | PHI:36 | 2 | PHI:365 | 1 |
| PHI:362 | 4 | PHI:356 | 3 | PHI:358 | 8 | PHI:36 | 1 | PHI:360 | 1 | PHI:367 | 1 |
| PHI:364 | 4 | PHI:358 | 3 | PHI:36 | 1 | PHI:360 | 1 | PHI:361 | 1 | PHI:368 | 1 |
| PHI:365 | 1 | PHI:36 | 2 | PHI:360 | 1 | PHI:361 | 1 | PHI:362 | 4 | PHI:371 | 1 |
| PHI:367 | 1 | PHI:360 | 1 | PHI:361 | 1 | PHI:362 | 4 | PHI:364 | 3 | PHI:372 | 1 |
| PHI:368 | 1 | PHI:361 | 1 | PHI:362 | 4 | PHI:364 | 4 | PHI:365 | 1 | PHI:373 | 3 |
| PHI:371 | 1 | PHI:362 | 4 | PHI:364 | 4 | PHI:365 | 1 | PHI:367 | 1 | PHI:375 | 1 |
| PHI:372 | 1 | PHI:364 | 3 | PHI:365 | 1 | PHI:367 | 1 | PHI:368 | 1 | PHI:376 | 2 |
| PHI:373 | 3 | PHI:365 | 1 | PHI:367 | 1 | PHI:368 | 1 | PHI:371 | 1 | PHI:378 | 6 |
| PHI:375 | 1 | PHI:367 | 1 | PHI:368 | 1 | PHI:371 | 1 | PHI:372 | 1 | PHI:380 | 3 |
| PHI:376 | 2 | PHI:368 | 1 | PHI:371 | 1 | PHI:372 | 1 | PHI:373 | 3 | PHI:382 | 3 |
| PHI:378 | 5 | PHI:371 | 1 | PHI:372 | 1 | PHI:373 | 3 | PHI:375 | 1 | PHI:384 | 2 |
| PHI:380 | 3 | PHI:372 | 1 | PHI:373 | 3 | PHI:375 | 1 | PHI:376 | 2 | PHI:386 | 2 |
| PHI:382 | 2 | PHI:373 | 3 | PHI:375 | 1 | PHI:376 | 2 | PHI:378 | 6 | PHI:389 | 1 |
| PHI:384 | 3 | PHI:375 | 1 | PHI:376 | 2 | PHI:378 | 5 | PHI:380 | 2 | PHI:390 | 1 |
| PHI:386 | 1 | PHI:376 | 2 | PHI:378 | 5 | PHI:380 | 3 | PHI:382 | 3 | PHI:391 | 1 |
| PHI:387 | 1 | PHI:378 | 5 | PHI:380 | 3 | PHI:382 | 2 | PHI:384 | 3 | PHI:392 | 2 |
| PHI:389 | 2 | PHI:380 | 3 | PHI:382 | 2 | PHI:384 | 3 | PHI:386 | 2 | PHI:393 | 2 |
| PHI:390 | 1 | PHI:382 | 3 | PHI:384 | 3 | PHI:386 | 2 | PHI:387 | 1 | PHI:394 | 1 |
| PHI:391 | 4 | PHI:384 | 3 | PHI:386 | 2 | PHI:387 | 1 | PHI:389 | 2 | PHI:397 | 2 |
| PHI:392 | 2 | PHI:386 | 2 | PHI:387 | 1 | PHI:389 | 2 | PHI:390 | 1 | PHI:398 | 1 |
| PHI:393 | 1 | PHI:387 | 1 | PHI:389 | 2 | PHI:390 | 1 | PHI:391 | 3 | PHI:399 | 1 |
| PHI:394 | 2 | PHI:389 | 2 | PHI:390 | 1 | PHI:391 | 4 | PHI:392 | 2 | PHI:40 | 1 |
| PHI:397 | 5 | PHI:390 | 1 | PHI:391 | 2 | PHI:392 | 2 | PHI:393 | 2 | PHI:401 | 1 |
| PHI:398 | 1 | PHI:391 | 3 | PHI:392 | 2 | PHI:393 | 2 | PHI:394 | 2 | PHI:404 | 10 |
| PHI:399 | 1 | PHI:392 | 2 | PHI:393 | 2 | PHI:394 | 2 | PHI:397 | 5 | PHI:405 | 4 |
| PHI:40 | 2 | PHI:393 | 2 | PHI:394 | 2 | PHI:397 | 5 | PHI:398 | 1 | PHI:406 | 4 |
| PHI:401 | 1 | PHI:394 | 2 | PHI:397 | 5 | PHI:398 | 1 | PHI:399 | 1 | PHI:409 | 1 |
| PHI:404 | 21 | PHI:397 | 5 | PHI:398 | 1 | PHI:399 | 1 | PHI:40 | 2 | PHI:412 | 4 |
| PHI:405 | 4 | PHI:398 | 1 | PHI:399 | 1 | PHI:40 | 2 | PHI:401 | 1 | PHI:413 | 12 |
| PHI:406 | 3 | PHI:399 | 1 | PHI:40 | 2 | PHI:401 | 1 | PHI:404 | 14 | PHI:414 | 2 |
| PHI:409 | 1 | PHI:40 | 2 | PHI:401 | 1 | PHI:404 | 21 | PHI:405 | 4 | PHI:416 | 2 |
| PHI:412 | 3 | PHI:401 | 1 | PHI:404 | 21 | PHI:405 | 4 | PHI:406 | 3 | PHI:417 | 2 |
| PHI:413 | 21 | PHI:404 | 14 | PHI:405 | 4 | PHI:406 | 3 | PHI:409 | 1 | PHI:418 | 4 |
| PHI:414 | 2 | PHI:405 | 4 | PHI:406 | 3 | PHI:409 | 1 | PHI:412 | 4 | PHI:419 | 8 |
| PHI:416 | 3 | PHI:406 | 2 | PHI:409 | 1 | PHI:412 | 4 | PHI:413 | 16 | PHI:420 | 1 |
| PHI:417 | 2 | PHI:409 | 1 | PHI:412 | 4 | PHI:413 | 22 | PHI:414 | 2 | PHI:423 | 26 |
| PHI:418 | 3 | PHI:412 | 5 | PHI:413 | 22 | PHI:414 | 2 | PHI:417 | 1 | PHI:424 | 1 |
| PHI:419 | 22 | PHI:413 | 16 | PHI:414 | 2 | PHI:416 | 2 | PHI:418 | 5 | PHI:427 | 1 |
| PHI:420 | 1 | PHI:414 | 2 | PHI:416 | 2 | PHI:417 | 2 | PHI:419 | 9 | PHI:428 | 1 |
| PHI:423 | 26 | PHI:417 | 1 | PHI:417 | 2 | PHI:418 | 3 | PHI:420 | 1 | PHI:430 | 1 |
| PHI:424 | 1 | PHI:418 | 4 | PHI:418 | 3 | PHI:419 | 21 | PHI:423 | 26 | PHI:431 | 2 |
| PHI:427 | 2 | PHI:419 | 9 | PHI:419 | 23 | PHI:420 | 1 | PHI:424 | 1 | PHI:432 | 4 |
| PHI:428 | 1 | PHI:420 | 1 | PHI:420 | 1 | PHI:423 | 26 | PHI:427 | 3 | PHI:434 | 2 |
| PHI:430 | 1 | PHI:423 | 26 | PHI:423 | 26 | PHI:424 | 1 | PHI:428 | 1 | PHI:435 | 1 |
| PHI:431 | 2 | PHI:424 | 1 | PHI:424 | 1 | PHI:427 | 2 | PHI:430 | 1 | PHI:436 | 1 |
| PHI:432 | 5 | PHI:427 | 2 | PHI:427 | 2 | PHI:428 | 1 | PHI:431 | 2 | PHI:438 | 31 |
| PHI:434 | 2 | PHI:428 | 1 | PHI:428 | 1 | PHI:430 | 1 | PHI:432 | 4 | PHI:439 | 1 |
| PHI:435 | 1 | PHI:430 | 1 | PHI:430 | 1 | PHI:431 | 2 | PHI:434 | 2 | PHI:44 | 2 |
| PHI:436 | 1 | PHI:431 | 2 | PHI:431 | 2 | PHI:432 | 5 | PHI:435 | 1 | PHI:440 | 13 |
| PHI:438 | 91 | PHI:432 | 5 | PHI:432 | 5 | PHI:434 | 2 | PHI:436 | 1 | PHI:441 | 28 |
| PHI:439 | 1 | PHI:434 | 2 | PHI:434 | 2 | PHI:435 | 1 | PHI:438 | 56 | PHI:442 | 1 |
| PHI:44 | 2 | PHI:435 | 1 | PHI:435 | 1 | PHI:436 | 1 | PHI:439 | 1 | PHI:443 | 4 |
| PHI:440 | 14 | PHI:436 | 1 | PHI:436 | 1 | PHI:438 | 93 | PHI:44 | 2 | PHI:444 | 1 |
| PHI:441 | 40 | PHI:438 | 54 | PHI:438 | 91 | PHI:439 | 1 | PHI:440 | 14 | PHI:445 | 1 |
| PHI:442 | 1 | PHI:439 | 1 | PHI:439 | 1 | PHI:44 | 2 | PHI:441 | 30 | PHI:446 | 1 |
| PHI:443 | 6 | PHI:44 | 1 | PHI:44 | 2 | PHI:440 | 15 | PHI:442 | 1 | PHI:447 | 2 |
| PHI:444 | 1 | PHI:440 | 13 | PHI:440 | 15 | PHI:441 | 43 | PHI:443 | 7 | PHI:449 | 2 |
| PHI:445 | 1 | PHI:441 | 29 | PHI:441 | 43 | PHI:442 | 1 | PHI:444 | 1 | PHI:451 | 1 |
| PHI:446 | 1 | PHI:442 | 1 | PHI:442 | 1 | PHI:443 | 7 | PHI:445 | 1 | PHI:454 | 1 |
| PHI:447 | 2 | PHI:443 | 7 | PHI:443 | 7 | PHI:444 | 1 | PHI:446 | 1 | PHI:455 | 2 |
| PHI:451 | 1 | PHI:444 | 1 | PHI:444 | 1 | PHI:445 | 1 | PHI:447 | 3 | PHI:463 | 1 |
| PHI:454 | 1 | PHI:445 | 1 | PHI:445 | 1 | PHI:446 | 1 | PHI:449 | 1 | PHI:464 | 1 |
| PHI:455 | 2 | PHI:446 | 1 | PHI:446 | 1 | PHI:447 | 2 | PHI:451 | 1 | PHI:465 | 9 |
| PHI:461 | 1 | PHI:447 | 3 | PHI:447 | 2 | PHI:449 | 1 | PHI:454 | 1 | PHI:467 | 1 |
| PHI:463 | 1 | PHI:449 | 1 | PHI:449 | 1 | PHI:451 | 1 | PHI:455 | 2 | PHI:469 | 2 |
| PHI:464 | 1 | PHI:451 | 1 | PHI:451 | 1 | PHI:454 | 1 | PHI:463 | 1 | PHI:470 | 1 |
| PHI:465 | 10 | PHI:454 | 1 | PHI:454 | 1 | PHI:455 | 2 | PHI:464 | 1 | PHI:474 | 2 |
| PHI:469 | 2 | PHI:455 | 2 | PHI:455 | 2 | PHI:461 | 1 | PHI:465 | 9 | PHI:475 | 1 |
| PHI:470 | 2 | PHI:463 | 1 | PHI:461 | 2 | PHI:463 | 1 | PHI:469 | 1 | PHI:479 | 1 |
| PHI:474 | 2 | PHI:464 | 1 | PHI:463 | 1 | PHI:464 | 1 | PHI:470 | 3 | PHI:481 | 1 |
| PHI:475 | 1 | PHI:465 | 9 | PHI:464 | 1 | PHI:465 | 10 | PHI:474 | 3 | PHI:482 | 13 |
| PHI:476 | 1 | PHI:469 | 1 | PHI:465 | 10 | PHI:469 | 2 | PHI:475 | 2 | PHI:485 | 1 |
| PHI:479 | 1 | PHI:470 | 3 | PHI:469 | 2 | PHI:470 | 2 | PHI:476 | 2 | PHI:486 | 1 |
| PHI:481 | 1 | PHI:474 | 4 | PHI:470 | 2 | PHI:474 | 2 | PHI:477 | 1 | PHI:488 | 2 |
| PHI:482 | 14 | PHI:475 | 1 | PHI:474 | 2 | PHI:475 | 1 | PHI:479 | 1 | PHI:489 | 1 |
| PHI:485 | 2 | PHI:476 | 1 | PHI:475 | 1 | PHI:476 | 1 | PHI:481 | 1 | PHI:491 | 1 |
| PHI:486 | 1 | PHI:477 | 1 | PHI:476 | 1 | PHI:479 | 1 | PHI:482 | 12 | PHI:493 | 2 |
| PHI:488 | 2 | PHI:479 | 1 | PHI:479 | 1 | PHI:481 | 1 | PHI:485 | 1 | PHI:494 | 1 |
| PHI:489 | 1 | PHI:481 | 1 | PHI:481 | 1 | PHI:482 | 16 | PHI:486 | 1 | PHI:496 | 1 |
| PHI:491 | 1 | PHI:482 | 11 | PHI:482 | 15 | PHI:485 | 2 | PHI:488 | 2 | PHI:497 | 1 |
| PHI:493 | 2 | PHI:485 | 1 | PHI:485 | 2 | PHI:486 | 1 | PHI:489 | 1 | PHI:500 | 3 |
| PHI:494 | 1 | PHI:486 | 1 | PHI:486 | 1 | PHI:488 | 2 | PHI:491 | 2 | PHI:501 | 1 |
| PHI:496 | 1 | PHI:488 | 2 | PHI:488 | 2 | PHI:489 | 1 | PHI:493 | 2 | PHI:502 | 2 |
| PHI:497 | 1 | PHI:489 | 1 | PHI:489 | 1 | PHI:491 | 1 | PHI:494 | 1 | PHI:503 | 1 |
| PHI:500 | 7 | PHI:491 | 2 | PHI:491 | 1 | PHI:493 | 2 | PHI:496 | 1 | PHI:504 | 5 |
| PHI:501 | 1 | PHI:493 | 2 | PHI:493 | 2 | PHI:494 | 1 | PHI:497 | 4 | PHI:505 | 1 |
| PHI:502 | 2 | PHI:494 | 1 | PHI:494 | 1 | PHI:496 | 1 | PHI:500 | 2 | PHI:506 | 2 |
| PHI:503 | 1 | PHI:496 | 1 | PHI:496 | 1 | PHI:497 | 1 | PHI:501 | 1 | PHI:508 | 4 |
| PHI:504 | 7 | PHI:497 | 6 | PHI:497 | 1 | PHI:500 | 7 | PHI:502 | 2 | PHI:509 | 7 |
| PHI:505 | 1 | PHI:500 | 3 | PHI:500 | 7 | PHI:501 | 1 | PHI:503 | 1 | PHI:510 | 6 |
| PHI:506 | 2 | PHI:501 | 1 | PHI:501 | 1 | PHI:502 | 2 | PHI:504 | 6 | PHI:511 | 27 |
| PHI:507 | 1 | PHI:502 | 1 | PHI:502 | 2 | PHI:503 | 1 | PHI:505 | 1 | PHI:512 | 1 |
| PHI:508 | 7 | PHI:503 | 1 | PHI:503 | 1 | PHI:504 | 7 | PHI:506 | 2 | PHI:513 | 8 |
| PHI:509 | 10 | PHI:504 | 6 | PHI:504 | 7 | PHI:505 | 1 | PHI:507 | 1 | PHI:517 | 2 |
| PHI:510 | 21 | PHI:505 | 1 | PHI:505 | 1 | PHI:506 | 2 | PHI:508 | 7 | PHI:518 | 3 |
| PHI:511 | 38 | PHI:506 | 2 | PHI:506 | 2 | PHI:507 | 1 | PHI:509 | 8 | PHI:519 | 2 |
| PHI:512 | 1 | PHI:507 | 1 | PHI:507 | 1 | PHI:508 | 8 | PHI:510 | 16 | PHI:52 | 1 |
| PHI:513 | 15 | PHI:508 | 8 | PHI:508 | 8 | PHI:509 | 11 | PHI:511 | 30 | PHI:520 | 1 |
| PHI:518 | 3 | PHI:509 | 8 | PHI:509 | 11 | PHI:510 | 22 | PHI:512 | 1 | PHI:522 | 1 |
| PHI:519 | 2 | PHI:510 | 13 | PHI:510 | 22 | PHI:511 | 39 | PHI:513 | 9 | PHI:524 | 1 |
| PHI:52 | 1 | PHI:511 | 30 | PHI:511 | 40 | PHI:512 | 1 | PHI:517 | 2 | PHI:528 | 3 |
| PHI:520 | 1 | PHI:512 | 1 | PHI:512 | 1 | PHI:513 | 15 | PHI:518 | 5 | PHI:538 | 55 |
| PHI:522 | 1 | PHI:513 | 8 | PHI:513 | 15 | PHI:517 | 1 | PHI:519 | 1 | PHI:541 | 13 |
| PHI:524 | 1 | PHI:517 | 2 | PHI:518 | 3 | PHI:518 | 3 | PHI:52 | 1 | PHI:542 | 1 |
| PHI:528 | 2 | PHI:518 | 4 | PHI:519 | 2 | PHI:519 | 2 | PHI:520 | 1 | PHI:543 | 4 |
| PHI:538 | 106 | PHI:520 | 1 | PHI:52 | 1 | PHI:52 | 1 | PHI:522 | 1 | PHI:544 | 21 |
| PHI:541 | 31 | PHI:522 | 1 | PHI:520 | 1 | PHI:520 | 1 | PHI:524 | 1 | PHI:546 | 1 |
| PHI:542 | 1 | PHI:524 | 1 | PHI:522 | 1 | PHI:522 | 1 | PHI:528 | 3 | PHI:547 | 3 |
| PHI:543 | 4 | PHI:528 | 2 | PHI:524 | 1 | PHI:524 | 1 | PHI:538 | 62 | PHI:548 | 4 |
| PHI:544 | 64 | PHI:538 | 60 | PHI:528 | 2 | PHI:528 | 2 | PHI:541 | 15 | PHI:55 | 18 |
| PHI:546 | 1 | PHI:541 | 16 | PHI:538 | 111 | PHI:538 | 110 | PHI:542 | 1 | PHI:550 | 1 |
| PHI:547 | 4 | PHI:542 | 1 | PHI:541 | 33 | PHI:541 | 33 | PHI:543 | 4 | PHI:552 | 4 |
| PHI:548 | 4 | PHI:543 | 4 | PHI:542 | 1 | PHI:542 | 1 | PHI:544 | 33 | PHI:553 | 3 |
| PHI:55 | 26 | PHI:544 | 32 | PHI:543 | 4 | PHI:543 | 4 | PHI:546 | 1 | PHI:564 | 2 |
| PHI:550 | 1 | PHI:546 | 1 | PHI:544 | 66 | PHI:544 | 65 | PHI:547 | 2 | PHI:565 | 1 |
| PHI:552 | 4 | PHI:547 | 2 | PHI:546 | 1 | PHI:546 | 1 | PHI:548 | 4 | PHI:566 | 2 |
| PHI:553 | 4 | PHI:548 | 4 | PHI:547 | 4 | PHI:547 | 4 | PHI:55 | 19 | PHI:568 | 1 |
| PHI:562 | 4 | PHI:55 | 20 | PHI:548 | 4 | PHI:548 | 4 | PHI:550 | 2 | PHI:57 | 1 |
| PHI:564 | 2 | PHI:550 | 1 | PHI:55 | 27 | PHI:55 | 27 | PHI:551 | 3 | PHI:577 | 3 |
| PHI:565 | 1 | PHI:551 | 2 | PHI:550 | 1 | PHI:550 | 1 | PHI:552 | 2 | PHI:58 | 1 |
| PHI:566 | 2 | PHI:552 | 2 | PHI:552 | 4 | PHI:552 | 4 | PHI:553 | 4 | PHI:587 | 4 |
| PHI:568 | 1 | PHI:553 | 5 | PHI:553 | 4 | PHI:553 | 4 | PHI:562 | 2 | PHI:59 | 19 |
| PHI:569 | 2 | PHI:562 | 2 | PHI:562 | 4 | PHI:562 | 4 | PHI:564 | 2 | PHI:591 | 1 |
| PHI:57 | 1 | PHI:564 | 2 | PHI:564 | 2 | PHI:564 | 2 | PHI:565 | 1 | PHI:593 | 2 |
| PHI:577 | 5 | PHI:565 | 1 | PHI:565 | 1 | PHI:565 | 1 | PHI:566 | 2 | PHI:594 | 2 |
| PHI:58 | 2 | PHI:566 | 2 | PHI:566 | 2 | PHI:566 | 2 | PHI:569 | 2 | PHI:595 | 4 |
| PHI:587 | 5 | PHI:569 | 3 | PHI:568 | 1 | PHI:568 | 1 | PHI:57 | 1 | PHI:598 | 8 |
| PHI:59 | 48 | PHI:57 | 1 | PHI:569 | 2 | PHI:569 | 2 | PHI:577 | 2 | PHI:599 | 1 |
| PHI:591 | 1 | PHI:577 | 2 | PHI:57 | 1 | PHI:57 | 1 | PHI:58 | 1 | PHI:60 | 1 |
| PHI:593 | 2 | PHI:58 | 1 | PHI:577 | 4 | PHI:577 | 4 | PHI:587 | 5 | PHI:600 | 1 |
| PHI:594 | 2 | PHI:587 | 5 | PHI:58 | 2 | PHI:58 | 2 | PHI:59 | 24 | PHI:61 | 2 |
| PHI:595 | 5 | PHI:59 | 26 | PHI:587 | 5 | PHI:587 | 5 | PHI:591 | 1 | PHI:612 | 1 |
| PHI:598 | 7 | PHI:591 | 1 | PHI:59 | 48 | PHI:59 | 48 | PHI:593 | 2 | PHI:616 | 1 |
| PHI:599 | 2 | PHI:593 | 2 | PHI:591 | 1 | PHI:591 | 1 | PHI:594 | 2 | PHI:62 | 1 |
| PHI:60 | 2 | PHI:594 | 2 | PHI:593 | 3 | PHI:593 | 2 | PHI:595 | 4 | PHI:624 | 2 |
| PHI:600 | 1 | PHI:595 | 4 | PHI:594 | 2 | PHI:594 | 2 | PHI:598 | 7 | PHI:645 | 2 |
| PHI:601 | 5 | PHI:598 | 7 | PHI:595 | 5 | PHI:595 | 4 | PHI:599 | 1 | PHI:65 | 2 |
| PHI:61 | 2 | PHI:599 | 1 | PHI:598 | 8 | PHI:598 | 8 | PHI:600 | 1 | PHI:650 | 1 |
| PHI:612 | 2 | PHI:60 | 1 | PHI:599 | 2 | PHI:599 | 2 | PHI:601 | 2 | PHI:651 | 1 |
| PHI:616 | 1 | PHI:600 | 1 | PHI:60 | 2 | PHI:60 | 2 | PHI:61 | 2 | PHI:652 | 1 |
| PHI:624 | 1 | PHI:601 | 2 | PHI:600 | 1 | PHI:600 | 1 | PHI:612 | 3 | PHI:66 | 1 |
| PHI:645 | 3 | PHI:61 | 2 | PHI:601 | 7 | PHI:601 | 7 | PHI:616 | 1 | PHI:667 | 1 |
| PHI:65 | 2 | PHI:612 | 2 | PHI:61 | 2 | PHI:61 | 2 | PHI:62 | 1 | PHI:668 | 4 |
| PHI:650 | 1 | PHI:616 | 1 | PHI:612 | 2 | PHI:612 | 2 | PHI:624 | 1 | PHI:67 | 1 |
| PHI:651 | 1 | PHI:62 | 1 | PHI:616 | 1 | PHI:616 | 1 | PHI:645 | 3 | PHI:673 | 1 |
| PHI:667 | 1 | PHI:624 | 2 | PHI:62 | 1 | PHI:62 | 1 | PHI:65 | 1 | PHI:674 | 1 |
| PHI:668 | 12 | PHI:645 | 2 | PHI:624 | 1 | PHI:624 | 1 | PHI:650 | 2 | PHI:675 | 2 |
| PHI:67 | 1 | PHI:65 | 2 | PHI:645 | 3 | PHI:645 | 3 | PHI:651 | 1 | PHI:677 | 1 |
| PHI:673 | 1 | PHI:650 | 1 | PHI:65 | 2 | PHI:65 | 2 | PHI:653 | 1 | PHI:69 | 1 |
| PHI:674 | 1 | PHI:651 | 1 | PHI:650 | 1 | PHI:650 | 1 | PHI:66 | 1 | PHI:693 | 1 |
| PHI:675 | 2 | PHI:653 | 1 | PHI:651 | 1 | PHI:651 | 1 | PHI:667 | 1 | PHI:694 | 2 |
| PHI:69 | 1 | PHI:66 | 1 | PHI:66 | 1 | PHI:66 | 1 | PHI:668 | 5 | PHI:695 | 1 |
| PHI:693 | 1 | PHI:667 | 1 | PHI:667 | 1 | PHI:667 | 1 | PHI:67 | 5 | PHI:697 | 5 |
| PHI:694 | 2 | PHI:668 | 5 | PHI:668 | 12 | PHI:668 | 12 | PHI:673 | 1 | PHI:703 | 1 |
| PHI:695 | 1 | PHI:67 | 4 | PHI:67 | 1 | PHI:67 | 1 | PHI:674 | 1 | PHI:714 | 2 |
| PHI:697 | 6 | PHI:673 | 1 | PHI:673 | 1 | PHI:673 | 1 | PHI:675 | 2 | PHI:716 | 5 |
| PHI:713 | 1 | PHI:674 | 1 | PHI:674 | 1 | PHI:674 | 1 | PHI:678 | 1 | PHI:731 | 1 |
| PHI:714 | 10 | PHI:675 | 2 | PHI:675 | 2 | PHI:675 | 2 | PHI:69 | 1 | PHI:734 | 30 |
| PHI:716 | 15 | PHI:680 | 1 | PHI:679 | 1 | PHI:69 | 1 | PHI:693 | 1 | PHI:737 | 17 |
| PHI:728 | 1 | PHI:69 | 1 | PHI:69 | 1 | PHI:693 | 1 | PHI:695 | 1 | PHI:741 | 1 |
| PHI:731 | 2 | PHI:690 | 1 | PHI:693 | 1 | PHI:694 | 2 | PHI:697 | 6 | PHI:748 | 7 |
| PHI:734 | 56 | PHI:693 | 1 | PHI:694 | 2 | PHI:695 | 1 | PHI:703 | 1 | PHI:777 | 2 |
| PHI:737 | 18 | PHI:695 | 1 | PHI:695 | 1 | PHI:697 | 6 | PHI:714 | 3 | PHI:781 | 4 |
| PHI:741 | 1 | PHI:697 | 5 | PHI:697 | 6 | PHI:703 | 1 | PHI:716 | 10 | PHI:783 | 1 |
| PHI:748 | 12 | PHI:703 | 1 | PHI:703 | 1 | PHI:713 | 1 | PHI:731 | 1 | PHI:784 | 15 |
| PHI:777 | 2 | PHI:714 | 3 | PHI:713 | 1 | PHI:714 | 10 | PHI:734 | 40 | PHI:785 | 1 |
| PHI:781 | 2 | PHI:716 | 11 | PHI:714 | 10 | PHI:716 | 16 | PHI:737 | 17 | PHI:789 | 2 |
| PHI:783 | 1 | PHI:731 | 1 | PHI:716 | 16 | PHI:728 | 1 | PHI:741 | 1 | PHI:791 | 1 |
| PHI:784 | 36 | PHI:734 | 43 | PHI:728 | 1 | PHI:731 | 2 | PHI:748 | 6 | PHI:792 | 2 |
| PHI:785 | 1 | PHI:737 | 14 | PHI:731 | 2 | PHI:734 | 54 | PHI:76 | 1 | PHI:795 | 1 |
| PHI:789 | 3 | PHI:741 | 1 | PHI:734 | 56 | PHI:737 | 18 | PHI:777 | 2 | PHI:796 | 2 |
| PHI:791 | 1 | PHI:748 | 6 | PHI:737 | 19 | PHI:741 | 1 | PHI:781 | 5 | PHI:797 | 2 |
| PHI:792 | 2 | PHI:777 | 2 | PHI:741 | 1 | PHI:748 | 13 | PHI:783 | 1 | PHI:799 | 3 |
| PHI:795 | 1 | PHI:781 | 5 | PHI:748 | 12 | PHI:777 | 2 | PHI:784 | 25 | PHI:80 | 1 |
| PHI:796 | 2 | PHI:783 | 1 | PHI:777 | 2 | PHI:781 | 4 | PHI:785 | 1 | PHI:800 | 1 |
| PHI:797 | 2 | PHI:784 | 24 | PHI:781 | 4 | PHI:783 | 1 | PHI:789 | 2 | PHI:801 | 1 |
| PHI:799 | 3 | PHI:785 | 1 | PHI:783 | 1 | PHI:784 | 37 | PHI:791 | 1 | PHI:802 | 2 |
| PHI:80 | 1 | PHI:789 | 2 | PHI:784 | 37 | PHI:785 | 1 | PHI:792 | 2 | PHI:803 | 2 |
| PHI:800 | 1 | PHI:791 | 1 | PHI:785 | 1 | PHI:789 | 3 | PHI:795 | 1 | PHI:804 | 1 |
| PHI:801 | 1 | PHI:792 | 2 | PHI:789 | 3 | PHI:791 | 1 | PHI:796 | 2 | PHI:805 | 1 |
| PHI:802 | 1 | PHI:795 | 1 | PHI:791 | 1 | PHI:792 | 2 | PHI:797 | 2 | PHI:806 | 1 |
| PHI:803 | 2 | PHI:796 | 2 | PHI:792 | 2 | PHI:795 | 1 | PHI:799 | 3 | PHI:807 | 1 |
| PHI:804 | 1 | PHI:797 | 2 | PHI:795 | 1 | PHI:796 | 2 | PHI:80 | 1 | PHI:808 | 1 |
| PHI:805 | 1 | PHI:799 | 3 | PHI:796 | 2 | PHI:797 | 2 | PHI:800 | 2 | PHI:81 | 5 |
| PHI:806 | 1 | PHI:80 | 1 | PHI:797 | 2 | PHI:799 | 3 | PHI:801 | 1 | PHI:810 | 1 |
| PHI:807 | 1 | PHI:800 | 2 | PHI:799 | 3 | PHI:80 | 1 | PHI:802 | 2 | PHI:811 | 2 |
| PHI:808 | 1 | PHI:801 | 1 | PHI:80 | 1 | PHI:800 | 1 | PHI:803 | 2 | PHI:812 | 12 |
| PHI:81 | 4 | PHI:802 | 2 | PHI:800 | 1 | PHI:801 | 1 | PHI:804 | 1 | PHI:815 | 1 |
| PHI:810 | 1 | PHI:803 | 2 | PHI:801 | 1 | PHI:802 | 2 | PHI:805 | 1 | PHI:816 | 3 |
| PHI:811 | 2 | PHI:804 | 1 | PHI:802 | 2 | PHI:803 | 2 | PHI:806 | 1 | PHI:817 | 1 |
| PHI:812 | 31 | PHI:805 | 1 | PHI:803 | 2 | PHI:804 | 1 | PHI:807 | 1 | PHI:819 | 1 |
| PHI:815 | 1 | PHI:806 | 1 | PHI:804 | 1 | PHI:805 | 1 | PHI:808 | 1 | PHI:820 | 2 |
| PHI:816 | 3 | PHI:807 | 2 | PHI:805 | 1 | PHI:806 | 1 | PHI:81 | 4 | PHI:822 | 1 |
| PHI:817 | 4 | PHI:808 | 1 | PHI:806 | 1 | PHI:807 | 1 | PHI:810 | 1 | PHI:823 | 2 |
| PHI:820 | 1 | PHI:81 | 4 | PHI:807 | 1 | PHI:808 | 1 | PHI:811 | 2 | PHI:830 | 1 |
| PHI:822 | 1 | PHI:810 | 1 | PHI:808 | 1 | PHI:81 | 4 | PHI:812 | 17 | PHI:831 | 1 |
| PHI:823 | 2 | PHI:811 | 2 | PHI:81 | 4 | PHI:810 | 1 | PHI:815 | 1 | PHI:835 | 1 |
| PHI:825 | 1 | PHI:812 | 20 | PHI:810 | 1 | PHI:811 | 2 | PHI:816 | 3 | PHI:837 | 1 |
| PHI:830 | 1 | PHI:815 | 1 | PHI:811 | 2 | PHI:812 | 31 | PHI:817 | 5 | PHI:838 | 2 |
| PHI:831 | 1 | PHI:816 | 3 | PHI:812 | 31 | PHI:815 | 1 | PHI:819 | 1 | PHI:84 | 2 |
| PHI:837 | 2 | PHI:817 | 3 | PHI:815 | 1 | PHI:816 | 3 | PHI:820 | 1 | PHI:842 | 1 |
| PHI:838 | 2 | PHI:819 | 1 | PHI:816 | 3 | PHI:817 | 6 | PHI:822 | 1 | PHI:843 | 3 |
| PHI:839 | 1 | PHI:820 | 1 | PHI:817 | 5 | PHI:819 | 1 | PHI:823 | 4 | PHI:845 | 1 |
| PHI:84 | 1 | PHI:822 | 1 | PHI:819 | 1 | PHI:820 | 1 | PHI:830 | 1 | PHI:846 | 4 |
| PHI:841 | 2 | PHI:823 | 3 | PHI:820 | 1 | PHI:822 | 1 | PHI:831 | 1 | PHI:849 | 1 |
| PHI:843 | 2 | PHI:830 | 2 | PHI:822 | 1 | PHI:823 | 3 | PHI:835 | 1 | PHI:853 | 3 |
| PHI:846 | 5 | PHI:831 | 1 | PHI:823 | 3 | PHI:825 | 1 | PHI:838 | 2 | PHI:854 | 2 |
| PHI:853 | 3 | PHI:837 | 1 | PHI:825 | 1 | PHI:830 | 1 | PHI:84 | 1 | PHI:856 | 1 |
| PHI:854 | 3 | PHI:838 | 2 | PHI:830 | 1 | PHI:831 | 1 | PHI:841 | 2 | PHI:857 | 1 |
| PHI:856 | 1 | PHI:84 | 1 | PHI:831 | 1 | PHI:837 | 2 | PHI:843 | 1 | PHI:858 | 1 |
| PHI:857 | 1 | PHI:842 | 1 | PHI:837 | 2 | PHI:838 | 2 | PHI:846 | 3 | PHI:860 | 4 |
| PHI:858 | 1 | PHI:843 | 2 | PHI:838 | 2 | PHI:839 | 2 | PHI:849 | 1 | PHI:862 | 2 |
| PHI:859 | 2 | PHI:844 | 1 | PHI:839 | 1 | PHI:84 | 1 | PHI:853 | 2 | PHI:864 | 6 |
| PHI:860 | 4 | PHI:846 | 2 | PHI:84 | 1 | PHI:841 | 1 | PHI:854 | 2 | PHI:867 | 4 |
| PHI:862 | 2 | PHI:849 | 1 | PHI:841 | 2 | PHI:843 | 3 | PHI:856 | 1 | PHI:868 | 1 |
| PHI:864 | 7 | PHI:853 | 1 | PHI:843 | 2 | PHI:844 | 1 | PHI:857 | 1 | PHI:872 | 3 |
| PHI:867 | 4 | PHI:854 | 2 | PHI:844 | 1 | PHI:846 | 5 | PHI:858 | 1 | PHI:873 | 1 |
| PHI:87 | 1 | PHI:856 | 1 | PHI:846 | 5 | PHI:849 | 1 | PHI:860 | 4 | PHI:875 | 1 |
| PHI:872 | 2 | PHI:857 | 1 | PHI:849 | 1 | PHI:853 | 3 | PHI:862 | 2 | PHI:876 | 19 |
| PHI:873 | 1 | PHI:858 | 1 | PHI:853 | 3 | PHI:854 | 3 | PHI:864 | 6 | PHI:877 | 1 |
| PHI:875 | 1 | PHI:860 | 4 | PHI:854 | 3 | PHI:856 | 1 | PHI:867 | 4 | PHI:881 | 16 |
| PHI:876 | 2 | PHI:862 | 2 | PHI:856 | 1 | PHI:857 | 1 | PHI:87 | 1 | PHI:882 | 5 |
| PHI:877 | 2 | PHI:864 | 6 | PHI:857 | 1 | PHI:858 | 1 | PHI:872 | 2 | PHI:885 | 1 |
| PHI:881 | 39 | PHI:867 | 4 | PHI:858 | 1 | PHI:859 | 1 | PHI:873 | 1 | PHI:886 | 4 |
| PHI:882 | 3 | PHI:87 | 1 | PHI:859 | 1 | PHI:860 | 7 | PHI:874 | 1 | PHI:887 | 1 |
| PHI:887 | 1 | PHI:872 | 2 | PHI:860 | 6 | PHI:862 | 2 | PHI:875 | 1 | PHI:888 | 1 |
| PHI:888 | 1 | PHI:873 | 1 | PHI:862 | 2 | PHI:864 | 6 | PHI:876 | 1 | PHI:889 | 8 |
| PHI:889 | 7 | PHI:874 | 2 | PHI:864 | 6 | PHI:867 | 5 | PHI:877 | 1 | PHI:89 | 2 |
| PHI:89 | 3 | PHI:875 | 1 | PHI:867 | 5 | PHI:87 | 1 | PHI:881 | 21 | PHI:890 | 3 |
| PHI:890 | 3 | PHI:876 | 1 | PHI:87 | 1 | PHI:872 | 2 | PHI:882 | 5 | PHI:891 | 1 |
| PHI:891 | 2 | PHI:877 | 1 | PHI:872 | 2 | PHI:873 | 1 | PHI:885 | 1 | PHI:893 | 3 |
| PHI:893 | 3 | PHI:881 | 21 | PHI:873 | 1 | PHI:875 | 1 | PHI:887 | 1 | PHI:901 | 4 |
| PHI:901 | 7 | PHI:882 | 5 | PHI:875 | 1 | PHI:876 | 3 | PHI:888 | 1 | PHI:903 | 3 |
| PHI:903 | 4 | PHI:885 | 1 | PHI:877 | 2 | PHI:877 | 2 | PHI:889 | 5 | PHI:911 | 3 |
| PHI:911 | 3 | PHI:887 | 1 | PHI:881 | 39 | PHI:881 | 40 | PHI:89 | 2 | PHI:922 | 14 |
| PHI:922 | 21 | PHI:888 | 1 | PHI:882 | 4 | PHI:882 | 4 | PHI:890 | 2 | PHI:923 | 3 |
| PHI:923 | 3 | PHI:889 | 9 | PHI:887 | 1 | PHI:887 | 1 | PHI:891 | 2 | PHI:96 | 2 |
| PHI:96 | 5 | PHI:89 | 2 | PHI:888 | 1 | PHI:888 | 1 | PHI:893 | 3 | PHI:97 | 2 |
| PHI:97 | 3 | PHI:890 | 1 | PHI:889 | 8 | PHI:889 | 9 | PHI:901 | 6 | PHI:981 | 12 |
| PHI:981 | 9 | PHI:891 | 3 | PHI:89 | 3 | PHI:89 | 3 | PHI:903 | 4 |  |  |
|  |  | PHI:893 | 3 | PHI:890 | 3 | PHI:890 | 3 | PHI:911 | 3 |  |  |
|  |  | PHI:901 | 6 | PHI:891 | 2 | PHI:891 | 2 | PHI:922 | 16 |  |  |
|  |  | PHI:903 | 4 | PHI:893 | 3 | PHI:893 | 3 | PHI:923 | 3 |  |  |
|  |  | PHI:911 | 3 | PHI:901 | 7 | PHI:901 | 7 | PHI:96 | 2 |  |  |
|  |  | PHI:922 | 16 | PHI:903 | 4 | PHI:903 | 4 | PHI:97 | 2 |  |  |
|  |  | PHI:923 | 3 | PHI:911 | 3 | PHI:911 | 3 | PHI:981 | 12 |  |  |
|  |  | PHI:96 | 2 | PHI:922 | 22 | PHI:922 | 21 |  |  |  |  |
|  |  | PHI:97 | 2 | PHI:923 | 3 | PHI:923 | 3 |  |  |  |  |
|  |  | PHI:981 | 12 | PHI:96 | 4 | PHI:96 | 4 |  |  |  |  |
|  |  |  |  | PHI:97 | 3 | PHI:97 | 3 |  |  |  |  |
|  |  |  |  | PHI:981 | 11 | PHI:981 | 11 |  |  |  |  |
| Total | 2155 | Total | 1728 | Total | 2245 | Total | 2242 | Total | 1728 | Total | 1503 |
