# Supplementary material for: New insights into the evolution of host specificity of three Penicillium species and the pathogenicity of P. Italicum involving the infection of Valencia orange (Citrus sinensis)
Source: Virulence. 2020 Jun 11;11(1):748–68. doi: 10.1080/21505594.2020.1773038 (PMC7549954; doi:10.1080/21505594.2020.1773038)
Supplement: Supplemental Material [file KVIR_A_1773038_SM2584.zip › Dataset S3.docx]

**Dataset** **S3** Expression of effector genes encoding CAZymes (A), GPCR (B), PHI (C), SSCP (D), secondary metabolism (SM)-related genes (E), speciation specific genes (F) in the *P. italicum* GL-Gan1 during the colonization of [Valencia orange](http://www.baidu.com/link?url=kHVVlo3thXkUrFrCgTimkoQHEzkfOO8xSGZies5qPeTcuPmUgL2pY0V7MFatpW8-ubOSAuHW9EqLBBz7UDz94xKdXX7bUJ7QwU2XbpJF9qv5OgDPWeSrQY0GWgTt-ikv&wd=&eqid=a3352eeb0000d7b0000000065c9c2b87)

**(A)** CAZymes

| Type | Gene ID | Expression Means | | | | |
| --- | --- | --- | --- | --- | --- | --- |
|  |  | GL0d | GL1d | GL3d | GL5d | GL10d |
| CE8 | GL_Gan1_GLEAN_10008717 | 0 | 2.345 | 1.33 | 0.99 | 1.43 |
|  | GL_Gan1_GLEAN_10003242 | 1.815 | 76.34 | 11.235 | 6.68 | 75.77 |
| CE12 | GL_Gan1_GLEAN_10004496 | 22.045 | 17.255 | 21.08 | 18.755 | 16.77 |
|  | GL_Gan1_GLEAN_10001636 | 30.385 | 35.645 | 13.91 | 46.06 | 13.595 |
|  | GL_Gan1_GLEAN_10002850 | 29.31 | 21.27 | 15.48 | 13.405 | 19.54 |
|  | GL_Gan1_GLEAN_10005653 | 94.555 | 516.55 | 344.13 | 336.82 | 371.83 |
|  | GL_Gan1_GLEAN_10005459 | 0 | 0.255 | 0.875 | 0.805 | 0 |
|  | GL_Gan1_GLEAN_10005573 | 0 | 0.67 | 0.15 | 0 | 0 |
| GH28 | GL_Gan1_GLEAN_10007452 | 4.04 | 15.875 | 12.085 | 9.71 | 12.4 |
|  | GL_Gan1_GLEAN_10009400 | 111.055 | 149.565 | 157.49 | 164.485 | 168.01 |
|  | GL_Gan1_GLEAN_10008929 | 37.41 | 22.455 | 26.665 | 25.645 | 21.15 |
|  | GL_Gan1_GLEAN_10008681 | 25.75 | 678.31 | 124.365 | 68.44 | 540.375 |
|  | GL_Gan1_GLEAN_10008705 | 7.16 | 13.73 | 0.575 | 1.04 | 10.805 |
|  | GL_Gan1_GLEAN_10008734 | 0 | 0 | 0.23 | 0.09 | 0 |
|  | GL_Gan1_GLEAN_10003694 | 25.765 | 5.655 | 4.61 | 3.74 | 4.96 |
|  | GL_Gan1_GLEAN_10002646 | 24.44 | 18.34 | 20.025 | 17.95 | 19.975 |
|  | GL_Gan1_GLEAN_10004959 | 3.155 | 13.655 | 6.955 | 7.49 | 21.455 |
|  | GL_Gan1_GLEAN_10003071 | 21.5 | 9.9 | 10.61 | 10.74 | 11.09 |
|  | GL_Gan1_GLEAN_10004367 | 35.95 | 38.655 | 37.585 | 38.55 | 32.215 |
|  | GL_Gan1_GLEAN_10003348 | 0 | 0.915 | 3.45 | 4.13 | 0.5 |
|  | GL_Gan1_GLEAN_10004813 | 12.71 | 4.365 | 5.015 | 2.795 | 5.71 |
|  | GL_Gan1_GLEAN_10006306 | 8.64 | 16.76 | 9.665 | 13.87 | 16.04 |
|  | GL_Gan1_GLEAN_10005669 | 33.03 | 1030.445 | 916.41 | 1005.12 | 1108.905 |
|  | GL_Gan1_GLEAN_10006043 | 2.695 | 15.44 | 15.245 | 10.31 | 16.835 |
|  | GL_Gan1_GLEAN_10002967 | 36.36 | 31.86 | 42.425 | 47.88 | 40.5 |
| GH78 | GL_Gan1_GLEAN_10008298 | 0 | 1.965 | 4.245 | 1.01 | 1.255 |
|  | GL_Gan1_GLEAN_10008492 | 0.77 | 0 | 0.295 | 0.275 | 0.275 |
|  | GL_Gan1_GLEAN_10001164 | 21.815 | 5.905 | 6.75 | 5.87 | 6.09 |
|  | GL_Gan1_GLEAN_10001184 | 0 | 3.7 | 0.76 | 0.41 | 4.815 |
|  | GL_Gan1_GLEAN_10004512 | 0 | 1.17 | 0.315 | 1 | 1.295 |
|  | GL_Gan1_GLEAN_10000472 | 5.025 | 1.96 | 0.205 | 0.285 | 2.67 |
|  | GL_Gan1_GLEAN_10000281 | 38.33 | 12.915 | 22.42 | 16.655 | 12.11 |
|  | GL_Gan1_GLEAN_10003779 | 19.12 | 29.53 | 16.795 | 19.315 | 21.01 |
|  | GL_Gan1_GLEAN_10004312 | 0.81 | 6.76 | 10.145 | 4.07 | 7.84 |
|  | GL_Gan1_GLEAN_10004322 | 19.475 | 5.375 | 8.7 | 6.235 | 5.825 |
|  | GL_Gan1_GLEAN_10007487 | 5.99 | 105 | 6.84 | 6.505 | 9.885 |
|  | GL_Gan1_GLEAN_10001745 | 0 | 1.415 | 1.99 | 1.345 | 1.24 |
|  | GL_Gan1_GLEAN_10008155 | 44.595 | 31.06 | 11.89 | 15.015 | 31.375 |
|  | GL_Gan1_GLEAN_10000534 | 1.6 | 13.995 | 7.14 | 5.765 | 12.4 |
|  | GL_Gan1_GLEAN_10000552 | 4.81 | 1.24 | 5.53 | 3.075 | 4.185 |
|  | GL_Gan1_GLEAN_10009169 | 52.635 | 37.935 | 50.22 | 54.855 | 49.6 |
|  | GL_Gan1_GLEAN_10009232 | 0 | 2.215 | 0.2 | 0.185 | 0.535 |
|  | GL_Gan1_GLEAN_10001626 | 0 | 1.43 | 2.225 | 1.365 | 0.77 |
|  | GL_Gan1_GLEAN_10003174 | 0 | 19.965 | 1.23 | 6.505 | 23.3 |
|  | GL_Gan1_GLEAN_10009144 | 0.745 | 60.165 | 9.46 | 34.335 | 50.77 |
|  | GL_Gan1_GLEAN_10008674 | 0 | 0 | 0.11 | 0.33 | 0.205 |
|  | GL_Gan1_GLEAN_10008706 | 0 | 11.785 | 0.815 | 2.74 | 5.545 |
|  | GL_Gan1_GLEAN_10008742 | 0 | 1.22 | 0.57 | 0.775 | 1.7 |
|  | GL_Gan1_GLEAN_10001901 | 137.75 | 46.195 | 25.16 | 20.075 | 53.83 |
|  | GL_Gan1_GLEAN_10000801 | 1.62 | 5.375 | 1.105 | 1.26 | 4.15 |
|  | GL_Gan1_GLEAN_10001368 | 0 | 1.51 | 0.145 | 0.045 | 1.995 |
|  | GL_Gan1_GLEAN_10001372 | 0 | 0.265 | 0 | 0 | 0 |
|  | GL_Gan1_GLEAN_10006434 | 0 | 3.12 | 2.41 | 3.6 | 2.775 |
|  | GL_Gan1_GLEAN_10000367 | 0 | 1.015 | 0.545 | 0.31 | 0.3 |
|  | GL_Gan1_GLEAN_10003713 | 0 | 0.2 | 0.55 | 0.195 | 0 |
|  | GL_Gan1_GLEAN_10007295 | 26.44 | 17.48 | 21.495 | 19.72 | 21.14 |
|  | GL_Gan1_GLEAN_10002609 | 7.075 | 6.055 | 2.16 | 4.11 | 6.225 |
|  | GL_Gan1_GLEAN_10004712 | 66.515 | 37.29 | 30.11 | 29.525 | 32.545 |
|  | GL_Gan1_GLEAN_10004763 | 3.425 | 8.9 | 1.075 | 2.56 | 8.68 |
|  | GL_Gan1_GLEAN_10004932 | 15.84 | 2.815 | 3.095 | 3.69 | 2.665 |
|  | GL_Gan1_GLEAN_10001452 | 87.945 | 9.065 | 4.64 | 6.9 | 5.465 |
|  | GL_Gan1_GLEAN_10003029 | 0 | 0.31 | 0 | 0.28 | 0.31 |
|  | GL_Gan1_GLEAN_10002234 | 0 | 0.19 | 0.045 | 0.08 | 0.105 |
|  | GL_Gan1_GLEAN_10003331 | 2.315 | 9.315 | 6.72 | 2.83 | 8.255 |
|  | GL_Gan1_GLEAN_10000302 | 14.09 | 169.44 | 58.435 | 77.045 | 127.095 |
|  | GL_Gan1_GLEAN_10005436 | 0.79 | 5.9 | 5.4 | 7.405 | 7.25 |
|  | GL_Gan1_GLEAN_10003989 | 16.86 | 46.265 | 19.77 | 12.735 | 28.83 |
|  | GL_Gan1_GLEAN_10007095 | 66.755 | 103.595 | 94.14 | 76.35 | 103.545 |
|  | GL_Gan1_GLEAN_10007096 | 0.6 | 0.66 | 0.88 | 0.77 | 0.57 |
|  | GL_Gan1_GLEAN_10002016 | 5.25 | 14.92 | 9.025 | 10.765 | 12.44 |
|  | GL_Gan1_GLEAN_10005294 | 0.84 | 2.225 | 0 | 0.115 | 1.075 |
|  | GL_Gan1_GLEAN_10002144 | 5.275 | 7.725 | 10.34 | 20.78 | 8.54 |
|  | GL_Gan1_GLEAN_10000160 | 22.37 | 2.535 | 3.455 | 3.985 | 1.93 |
|  | GL_Gan1_GLEAN_10007776 | 3.175 | 3.51 | 4.995 | 4.06 | 4.485 |
|  | GL_Gan1_GLEAN_10006101 | 0 | 0.54 | 0.855 | 1.445 | 0.9 |
|  | GL_Gan1_GLEAN_10006128 | 40.22 | 40.92 | 56.22 | 36.2 | 45.935 |
|  | GL_Gan1_GLEAN_10002933 | 0.87 | 4.4 | 4.56 | 3.8 | 3.03 |
|  | GL_Gan1_GLEAN_10005823 | 0 | 0.2 | 0.52 | 0.35 | 0.345 |
|  | GL_Gan1_GLEAN_10003630 | 3.42 | 22.675 | 14.56 | 13.065 | 24.535 |
|  | GL_Gan1_GLEAN_10000427 | 13.18 | 27.97 | 9.855 | 38.045 | 17.785 |
|  | GL_Gan1_GLEAN_10006581 | 3.33 | 1.03 | 0.455 | 0.31 | 1.39 |
| GH95 | GL_Gan1_GLEAN_10001477 | 145.485 | 279.135 | 237.76 | 262.22 | 274.29 |
|  | GL_Gan1_GLEAN_10005127 | 0.55 | 0.485 | 0.465 | 0.32 | 0.285 |
|  | GL_Gan1_GLEAN_10005132 | 11.55 | 26.45 | 17.39 | 14.52 | 24.555 |
| GH105 | GL_Gan1_GLEAN_10002416 | 0 | 0.87 | 4.33 | 3.75 | 0 |
|  | GL_Gan1_GLEAN_10006994 | 3.325 | 2.21 | 6.365 | 5.15 | 4.09 |
|  | GL_Gan1_GLEAN_10007769 | 0 | 4.695 | 5.29 | 3.735 | 3.52 |
| PL1 | GL_Gan1_GLEAN_10007363 | 92.295 | 1427.52 | 1697.36 | 1267.27 | 1180.195 |
|  | GL_Gan1_GLEAN_10000542 | 19.845 | 10.38 | 5.535 | 7.075 | 6.475 |
|  | GL_Gan1_GLEAN_10009022 | 17.16 | 92.015 | 19.06 | 16.675 | 72.845 |
|  | GL_Gan1_GLEAN_10001454 | 34.13 | 22.655 | 15.345 | 15.48 | 19.625 |
|  | GL_Gan1_GLEAN_10006087 | 3.615 | 31.22 | 15.035 | 13.99 | 26.89 |
|  | GL_Gan1_GLEAN_10005466 | 0 | 0 | 0.925 | 0.375 | 0.735 |
| PL3 | GL_Gan1_GLEAN_10002598 | 0 | 48.685 | 1.48 | 1.39 | 20.695 |
| PL4 | GL_Gan1_GLEAN_10004450 | 4.17 | 3.375 | 3.12 | 6.325 | 2.54 |
|  | GL_Gan1_GLEAN_10002352 | 7.055 | 2.635 | 6.05 | 6.22 | 2.39 |
|  | GL_Gan1_GLEAN_10004325 | 0 | 0 | 0 | 3.95 | 0 |
|  | GL_Gan1_GLEAN_10003202 | 1.335 | 1.17 | 2.395 | 3.02 | 1.43 |
|  | GL_Gan1_GLEAN_10008590 | 22.66 | 24.94 | 26.94 | 22.83 | 26.735 |
|  | GL_Gan1_GLEAN_10002344 | 6.175 | 24.15 | 29.4 | 36.855 | 21.445 |
|  | GL_Gan1_GLEAN_10004420 | 1.23 | 5.14 | 3.375 | 2.675 | 3.695 |
|  | GL_Gan1_GLEAN_10007082 | 35.16 | 9.505 | 18.815 | 17.485 | 9.375 |
|  | GL_Gan1_GLEAN_10007724 | 16.145 | 45.155 | 35.53 | 29.345 | 46.545 |

**(B)** GPCR

| type | Gene ID | Expression Means | | | | |
| --- | --- | --- | --- | --- | --- | --- |
|  |  | GL0d | GL1d | GL3d | GL5d | GL10d |
| I (pheromone receptors) | GL_Gan1_GLEAN_10003614 | 3.235 | 5.71 | 5.47 | 6.88 | 7.5 |
| II (pheromone receptors) | GL_Gan1_GLEAN_10000918 | 4.91 | 4.225 | 2.18 | 4.275 | 2.205 |
|  | GL_Gan1_GLEAN_10000382 | 4.91 | 4.225 | 2.18 | 4.275 | 2.205 |
| III (related to *A*. *nidulan* GprC, GprD, and GprE) | - |  |  |  |  |  |
| IV (nitrogen sensors) | GL_Gan1_GLEAN_10007008 | 91.39 | 30.48 | 29.14 | 33.135 | 26.575 |
| V (cAMP receptor-like) | - |  |  |  |  |  |
| VI (GPCRs containing RGS domain) | GL_Gan1_GLEAN_10009444 | 4.4 | 37.41 | 44.625 | 38.795 | 45.645 |
| VII (related to rat growth hormone releasing factor) | - |  |  |  |  |  |
| VIII (related to human steroid receptor mPR) | GL_Gan1_GLEAN_10002686 | 0 | 1.17 | 1.475 | 1.415 | 2.42 |
|  | GL_Gan1_GLEAN_10006183 | 2.655 | 9.675 | 5.705 | 8.285 | 9.655 |
|  | GL_Gan1_GLEAN_10002116 | 33.7 | 39.295 | 65.98 | 48.67 | 35.01 |
| IX (microbial opsins) | GL_Gan1_GLEAN_10008789 | 0 | 0.32 | 0.94 | 1.34 | 0.4 |
|  | GL_Gan1_GLEAN_10002479 | 1582.995 | 123.61 | 175.975 | 245.04 | 88.425 |
| X (similar to PTM1) | GL_Gan1_GLEAN_10005748 | 59.46 | 83.17 | 72.69 | 82.91 | 95.65 |
| XI (similar to GPCR89) | - |  |  |  |  |  |
| XII (family C-like GPCRs) | GL_Gan1_GLEAN_10004881 | 0 | 0 | 0.26 | 0.21 | 0 |
| XIII (related to GPR11 of *P*. *sojae*) | - |  |  |  |  |  |
| PTH11-like | - |  |  |  |  |  |

**(C)** PHI

| gene ID | Pathogen species | Phenotype | Expression Means | | | | ` |
| --- | --- | --- | --- | --- | --- | --- | --- |
|  |  |  | GL0d | GL1d | GL3d | GL5d | GL10d |
| GL_Gan1_GLEAN_10000253 | Magnaporthe grisea | Reduced virulence | 4.125 | 5.495 | 5.645 | 6.865 | 6.385 |
| GL_Gan1_GLEAN_10003382 | Ustilago maydis | Reduced virulence | 11.355 | 4.84 | 4.42 | 5.755 | 4.045 |
| GL_Gan1_GLEAN_10003384 | Magnaporthe grisea | Reduced virulence | 47.995 | 31.43 | 28.82 | 32.885 | 28.6 |
| GL_Gan1_GLEAN_10003386 | Fusarium oxysporum | Reduced virulence | 282.11 | 73.405 | 47.325 | 51.665 | 59.1 |
| GL_Gan1_GLEAN_10003406 | Colletotrichum lagenarium | Loss of pathogenicity | 230.16 | 395.685 | 295.415 | 251.165 | 445.55 |
| GL_Gan1_GLEAN_10003407 | Cercospora nicotianae | Reduced virulence | 5.88 | 11.62 | 7.86 | 13.445 | 17.49 |
| GL_Gan1_GLEAN_10003430 | Magnaporthe grisea | Reduced virulence | 42.165 | 22.35 | 25.82 | 21.49 | 24.04 |
| GL_Gan1_GLEAN_10003432 | Claviceps purpurea | Reduced virulence | 34.95 | 15.465 | 10.045 | 9.99 | 15.36 |
| GL_Gan1_GLEAN_10003433 | Magnaporthe grisea | Reduced virulence | 19.235 | 8.475 | 10.485 | 11.81 | 11.33 |
| GL_Gan1_GLEAN_10003442 | Candida albicans | Reduced virulence | 18.875 | 102.9 | 126.995 | 117.49 | 164.62 |
| GL_Gan1_GLEAN_10000583 | Cercospora nicotianae | Reduced virulence | 0 | 0 | 0.18 | 0.215 | 0.29 |
| GL_Gan1_GLEAN_10000585 | Aspergillus fumigatus | Reduced virulence | 20.475 | 19.255 | 19.68 | 14.04 | 19.51 |
| GL_Gan1_GLEAN_10000586 | Magnaporthe grisea | Effector (plant avirulence determinant) | 15.025 | 12.235 | 12.34 | 7.725 | 8.475 |
| GL_Gan1_GLEAN_10000588 | Stagonospora nodorum | Unaffected pathogenicity | 257.135 | 643.875 | 234.08 | 235.82 | 563 |
| GL_Gan1_GLEAN_10000589 | Candida albicans | Reduced virulence | 0.795 | 0.87 | 0.62 | 0.735 | 0.435 |
| GL_Gan1_GLEAN_10000590 | Magnaporthe grisea | Reduced virulence | 24.885 | 53.93 | 54.525 | 52.635 | 42.46 |
| GL_Gan1_GLEAN_10000593 | Cercospora nicotianae | Reduced virulence | 0 | 1.615 | 1.14 | 1.275 | 1.02 |
| GL_Gan1_GLEAN_10000698 | Fusarium oxysporum | Loss of pathogenicity | 20.905 | 5.1 | 5.865 | 5.145 | 6.165 |
| GL_Gan1_GLEAN_10000699 | Candida albicans | Reduced virulence | 13.82 | 8.985 | 12.16 | 10.775 | 10.725 |
| GL_Gan1_GLEAN_10005676 | Blastomyces dermatitidis | Loss of pathogenicity | 0 | 0 | 1.54 | 1 | 0.45 |
| GL_Gan1_GLEAN_10000720 | Fusarium oxysporum | Loss of pathogenicity | 57.57 | 12.63 | 12.475 | 9.31 | 15.25 |
| GL_Gan1_GLEAN_10001328 | Cochliobolus carbonum | Reduced virulence | 221.635 | 201.64 | 284.62 | 227.515 | 302.845 |
| GL_Gan1_GLEAN_10001329 | Magnaporthe grisea | Reduced virulence | 18.085 | 14.915 | 35.775 | 27.245 | 20.26 |
| GL_Gan1_GLEAN_10001333 | Magnaporthe grisea | Loss of pathogenicity | 32.27 | 30.755 | 35.515 | 33.285 | 36.44 |
| GL_Gan1_GLEAN_10001341 | Magnaporthe grisea | Reduced virulence | 4.015 | 13.485 | 16.51 | 19.785 | 18.525 |
| GL_Gan1_GLEAN_10001344 | Colletotrichum lagenarium | Reduced virulence | 11.21 | 9.205 | 25.045 | 15.61 | 12.47 |
| GL_Gan1_GLEAN_10001357 | Botrytis cinerea | Unaffected pathogenicity | 3.99 | 22.365 | 35.68 | 30.505 | 55.39 |
| GL_Gan1_GLEAN_10002925 | Cladosporium fulvum | Reduced virulence | 0.615 | 0.54 | 0.275 | 0.405 | 1.215 |
| GL_Gan1_GLEAN_10008266 | Aspergillus fumigatus | Reduced virulence | 4.64 | 26.585 | 32.535 | 38.56 | 24.44 |
| GL_Gan1_GLEAN_10008270 | Botrytis cinerea | Unaffected pathogenicity | 4.105 | 1.815 | 4.91 | 4.735 | 4.53 |
| GL_Gan1_GLEAN_10008271 | Cochliobolus carbonum | Unaffected pathogenicity | 198.995 | 41.195 | 34.155 | 43.18 | 24.785 |
| GL_Gan1_GLEAN_10008291 | Magnaporthe grisea | Reduced virulence | 84.785 | 190.51 | 51.62 | 66.355 | 169.135 |
| GL_Gan1_GLEAN_10007439 | Claviceps purpurea | Loss of pathogenicity | 9.915 | 30.3 | 32.56 | 34.525 | 37.54 |
| GL_Gan1_GLEAN_10008313 | Ustilago maydis | Loss of pathogenicity | 7.08 | 33.375 | 7.26 | 7.455 | 26.94 |
| GL_Gan1_GLEAN_10008319 | Botrytis cinerea | Reduced virulence | 2.11 | 59.835 | 123.75 | 96.075 | 64.17 |
| GL_Gan1_GLEAN_10008327 | Fusarium oxysporum | Unaffected pathogenicity | 13.625 | 25.485 | 35.105 | 35.74 | 58.01 |
| GL_Gan1_GLEAN_10008334 | Candida albicans | Reduced virulence | 2.385 | 40.61 | 28.98 | 32.29 | 30.52 |
| GL_Gan1_GLEAN_10008341 | Fusarium graminearum | Reduced virulence | 2.225 | 10.55 | 15.75 | 15.19 | 23.645 |
| GL_Gan1_GLEAN_10008347 | Cryptococcus neoformans | Unaffected pathogenicity | 9.805 | 110.82 | 42.225 | 43.39 | 78.965 |
| GL_Gan1_GLEAN_10008356 | Cladosporium fulvum | Reduced virulence | 2.795 | 25.315 | 13.41 | 10.445 | 37.225 |
| GL_Gan1_GLEAN_10008358 | Magnaporthe grisea | Reduced virulence | 48.94 | 38.695 | 19.085 | 20.255 | 34.485 |
| GL_Gan1_GLEAN_10008368 | Cercospora nicotianae | Reduced virulence | 12 | 14.135 | 19.79 | 25.7 | 26.56 |
| GL_Gan1_GLEAN_10008369 | Colletotrichum lindemuthianum | Loss of pathogenicity | 25.305 | 8.08 | 10.155 | 9.975 | 9.66 |
| GL_Gan1_GLEAN_10000931 | Claviceps purpurea | Unaffected pathogenicity | 3.195 | 5.15 | 6.255 | 6.16 | 4.72 |
| GL_Gan1_GLEAN_10008372 | Cryptococcus neoformans | Reduced virulence | 140.26 | 98.505 | 164.765 | 187.275 | 142.525 |
| GL_Gan1_GLEAN_10008374 | Cochliobolus heterostrophus | Reduced virulence | 756.485 | 239.68 | 254.155 | 253.12 | 164.485 |
| GL_Gan1_GLEAN_10000802 | Claviceps purpurea | Reduced virulence | 0 | 0.62 | 0.38 | 0.5 | 0.505 |
| GL_Gan1_GLEAN_10008390 | Cryptococcus neoformans | Unaffected pathogenicity | 0 | 17.595 | 20.18 | 15.395 | 22.69 |
| GL_Gan1_GLEAN_10008402 | Fusarium oxysporum | Unaffected pathogenicity | 0.475 | 0.525 | 0.395 | 0.275 | 0.45 |
| GL_Gan1_GLEAN_10008404 | Saccharomyces cerevisiae | Reduced virulence | 42.56 | 97.875 | 142.665 | 134.94 | 106.675 |
| GL_Gan1_GLEAN_10008411 | Magnaporthe grisea | Reduced virulence | 1.145 | 25.025 | 24.415 | 26.99 | 31.545 |
| GL_Gan1_GLEAN_10008412 | Candida albicans | Reduced virulence | 68.345 | 44.195 | 44.565 | 46.095 | 60.58 |
| GL_Gan1_GLEAN_10008414 | Colletotrichum lindemuthianum | Reduced virulence | 69.06 | 45.465 | 61.205 | 71.17 | 34.655 |
| GL_Gan1_GLEAN_10000290 | Claviceps purpurea | Loss of pathogenicity | 58.175 | 86.895 | 80.565 | 93.35 | 99.095 |
| GL_Gan1_GLEAN_10008417 | Fusarium oxysporum | Unaffected pathogenicity | 48.24 | 12.54 | 19.205 | 14.495 | 15.13 |
| GL_Gan1_GLEAN_10008418 | Botrytis cinerea | Unaffected pathogenicity | 27.77 | 47.53 | 38.9 | 25.525 | 34.385 |
| GL_Gan1_GLEAN_10008422 | Colletotrichum gloeosporioides | Loss of pathogenicity | 72.085 | 53.42 | 24.02 | 29.435 | 50.625 |
| GL_Gan1_GLEAN_10001047 | Claviceps purpurea | Loss of pathogenicity | 8.89 | 73.475 | 85.765 | 88.225 | 109.8 |
| GL_Gan1_GLEAN_10008429 | Saccharomyces cerevisiae | Reduced virulence | 52.785 | 200.65 | 65.965 | 54.065 | 172.815 |
| GL_Gan1_GLEAN_10008430 | Cryptococcus neoformans | Reduced virulence | 42.9 | 45.06 | 81.91 | 73.475 | 32.81 |
| GL_Gan1_GLEAN_10008435 | Candida albicans | Unaffected pathogenicity | 4.285 | 15.03 | 10.045 | 9.055 | 16.275 |
| GL_Gan1_GLEAN_10005170 | Claviceps purpurea | Reduced virulence | 419.2 | 68.28 | 84.05 | 85.785 | 83.625 |
| GL_Gan1_GLEAN_10008443 | Colletotrichum lindemuthianum | Loss of pathogenicity | 3.05 | 4.455 | 2.71 | 2.08 | 4.105 |
| GL_Gan1_GLEAN_10008447 | Magnaporthe grisea | Reduced virulence | 19.59 | 32.415 | 22.915 | 28.325 | 34.585 |
| GL_Gan1_GLEAN_10008448 | Candida albicans | Reduced virulence | 2.595 | 2.86 | 1.765 | 1.89 | 2.575 |
| GL_Gan1_GLEAN_10008459 | Ustilago maydis | Reduced virulence | 8.085 | 46.11 | 29.175 | 26.53 | 63.895 |
| GL_Gan1_GLEAN_10008465 | Colletotrichum lindemuthianum | Loss of pathogenicity | 4.275 | 3.57 | 3.375 | 2.055 | 3.12 |
| GL_Gan1_GLEAN_10008468 | Cochliobolus heterostrophus | Loss of pathogenicity | 28.31 | 31.62 | 41.865 | 45.07 | 30.64 |
| GL_Gan1_GLEAN_10008469 | Aspergillus fumigatus | Reduced virulence | 46.265 | 19.535 | 30.93 | 32.87 | 24.665 |
| GL_Gan1_GLEAN_10008470 | Candida albicans | Reduced virulence | 53.235 | 36.34 | 64.9 | 60.88 | 36.215 |
| GL_Gan1_GLEAN_10003975 | Claviceps purpurea | Unaffected pathogenicity | 479.855 | 15.825 | 56.68 | 61.96 | 18.02 |
| GL_Gan1_GLEAN_10008493 | Colletotrichum lagenarium | Reduced virulence | 29.34 | 36.455 | 53.05 | 49.495 | 41.515 |
| GL_Gan1_GLEAN_10008497 | Cercospora nicotianae | Reduced virulence | 0 | 0.455 | 0.62 | 0.46 | 0.17 |
| GL_Gan1_GLEAN_10003294 | Claviceps purpurea | Reduced virulence | 94.53 | 70.995 | 85.695 | 82.57 | 70.855 |
| GL_Gan1_GLEAN_10008514 | Cochliobolus heterostrophus | Reduced virulence | 6.175 | 4.905 | 9.995 | 8.985 | 7.435 |
| GL_Gan1_GLEAN_10008515 | Aspergillus fumigatus | Reduced virulence | 16.14 | 58.595 | 123.625 | 149.375 | 53.895 |
| GL_Gan1_GLEAN_10000112 | Cochliobolus heterostrophus | Reduced virulence | 15.39 | 6.44 | 6.115 | 5.72 | 6.5 |
| GL_Gan1_GLEAN_10000113 | Cochliobolus heterostrophus | Reduced virulence | 0 | 8.4 | 7.095 | 11.655 | 13.22 |
| GL_Gan1_GLEAN_10000115 | Magnaporthe grisea | Effector (plant avirulence determinant) | 4.61 | 36.43 | 80.695 | 68.385 | 62.875 |
| GL_Gan1_GLEAN_10001148 | Fusarium oxysporum | Loss of pathogenicity | 37.24 | 10.14 | 12.845 | 13.925 | 11.025 |
| GL_Gan1_GLEAN_10001150 | Magnaporthe grisea | Reduced virulence | 0 | 1.395 | 1.495 | 1.64 | 0.715 |
| GL_Gan1_GLEAN_10001152 | Phytophthora infestans | Reduced virulence | 42.37 | 89.51 | 78.055 | 73.545 | 90.985 |
| GL_Gan1_GLEAN_10001155 | Cryptococcus neoformans | Reduced virulence | 81.155 | 46.865 | 51.7 | 52.49 | 48.61 |
| GL_Gan1_GLEAN_10001161 | Candida albicans | Reduced virulence | 17.825 | 18.27 | 20.415 | 18.545 | 16.7 |
| GL_Gan1_GLEAN_10001171 | Colletotrichum lindemuthianum | Reduced virulence | 30.805 | 47.585 | 49.265 | 43.365 | 44.295 |
| GL_Gan1_GLEAN_10001176 | Botrytis cinerea | Unaffected pathogenicity | 49.985 | 33.17 | 9.76 | 11.145 | 24.025 |
| GL_Gan1_GLEAN_10001180 | Trichoderma virens | Reduced virulence | 0.415 | 0.455 | 0.745 | 0.595 | 0.21 |
| GL_Gan1_GLEAN_10004437 | Botrytis cinerea | Unaffected pathogenicity | 0.55 | 0.36 | 0.25 | 0.345 | 0.46 |
| GL_Gan1_GLEAN_10004440 | Botrytis cinerea | Unaffected pathogenicity | 45.105 | 10.855 | 4.61 | 9.745 | 8.72 |
| GL_Gan1_GLEAN_10004443 | Candida albicans | Reduced virulence | 44.555 | 13.695 | 4.925 | 6.1 | 10.545 |
| GL_Gan1_GLEAN_10004446 | Colletotrichum acutatum | Loss of pathogenicity | 21.685 | 44.325 | 76.725 | 74.585 | 50.875 |
| GL_Gan1_GLEAN_10004453 | Botrytis cinerea | Reduced virulence | 34.8 | 25.895 | 28.34 | 25.475 | 29.485 |
| GL_Gan1_GLEAN_10004458 | Aspergillus fumigatus | Reduced virulence | 0 | 0.21 | 0.165 | 0.165 | 0 |
| GL_Gan1_GLEAN_10004459 | Candida albicans | Reduced virulence | 0.58 | 0.505 | 1.945 | 1.805 | 0.565 |
| GL_Gan1_GLEAN_10004465 | Botrytis cinerea | Unaffected pathogenicity | 1.49 | 6.53 | 10.615 | 9.515 | 4.215 |
| GL_Gan1_GLEAN_10004466 | Botrytis cinerea | Reduced virulence | 33.525 | 51.115 | 64.28 | 48.175 | 40.335 |
| GL_Gan1_GLEAN_10004467 | Candida albicans | Reduced virulence | 62.49 | 13.305 | 19.865 | 19.19 | 12.465 |
| GL_Gan1_GLEAN_10005272 | Claviceps purpurea | Reduced virulence | 552.71 | 90.635 | 129.57 | 146.195 | 64.18 |
| GL_Gan1_GLEAN_10004471 | Cercospora nicotianae | Reduced virulence | 3.03 | 1.555 | 1.82 | 1.1 | 0.59 |
| GL_Gan1_GLEAN_10004484 | Cochliobolus carbonum | Reduced virulence | 37.725 | 52.035 | 37.38 | 42.23 | 50.165 |
| GL_Gan1_GLEAN_10005805 | Claviceps purpurea | Reduced virulence | 13.35 | 22.555 | 33.805 | 36.605 | 32.515 |
| GL_Gan1_GLEAN_10004496 | Cercospora nicotianae | Reduced virulence | 22.045 | 17.255 | 21.08 | 18.755 | 16.77 |
| GL_Gan1_GLEAN_10004497 | Magnaporthe grisea | Reduced virulence | 178.025 | 13.655 | 14.08 | 13.495 | 4.555 |
| GL_Gan1_GLEAN_10004499 | Candida albicans | Reduced virulence | 1.135 | 3.375 | 2.715 | 2.21 | 4.42 |
| GL_Gan1_GLEAN_10004510 | Colletotrichum lagenarium | Loss of pathogenicity | 5.535 | 17.255 | 21.955 | 34.555 | 20.43 |
| GL_Gan1_GLEAN_10004514 | Candida albicans | Reduced virulence | 0 | 1.48 | 1.04 | 4.335 | 2.01 |
| GL_Gan1_GLEAN_10004515 | Fusarium graminearum | Reduced virulence | 11.825 | 21.85 | 12.83 | 16.125 | 25.225 |
| GL_Gan1_GLEAN_10004520 | Fusarium graminearum | Unaffected pathogenicity | 0 | 1.69 | 0.835 | 0.84 | 0.855 |
| GL_Gan1_GLEAN_10004524 | Botrytis cinerea | Reduced virulence | 1.95 | 14.25 | 2.9 | 7.8 | 19.805 |
| GL_Gan1_GLEAN_10004526 | Ustilago maydis | Reduced virulence | 8.145 | 14.34 | 16.11 | 13.54 | 14.06 |
| GL_Gan1_GLEAN_10004531 | Cochliobolus heterostrophus | Unaffected pathogenicity | 90.17 | 38.57 | 39.11 | 41.19 | 48.08 |
| GL_Gan1_GLEAN_10004535 | Magnaporthe grisea | Reduced virulence | 0 | 2.92 | 3.6 | 2.79 | 1.19 |
| GL_Gan1_GLEAN_10004536 | Candida albicans | Reduced virulence | 0 | 3.865 | 4.97 | 4.775 | 2.925 |
| GL_Gan1_GLEAN_10004538 | Candida albicans | Reduced virulence | 0 | 9.765 | 6.975 | 6.04 | 6.035 |
| GL_Gan1_GLEAN_10004543 | Botrytis cinerea | Unaffected pathogenicity | 0 | 4.405 | 2.755 | 2.655 | 1.495 |
| GL_Gan1_GLEAN_10000253 | Magnaporthe grisea | Reduced virulence | 41.74243707 | 10.61270252 | 20.50002975 | 22.54612586 | 9.314906178 |
| GL_Gan1_GLEAN_10003382 | Ustilago maydis | Reduced virulence | 41.57974895 | 10.08819439 | 20.18746879 | 22.28137765 | 8.73948627 |
| GL_Gan1_GLEAN_10003384 | Magnaporthe grisea | Reduced virulence | 41.41706084 | 9.563686262 | 19.87490784 | 22.01662945 | 8.164066362 |
| GL_Gan1_GLEAN_10003386 | Fusarium oxysporum | Reduced virulence | 41.25437272 | 9.039178135 | 19.56234688 | 21.75188124 | 7.588646453 |
| GL_Gan1_GLEAN_10003406 | Colletotrichum lagenarium | Loss of pathogenicity | 41.09168461 | 8.514670007 | 19.24978592 | 21.48713304 | 7.013226545 |
| GL_Gan1_GLEAN_10003407 | Cercospora nicotianae | Reduced virulence | 40.92899649 | 7.99016188 | 18.93722497 | 21.22238483 | 6.437806636 |
| GL_Gan1_GLEAN_10003430 | Magnaporthe grisea | Reduced virulence | 40.76630837 | 7.465653752 | 18.62466401 | 20.95763663 | 5.862386728 |
| GL_Gan1_GLEAN_10003432 | Claviceps purpurea | Reduced virulence | 40.60362026 | 6.941145625 | 18.31210305 | 20.69288842 | 5.286966819 |
| GL_Gan1_GLEAN_10003433 | Magnaporthe grisea | Reduced virulence | 40.44093214 | 6.416637497 | 17.9995421 | 20.42814022 | 4.711546911 |
| GL_Gan1_GLEAN_10003442 | Candida albicans | Reduced virulence | 40.27824402 | 5.89212937 | 17.68698114 | 20.16339201 | 4.136127002 |
| GL_Gan1_GLEAN_10000583 | Cercospora nicotianae | Reduced virulence | 40.11555591 | 5.367621242 | 17.37442018 | 19.89864381 | 3.560707094 |
| GL_Gan1_GLEAN_10000585 | Aspergillus fumigatus | Reduced virulence | 39.95286779 | 4.843113114 | 17.06185923 | 19.6338956 | 2.985287185 |
| GL_Gan1_GLEAN_10000586 | Magnaporthe grisea | Effector (plant avirulence determinant) | 39.79017967 | 4.318604987 | 16.74929827 | 19.3691474 | 2.409867277 |
| GL_Gan1_GLEAN_10000588 | Stagonospora nodorum | Unaffected pathogenicity | 39.62749156 | 3.794096859 | 16.43673732 | 19.1043992 | 1.834447368 |
| GL_Gan1_GLEAN_10000589 | Candida albicans | Reduced virulence | 39.46480344 | 3.269588732 | 16.12417636 | 18.83965099 | 1.25902746 |
| GL_Gan1_GLEAN_10000590 | Magnaporthe grisea | Reduced virulence | 39.30211532 | 2.745080604 | 15.8116154 | 18.57490279 | 0.683607551 |
| GL_Gan1_GLEAN_10000593 | Cercospora nicotianae | Reduced virulence | 39.13942721 | 2.220572477 | 15.49905445 | 18.31015458 | 0.108187643 |
| GL_Gan1_GLEAN_10000698 | Fusarium oxysporum | Loss of pathogenicity | 38.97673909 | 1.696064349 | 15.18649349 | 18.04540638 | -0.46723227 |
| GL_Gan1_GLEAN_10000699 | Candida albicans | Reduced virulence | 38.81405097 | 1.171556222 | 14.87393253 | 17.78065817 | -1.04265217 |
| GL_Gan1_GLEAN_10005676 | Blastomyces dermatitidis | Loss of pathogenicity | 38.65136286 | 0.647048094 | 14.56137158 | 17.51590997 | -1.61807208 |
| GL_Gan1_GLEAN_10000720 | Fusarium oxysporum | Loss of pathogenicity | 38.48867474 | 0.122539967 | 14.24881062 | 17.25116176 | -2.19349199 |
| GL_Gan1_GLEAN_10001328 | Cochliobolus carbonum | Reduced virulence | 38.32598663 | -0.40196816 | 13.93624966 | 16.98641356 | -2.7689119 |
| GL_Gan1_GLEAN_10001329 | Magnaporthe grisea | Reduced virulence | 38.16329851 | -0.92647629 | 13.62368871 | 16.72166535 | -3.34433181 |
| GL_Gan1_GLEAN_10001333 | Magnaporthe grisea | Loss of pathogenicity | 38.00061039 | -1.45098442 | 13.31112775 | 16.45691715 | -3.91975172 |
| GL_Gan1_GLEAN_10001341 | Magnaporthe grisea | Reduced virulence | 37.83792228 | -1.97549254 | 12.9985668 | 16.19216894 | -4.49517162 |
| GL_Gan1_GLEAN_10001344 | Colletotrichum lagenarium | Reduced virulence | 37.67523416 | -2.50000067 | 12.68600584 | 15.92742074 | -5.07059153 |
| GL_Gan1_GLEAN_10001357 | Botrytis cinerea | Unaffected pathogenicity | 37.51254604 | -3.0245088 | 12.37344488 | 15.66267253 | -5.64601144 |
| GL_Gan1_GLEAN_10002925 | Cladosporium fulvum | Reduced virulence | 37.34985793 | -3.54901693 | 12.06088393 | 15.39792433 | -6.22143135 |
| GL_Gan1_GLEAN_10008266 | Aspergillus fumigatus | Reduced virulence | 37.18716981 | -4.07352505 | 11.74832297 | 15.13317612 | -6.79685126 |
| GL_Gan1_GLEAN_10008270 | Botrytis cinerea | Unaffected pathogenicity | 37.02448169 | -4.59803318 | 11.43576201 | 14.86842792 | -7.37227117 |
| GL_Gan1_GLEAN_10008271 | Cochliobolus carbonum | Unaffected pathogenicity | 36.86179358 | -5.12254131 | 11.12320106 | 14.60367971 | -7.94769108 |
| GL_Gan1_GLEAN_10008291 | Magnaporthe grisea | Reduced virulence | 36.69910546 | -5.64704944 | 10.8106401 | 14.33893151 | -8.52311098 |
| GL_Gan1_GLEAN_10007439 | Claviceps purpurea | Loss of pathogenicity | 36.53641734 | -6.17155756 | 10.49807914 | 14.0741833 | -9.09853089 |
| GL_Gan1_GLEAN_10008313 | Ustilago maydis | Loss of pathogenicity | 36.37372923 | -6.69606569 | 10.18551819 | 13.8094351 | -9.6739508 |
| GL_Gan1_GLEAN_10008319 | Botrytis cinerea | Reduced virulence | 36.21104111 | -7.22057382 | 9.872957232 | 13.54468689 | -10.2493707 |
| GL_Gan1_GLEAN_10008327 | Fusarium oxysporum | Unaffected pathogenicity | 36.04835299 | -7.74508195 | 9.560396276 | 13.27993869 | -10.8247906 |
| GL_Gan1_GLEAN_10008334 | Candida albicans | Reduced virulence | 35.88566488 | -8.26959007 | 9.247835319 | 13.01519048 | -11.4002105 |
| GL_Gan1_GLEAN_10008341 | Fusarium graminearum | Reduced virulence | 35.72297676 | -8.7940982 | 8.935274363 | 12.75044228 | -11.9756304 |
| GL_Gan1_GLEAN_10008347 | Cryptococcus neoformans | Unaffected pathogenicity | 35.56028865 | -9.31860633 | 8.622713406 | 12.48569407 | -12.5510503 |
| GL_Gan1_GLEAN_10008356 | Cladosporium fulvum | Reduced virulence | 35.39760053 | -9.84311446 | 8.31015245 | 12.22094587 | -13.1264703 |
| GL_Gan1_GLEAN_10008358 | Magnaporthe grisea | Reduced virulence | 35.23491241 | -10.3676226 | 7.997591494 | 11.95619766 | -13.7018902 |
| GL_Gan1_GLEAN_10008368 | Cercospora nicotianae | Reduced virulence | 35.0722243 | -10.8921307 | 7.685030537 | 11.69144946 | -14.2773101 |
| GL_Gan1_GLEAN_10008369 | Colletotrichum lindemuthianum | Loss of pathogenicity | 34.90953618 | -11.4166388 | 7.372469581 | 11.42670125 | -14.85273 |
| GL_Gan1_GLEAN_10000931 | Claviceps purpurea | Unaffected pathogenicity | 34.74684806 | -11.941147 | 7.059908625 | 11.16195305 | -15.4281499 |
| GL_Gan1_GLEAN_10008372 | Cryptococcus neoformans | Reduced virulence | 34.58415995 | -12.4656551 | 6.747347668 | 10.89720484 | -16.0035698 |
| GL_Gan1_GLEAN_10008374 | Cochliobolus heterostrophus | Reduced virulence | 34.42147183 | -12.9901632 | 6.434786712 | 10.63245664 | -16.5789897 |
| GL_Gan1_GLEAN_10000802 | Claviceps purpurea | Reduced virulence | 34.25878371 | -13.5146713 | 6.122225756 | 10.36770844 | -17.1544096 |
| GL_Gan1_GLEAN_10008390 | Cryptococcus neoformans | Unaffected pathogenicity | 34.0960956 | -14.0391795 | 5.809664799 | 10.10296023 | -17.7298295 |
| GL_Gan1_GLEAN_10008402 | Fusarium oxysporum | Unaffected pathogenicity | 33.93340748 | -14.5636876 | 5.497103843 | 9.838212026 | -18.3052494 |
| GL_Gan1_GLEAN_10008404 | Saccharomyces cerevisiae | Reduced virulence | 33.77071936 | -15.0881957 | 5.184542886 | 9.573463821 | -18.8806693 |
| GL_Gan1_GLEAN_10008411 | Magnaporthe grisea | Reduced virulence | 33.60803125 | -15.6127039 | 4.87198193 | 9.308715616 | -19.4560892 |
| GL_Gan1_GLEAN_10008412 | Candida albicans | Reduced virulence | 33.44534313 | -16.137212 | 4.559420974 | 9.043967411 | -20.0315092 |
| GL_Gan1_GLEAN_10008414 | Colletotrichum lindemuthianum | Reduced virulence | 33.28265501 | -16.6617201 | 4.246860017 | 8.779219206 | -20.6069291 |
| GL_Gan1_GLEAN_10000290 | Claviceps purpurea | Loss of pathogenicity | 33.1199669 | -17.1862282 | 3.934299061 | 8.514471001 | -21.182349 |
| GL_Gan1_GLEAN_10008417 | Fusarium oxysporum | Unaffected pathogenicity | 32.95727878 | -17.7107364 | 3.621738105 | 8.249722796 | -21.7577689 |
| GL_Gan1_GLEAN_10008418 | Botrytis cinerea | Unaffected pathogenicity | 32.79459067 | -18.2352445 | 3.309177148 | 7.984974592 | -22.3331888 |
| GL_Gan1_GLEAN_10008422 | Colletotrichum gloeosporioides | Loss of pathogenicity | 32.63190255 | -18.7597526 | 2.996616192 | 7.720226387 | -22.9086087 |
| GL_Gan1_GLEAN_10001047 | Claviceps purpurea | Loss of pathogenicity | 32.46921443 | -19.2842608 | 2.684055236 | 7.455478182 | -23.4840286 |
| GL_Gan1_GLEAN_10008429 | Saccharomyces cerevisiae | Reduced virulence | 32.30652632 | -19.8087689 | 2.371494279 | 7.190729977 | -24.0594485 |
| GL_Gan1_GLEAN_10008430 | Cryptococcus neoformans | Reduced virulence | 32.1438382 | -20.333277 | 2.058933323 | 6.925981772 | -24.6348684 |
| GL_Gan1_GLEAN_10008435 | Candida albicans | Unaffected pathogenicity | 31.98115008 | -20.8577851 | 1.746372366 | 6.661233567 | -25.2102883 |
| GL_Gan1_GLEAN_10005170 | Claviceps purpurea | Reduced virulence | 31.81846197 | -21.3822933 | 1.43381141 | 6.396485363 | -25.7857082 |
| GL_Gan1_GLEAN_10008443 | Colletotrichum lindemuthianum | Loss of pathogenicity | 31.65577385 | -21.9068014 | 1.121250454 | 6.131737158 | -26.3611281 |
| GL_Gan1_GLEAN_10008447 | Magnaporthe grisea | Reduced virulence | 31.49308573 | -22.4313095 | 0.808689497 | 5.866988953 | -26.9365481 |
| GL_Gan1_GLEAN_10008448 | Candida albicans | Reduced virulence | 31.33039762 | -22.9558176 | 0.496128541 | 5.602240748 | -27.511968 |
| GL_Gan1_GLEAN_10008459 | Ustilago maydis | Reduced virulence | 31.1677095 | -23.4803258 | 0.183567585 | 5.337492543 | -28.0873879 |
| GL_Gan1_GLEAN_10008465 | Colletotrichum lindemuthianum | Loss of pathogenicity | 31.00502138 | -24.0048339 | -0.12899337 | 5.072744338 | -28.6628078 |
| GL_Gan1_GLEAN_10008468 | Cochliobolus heterostrophus | Loss of pathogenicity | 30.84233327 | -24.529342 | -0.44155433 | 4.807996134 | -29.2382277 |
| GL_Gan1_GLEAN_10008469 | Aspergillus fumigatus | Reduced virulence | 30.67964515 | -25.0538502 | -0.75411528 | 4.543247929 | -29.8136476 |
| GL_Gan1_GLEAN_10008470 | Candida albicans | Reduced virulence | 30.51695703 | -25.5783583 | -1.06667624 | 4.278499724 | -30.3890675 |
| GL_Gan1_GLEAN_10003975 | Claviceps purpurea | Unaffected pathogenicity | 30.35426892 | -26.1028664 | -1.3792372 | 4.013751519 | -30.9644874 |
| GL_Gan1_GLEAN_10008493 | Colletotrichum lagenarium | Reduced virulence | 30.1915808 | -26.6273745 | -1.69179815 | 3.749003314 | -31.5399073 |
| GL_Gan1_GLEAN_10008497 | Cercospora nicotianae | Reduced virulence | 30.02889269 | -27.1518827 | -2.00435911 | 3.484255109 | -32.1153272 |
| GL_Gan1_GLEAN_10003294 | Claviceps purpurea | Reduced virulence | 29.86620457 | -27.6763908 | -2.31692007 | 3.219506904 | -32.6907471 |
| GL_Gan1_GLEAN_10008514 | Cochliobolus heterostrophus | Reduced virulence | 29.70351645 | -28.2008989 | -2.62948102 | 2.9547587 | -33.266167 |
| GL_Gan1_GLEAN_10008515 | Aspergillus fumigatus | Reduced virulence | 29.54082834 | -28.725407 | -2.94204198 | 2.690010495 | -33.841587 |
| GL_Gan1_GLEAN_10000112 | Cochliobolus heterostrophus | Reduced virulence | 29.37814022 | -29.2499152 | -3.25460294 | 2.42526229 | -34.4170069 |
| GL_Gan1_GLEAN_10000113 | Cochliobolus heterostrophus | Reduced virulence | 29.2154521 | -29.7744233 | -3.56716389 | 2.160514085 | -34.9924268 |
| GL_Gan1_GLEAN_10000115 | Magnaporthe grisea | Effector (plant avirulence determinant) | 29.05276399 | -30.2989314 | -3.87972485 | 1.89576588 | -35.5678467 |
| GL_Gan1_GLEAN_10001148 | Fusarium oxysporum | Loss of pathogenicity | 28.89007587 | -30.8234396 | -4.1922858 | 1.631017675 | -36.1432666 |
| GL_Gan1_GLEAN_10001150 | Magnaporthe grisea | Reduced virulence | 28.72738775 | -31.3479477 | -4.50484676 | 1.366269471 | -36.7186865 |
| GL_Gan1_GLEAN_10001152 | Phytophthora infestans | Reduced virulence | 28.56469964 | -31.8724558 | -4.81740772 | 1.101521266 | -37.2941064 |
| GL_Gan1_GLEAN_10001155 | Cryptococcus neoformans | Reduced virulence | 28.40201152 | -32.3969639 | -5.12996867 | 0.836773061 | -37.8695263 |
| GL_Gan1_GLEAN_10001161 | Candida albicans | Reduced virulence | 28.2393234 | -32.9214721 | -5.44252963 | 0.572024856 | -38.4449462 |
| GL_Gan1_GLEAN_10001171 | Colletotrichum lindemuthianum | Reduced virulence | 28.07663529 | -33.4459802 | -5.75509059 | 0.307276651 | -39.0203661 |
| GL_Gan1_GLEAN_10001176 | Botrytis cinerea | Unaffected pathogenicity | 27.91394717 | -33.9704883 | -6.06765154 | 0.042528446 | -39.595786 |
| GL_Gan1_GLEAN_10001180 | Trichoderma virens | Reduced virulence | 27.75125905 | -34.4949964 | -6.3802125 | -0.22221976 | -40.1712059 |
| GL_Gan1_GLEAN_10004437 | Botrytis cinerea | Unaffected pathogenicity | 27.58857094 | -35.0195046 | -6.69277346 | -0.48696796 | -40.7466259 |
| GL_Gan1_GLEAN_10004440 | Botrytis cinerea | Unaffected pathogenicity | 27.42588282 | -35.5440127 | -7.00533441 | -0.75171617 | -41.3220458 |
| GL_Gan1_GLEAN_10004443 | Candida albicans | Reduced virulence | 27.26319471 | -36.0685208 | -7.31789537 | -1.01646437 | -41.8974657 |
| GL_Gan1_GLEAN_10004446 | Colletotrichum acutatum | Loss of pathogenicity | 27.10050659 | -36.593029 | -7.63045632 | -1.28121258 | -42.4728856 |
| GL_Gan1_GLEAN_10004453 | Botrytis cinerea | Reduced virulence | 26.93781847 | -37.1175371 | -7.94301728 | -1.54596078 | -43.0483055 |
| GL_Gan1_GLEAN_10004458 | Aspergillus fumigatus | Reduced virulence | 26.77513036 | -37.6420452 | -8.25557824 | -1.81070899 | -43.6237254 |
| GL_Gan1_GLEAN_10004459 | Candida albicans | Reduced virulence | 26.61244224 | -38.1665533 | -8.56813919 | -2.07545719 | -44.1991453 |
| GL_Gan1_GLEAN_10004465 | Botrytis cinerea | Unaffected pathogenicity | 26.44975412 | -38.6910615 | -8.88070015 | -2.3402054 | -44.7745652 |
| GL_Gan1_GLEAN_10004466 | Botrytis cinerea | Reduced virulence | 26.28706601 | -39.2155696 | -9.19326111 | -2.6049536 | -45.3499851 |
| GL_Gan1_GLEAN_10004467 | Candida albicans | Reduced virulence | 26.12437789 | -39.7400777 | -9.50582206 | -2.86970181 | -45.925405 |
| GL_Gan1_GLEAN_10005272 | Claviceps purpurea | Reduced virulence | 25.96168977 | -40.2645859 | -9.81838302 | -3.13445001 | -46.5008249 |
| GL_Gan1_GLEAN_10004471 | Cercospora nicotianae | Reduced virulence | 25.79900166 | -40.789094 | -10.130944 | -3.39919822 | -47.0762449 |
| GL_Gan1_GLEAN_10004484 | Cochliobolus carbonum | Reduced virulence | 25.63631354 | -41.3136021 | -10.4435049 | -3.66394642 | -47.6516648 |
| GL_Gan1_GLEAN_10005805 | Claviceps purpurea | Reduced virulence | 25.47362542 | -41.8381102 | -10.7560659 | -3.92869463 | -48.2270847 |
| GL_Gan1_GLEAN_10004496 | Cercospora nicotianae | Reduced virulence | 25.31093731 | -42.3626184 | -11.0686268 | -4.19344283 | -48.8025046 |
| GL_Gan1_GLEAN_10004497 | Magnaporthe grisea | Reduced virulence | 25.14824919 | -42.8871265 | -11.3811878 | -4.45819104 | -49.3779245 |
| GL_Gan1_GLEAN_10004499 | Candida albicans | Reduced virulence | 24.98556107 | -43.4116346 | -11.6937488 | -4.72293924 | -49.9533444 |
| GL_Gan1_GLEAN_10004510 | Colletotrichum lagenarium | Loss of pathogenicity | 24.82287296 | -43.9361427 | -12.0063097 | -4.98768745 | -50.5287643 |
| GL_Gan1_GLEAN_10004514 | Candida albicans | Reduced virulence | 24.66018484 | -44.4606509 | -12.3188707 | -5.25243565 | -51.1041842 |
| GL_Gan1_GLEAN_10004515 | Fusarium graminearum | Reduced virulence | 24.49749673 | -44.985159 | -12.6314316 | -5.51718386 | -51.6796041 |
| GL_Gan1_GLEAN_10004520 | Fusarium graminearum | Unaffected pathogenicity | 24.33480861 | -45.5096671 | -12.9439926 | -5.78193206 | -52.255024 |
| GL_Gan1_GLEAN_10004524 | Botrytis cinerea | Reduced virulence | 24.17212049 | -46.0341753 | -13.2565535 | -6.04668027 | -52.8304439 |
| GL_Gan1_GLEAN_10004526 | Ustilago maydis | Reduced virulence | 24.00943238 | -46.5586834 | -13.5691145 | -6.31142847 | -53.4058638 |
| GL_Gan1_GLEAN_10004531 | Cochliobolus heterostrophus | Unaffected pathogenicity | 23.84674426 | -47.0831915 | -13.8816755 | -6.57617667 | -53.9812838 |
| GL_Gan1_GLEAN_10004535 | Magnaporthe grisea | Reduced virulence | 23.68405614 | -47.6076996 | -14.1942364 | -6.84092488 | -54.5567037 |
| GL_Gan1_GLEAN_10004536 | Candida albicans | Reduced virulence | 23.52136803 | -48.1322078 | -14.5067974 | -7.10567308 | -55.1321236 |
| GL_Gan1_GLEAN_10004538 | Candida albicans | Reduced virulence | 23.35867991 | -48.6567159 | -14.8193583 | -7.37042129 | -55.7075435 |
| GL_Gan1_GLEAN_10004543 | Botrytis cinerea | Unaffected pathogenicity | 23.19599179 | -49.181224 | -15.1319193 | -7.63516949 | -56.2829634 |
| GL_Gan1_GLEAN_10000253 | Magnaporthe grisea | Reduced virulence | 23.03330368 | -49.7057321 | -15.4444802 | -7.8999177 | -56.8583833 |
| GL_Gan1_GLEAN_10003382 | Ustilago maydis | Reduced virulence | 22.87061556 | -50.2302403 | -15.7570412 | -8.1646659 | -57.4338032 |
| GL_Gan1_GLEAN_10003384 | Magnaporthe grisea | Reduced virulence | 22.70792744 | -50.7547484 | -16.0696021 | -8.42941411 | -58.0092231 |
| GL_Gan1_GLEAN_10003386 | Fusarium oxysporum | Reduced virulence | 22.54523933 | -51.2792565 | -16.3821631 | -8.69416231 | -58.584643 |
| GL_Gan1_GLEAN_10003406 | Colletotrichum lagenarium | Loss of pathogenicity | 22.38255121 | -51.8037647 | -16.6947241 | -8.95891052 | -59.1600629 |
| GL_Gan1_GLEAN_10003407 | Cercospora nicotianae | Reduced virulence | 22.21986309 | -52.3282728 | -17.007285 | -9.22365872 | -59.7354828 |
| GL_Gan1_GLEAN_10003430 | Magnaporthe grisea | Reduced virulence | 22.05717498 | -52.8527809 | -17.319846 | -9.48840693 | -60.3109027 |
| GL_Gan1_GLEAN_10003432 | Claviceps purpurea | Reduced virulence | 21.89448686 | -53.377289 | -17.6324069 | -9.75315513 | -60.8863227 |
| GL_Gan1_GLEAN_10003433 | Magnaporthe grisea | Reduced virulence | 21.73179875 | -53.9017972 | -17.9449679 | -10.0179033 | -61.4617426 |
| GL_Gan1_GLEAN_10003442 | Candida albicans | Reduced virulence | 21.56911063 | -54.4263053 | -18.2575288 | -10.2826515 | -62.0371625 |
| GL_Gan1_GLEAN_10000583 | Cercospora nicotianae | Reduced virulence | 21.40642251 | -54.9508134 | -18.5700898 | -10.5473997 | -62.6125824 |
| GL_Gan1_GLEAN_10000585 | Aspergillus fumigatus | Reduced virulence | 21.2437344 | -55.4753215 | -18.8826508 | -10.812148 | -63.1880023 |
| GL_Gan1_GLEAN_10000586 | Magnaporthe grisea | Effector (plant avirulence determinant) | 21.08104628 | -55.9998297 | -19.1952117 | -11.0768962 | -63.7634222 |
| GL_Gan1_GLEAN_10000588 | Stagonospora nodorum | Unaffected pathogenicity | 20.91835816 | -56.5243378 | -19.5077727 | -11.3416444 | -64.3388421 |
| GL_Gan1_GLEAN_10000589 | Candida albicans | Reduced virulence | 20.75567005 | -57.0488459 | -19.8203336 | -11.6063926 | -64.914262 |
| GL_Gan1_GLEAN_10000590 | Magnaporthe grisea | Reduced virulence | 20.59298193 | -57.5733541 | -20.1328946 | -11.8711408 | -65.4896819 |
| GL_Gan1_GLEAN_10000593 | Cercospora nicotianae | Reduced virulence | 20.43029381 | -58.0978622 | -20.4454555 | -12.135889 | -66.0651018 |
| GL_Gan1_GLEAN_10000698 | Fusarium oxysporum | Loss of pathogenicity | 20.2676057 | -58.6223703 | -20.7580165 | -12.4006372 | -66.6405217 |
| GL_Gan1_GLEAN_10000699 | Candida albicans | Reduced virulence | 20.10491758 | -59.1468784 | -21.0705774 | -12.6653854 | -67.2159416 |
| GL_Gan1_GLEAN_10005676 | Blastomyces dermatitidis | Loss of pathogenicity | 19.94222946 | -59.6713866 | -21.3831384 | -12.9301336 | -67.7913616 |
| GL_Gan1_GLEAN_10000720 | Fusarium oxysporum | Loss of pathogenicity | 19.77954135 | -60.1958947 | -21.6956994 | -13.1948818 | -68.3667815 |
| GL_Gan1_GLEAN_10001328 | Cochliobolus carbonum | Reduced virulence | 19.61685323 | -60.7204028 | -22.0082603 | -13.45963 | -68.9422014 |
| GL_Gan1_GLEAN_10001329 | Magnaporthe grisea | Reduced virulence | 19.45416511 | -61.244911 | -22.3208213 | -13.7243782 | -69.5176213 |
| GL_Gan1_GLEAN_10001333 | Magnaporthe grisea | Loss of pathogenicity | 19.291477 | -61.7694191 | -22.6333822 | -13.9891264 | -70.0930412 |
| GL_Gan1_GLEAN_10001341 | Magnaporthe grisea | Reduced virulence | 19.12878888 | -62.2939272 | -22.9459432 | -14.2538746 | -70.6684611 |
| GL_Gan1_GLEAN_10001344 | Colletotrichum lagenarium | Reduced virulence | 18.96610077 | -62.8184353 | -23.2585041 | -14.5186228 | -71.243881 |
| GL_Gan1_GLEAN_10001357 | Botrytis cinerea | Unaffected pathogenicity | 18.80341265 | -63.3429435 | -23.5710651 | -14.783371 | -71.8193009 |
| GL_Gan1_GLEAN_10002925 | Cladosporium fulvum | Reduced virulence | 18.64072453 | -63.8674516 | -23.8836261 | -15.0481192 | -72.3947208 |
| GL_Gan1_GLEAN_10008266 | Aspergillus fumigatus | Reduced virulence | 18.47803642 | -64.3919597 | -24.196187 | -15.3128674 | -72.9701407 |
| GL_Gan1_GLEAN_10008270 | Botrytis cinerea | Unaffected pathogenicity | 18.3153483 | -64.9164678 | -24.508748 | -15.5776156 | -73.5455606 |
| GL_Gan1_GLEAN_10008271 | Cochliobolus carbonum | Unaffected pathogenicity | 18.15266018 | -65.440976 | -24.8213089 | -15.8423638 | -74.1209805 |
| GL_Gan1_GLEAN_10008291 | Magnaporthe grisea | Reduced virulence | 17.98997207 | -65.9654841 | -25.1338699 | -16.107112 | -74.6964005 |
| GL_Gan1_GLEAN_10007439 | Claviceps purpurea | Loss of pathogenicity | 17.82728395 | -66.4899922 | -25.4464308 | -16.3718603 | -75.2718204 |
| GL_Gan1_GLEAN_10008313 | Ustilago maydis | Loss of pathogenicity | 17.66459583 | -67.0145004 | -25.7589918 | -16.6366085 | -75.8472403 |
| GL_Gan1_GLEAN_10008319 | Botrytis cinerea | Reduced virulence | 17.50190772 | -67.5390085 | -26.0715527 | -16.9013567 | -76.4226602 |
| GL_Gan1_GLEAN_10008327 | Fusarium oxysporum | Unaffected pathogenicity | 17.3392196 | -68.0635166 | -26.3841137 | -17.1661049 | -76.9980801 |
| GL_Gan1_GLEAN_10008334 | Candida albicans | Reduced virulence | 17.17653148 | -68.5880247 | -26.6966747 | -17.4308531 | -77.5735 |
| GL_Gan1_GLEAN_10008341 | Fusarium graminearum | Reduced virulence | 17.01384337 | -69.1125329 | -27.0092356 | -17.6956013 | -78.1489199 |
| GL_Gan1_GLEAN_10008347 | Cryptococcus neoformans | Unaffected pathogenicity | 16.85115525 | -69.637041 | -27.3217966 | -17.9603495 | -78.7243398 |
| GL_Gan1_GLEAN_10008356 | Cladosporium fulvum | Reduced virulence | 16.68846713 | -70.1615491 | -27.6343575 | -18.2250977 | -79.2997597 |
| GL_Gan1_GLEAN_10008358 | Magnaporthe grisea | Reduced virulence | 16.52577902 | -70.6860572 | -27.9469185 | -18.4898459 | -79.8751796 |
| GL_Gan1_GLEAN_10008368 | Cercospora nicotianae | Reduced virulence | 16.3630909 | -71.2105654 | -28.2594794 | -18.7545941 | -80.4505995 |
| GL_Gan1_GLEAN_10008369 | Colletotrichum lindemuthianum | Loss of pathogenicity | 16.20040279 | -71.7350735 | -28.5720404 | -19.0193423 | -81.0260195 |
| GL_Gan1_GLEAN_10000931 | Claviceps purpurea | Unaffected pathogenicity | 16.03771467 | -72.2595816 | -28.8846014 | -19.2840905 | -81.6014394 |
| GL_Gan1_GLEAN_10008372 | Cryptococcus neoformans | Reduced virulence | 15.87502655 | -72.7840898 | -29.1971623 | -19.5488387 | -82.1768593 |
| GL_Gan1_GLEAN_10008374 | Cochliobolus heterostrophus | Reduced virulence | 15.71233844 | -73.3085979 | -29.5097233 | -19.8135869 | -82.7522792 |
| GL_Gan1_GLEAN_10000802 | Claviceps purpurea | Reduced virulence | 15.54965032 | -73.833106 | -29.8222842 | -20.0783351 | -83.3276991 |
| GL_Gan1_GLEAN_10008390 | Cryptococcus neoformans | Unaffected pathogenicity | 15.3869622 | -74.3576141 | -30.1348452 | -20.3430833 | -83.903119 |
| GL_Gan1_GLEAN_10008402 | Fusarium oxysporum | Unaffected pathogenicity | 15.22427409 | -74.8821223 | -30.4474061 | -20.6078315 | -84.4785389 |
| GL_Gan1_GLEAN_10008404 | Saccharomyces cerevisiae | Reduced virulence | 15.06158597 | -75.4066304 | -30.7599671 | -20.8725797 | -85.0539588 |
| GL_Gan1_GLEAN_10008411 | Magnaporthe grisea | Reduced virulence | 14.89889785 | -75.9311385 | -31.0725281 | -21.1373279 | -85.6293787 |
| GL_Gan1_GLEAN_10008412 | Candida albicans | Reduced virulence | 14.73620974 | -76.4556467 | -31.385089 | -21.4020761 | -86.2047986 |
| GL_Gan1_GLEAN_10008414 | Colletotrichum lindemuthianum | Reduced virulence | 14.57352162 | -76.9801548 | -31.69765 | -21.6668244 | -86.7802185 |
| GL_Gan1_GLEAN_10000290 | Claviceps purpurea | Loss of pathogenicity | 14.4108335 | -77.5046629 | -32.0102109 | -21.9315726 | -87.3556384 |
| GL_Gan1_GLEAN_10008417 | Fusarium oxysporum | Unaffected pathogenicity | 14.24814539 | -78.029171 | -32.3227719 | -22.1963208 | -87.9310584 |
| GL_Gan1_GLEAN_10008418 | Botrytis cinerea | Unaffected pathogenicity | 14.08545727 | -78.5536792 | -32.6353328 | -22.461069 | -88.5064783 |
| GL_Gan1_GLEAN_10008422 | Colletotrichum gloeosporioides | Loss of pathogenicity | 13.92276915 | -79.0781873 | -32.9478938 | -22.7258172 | -89.0818982 |
| GL_Gan1_GLEAN_10001047 | Claviceps purpurea | Loss of pathogenicity | 13.76008104 | -79.6026954 | -33.2604547 | -22.9905654 | -89.6573181 |
| GL_Gan1_GLEAN_10008429 | Saccharomyces cerevisiae | Reduced virulence | 13.59739292 | -80.1272035 | -33.5730157 | -23.2553136 | -90.232738 |
| GL_Gan1_GLEAN_10008430 | Cryptococcus neoformans | Reduced virulence | 13.43470481 | -80.6517117 | -33.8855767 | -23.5200618 | -90.8081579 |
| GL_Gan1_GLEAN_10008435 | Candida albicans | Unaffected pathogenicity | 13.27201669 | -81.1762198 | -34.1981376 | -23.78481 | -91.3835778 |
| GL_Gan1_GLEAN_10005170 | Claviceps purpurea | Reduced virulence | 13.10932857 | -81.7007279 | -34.5106986 | -24.0495582 | -91.9589977 |
| GL_Gan1_GLEAN_10008443 | Colletotrichum lindemuthianum | Loss of pathogenicity | 12.94664046 | -82.2252361 | -34.8232595 | -24.3143064 | -92.5344176 |
| GL_Gan1_GLEAN_10008447 | Magnaporthe grisea | Reduced virulence | 12.78395234 | -82.7497442 | -35.1358205 | -24.5790546 | -93.1098375 |
| GL_Gan1_GLEAN_10008448 | Candida albicans | Reduced virulence | 12.62126422 | -83.2742523 | -35.4483814 | -24.8438028 | -93.6852574 |
| GL_Gan1_GLEAN_10008459 | Ustilago maydis | Reduced virulence | 12.45857611 | -83.7987604 | -35.7609424 | -25.108551 | -94.2606773 |
| GL_Gan1_GLEAN_10008465 | Colletotrichum lindemuthianum | Loss of pathogenicity | 12.29588799 | -84.3232686 | -36.0735034 | -25.3732992 | -94.8360973 |
| GL_Gan1_GLEAN_10008468 | Cochliobolus heterostrophus | Loss of pathogenicity | 12.13319987 | -84.8477767 | -36.3860643 | -25.6380474 | -95.4115172 |
| GL_Gan1_GLEAN_10008469 | Aspergillus fumigatus | Reduced virulence | 11.97051176 | -85.3722848 | -36.6986253 | -25.9027956 | -95.9869371 |
| GL_Gan1_GLEAN_10008470 | Candida albicans | Reduced virulence | 11.80782364 | -85.8967929 | -37.0111862 | -26.1675438 | -96.562357 |
| GL_Gan1_GLEAN_10003975 | Claviceps purpurea | Unaffected pathogenicity | 11.64513552 | -86.4213011 | -37.3237472 | -26.432292 | -97.1377769 |
| GL_Gan1_GLEAN_10008493 | Colletotrichum lagenarium | Reduced virulence | 11.48244741 | -86.9458092 | -37.6363081 | -26.6970402 | -97.7131968 |
| GL_Gan1_GLEAN_10008497 | Cercospora nicotianae | Reduced virulence | 11.31975929 | -87.4703173 | -37.9488691 | -26.9617884 | -98.2886167 |
| GL_Gan1_GLEAN_10003294 | Claviceps purpurea | Reduced virulence | 11.15707117 | -87.9948255 | -38.26143 | -27.2265367 | -98.8640366 |
| GL_Gan1_GLEAN_10008514 | Cochliobolus heterostrophus | Reduced virulence | 10.99438306 | -88.5193336 | -38.573991 | -27.4912849 | -99.4394565 |
| GL_Gan1_GLEAN_10008515 | Aspergillus fumigatus | Reduced virulence | 10.83169494 | -89.0438417 | -38.886552 | -27.7560331 | -100.014876 |
| GL_Gan1_GLEAN_10000112 | Cochliobolus heterostrophus | Reduced virulence | 10.66900683 | -89.5683498 | -39.1991129 | -28.0207813 | -100.590296 |
| GL_Gan1_GLEAN_10000113 | Cochliobolus heterostrophus | Reduced virulence | 10.50631871 | -90.092858 | -39.5116739 | -28.2855295 | -101.165716 |
| GL_Gan1_GLEAN_10000115 | Magnaporthe grisea | Effector (plant avirulence determinant) | 10.34363059 | -90.6173661 | -39.8242348 | -28.5502777 | -101.741136 |
| GL_Gan1_GLEAN_10001148 | Fusarium oxysporum | Loss of pathogenicity | 10.18094248 | -91.1418742 | -40.1367958 | -28.8150259 | -102.316556 |
| GL_Gan1_GLEAN_10001150 | Magnaporthe grisea | Reduced virulence | 10.01825436 | -91.6663823 | -40.4493567 | -29.0797741 | -102.891976 |
| GL_Gan1_GLEAN_10001152 | Phytophthora infestans | Reduced virulence | 9.855566243 | -92.1908905 | -40.7619177 | -29.3445223 | -103.467396 |
| GL_Gan1_GLEAN_10001155 | Cryptococcus neoformans | Reduced virulence | 9.692878127 | -92.7153986 | -41.0744787 | -29.6092705 | -104.042816 |
| GL_Gan1_GLEAN_10001161 | Candida albicans | Reduced virulence | 9.53019001 | -93.2399067 | -41.3870396 | -29.8740187 | -104.618236 |
| GL_Gan1_GLEAN_10001171 | Colletotrichum lindemuthianum | Reduced virulence | 9.367501894 | -93.7644149 | -41.6996006 | -30.1387669 | -105.193656 |
| GL_Gan1_GLEAN_10001176 | Botrytis cinerea | Unaffected pathogenicity | 9.204813777 | -94.288923 | -42.0121615 | -30.4035151 | -105.769076 |
| GL_Gan1_GLEAN_10001180 | Trichoderma virens | Reduced virulence | 9.042125661 | -94.8134311 | -42.3247225 | -30.6682633 | -106.344495 |
| GL_Gan1_GLEAN_10004437 | Botrytis cinerea | Unaffected pathogenicity | 8.879437544 | -95.3379392 | -42.6372834 | -30.9330115 | -106.919915 |
| GL_Gan1_GLEAN_10004440 | Botrytis cinerea | Unaffected pathogenicity | 8.716749428 | -95.8624474 | -42.9498444 | -31.1977597 | -107.495335 |
| GL_Gan1_GLEAN_10004443 | Candida albicans | Reduced virulence | 8.554061311 | -96.3869555 | -43.2624053 | -31.4625079 | -108.070755 |
| GL_Gan1_GLEAN_10004446 | Colletotrichum acutatum | Loss of pathogenicity | 8.391373195 | -96.9114636 | -43.5749663 | -31.7272561 | -108.646175 |
| GL_Gan1_GLEAN_10004453 | Botrytis cinerea | Reduced virulence | 8.228685079 | -97.4359718 | -43.8875273 | -31.9920043 | -109.221595 |
| GL_Gan1_GLEAN_10004458 | Aspergillus fumigatus | Reduced virulence | 8.065996962 | -97.9604799 | -44.2000882 | -32.2567525 | -109.797015 |
| GL_Gan1_GLEAN_10004459 | Candida albicans | Reduced virulence | 7.903308846 | -98.484988 | -44.5126492 | -32.5215007 | -110.372435 |
| GL_Gan1_GLEAN_10004465 | Botrytis cinerea | Unaffected pathogenicity | 7.740620729 | -99.0094961 | -44.8252101 | -32.786249 | -110.947855 |
| GL_Gan1_GLEAN_10004466 | Botrytis cinerea | Reduced virulence | 7.577932613 | -99.5340043 | -45.1377711 | -33.0509972 | -111.523275 |
| GL_Gan1_GLEAN_10004467 | Candida albicans | Reduced virulence | 7.415244496 | -100.058512 | -45.450332 | -33.3157454 | -112.098695 |
| GL_Gan1_GLEAN_10005272 | Claviceps purpurea | Reduced virulence | 7.25255638 | -100.583021 | -45.762893 | -33.5804936 | -112.674114 |
| GL_Gan1_GLEAN_10004471 | Cercospora nicotianae | Reduced virulence | 7.089868263 | -101.107529 | -46.075454 | -33.8452418 | -113.249534 |
| GL_Gan1_GLEAN_10004484 | Cochliobolus carbonum | Reduced virulence | 6.927180147 | -101.632037 | -46.3880149 | -34.10999 | -113.824954 |
| GL_Gan1_GLEAN_10005805 | Claviceps purpurea | Reduced virulence | 6.76449203 | -102.156545 | -46.7005759 | -34.3747382 | -114.400374 |
| GL_Gan1_GLEAN_10004496 | Cercospora nicotianae | Reduced virulence | 6.601803914 | -102.681053 | -47.0131368 | -34.6394864 | -114.975794 |
| GL_Gan1_GLEAN_10004497 | Magnaporthe grisea | Reduced virulence | 6.439115797 | -103.205561 | -47.3256978 | -34.9042346 | -115.551214 |
| GL_Gan1_GLEAN_10004499 | Candida albicans | Reduced virulence | 6.276427681 | -103.730069 | -47.6382587 | -35.1689828 | -116.126634 |
| GL_Gan1_GLEAN_10004510 | Colletotrichum lagenarium | Loss of pathogenicity | 6.113739564 | -104.254577 | -47.9508197 | -35.433731 | -116.702054 |
| GL_Gan1_GLEAN_10004514 | Candida albicans | Reduced virulence | 5.951051448 | -104.779086 | -48.2633807 | -35.6984792 | -117.277474 |
| GL_Gan1_GLEAN_10004515 | Fusarium graminearum | Reduced virulence | 5.788363331 | -105.303594 | -48.5759416 | -35.9632274 | -117.852894 |
| GL_Gan1_GLEAN_10004520 | Fusarium graminearum | Unaffected pathogenicity | 5.625675215 | -105.828102 | -48.8885026 | -36.2279756 | -118.428314 |
| GL_Gan1_GLEAN_10004524 | Botrytis cinerea | Reduced virulence | 5.462987099 | -106.35261 | -49.2010635 | -36.4927238 | -119.003733 |
| GL_Gan1_GLEAN_10004526 | Ustilago maydis | Reduced virulence | 5.300298982 | -106.877118 | -49.5136245 | -36.757472 | -119.579153 |
| GL_Gan1_GLEAN_10004531 | Cochliobolus heterostrophus | Unaffected pathogenicity | 5.137610866 | -107.401626 | -49.8261854 | -37.0222202 | -120.154573 |
| GL_Gan1_GLEAN_10004535 | Magnaporthe grisea | Reduced virulence | 4.974922749 | -107.926134 | -50.1387464 | -37.2869684 | -120.729993 |
| GL_Gan1_GLEAN_10004536 | Candida albicans | Reduced virulence | 4.812234633 | -108.450642 | -50.4513073 | -37.5517166 | -121.305413 |
| GL_Gan1_GLEAN_10004538 | Candida albicans | Reduced virulence | 4.649546516 | -108.975151 | -50.7638683 | -37.8164648 | -121.880833 |
| GL_Gan1_GLEAN_10004543 | Botrytis cinerea | Unaffected pathogenicity | 4.4868584 | -109.499659 | -51.0764293 | -38.0812131 | -122.456253 |
| GL_Gan1_GLEAN_10000253 | Magnaporthe grisea | Reduced virulence | 4.324170283 | -110.024167 | -51.3889902 | -38.3459613 | -123.031673 |
| GL_Gan1_GLEAN_10003382 | Ustilago maydis | Reduced virulence | 4.161482167 | -110.548675 | -51.7015512 | -38.6107095 | -123.607093 |
| GL_Gan1_GLEAN_10003384 | Magnaporthe grisea | Reduced virulence | 3.99879405 | -111.073183 | -52.0141121 | -38.8754577 | -124.182513 |
| GL_Gan1_GLEAN_10003386 | Fusarium oxysporum | Reduced virulence | 3.836105934 | -111.597691 | -52.3266731 | -39.1402059 | -124.757932 |
| GL_Gan1_GLEAN_10003406 | Colletotrichum lagenarium | Loss of pathogenicity | 3.673417817 | -112.122199 | -52.639234 | -39.4049541 | -125.333352 |
| GL_Gan1_GLEAN_10003407 | Cercospora nicotianae | Reduced virulence | 3.510729701 | -112.646707 | -52.951795 | -39.6697023 | -125.908772 |
| GL_Gan1_GLEAN_10003430 | Magnaporthe grisea | Reduced virulence | 3.348041584 | -113.171216 | -53.264356 | -39.9344505 | -126.484192 |
| GL_Gan1_GLEAN_10003432 | Claviceps purpurea | Reduced virulence | 3.185353468 | -113.695724 | -53.5769169 | -40.1991987 | -127.059612 |
| GL_Gan1_GLEAN_10003433 | Magnaporthe grisea | Reduced virulence | 3.022665352 | -114.220232 | -53.8894779 | -40.4639469 | -127.635032 |
| GL_Gan1_GLEAN_10003442 | Candida albicans | Reduced virulence | 2.859977235 | -114.74474 | -54.2020388 | -40.7286951 | -128.210452 |
| GL_Gan1_GLEAN_10000583 | Cercospora nicotianae | Reduced virulence | 2.697289119 | -115.269248 | -54.5145998 | -40.9934433 | -128.785872 |
| GL_Gan1_GLEAN_10000585 | Aspergillus fumigatus | Reduced virulence | 2.534601002 | -115.793756 | -54.8271607 | -41.2581915 | -129.361292 |
| GL_Gan1_GLEAN_10000586 | Magnaporthe grisea | Effector (plant avirulence determinant) | 2.371912886 | -116.318264 | -55.1397217 | -41.5229397 | -129.936712 |
| GL_Gan1_GLEAN_10000588 | Stagonospora nodorum | Unaffected pathogenicity | 2.209224769 | -116.842772 | -55.4522826 | -41.7876879 | -130.512132 |
| GL_Gan1_GLEAN_10000589 | Candida albicans | Reduced virulence | 2.046536653 | -117.367281 | -55.7648436 | -42.0524361 | -131.087551 |
| GL_Gan1_GLEAN_10000590 | Magnaporthe grisea | Reduced virulence | 1.883848536 | -117.891789 | -56.0774046 | -42.3171843 | -131.662971 |
| GL_Gan1_GLEAN_10000593 | Cercospora nicotianae | Reduced virulence | 1.72116042 | -118.416297 | -56.3899655 | -42.5819325 | -132.238391 |
| GL_Gan1_GLEAN_10000698 | Fusarium oxysporum | Loss of pathogenicity | 1.558472303 | -118.940805 | -56.7025265 | -42.8466807 | -132.813811 |
| GL_Gan1_GLEAN_10000699 | Candida albicans | Reduced virulence | 1.395784187 | -119.465313 | -57.0150874 | -43.1114289 | -133.389231 |
| GL_Gan1_GLEAN_10005676 | Blastomyces dermatitidis | Loss of pathogenicity | 1.23309607 | -119.989821 | -57.3276484 | -43.3761771 | -133.964651 |
| GL_Gan1_GLEAN_10000720 | Fusarium oxysporum | Loss of pathogenicity | 1.070407954 | -120.514329 | -57.6402093 | -43.6409254 | -134.540071 |
| GL_Gan1_GLEAN_10001328 | Cochliobolus carbonum | Reduced virulence | 0.907719837 | -121.038837 | -57.9527703 | -43.9056736 | -135.115491 |
| GL_Gan1_GLEAN_10001329 | Magnaporthe grisea | Reduced virulence | 0.745031721 | -121.563346 | -58.2653313 | -44.1704218 | -135.690911 |
| GL_Gan1_GLEAN_10001333 | Magnaporthe grisea | Loss of pathogenicity | 0.582343605 | -122.087854 | -58.5778922 | -44.43517 | -136.266331 |
| GL_Gan1_GLEAN_10001341 | Magnaporthe grisea | Reduced virulence | 0.419655488 | -122.612362 | -58.8904532 | -44.6999182 | -136.841751 |
| GL_Gan1_GLEAN_10001344 | Colletotrichum lagenarium | Reduced virulence | 0.256967372 | -123.13687 | -59.2030141 | -44.9646664 | -137.41717 |
| GL_Gan1_GLEAN_10001357 | Botrytis cinerea | Unaffected pathogenicity | 0.094279255 | -123.661378 | -59.5155751 | -45.2294146 | -137.99259 |
| GL_Gan1_GLEAN_10002925 | Cladosporium fulvum | Reduced virulence | -0.06840886 | -124.185886 | -59.828136 | -45.4941628 | -138.56801 |
| GL_Gan1_GLEAN_10008266 | Aspergillus fumigatus | Reduced virulence | -0.23109698 | -124.710394 | -60.140697 | -45.758911 | -139.14343 |
| GL_Gan1_GLEAN_10008270 | Botrytis cinerea | Unaffected pathogenicity | -0.39378509 | -125.234903 | -60.4532579 | -46.0236592 | -139.71885 |
| GL_Gan1_GLEAN_10008271 | Cochliobolus carbonum | Unaffected pathogenicity | -0.55647321 | -125.759411 | -60.7658189 | -46.2884074 | -140.29427 |
| GL_Gan1_GLEAN_10008291 | Magnaporthe grisea | Reduced virulence | -0.71916133 | -126.283919 | -61.0783799 | -46.5531556 | -140.86969 |
| GL_Gan1_GLEAN_10007439 | Claviceps purpurea | Loss of pathogenicity | -0.88184944 | -126.808427 | -61.3909408 | -46.8179038 | -141.44511 |
| GL_Gan1_GLEAN_10008313 | Ustilago maydis | Loss of pathogenicity | -1.04453756 | -127.332935 | -61.7035018 | -47.082652 | -142.02053 |
| GL_Gan1_GLEAN_10008319 | Botrytis cinerea | Reduced virulence | -1.20722568 | -127.857443 | -62.0160627 | -47.3474002 | -142.59595 |
| GL_Gan1_GLEAN_10008327 | Fusarium oxysporum | Unaffected pathogenicity | -1.36991379 | -128.381951 | -62.3286237 | -47.6121484 | -143.17137 |
| GL_Gan1_GLEAN_10008334 | Candida albicans | Reduced virulence | -1.53260191 | -128.906459 | -62.6411846 | -47.8768966 | -143.746789 |
| GL_Gan1_GLEAN_10008341 | Fusarium graminearum | Reduced virulence | -1.69529003 | -129.430968 | -62.9537456 | -48.1416448 | -144.322209 |
| GL_Gan1_GLEAN_10008347 | Cryptococcus neoformans | Unaffected pathogenicity | -1.85797814 | -129.955476 | -63.2663066 | -48.406393 | -144.897629 |
| GL_Gan1_GLEAN_10008356 | Cladosporium fulvum | Reduced virulence | -2.02066626 | -130.479984 | -63.5788675 | -48.6711412 | -145.473049 |
| GL_Gan1_GLEAN_10008358 | Magnaporthe grisea | Reduced virulence | -2.18335438 | -131.004492 | -63.8914285 | -48.9358895 | -146.048469 |
| GL_Gan1_GLEAN_10008368 | Cercospora nicotianae | Reduced virulence | -2.34604249 | -131.529 | -64.2039894 | -49.2006377 | -146.623889 |
| GL_Gan1_GLEAN_10008369 | Colletotrichum lindemuthianum | Loss of pathogenicity | -2.50873061 | -132.053508 | -64.5165504 | -49.4653859 | -147.199309 |
| GL_Gan1_GLEAN_10000931 | Claviceps purpurea | Unaffected pathogenicity | -2.67141872 | -132.578016 | -64.8291113 | -49.7301341 | -147.774729 |
| GL_Gan1_GLEAN_10008372 | Cryptococcus neoformans | Reduced virulence | -2.83410684 | -133.102524 | -65.1416723 | -49.9948823 | -148.350149 |
| GL_Gan1_GLEAN_10008374 | Cochliobolus heterostrophus | Reduced virulence | -2.99679496 | -133.627033 | -65.4542333 | -50.2596305 | -148.925569 |
| GL_Gan1_GLEAN_10000802 | Claviceps purpurea | Reduced virulence | -3.15948307 | -134.151541 | -65.7667942 | -50.5243787 | -149.500989 |
| GL_Gan1_GLEAN_10008390 | Cryptococcus neoformans | Unaffected pathogenicity | -3.32217119 | -134.676049 | -66.0793552 | -50.7891269 | -150.076408 |
| GL_Gan1_GLEAN_10008402 | Fusarium oxysporum | Unaffected pathogenicity | -3.48485931 | -135.200557 | -66.3919161 | -51.0538751 | -150.651828 |
| GL_Gan1_GLEAN_10008404 | Saccharomyces cerevisiae | Reduced virulence | -3.64754742 | -135.725065 | -66.7044771 | -51.3186233 | -151.227248 |
| GL_Gan1_GLEAN_10008411 | Magnaporthe grisea | Reduced virulence | -3.81023554 | -136.249573 | -67.017038 | -51.5833715 | -151.802668 |
| GL_Gan1_GLEAN_10008412 | Candida albicans | Reduced virulence | -3.97292366 | -136.774081 | -67.329599 | -51.8481197 | -152.378088 |
| GL_Gan1_GLEAN_10008414 | Colletotrichum lindemuthianum | Reduced virulence | -4.13561177 | -137.298589 | -67.6421599 | -52.1128679 | -152.953508 |
| GL_Gan1_GLEAN_10000290 | Claviceps purpurea | Loss of pathogenicity | -4.29829989 | -137.823098 | -67.9547209 | -52.3776161 | -153.528928 |
| GL_Gan1_GLEAN_10008417 | Fusarium oxysporum | Unaffected pathogenicity | -4.46098801 | -138.347606 | -68.2672819 | -52.6423643 | -154.104348 |
| GL_Gan1_GLEAN_10008418 | Botrytis cinerea | Unaffected pathogenicity | -4.62367612 | -138.872114 | -68.5798428 | -52.9071125 | -154.679768 |
| GL_Gan1_GLEAN_10008422 | Colletotrichum gloeosporioides | Loss of pathogenicity | -4.78636424 | -139.396622 | -68.8924038 | -53.1718607 | -155.255188 |
| GL_Gan1_GLEAN_10001047 | Claviceps purpurea | Loss of pathogenicity | -4.94905236 | -139.92113 | -69.2049647 | -53.4366089 | -155.830608 |
| GL_Gan1_GLEAN_10008429 | Saccharomyces cerevisiae | Reduced virulence | -5.11174047 | -140.445638 | -69.5175257 | -53.7013571 | -156.406027 |
| GL_Gan1_GLEAN_10008430 | Cryptococcus neoformans | Reduced virulence | -5.27442859 | -140.970146 | -69.8300866 | -53.9661053 | -156.981447 |
| GL_Gan1_GLEAN_10008435 | Candida albicans | Unaffected pathogenicity | -5.4371167 | -141.494654 | -70.1426476 | -54.2308535 | -157.556867 |
| GL_Gan1_GLEAN_10005170 | Claviceps purpurea | Reduced virulence | -5.59980482 | -142.019163 | -70.4552086 | -54.4956018 | -158.132287 |
| GL_Gan1_GLEAN_10008443 | Colletotrichum lindemuthianum | Loss of pathogenicity | -5.76249294 | -142.543671 | -70.7677695 | -54.76035 | -158.707707 |
| GL_Gan1_GLEAN_10008447 | Magnaporthe grisea | Reduced virulence | -5.92518105 | -143.068179 | -71.0803305 | -55.0250982 | -159.283127 |
| GL_Gan1_GLEAN_10008448 | Candida albicans | Reduced virulence | -6.08786917 | -143.592687 | -71.3928914 | -55.2898464 | -159.858547 |
| GL_Gan1_GLEAN_10008459 | Ustilago maydis | Reduced virulence | -6.25055729 | -144.117195 | -71.7054524 | -55.5545946 | -160.433967 |
| GL_Gan1_GLEAN_10008465 | Colletotrichum lindemuthianum | Loss of pathogenicity | -6.4132454 | -144.641703 | -72.0180133 | -55.8193428 | -161.009387 |
| GL_Gan1_GLEAN_10008468 | Cochliobolus heterostrophus | Loss of pathogenicity | -6.57593352 | -145.166211 | -72.3305743 | -56.084091 | -161.584807 |
| GL_Gan1_GLEAN_10008469 | Aspergillus fumigatus | Reduced virulence | -6.73862164 | -145.690719 | -72.6431352 | -56.3488392 | -162.160227 |
| GL_Gan1_GLEAN_10008470 | Candida albicans | Reduced virulence | -6.90130975 | -146.215228 | -72.9556962 | -56.6135874 | -162.735646 |
| GL_Gan1_GLEAN_10003975 | Claviceps purpurea | Unaffected pathogenicity | -7.06399787 | -146.739736 | -73.2682572 | -56.8783356 | -163.311066 |
| GL_Gan1_GLEAN_10008493 | Colletotrichum lagenarium | Reduced virulence | -7.22668599 | -147.264244 | -73.5808181 | -57.1430838 | -163.886486 |
| GL_Gan1_GLEAN_10008497 | Cercospora nicotianae | Reduced virulence | -7.3893741 | -147.788752 | -73.8933791 | -57.407832 | -164.461906 |
| GL_Gan1_GLEAN_10003294 | Claviceps purpurea | Reduced virulence | -7.55206222 | -148.31326 | -74.20594 | -57.6725802 | -165.037326 |
| GL_Gan1_GLEAN_10008514 | Cochliobolus heterostrophus | Reduced virulence | -7.71475034 | -148.837768 | -74.518501 | -57.9373284 | -165.612746 |
| GL_Gan1_GLEAN_10008515 | Aspergillus fumigatus | Reduced virulence | -7.87743845 | -149.362276 | -74.8310619 | -58.2020766 | -166.188166 |
| GL_Gan1_GLEAN_10000112 | Cochliobolus heterostrophus | Reduced virulence | -8.04012657 | -149.886785 | -75.1436229 | -58.4668248 | -166.763586 |
| GL_Gan1_GLEAN_10000113 | Cochliobolus heterostrophus | Reduced virulence | -8.20281468 | -150.411293 | -75.4561839 | -58.731573 | -167.339006 |
| GL_Gan1_GLEAN_10000115 | Magnaporthe grisea | Effector (plant avirulence determinant) | -8.3655028 | -150.935801 | -75.7687448 | -58.9963212 | -167.914426 |
| GL_Gan1_GLEAN_10001148 | Fusarium oxysporum | Loss of pathogenicity | -8.52819092 | -151.460309 | -76.0813058 | -59.2610694 | -168.489846 |
| GL_Gan1_GLEAN_10001150 | Magnaporthe grisea | Reduced virulence | -8.69087903 | -151.984817 | -76.3938667 | -59.5258176 | -169.065265 |
| GL_Gan1_GLEAN_10001152 | Phytophthora infestans | Reduced virulence | -8.85356715 | -152.509325 | -76.7064277 | -59.7905658 | -169.640685 |
| GL_Gan1_GLEAN_10001155 | Cryptococcus neoformans | Reduced virulence | -9.01625527 | -153.033833 | -77.0189886 | -60.0553141 | -170.216105 |
| GL_Gan1_GLEAN_10001161 | Candida albicans | Reduced virulence | -9.17894338 | -153.558341 | -77.3315496 | -60.3200623 | -170.791525 |
| GL_Gan1_GLEAN_10001171 | Colletotrichum lindemuthianum | Reduced virulence | -9.3416315 | -154.08285 | -77.6441105 | -60.5848105 | -171.366945 |
| GL_Gan1_GLEAN_10001176 | Botrytis cinerea | Unaffected pathogenicity | -9.50431962 | -154.607358 | -77.9566715 | -60.8495587 | -171.942365 |
| GL_Gan1_GLEAN_10001180 | Trichoderma virens | Reduced virulence | -9.66700773 | -155.131866 | -78.2692325 | -61.1143069 | -172.517785 |
| GL_Gan1_GLEAN_10004437 | Botrytis cinerea | Unaffected pathogenicity | -9.82969585 | -155.656374 | -78.5817934 | -61.3790551 | -173.093205 |
| GL_Gan1_GLEAN_10004440 | Botrytis cinerea | Unaffected pathogenicity | -9.99238397 | -156.180882 | -78.8943544 | -61.6438033 | -173.668625 |
| GL_Gan1_GLEAN_10004443 | Candida albicans | Reduced virulence | -10.1550721 | -156.70539 | -79.2069153 | -61.9085515 | -174.244045 |
| GL_Gan1_GLEAN_10004446 | Colletotrichum acutatum | Loss of pathogenicity | -10.3177602 | -157.229898 | -79.5194763 | -62.1732997 | -174.819465 |
| GL_Gan1_GLEAN_10004453 | Botrytis cinerea | Reduced virulence | -10.4804483 | -157.754406 | -79.8320372 | -62.4380479 | -175.394884 |
| GL_Gan1_GLEAN_10004458 | Aspergillus fumigatus | Reduced virulence | -10.6431364 | -158.278915 | -80.1445982 | -62.7027961 | -175.970304 |
| GL_Gan1_GLEAN_10004459 | Candida albicans | Reduced virulence | -10.8058245 | -158.803423 | -80.4571592 | -62.9675443 | -176.545724 |
| GL_Gan1_GLEAN_10004465 | Botrytis cinerea | Unaffected pathogenicity | -10.9685127 | -159.327931 | -80.7697201 | -63.2322925 | -177.121144 |
| GL_Gan1_GLEAN_10004466 | Botrytis cinerea | Reduced virulence | -11.1312008 | -159.852439 | -81.0822811 | -63.4970407 | -177.696564 |
| GL_Gan1_GLEAN_10004467 | Candida albicans | Reduced virulence | -11.2938889 | -160.376947 | -81.394842 | -63.7617889 | -178.271984 |
| GL_Gan1_GLEAN_10005272 | Claviceps purpurea | Reduced virulence | -11.456577 | -160.901455 | -81.707403 | -64.0265371 | -178.847404 |
| GL_Gan1_GLEAN_10004471 | Cercospora nicotianae | Reduced virulence | -11.6192651 | -161.425963 | -82.0199639 | -64.2912853 | -179.422824 |
| GL_Gan1_GLEAN_10004484 | Cochliobolus carbonum | Reduced virulence | -11.7819532 | -161.950471 | -82.3325249 | -64.5560335 | -179.998244 |
| GL_Gan1_GLEAN_10005805 | Claviceps purpurea | Reduced virulence | -11.9446414 | -162.47498 | -82.6450859 | -64.8207817 | -180.573664 |
| GL_Gan1_GLEAN_10004496 | Cercospora nicotianae | Reduced virulence | -12.1073295 | -162.999488 | -82.9576468 | -65.0855299 | -181.149084 |
| GL_Gan1_GLEAN_10004497 | Magnaporthe grisea | Reduced virulence | -12.2700176 | -163.523996 | -83.2702078 | -65.3502782 | -181.724503 |
| GL_Gan1_GLEAN_10004499 | Candida albicans | Reduced virulence | -12.4327057 | -164.048504 | -83.5827687 | -65.6150264 | -182.299923 |
| GL_Gan1_GLEAN_10004510 | Colletotrichum lagenarium | Loss of pathogenicity | -12.5953938 | -164.573012 | -83.8953297 | -65.8797746 | -182.875343 |
| GL_Gan1_GLEAN_10004514 | Candida albicans | Reduced virulence | -12.7580819 | -165.09752 | -84.2078906 | -66.1445228 | -183.450763 |
| GL_Gan1_GLEAN_10004515 | Fusarium graminearum | Reduced virulence | -12.9207701 | -165.622028 | -84.5204516 | -66.409271 | -184.026183 |
| GL_Gan1_GLEAN_10004520 | Fusarium graminearum | Unaffected pathogenicity | -13.0834582 | -166.146536 | -84.8330125 | -66.6740192 | -184.601603 |
| GL_Gan1_GLEAN_10004524 | Botrytis cinerea | Reduced virulence | -13.2461463 | -166.671045 | -85.1455735 | -66.9387674 | -185.177023 |
| GL_Gan1_GLEAN_10004526 | Ustilago maydis | Reduced virulence | -13.4088344 | -167.195553 | -85.4581345 | -67.2035156 | -185.752443 |
| GL_Gan1_GLEAN_10004531 | Cochliobolus heterostrophus | Unaffected pathogenicity | -13.5715225 | -167.720061 | -85.7706954 | -67.4682638 | -186.327863 |
| GL_Gan1_GLEAN_10004535 | Magnaporthe grisea | Reduced virulence | -13.7342106 | -168.244569 | -86.0832564 | -67.733012 | -186.903283 |
| GL_Gan1_GLEAN_10004536 | Candida albicans | Reduced virulence | -13.8968988 | -168.769077 | -86.3958173 | -67.9977602 | -187.478703 |
| GL_Gan1_GLEAN_10004538 | Candida albicans | Reduced virulence | -14.0595869 | -169.293585 | -86.7083783 | -68.2625084 | -188.054122 |
| GL_Gan1_GLEAN_10004543 | Botrytis cinerea | Unaffected pathogenicity | -14.222275 | -169.818093 | -87.0209392 | -68.5272566 | -188.629542 |
| GL_Gan1_GLEAN_10001622 | Cryptococcus neoformans | Reduced virulence | 337.065 | 414.425 | 322.705 | 250.67 | 306.935 |
| GL_Gan1_GLEAN_10008856 | Candida albicans | Reduced virulence | 56.91 | 31.43 | 35.475 | 35.695 | 36.77 |
| GL_Gan1_GLEAN_10008860 | Candida albicans | Reduced virulence | 3.08 | 4.53 | 6.7 | 4.12 | 5.145 |
| GL_Gan1_GLEAN_10008867 | Candida albicans | Reduced virulence | 25.365 | 93.41 | 73.185 | 103.485 | 90.92 |
| GL_Gan1_GLEAN_10008877 | Candida albicans | Reduced virulence | 55.51 | 31.195 | 28.06 | 31.27 | 34.105 |
| GL_Gan1_GLEAN_10008885 | Fusarium oxysporum | Loss of pathogenicity | 2.465 | 1.63 | 1.8 | 1.88 | 1.57 |
| GL_Gan1_GLEAN_10008889 | Magnaporthe grisea | Reduced virulence | 18.535 | 6.99 | 5.165 | 5.72 | 6.165 |
| GL_Gan1_GLEAN_10008893 | Fusarium oxysporum | Loss of pathogenicity | 59.485 | 26.19 | 31.145 | 29.88 | 33.365 |
| GL_Gan1_GLEAN_10008897 | Magnaporthe grisea | Loss of pathogenicity | 77.5 | 50.775 | 36.025 | 32.895 | 43.87 |
| GL_Gan1_GLEAN_10008908 | Fusarium oxysporum | Reduced virulence | 83.215 | 51.54 | 91.52 | 105.885 | 72.88 |
| GL_Gan1_GLEAN_10008917 | Stagonospora nodorum | Unaffected pathogenicity | 55.78 | 61.695 | 50.96 | 101.875 | 53.56 |
| GL_Gan1_GLEAN_10008918 | Magnaporthe grisea | Loss of pathogenicity | 59.345 | 58.565 | 63.115 | 57.36 | 84.615 |
| GL_Gan1_GLEAN_10008925 | Magnaporthe grisea | Loss of pathogenicity | 1.76 | 23.635 | 10.59 | 17.36 | 17.62 |
| GL_Gan1_GLEAN_10003167 | Cryptococcus neoformans | Reduced virulence | 86.795 | 37.885 | 77.125 | 83.845 | 58.11 |
| GL_Gan1_GLEAN_10008928 | Botrytis cinerea | Unaffected pathogenicity | 26.025 | 13.43 | 18.63 | 15.74 | 19.39 |
| GL_Gan1_GLEAN_10008941 | Cochliobolus carbonum | Reduced virulence | 10.59 | 21.765 | 27.745 | 23.395 | 26.1 |
| GL_Gan1_GLEAN_10008944 | Cochliobolus heterostrophus | Loss of pathogenicity | 365 | 355.505 | 411.53 | 491.33 | 335.54 |
| GL_Gan1_GLEAN_10008947 | Botrytis cinerea | Reduced virulence | 533.015 | 27.85 | 30.14 | 46.965 | 18.24 |
| GL_Gan1_GLEAN_10008951 | Cercospora zeae-maydis | Reduced virulence | 288.875 | 31.645 | 49.11 | 42.94 | 26.905 |
| GL_Gan1_GLEAN_10008952 | Fusarium oxysporum | Loss of pathogenicity | 2.405 | 4.435 | 3.09 | 4.025 | 3.575 |
| GL_Gan1_GLEAN_10008953 | Magnaporthe grisea | Reduced virulence | 0 | 1.67 | 2.99 | 1.935 | 1.92 |
| GL_Gan1_GLEAN_10008959 | Cochliobolus heterostrophus | Reduced virulence | 3.595 | 37.6 | 41.26 | 50.29 | 19.76 |
| GL_Gan1_GLEAN_10008966 | Candida albicans | Reduced virulence | 11.62 | 14.6 | 9.31 | 9.325 | 12.93 |
| GL_Gan1_GLEAN_10008978 | Candida albicans | Reduced virulence | 39.22 | 104.215 | 67.435 | 72.435 | 119.475 |
| GL_Gan1_GLEAN_10008982 | Magnaporthe grisea | Reduced virulence | 69.635 | 45.195 | 29.01 | 37.175 | 30.69 |
| GL_Gan1_GLEAN_10008986 | Candida albicans | Reduced virulence | 512.48 | 78.75 | 338.97 | 113.145 | 285.92 |
| GL_Gan1_GLEAN_10008988 | Ustilago maydis | Reduced virulence | 266.015 | 265.77 | 283.88 | 261.51 | 224.305 |
| GL_Gan1_GLEAN_10008994 | Ustilago maydis | Unaffected pathogenicity | 10.125 | 0 | 1.79 | 3.01 | 1.79 |
| GL_Gan1_GLEAN_10009003 | Fusarium oxysporum | Loss of pathogenicity | 9.93 | 11.35 | 7.62 | 8.045 | 11.28 |
| GL_Gan1_GLEAN_10009004 | Magnaporthe grisea | Reduced virulence | 0 | 8.245 | 5.97 | 2.515 | 6.33 |
| GL_Gan1_GLEAN_10009007 | Botrytis cinerea | Reduced virulence | 49.06 | 41.125 | 28.035 | 20.825 | 49.075 |
| GL_Gan1_GLEAN_10009010 | Candida albicans | Reduced virulence | 4.1 | 3.07 | 2.47 | 1.33 | 4.425 |
| GL_Gan1_GLEAN_10008926 | Cryptococcus neoformans | Unaffected pathogenicity | 11.825 | 49.685 | 67.86 | 69.42 | 52.625 |
| GL_Gan1_GLEAN_10009019 | Vibrio cholerae | Reduced virulence | 36.77 | 55.16 | 47.735 | 45.99 | 53.36 |
| GL_Gan1_GLEAN_10009022 | Colletotrichum gloeosporioides | Reduced virulence | 17.16 | 92.015 | 19.06 | 16.675 | 72.845 |
| GL_Gan1_GLEAN_10009027 | Candida albicans | Reduced virulence | 11.305 | 1.045 | 2.055 | 1.25 | 1.105 |
| GL_Gan1_GLEAN_10009030 | Botrytis cinerea | Unaffected pathogenicity | 16.245 | 14.255 | 17.845 | 16.71 | 14.94 |
| GL_Gan1_GLEAN_10009032 | Candida albicans | Reduced virulence | 52.405 | 36.87 | 39.345 | 38.935 | 38.75 |
| GL_Gan1_GLEAN_10009034 | Cryptococcus neoformans | Reduced virulence | 121.335 | 190.175 | 89.04 | 59.215 | 229.88 |
| GL_Gan1_GLEAN_10009035 | Cryptococcus neoformans | Reduced virulence | 114.36 | 363.565 | 161.345 | 122.335 | 337.985 |
| GL_Gan1_GLEAN_10009044 | Stagonospora nodorum | Unaffected pathogenicity | 0 | 0.815 | 2.38 | 2.095 | 1.575 |
| GL_Gan1_GLEAN_10009046 | Botrytis cinerea | Unaffected pathogenicity | 0.82 | 0 | 0.045 | 0.075 | 0 |
| GL_Gan1_GLEAN_10009053 | Cladosporium fulvum | Reduced virulence | 0 | 2.64 | 1.065 | 1.17 | 2.625 |
| GL_Gan1_GLEAN_10009110 | Cryptococcus neoformans | Reduced virulence | 191.59 | 84.27 | 81.665 | 85.555 | 104.005 |
| GL_Gan1_GLEAN_10009061 | Mycosphaerella graminicola | Reduced virulence | 384.06 | 38.495 | 46.27 | 51.28 | 53.075 |
| GL_Gan1_GLEAN_10009062 | Mycosphaerella graminicola | Reduced virulence | 318.205 | 24.325 | 29.11 | 33.275 | 36.825 |
| GL_Gan1_GLEAN_10009066 | Candida albicans | Reduced virulence | 125.76 | 73.3 | 131.53 | 143.255 | 84.06 |
| GL_Gan1_GLEAN_10009068 | Magnaporthe grisea | Loss of pathogenicity | 0 | 0.145 | 0.225 | 0.255 | 0.105 |
| GL_Gan1_GLEAN_10009069 | Cochliobolus carbonum | Reduced virulence | 77.39 | 38.065 | 27.625 | 28.505 | 44.22 |
| GL_Gan1_GLEAN_10009076 | Magnaporthe grisea | Reduced virulence | 14.99 | 6.72 | 6.4 | 7.13 | 7.345 |
| GL_Gan1_GLEAN_10009087 | Fusarium graminearum | Reduced virulence | 20.31 | 14.995 | 16.4 | 17.29 | 20.1 |
| GL_Gan1_GLEAN_10009102 | Candida albicans | Reduced virulence | 5.41 | 14.52 | 19.62 | 13.81 | 15.595 |
| GL_Gan1_GLEAN_10009103 | Magnaporthe grisea | Reduced virulence | 719.95 | 296.315 | 262.22 | 339.165 | 151.985 |
| GL_Gan1_GLEAN_10009106 | Epichloe festucae | Wild-type mutualism | 59.285 | 10.205 | 23.24 | 16.09 | 15.02 |
| GL_Gan1_GLEAN_10006959 | Cryptococcus neoformans | Reduced virulence | 195.925 | 410.76 | 307.225 | 309.605 | 340.65 |
| GL_Gan1_GLEAN_10009115 | Fusarium oxysporum | Reduced virulence | 62.765 | 113.7 | 80.095 | 116.285 | 112.85 |
| GL_Gan1_GLEAN_10009119 | Colletotrichum lagenarium | Loss of pathogenicity | 140.695 | 201.42 | 156.845 | 163.84 | 175.11 |
| GL_Gan1_GLEAN_10009123 | Botrytis cinerea | Reduced virulence | 1.09 | 5.745 | 5.54 | 8.26 | 5.215 |
| GL_Gan1_GLEAN_10009124 | Gibberella moniliformis | Unaffected pathogenicity | 10.62 | 16.15 | 30.965 | 28.31 | 26.835 |
| GL_Gan1_GLEAN_10009128 | Colletotrichum lagenarium | Reduced virulence | 154.685 | 393.945 | 697.635 | 607.275 | 377.905 |
| GL_Gan1_GLEAN_10009132 | Cercospora nicotianae | Reduced virulence | 24.065 | 77.74 | 266.005 | 302.51 | 159.045 |
| GL_Gan1_GLEAN_10009133 | Ustilago maydis | Unaffected pathogenicity | 16.11 | 17.03 | 41.765 | 42.73 | 26.855 |
| GL_Gan1_GLEAN_10009142 | Botrytis cinerea | Reduced virulence | 5.235 | 17.62 | 14 | 13.18 | 19.69 |
| GL_Gan1_GLEAN_10009150 | Colletotrichum lagenarium | Loss of pathogenicity | 14.98 | 18.07 | 8.815 | 6.07 | 21.33 |
| GL_Gan1_GLEAN_10009157 | Fusarium oxysporum | Unaffected pathogenicity | 19.53 | 29.66 | 49.675 | 44.68 | 34.285 |
| GL_Gan1_GLEAN_10000658 | Stagonospora nodorum | Unaffected pathogenicity | 32.485 | 20.675 | 15.55 | 16.475 | 19.71 |
| GL_Gan1_GLEAN_10000668 | Botrytis cinerea | Unaffected pathogenicity | 3.57 | 0.735 | 0.905 | 1.595 | 1.22 |
| GL_Gan1_GLEAN_10006823 | Cercospora nicotianae | Reduced virulence | 46.29 | 41.72 | 29.665 | 24 | 44.795 |
| GL_Gan1_GLEAN_10006828 | Botrytis cinerea | Reduced virulence | 0 | 2.26 | 1.74 | 0.24 | 1.83 |
| GL_Gan1_GLEAN_10006830 | Magnaporthe grisea | Reduced virulence | 47.56 | 11.455 | 7.75 | 7.535 | 8 |
| GL_Gan1_GLEAN_10006831 | Magnaporthe grisea | Reduced virulence | 20.275 | 0.66 | 0.645 | 0.395 | 0.245 |
| GL_Gan1_GLEAN_10006832 | Ustilago maydis | Reduced virulence | 6.325 | 24.495 | 51.885 | 38.975 | 42.905 |
| GL_Gan1_GLEAN_10006833 | Botrytis cinerea | Reduced virulence | 6.355 | 177.205 | 175.735 | 103.745 | 360.53 |
| GL_Gan1_GLEAN_10006839 | Botrytis cinerea | Unaffected pathogenicity | 170.225 | 327.73 | 712.905 | 547.325 | 640.905 |
| GL_Gan1_GLEAN_10006840 | Botrytis cinerea | Reduced virulence | 52.56 | 9.72 | 17.095 | 13.57 | 11.075 |
| GL_Gan1_GLEAN_10006852 | Candida glabrata | Reduced virulence | 0.31 | 12.575 | 15.455 | 15.86 | 16.43 |
| GL_Gan1_GLEAN_10008610 | Cryptococcus neoformans | Reduced virulence | 21.605 | 28.79 | 26.37 | 23.78 | 32.86 |
| GL_Gan1_GLEAN_10006884 | Leptosphaeria maculans | Unaffected pathogenicity | 12.94 | 80.32 | 68.77 | 88.675 | 94.705 |
| GL_Gan1_GLEAN_10006888 | Candida albicans | Reduced virulence | 30.89 | 30.2 | 44.545 | 48.895 | 26.375 |
| GL_Gan1_GLEAN_10006899 | Stagonospora nodorum | Unaffected pathogenicity | 13.25 | 8.265 | 8.57 | 7.745 | 6.965 |
| GL_Gan1_GLEAN_10006907 | Ustilago maydis | Loss of pathogenicity | 11.395 | 12.905 | 14.255 | 15.185 | 13.295 |
| GL_Gan1_GLEAN_10006909 | Ustilago maydis | Unaffected pathogenicity | 10.595 | 5.575 | 4.885 | 7.43 | 6.96 |
| GL_Gan1_GLEAN_10005901 | Cryptococcus neoformans | Loss of pathogenicity | 342.86 | 587.25 | 578.19 | 560.99 | 560.23 |
| GL_Gan1_GLEAN_10006919 | Ustilago maydis | Unaffected pathogenicity | 9.33 | 5.97 | 7.06 | 11.24 | 6.08 |
| GL_Gan1_GLEAN_10006920 | Fusarium graminearum | Reduced virulence | 203.245 | 425.29 | 270.81 | 356.33 | 532.355 |
| GL_Gan1_GLEAN_10006923 | Stagonospora nodorum | Unaffected pathogenicity | 7.96 | 22.985 | 18.625 | 13.76 | 24.725 |
| GL_Gan1_GLEAN_10006925 | Candida albicans | Reduced virulence | 0 | 1.46 | 3.44 | 2.77 | 1.945 |
| GL_Gan1_GLEAN_10006934 | Magnaporthe grisea | Unaffected pathogenicity | 41.235 | 7.265 | 9.625 | 11.105 | 8.405 |
| GL_Gan1_GLEAN_10006935 | Salmonella enterica | Reduced virulence | 11.7 | 21.4 | 30.53 | 26.325 | 29.61 |
| GL_Gan1_GLEAN_10006943 | Candida albicans | Reduced virulence | 70.09 | 49.28 | 97.11 | 97.905 | 64.075 |
| GL_Gan1_GLEAN_10006947 | Candida albicans | Reduced virulence | 92.92 | 28.71 | 66.335 | 72.89 | 48.24 |
| GL_Gan1_GLEAN_10006952 | Magnaporthe grisea | Reduced virulence | 48.775 | 79.945 | 60.835 | 82.43 | 81.115 |
| GL_Gan1_GLEAN_10006956 | Magnaporthe grisea | Reduced virulence | 21.325 | 27.87 | 22.115 | 23.325 | 24.575 |
| GL_Gan1_GLEAN_10006957 | Candida albicans | Reduced virulence | 74.145 | 60.825 | 99.875 | 122.88 | 71.4 |
| GL_Gan1_GLEAN_10005941 | Cryptococcus neoformans | Reduced virulence | 67.56 | 43.66 | 53.235 | 57.83 | 49.67 |
| GL_Gan1_GLEAN_10006960 | Cryptococcus neoformans | Reduced virulence | 714.545 | 186.49 | 329.52 | 395.61 | 360.39 |
| GL_Gan1_GLEAN_10006972 | Botrytis cinerea | Unaffected pathogenicity | 16.015 | 1343.42 | 765.89 | 643.34 | 976.785 |
| GL_Gan1_GLEAN_10006973 | Botrytis cinerea | Reduced virulence | 16.085 | 101.71 | 304.055 | 277.905 | 266.81 |
| GL_Gan1_GLEAN_10008518 | Fusarium oxysporum | Loss of pathogenicity | 33.675 | 24 | 37.62 | 33.725 | 32.085 |
| GL_Gan1_GLEAN_10008524 | Fusarium oxysporum | Unaffected pathogenicity | 40.66 | 20.775 | 22.465 | 24.785 | 24.125 |
| GL_Gan1_GLEAN_10008532 | Mycosphaerella graminicola | Resistant to chemical | 56.65 | 163.455 | 241.13 | 230.545 | 215.52 |
| GL_Gan1_GLEAN_10008539 | Magnaporthe grisea | Reduced virulence | 1481.11 | 1785.03 | 1654.605 | 1862.85 | 1471.455 |
| GL_Gan1_GLEAN_10008541 | Pseudomonas syringae | Unaffected pathogenicity | 23.44 | 14.46 | 14.69 | 13.875 | 15.915 |
| GL_Gan1_GLEAN_10008548 | Ustilago maydis | Reduced virulence | 27.535 | 22.98 | 30.81 | 34.51 | 34.425 |
| GL_Gan1_GLEAN_10008553 | Magnaporthe grisea | Reduced virulence | 0 | 2.6 | 1.035 | 3.485 | 2.055 |
| GL_Gan1_GLEAN_10008554 | Candida albicans | Reduced virulence | 3.065 | 1.67 | 2.805 | 2.47 | 1.87 |
| GL_Gan1_GLEAN_10008558 | Magnaporthe grisea | chemistry target - phenotype unknown | 86.33 | 295.61 | 614.535 | 543.795 | 305.62 |
| GL_Gan1_GLEAN_10008574 | Botrytis cinerea | Reduced virulence | 23.435 | 10.7 | 15.43 | 12.555 | 13.17 |
| GL_Gan1_GLEAN_10008576 | Trichoderma virens | Reduced virulence | 0.425 | 27.16 | 1.77 | 2.805 | 16.74 |
| GL_Gan1_GLEAN_10008598 | Magnaporthe grisea | Reduced virulence | 49.265 | 16.115 | 16.09 | 22.32 | 14.555 |
| GL_Gan1_GLEAN_10000638 | Cryptococcus neoformans | Reduced virulence | 69.71 | 44.64 | 44.215 | 46.425 | 56.295 |
| GL_Gan1_GLEAN_10008611 | Magnaporthe grisea | Reduced virulence | 79.49 | 178.335 | 117.68 | 121.26 | 178.235 |
| GL_Gan1_GLEAN_10008622 | Magnaporthe grisea | Loss of pathogenicity | 22.655 | 22.345 | 12.57 | 12.935 | 24.44 |
| GL_Gan1_GLEAN_10008624 | Magnaporthe grisea | Reduced virulence | 83.25 | 44.05 | 41.87 | 44 | 40.995 |
| GL_Gan1_GLEAN_10008625 | Botrytis cinerea | Reduced virulence | 0 | 0.26 | 0.06 | 0.155 | 0.415 |
| GL_Gan1_GLEAN_10008631 | Candida albicans | Reduced virulence | 14.45 | 4.05 | 7.825 | 5.035 | 6.985 |
| GL_Gan1_GLEAN_10008639 | Botrytis cinerea | Reduced virulence | 1.015 | 0.23 | 0.15 | 0.23 | 0.365 |
| GL_Gan1_GLEAN_10008643 | Cercospora nicotianae | Reduced virulence | 0.885 | 3.68 | 6.225 | 4.255 | 4.85 |
| GL_Gan1_GLEAN_10008644 | Cercospora nicotianae | Reduced virulence | 9.66 | 8.465 | 6.755 | 7.31 | 7.585 |
| GL_Gan1_GLEAN_10008653 | Candida albicans | Reduced virulence | 145.97 | 125.705 | 223.425 | 265.68 | 246.34 |
| GL_Gan1_GLEAN_10008656 | Candida albicans | Reduced virulence | 17.995 | 4.795 | 7.755 | 6.51 | 6.38 |
| GL_Gan1_GLEAN_10008659 | Cochliobolus carbonum | Reduced virulence | 0 | 0 | 0 | 0 | 0 |
| GL_Gan1_GLEAN_10006322 | Cryptococcus neoformans | Reduced virulence | 55.96 | 66.855 | 76.09 | 69.61 | 83.615 |
| GL_Gan1_GLEAN_10008680 | Candida albicans | Reduced virulence | 106.42 | 40.85 | 42.455 | 37.72 | 34.215 |
| GL_Gan1_GLEAN_10008681 | Cochliobolus carbonum | Unaffected pathogenicity | 25.75 | 678.31 | 124.365 | 68.44 | 540.375 |
| GL_Gan1_GLEAN_10008683 | Aspergillus fumigatus | Loss of pathogenicity | 21.605 | 19.85 | 42.535 | 45.535 | 23.225 |
| GL_Gan1_GLEAN_10008685 | Magnaporthe grisea | Reduced virulence | 0 | 0.62 | 1.68 | 2.135 | 1.565 |
| GL_Gan1_GLEAN_10008691 | Candida albicans | Reduced virulence | 42.31 | 14.03 | 23.48 | 20.895 | 22.405 |
| GL_Gan1_GLEAN_10008692 | Gibberella moniliformis | Unaffected pathogenicity | 137.61 | 64.15 | 73.83 | 66.82 | 60.23 |
| GL_Gan1_GLEAN_10008696 | Fusarium oxysporum | Unaffected pathogenicity | 5.08 | 6.81 | 3.81 | 3.01 | 6.09 |
| GL_Gan1_GLEAN_10008705 | Cochliobolus carbonum | Unaffected pathogenicity | 7.16 | 13.73 | 0.575 | 1.04 | 10.805 |
| GL_Gan1_GLEAN_10008713 | Fusarium oxysporum | Loss of pathogenicity | 10.675 | 8.15 | 5.32 | 4.98 | 5.8 |
| GL_Gan1_GLEAN_10008717 | Botrytis cinerea | Reduced virulence | 0 | 2.345 | 1.33 | 0.99 | 1.43 |
| GL_Gan1_GLEAN_10008718 | Candida albicans | Reduced virulence | 297.79 | 1633.205 | 801.82 | 594.92 | 1540.15 |
| GL_Gan1_GLEAN_10008734 | Fusarium oxysporum | Loss of pathogenicity | 0 | 0 | 0.23 | 0.09 | 0 |
| GL_Gan1_GLEAN_10008738 | Colletotrichum lindemuthianum | Loss of pathogenicity | 5.16 | 7.425 | 6.625 | 7.825 | 8.37 |
| GL_Gan1_GLEAN_10008746 | Aspergillus fumigatus | Reduced virulence | 319.545 | 2023.46 | 3114.81 | 2779.165 | 2941.35 |
| GL_Gan1_GLEAN_10006399 | Cryptococcus neoformans | Loss of pathogenicity | 109.395 | 245.22 | 222.28 | 251.83 | 274.495 |
| GL_Gan1_GLEAN_10008757 | Gibberella moniliformis | Unaffected pathogenicity | 29.875 | 7.995 | 9 | 5.245 | 6.945 |
| GL_Gan1_GLEAN_10008760 | Fusarium graminearum | Unaffected pathogenicity | 9.35 | 4.42 | 3.075 | 2.02 | 2.385 |
| GL_Gan1_GLEAN_10008761 | Botrytis cinerea | Reduced virulence | 1.555 | 4.14 | 0.33 | 0.42 | 2.445 |
| GL_Gan1_GLEAN_10008763 | Stagonospora nodorum | Unaffected pathogenicity | 4.29 | 3.79 | 7.55 | 4.37 | 3.53 |
| GL_Gan1_GLEAN_10008771 | Candida albicans | Reduced virulence | 63.125 | 35.475 | 59.77 | 77.49 | 50.03 |
| GL_Gan1_GLEAN_10008777 | Candida albicans | Reduced virulence | 121.165 | 103.67 | 162.21 | 207.42 | 100.665 |
| GL_Gan1_GLEAN_10006481 | Cryptococcus neoformans | Reduced virulence | 1247.495 | 994.64 | 1096.17 | 1303.355 | 1081.6 |
| GL_Gan1_GLEAN_10008799 | Aspergillus fumigatus | Loss of pathogenicity | 202.745 | 128.155 | 104.63 | 104.545 | 148.115 |
| GL_Gan1_GLEAN_10000457 | Cryptococcus neoformans | Loss of pathogenicity | 3.975 | 10.93 | 7.77 | 5.79 | 10.965 |
| GL_Gan1_GLEAN_10008806 | Candida albicans | Loss of pathogenicity | 53.94 | 221.9 | 180.33 | 210.075 | 163.135 |
| GL_Gan1_GLEAN_10007861 | Cryptococcus neoformans | Increased virulence (Hypervirulence) | 55.455 | 28.07 | 43.625 | 39.315 | 44.29 |
| GL_Gan1_GLEAN_10008812 | Candida albicans | Reduced virulence | 1251.585 | 1803.14 | 2180.325 | 2094.625 | 2300.85 |
| GL_Gan1_GLEAN_10008813 | Cryphonectria parasitica | Reduced virulence | 21.87 | 24.22 | 23.47 | 18.43 | 13.625 |
| GL_Gan1_GLEAN_10008816 | Aspergillus fumigatus | Reduced virulence | 5.165 | 8.275 | 12.205 | 9.425 | 6.42 |
| GL_Gan1_GLEAN_10000214 | Alternaria alternata | Loss of pathogenicity | 3.015 | 15.555 | 13.66 | 19.565 | 15.49 |
| GL_Gan1_GLEAN_10005872 | Ustilago maydis | Reduced virulence | 8.63 | 6.445 | 6.645 | 8.17 | 9.125 |
| GL_Gan1_GLEAN_10005877 | Phytophthora infestans | Reduced virulence | 0 | 0 | 0.935 | 0.72 | 0.165 |
| GL_Gan1_GLEAN_10005878 | Candida albicans | Reduced virulence | 1.04 | 15.17 | 6.93 | 8.14 | 15.375 |
| GL_Gan1_GLEAN_10005890 | Fusarium oxysporum | Unaffected pathogenicity | 76.365 | 32.93 | 29.805 | 26.395 | 38.585 |
| GL_Gan1_GLEAN_10005892 | Botrytis cinerea | Unaffected pathogenicity | 188.705 | 20.06 | 14.69 | 13.07 | 15.8 |
| GL_Gan1_GLEAN_10005898 | Fusarium oxysporum | Loss of pathogenicity | 38.85 | 10.35 | 10.785 | 8.905 | 11.705 |
| GL_Gan1_GLEAN_10007941 | Cryptococcus neoformans | Loss of pathogenicity | 115.01 | 94.425 | 95.495 | 109.835 | 122.515 |
| GL_Gan1_GLEAN_10005910 | Aspergillus fumigatus | Reduced virulence | 33.595 | 98.65 | 95.07 | 88.925 | 115.885 |
| GL_Gan1_GLEAN_10005912 | Alternaria alternata | Loss of pathogenicity | 18.095 | 7.58 | 10.085 | 7.485 | 9.03 |
| GL_Gan1_GLEAN_10005914 | Fusarium oxysporum | Reduced virulence | 170.1 | 142.865 | 208.995 | 234.13 | 200.875 |
| GL_Gan1_GLEAN_10005924 | Magnaporthe grisea | Reduced virulence | 1.305 | 0.575 | 2.925 | 1.455 | 1.91 |
| GL_Gan1_GLEAN_10005928 | Botrytis cinerea | Unaffected pathogenicity | 0 | 0.565 | 0.26 | 0.495 | 1.735 |
| GL_Gan1_GLEAN_10005933 | Colletotrichum lagenarium | Reduced virulence | 28.89 | 42.205 | 33.405 | 26.38 | 39.985 |
| GL_Gan1_GLEAN_10005937 | Candida albicans | Reduced virulence | 0 | 15.7 | 14.055 | 12.86 | 21.095 |
| GL_Gan1_GLEAN_10008002 | Cryptococcus neoformans | Loss of pathogenicity | 45.115 | 71.325 | 87.885 | 93.405 | 80.505 |
| GL_Gan1_GLEAN_10005945 | Candida albicans | Reduced virulence | 126.645 | 107.94 | 101.67 | 139.01 | 102.605 |
| GL_Gan1_GLEAN_10005946 | Fusarium oxysporum | Increased virulence (Hypervirulence) | 8.675 | 22.44 | 23.03 | 20.73 | 21.92 |
| GL_Gan1_GLEAN_10005948 | Botrytis cinerea | Unaffected pathogenicity | 138.255 | 119.035 | 195.165 | 209.795 | 173.73 |
| GL_Gan1_GLEAN_10005955 | Cercospora nicotianae | Reduced virulence | 19.35 | 19.77 | 38.275 | 41.335 | 31.235 |
| GL_Gan1_GLEAN_10005956 | Cochliobolus carbonum | Loss of pathogenicity | 10.565 | 22.245 | 22.775 | 31.175 | 29.95 |
| GL_Gan1_GLEAN_10005957 | Candida albicans | Reduced virulence | 8.72 | 32.265 | 23.24 | 53.78 | 63.505 |
| GL_Gan1_GLEAN_10005958 | Candida albicans | Reduced virulence | 0 | 0 | 0.05 | 0.08 | 0.155 |
| GL_Gan1_GLEAN_10005965 | Candida albicans | Reduced virulence | 14.04 | 77.77 | 51.415 | 12.96 | 47.945 |
| GL_Gan1_GLEAN_10005967 | Magnaporthe grisea | Loss of pathogenicity | 1.14 | 0.255 | 0.29 | 0.205 | 0.315 |
| GL_Gan1_GLEAN_10005970 | Magnaporthe grisea | Reduced virulence | 0 | 0 | 0 | 0 | 0 |
| GL_Gan1_GLEAN_10005971 | Aspergillus fumigatus | Loss of pathogenicity | 51.535 | 59.735 | 61.52 | 69.125 | 65.015 |
| GL_Gan1_GLEAN_10005974 | Candida albicans | Reduced virulence | 10.6 | 24.76 | 23.73 | 41.35 | 31.75 |
| GL_Gan1_GLEAN_10005984 | Magnaporthe grisea | Reduced virulence | 186.315 | 80.38 | 181.31 | 191.625 | 119.965 |
| GL_Gan1_GLEAN_10005991 | Magnaporthe grisea | Loss of pathogenicity | 10.81 | 11.09 | 10.36 | 10.135 | 16.905 |
| GL_Gan1_GLEAN_10001907 | Fusarium oxysporum | Unaffected pathogenicity | 35.43 | 15.695 | 18.655 | 20.595 | 16.335 |
| GL_Gan1_GLEAN_10001908 | Candida albicans | Reduced virulence | 98.24 | 42.365 | 67.215 | 66.225 | 63.305 |
| GL_Gan1_GLEAN_10001915 | Botrytis cinerea | Unaffected pathogenicity | 99.075 | 10.035 | 11.325 | 13.295 | 8.015 |
| GL_Gan1_GLEAN_10001926 | Cryptococcus neoformans | Reduced virulence | 56.82 | 29.8 | 56.05 | 57.675 | 38.57 |
| GL_Gan1_GLEAN_10001935 | Colletotrichum gloeosporioides | Unaffected pathogenicity | 3.465 | 0.355 | 0.67 | 0.145 | 1.22 |
| GL_Gan1_GLEAN_10000855 | Saccharomyces cerevisiae | Reduced virulence | 3.4 | 22.815 | 14.98 | 14.495 | 23.86 |
| GL_Gan1_GLEAN_10000857 | Botrytis cinerea | Unaffected pathogenicity | 0 | 0.185 | 0 | 0.04 | 0.975 |
| GL_Gan1_GLEAN_10000865 | Botrytis cinerea | Reduced virulence | 7.935 | 2.77 | 3.45 | 4.525 | 4.71 |
| GL_Gan1_GLEAN_10000866 | Botrytis cinerea | Unaffected pathogenicity | 5.105 | 54.305 | 29.125 | 32.245 | 44.045 |
| GL_Gan1_GLEAN_10001189 | Cryptococcus neoformans | Reduced virulence | 118.21 | 47.92 | 104.88 | 103.36 | 47.395 |
| GL_Gan1_GLEAN_10000871 | Candida albicans | Reduced virulence | 50.99 | 47.72 | 41.61 | 35.015 | 68.845 |
| GL_Gan1_GLEAN_10000881 | Candida albicans | Reduced virulence | 52.95 | 221.64 | 175.99 | 187.19 | 186.745 |
| GL_Gan1_GLEAN_10000882 | Botrytis cinerea | Unaffected pathogenicity | 9.545 | 4.18 | 4.635 | 5.365 | 4.615 |
| GL_Gan1_GLEAN_10000883 | Magnaporthe grisea | Loss of pathogenicity | 104.625 | 49.38 | 50.55 | 58.405 | 48.66 |
| GL_Gan1_GLEAN_10000011 | Phytophthora sojae | Effector (plant avirulence determinant) | 24.63 | 1.485 | 3.025 | 2.025 | 1.275 |
| GL_Gan1_GLEAN_10003827 | Colletotrichum lindemuthianum | Loss of pathogenicity | 5.055 | 11.01 | 10.125 | 9.85 | 11.42 |
| GL_Gan1_GLEAN_10003828 | Botrytis cinerea | Unaffected pathogenicity | 3.685 | 5.105 | 15.69 | 11.21 | 5.295 |
| GL_Gan1_GLEAN_10003835 | Botrytis cinerea | Unaffected pathogenicity | 3.34 | 1.285 | 2.045 | 1.695 | 1.16 |
| GL_Gan1_GLEAN_10003836 | Fusarium oxysporum | Loss of pathogenicity | 7.31 | 104.405 | 132.685 | 139.01 | 140.77 |
| GL_Gan1_GLEAN_10003843 | Fusarium oxysporum | Loss of pathogenicity | 27.3 | 13.515 | 22.3 | 19.865 | 20.17 |
| GL_Gan1_GLEAN_10003846 | Fusarium graminearum | Reduced virulence | 19.56 | 74.23 | 81.64 | 85.3 | 61.115 |
| GL_Gan1_GLEAN_10003848 | Magnaporthe grisea | Reduced virulence | 1.475 | 19.96 | 15.615 | 18.315 | 30.535 |
| GL_Gan1_GLEAN_10003855 | Magnaporthe grisea | Reduced virulence | 13.72 | 69.58 | 68.075 | 69.215 | 102.42 |
| GL_Gan1_GLEAN_10003869 | Alternaria alternata | Loss of pathogenicity | 34.95 | 6.655 | 9.1 | 6.62 | 8.515 |
| GL_Gan1_GLEAN_10003870 | Ustilago maydis | Loss of pathogenicity | 116.89 | 63.06 | 79.975 | 84.745 | 75.94 |
| GL_Gan1_GLEAN_10003877 | Colletotrichum lagenarium | Loss of pathogenicity | 4.4 | 1.775 | 1.91 | 1.955 | 2.405 |
| GL_Gan1_GLEAN_10003878 | Alternaria alternata | Loss of pathogenicity | 5.015 | 16.79 | 32.54 | 21.135 | 25.41 |
| GL_Gan1_GLEAN_10003879 | Aspergillus fumigatus | Loss of pathogenicity | 7.92 | 18.89 | 20.42 | 17.105 | 28.94 |
| GL_Gan1_GLEAN_10003891 | Colletotrichum trifolii | Loss of pathogenicity | 18.59 | 37 | 24.21 | 24.03 | 37.265 |
| GL_Gan1_GLEAN_10003895 | Magnaporthe grisea | Reduced virulence | 44.34 | 22.68 | 46.68 | 38.545 | 31.225 |
| GL_Gan1_GLEAN_10003896 | Magnaporthe grisea | Reduced virulence | 29.555 | 7.865 | 10.915 | 11.62 | 8.45 |
| GL_Gan1_GLEAN_10002051 | Candida albicans | Reduced virulence | 1.2 | 8.215 | 6.15 | 5.355 | 8.015 |
| GL_Gan1_GLEAN_10002065 | Magnaporthe grisea | Reduced virulence | 7.53 | 0.8 | 1 | 0.64 | 1.38 |
| GL_Gan1_GLEAN_10002071 | Ustilago maydis | Loss of pathogenicity | 7.79 | 47.78 | 72.385 | 67.03 | 51.545 |
| GL_Gan1_GLEAN_10002074 | Ustilago maydis | Reduced virulence | 33.075 | 40.865 | 25.19 | 20.69 | 46.375 |
| GL_Gan1_GLEAN_10002075 | Magnaporthe grisea | Unaffected pathogenicity | 45.825 | 33.835 | 63.09 | 51.025 | 47.275 |
| GL_Gan1_GLEAN_10002083 | Mycosphaerella graminicola | Sensitive to chemical | 4.965 | 76.145 | 129.655 | 107.04 | 109.135 |
| GL_Gan1_GLEAN_10002085 | Magnaporthe grisea | Reduced virulence | 2.835 | 1.99 | 1.835 | 1.2 | 0.815 |
| GL_Gan1_GLEAN_10002102 | Botrytis cinerea | Unaffected pathogenicity | 0 | 21.135 | 11.355 | 5.28 | 21.875 |
| GL_Gan1_GLEAN_10002105 | Candida albicans | Reduced virulence | 0 | 0.435 | 0.355 | 0.82 | 1.05 |
| GL_Gan1_GLEAN_10002108 | Ustilago maydis | Reduced virulence | 24.415 | 15.29 | 20.485 | 18.225 | 22.37 |
| GL_Gan1_GLEAN_10001076 | Cryptococcus neoformans | Reduced virulence | 160.295 | 104.415 | 177.64 | 203.615 | 116.085 |
| GL_Gan1_GLEAN_10000804 | Cochliobolus heterostrophus | Loss of pathogenicity | 86.375 | 93.86 | 102.7 | 115.685 | 90.35 |
| GL_Gan1_GLEAN_10000807 | Magnaporthe grisea | Reduced virulence | 48.975 | 51.965 | 80.645 | 79.245 | 51.52 |
| GL_Gan1_GLEAN_10000813 | Magnaporthe grisea | Reduced virulence | 0 | 1.04 | 0.18 | 0.08 | 1.745 |
| GL_Gan1_GLEAN_10007200 | Cryptococcus neoformans | Reduced virulence | 2.935 | 15.645 | 14.05 | 7.45 | 19.325 |
| GL_Gan1_GLEAN_10000816 | Botrytis cinerea | Reduced virulence | 0 | 4.665 | 0.36 | 0.3 | 3.965 |
| GL_Gan1_GLEAN_10000827 | Candida albicans | Reduced virulence | 78.595 | 38.605 | 76.185 | 86.2 | 50.435 |
| GL_Gan1_GLEAN_10000830 | Botrytis cinerea | Reduced virulence | 9.075 | 8.995 | 7.23 | 9.365 | 9.495 |
| GL_Gan1_GLEAN_10001377 | Colletotrichum lagenarium | Loss of pathogenicity | 68.805 | 37.805 | 46.87 | 53.93 | 50.92 |
| GL_Gan1_GLEAN_10001378 | Verticillium fungicola | Reduced virulence | 1.375 | 9.715 | 5.595 | 5.365 | 6.585 |
| GL_Gan1_GLEAN_10001379 | Salmonella enterica |  | 7.485 | 1.935 | 5.075 | 2.36 | 1.525 |
| GL_Gan1_GLEAN_10001401 | Botrytis cinerea | Unaffected pathogenicity | 12.64 | 14.185 | 8.275 | 8.33 | 10.98 |
| GL_Gan1_GLEAN_10001402 | Ustilago maydis | Unaffected pathogenicity | 3.745 | 0.77 | 0.74 | 0.31 | 0.695 |
| GL_Gan1_GLEAN_10000368 | Fusarium graminearum | Unaffected pathogenicity | 68.085 | 15.21 | 19.89 | 17.745 | 17.765 |
| GL_Gan1_GLEAN_10000373 | Magnaporthe grisea | Reduced virulence | 217.92 | 373.52 | 387.63 | 437.035 | 424.64 |
| GL_Gan1_GLEAN_10000188 | Ustilago maydis | Unaffected pathogenicity | 9.67 | 21.44 | 17.75 | 17.095 | 24.935 |
| GL_Gan1_GLEAN_10000191 | Fusarium graminearum | Reduced virulence | 7.435 | 48.26 | 21.735 | 24.055 | 48.9 |
| GL_Gan1_GLEAN_10000196 | Fusarium graminearum | Reduced virulence | 1.19 | 0.785 | 0.285 | 0.315 | 0 |
| GL_Gan1_GLEAN_10000086 | Trichoderma virens | Reduced virulence | 4.935 | 7.35 | 15.21 | 15.74 | 19.955 |
| GL_Gan1_GLEAN_10002670 | Colletotrichum lagenarium | Reduced virulence | 0 | 2.33 | 3.555 | 3.105 | 3.33 |
| GL_Gan1_GLEAN_10002683 | Stagonospora nodorum | Unaffected pathogenicity | 0 | 0.31 | 0.12 | 0.125 | 0 |
| GL_Gan1_GLEAN_10002684 | Botrytis cinerea | Reduced virulence | 0.74 | 4.575 | 4.97 | 3.83 | 5.08 |
| GL_Gan1_GLEAN_10002685 | Fusarium oxysporum | Unaffected pathogenicity | 1.825 | 3.495 | 3.665 | 3.52 | 3.145 |
| GL_Gan1_GLEAN_10002687 | Aspergillus fumigatus | Reduced virulence | 4.765 | 19.145 | 7.46 | 12.605 | 20.58 |
| GL_Gan1_GLEAN_10002693 | Salmonella enterica | Reduced virulence | 129.18 | 216.47 | 159.045 | 164.905 | 194.935 |
| GL_Gan1_GLEAN_10002695 | Ustilago maydis | Unaffected pathogenicity | 5.65 | 4.37 | 4.98 | 3.65 | 8.455 |
| GL_Gan1_GLEAN_10002697 | Magnaporthe grisea | Reduced virulence | 31.905 | 5.93 | 11.32 | 8.46 | 5.195 |
| GL_Gan1_GLEAN_10002699 | Ustilago maydis | Unaffected pathogenicity | 9.91 | 11.985 | 5.145 | 5.4 | 17.92 |
| GL_Gan1_GLEAN_10002707 | Colletotrichum lagenarium | Loss of pathogenicity | 11.81 | 36.61 | 64.355 | 43.63 | 71.775 |
| GL_Gan1_GLEAN_10002720 | Ustilago maydis | Unaffected pathogenicity | 2.89 | 0.295 | 0.035 | 0.065 | 0.35 |
| GL_Gan1_GLEAN_10007223 | Cryptococcus neoformans | Reduced virulence | 40.225 | 898.65 | 604.92 | 563.55 | 758.28 |
| GL_Gan1_GLEAN_10002466 | Fusarium oxysporum | Unaffected pathogenicity | 28.19 | 13.09 | 15.165 | 10.8 | 13.285 |
| GL_Gan1_GLEAN_10007279 | Cryptococcus neoformans | Reduced virulence | 14.68 | 26.97 | 33.625 | 28.28 | 32.595 |
| GL_Gan1_GLEAN_10002474 | Ustilago maydis | Reduced virulence | 8.77 | 10.22 | 5.17 | 5.04 | 12.66 |
| GL_Gan1_GLEAN_10002606 | Cryptococcus neoformans | Loss of pathogenicity | 170.54 | 295.215 | 188.595 | 192.35 | 257.29 |
| GL_Gan1_GLEAN_10002488 | Botrytis cinerea | Reduced virulence | 5.6 | 2.725 | 2.14 | 1.72 | 2.82 |
| GL_Gan1_GLEAN_10002492 | Alternaria alternata | Loss of pathogenicity | 25.56 | 14.51 | 13.715 | 10 | 13.675 |
| GL_Gan1_GLEAN_10002497 | Cercospora nicotianae | Reduced virulence | 11.14 | 2 | 1.905 | 1.79 | 2.65 |
| GL_Gan1_GLEAN_10002500 | Fusarium graminearum | Reduced virulence | 28.78 | 92.835 | 61.54 | 48.64 | 111.595 |
| GL_Gan1_GLEAN_10002502 | Magnaporthe grisea | Reduced virulence | 42.08 | 82.87 | 116.165 | 105.495 | 91.885 |
| GL_Gan1_GLEAN_10002513 | Botrytis cinerea | Unaffected pathogenicity | 14.285 | 5.95 | 12.1 | 9.175 | 10.79 |
| GL_Gan1_GLEAN_10002518 | Candida albicans | Reduced virulence | 16.605 | 225.855 | 33.375 | 36.705 | 209.28 |
| GL_Gan1_GLEAN_10002519 | Fusarium oxysporum | Unaffected pathogenicity | 3.605 | 1.98 | 2.97 | 3.365 | 3.16 |
| GL_Gan1_GLEAN_10002521 | Cryptococcus neoformans | Unaffected pathogenicity | 0 | 8.96 | 1.205 | 2.455 | 6.47 |
| GL_Gan1_GLEAN_10002527 | Nectria haematococca (related: Fusarium solani) | Reduced virulence | 0 | 0.205 | 0.245 | 0.125 | 0 |
| GL_Gan1_GLEAN_10002611 | Cryptococcus neoformans | Reduced virulence | 81.285 | 96.74 | 71.86 | 76.935 | 104.17 |
| GL_Gan1_GLEAN_10002530 | Magnaporthe grisea | Loss of pathogenicity | 0 | 1.765 | 2.385 | 2.755 | 1.405 |
| GL_Gan1_GLEAN_10004122 | Botrytis cinerea | Reduced virulence | 2.335 | 75.685 | 17.2 | 54.625 | 65.945 |
| GL_Gan1_GLEAN_10004126 | Botrytis cinerea | Unaffected pathogenicity | 16.96 | 14.42 | 11.3 | 8.02 | 13.84 |
| GL_Gan1_GLEAN_10001605 | Cryptococcus neoformans | Reduced virulence | 174.46 | 68.02 | 96.175 | 110.255 | 72.6 |
| GL_Gan1_GLEAN_10004130 | Botrytis cinerea | Reduced virulence | 7.715 | 35.715 | 29.725 | 50.95 | 27.175 |
| GL_Gan1_GLEAN_10004147 | Fusarium oxysporum | Loss of pathogenicity | 81.425 | 41.44 | 50.655 | 56.055 | 38.895 |
| GL_Gan1_GLEAN_10004861 | Cryptococcus neoformans | Reduced virulence | 101 | 48.11 | 98.8 | 112.08 | 72.17 |
| GL_Gan1_GLEAN_10004164 | Magnaporthe grisea | Reduced virulence | 40.4 | 36.82 | 77.415 | 60.18 | 48.74 |
| GL_Gan1_GLEAN_10004173 | Magnaporthe grisea | Reduced virulence | 41.97 | 681.3 | 446.525 | 644.68 | 747.65 |
| GL_Gan1_GLEAN_10004182 | Magnaporthe grisea | Reduced virulence | 0 | 1.85 | 3.06 | 2.705 | 4.07 |
| GL_Gan1_GLEAN_10004191 | Cercospora nicotianae | Reduced virulence | 0 | 0.755 | 0.18 | 0.56 | 0.68 |
| GL_Gan1_GLEAN_10004192 | Gibberella moniliformis | Unaffected pathogenicity | 6.05 | 3.095 | 5.48 | 4.47 | 3.695 |
| GL_Gan1_GLEAN_10004194 | Salmonella enterica | Reduced virulence | 112.655 | 61.75 | 49.25 | 46.3 | 73.55 |
| GL_Gan1_GLEAN_10004199 | Magnaporthe grisea | Reduced virulence | 32.475 | 42.09 | 61.165 | 47.18 | 65.65 |
| GL_Gan1_GLEAN_10004200 | Candida albicans | Reduced virulence | 8.79 | 27.62 | 36.77 | 30.55 | 36.215 |
| GL_Gan1_GLEAN_10004203 | Fusarium oxysporum | Increased virulence (Hypervirulence) | 34.97 | 41.615 | 24.985 | 20.97 | 48.965 |
| GL_Gan1_GLEAN_10004208 | Candida albicans | Unaffected pathogenicity | 2.465 | 11.85 | 7.055 | 7.42 | 14.39 |
| GL_Gan1_GLEAN_10004209 | Botrytis cinerea | Unaffected pathogenicity | 0 | 0.47 | 0.125 | 0.185 | 0.425 |
| GL_Gan1_GLEAN_10004218 | Colletotrichum gloeosporioides | Reduced virulence | 230.685 | 13.025 | 9.06 | 10.195 | 8.985 |
| GL_Gan1_GLEAN_10002334 | Cryptococcus neoformans | Unaffected pathogenicity | 101.095 | 90.235 | 133.31 | 116.93 | 68.185 |
| GL_Gan1_GLEAN_10004029 | Cryptococcus neoformans | Loss of pathogenicity | 11.365 | 19.625 | 23.45 | 25 | 17.715 |
| GL_Gan1_GLEAN_10002202 | Cryptococcus neoformans | Reduced virulence | 35.13 | 183.65 | 95.465 | 84.52 | 151.885 |
| GL_Gan1_GLEAN_10006325 | Candida albicans | Reduced virulence | 43.71 | 65.335 | 59.375 | 61.03 | 88.69 |
| GL_Gan1_GLEAN_10006326 | Magnaporthe grisea | Reduced virulence | 57.61 | 10.265 | 11.955 | 14.04 | 11.35 |
| GL_Gan1_GLEAN_10006334 | Candida albicans | Reduced virulence | 140.65 | 169.665 | 148.255 | 103.595 | 101.17 |
| GL_Gan1_GLEAN_10006335 | Cryptococcus neoformans | Reduced virulence | 36.37 | 18.405 | 25.58 | 28.42 | 23.105 |
| GL_Gan1_GLEAN_10006338 | Fusarium graminearum | Reduced virulence | 35.38 | 59.71 | 52.04 | 45.525 | 55.935 |
| GL_Gan1_GLEAN_10006342 | Candida albicans | Reduced virulence | 5.03 | 27.765 | 24.295 | 24.89 | 30.54 |
| GL_Gan1_GLEAN_10006345 | Magnaporthe grisea | Reduced virulence | 33.34 | 30.285 | 29.775 | 28.56 | 37.35 |
| GL_Gan1_GLEAN_10006350 | Candida albicans | Reduced virulence | 8.065 | 31.76 | 36.455 | 37.69 | 34.27 |
| GL_Gan1_GLEAN_10006364 | Mycosphaerella graminicola | Loss of pathogenicity | 21.235 | 69.665 | 57.285 | 57.455 | 76.285 |
| GL_Gan1_GLEAN_10006381 | Magnaporthe grisea | Reduced virulence | 0 | 6.28 | 18.71 | 6.335 | 7.785 |
| GL_Gan1_GLEAN_10006382 | Botrytis cinerea | Reduced virulence | 2.085 | 13.57 | 18.495 | 5.005 | 17.805 |
| GL_Gan1_GLEAN_10001254 | Cryptococcus neoformans | Loss of pathogenicity | 94.9 | 187.915 | 101.735 | 119.5 | 141.455 |
| GL_Gan1_GLEAN_10006403 | Candida albicans | Loss of pathogenicity | 9.845 | 88.655 | 60.17 | 70.885 | 98.1 |
| GL_Gan1_GLEAN_10006417 | Magnaporthe grisea | Reduced virulence | 71.675 | 204.06 | 181.115 | 169.095 | 206.54 |
| GL_Gan1_GLEAN_10006430 | Colletotrichum lindemuthianum | Loss of pathogenicity | 2.055 | 16.555 | 18.035 | 22.625 | 13.985 |
| GL_Gan1_GLEAN_10006460 | Ustilago maydis | Reduced virulence | 21.805 | 13.08 | 12.505 | 14.42 | 19.03 |
| GL_Gan1_GLEAN_10006467 | Colletotrichum lagenarium | Reduced virulence | 0 | 0 | 0.575 | 0.905 | 0.31 |
| GL_Gan1_GLEAN_10006468 | Gibberella moniliformis | Unaffected pathogenicity | 0 | 13.82 | 11.015 | 9.51 | 13.835 |
| GL_Gan1_GLEAN_10006474 | Botrytis cinerea | Reduced virulence | 0 | 0 | 0 | 0 | 0 |
| GL_Gan1_GLEAN_10003303 | Cryptococcus neoformans | Reduced virulence | 350.87 | 59.735 | 63.575 | 63.365 | 92.775 |
| GL_Gan1_GLEAN_10006488 | Magnaporthe grisea | Loss of pathogenicity | 7.52 | 18.64 | 15.55 | 15.49 | 22.29 |
| GL_Gan1_GLEAN_10000449 | Candida albicans | Reduced virulence | 7.47 | 19.56 | 11.25 | 15.125 | 15.945 |
| GL_Gan1_GLEAN_10000455 | Magnaporthe grisea | Loss of pathogenicity | 5.255 | 6.39 | 4.925 | 4.475 | 6.61 |
| GL_Gan1_GLEAN_10006245 | Cryptococcus neoformans | Reduced virulence | 4.26 | 1.19 | 1.33 | 1.82 | 1.13 |
| GL_Gan1_GLEAN_10003073 | Ustilago maydis | Unaffected pathogenicity | 18.68 | 25.735 | 34.64 | 32.575 | 34.165 |
| GL_Gan1_GLEAN_10003074 | Stagonospora nodorum | Unaffected pathogenicity | 0 | 0.21 | 0.05 | 0 | 0.26 |
| GL_Gan1_GLEAN_10003082 | Ustilago maydis | Reduced virulence | 32.86 | 91.535 | 58.315 | 57.885 | 111.61 |
| GL_Gan1_GLEAN_10003086 | Fusarium oxysporum | Reduced virulence | 146.005 | 128.21 | 145.305 | 163.46 | 143.11 |
| GL_Gan1_GLEAN_10003089 | Cochliobolus heterostrophus | Reduced virulence | 18.375 | 14.105 | 9.44 | 10.34 | 10.905 |
| GL_Gan1_GLEAN_10003093 | Stagonospora nodorum | Unaffected pathogenicity | 0 | 37.515 | 4.555 | 5.345 | 40.405 |
| GL_Gan1_GLEAN_10003094 | Cryptococcus neoformans | Reduced virulence | 153.265 | 205.485 | 276.645 | 147.755 | 220.175 |
| GL_Gan1_GLEAN_10003103 | Ustilago maydis | Loss of pathogenicity | 7.715 | 46.855 | 43.28 | 50.57 | 57.6 |
| GL_Gan1_GLEAN_10005344 | Cryptococcus neoformans | Reduced virulence | 4.29 | 7.435 | 5.4 | 5.03 | 6.34 |
| GL_Gan1_GLEAN_10003111 | Fusarium oxysporum | Unaffected pathogenicity | 11.14 | 22.985 | 19.835 | 14.065 | 32.23 |
| GL_Gan1_GLEAN_10003112 | Magnaporthe grisea | Reduced virulence | 13.595 | 18.415 | 15.73 | 12.48 | 21.205 |
| GL_Gan1_GLEAN_10003117 | Fusarium oxysporum | Reduced virulence | 121.97 | 427.38 | 878.275 | 549.34 | 411.39 |
| GL_Gan1_GLEAN_10003118 | Aspergillus fumigatus | Reduced virulence | 556.38 | 949.215 | 896.93 | 753.92 | 1003.845 |
| GL_Gan1_GLEAN_10003120 | Fusarium graminearum | Reduced virulence | 12.035 | 46.605 | 51.035 | 41.765 | 52.415 |
| GL_Gan1_GLEAN_10003123 | Magnaporthe grisea | Loss of pathogenicity | 14.85 | 31.795 | 26.165 | 20.37 | 31.94 |
| GL_Gan1_GLEAN_10003125 | Botrytis cinerea | Unaffected pathogenicity | 113.305 | 16.805 | 19.62 | 6.975 | 28.535 |
| GL_Gan1_GLEAN_10003139 | Ustilago maydis | Reduced virulence | 85.625 | 158.135 | 117.795 | 113.29 | 142.43 |
| GL_Gan1_GLEAN_10003155 | Colletotrichum lindemuthianum | Reduced virulence | 61.55 | 96.105 | 104.715 | 91.665 | 80.555 |
| GL_Gan1_GLEAN_10003156 | Candida albicans | Reduced virulence | 79.305 | 151.575 | 149.245 | 143.435 | 240.895 |
| GL_Gan1_GLEAN_10003157 | Botrytis cinerea | Unaffected pathogenicity | 68.88 | 38.715 | 45.875 | 35.405 | 44.14 |
| GL_Gan1_GLEAN_10003159 | Coccidioides posadasii | Reduced virulence | 42.075 | 23.565 | 55.495 | 37.41 | 36.275 |
| GL_Gan1_GLEAN_10000349 | Cochliobolus heterostrophus | Reduced virulence | 224.09 | 51.37 | 52.795 | 66.87 | 40.09 |
| GL_Gan1_GLEAN_10000359 | Magnaporthe grisea | Reduced virulence | 18.39 | 12.445 | 13.15 | 14.135 | 17.065 |
| GL_Gan1_GLEAN_10000363 | Candida albicans | Reduced virulence | 18.08 | 4.895 | 7.51 | 6.895 | 3.785 |
| GL_Gan1_GLEAN_10000364 | Colletotrichum lagenarium | Reduced virulence | 0 | 7.535 | 2.5 | 2.325 | 8.755 |
| GL_Gan1_GLEAN_10000365 | Cercospora nicotianae | Reduced virulence | 0.64 | 3.215 | 2.14 | 3.94 | 2.57 |
| GL_Gan1_GLEAN_10000366 | Stagonospora nodorum | Unaffected pathogenicity | 0 | 0.785 | 1.15 | 1.38 | 0.88 |
| GL_Gan1_GLEAN_10003638 | Candida albicans | Reduced virulence | 1.435 | 2.745 | 2.105 | 1.79 | 2.44 |
| GL_Gan1_GLEAN_10003642 | Candida albicans | Loss of pathogenicity | 164.195 | 76.725 | 102.98 | 86.515 | 88.105 |
| GL_Gan1_GLEAN_10003645 | Candida albicans | Reduced virulence | 158.57 | 32.345 | 32.835 | 31.13 | 38.565 |
| GL_Gan1_GLEAN_10003654 | Cochliobolus heterostrophus | Reduced virulence | 0 | 0.885 | 0.385 | 0.405 | 0.815 |
| GL_Gan1_GLEAN_10003655 | Candida albicans | Reduced virulence | 42.435 | 40.92 | 67.56 | 53.315 | 84.42 |
| GL_Gan1_GLEAN_10005361 | Cryptococcus neoformans | Reduced virulence | 4.43 | 24.94 | 21.145 | 22.15 | 25.495 |
| GL_Gan1_GLEAN_10003691 | Colletotrichum lagenarium | Reduced virulence | 1.555 | 31.62 | 21.695 | 10.265 | 37.335 |
| GL_Gan1_GLEAN_10003695 | Ustilago maydis | Loss of pathogenicity | 7.63 | 30.175 | 21.765 | 21.815 | 29.97 |
| GL_Gan1_GLEAN_10003710 | Saccharomyces cerevisiae | Reduced virulence | 26.365 | 13.285 | 17.41 | 19.34 | 16.995 |
| GL_Gan1_GLEAN_10003716 | Magnaporthe grisea | Reduced virulence | 26.52 | 28.24 | 27.665 | 28.13 | 25.49 |
| GL_Gan1_GLEAN_10003719 | Leptosphaeria maculans | Unaffected pathogenicity | 16.865 | 536.675 | 754.75 | 472.41 | 854.22 |
| GL_Gan1_GLEAN_10003723 | Ustilago maydis | Loss of pathogenicity | 168.525 | 58.935 | 59.605 | 58.125 | 71.735 |
| GL_Gan1_GLEAN_10003725 | Botrytis cinerea | Reduced virulence | 42.58 | 29.76 | 38.585 | 42.835 | 34.29 |
| GL_Gan1_GLEAN_10000062 | Magnaporthe grisea | Reduced virulence | 54.495 | 71.54 | 81.18 | 79.6 | 67.055 |
| GL_Gan1_GLEAN_10000044 | Magnaporthe grisea | Loss of pathogenicity | 13.515 | 15.735 | 19.965 | 21.62 | 19.615 |
| GL_Gan1_GLEAN_10007801 | Candida albicans | Reduced virulence | 43.15 | 38.085 | 44.37 | 45.785 | 44.715 |
| GL_Gan1_GLEAN_10007830 | Candida albicans | Reduced virulence | 157.95 | 106.59 | 70.79 | 56.815 | 122.015 |
| GL_Gan1_GLEAN_10007833 | Candida glabrata | Reduced virulence | 1.31 | 6.7 | 4.625 | 6.57 | 7.39 |
| GL_Gan1_GLEAN_10007837 | Candida albicans | Reduced virulence | 47.66 | 36.51 | 35.47 | 39.335 | 38.295 |
| GL_Gan1_GLEAN_10007838 | Candida albicans | Reduced virulence | 21.05 | 133.95 | 37.86 | 29.74 | 127.795 |
| GL_Gan1_GLEAN_10007844 | Candida albicans | Loss of pathogenicity | 31.065 | 20.925 | 22.475 | 23.05 | 27.145 |
| GL_Gan1_GLEAN_10007846 | Botrytis cinerea | Unaffected pathogenicity | 4.12 | 0.755 | 0.38 | 0.515 | 0.335 |
| GL_Gan1_GLEAN_10007849 | Cochliobolus heterostrophus | Reduced virulence | 1.165 | 2.81 | 4.97 | 4.245 | 4.065 |
| GL_Gan1_GLEAN_10007854 | Alternaria alternata | Loss of pathogenicity | 12.05 | 5.89 | 5.96 | 3.495 | 5.515 |
| GL_Gan1_GLEAN_10007860 | Candida albicans | Reduced virulence | 23.175 | 172.725 | 117.93 | 153.12 | 164.08 |
| GL_Gan1_GLEAN_10005399 | Cryptococcus neoformans | Reduced virulence | 285.625 | 278.805 | 292.82 | 249.53 | 256.885 |
| GL_Gan1_GLEAN_10007863 | Colletotrichum lagenarium | Loss of pathogenicity | 176.8 | 41.5 | 53.485 | 58.55 | 44.875 |
| GL_Gan1_GLEAN_10007864 | Fusarium graminearum | Unaffected pathogenicity | 3.115 | 31.41 | 11.05 | 12.07 | 30.645 |
| GL_Gan1_GLEAN_10007865 | Botrytis cinerea | Reduced virulence | 0 | 3.275 | 1.885 | 2.485 | 3.225 |
| GL_Gan1_GLEAN_10007868 | Botrytis cinerea | Unaffected pathogenicity | 129.62 | 19.55 | 14.185 | 23.745 | 12.745 |
| GL_Gan1_GLEAN_10007869 | Fusarium graminearum | Reduced virulence | 25.89 | 40.975 | 39.97 | 49.04 | 42.035 |
| GL_Gan1_GLEAN_10007877 | Candida albicans | Loss of pathogenicity | 20.265 | 11.365 | 6.99 | 7.775 | 12.095 |
| GL_Gan1_GLEAN_10007881 | Colletotrichum graminicola | Reduced virulence | 4.1 | 13.395 | 7.15 | 6.505 | 13.845 |
| GL_Gan1_GLEAN_10007888 | Magnaporthe grisea | Reduced virulence | 21.355 | 31.61 | 37.825 | 35.78 | 48.58 |
| GL_Gan1_GLEAN_10007894 | Stagonospora nodorum | Unaffected pathogenicity | 21.025 | 5.265 | 5.165 | 5.4 | 3.48 |
| GL_Gan1_GLEAN_10007896 | Magnaporthe grisea | Reduced virulence | 80.715 | 1229.9 | 716.935 | 830.35 | 1237.055 |
| GL_Gan1_GLEAN_10005607 | Cryptococcus neoformans | Reduced virulence | 3244.025 | 466.15 | 716.3 | 915.875 | 597.81 |
| GL_Gan1_GLEAN_10007907 | Candida albicans | Reduced virulence | 32.275 | 43.165 | 55.21 | 51.055 | 39.25 |
| GL_Gan1_GLEAN_10007921 | Colletotrichum gloeosporioides | Unaffected pathogenicity | 6.305 | 8.04 | 11.125 | 7.87 | 10.575 |
| GL_Gan1_GLEAN_10007924 | Candida albicans | Reduced virulence | 3.745 | 33.82 | 17.185 | 12.88 | 33.65 |
| GL_Gan1_GLEAN_10007928 | Candida albicans | Reduced virulence | 102.77 | 79.775 | 87.655 | 96.34 | 76.475 |
| GL_Gan1_GLEAN_10007935 | Magnaporthe grisea | Reduced virulence | 1.645 | 37.195 | 32.14 | 31.515 | 40.235 |
| GL_Gan1_GLEAN_10007937 | Candida albicans | Reduced virulence | 98.165 | 801 | 318.145 | 406.285 | 732.78 |
| GL_Gan1_GLEAN_10005625 | Cryptococcus neoformans | Reduced virulence | 95.32 | 116.89 | 123.41 | 130.54 | 151.08 |
| GL_Gan1_GLEAN_10005108 | Cryptococcus neoformans | Reduced virulence | 57.585 | 40.795 | 29.595 | 30.23 | 45.68 |
| GL_Gan1_GLEAN_10001734 | Cryptococcus neoformans | Reduced virulence | 289.145 | 145.175 | 152.1 | 134.445 | 105.08 |
| GL_Gan1_GLEAN_10007970 | Septoria lycopersici | Unaffected pathogenicity | 16.625 | 12.87 | 14.22 | 13.505 | 15.97 |
| GL_Gan1_GLEAN_10007972 | Fusarium graminearum | Reduced virulence | 89.67 | 55.005 | 61.58 | 59.445 | 53.045 |
| GL_Gan1_GLEAN_10007978 | Ustilago maydis | Reduced virulence | 28.08 | 36.455 | 26.19 | 25.295 | 36.32 |
| GL_Gan1_GLEAN_10007988 | Ustilago maydis | Unaffected pathogenicity | 9.52 | 115.675 | 55.96 | 62.65 | 96.85 |
| GL_Gan1_GLEAN_10007032 | Cryptococcus neoformans | Reduced virulence | 132.475 | 78.685 | 146.69 | 175.75 | 86.23 |
| GL_Gan1_GLEAN_10007996 | Aspergillus fumigatus | Reduced virulence | 135.56 | 227.59 | 329.935 | 309.945 | 263.85 |
| GL_Gan1_GLEAN_10007999 | Ustilago maydis | Reduced virulence | 40.375 | 18.465 | 26.68 | 23.5 | 21.85 |
| GL_Gan1_GLEAN_10007043 | Cryptococcus neoformans | Reduced virulence | 107.59 | 38.035 | 40.32 | 49.9 | 56.6 |
| GL_Gan1_GLEAN_10008005 | Botrytis cinerea | Unaffected pathogenicity | 0.65 | 0 | 0.065 | 0 | 0.21 |
| GL_Gan1_GLEAN_10008013 | Magnaporthe grisea | Loss of pathogenicity | 0 | 1.26 | 3.37 | 1.9 | 1.555 |
| GL_Gan1_GLEAN_10007130 | Cryptococcus neoformans | Reduced virulence | 0 | 1.19 | 3.695 | 1.6 | 1.51 |
| GL_Gan1_GLEAN_10001194 | Cochliobolus heterostrophus | Unaffected pathogenicity | 70.76 | 48.28 | 72.745 | 64.785 | 50.91 |
| GL_Gan1_GLEAN_10001202 | Candida albicans | Reduced virulence | 16.155 | 41.895 | 64.18 | 52.01 | 62.575 |
| GL_Gan1_GLEAN_10001210 | Colletotrichum lindemuthianum | Loss of pathogenicity | 11.6 | 15.085 | 7.68 | 6.105 | 15 |
| GL_Gan1_GLEAN_10001219 | Ustilago maydis | Reduced virulence | 3.635 | 10.925 | 11.075 | 9.82 | 13.385 |
| GL_Gan1_GLEAN_10001220 | Candida albicans | Reduced virulence | 5.81 | 1.415 | 3.77 | 2.42 | 2.45 |
| GL_Gan1_GLEAN_10001230 | Magnaporthe grisea | Reduced virulence | 102.35 | 58.3 | 72.94 | 77.465 | 87.425 |
| GL_Gan1_GLEAN_10001232 | Candida albicans | Reduced virulence | 0 | 1.53 | 2.52 | 2.085 | 1.905 |
| GL_Gan1_GLEAN_10001234 | Septoria lycopersici | Unaffected pathogenicity | 14.91 | 24.53 | 16.655 | 12.62 | 29.975 |
| GL_Gan1_GLEAN_10000049 | Magnaporthe grisea | Reduced virulence | 2.09 | 104.9 | 78.895 | 44.475 | 94.035 |
| GL_Gan1_GLEAN_10000087 | Stagonospora nodorum | Unaffected pathogenicity | 0 | 8.365 | 0.755 | 1.61 | 9.825 |
| GL_Gan1_GLEAN_10000497 | Aspergillus fumigatus | Reduced virulence | 0 | 0.865 | 0.71 | 1.61 | 2.705 |
| GL_Gan1_GLEAN_10000508 | Botrytis cinerea | Unaffected pathogenicity | 3.835 | 3.055 | 3.33 | 3.375 | 3.6 |
| GL_Gan1_GLEAN_10001853 | Pseudomonas syringae | Unaffected pathogenicity | 161.805 | 71.295 | 35.345 | 41.025 | 79.43 |
| GL_Gan1_GLEAN_10001854 | Colletotrichum lindemuthianum | Reduced virulence | 60.755 | 75.33 | 70.845 | 82.565 | 66.15 |
| GL_Gan1_GLEAN_10001864 | Cochliobolus heterostrophus | Reduced virulence | 24.805 | 5.985 | 9.37 | 7.31 | 8.42 |
| GL_Gan1_GLEAN_10001871 | Histoplasma capsulatum | Loss of pathogenicity | 379.545 | 812.785 | 1393.9 | 1458.98 | 867.99 |
| GL_Gan1_GLEAN_10001875 | Candida albicans | Reduced virulence | 53.52 | 57.485 | 66.785 | 75.97 | 57.615 |
| GL_Gan1_GLEAN_10001886 | Magnaporthe grisea | Reduced virulence | 76.385 | 32.325 | 22.235 | 23.85 | 30.725 |
| GL_Gan1_GLEAN_10002004 | Cryptococcus neoformans | Reduced virulence | 143.03 | 154.7 | 144.565 | 157.765 | 168.62 |
| GL_Gan1_GLEAN_10001080 | Magnaporthe grisea | Unaffected pathogenicity | 124.235 | 103.85 | 113.22 | 130.175 | 123.515 |
| GL_Gan1_GLEAN_10001083 | Candida albicans | Reduced virulence | 113.76 | 263.235 | 272.22 | 307.855 | 241.95 |
| GL_Gan1_GLEAN_10001085 | Botrytis cinerea | Reduced virulence | 0 | 0 | 1.235 | 0.34 | 2.065 |
| GL_Gan1_GLEAN_10001086 | Cercospora nicotianae | Reduced virulence | 0 | 7.855 | 2.7 | 2.145 | 2.195 |
| GL_Gan1_GLEAN_10001090 | Candida albicans | Reduced virulence | 62.065 | 37.365 | 45.775 | 54.695 | 46.335 |
| GL_Gan1_GLEAN_10001959 | Fusarium graminearum | Reduced virulence | 178.47 | 250.8 | 206.995 | 206.51 | 264.985 |
| GL_Gan1_GLEAN_10001966 | Candida glabrata | Increased virulence (Hypervirulence) | 84.465 | 54.88 | 43.295 | 40.225 | 85.45 |
| GL_Gan1_GLEAN_10005239 | Cryptococcus neoformans | Reduced virulence | 49.55 | 18.415 | 20.68 | 19.04 | 21.415 |
| GL_Gan1_GLEAN_10001973 | Magnaporthe grisea | Unaffected pathogenicity | 28.86 | 15.265 | 16.135 | 20.425 | 19.14 |
| GL_Gan1_GLEAN_10005285 | Cryptococcus neoformans | Reduced virulence | 50.935 | 81.945 | 46.475 | 52.535 | 77.685 |
| GL_Gan1_GLEAN_10001980 | Candida albicans | Reduced virulence | 5.25 | 39.865 | 21.3 | 38.15 | 33.37 |
| GL_Gan1_GLEAN_10007163 | Cryptococcus neoformans | Reduced virulence | 49.065 | 32.42 | 40.06 | 40.74 | 45.16 |
| GL_Gan1_GLEAN_10007164 | Cryptococcus neoformans | Reduced virulence | 191.24 | 103.36 | 105.95 | 107.68 | 100.57 |
| GL_Gan1_GLEAN_10007191 | Magnaporthe grisea | Loss of pathogenicity | 6.795 | 3.195 | 3.515 | 2.62 | 2.23 |
| GL_Gan1_GLEAN_10007199 | Magnaporthe grisea | Reduced virulence | 3.035 | 4.92 | 7.625 | 4.365 | 7.28 |
| GL_Gan1_GLEAN_10000978 | Cryptococcus neoformans | Reduced virulence | 149.195 | 126.69 | 215.83 | 263.02 | 121.935 |
| GL_Gan1_GLEAN_10007203 | Nectria haematococca (related: Fusarium solani) | Reduced virulence | 9.775 | 57.99 | 131.69 | 64.465 | 93.1 |
| GL_Gan1_GLEAN_10007213 | Candida albicans | Reduced virulence | 61.525 | 177.885 | 209.645 | 156.32 | 227.53 |
| GL_Gan1_GLEAN_10004570 | Cryptococcus neoformans | Reduced virulence | 85.93 | 59.41 | 59.275 | 61.655 | 57.52 |
| GL_Gan1_GLEAN_10004585 | Cryptococcus neoformans | Reduced virulence | 91.14 | 30.17 | 33.24 | 35.6 | 30.26 |
| GL_Gan1_GLEAN_10004609 | Cryptococcus neoformans | Reduced virulence | 4.66 | 10.265 | 8.555 | 8.745 | 5.745 |
| GL_Gan1_GLEAN_10007229 | Fusarium oxysporum | Unaffected pathogenicity | 13.99 | 2.68 | 7.365 | 8.305 | 3.68 |
| GL_Gan1_GLEAN_10000166 | Cryptococcus neoformans | Reduced virulence | 40.495 | 99.585 | 100.63 | 93.875 | 66.83 |
| GL_Gan1_GLEAN_10007235 | Verticillium fungicola | Reduced virulence | 20.255 | 11.925 | 33.34 | 18.255 | 23.075 |
| GL_Gan1_GLEAN_10007245 | Botrytis cinerea | Unaffected pathogenicity | 0 | 0.625 | 0.245 | 0.15 | 0.895 |
| GL_Gan1_GLEAN_10007263 | Botrytis cinerea | Reduced virulence | 9.595 | 4.83 | 3.4 | 4.035 | 4.04 |
| GL_Gan1_GLEAN_10007266 | Fusarium oxysporum | Unaffected pathogenicity | 1.475 | 2.76 | 6.43 | 5.47 | 3.045 |
| GL_Gan1_GLEAN_10007267 | Cercospora nicotianae | Reduced virulence | 0.85 | 3.71 | 12.345 | 6.995 | 4.515 |
| GL_Gan1_GLEAN_10002241 | Cryptococcus neoformans | Loss of pathogenicity | 0 | 4.295 | 4.88 | 3.925 | 3.1 |
| GL_Gan1_GLEAN_10007295 | Fusarium oxysporum | Loss of pathogenicity | 26.44 | 17.48 | 21.495 | 19.72 | 21.14 |
| GL_Gan1_GLEAN_10007298 | Ustilago maydis | Unaffected pathogenicity | 5.41 | 10.61 | 4.69 | 3.185 | 10.475 |
| GL_Gan1_GLEAN_10007302 | Candida albicans | Reduced virulence | 2.545 | 6.98 | 7.84 | 7.415 | 6.745 |
| GL_Gan1_GLEAN_10007306 | Aspergillus fumigatus | Reduced virulence | 0 | 9.385 | 4.37 | 6.56 | 6.44 |
| GL_Gan1_GLEAN_10007323 | Magnaporthe grisea | Unaffected pathogenicity | 40.94 | 76.185 | 88.225 | 85.515 | 79.465 |
| GL_Gan1_GLEAN_10007325 | Ustilago maydis | Reduced virulence | 3.87 | 6.055 | 6.015 | 4.73 | 8.25 |
| GL_Gan1_GLEAN_10007331 | Cryptococcus neoformans | Loss of pathogenicity | 8.685 | 32.59 | 21.635 | 19.13 | 32.78 |
| GL_Gan1_GLEAN_10007337 | Botrytis cinerea | Unaffected pathogenicity | 0 | 0.745 | 4.265 | 2.32 | 3.32 |
| GL_Gan1_GLEAN_10007342 | Botrytis cinerea | Unaffected pathogenicity | 0 | 0.56 | 0.185 | 0.225 | 0.43 |
| GL_Gan1_GLEAN_10002595 | Candida albicans | Loss of pathogenicity | 26.635 | 38.57 | 41.415 | 42.6 | 48.795 |
| GL_Gan1_GLEAN_10002596 | Candida albicans | Reduced virulence | 23.64 | 18.33 | 14.715 | 12.895 | 20.03 |
| GL_Gan1_GLEAN_10002598 | Nectria haematococca (related: Fusarium solani) | Reduced virulence | 0 | 48.685 | 1.48 | 1.39 | 20.695 |
| GL_Gan1_GLEAN_10007628 | Cryptococcus neoformans | Reduced virulence | 110.855 | 62.74 | 82.845 | 54.775 | 94.37 |
| GL_Gan1_GLEAN_10007701 | Cryptococcus neoformans | Reduced virulence | 76.805 | 128.16 | 115.985 | 101.63 | 126.93 |
| GL_Gan1_GLEAN_10002614 | Aspergillus fumigatus | Reduced virulence | 330.74 | 498.455 | 949.19 | 833.205 | 703.37 |
| GL_Gan1_GLEAN_10002619 | Alternaria alternata | Loss of pathogenicity | 20.84 | 21.45 | 23.135 | 22.745 | 26.765 |
| GL_Gan1_GLEAN_10002624 | Ustilago maydis | Loss of pathogenicity | 5.77 | 12.89 | 4.24 | 4.41 | 16.27 |
| GL_Gan1_GLEAN_10002638 | Magnaporthe grisea | Reduced virulence | 0 | 3.225 | 0.89 | 0.78 | 1.935 |
| GL_Gan1_GLEAN_10002639 | Colletotrichum lagenarium | Loss of pathogenicity | 69.88 | 30.15 | 47.035 | 56.075 | 44.92 |
| GL_Gan1_GLEAN_10002645 | Candida albicans | Reduced virulence | 14.045 | 23.83 | 29.47 | 30.355 | 25.645 |
| GL_Gan1_GLEAN_10002646 | Colletotrichum lindemuthianum | Loss of pathogenicity | 24.44 | 18.34 | 20.025 | 17.95 | 19.975 |
| GL_Gan1_GLEAN_10002648 | Ustilago maydis | Reduced virulence | 24.495 | 51.98 | 42.79 | 40.685 | 69.275 |
| GL_Gan1_GLEAN_10001588 | Magnaporthe grisea | Unaffected pathogenicity | 17.095 | 37.36 | 28.495 | 25.505 | 36.6 |
| GL_Gan1_GLEAN_10007750 | Cryptococcus neoformans | Increased virulence (Hypervirulence) | 33.815 | 38.325 | 22.64 | 23.57 | 36.805 |
| GL_Gan1_GLEAN_10001604 | Candida albicans | Reduced virulence | 103.765 | 47.025 | 37.5 | 37.51 | 51.795 |
| GL_Gan1_GLEAN_10006054 | Cryptococcus neoformans | Unaffected pathogenicity | 27.92 | 27.605 | 49.385 | 24.32 | 43.515 |
| GL_Gan1_GLEAN_10001607 | Candida albicans | Reduced virulence | 27.92 | 4.01 | 5.24 | 5.335 | 3.64 |
| GL_Gan1_GLEAN_10001609 | Candida albicans | Reduced virulence | 9.675 | 11.555 | 15.255 | 16.18 | 14.785 |
| GL_Gan1_GLEAN_10001613 | Fusarium oxysporum | Loss of pathogenicity | 41.81 | 23.945 | 22.69 | 23.7 | 30.5 |
| GL_Gan1_GLEAN_10001615 | Mycosphaerella graminicola | Unaffected pathogenicity | 1.74 | 1.4 | 4.775 | 2.425 | 2.605 |
| GL_Gan1_GLEAN_10001618 | Mycosphaerella graminicola | Resistant to chemical | 0 | 0.18 | 0.905 | 0.57 | 0.225 |
| GL_Gan1_GLEAN_10000728 | Botrytis cinerea | Reduced virulence | 11.28 | 27.895 | 6.105 | 6.595 | 39.545 |
| GL_Gan1_GLEAN_10000735 | Fusarium graminearum | Reduced virulence | 19.78 | 31.99 | 29.765 | 33.74 | 38.38 |
| GL_Gan1_GLEAN_10000737 | Magnaporthe grisea | Reduced virulence | 0 | 0.52 | 0.66 | 0.725 | 0.375 |
| GL_Gan1_GLEAN_10000747 | Magnaporthe grisea | Reduced virulence | 54.105 | 48.9 | 64.23 | 56.355 | 46.62 |
| GL_Gan1_GLEAN_10000750 | Candida albicans | Reduced virulence | 125.225 | 33.75 | 50.575 | 52.53 | 49.37 |
| GL_Gan1_GLEAN_10000095 | Ustilago maydis | Unaffected pathogenicity | 2.73 | 124.015 | 140.31 | 47.315 | 84.585 |
| GL_Gan1_GLEAN_10000099 | Stagonospora nodorum | Unaffected pathogenicity | 2.555 | 11.675 | 13.985 | 5.94 | 4.745 |
| GL_Gan1_GLEAN_10004667 | Colletotrichum lagenarium | Reduced virulence | 3.425 | 17.705 | 17.01 | 14.045 | 10.495 |
| GL_Gan1_GLEAN_10004672 | Candida albicans | Loss of pathogenicity | 16.605 | 32.07 | 19.255 | 20.14 | 32.255 |
| GL_Gan1_GLEAN_10004683 | Colletotrichum lindemuthianum | Reduced virulence | 68.54 | 147.235 | 108.69 | 109.03 | 136.245 |
| GL_Gan1_GLEAN_10004689 | Fusarium oxysporum | Unaffected pathogenicity | 57.89 | 8.975 | 9.88 | 7.49 | 8.55 |
| GL_Gan1_GLEAN_10004695 | Candida albicans | Reduced virulence | 5.345 | 14.075 | 11.965 | 15.45 | 14.005 |
| GL_Gan1_GLEAN_10004709 | Candida albicans | Reduced virulence | 8.845 | 30.745 | 31.585 | 21.535 | 36.675 |
| GL_Gan1_GLEAN_10004713 | Fusarium graminearum | Unaffected pathogenicity | 17.99 | 57.6 | 113.02 | 110.995 | 78.28 |
| GL_Gan1_GLEAN_10004714 | Magnaporthe grisea | Reduced virulence | 50.11 | 10.315 | 15.82 | 16.525 | 9.29 |
| GL_Gan1_GLEAN_10000916 | Cryptococcus neoformans | Loss of pathogenicity | 74.42 | 146.07 | 150.885 | 172.375 | 149.5 |
| GL_Gan1_GLEAN_10004720 | Fusarium oxysporum | Reduced virulence | 79.355 | 45.285 | 52.155 | 58.315 | 54.03 |
| GL_Gan1_GLEAN_10004723 | Candida tropicalis | Reduced virulence | 1.045 | 7.575 | 3.955 | 6.975 | 7.14 |
| GL_Gan1_GLEAN_10004731 | Candida albicans | Reduced virulence | 37.98 | 84.875 | 98.38 | 88.635 | 110.33 |
| GL_Gan1_GLEAN_10004733 | Aspergillus fumigatus | Reduced virulence | 5.235 | 5.395 | 7.915 | 6.255 | 6.57 |
| GL_Gan1_GLEAN_10004734 | Fusarium oxysporum | Loss of pathogenicity | 16.08 | 2.965 | 6.87 | 6.725 | 6.72 |
| GL_Gan1_GLEAN_10004752 | Candida albicans | Reduced virulence | 6.04 | 11.875 | 21.255 | 19.475 | 16.05 |
| GL_Gan1_GLEAN_10004755 | Magnaporthe grisea | Reduced virulence | 33.185 | 12.12 | 10.3 | 10.845 | 7.795 |
| GL_Gan1_GLEAN_10004762 | Cryptococcus neoformans | Reduced virulence | 69.355 | 33.015 | 85.985 | 92.195 | 66.145 |
| GL_Gan1_GLEAN_10004767 | Ustilago maydis | Reduced virulence | 8.5 | 18.735 | 9.34 | 10.43 | 24.89 |
| GL_Gan1_GLEAN_10004775 | Magnaporthe grisea | Loss of pathogenicity | 28.08 | 39.69 | 51.65 | 63.16 | 75.23 |
| GL_Gan1_GLEAN_10002552 | Cryptococcus neoformans | Loss of pathogenicity | 287.425 | 250.295 | 238.395 | 215.12 | 187.255 |
| GL_Gan1_GLEAN_10004880 | Candida albicans | Reduced virulence | 181.38 | 192.68 | 188.25 | 181.57 | 205.8 |
| GL_Gan1_GLEAN_10004883 | Alternaria alternata | Loss of pathogenicity | 0 | 3.69 | 1.96 | 1.605 | 1.89 |
| GL_Gan1_GLEAN_10002979 | Cryptococcus neoformans | Reduced virulence | 1878.985 | 309.715 | 205.875 | 174.015 | 347.425 |
| GL_Gan1_GLEAN_10004914 | Stagonospora nodorum | Unaffected pathogenicity | 46.35 | 8.225 | 8.43 | 8.49 | 9.205 |
| GL_Gan1_GLEAN_10004918 | Magnaporthe grisea | Reduced virulence | 48.305 | 20.08 | 30.145 | 39.53 | 23.485 |
| GL_Gan1_GLEAN_10004920 | Magnaporthe grisea | Loss of pathogenicity | 0 | 5.575 | 2.99 | 3.38 | 5.86 |
| GL_Gan1_GLEAN_10004921 | Colletotrichum gloeosporioides | Unaffected pathogenicity | 2.69 | 10.825 | 7.64 | 8.555 | 11.73 |
| GL_Gan1_GLEAN_10004930 | Cryptococcus neoformans | Reduced virulence | 3.575 | 17.545 | 20.385 | 23.885 | 19.56 |
| GL_Gan1_GLEAN_10004934 | Cryptococcus neoformans | Loss of pathogenicity | 1.595 | 3.885 | 3.325 | 2.375 | 4.91 |
| GL_Gan1_GLEAN_10004944 | Magnaporthe grisea | Loss of pathogenicity | 47.285 | 28.44 | 33.85 | 23.925 | 43.26 |
| GL_Gan1_GLEAN_10004945 | Ustilago maydis | Reduced virulence | 136.41 | 83.34 | 157.54 | 171.07 | 99.28 |
| GL_Gan1_GLEAN_10004946 | Colletotrichum lindemuthianum | Reduced virulence | 10.225 | 34.67 | 36.845 | 39.005 | 37.09 |
| GL_Gan1_GLEAN_10004950 | Botrytis cinerea | Unaffected pathogenicity | 12.5 | 40.165 | 24.99 | 36.71 | 68.245 |
| GL_Gan1_GLEAN_10004953 | Candida albicans | Reduced virulence | 13.33 | 5.22 | 5.845 | 4.985 | 2.835 |
| GL_Gan1_GLEAN_10004959 | Fusarium oxysporum | Unaffected pathogenicity | 3.155 | 13.655 | 6.955 | 7.49 | 21.455 |
| GL_Gan1_GLEAN_10004960 | Pseudomonas syringae | Unaffected pathogenicity | 19.955 | 126.985 | 32.625 | 69.245 | 172.845 |
| GL_Gan1_GLEAN_10004961 | Mycosphaerella graminicola | Reduced virulence | 11.44 | 25.865 | 27.07 | 27.855 | 40.175 |
| GL_Gan1_GLEAN_10004963 | Botrytis cinerea | Unaffected pathogenicity | 8.325 | 42.475 | 37.72 | 51.68 | 41.945 |
| GL_Gan1_GLEAN_10004964 | Ustilago maydis | Loss of pathogenicity | 80.425 | 39.445 | 36.905 | 35.205 | 58.75 |
| GL_Gan1_GLEAN_10005496 | Cryptococcus neoformans | Reduced virulence | 35.58 | 22.205 | 23.89 | 25.705 | 19.13 |
| GL_Gan1_GLEAN_10001416 | Aspergillus fumigatus | Reduced virulence | 89.345 | 30.39 | 34.955 | 33.29 | 43.245 |
| GL_Gan1_GLEAN_10001419 | Magnaporthe grisea | Loss of pathogenicity | 75.38 | 28.43 | 22.42 | 22.09 | 25.91 |
| GL_Gan1_GLEAN_10001423 | Gibberella moniliformis | Unaffected pathogenicity | 1.535 | 2.945 | 1.465 | 1.095 | 2.06 |
| GL_Gan1_GLEAN_10001437 | Saccharomyces cerevisiae | Reduced virulence | 213.395 | 667.635 | 518.98 | 549.635 | 567.15 |
| GL_Gan1_GLEAN_10001441 | Candida albicans | Loss of pathogenicity | 40.365 | 125.83 | 88.805 | 80.82 | 134.015 |
| GL_Gan1_GLEAN_10001451 | Candida albicans | Reduced virulence | 0 | 24.87 | 12.77 | 12.325 | 28.425 |
| GL_Gan1_GLEAN_10001458 | Colletotrichum lindemuthianum | Reduced virulence | 132.79 | 343.76 | 324.99 | 366.53 | 310.04 |
| GL_Gan1_GLEAN_10002308 | Candida albicans | Reduced virulence | 38.375 | 190.57 | 257.125 | 229.725 | 172.865 |
| GL_Gan1_GLEAN_10002311 | Ustilago maydis | Reduced virulence | 6.08 | 12.545 | 8.715 | 6.73 | 10.275 |
| GL_Gan1_GLEAN_10002315 | Magnaporthe grisea | Reduced virulence | 10.61 | 4.475 | 8.42 | 4.285 | 9.94 |
| GL_Gan1_GLEAN_10002321 | Fusarium sporotrichioides | Unaffected pathogenicity | 0 | 0.17 | 0.58 | 0.565 | 0.445 |
| GL_Gan1_GLEAN_10002324 | Candida albicans | Reduced virulence | 120.67 | 86.005 | 64.925 | 42.63 | 97.98 |
| GL_Gan1_GLEAN_10002325 | Candida albicans | Reduced virulence | 442.51 | 80.065 | 65.8 | 55.895 | 83.96 |
| GL_Gan1_GLEAN_10002326 | Botrytis cinerea | Unaffected pathogenicity | 7.99 | 39.01 | 14.55 | 14.015 | 35.235 |
| GL_Gan1_GLEAN_10002328 | Botrytis cinerea | Reduced virulence | 31.64 | 15.24 | 27.7 | 27.495 | 28.605 |
| GL_Gan1_GLEAN_10002332 | Claviceps purpurea | Reduced virulence | 1.645 | 8.02 | 2.18 | 2.24 | 6.845 |
| GL_Gan1_GLEAN_10005714 | Cryptococcus neoformans | Reduced virulence | 4.94 | 6.125 | 4.365 | 4.155 | 4.67 |
| GL_Gan1_GLEAN_10002336 | Colletotrichum lindemuthianum | Loss of pathogenicity | 23.015 | 3.36 | 4.69 | 4.275 | 5.27 |
| GL_Gan1_GLEAN_10001291 | Candida albicans | Unaffected pathogenicity | 476.795 | 553.225 | 513.665 | 449.425 | 480.9 |
| GL_Gan1_GLEAN_10001295 | Botrytis cinerea | Unaffected pathogenicity | 8.605 | 2.165 | 11.97 | 4.91 | 5.18 |
| GL_Gan1_GLEAN_10001296 | Botrytis cinerea | Reduced virulence | 15.655 | 9.07 | 5.835 | 6.76 | 12.885 |
| GL_Gan1_GLEAN_10001300 | Magnaporthe grisea | Reduced virulence | 13.245 | 7.35 | 8.865 | 9.375 | 7.47 |
| GL_Gan1_GLEAN_10001302 | Colletotrichum lindemuthianum | Loss of pathogenicity | 13.675 | 28.03 | 26.64 | 26.65 | 30.965 |
| GL_Gan1_GLEAN_10001303 | Colletotrichum lindemuthianum | Loss of pathogenicity | 31.73 | 19.07 | 19.87 | 21.35 | 15.555 |
| GL_Gan1_GLEAN_10001308 | Saccharomyces cerevisiae | Reduced virulence | 58.91 | 59.52 | 59.635 | 72.68 | 69.76 |
| GL_Gan1_GLEAN_10001310 | Candida albicans | Reduced virulence | 108.59 | 46.85 | 75.885 | 75.41 | 56.63 |
| GL_Gan1_GLEAN_10005778 | Cryptococcus neoformans | Unaffected pathogenicity | 16.745 | 160.35 | 54.31 | 44 | 107.955 |
| GL_Gan1_GLEAN_10002443 | Candida albicans | Reduced virulence | 15.355 | 68.855 | 20.51 | 33.295 | 95 |
| GL_Gan1_GLEAN_10002456 | Candida albicans | Reduced virulence | 0 | 0.285 | 0.855 | 1.01 | 0.66 |
| GL_Gan1_GLEAN_10004027 | Ustilago maydis | Reduced virulence | 202.84 | 195.05 | 212.975 | 264.725 | 217.795 |
| GL_Gan1_GLEAN_10000773 | Cryptococcus neoformans | Loss of pathogenicity | 24.38 | 17.65 | 20.12 | 17.705 | 20.05 |
| GL_Gan1_GLEAN_10004031 | Fusarium graminearum | Unaffected pathogenicity | 1.07 | 4.225 | 3.375 | 3.185 | 3.995 |
| GL_Gan1_GLEAN_10004032 | Magnaporthe grisea | Reduced virulence | 14.3 | 0 | 0.37 | 0.345 | 0.25 |
| GL_Gan1_GLEAN_10004035 | Botrytis cinerea | Reduced virulence | 6.585 | 0 | 0.235 | 0.205 | 0 |
| GL_Gan1_GLEAN_10004036 | Magnaporthe grisea | Reduced virulence | 19.995 | 3.965 | 7.39 | 8.29 | 2.51 |
| GL_Gan1_GLEAN_10004037 | Fusarium graminearum | Unaffected pathogenicity | 17.735 | 1.59 | 1.265 | 2.075 | 1.845 |
| GL_Gan1_GLEAN_10004039 | Colletotrichum lagenarium | Reduced virulence | 364.255 | 148.27 | 63 | 82.4 | 107.455 |
| GL_Gan1_GLEAN_10004055 | Ustilago maydis | Unaffected pathogenicity | 172.25 | 52.34 | 27.48 | 28.05 | 44.395 |
| GL_Gan1_GLEAN_10004068 | Candida albicans | Reduced virulence | 0.58 | 4.715 | 8.025 | 9.79 | 4.61 |
| GL_Gan1_GLEAN_10004072 | Candida albicans | Reduced virulence | 11.25 | 50.48 | 22.1 | 13.425 | 48.905 |
| GL_Gan1_GLEAN_10004073 | Stagonospora nodorum | Unaffected pathogenicity | 5.08 | 13.44 | 8.22 | 4.81 | 21.805 |
| GL_Gan1_GLEAN_10004081 | Ustilago maydis | Unaffected pathogenicity | 8.075 | 1.595 | 0.935 | 0.525 | 1.44 |
| GL_Gan1_GLEAN_10004084 | Salmonella enterica |  | 162.79 | 19.645 | 18.84 | 25.07 | 18.58 |
| GL_Gan1_GLEAN_10004097 | Fusarium oxysporum | Loss of pathogenicity | 0.945 | 0 | 0.14 | 0.335 | 0.485 |
| GL_Gan1_GLEAN_10004098 | Candida albicans | Reduced virulence | 0 | 0.28 | 0 | 0.165 | 0.155 |
| GL_Gan1_GLEAN_10004104 | Botrytis cinerea | Unaffected pathogenicity | 0 | 0 | 0.04 | 0.105 | 0.095 |
| GL_Gan1_GLEAN_10004109 | Botrytis cinerea | Reduced virulence | 1.115 | 26.05 | 15.04 | 16.105 | 23.05 |
| GL_Gan1_GLEAN_10004110 | Fusarium graminearum | Unaffected pathogenicity | 11.805 | 103.325 | 8.95 | 11.455 | 85.695 |
| GL_Gan1_GLEAN_10004112 | Magnaporthe grisea | Reduced virulence | 0 | 0.205 | 0.135 | 0.37 | 0.115 |
| GL_Gan1_GLEAN_10004117 | Candida albicans | Reduced virulence | 31.305 | 32.4 | 30.285 | 30.225 | 31.11 |
| GL_Gan1_GLEAN_10004119 | Candida albicans | Reduced virulence | 1.145 | 1.755 | 1.415 | 1.73 | 1.68 |
| GL_Gan1_GLEAN_10003577 | Cryptococcus neoformans | Reduced virulence | 122.5 | 50.1 | 63.51 | 53.505 | 79.795 |
| GL_Gan1_GLEAN_10002745 | Magnaporthe grisea | Reduced virulence | 585.16 | 62.32 | 89.765 | 91.755 | 71.17 |
| GL_Gan1_GLEAN_10002749 | Candida albicans | Loss of pathogenicity | 5.275 | 33.7 | 20.285 | 24.235 | 40.62 |
| GL_Gan1_GLEAN_10002754 | Aspergillus fumigatus | Increased virulence (Hypervirulence) | 14.7 | 94.45 | 81.265 | 95.39 | 126.2 |
| GL_Gan1_GLEAN_10002759 | Colletotrichum lindemuthianum | Loss of pathogenicity | 2.45 | 4.71 | 6.97 | 4.89 | 3.19 |
| GL_Gan1_GLEAN_10002773 | Ustilago maydis | Reduced virulence | 32.235 | 9.395 | 11.875 | 8.31 | 8.4 |
| GL_Gan1_GLEAN_10002782 | Botrytis cinerea | Reduced virulence | 0 | 0.685 | 0 | 0.985 | 0.56 |
| GL_Gan1_GLEAN_10002785 | Botrytis cinerea | Unaffected pathogenicity | 25.62 | 2.8 | 10 | 4.765 | 4.085 |
| GL_Gan1_GLEAN_10002787 | Botrytis cinerea | Unaffected pathogenicity | 9.835 | 3.605 | 1.48 | 3.525 | 2.46 |
| GL_Gan1_GLEAN_10002788 | Cryptococcus neoformans | Reduced virulence | 48.99 | 7.6 | 6.445 | 7.915 | 7.53 |
| GL_Gan1_GLEAN_10002789 | Botrytis cinerea | Reduced virulence | 3.78 | 2.345 | 1.21 | 3.97 | 3.49 |
| GL_Gan1_GLEAN_10002790 | Fusarium oxysporum | Loss of pathogenicity | 29.095 | 14.465 | 17.005 | 19.19 | 19.23 |
| GL_Gan1_GLEAN_10002792 | Cercospora nicotianae | Reduced virulence | 203.72 | 9.655 | 9.18 | 15.99 | 13.895 |
| GL_Gan1_GLEAN_10002794 | Stagonospora nodorum | Unaffected pathogenicity | 4.87 | 34.505 | 16.595 | 6.595 | 30.445 |
| GL_Gan1_GLEAN_10000068 | Cercospora zeae-maydis | Reduced virulence | 40.06 | 36.66 | 56.62 | 51.23 | 23.28 |
| GL_Gan1_GLEAN_10001470 | Colletotrichum graminicola | Reduced virulence | 39.62 | 52.95 | 39.01 | 49.98 | 57.33 |
| GL_Gan1_GLEAN_10001472 | Candida albicans | Reduced virulence | 20.58 | 16.03 | 17.89 | 17.355 | 19.845 |
| GL_Gan1_GLEAN_10001474 | Candida albicans | Loss of pathogenicity | 21.87 | 29.805 | 36.18 | 35.205 | 35.225 |
| GL_Gan1_GLEAN_10001478 | Aspergillus fumigatus | Reduced virulence | 300.73 | 190.31 | 214.19 | 247.455 | 113.635 |
| GL_Gan1_GLEAN_10001485 | Botrytis cinerea | Reduced virulence | 7.035 | 6.81 | 8.86 | 5.165 | 6.52 |
| GL_Gan1_GLEAN_10001486 | Candida albicans | Reduced virulence | 0 | 9.43 | 15.125 | 6.53 | 8.295 |
| GL_Gan1_GLEAN_10001488 | Fusarium oxysporum | Unaffected pathogenicity | 22.39 | 10.615 | 17.37 | 12.725 | 12.05 |
| GL_Gan1_GLEAN_10001489 | Colletotrichum lagenarium | Reduced virulence | 23.63 | 35.81 | 39.29 | 33.39 | 33.28 |
| GL_Gan1_GLEAN_10001498 | Magnaporthe grisea | Reduced virulence | 0 | 5.875 | 7.74 | 4.27 | 4.71 |
| GL_Gan1_GLEAN_10001504 | Trichoderma virens | Reduced virulence | 0 | 1.92 | 1.015 | 0.805 | 2.43 |
| GL_Gan1_GLEAN_10003004 | Candida albicans | Reduced virulence | 0.28 | 7.745 | 3.825 | 5.265 | 10.78 |
| GL_Gan1_GLEAN_10003006 | Cochliobolus heterostrophus | Reduced virulence | 108.96 | 158.64 | 209.625 | 148.95 | 161.405 |
| GL_Gan1_GLEAN_10003014 | Ustilago maydis | Reduced virulence | 83.845 | 48.8 | 61.875 | 57.295 | 73.37 |
| GL_Gan1_GLEAN_10003030 | Mycosphaerella graminicola | Reduced virulence | 0 | 15.3 | 12.755 | 12.535 | 15.19 |
| GL_Gan1_GLEAN_10003032 | Ustilago maydis | Unaffected pathogenicity | 0 | 3.115 | 0.76 | 1.15 | 3.655 |
| GL_Gan1_GLEAN_10003033 | Fusarium graminearum | Reduced virulence | 0 | 0 | 0 | 0 | 0.19 |
| GL_Gan1_GLEAN_10003050 | Ustilago maydis | Reduced virulence | 214.02 | 69.675 | 66.565 | 58.885 | 81.875 |
| GL_Gan1_GLEAN_10003052 | Ustilago maydis | Reduced virulence | 21.695 | 17.485 | 13.595 | 14.525 | 18.64 |
| GL_Gan1_GLEAN_10003059 | Cryptococcus neoformans | Reduced virulence | 45.33 | 51.11 | 57.225 | 62.615 | 56.585 |
| GL_Gan1_GLEAN_10003066 | Ustilago maydis | Loss of pathogenicity | 58.905 | 41.71 | 56.595 | 69.025 | 60.485 |
| GL_Gan1_GLEAN_10003070 | Candida albicans | Reduced virulence | 34.455 | 31.61 | 30.21 | 38.25 | 29.26 |
| GL_Gan1_GLEAN_10003071 | Fusarium oxysporum | Unaffected pathogenicity | 21.5 | 9.9 | 10.61 | 10.74 | 11.09 |
| GL_Gan1_GLEAN_10002183 | Magnaporthe grisea | Reduced virulence | 9.255 | 4.935 | 4.73 | 6.925 | 3.595 |
| GL_Gan1_GLEAN_10002184 | Botrytis cinerea | Unaffected pathogenicity | 17.97 | 17.665 | 18.735 | 11.885 | 14.68 |
| GL_Gan1_GLEAN_10002185 | Alternaria alternata | Loss of pathogenicity | 0.575 | 18.9 | 4.02 | 1.475 | 14.055 |
| GL_Gan1_GLEAN_10002186 | Botrytis cinerea | Reduced virulence | 7.935 | 23.835 | 28.275 | 9.915 | 13.75 |
| GL_Gan1_GLEAN_10002190 | Fusarium oxysporum | Loss of pathogenicity | 18.805 | 21.78 | 43.555 | 26.73 | 28.23 |
| GL_Gan1_GLEAN_10002193 | Candida albicans | Reduced virulence | 0 | 1.02 | 1.965 | 1.275 | 2.17 |
| GL_Gan1_GLEAN_10002201 | Candida albicans | Loss of pathogenicity | 2.1 | 6.25 | 6.98 | 6.515 | 6.67 |
| GL_Gan1_GLEAN_10006638 | Cryptococcus neoformans | Reduced virulence | 111.13 | 105.055 | 150.15 | 173.245 | 72.325 |
| GL_Gan1_GLEAN_10002206 | Magnaporthe grisea | Loss of pathogenicity | 9.865 | 3.93 | 3.515 | 2.495 | 3.705 |
| GL_Gan1_GLEAN_10002207 | Fusarium oxysporum | Unaffected pathogenicity | 25.805 | 5.035 | 4.52 | 3.84 | 6.695 |
| GL_Gan1_GLEAN_10002218 | Candida albicans | Reduced virulence | 387.21 | 437.67 | 268.41 | 199.11 | 446.765 |
| GL_Gan1_GLEAN_10002223 | Fusarium oxysporum | Unaffected pathogenicity | 235.95 | 28.285 | 12.715 | 15.73 | 30.88 |
| GL_Gan1_GLEAN_10002226 | Stagonospora nodorum | Unaffected pathogenicity | 0 | 43.84 | 10.225 | 14.085 | 44.96 |
| GL_Gan1_GLEAN_10002229 | Colletotrichum lindemuthianum | Reduced virulence | 0 | 14.675 | 19.73 | 15.98 | 13.64 |
| GL_Gan1_GLEAN_10004330 | Cochliobolus carbonum | Reduced virulence | 211.085 | 31.835 | 34.175 | 39.445 | 26.48 |
| GL_Gan1_GLEAN_10004331 | Botrytis cinerea | Unaffected pathogenicity | 19.24 | 25.43 | 4.62 | 4.575 | 25.14 |
| GL_Gan1_GLEAN_10004334 | Magnaporthe grisea | Reduced virulence | 2.96 | 5.34 | 2.935 | 4.05 | 4.59 |
| GL_Gan1_GLEAN_10004335 | Botrytis cinerea | Reduced virulence | 10.515 | 0.735 | 3.49 | 2.045 | 2.26 |
| GL_Gan1_GLEAN_10004339 | Magnaporthe grisea | Reduced virulence | 3217.235 | 78.92 | 53.795 | 18.825 | 90.13 |
| GL_Gan1_GLEAN_10004351 | Colletotrichum lagenarium | Reduced virulence | 6.295 | 53.525 | 54.135 | 39.305 | 52.365 |
| GL_Gan1_GLEAN_10004354 | Fusarium graminearum | Reduced virulence | 36.525 | 103.255 | 119.065 | 114.715 | 152.645 |
| GL_Gan1_GLEAN_10004355 | Magnaporthe grisea | Loss of pathogenicity | 8.495 | 16.1 | 46.545 | 74.35 | 13.58 |
| GL_Gan1_GLEAN_10004361 | Cryptococcus neoformans | Reduced virulence | 58.175 | 28.04 | 49.13 | 53.92 | 32.305 |
| GL_Gan1_GLEAN_10004363 | Pseudomonas syringae | Unaffected pathogenicity | 59.87 | 92.24 | 84.85 | 62.37 | 100.65 |
| GL_Gan1_GLEAN_10004364 | Ustilago maydis | Loss of pathogenicity | 3.69 | 11.35 | 11.64 | 6.365 | 13.195 |
| GL_Gan1_GLEAN_10004365 | Candida albicans | Reduced virulence | 103.035 | 84.385 | 83.72 | 60.315 | 93.46 |
| GL_Gan1_GLEAN_10004369 | Fusarium graminearum | Reduced virulence | 35.45 | 24.945 | 28.595 | 23.165 | 29.565 |
| GL_Gan1_GLEAN_10004379 | Magnaporthe grisea | Unaffected pathogenicity | 67.785 | 26.08 | 39.78 | 39.025 | 41.4 |
| GL_Gan1_GLEAN_10004391 | Fusarium oxysporum | Loss of pathogenicity | 60.34 | 11 | 7.635 | 6.61 | 8.35 |
| GL_Gan1_GLEAN_10004394 | Cryptococcus neoformans | Reduced virulence | 38.645 | 22.32 | 43.885 | 47.575 | 33.02 |
| GL_Gan1_GLEAN_10004398 | Fusarium graminearum | Unaffected pathogenicity | 2.09 | 0 | 0.5 | 0.245 | 0.44 |
| GL_Gan1_GLEAN_10004415 | Alternaria alternata | Loss of pathogenicity | 8.755 | 1.99 | 1.885 | 1.505 | 1.275 |
| GL_Gan1_GLEAN_10004418 | Magnaporthe grisea | Loss of pathogenicity | 8.745 | 5.85 | 1.87 | 2.255 | 4.97 |
| GL_Gan1_GLEAN_10004425 | Cochliobolus carbonum | Loss of pathogenicity | 4.43 | 10.85 | 6.455 | 4.06 | 6.935 |
| GL_Gan1_GLEAN_10004427 | Cercospora nicotianae | Reduced virulence | 11.205 | 18.985 | 12.8 | 13.04 | 16.715 |
| GL_Gan1_GLEAN_10004428 | Botrytis cinerea | Reduced virulence | 1.135 | 6.19 | 2.9 | 2.92 | 4.575 |
| GL_Gan1_GLEAN_10004432 | Magnaporthe grisea | Effector (plant avirulence determinant) | 41.95 | 27.61 | 26.745 | 19.725 | 18.31 |
| GL_Gan1_GLEAN_10001244 | Fusarium oxysporum | Unaffected pathogenicity | 25.465 | 9.235 | 13.37 | 10.785 | 9.965 |
| GL_Gan1_GLEAN_10001248 | Botrytis cinerea | Unaffected pathogenicity | 18.17 | 2.47 | 4.615 | 3.475 | 3.685 |
| GL_Gan1_GLEAN_10001251 | Botrytis cinerea | Reduced virulence | 402.49 | 330.045 | 279.405 | 295.75 | 248.96 |
| GL_Gan1_GLEAN_10009012 | Gloeocercospora sorghi | Unaffected pathogenicity | 9.625 | 53.06 | 18.08 | 19.025 | 72.205 |
| GL_Gan1_GLEAN_10001261 | Botrytis cinerea | Unaffected pathogenicity | 5.5 | 3.07 | 2.255 | 2.18 | 4.16 |
| GL_Gan1_GLEAN_10000815 | Gloeocercospora sorghi | Unaffected pathogenicity | 0 | 44.915 | 10.105 | 3.505 | 37.78 |
| GL_Gan1_GLEAN_10007234 | Gloeocercospora sorghi | Unaffected pathogenicity | 7.23 | 225.6 | 105.875 | 164.255 | 236.84 |
| GL_Gan1_GLEAN_10003319 | Botrytis cinerea | Unaffected pathogenicity | 1.53 | 9.085 | 6.545 | 6.65 | 19.295 |
| GL_Gan1_GLEAN_10003322 | Salmonella enterica | Unaffected pathogenicity | 3.69 | 2.65 | 1.59 | 1.765 | 1.885 |
| GL_Gan1_GLEAN_10003324 | Magnaporthe grisea | Reduced virulence | 140.355 | 338.44 | 192.64 | 174.55 | 353.64 |
| GL_Gan1_GLEAN_10008802 | Histoplasma capsulatum | Loss of pathogenicity | 111.995 | 240.45 | 218.93 | 233.695 | 223.125 |
| GL_Gan1_GLEAN_10003328 | Ustilago maydis | Unaffected pathogenicity | 52.655 | 12.13 | 10.23 | 7.825 | 11.48 |
| GL_Gan1_GLEAN_10003329 | Magnaporthe grisea | Loss of pathogenicity | 499.09 | 172.07 | 369.39 | 415.235 | 324.5 |
| GL_Gan1_GLEAN_10003330 | Aspergillus fumigatus | Reduced virulence | 18.07 | 71.52 | 89.61 | 51.94 | 90.1 |
| GL_Gan1_GLEAN_10003335 | Candida albicans | Reduced virulence | 5.72 | 3.165 | 4.12 | 5.26 | 3.935 |
| GL_Gan1_GLEAN_10003340 | Magnaporthe grisea | Reduced virulence | 182.36 | 42.32 | 33.9 | 31.405 | 29.92 |
| GL_Gan1_GLEAN_10003341 | Fusarium graminearum | Unaffected pathogenicity | 45.335 | 82.185 | 13.475 | 15.415 | 59.94 |
| GL_Gan1_GLEAN_10003342 | Botrytis cinerea | Unaffected pathogenicity | 2.48 | 0.73 | 0.695 | 1.175 | 3.015 |
| GL_Gan1_GLEAN_10000702 | Leptosphaeria maculans | Reduced virulence | 9.175 | 13.985 | 11.79 | 9.375 | 13.18 |
| GL_Gan1_GLEAN_10003346 | Candida albicans | Reduced virulence | 25.635 | 10.36 | 8.56 | 9.81 | 13.135 |
| GL_Gan1_GLEAN_10003351 | Saccharomyces cerevisiae | Reduced virulence | 269.475 | 236.865 | 315.02 | 348.015 | 269.125 |
| GL_Gan1_GLEAN_10003370 | Candida albicans | Reduced virulence | 41.43 | 7.11 | 5.17 | 7.38 | 5.505 |
| GL_Gan1_GLEAN_10003373 | Magnaporthe grisea | Reduced virulence | 13.465 | 17.975 | 20.97 | 18.57 | 21.575 |
| GL_Gan1_GLEAN_10003376 | Botrytis cinerea | Unaffected pathogenicity | 0 | 0.81 | 1.44 | 1.285 | 1.045 |
| GL_Gan1_GLEAN_10000236 | Candida albicans | Loss of pathogenicity | 24.38 | 15.185 | 25.69 | 22.95 | 26.76 |
| GL_Gan1_GLEAN_10000238 | Colletotrichum lagenarium | Loss of pathogenicity | 0 | 1.305 | 0.19 | 0.79 | 0.71 |
| GL_Gan1_GLEAN_10001021 | Magnaporthe grisea | Reduced virulence | 0 | 7.01 | 0.715 | 0.465 | 6.96 |
| GL_Gan1_GLEAN_10006776 | Leptosphaeria maculans | Reduced virulence | 17.06 | 5.375 | 5.545 | 5.825 | 4.48 |
| GL_Gan1_GLEAN_10001057 | Cochliobolus carbonum | Reduced virulence | 16.38 | 23.02 | 31.13 | 33.315 | 28.145 |
| GL_Gan1_GLEAN_10000299 | Colletotrichum lagenarium | Reduced virulence | 2.725 | 12.085 | 4.235 | 3.37 | 12.165 |
| GL_Gan1_GLEAN_10000304 | Botrytis cinerea | Unaffected pathogenicity | 40.915 | 19.23 | 24.99 | 26.655 | 26.245 |
| GL_Gan1_GLEAN_10000305 | Fusarium oxysporum | Unaffected pathogenicity | 0 | 2.04 | 6.155 | 29.535 | 11.385 |
| GL_Gan1_GLEAN_10000309 | Stagonospora nodorum | Unaffected pathogenicity | 0.995 | 4.79 | 1.78 | 1.75 | 2.325 |
| GL_Gan1_GLEAN_10009386 | Leptosphaeria maculans | Reduced virulence | 363.59 | 225.64 | 211.055 | 200.35 | 266.46 |
| GL_Gan1_GLEAN_10000043 | Alternaria alternata | Loss of pathogenicity | 0 | 0.08 | 0.12 | 0.11 | 0.105 |
| GL_Gan1_GLEAN_10004779 | Magnaporthe grisea | Reduced virulence | 106.9 | 140.945 | 144.245 | 127.42 | 184.965 |
| GL_Gan1_GLEAN_10004787 | Aspergillus fumigatus | Reduced virulence | 616.865 | 436.88 | 555.64 | 628.275 | 224.9 |
| GL_Gan1_GLEAN_10004789 | Fusarium oxysporum | Loss of pathogenicity | 5.505 | 6.865 | 7.665 | 7.125 | 8.35 |
| GL_Gan1_GLEAN_10004798 | Fusarium oxysporum | Unaffected pathogenicity | 6.88 | 12.045 | 16.59 | 10.975 | 13.405 |
| GL_Gan1_GLEAN_10004800 | Candida albicans | Reduced virulence | 7.005 | 3.735 | 6.015 | 5.31 | 5.15 |
| GL_Gan1_GLEAN_10004801 | Cercospora nicotianae | Reduced virulence | 0 | 5.345 | 5.065 | 3.455 | 6.155 |
| GL_Gan1_GLEAN_10004805 | Botrytis cinerea | Unaffected pathogenicity | 27.355 | 8.56 | 5.95 | 8.485 | 7.76 |
| GL_Gan1_GLEAN_10004806 | Fusarium oxysporum | Loss of pathogenicity | 9.095 | 39.69 | 29.825 | 42.69 | 51.09 |
| GL_Gan1_GLEAN_10004810 | Magnaporthe grisea | Reduced virulence | 47.05 | 13.515 | 16.15 | 105 | 9.54 |
| GL_Gan1_GLEAN_10004813 | Cochliobolus carbonum | Unaffected pathogenicity | 12.71 | 4.365 | 5.015 | 2.795 | 5.71 |
| GL_Gan1_GLEAN_10004823 | Fusarium oxysporum | Reduced virulence | 56.85 | 1568.795 | 1658.13 | 1257.145 | 2169.415 |
| GL_Gan1_GLEAN_10004824 | Botrytis cinerea | Reduced virulence | 9.56 | 8.385 | 8.255 | 7.26 | 7 |
| GL_Gan1_GLEAN_10004826 | Cercospora nicotianae | Reduced virulence | 4.69 | 2.705 | 4.97 | 4.18 | 2.88 |
| GL_Gan1_GLEAN_10004829 | Colletotrichum lagenarium | Reduced virulence | 55.545 | 12.91 | 24.41 | 23.95 | 10.185 |
| GL_Gan1_GLEAN_10004831 | Botrytis cinerea | Reduced virulence | 1.605 | 0 | 0.87 | 0.75 | 1.05 |
| GL_Gan1_GLEAN_10004832 | Gibberella moniliformis | Unaffected pathogenicity | 25.205 | 6.135 | 6.65 | 8.45 | 6.66 |
| GL_Gan1_GLEAN_10004835 | Botrytis cinerea | Reduced virulence | 7.725 | 11.16 | 13.065 | 11.26 | 13.84 |
| GL_Gan1_GLEAN_10004836 | Fusarium oxysporum | Loss of pathogenicity | 34.045 | 13.41 | 15.965 | 17.05 | 14.545 |
| GL_Gan1_GLEAN_10004837 | Cryptococcus neoformans | Reduced virulence | 4.675 | 7.195 | 9.15 | 7.79 | 8.36 |
| GL_Gan1_GLEAN_10004838 | Magnaporthe grisea | Reduced virulence | 612.85 | 141.045 | 89.995 | 123.325 | 110.835 |
| GL_Gan1_GLEAN_10004840 | Cercospora kikuchii | Reduced virulence | 3.325 | 32.25 | 7.33 | 14.245 | 23.745 |
| GL_Gan1_GLEAN_10004842 | Botrytis cinerea | Unaffected pathogenicity | 5.225 | 9.64 | 9.675 | 10.595 | 9.235 |
| GL_Gan1_GLEAN_10008810 | Leptosphaeria maculans | Reduced virulence | 41.76 | 98.72 | 142.915 | 118.135 | 101.525 |
| GL_Gan1_GLEAN_10004845 | Fusarium graminearum | Reduced virulence | 0 | 11.86 | 7.41 | 6.795 | 10.315 |
| GL_Gan1_GLEAN_10006151 | Ustilago maydis | Loss of pathogenicity | 25.725 | 20.265 | 22.53 | 19.975 | 24.125 |
| GL_Gan1_GLEAN_10006155 | Gibberella moniliformis | Unaffected pathogenicity | 1.39 | 1.33 | 1.22 | 1.19 | 1.35 |
| GL_Gan1_GLEAN_10006157 | Botrytis cinerea | Reduced virulence | 0 | 1.39 | 0.19 | 0.315 | 0.38 |
| GL_Gan1_GLEAN_10006158 | Botrytis cinerea | Reduced virulence | 4.15 | 0.365 | 0.7 | 0.295 | 0.105 |
| GL_Gan1_GLEAN_10006162 | Fusarium graminearum | Unaffected pathogenicity | 1.425 | 4.845 | 2.66 | 2.425 | 2.77 |
| GL_Gan1_GLEAN_10006170 | Magnaporthe grisea | Unaffected pathogenicity | 812.375 | 308.465 | 348.68 | 457.525 | 158.315 |
| GL_Gan1_GLEAN_10003666 | Leptosphaeria maculans | Reduced virulence | 17.145 | 123.67 | 518.205 | 228.215 | 258.11 |
| GL_Gan1_GLEAN_10006175 | Ustilago maydis | Reduced virulence | 23.795 | 3.645 | 5.08 | 4.86 | 3.12 |
| GL_Gan1_GLEAN_10006180 | Botrytis cinerea | Unaffected pathogenicity | 9.24 | 7.82 | 14.185 | 13.175 | 10.34 |
| GL_Gan1_GLEAN_10006181 | Magnaporthe grisea | Reduced virulence | 6.55 | 16.655 | 24.425 | 14.775 | 14.495 |
| GL_Gan1_GLEAN_10006199 | Magnaporthe grisea | Reduced virulence | 8.125 | 20.645 | 28.96 | 30.93 | 27.415 |
| GL_Gan1_GLEAN_10006203 | Candida albicans | Reduced virulence | 25.395 | 34.875 | 24.785 | 27.15 | 28.56 |
| GL_Gan1_GLEAN_10006205 | Candida albicans | Reduced virulence | 80.34 | 317.23 | 353.195 | 359.735 | 448.435 |
| GL_Gan1_GLEAN_10006206 | Magnaporthe grisea | Loss of pathogenicity | 196.37 | 48.125 | 39.475 | 38.61 | 48.13 |
| GL_Gan1_GLEAN_10006209 | Fusarium oxysporum | Loss of pathogenicity | 0 | 0.525 | 0.25 | 0.685 | 0.4 |
| GL_Gan1_GLEAN_10006214 | Candida albicans | Reduced virulence | 167.425 | 35.535 | 63.775 | 53.545 | 50.915 |
| GL_Gan1_GLEAN_10002740 | Leptosphaeria maculans | Reduced virulence | 110.595 | 187.31 | 125.95 | 124.535 | 132.745 |
| GL_Gan1_GLEAN_10006223 | Cercospora zeae-maydis | Reduced virulence | 59.8 | 25.055 | 32.08 | 36.835 | 36.06 |
| GL_Gan1_GLEAN_10006231 | Cryptococcus neoformans | Reduced virulence | 4.615 | 27.59 | 29.525 | 29.435 | 33.635 |
| GL_Gan1_GLEAN_10006233 | Candida albicans | Reduced virulence | 42.265 | 74.71 | 149.295 | 138.57 | 81.075 |
| GL_Gan1_GLEAN_10006171 | Leptosphaeria maculans | Reduced virulence | 98.83 | 63.23 | 74.865 | 59.97 | 62.375 |
| GL_Gan1_GLEAN_10006246 | Candida albicans | Reduced virulence | 27.87 | 18.495 | 24.385 | 24.72 | 23.72 |
| GL_Gan1_GLEAN_10006247 | Colletotrichum gloeosporioides | Unaffected pathogenicity | 13.15 | 4.2 | 7.805 | 6.23 | 6.21 |
| GL_Gan1_GLEAN_10006254 | Magnaporthe grisea | Loss of pathogenicity | 33.955 | 30.91 | 26.145 | 35.43 | 31.26 |
| GL_Gan1_GLEAN_10006263 | Fusarium graminearum | Reduced virulence | 0 | 0.315 | 0 | 0 | 0 |
| GL_Gan1_GLEAN_10006267 | Candida albicans | Reduced virulence | 11.855 | 6.695 | 2.97 | 3.325 | 2.06 |
| GL_Gan1_GLEAN_10006272 | Cryptococcus neoformans | Loss of pathogenicity | 1.29 | 1.42 | 2.045 | 2.815 | 1.325 |
| GL_Gan1_GLEAN_10006274 | Candida albicans | Reduced virulence | 3.62 | 15.585 | 25.75 | 23.18 | 17.24 |
| GL_Gan1_GLEAN_10006290 | Cryptococcus neoformans | Reduced virulence | 53.66 | 40.555 | 89.605 | 90.19 | 60.35 |
| GL_Gan1_GLEAN_10006299 | Magnaporthe grisea | Reduced virulence | 2.635 | 13.415 | 22.435 | 36.005 | 10.74 |
| GL_Gan1_GLEAN_10006306 | Cochliobolus carbonum | Unaffected pathogenicity | 8.64 | 16.76 | 9.665 | 13.87 | 16.04 |
| GL_Gan1_GLEAN_10000509 | Aspergillus fumigatus | Loss of pathogenicity | 12.745 | 38.895 | 22.01 | 25.265 | 48.09 |
| GL_Gan1_GLEAN_10000511 | Magnaporthe grisea | Unaffected pathogenicity | 260.035 | 136.685 | 101.57 | 110.375 | 151.91 |
| GL_Gan1_GLEAN_10000515 | Candida albicans | Reduced virulence | 38.87 | 26.68 | 34.66 | 39.28 | 34.385 |
| GL_Gan1_GLEAN_10000516 | Magnaporthe grisea | Unaffected pathogenicity | 67.785 | 30.405 | 41.055 | 40.67 | 38.615 |
| GL_Gan1_GLEAN_10000523 | Fusarium oxysporum | Unaffected pathogenicity | 28.03 | 8.84 | 12.28 | 12.175 | 12.675 |
| GL_Gan1_GLEAN_10000524 | Botrytis cinerea | Unaffected pathogenicity | 27.36 | 11.585 | 6.23 | 7.38 | 9.845 |
| GL_Gan1_GLEAN_10002803 | Fusarium graminearum | Reduced virulence | 5.225 | 2.975 | 7.5 | 5.62 | 2.785 |
| GL_Gan1_GLEAN_10002817 | Candida albicans | Reduced virulence | 123.56 | 106.485 | 118.825 | 105.62 | 151.515 |
| GL_Gan1_GLEAN_10002824 | Magnaporthe grisea | Reduced virulence | 0 | 33.64 | 3.48 | 3.86 | 25.355 |
| GL_Gan1_GLEAN_10002825 | Botrytis cinerea | Reduced virulence | 21.255 | 32.07 | 27.09 | 25.44 | 32.455 |
| GL_Gan1_GLEAN_10002832 | Botrytis cinerea | Reduced virulence | 15.785 | 22.46 | 7.945 | 8.175 | 20.3 |
| GL_Gan1_GLEAN_10002836 | Botrytis cinerea | Reduced virulence | 14.87 | 2.56 | 0.645 | 0.41 | 3.27 |
| GL_Gan1_GLEAN_10002837 | Candida albicans | Reduced virulence | 42.25 | 434.64 | 374.985 | 231.47 | 555.735 |
| GL_Gan1_GLEAN_10005179 | Leptosphaeria maculans | Unaffected pathogenicity | 1259.465 | 1822.955 | 1412.01 | 1149.32 | 1567.065 |
| GL_Gan1_GLEAN_10002842 | Magnaporthe grisea | Reduced virulence | 25.5 | 56.98 | 49.995 | 48.39 | 38.105 |
| GL_Gan1_GLEAN_10002847 | Botrytis cinerea | Unaffected pathogenicity | 35.845 | 332.125 | 176 | 332.56 | 520.295 |
| GL_Gan1_GLEAN_10002850 | Cercospora nicotianae | Reduced virulence | 29.31 | 21.27 | 15.48 | 13.405 | 19.54 |
| GL_Gan1_GLEAN_10002854 | Magnaporthe grisea | Reduced virulence | 0 | 0 | 0.38 | 0.295 | 0.46 |
| GL_Gan1_GLEAN_10000145 | Botrytis cinerea | Reduced virulence | 73.37 | 122.885 | 117.78 | 120.27 | 113.385 |
| GL_Gan1_GLEAN_10005326 | Candida albicans | Reduced virulence | 11.39 | 20.255 | 29.78 | 22.605 | 26.915 |
| GL_Gan1_GLEAN_10005331 | Botrytis cinerea | Reduced virulence | 5.455 | 12.635 | 14.545 | 16.56 | 11.815 |
| GL_Gan1_GLEAN_10005337 | Candida albicans | Reduced virulence | 57.56 | 30.83 | 48.015 | 44.075 | 43.255 |
| GL_Gan1_GLEAN_10002555 | Leptosphaeria maculans | Reduced virulence | 25.84 | 91.14 | 77.96 | 52.675 | 90.795 |
| GL_Gan1_GLEAN_10005350 | Magnaporthe grisea | Reduced virulence | 133.56 | 12.405 | 9.57 | 11.64 | 10.125 |
| GL_Gan1_GLEAN_10005353 | Candida albicans | Reduced virulence | 2.81 | 9.74 | 13.365 | 15.7 | 12.75 |
| GL_Gan1_GLEAN_10005539 | Leptosphaeria maculans | Unaffected pathogenicity | 89.285 | 207.955 | 1071.515 | 848.495 | 250.75 |
| GL_Gan1_GLEAN_10005365 | Magnaporthe grisea | Reduced virulence | 20.325 | 29.18 | 34.41 | 33.125 | 37.865 |
| GL_Gan1_GLEAN_10005384 | Ustilago maydis | Unaffected pathogenicity | 0 | 4.015 | 1.185 | 1.885 | 3.355 |
| GL_Gan1_GLEAN_10005388 | Magnaporthe grisea | Reduced virulence | 43.03 | 31.59 | 56.25 | 44.485 | 42.785 |
| GL_Gan1_GLEAN_10005392 | Candida albicans | Reduced virulence | 31.115 | 26.215 | 33.82 | 37.53 | 33.52 |
| GL_Gan1_GLEAN_10006627 | Leptosphaeria maculans | Reduced virulence | 55.945 | 325.22 | 363.655 | 329.785 | 308.885 |
| GL_Gan1_GLEAN_10005403 | Botrytis cinerea | Unaffected pathogenicity | 0 | 0.805 | 2.015 | 0.86 | 1.07 |
| GL_Gan1_GLEAN_10005404 | Cryptococcus neoformans | Reduced virulence | 71.87 | 42.675 | 66.9 | 79.105 | 57.2 |
| GL_Gan1_GLEAN_10005405 | Candida albicans | Reduced virulence | 8.92 | 17.52 | 8.5 | 8 | 22.575 |
| GL_Gan1_GLEAN_10005409 | Fusarium oxysporum | Unaffected pathogenicity | 106.265 | 10.05 | 8.85 | 10.48 | 4.08 |
| GL_Gan1_GLEAN_10005411 | Colletotrichum gloeosporioides | Unaffected pathogenicity | 29.22 | 63.74 | 87.5 | 72.055 | 104.23 |
| GL_Gan1_GLEAN_10005416 | Candida albicans | Reduced virulence | 9.855 | 5.64 | 2.59 | 6.655 | 8.25 |
| GL_Gan1_GLEAN_10005422 | Botrytis cinerea | Reduced virulence | 0 | 0.375 | 1.2 | 1.56 | 1.155 |
| GL_Gan1_GLEAN_10005423 | Magnaporthe grisea | Effector (plant avirulence determinant) | 2.83 | 8.125 | 21.055 | 9.755 | 8.14 |
| GL_Gan1_GLEAN_10005431 | Phytophthora infestans | Reduced virulence | 34.23 | 25.5 | 21.375 | 22.865 | 25.09 |
| GL_Gan1_GLEAN_10005434 | Saccharomyces cerevisiae | Reduced virulence | 94.815 | 83.18 | 73.895 | 74.445 | 80.29 |
| GL_Gan1_GLEAN_10005440 | Cochliobolus heterostrophus | Reduced virulence | 0.31 | 0.04 | 0.19 | 0.195 | 0.17 |
| GL_Gan1_GLEAN_10008370 | Pseudomonas syringae | Effector (plant avirulence determinant) | 16.025 | 15.305 | 26.32 | 22.18 | 22.76 |
| GL_Gan1_GLEAN_10005578 | Magnaporthe grisea | Reduced virulence | 11.275 | 24.24 | 60.69 | 51.5 | 24.53 |
| GL_Gan1_GLEAN_10005585 | Alternaria alternata | Loss of pathogenicity | 28.09 | 38.815 | 31.17 | 24.495 | 48.48 |
| GL_Gan1_GLEAN_10005586 | Botrytis cinerea | Unaffected pathogenicity | 122.715 | 31.09 | 47.075 | 49.27 | 38.9 |
| GL_Gan1_GLEAN_10005588 | Candida albicans | Reduced virulence | 55.48 | 10.78 | 19.295 | 19.045 | 11.86 |
| GL_Gan1_GLEAN_10005597 | Cercospora nicotianae | Reduced virulence | 6.495 | 54.145 | 7.56 | 12.675 | 51.14 |
| GL_Gan1_GLEAN_10005598 | Botrytis cinerea | Reduced virulence | 0 | 6.175 | 2.395 | 2.37 | 4.14 |
| GL_Gan1_GLEAN_10005599 | Ustilago maydis | Unaffected pathogenicity | 6.925 | 2.67 | 5.345 | 3.175 | 2.855 |
| GL_Gan1_GLEAN_10005605 | Magnaporthe grisea | Reduced virulence | 55.05 | 71.51 | 69.165 | 56.61 | 82.905 |
| GL_Gan1_GLEAN_10004253 | Pseudomonas syringae | Effector (plant avirulence determinant) | 350.985 | 131.495 | 98.97 | 109.095 | 113.275 |
| GL_Gan1_GLEAN_10005609 | Magnaporthe grisea | Reduced virulence | 23.615 | 13.09 | 14 | 16.35 | 18.275 |
| GL_Gan1_GLEAN_10005616 | Mycosphaerella graminicola | Reduced virulence | 513.5 | 164.21 | 285.525 | 279.605 | 261.22 |
| GL_Gan1_GLEAN_10005624 | Cochliobolus heterostrophus | Loss of pathogenicity | 19.46 | 105.305 | 74.985 | 72.96 | 119.725 |
| GL_Gan1_GLEAN_10006870 | Pseudomonas syringae | Effector (plant avirulence determinant) | 38.34 | 16.195 | 12.575 | 14.12 | 15.065 |
| GL_Gan1_GLEAN_10005627 | Alternaria alternata | Loss of pathogenicity | 9.085 | 9.895 | 11.7 | 10.715 | 8.64 |
| GL_Gan1_GLEAN_10005645 | Cochliobolus carbonum | Loss of pathogenicity | 371.445 | 152.775 | 122.5 | 235.39 | 141.65 |
| GL_Gan1_GLEAN_10005650 | Candida albicans | Reduced virulence | 38.465 | 29.13 | 22.74 | 16.54 | 24.88 |
| GL_Gan1_GLEAN_10005653 | Cercospora nicotianae | Reduced virulence | 94.555 | 516.55 | 344.13 | 336.82 | 371.83 |
| GL_Gan1_GLEAN_10005657 | Magnaporthe grisea | Reduced virulence | 71.08 | 61.06 | 39.1 | 38.23 | 66.8 |
| GL_Gan1_GLEAN_10005660 | Cochliobolus miyabeanus | Reduced virulence | 5.185 | 5.72 | 3.06 | 3.355 | 7.615 |
| GL_Gan1_GLEAN_10005661 | Magnaporthe grisea | Loss of pathogenicity | 0 | 6.43 | 0.82 | 1.39 | 4.55 |
| GL_Gan1_GLEAN_10005663 | Alternaria alternata | Loss of pathogenicity | 1.53 | 10.76 | 6.945 | 5.315 | 7.965 |
| GL_Gan1_GLEAN_10005664 | Candida albicans | Reduced virulence | 0 | 0.905 | 0.06 | 0.215 | 0.76 |
| GL_Gan1_GLEAN_10005669 | Cochliobolus carbonum | Unaffected pathogenicity | 33.03 | 1030.445 | 916.41 | 1005.12 | 1108.905 |
| GL_Gan1_GLEAN_10005671 | Alternaria alternata | Loss of pathogenicity | 29.055 | 19.975 | 22.265 | 20.135 | 26.62 |
| GL_Gan1_GLEAN_10006915 | Pseudomonas syringae | Effector (plant avirulence determinant) | 62.8 | 88.22 | 91.81 | 106.26 | 79.885 |
| GL_Gan1_GLEAN_10005678 | Colletotrichum lagenarium | Loss of pathogenicity | 9.68 | 13.63 | 5.87 | 4.82 | 14.49 |
| GL_Gan1_GLEAN_10005694 | Cercospora kikuchii | Reduced virulence | 16.865 | 158.97 | 255.705 | 264.185 | 309.785 |
| GL_Gan1_GLEAN_10005695 | Fusarium oxysporum | Loss of pathogenicity | 64.37 | 34.335 | 46.245 | 40.745 | 33.265 |
| GL_Gan1_GLEAN_10005697 | Fusarium oxysporum | Loss of pathogenicity | 4.665 | 28.875 | 49.7 | 48.2 | 33.17 |
| GL_Gan1_GLEAN_10005698 | Botrytis cinerea | Reduced virulence | 0 | 3.34 | 1.69 | 1.77 | 1.05 |
| GL_Gan1_GLEAN_10005699 | Candida albicans | Reduced virulence | 0 | 2.39 | 2.7 | 2.915 | 1.845 |
| GL_Gan1_GLEAN_10005700 | Cochliobolus heterostrophus | Reduced virulence | 8.4 | 57.2 | 14.405 | 30.39 | 62.67 |
| GL_Gan1_GLEAN_10005702 | Stagonospora nodorum | Unaffected pathogenicity | 2.825 | 148.49 | 20.53 | 137.475 | 184.325 |
| GL_Gan1_GLEAN_10005703 | Aspergillus fumigatus | Reduced virulence | 0.54 | 17.74 | 2.63 | 17.475 | 30.45 |
| GL_Gan1_GLEAN_10005707 | Alternaria alternata | Loss of pathogenicity | 281.415 | 133.13 | 144.995 | 129.285 | 160.175 |
| GL_Gan1_GLEAN_10005073 | Botrytis cinerea | Unaffected pathogenicity | 3.905 | 1.79 | 1.2 | 0.995 | 5.035 |
| GL_Gan1_GLEAN_10005074 | Botrytis cinerea | Unaffected pathogenicity | 0 | 0 | 0.085 | 0.035 | 0 |
| GL_Gan1_GLEAN_10005075 | Cryptococcus neoformans | Reduced virulence | 8.18 | 5.225 | 5.795 | 7.255 | 4.56 |
| GL_Gan1_GLEAN_10005077 | Ustilago maydis | Unaffected pathogenicity | 6.225 | 12.845 | 7.01 | 9.08 | 11.63 |
| GL_Gan1_GLEAN_10005079 | Cochliobolus heterostrophus | Reduced virulence | 4.86 | 5.235 | 4.735 | 2.975 | 6.17 |
| GL_Gan1_GLEAN_10005083 | Candida albicans | Reduced virulence | 148.7 | 304.375 | 232.35 | 263.1 | 248.83 |
| GL_Gan1_GLEAN_10005085 | Colletotrichum lagenarium | Loss of pathogenicity | 100.44 | 94.47 | 76.76 | 62.565 | 128.66 |
| GL_Gan1_GLEAN_10008666 | Pseudomonas syringae | Effector (plant avirulence determinant) | 243.425 | 300.515 | 345.67 | 286.97 | 368.785 |
| GL_Gan1_GLEAN_10005117 | Magnaporthe grisea | Reduced virulence | 192.625 | 628.805 | 549.995 | 520.915 | 492.745 |
| GL_Gan1_GLEAN_10005123 | Aspergillus fumigatus | Reduced virulence | 24.14 | 10.27 | 19.515 | 12.14 | 14.45 |
| GL_Gan1_GLEAN_10005124 | Ustilago maydis | Reduced virulence | 27.82 | 46.71 | 41.965 | 39.055 | 55.645 |
| GL_Gan1_GLEAN_10005126 | Botrytis cinerea | Unaffected pathogenicity | 0 | 0.835 | 0.56 | 0.605 | 0.245 |
| GL_Gan1_GLEAN_10005144 | Cryptococcus neoformans | Loss of pathogenicity | 351.095 | 1722.61 | 2731.485 | 2004.255 | 2445.12 |
| GL_Gan1_GLEAN_10005145 | Candida albicans | Reduced virulence | 2.07 | 3.63 | 3.36 | 2.29 | 0.82 |
| GL_Gan1_GLEAN_10005146 | Magnaporthe grisea | Loss of pathogenicity | 25.005 | 24.11 | 27.73 | 28.39 | 24.545 |
| GL_Gan1_GLEAN_10005152 | Candida albicans | Reduced virulence | 16.93 | 8.685 | 11.185 | 9.94 | 9.435 |
| GL_Gan1_GLEAN_10005161 | Magnaporthe grisea | Reduced virulence | 12.445 | 27.43 | 31.04 | 26.8 | 35.395 |
| GL_Gan1_GLEAN_10005162 | Candida albicans | Loss of pathogenicity | 13.215 | 29.515 | 18.845 | 20.725 | 32.23 |
| GL_Gan1_GLEAN_10006310 | Pseudomonas syringae | Effector (plant avirulence determinant) | 84.335 | 28.625 | 38.735 | 39.54 | 28.125 |
| GL_Gan1_GLEAN_10003108 | Pseudomonas syringae | Effector (plant avirulence determinant) | 53.59 | 25.12 | 20.205 | 22.67 | 22.575 |
| GL_Gan1_GLEAN_10005177 | Ustilago maydis | Loss of pathogenicity | 84.93 | 44.785 | 47.925 | 47.105 | 54.85 |
| GL_Gan1_GLEAN_10005178 | Colletotrichum lagenarium | Loss of pathogenicity | 196.705 | 167.955 | 134.765 | 136.15 | 167.175 |
| GL_Gan1_GLEAN_10007994 | Pseudomonas syringae | Effector (plant avirulence determinant) | 175.02 | 124.25 | 82.215 | 49.73 | 109 |
| GL_Gan1_GLEAN_10000849 | Ustilago maydis | Unaffected pathogenicity | 0 | 0.47 | 0.315 | 0.53 | 0.09 |
| GL_Gan1_GLEAN_10003911 | Colletotrichum lindemuthianum | Reduced virulence | 261.205 | 283.45 | 281.345 | 314.29 | 278.85 |
| GL_Gan1_GLEAN_10003912 | Colletotrichum lagenarium | Loss of pathogenicity | 90.77 | 32.27 | 48.73 | 54.01 | 33.84 |
| GL_Gan1_GLEAN_10003914 | Candida albicans | Loss of pathogenicity | 22.645 | 10.615 | 14.815 | 14.51 | 12.15 |
| GL_Gan1_GLEAN_10003927 | Colletotrichum lagenarium | Loss of pathogenicity | 82.155 | 132.915 | 99.235 | 101.73 | 140.945 |
| GL_Gan1_GLEAN_10003944 | Colletotrichum lagenarium | Reduced virulence | 56.595 | 83.6 | 68.795 | 60.9 | 94.475 |
| GL_Gan1_GLEAN_10003946 | Botrytis cinerea | Unaffected pathogenicity | 2.625 | 27.17 | 21.645 | 22.3 | 18.435 |
| GL_Gan1_GLEAN_10003950 | Fusarium graminearum | Reduced virulence | 136.29 | 127.16 | 119.84 | 147.435 | 119.33 |
| GL_Gan1_GLEAN_10003953 | Fusarium oxysporum | Unaffected pathogenicity | 25.87 | 10.36 | 15.61 | 14.525 | 14.855 |
| GL_Gan1_GLEAN_10003954 | Candida albicans | Reduced virulence | 19.18 | 65.59 | 23.71 | 28.62 | 50.325 |
| GL_Gan1_GLEAN_10003956 | Magnaporthe grisea | Reduced virulence | 50.27 | 62.445 | 38.73 | 47.265 | 52.725 |
| GL_Gan1_GLEAN_10003958 | Botrytis cinerea | Unaffected pathogenicity | 5.07 | 96.72 | 15.16 | 15.31 | 97.265 |
| GL_Gan1_GLEAN_10003959 | Botrytis cinerea | Reduced virulence | 921.915 | 802.41 | 820.425 | 814.305 | 776.73 |
| GL_Gan1_GLEAN_10003964 | Magnaporthe grisea | Reduced virulence | 91.105 | 104.515 | 63.585 | 51.295 | 85.45 |
| GL_Gan1_GLEAN_10003965 | Mycosphaerella graminicola | Unaffected pathogenicity | 6.6 | 6.025 | 7.645 | 4.1 | 5.345 |
| GL_Gan1_GLEAN_10003966 | Magnaporthe grisea | Reduced virulence | 9.29 | 11.33 | 12.915 | 11.12 | 9.75 |
| GL_Gan1_GLEAN_10003967 | Mycosphaerella graminicola | Unaffected pathogenicity | 0.935 | 4.12 | 4.595 | 3.18 | 6.725 |
| GL_Gan1_GLEAN_10003306 | Pseudomonas syringae | Effector (plant avirulence determinant) | 113.86 | 76.175 | 73.795 | 72.81 | 79.32 |
| GL_Gan1_GLEAN_10003981 | Fusarium oxysporum | Reduced virulence | 10.68 | 16.21 | 8.91 | 10.42 | 19.585 |
| GL_Gan1_GLEAN_10003995 | Candida albicans | Reduced virulence | 223.035 | 8.3 | 11.205 | 12.04 | 4.85 |
| GL_Gan1_GLEAN_10004008 | Magnaporthe grisea | Reduced virulence | 24.505 | 15.665 | 22.32 | 19.035 | 15.275 |
| GL_Gan1_GLEAN_10004011 | Candida albicans | Reduced virulence | 40.105 | 18.7 | 26.615 | 24.055 | 30.69 |
| GL_Gan1_GLEAN_10004013 | Botrytis cinerea | Reduced virulence | 0 | 58.825 | 70.625 | 71.305 | 174.115 |
| GL_Gan1_GLEAN_10004019 | Ustilago maydis | Loss of pathogenicity | 4.81 | 22.52 | 24.05 | 21.27 | 26.65 |
| GL_Gan1_GLEAN_10004021 | Botrytis cinerea | Reduced virulence | 0 | 0.485 | 1.825 | 1.04 | 0.35 |
| GL_Gan1_GLEAN_10001698 | Botrytis cinerea | Reduced virulence | 54.14 | 20.995 | 18.885 | 15.26 | 22.885 |
| GL_Gan1_GLEAN_10001700 | Fusarium oxysporum | Unaffected pathogenicity | 82.545 | 65.295 | 74.665 | 78.07 | 52.995 |
| GL_Gan1_GLEAN_10001701 | Fusarium oxysporum | Loss of pathogenicity | 12.325 | 4.835 | 4.345 | 5.76 | 6.875 |
| GL_Gan1_GLEAN_10001703 | Ustilago maydis | Reduced virulence | 0.42 | 7.205 | 4.685 | 3.29 | 8.305 |
| GL_Gan1_GLEAN_10001711 | Botrytis cinerea | Reduced virulence | 442.165 | 288.865 | 415.33 | 498.27 | 312.35 |
| GL_Gan1_GLEAN_10001722 | Cercospora zeae-maydis | Unaffected pathogenicity | 60.435 | 18.4 | 10.995 | 15.635 | 16.46 |
| GL_Gan1_GLEAN_10001727 | Stagonospora nodorum | Unaffected pathogenicity | 3.84 | 49.905 | 22.56 | 21.69 | 36.68 |
| GL_Gan1_GLEAN_10001729 | Candida albicans | Reduced virulence | 43.28 | 24.865 | 25.415 | 22.015 | 22.525 |
| GL_Gan1_GLEAN_10001730 | Botrytis cinerea | Unaffected pathogenicity | 0.505 | 4.645 | 4.885 | 4.355 | 4.58 |
| GL_Gan1_GLEAN_10001733 | Ustilago maydis | Unaffected pathogenicity | 0 | 0 | 0.045 | 0.08 | 0 |
| GL_Gan1_GLEAN_10000314 | Pseudomonas syringae | Effector (plant avirulence determinant) | 227.07 | 171.07 | 156.98 | 181.745 | 169.325 |
| GL_Gan1_GLEAN_10000089 | Cochliobolus carbonum | Reduced virulence | 3.42 | 11.915 | 3.1 | 3.97 | 11.37 |
| GL_Gan1_GLEAN_10006984 | Cercospora zeae-maydis | Reduced virulence | 46.5 | 110.535 | 121.17 | 118.375 | 157.77 |
| GL_Gan1_GLEAN_10006987 | Candida albicans | Loss of pathogenicity | 639.585 | 1233.22 | 1097.4 | 1219 | 1089.095 |
| GL_Gan1_GLEAN_10005441 | Pseudomonas syringae | Effector (plant avirulence determinant) | 34.185 | 78.3 | 66.13 | 76.255 | 72.905 |
| GL_Gan1_GLEAN_10006993 | Botrytis cinerea | Unaffected pathogenicity | 0 | 0.175 | 0.43 | 0.205 | 0.58 |
| GL_Gan1_GLEAN_10007000 | Fusarium oxysporum | Reduced virulence | 143.775 | 20.87 | 25.19 | 21.79 | 27.2 |
| GL_Gan1_GLEAN_10007002 | Candida albicans | Loss of pathogenicity | 17.315 | 21.845 | 25.235 | 21.17 | 28.215 |
| GL_Gan1_GLEAN_10007003 | Aspergillus fumigatus | Loss of pathogenicity | 38.155 | 64.46 | 51.815 | 42.745 | 73.475 |
| GL_Gan1_GLEAN_10007010 | Botrytis cinerea | Unaffected pathogenicity | 1.43 | 1.88 | 2.82 | 0.8 | 4.065 |
| GL_Gan1_GLEAN_10007012 | Magnaporthe grisea | Reduced virulence | 31.55 | 46.655 | 33.07 | 30.53 | 50.07 |
| GL_Gan1_GLEAN_10007019 | Colletotrichum gloeosporioides | Loss of pathogenicity | 722.465 | 1989.85 | 3018.14 | 3015.395 | 1398.9 |
| GL_Gan1_GLEAN_10007026 | Phytophthora infestans | Reduced virulence | 83.495 | 60.825 | 115.805 | 125.92 | 85.455 |
| GL_Gan1_GLEAN_10007065 | Pseudomonas syringae | Effector (plant avirulence determinant) | 135.26 | 148.12 | 158.25 | 120.12 | 185.035 |
| GL_Gan1_GLEAN_10007037 | Magnaporthe grisea | Reduced virulence | 11.155 | 13.545 | 21.325 | 16.285 | 18.09 |
| GL_Gan1_GLEAN_10007038 | Colletotrichum lagenarium | Loss of pathogenicity | 95.745 | 209.82 | 190.715 | 181.725 | 203 |
| GL_Gan1_GLEAN_10000465 | Rhynchosporium secalis | Resistant to chemical | 37.695 | 4.625 | 3.55 | 3.45 | 3.665 |
| GL_Gan1_GLEAN_10007047 | Candida albicans | Reduced virulence | 1.43 | 15.62 | 7.915 | 5.66 | 16.135 |
| GL_Gan1_GLEAN_10007062 | Fusarium oxysporum | Unaffected pathogenicity | 14.84 | 13.295 | 13.655 | 12.52 | 11.33 |
| GL_Gan1_GLEAN_10007063 | Claviceps purpurea | Loss of pathogenicity | 25.09 | 35.715 | 29.295 | 32.195 | 39.73 |
| GL_Gan1_GLEAN_10000870 | Rhynchosporium secalis | Resistant to chemical | 95.405 | 1019.63 | 676.675 | 789.525 | 1034.925 |
| GL_Gan1_GLEAN_10007068 | Magnaporthe grisea | Loss of pathogenicity | 27.77 | 89.59 | 117.165 | 97.95 | 122.08 |
| GL_Gan1_GLEAN_10007080 | Alternaria alternata | Loss of pathogenicity | 29.925 | 31.735 | 29.515 | 32.135 | 39.57 |
| GL_Gan1_GLEAN_10007090 | Trichoderma virens | Reduced virulence | 1.185 | 1.155 | 1.04 | 1.38 | 1.83 |
| GL_Gan1_GLEAN_10007104 | Magnaporthe grisea | Reduced virulence | 6.005 | 27.22 | 12.915 | 15.11 | 26.82 |
| GL_Gan1_GLEAN_10007108 | Candida albicans | Reduced virulence | 7.645 | 6.955 | 9.785 | 11.49 | 1.73 |
| GL_Gan1_GLEAN_10007119 | Magnaporthe grisea | Reduced virulence | 17.82 | 73.955 | 95.815 | 82.77 | 113.05 |
| GL_Gan1_GLEAN_10007120 | Cochliobolus carbonum | Reduced virulence | 41.945 | 28.295 | 28.335 | 32.545 | 32.005 |
| GL_Gan1_GLEAN_10007127 | Botrytis cinerea | Unaffected pathogenicity | 26.455 | 43.04 | 62.75 | 50.345 | 60.51 |
| GL_Gan1_GLEAN_10004559 | Rhynchosporium secalis | Resistant to chemical | 24.02 | 28.08 | 29.265 | 27.485 | 28.65 |
| GL_Gan1_GLEAN_10007131 | Candida albicans | Reduced virulence | 148.005 | 83.71 | 104.49 | 106.545 | 102.59 |
| GL_Gan1_GLEAN_10007138 | Candida albicans | Reduced virulence | 0 | 1.68 | 1.595 | 1.575 | 0.825 |
| GL_Gan1_GLEAN_10007139 | Candida albicans | Reduced virulence | 24.095 | 16.315 | 18.99 | 19.79 | 17.46 |
| GL_Gan1_GLEAN_10007149 | Fusarium oxysporum | Loss of pathogenicity | 34.965 | 21.31 | 26.185 | 23.05 | 29.225 |
| GL_Gan1_GLEAN_10007151 | Magnaporthe grisea | Unaffected pathogenicity | 1 | 5.27 | 6.62 | 6.43 | 6.64 |
| GL_Gan1_GLEAN_10007152 | Magnaporthe grisea | Reduced virulence | 4.445 | 9.385 | 11.12 | 10.485 | 9.035 |
| GL_Gan1_GLEAN_10000069 | Candida albicans | Reduced virulence | 36.765 | 25 | 38.98 | 44.21 | 32.745 |
| GL_Gan1_GLEAN_10000182 | Candida albicans | Reduced virulence | 19.12 | 23.07 | 12.735 | 12.59 | 18.595 |
| GL_Gan1_GLEAN_10008852 | Salmonella enterica | Reduced virulence | 28.625 | 9.645 | 10.815 | 10.345 | 15.035 |
| GL_Gan1_GLEAN_10002008 | Colletotrichum lindemuthianum | Reduced virulence | 8.08 | 12.57 | 8.105 | 7.835 | 19.945 |
| GL_Gan1_GLEAN_10002012 | Aspergillus fumigatus | Loss of pathogenicity | 67.315 | 40.755 | 20.145 | 19.315 | 38.845 |
| GL_Gan1_GLEAN_10002015 | Candida albicans | Reduced virulence | 29.065 | 35.585 | 52.74 | 51.185 | 39.64 |
| GL_Gan1_GLEAN_10002019 | Magnaporthe grisea | Loss of pathogenicity | 320.12 | 292.54 | 306.73 | 300.74 | 294.99 |
| GL_Gan1_GLEAN_10002023 | Magnaporthe grisea | Reduced virulence | 137.275 | 45.005 | 65.645 | 61.105 | 73.225 |
| GL_Gan1_GLEAN_10002031 | Colletotrichum lindemuthianum | Loss of pathogenicity | 48.305 | 57.175 | 56.44 | 72.025 | 80.635 |
| GL_Gan1_GLEAN_10002035 | Candida albicans | Loss of pathogenicity | 62.18 | 61.34 | 80.885 | 68.665 | 80.8 |
| GL_Gan1_GLEAN_10002038 | Botrytis cinerea | Reduced virulence | 1.09 | 29.09 | 79.455 | 66.035 | 37.29 |
| GL_Gan1_GLEAN_10002040 | Magnaporthe grisea | Reduced virulence | 1225.785 | 294.495 | 314.14 | 321.185 | 228.755 |
| GL_Gan1_GLEAN_10002045 | Botrytis cinerea | Unaffected pathogenicity | 3.765 | 0.83 | 0.81 | 0.905 | 1.67 |
| GL_Gan1_GLEAN_10003226 | Aspergillus fumigatus | Reduced virulence | 0 | 1.585 | 2.28 | 2.075 | 0.965 |
| GL_Gan1_GLEAN_10003227 | Botrytis cinerea | Reduced virulence | 9.05 | 19.175 | 9.785 | 14.925 | 23.59 |
| GL_Gan1_GLEAN_10003228 | Colletotrichum lagenarium | Reduced virulence | 15.69 | 144.12 | 116.825 | 154.685 | 128.84 |
| GL_Gan1_GLEAN_10003241 | Magnaporthe grisea | Loss of pathogenicity | 12.765 | 8.745 | 12.86 | 9.265 | 9.005 |
| GL_Gan1_GLEAN_10003242 | Botrytis cinerea | Unaffected pathogenicity | 1.815 | 76.34 | 11.235 | 6.68 | 75.77 |
| GL_Gan1_GLEAN_10003247 | Candida tropicalis | Reduced virulence | 1.725 | 7.85 | 4.945 | 3.695 | 6.33 |
| GL_Gan1_GLEAN_10003252 | Fusarium graminearum | Unaffected pathogenicity | 75.405 | 1.325 | 0.68 | 1.285 | 0.91 |
| GL_Gan1_GLEAN_10003257 | Fusarium graminearum | Loss of pathogenicity | 151.925 | 171.685 | 166.8 | 187.485 | 173.005 |
| GL_Gan1_GLEAN_10003260 | Ustilago maydis | Reduced virulence | 6.82 | 11.955 | 6.73 | 8.12 | 10.785 |
| GL_Gan1_GLEAN_10003266 | Candida albicans | Reduced virulence | 8.19 | 0.985 | 1.66 | 1.755 | 1.575 |
| GL_Gan1_GLEAN_10003267 | Colletotrichum gloeosporioides | Loss of pathogenicity | 63.15 | 48.045 | 54.975 | 60.49 | 50.045 |
| GL_Gan1_GLEAN_10003274 | Candida albicans | Reduced virulence | 60.23 | 41.24 | 58.81 | 64.605 | 39.195 |
| GL_Gan1_GLEAN_10003278 | Fusarium oxysporum | Loss of pathogenicity | 4.735 | 5.185 | 6.335 | 7.57 | 7.465 |
| GL_Gan1_GLEAN_10003287 | Magnaporthe grisea | Reduced virulence | 21.47 | 63.705 | 108.275 | 90.305 | 85.12 |
| GL_Gan1_GLEAN_10003289 | Botrytis cinerea | Unaffected pathogenicity | 156.075 | 38.41 | 50.855 | 45.055 | 27.68 |
| GL_Gan1_GLEAN_10003290 | Candida albicans | Loss of pathogenicity | 27.86 | 21.57 | 20.065 | 20.8 | 23.79 |
| GL_Gan1_GLEAN_10002471 | Salmonella enterica | Reduced virulence | 67.585 | 105.07 | 80.215 | 89.79 | 110.425 |
| GL_Gan1_GLEAN_10003292 | Mycosphaerella graminicola | Sensitive to chemical | 109.515 | 239.995 | 290.98 | 275.2 | 288.105 |
| GL_Gan1_GLEAN_10004152 | Salmonella enterica | Reduced virulence | 778.79 | 1803.54 | 1349.715 | 1374.265 | 1891.665 |
| GL_Gan1_GLEAN_10003295 | Ustilago maydis | Unaffected pathogenicity | 29.77 | 20.155 | 29.245 | 25.225 | 26.61 |
| GL_Gan1_GLEAN_10001129 | Magnaporthe grisea | Unaffected pathogenicity | 34.875 | 9.515 | 15.45 | 14.99 | 14.37 |
| GL_Gan1_GLEAN_10001130 | Salmonella enterica | Unaffected pathogenicity | 14.61 | 1.21 | 2.34 | 3.175 | 2.5 |
| GL_Gan1_GLEAN_10001140 | Magnaporthe grisea | Reduced virulence | 27.3 | 6.97 | 17.105 | 10.515 | 10.635 |
| GL_Gan1_GLEAN_10001145 | Cochliobolus heterostrophus | Reduced virulence | 4.875 | 0.255 | 0.71 | 0.305 | 0.635 |
| GL_Gan1_GLEAN_10001146 | Magnaporthe grisea | Effector (plant avirulence determinant) | 12.625 | 14.855 | 9.07 | 9.32 | 9.01 |
| GL_Gan1_GLEAN_10000323 | Candida albicans | Reduced virulence | 13.545 | 13.725 | 12.49 | 15.035 | 16.575 |
| GL_Gan1_GLEAN_10005197 | Stagonospora nodorum | Unaffected pathogenicity | 15.31 | 5.43 | 3.485 | 3.52 | 3.03 |
| GL_Gan1_GLEAN_10005212 | Ustilago maydis | Loss of pathogenicity | 37.765 | 9.31 | 10.03 | 8.55 | 11.39 |
| GL_Gan1_GLEAN_10005213 | Fusarium oxysporum | Loss of pathogenicity | 7.465 | 2.98 | 4.665 | 3.395 | 2.575 |
| GL_Gan1_GLEAN_10005221 | Phytophthora infestans | Reduced virulence | 25.545 | 49.52 | 31.12 | 29.405 | 49.805 |
| GL_Gan1_GLEAN_10005222 | Colletotrichum gloeosporioides | Unaffected pathogenicity | 43.88 | 29.68 | 32.235 | 44.12 | 37.67 |
| GL_Gan1_GLEAN_10005227 | Cercospora nicotianae | Reduced virulence | 3.94 | 0.33 | 0.4 | 0.69 | 0.21 |
| GL_Gan1_GLEAN_10001598 | Salmonella enterica | Reduced virulence | 89.835 | 208.915 | 157.13 | 163.07 | 203.955 |
| GL_Gan1_GLEAN_10005246 | Botrytis cinerea | Unaffected pathogenicity | 473.47 | 1733.495 | 1166.98 | 2277.66 | 1414.585 |
| GL_Gan1_GLEAN_10005248 | Candida albicans | Reduced virulence | 7.965 | 23.095 | 21.54 | 33.97 | 24.115 |
| GL_Gan1_GLEAN_10005251 | Cercospora nicotianae | Reduced virulence | 2.55 | 0.565 | 0.49 | 0.825 | 0.505 |
| GL_Gan1_GLEAN_10005262 | Magnaporthe grisea | Reduced virulence | 331.35 | 46.785 | 69.04 | 66.505 | 28.475 |
| GL_Gan1_GLEAN_10000280 | Septoria lycopersici | Unaffected pathogenicity | 3.835 | 4.225 | 4.06 | 3.05 | 3.95 |
| GL_Gan1_GLEAN_10004127 | Septoria lycopersici | Unaffected pathogenicity | 25.915 | 20.73 | 21.29 | 25.095 | 26.455 |
| GL_Gan1_GLEAN_10005282 | Colletotrichum lindemuthianum | Reduced virulence | 220.8 | 209.985 | 207.145 | 188.615 | 186.95 |
| GL_Gan1_GLEAN_10008423 | Stagonospora nodorum | Unaffected pathogenicity | 7.525 | 152.26 | 129.995 | 138.105 | 131.23 |
| GL_Gan1_GLEAN_10004468 | Stagonospora nodorum | Unaffected pathogenicity | 1709.905 | 1212.165 | 2171.195 | 1139.635 | 1132.48 |
| GL_Gan1_GLEAN_10005314 | Magnaporthe grisea | Reduced virulence | 52.285 | 74.705 | 62.44 | 67.11 | 44.2 |
| GL_Gan1_GLEAN_10002117 | Cercospora nicotianae | Reduced virulence | 0.96 | 0.215 | 1.01 | 0.55 | 0.46 |
| GL_Gan1_GLEAN_10002118 | Stagonospora nodorum | Unaffected pathogenicity | 10.125 | 13.18 | 16.295 | 9.595 | 12.35 |
| GL_Gan1_GLEAN_10002119 | Magnaporthe grisea | Reduced virulence | 764.605 | 309.415 | 446.165 | 472.21 | 357.535 |
| GL_Gan1_GLEAN_10002120 | Botrytis cinerea | Reduced virulence | 108.22 | 47.335 | 53.72 | 55.22 | 47.455 |
| GL_Gan1_GLEAN_10002141 | Fusarium oxysporum | Unaffected pathogenicity | 51.055 | 9.86 | 12.585 | 11.735 | 13.215 |
| GL_Gan1_GLEAN_10002151 | Ustilago maydis | Loss of pathogenicity | 70.945 | 44.23 | 75.45 | 77.865 | 55.08 |
| GL_Gan1_GLEAN_10002155 | Colletotrichum lagenarium | Loss of pathogenicity | 848.285 | 233.465 | 271.97 | 247.865 | 214.155 |
| GL_Gan1_GLEAN_10002157 | Candida albicans | Loss of pathogenicity | 35.045 | 11 | 25.8 | 19.555 | 21.38 |
| GL_Gan1_GLEAN_10004485 | Stagonospora nodorum | Unaffected pathogenicity | 13.025 | 13.68 | 13.795 | 13.28 | 16.215 |
| GL_Gan1_GLEAN_10002165 | Botrytis cinerea | Unaffected pathogenicity | 0.805 | 1.065 | 0.755 | 0.83 | 0.7 |
| GL_Gan1_GLEAN_10002169 | Fusarium oxysporum | Loss of pathogenicity | 13.905 | 6.76 | 4.215 | 4.215 | 6.965 |
| GL_Gan1_GLEAN_10000124 | Cercospora nicotianae | Reduced virulence | 17.335 | 51.53 | 66.295 | 24.285 | 43.67 |
| GL_Gan1_GLEAN_10000125 | Botrytis cinerea | Reduced virulence | 96.955 | 21.05 | 31.89 | 18.385 | 16.005 |
| GL_Gan1_GLEAN_10000348 | Candida albicans | Reduced virulence | 12.255 | 6.34 | 6.285 | 5.845 | 7.72 |
| GL_Gan1_GLEAN_10000243 | Cochliobolus carbonum | Loss of pathogenicity | 0.265 | 0.17 | 0.145 | 0.055 | 0 |
| GL_Gan1_GLEAN_10000244 | Cercospora nicotianae | Reduced virulence | 2.98 | 10.525 | 15.225 | 16.38 | 10.88 |
| GL_Gan1_GLEAN_10000948 | Colletotrichum lagenarium | Loss of pathogenicity | 15.055 | 8.69 | 12.065 | 10.29 | 10.96 |
| GL_Gan1_GLEAN_10000954 | Botrytis cinerea | Unaffected pathogenicity | 0.77 | 1.36 | 3.745 | 2.54 | 2.315 |
| GL_Gan1_GLEAN_10000962 | Fusarium oxysporum | Unaffected pathogenicity | 159.445 | 79.995 | 66.765 | 39.64 | 66.765 |
| GL_Gan1_GLEAN_10000963 | Fusarium oxysporum | Unaffected pathogenicity | 1.615 | 10.225 | 11.535 | 13.495 | 10.21 |
| GL_Gan1_GLEAN_10006747 | Stagonospora nodorum | Reduced virulence | 113.86 | 20.795 | 12.13 | 16.675 | 24.555 |
| GL_Gan1_GLEAN_10008157 | Stagonospora nodorum | Reduced virulence | 82.905 | 71.75 | 52.69 | 61.195 | 80.47 |
| GL_Gan1_GLEAN_10009198 | Stagonospora nodorum | Unaffected pathogenicity | 0 | 0.885 | 1.075 | 1.375 | 2.285 |
| GL_Gan1_GLEAN_10004576 | Botrytis cinerea | Reduced virulence | 9.245 | 5.93 | 7.42 | 6.26 | 12.19 |
| GL_Gan1_GLEAN_10004583 | Aspergillus fumigatus | Loss of pathogenicity | 90.46 | 84.74 | 108.255 | 108.29 | 105.885 |
| GL_Gan1_GLEAN_10009199 | Stagonospora nodorum | Unaffected pathogenicity | 3.145 | 119.025 | 29.045 | 128.84 | 201.59 |
| GL_Gan1_GLEAN_10004589 | Magnaporthe grisea | Reduced virulence | 0 | 0 | 0.05 | 0.205 | 0 |
| GL_Gan1_GLEAN_10004606 | Botrytis cinerea | Unaffected pathogenicity | 25.455 | 13.125 | 14.64 | 7.1 | 23.205 |
| GL_Gan1_GLEAN_10008753 | Stagonospora nodorum | Unaffected pathogenicity | 0 | 1.055 | 2.97 | 1.83 | 1.405 |
| GL_Gan1_GLEAN_10004611 | Botrytis cinerea | Unaffected pathogenicity | 6.175 | 1.81 | 5.92 | 3.775 | 2.725 |
| GL_Gan1_GLEAN_10004632 | Magnaporthe grisea | Effector (plant avirulence determinant) | 2.475 | 11.985 | 4.445 | 3.155 | 11.75 |
| GL_Gan1_GLEAN_10004636 | Magnaporthe grisea | Loss of pathogenicity | 17.065 | 14.505 | 12.51 | 15.635 | 16.49 |
| GL_Gan1_GLEAN_10004645 | Candida albicans | Reduced virulence | 125.825 | 3.545 | 13.315 | 18.86 | 3.41 |
| GL_Gan1_GLEAN_10003451 | Botrytis cinerea | Reduced virulence | 3.475 | 14.41 | 6.355 | 6.53 | 20.09 |
| GL_Gan1_GLEAN_10003454 | Candida albicans | Reduced virulence | 678.83 | 1599.16 | 1585.175 | 1738.965 | 1163.875 |
| GL_Gan1_GLEAN_10003459 | Magnaporthe grisea | Loss of pathogenicity | 49.025 | 67.435 | 54.17 | 49.64 | 67.385 |
| GL_Gan1_GLEAN_10003461 | Botrytis cinerea | Reduced virulence | 0.81 | 0 | 0.045 | 0.14 | 0 |
| GL_Gan1_GLEAN_10003467 | Magnaporthe grisea | Reduced virulence | 23.7 | 3.835 | 4.63 | 3.915 | 1.1 |
| GL_Gan1_GLEAN_10003488 | Magnaporthe grisea | Reduced virulence | 10.38 | 32.12 | 36.27 | 36.32 | 36.325 |
| GL_Gan1_GLEAN_10003507 | Fusarium oxysporum | Reduced virulence | 7.025 | 44.135 | 34.675 | 33.495 | 46.065 |
| GL_Gan1_GLEAN_10003522 | Candida albicans | Reduced virulence | 6.4 | 18.23 | 31.685 | 33.52 | 25.93 |
| GL_Gan1_GLEAN_10003539 | Magnaporthe grisea | Reduced virulence | 2.96 | 0.975 | 2.9 | 4.27 | 1.215 |
| GL_Gan1_GLEAN_10000159 | Ustilago maydis | Unaffected pathogenicity | 0.69 | 24.045 | 405 | 31.26 | 48.55 |
| GL_Gan1_GLEAN_10000162 | Fusarium oxysporum | Loss of pathogenicity | 3.065 | 1.72 | 3.78 | 2.905 | 1.905 |
| GL_Gan1_GLEAN_10002485 | Stagonospora nodorum | Unaffected pathogenicity | 145.265 | 9.98 | 5.425 | 7.28 | 11.865 |
| GL_Gan1_GLEAN_10000603 | Magnaporthe grisea | Reduced virulence | 0 | 0 | 0 | 0 | 0.31 |
| GL_Gan1_GLEAN_10000611 | Cochliobolus heterostrophus | Unaffected pathogenicity | 417.435 | 82.015 | 103.685 | 125.51 | 64.135 |
| GL_Gan1_GLEAN_10000612 | Candida albicans | Unaffected pathogenicity | 336.59 | 230.47 | 222.555 | 258.065 | 274.45 |
| GL_Gan1_GLEAN_10000613 | Cochliobolus carbonum | Lethal | 11.96 | 45.815 | 70.83 | 65.6 | 50.31 |
| GL_Gan1_GLEAN_10000615 | Magnaporthe grisea | Loss of pathogenicity | 2.775 | 13.605 | 23.115 | 29.865 | 16.08 |
| GL_Gan1_GLEAN_10000616 | Botrytis cinerea | Unaffected pathogenicity | 1.645 | 3.975 | 6.24 | 9.445 | 9.04 |
| GL_Gan1_GLEAN_10002239 | Botrytis cinerea | Unaffected pathogenicity | 0 | 0 | 1.14 | 1.035 | 1.175 |
| GL_Gan1_GLEAN_10002240 | Botrytis cinerea | Unaffected pathogenicity | 8.69 | 0.84 | 6.09 | 2.945 | 2.245 |
| GL_Gan1_GLEAN_10002528 | Stagonospora nodorum | Unaffected pathogenicity | 0 | 0.73 | 0.825 | 0.595 | 0.44 |
| GL_Gan1_GLEAN_10002246 | Candida albicans | Reduced virulence | 20.895 | 18.515 | 24.41 | 19.89 | 23.685 |
| GL_Gan1_GLEAN_10002262 | Botrytis cinerea | Unaffected pathogenicity | 0.665 | 13.31 | 2.115 | 3.54 | 7.035 |
| GL_Gan1_GLEAN_10002264 | Mycosphaerella graminicola | Resistant to chemical | 0 | 0.19 | 0.605 | 1.765 | 1.49 |
| GL_Gan1_GLEAN_10002278 | Candida glabrata | Increased virulence (Hypervirulence) | 35.275 | 14.54 | 16.035 | 14.825 | 16.165 |
| GL_Gan1_GLEAN_10002291 | Magnaporthe grisea | Unaffected pathogenicity | 56.36 | 109.13 | 132.02 | 124.62 | 158.25 |
| GL_Gan1_GLEAN_10002293 | Magnaporthe grisea | Loss of pathogenicity | 10 | 14.125 | 19.35 | 22.125 | 25.615 |
| GL_Gan1_GLEAN_10007568 | Colletotrichum gloeosporioides | Reduced virulence | 49.175 | 31.16 | 31.86 | 27.96 | 37.955 |
| GL_Gan1_GLEAN_10007578 | Magnaporthe grisea | Reduced virulence | 26.33 | 45.885 | 40.99 | 44.085 | 51.255 |
| GL_Gan1_GLEAN_10007901 | Stagonospora nodorum | Unaffected pathogenicity | 6.945 | 1.7 | 7.655 | 5.68 | 1.67 |
| GL_Gan1_GLEAN_10007596 | Botrytis cinerea | Reduced virulence | 11.115 | 4.22 | 3 | 2.245 | 3.27 |
| GL_Gan1_GLEAN_10007611 | Magnaporthe grisea | Reduced virulence | 36.23 | 34.48 | 49.72 | 40.875 | 57.645 |
| GL_Gan1_GLEAN_10007950 | Stagonospora nodorum | Unaffected pathogenicity | 0 | 9.38 | 1.295 | 1.125 | 10.085 |
| GL_Gan1_GLEAN_10007644 | Cochliobolus carbonum | Reduced virulence | 82.48 | 54.81 | 56.01 | 62.77 | 65.01 |
| GL_Gan1_GLEAN_10007653 | Fusarium oxysporum | Reduced virulence | 70.525 | 73.52 | 103.12 | 89.06 | 91.585 |
| GL_Gan1_GLEAN_10007657 | Trichoderma virens | Reduced virulence | 1.06 | 1.1 | 1.355 | 1.61 | 0.78 |
| GL_Gan1_GLEAN_10007673 | Candida albicans | Reduced virulence | 52.145 | 36.26 | 35.81 | 39.21 | 45.68 |
| GL_Gan1_GLEAN_10007680 | Magnaporthe grisea | Reduced virulence | 10.84 | 82.075 | 11.34 | 8.68 | 109.82 |
| GL_Gan1_GLEAN_10007682 | Candida albicans | Reduced virulence | 27.155 | 30.735 | 30.17 | 27.285 | 44.48 |
| GL_Gan1_GLEAN_10007683 | Botrytis cinerea | Unaffected pathogenicity | 21.245 | 94.53 | 11.19 | 14.59 | 81.26 |
| GL_Gan1_GLEAN_10007685 | Magnaporthe grisea | Effector (plant avirulence determinant) | 96.055 | 188.65 | 170.845 | 193.595 | 193.535 |
| GL_Gan1_GLEAN_10007693 | Botrytis cinerea | Unaffected pathogenicity | 6.59 | 9.355 | 7.945 | 10.485 | 27.99 |
| GL_Gan1_GLEAN_10007957 | Stagonospora nodorum | Reduced virulence | 2 | 4.415 | 2.985 | 2.55 | 5.685 |
| GL_Gan1_GLEAN_10007719 | Magnaporthe grisea | Loss of pathogenicity | 28.065 | 5.03 | 5.7 | 7.11 | 3.13 |
| GL_Gan1_GLEAN_10007723 | Cochliobolus carbonum | Reduced virulence | 188.64 | 96.35 | 108.05 | 112.185 | 98.47 |
| GL_Gan1_GLEAN_10007726 | Botrytis cinerea | Unaffected pathogenicity | 0.755 | 2.315 | 0.92 | 1.205 | 3.8 |
| GL_Gan1_GLEAN_10007727 | Colletotrichum lindemuthianum | Reduced virulence | 92.515 | 32.22 | 46.575 | 42.085 | 29.32 |
| GL_Gan1_GLEAN_10007736 | Botrytis cinerea | Unaffected pathogenicity | 95.98 | 15.03 | 14.15 | 19.15 | 9.79 |
| GL_Gan1_GLEAN_10007738 | Mycosphaerella graminicola | unaffected pathogenicty | 99.97 | 8.765 | 15.41 | 17.68 | 10.02 |
| GL_Gan1_GLEAN_10007739 | Cercospora nicotianae | Reduced virulence | 0 | 20.91 | 8.2 | 7.435 | 12.995 |
| GL_Gan1_GLEAN_10001970 | Stagonospora nodorum | Reduced virulence | 105.505 | 225.565 | 220.845 | 212.665 | 235.265 |
| GL_Gan1_GLEAN_10007754 | Cochliobolus heterostrophus | Unaffected pathogenicity | 88.9 | 47.045 | 45.67 | 39.9 | 46.735 |
| GL_Gan1_GLEAN_10007755 | Ustilago maydis | Unaffected pathogenicity | 27.015 | 7.94 | 10.64 | 4.765 | 8.915 |
| GL_Gan1_GLEAN_10007759 | Cercospora nicotianae | Reduced virulence | 0.995 | 7.425 | 8.33 | 5.255 | 8.855 |
| GL_Gan1_GLEAN_10007771 | Fusarium oxysporum | Unaffected pathogenicity | 0 | 23.01 | 10.03 | 19.03 | 27.35 |
| GL_Gan1_GLEAN_10007772 | Botrytis elliptica | Unaffected pathogenicity | 83.615 | 473.66 | 428.47 | 439.095 | 549.455 |
| GL_Gan1_GLEAN_10007774 | Botrytis cinerea | Reduced virulence | 0.3 | 6.61 | 10.785 | 14.095 | 13.175 |
| GL_Gan1_GLEAN_10007775 | Magnaporthe grisea | Reduced virulence | 14.48 | 7.68 | 8.73 | 7.735 | 8.785 |
| GL_Gan1_GLEAN_10007777 | Botrytis cinerea | Unaffected pathogenicity | 20.31 | 6.415 | 4.995 | 4.525 | 5.675 |
| GL_Gan1_GLEAN_10007785 | Magnaporthe grisea | Reduced virulence | 36.655 | 19.645 | 29.465 | 22.925 | 23.755 |
| GL_Gan1_GLEAN_10007788 | Botrytis cinerea | Unaffected pathogenicity | 3.39 | 2.785 | 1.925 | 2.44 | 1.98 |
| GL_Gan1_GLEAN_10007789 | Candida albicans | Reduced virulence | 25.35 | 7.015 | 16.91 | 28.56 | 9.52 |
| GL_Gan1_GLEAN_10007791 | Cercospora nicotianae | Reduced virulence | 5.105 | 37.12 | 73.825 | 74.985 | 36.67 |
| GL_Gan1_GLEAN_10007793 | Magnaporthe grisea | Reduced virulence | 1.585 | 3.49 | 3.095 | 4.055 | 4.965 |
| GL_Gan1_GLEAN_10006001 | Magnaporthe grisea | Loss of pathogenicity | 20.43 | 36.295 | 27.625 | 23.445 | 31.305 |
| GL_Gan1_GLEAN_10006007 | Candida albicans | Reduced virulence | 114.305 | 34.635 | 55.83 | 62.39 | 50.145 |
| GL_Gan1_GLEAN_10006015 | Botrytis cinerea | Reduced virulence | 83.81 | 141.09 | 87.995 | 69.27 | 145.725 |
| GL_Gan1_GLEAN_10006021 | Magnaporthe grisea | Reduced virulence | 25.13 | 208.905 | 75.07 | 48.57 | 248.04 |
| GL_Gan1_GLEAN_10006029 | Candida albicans | Reduced virulence | 2.955 | 5.5 | 2.42 | 0.71 | 1.7 |
| GL_Gan1_GLEAN_10006037 | Botrytis cinerea | Reduced virulence | 2.155 | 95.53 | 84.95 | 84.52 | 90.91 |
| GL_Gan1_GLEAN_10006046 | Colletotrichum lagenarium | Reduced virulence | 0.805 | 3.585 | 0.385 | 0.21 | 2.63 |
| GL_Gan1_GLEAN_10006050 | Colletotrichum lagenarium | Reduced virulence | 117.73 | 46.41 | 124.405 | 65.09 | 55.72 |
| GL_Gan1_GLEAN_10006051 | Colletotrichum lagenarium | Reduced virulence | 92.265 | 26.305 | 87.355 | 54.245 | 26.865 |
| GL_Gan1_GLEAN_10001977 | Stagonospora nodorum | Unaffected pathogenicity | 0 | 4.61 | 6.175 | 6.97 | 5.12 |
| GL_Gan1_GLEAN_10006056 | Phytophthora infestans | Reduced virulence | 12.265 | 17.88 | 16.86 | 15.225 | 13.76 |
| GL_Gan1_GLEAN_10006057 | Magnaporthe grisea | Loss of pathogenicity | 17.66 | 17.355 | 19.215 | 17.43 | 14.765 |
| GL_Gan1_GLEAN_10006058 | Candida albicans | Reduced virulence | 44.73 | 265.36 | 201.925 | 248.74 | 265.83 |
| GL_Gan1_GLEAN_10006059 | Cochliobolus heterostrophus | Loss of pathogenicity | 84.085 | 37.225 | 83.275 | 104.265 | 57.535 |
| GL_Gan1_GLEAN_10006060 | Mycosphaerella graminicola | Resistant to chemical | 0.705 | 0.62 | 1.29 | 2.18 | 0.59 |
| GL_Gan1_GLEAN_10006062 | Candida albicans | Reduced virulence | 67.39 | 86.555 | 86.49 | 95.415 | 76.43 |
| GL_Gan1_GLEAN_10006065 | Candida albicans | Loss of pathogenicity | 74.015 | 32.415 | 39.085 | 37.395 | 42.075 |
| GL_Gan1_GLEAN_10006089 | Aspergillus fumigatus | Reduced virulence | 79.5 | 8.36 | 41.76 | 15.62 | 32.495 |
| GL_Gan1_GLEAN_10006093 | Ustilago maydis | Loss of pathogenicity | 84.73 | 21.11 | 35.06 | 29.08 | 33.345 |
| GL_Gan1_GLEAN_10006100 | Ustilago maydis | Loss of pathogenicity | 9.32 | 52.385 | 23.04 | 22.165 | 35.285 |
| GL_Gan1_GLEAN_10006105 | Aspergillus nidulans | Reduced virulence | 129.065 | 106.01 | 78.28 | 82.06 | 157.37 |
| GL_Gan1_GLEAN_10006106 | Candida albicans | Loss of pathogenicity | 268.265 | 24.03 | 34.575 | 35.075 | 34.19 |
| GL_Gan1_GLEAN_10006109 | Ustilago maydis | Loss of pathogenicity | 9.925 | 15.64 | 14.285 | 13.51 | 19.93 |
| GL_Gan1_GLEAN_10006115 | Magnaporthe grisea | Reduced virulence | 139.905 | 39.445 | 45.36 | 48.24 | 28.79 |
| GL_Gan1_GLEAN_10006116 | Fusarium oxysporum | Reduced virulence | 378.14 | 179.235 | 414.33 | 360.945 | 291.835 |
| GL_Gan1_GLEAN_10006119 | Candida albicans | Reduced virulence | 106.125 | 24.455 | 46.615 | 27.795 | 33.93 |
| GL_Gan1_GLEAN_10006123 | Colletotrichum lindemuthianum | Reduced virulence | 766.805 | 869.285 | 717.64 | 820.455 | 760.37 |
| GL_Gan1_GLEAN_10006132 | Cochliobolus carbonum | Reduced virulence | 1.905 | 2.99 | 2.29 | 2.83 | 4.69 |
| GL_Gan1_GLEAN_10006135 | Botrytis cinerea | Reduced virulence | 16.02 | 20.055 | 18.21 | 15.61 | 26.365 |
| GL_Gan1_GLEAN_10006137 | Aspergillus fumigatus | Reduced virulence | 1.82 | 3.325 | 3.845 | 2.405 | 5.17 |
| GL_Gan1_GLEAN_10006141 | Candida albicans | Reduced virulence | 16.77 | 81.055 | 22.185 | 64.435 | 153.125 |
| GL_Gan1_GLEAN_10000888 | Candida albicans | Reduced virulence | 5.115 | 29.685 | 30.325 | 29.68 | 31.495 |
| GL_Gan1_GLEAN_10000896 | Magnaporthe grisea | Effector (plant avirulence determinant) | 2.68 | 5.15 | 4.685 | 4.05 | 4.21 |
| GL_Gan1_GLEAN_10000899 | Botrytis cinerea | Reduced virulence | 7.205 | 1.105 | 1.815 | 1.985 | 0.725 |
| GL_Gan1_GLEAN_10000901 | Botrytis cinerea | Unaffected pathogenicity | 0.74 | 1.14 | 2.205 | 2.435 | 1.02 |
| GL_Gan1_GLEAN_10007214 | Stagonospora nodorum | Unaffected pathogenicity | 0 | 0.435 | 0.72 | 0.43 | 0 |
| GL_Gan1_GLEAN_10000203 | Cochliobolus carbonum | Reduced virulence | 1.955 | 1.405 | 2.575 | 1.86 | 1.515 |
| GL_Gan1_GLEAN_10000208 | Botrytis cinerea | Reduced virulence | 20.485 | 33.085 | 31.765 | 24.12 | 28.63 |
| GL_Gan1_GLEAN_10002532 | Magnaporthe grisea | Unaffected pathogenicity | 2.675 | 9.005 | 14.72 | 13.61 | 10.47 |
| GL_Gan1_GLEAN_10002538 | Candida albicans | Reduced virulence | 0 | 10.085 | 4.685 | 3.725 | 11.11 |
| GL_Gan1_GLEAN_10002540 | Aspergillus fumigatus | Reduced virulence | 58.125 | 48.11 | 62.62 | 42.28 | 68.34 |
| GL_Gan1_GLEAN_10002541 | Colletotrichum lindemuthianum | Loss of pathogenicity | 0 | 4.87 | 4.165 | 3.975 | 5.03 |
| GL_Gan1_GLEAN_10007215 | Stagonospora nodorum | Unaffected pathogenicity | 570.57 | 304.395 | 83.515 | 101.905 | 86.95 |
| GL_Gan1_GLEAN_10002553 | Ustilago maydis | Reduced virulence | 12.705 | 50.545 | 38.435 | 40.265 | 60.165 |
| GL_Gan1_GLEAN_10004716 | Stagonospora nodorum | Unaffected pathogenicity | 4.585 | 1.68 | 2.175 | 3.895 | 1.215 |
| GL_Gan1_GLEAN_10002568 | Botrytis cinerea | Unaffected pathogenicity | 0 | 25.57 | 4.475 | 4.315 | 23.74 |
| GL_Gan1_GLEAN_10002570 | Candida albicans | Reduced virulence | 0 | 0.175 | 0.5 | 0.505 | 0.25 |
| GL_Gan1_GLEAN_10002577 | Burkholderia glumae | Resistant to chemical | 30.7 | 28.735 | 26.62 | 29.595 | 38.345 |
| GL_Gan1_GLEAN_10002437 | Stagonospora nodorum | Unaffected pathogenicity | 46.71 | 16.42 | 11.53 | 17.74 | 26.8 |
| GL_Gan1_GLEAN_10002588 | Fusarium oxysporum | Unaffected pathogenicity | 2.055 | 12.44 | 5.03 | 30.165 | 17.47 |
| GL_Gan1_GLEAN_10002590 | Saccharomyces cerevisiae | Reduced virulence | 0.715 | 3.62 | 3.685 | 3.11 | 2.89 |
| GL_Gan1_GLEAN_10002594 | Ustilago maydis | Unaffected pathogenicity | 151.71 | 440.445 | 252.045 | 258.94 | 393.875 |
| GL_Gan1_GLEAN_10003326 | Stagonospora nodorum | Loss of pathogenicity | 384.07 | 368.205 | 383.695 | 370.235 | 451.35 |
| GL_Gan1_GLEAN_10002930 | Botrytis cinerea | Reduced virulence | 147.065 | 25.895 | 13.735 | 13.44 | 17.535 |
| GL_Gan1_GLEAN_10002935 | Ustilago maydis | Reduced virulence | 18.085 | 91.58 | 79.6 | 95.99 | 86.985 |
| GL_Gan1_GLEAN_10002938 | Fusarium oxysporum | Loss of pathogenicity | 23.595 | 5.075 | 5.19 | 5.73 | 6.08 |
| GL_Gan1_GLEAN_10003345 | Stagonospora nodorum | Reduced virulence | 14.93 | 12.5 | 7.74 | 7.94 | 9.725 |
| GL_Gan1_GLEAN_10002950 | Candida albicans | Reduced virulence | 7.935 | 21.195 | 18.69 | 18.91 | 28.04 |
| GL_Gan1_GLEAN_10002958 | Magnaporthe grisea | Reduced virulence | 0 | 0 | 0 | 0.055 | 0 |
| GL_Gan1_GLEAN_10002959 | Fusarium oxysporum | Unaffected pathogenicity | 2.92 | 2.565 | 2.62 | 2.47 | 3.1 |
| GL_Gan1_GLEAN_10002967 | Fusarium oxysporum | Unaffected pathogenicity | 36.36 | 31.86 | 42.425 | 47.88 | 40.5 |
| GL_Gan1_GLEAN_10004843 | Stagonospora nodorum | Unaffected pathogenicity | 11.605 | 27.38 | 12.71 | 32.01 | 27.595 |
| GL_Gan1_GLEAN_10002988 | Saccharomyces cerevisiae | Reduced virulence | 35.475 | 87.94 | 57.04 | 46.635 | 71.29 |
| GL_Gan1_GLEAN_10005447 | Candida albicans | Reduced virulence | 0.72 | 6.46 | 2.8 | 4.895 | 7.83 |
| GL_Gan1_GLEAN_10005448 | Mycosphaerella graminicola | Resistant to chemical | 0 | 1.22 | 0.24 | 0.25 | 0.855 |
| GL_Gan1_GLEAN_10005452 | Colletotrichum gloeosporioides | Reduced virulence | 8.74 | 3.515 | 2.555 | 2.695 | 3.755 |
| GL_Gan1_GLEAN_10005459 | Cercospora nicotianae | Reduced virulence | 0 | 0.255 | 0.875 | 0.805 | 0 |
| GL_Gan1_GLEAN_10005464 | Candida albicans | Reduced virulence | 0.84 | 11.48 | 16.48 | 20.365 | 14.05 |
| GL_Gan1_GLEAN_10005472 | Candida albicans | Reduced virulence | 767.365 | 7405 | 593.555 | 699.415 | 625.545 |
| GL_Gan1_GLEAN_10005481 | Cryptococcus neoformans | Reduced virulence | 40.825 | 38.18 | 29.035 | 29.02 | 42.215 |
| GL_Gan1_GLEAN_10005490 | Candida albicans | Reduced virulence | 23.975 | 27.39 | 43.02 | 39.31 | 43.855 |
| GL_Gan1_GLEAN_10002838 | Stagonospora nodorum | Unaffected pathogenicity | 10 | 47.775 | 45.875 | 41.47 | 74.545 |
| GL_Gan1_GLEAN_10005499 | Aspergillus fumigatus | Reduced virulence | 495.905 | 175.83 | 162.855 | 149.965 | 162.265 |
| GL_Gan1_GLEAN_10005525 | Candida albicans | Reduced virulence | 0 | 20.555 | 17.155 | 18.28 | 15.21 |
| GL_Gan1_GLEAN_10005526 | Botrytis cinerea | Reduced virulence | 14.125 | 128.915 | 71.21 | 45.505 | 151.3 |
| GL_Gan1_GLEAN_10005529 | Magnaporthe grisea | Reduced virulence | 22.445 | 9.69 | 9.305 | 9.15 | 11.24 |
| GL_Gan1_GLEAN_10005530 | Magnaporthe grisea | Loss of pathogenicity | 71.47 | 72.575 | 55.215 | 44.96 | 97.285 |
| GL_Gan1_GLEAN_10005532 | Cercospora nicotianae | Reduced virulence | 37.655 | 37.03 | 40.905 | 32.61 | 76.815 |
| GL_Gan1_GLEAN_10006990 | Stagonospora nodorum | Unaffected pathogenicity | 11.63 | 138.045 | 120.75 | 106.16 | 123.945 |
| GL_Gan1_GLEAN_10005550 | Cochliobolus heterostrophus | Unaffected pathogenicity | 341.48 | 114.12 | 118.905 | 115.25 | 150.03 |
| GL_Gan1_GLEAN_10005565 | Colletotrichum lindemuthianum | Loss of pathogenicity | 75.3 | 26.115 | 16.595 | 24.625 | 20.67 |
| GL_Gan1_GLEAN_10005568 | Aspergillus fumigatus | Reduced virulence | 92.25 | 30.09 | 37.09 | 36.67 | 34.275 |
| GL_Gan1_GLEAN_10005575 | Magnaporthe grisea | Reduced virulence | 87.44 | 100.325 | 58.73 | 46.615 | 66.205 |
| GL_Gan1_GLEAN_10005712 | Candida albicans | Reduced virulence | 28.58 | 11.73 | 13.715 | 11.855 | 11.25 |
| GL_Gan1_GLEAN_10003291 | Stagonospora nodorum | Unaffected pathogenicity | 1.935 | 29.425 | 23.25 | 27.895 | 32.595 |
| GL_Gan1_GLEAN_10005715 | Candida albicans | Reduced virulence | 24.265 | 29.86 | 26.385 | 25.98 | 26.35 |
| GL_Gan1_GLEAN_10005716 | Cercospora nicotianae | Reduced virulence | 9.815 | 18.02 | 7.335 | 7.725 | 17.925 |
| GL_Gan1_GLEAN_10005721 | Colletotrichum lindemuthianum | Reduced virulence | 111.805 | 197.075 | 169.47 | 176.25 | 160.58 |
| GL_Gan1_GLEAN_10005723 | Ustilago maydis | Loss of pathogenicity | 11.335 | 11.38 | 15.4 | 15.56 | 21.09 |
| GL_Gan1_GLEAN_10005724 | Candida albicans | Reduced virulence | 222.25 | 133.475 | 177.78 | 194.94 | 205.16 |
| GL_Gan1_GLEAN_10005300 | Stagonospora nodorum | Reduced virulence | 217.73 | 129.22 | 237.375 | 230.08 | 162.58 |
| GL_Gan1_GLEAN_10005740 | Candida albicans | Reduced virulence | 359.6 | 42.805 | 51.35 | 49.505 | 48.765 |
| GL_Gan1_GLEAN_10005752 | Magnaporthe grisea | Reduced virulence | 3.48 | 21.635 | 28.015 | 43.955 | 27.205 |
| GL_Gan1_GLEAN_10005760 | Stagonospora nodorum | Unaffected pathogenicity | 17.115 | 32.725 | 17.425 | 16.065 | 28.34 |
| GL_Gan1_GLEAN_10005764 | Alternaria alternata | Loss of pathogenicity | 13.035 | 19.815 | 27.81 | 30.63 | 21.475 |
| GL_Gan1_GLEAN_10005767 | Salmonella enterica | Reduced virulence | 75.335 | 15.415 | 30.665 | 28.41 | 19.235 |
| GL_Gan1_GLEAN_10005777 | Ustilago maydis | Reduced virulence | 2.82 | 9.365 | 17.185 | 16.995 | 15.365 |
| GL_Gan1_GLEAN_10002159 | Stagonospora nodorum | Reduced virulence | 114.74 | 99.445 | 145.21 | 141.815 | 135.34 |
| GL_Gan1_GLEAN_10005779 | Candida albicans | Loss of pathogenicity | 30.725 | 162.095 | 78.29 | 70.29 | 124.8 |
| GL_Gan1_GLEAN_10005785 | Ustilago maydis | Reduced virulence | 36.97 | 17.34 | 18.81 | 16.345 | 21.26 |
| GL_Gan1_GLEAN_10005787 | Colletotrichum lagenarium | Reduced virulence | 443.32 | 269.92 | 183.94 | 294.76 | 232.09 |
| GL_Gan1_GLEAN_10005788 | Candida albicans | Reduced virulence | 1.385 | 10.13 | 11.39 | 8.23 | 9.615 |
| GL_Gan1_GLEAN_10005794 | Fusarium oxysporum | Loss of pathogenicity | 55.97 | 37.16 | 22.365 | 28.16 | 28.26 |
| GL_Gan1_GLEAN_10005800 | Candida albicans | Reduced virulence | 93.995 | 53.225 | 48.305 | 49.665 | 51.215 |
| GL_Gan1_GLEAN_10007585 | Stagonospora nodorum | Unaffected pathogenicity | 34.74 | 50.875 | 41.58 | 41.9 | 49.07 |
| GL_Gan1_GLEAN_10005812 | Alternaria alternata | Loss of pathogenicity | 70.67 | 89.105 | 85.455 | 95.265 | 100.82 |
| GL_Gan1_GLEAN_10005839 | Candida albicans | Reduced virulence | 10.43 | 1.055 | 0.715 | 0.65 | 1.27 |
| GL_Gan1_GLEAN_10005840 | Magnaporthe grisea | Loss of pathogenicity | 12.5 | 2.95 | 4.82 | 3.37 | 3.54 |
| GL_Gan1_GLEAN_10005841 | Stagonospora nodorum | Unaffected pathogenicity | 679.82 | 7.66 | 17.65 | 36.75 | 3.06 |
| GL_Gan1_GLEAN_10005842 | Cochliobolus heterostrophus | Reduced virulence | 58.485 | 22.39 | 16.055 | 16.77 | 23.925 |
| GL_Gan1_GLEAN_10005857 | Magnaporthe grisea | Reduced virulence | 14.09 | 8.115 | 7.135 | 9.155 | 4.185 |
| GL_Gan1_GLEAN_10000051 | Gibberella moniliformis | Unaffected pathogenicity | 4.715 | 6.055 | 14.92 | 5.53 | 6.49 |
| GL_Gan1_GLEAN_10000022 | Aspergillus fumigatus | Reduced virulence | 44.265 | 73.395 | 51.72 | 51.905 | 82.615 |
| GL_Gan1_GLEAN_10000023 | Fusarium graminearum | Reduced virulence | 118.485 | 28.8 | 44.325 | 44.255 | 40.565 |
| GL_Gan1_GLEAN_10000763 | Saccharomyces cerevisiae | Reduced virulence | 76.745 | 172.07 | 99.1 | 90.21 | 138.055 |
| GL_Gan1_GLEAN_10002581 | Stagonospora nodorum | Unaffected pathogenicity | 0 | 2.37 | 7.485 | 5.99 | 3.645 |
| GL_Gan1_GLEAN_10000774 | Botrytis cinerea | Unaffected pathogenicity | 15.22 | 22.385 | 20.725 | 19.405 | 22.54 |
| GL_Gan1_GLEAN_10000777 | Candida albicans | Reduced virulence | 64.91 | 42.35 | 46.045 | 55.12 | 40.7 |
| GL_Gan1_GLEAN_10000779 | Mycosphaerella graminicola | Unaffected pathogenicity | 47.485 | 32.37 | 18.46 | 17.34 | 35.48 |
| GL_Gan1_GLEAN_10000782 | Candida albicans | Reduced virulence | 28.215 | 54.355 | 13.975 | 18.435 | 17.245 |
| GL_Gan1_GLEAN_10000784 | Fusarium oxysporum | Loss of pathogenicity | 0.545 | 5.22 | 4.835 | 6.025 | 4.575 |
| GL_Gan1_GLEAN_10003555 | Candida albicans | Reduced virulence | 83.57 | 60.735 | 54.4 | 57.155 | 74.295 |
| GL_Gan1_GLEAN_10003556 | Stagonospora nodorum | Unaffected pathogenicity | 61.285 | 90.355 | 196.555 | 202.315 | 97.2 |
| GL_Gan1_GLEAN_10003559 | Cochliobolus heterostrophus | Reduced virulence | 5.07 | 10.805 | 3.4 | 2.665 | 9.425 |
| GL_Gan1_GLEAN_10003565 | Candida albicans | Reduced virulence | 42.805 | 68.23 | 67.705 | 91.275 | 59.835 |
| GL_Gan1_GLEAN_10003572 | Botrytis cinerea | Reduced virulence | 1.11 | 6.32 | 3.77 | 6.295 | 3.995 |
| GL_Gan1_GLEAN_10002939 | Stagonospora nodorum | Unaffected pathogenicity | 95.375 | 123.61 | 107.41 | 101.06 | 102.38 |
| GL_Gan1_GLEAN_10003578 | Botrytis cinerea | Unaffected pathogenicity | 0.74 | 29.54 | 33.47 | 9.71 | 15.515 |
| GL_Gan1_GLEAN_10003582 | Candida albicans | Loss of pathogenicity | 4.065 | 18.755 | 18.585 | 23.025 | 20.49 |
| GL_Gan1_GLEAN_10003586 | Botrytis cinerea | Unaffected pathogenicity | 46.485 | 26.83 | 29.055 | 29.72 | 27.56 |
| GL_Gan1_GLEAN_10001015 | Stagonospora nodorum | Unaffected pathogenicity | 55.86 | 124.255 | 90.075 | 83.635 | 67.095 |
| GL_Gan1_GLEAN_10003589 | Fusarium oxysporum | Unaffected pathogenicity | 2.185 | 4.98 | 5.925 | 5.46 | 5.175 |
| GL_Gan1_GLEAN_10003594 | Cochliobolus carbonum | Reduced virulence | 8.92 | 0.94 | 3.295 | 4.315 | 1.525 |
| GL_Gan1_GLEAN_10003603 | Botrytis cinerea | Unaffected pathogenicity | 0 | 0.98 | 1.91 | 0.815 | 0.995 |
| GL_Gan1_GLEAN_10003611 | Botrytis cinerea | Unaffected pathogenicity | 0 | 0 | 0.18 | 0 | 0 |
| GL_Gan1_GLEAN_10003613 | Ustilago maydis | Reduced virulence | 174.77 | 111.095 | 94.755 | 98.815 | 103.77 |
| GL_Gan1_GLEAN_10003617 | Aspergillus fumigatus | Reduced virulence | 48.7 | 143.48 | 281.875 | 238.635 | 215.505 |
| GL_Gan1_GLEAN_10003626 | Alternaria alternata | Loss of pathogenicity | 3.44 | 4.555 | 7.01 | 5.34 | 5.05 |
| GL_Gan1_GLEAN_10000333 | Botrytis cinerea | Unaffected pathogenicity | 7.33 | 1.355 | 0.78 | 0.48 | 3.24 |
| GL_Gan1_GLEAN_10009176 | Trichoderma virens | Reduced virulence | 3.84 | 7.995 | 3.21 | 9.925 | 12.235 |
| GL_Gan1_GLEAN_10000338 | Ustilago maydis | Reduced virulence | 8.1 | 76.605 | 127.605 | 91.08 | 119.135 |
| GL_Gan1_GLEAN_10000035 | Claviceps purpurea | Reduced virulence | 89.285 | 46.015 | 23.455 | 26.96 | 36.785 |
| GL_Gan1_GLEAN_10000485 | Botrytis cinerea | Reduced virulence | 17.015 | 22.405 | 25.52 | 21.075 | 28.74 |
| GL_Gan1_GLEAN_10000428 | Cercospora nicotianae | Reduced virulence | 5.2 | 4.005 | 2.24 | 7.89 | 6.235 |
| GL_Gan1_GLEAN_10000431 | Candida albicans | Loss of pathogenicity | 48.435 | 19.4 | 19.05 | 19.86 | 17.845 |
| GL_Gan1_GLEAN_10006492 | Candida albicans | Reduced virulence | 0 | 1.555 | 1.5 | 1.225 | 0.425 |
| GL_Gan1_GLEAN_10006506 | Botrytis cinerea | Unaffected pathogenicity | 2.905 | 0.155 | 0.345 | 0.745 | 0.485 |
| GL_Gan1_GLEAN_10006507 | Candida albicans | Reduced virulence | 216.725 | 371.095 | 436.765 | 423.925 | 418.365 |
| GL_Gan1_GLEAN_10006513 | Candida albicans | Reduced virulence | 14.605 | 26.7 | 31.455 | 34.33 | 31.605 |
| GL_Gan1_GLEAN_10006517 | Candida albicans | Reduced virulence | 90.59 | 41.39 | 35.435 | 38.53 | 35.29 |
| GL_Gan1_GLEAN_10006534 | Magnaporthe grisea | Reduced virulence | 15.955 | 7.35 | 11.255 | 10.69 | 9.93 |
| GL_Gan1_GLEAN_10006536 | Botrytis cinerea | Unaffected pathogenicity | 6.84 | 3.105 | 4.295 | 4.97 | 5.835 |
| GL_Gan1_GLEAN_10006538 | Magnaporthe grisea | Reduced virulence | 58.555 | 42.07 | 43.93 | 44.09 | 55.155 |
| GL_Gan1_GLEAN_10006547 | Botrytis cinerea | Reduced virulence | 10.165 | 21.77 | 18.79 | 10.78 | 30.045 |
| GL_Gan1_GLEAN_10006548 | Cryptococcus neoformans | Loss of pathogenicity | 27.875 | 34.585 | 32.125 | 35.845 | 40.79 |
| GL_Gan1_GLEAN_10006550 | Candida albicans | Reduced virulence | 3.97 | 54.31 | 30.685 | 31.23 | 54.215 |
| GL_Gan1_GLEAN_10001634 | Trichoderma virens | Reduced virulence | 13.555 | 28.88 | 73.615 | 60.415 | 27.975 |
| GL_Gan1_GLEAN_10006553 | Magnaporthe grisea | Effector (plant avirulence determinant) | 17.09 | 19.705 | 17.8 | 21.315 | 16.045 |
| GL_Gan1_GLEAN_10006562 | Magnaporthe grisea | Reduced virulence | 329.75 | 406.27 | 555.825 | 515.94 | 345.415 |
| GL_Gan1_GLEAN_10006565 | Magnaporthe grisea | Reduced virulence | 11.15 | 16.185 | 19.525 | 16.35 | 23.275 |
| GL_Gan1_GLEAN_10006566 | Ustilago maydis | Loss of pathogenicity | 25.785 | 43.995 | 38.42 | 40.215 | 53.03 |
| GL_Gan1_GLEAN_10006568 | Fusarium oxysporum | Loss of pathogenicity | 40.07 | 53.5 | 82.64 | 88.575 | 62.14 |
| GL_Gan1_GLEAN_10006569 | Colletotrichum lindemuthianum | Loss of pathogenicity | 7.045 | 10.055 | 10.25 | 6.615 | 11.96 |
| GL_Gan1_GLEAN_10006570 | Cercospora nicotianae | Reduced virulence | 3.7 | 2.085 | 0.41 | 0.965 | 2.22 |
| GL_Gan1_GLEAN_10006574 | Candida albicans | Reduced virulence | 21.59 | 92.025 | 63.6 | 107.52 | 143.53 |
| GL_Gan1_GLEAN_10006577 | Ustilago maydis | Unaffected pathogenicity | 0 | 2.405 | 1.495 | 2.07 | 3.665 |
| GL_Gan1_GLEAN_10006578 | Magnaporthe grisea | Reduced virulence | 0 | 9.8 | 2.325 | 2.835 | 9.63 |
| GL_Gan1_GLEAN_10006580 | Magnaporthe grisea | Reduced virulence | 3.92 | 12.725 | 9.12 | 12.235 | 12.905 |
| GL_Gan1_GLEAN_10006583 | Magnaporthe grisea | Reduced virulence | 0 | 3.565 | 0.055 | 0.425 | 2.21 |
| GL_Gan1_GLEAN_10006584 | Ustilago maydis | Unaffected pathogenicity | 0 | 15.395 | 0.795 | 4.81 | 14.945 |
| GL_Gan1_GLEAN_10006599 | Botrytis cinerea | Reduced virulence | 0 | 0 | 0.345 | 0.415 | 0.515 |
| GL_Gan1_GLEAN_10006614 | Ustilago maydis | Unaffected pathogenicity | 10.12 | 48.05 | 39.915 | 38.93 | 54.755 |
| GL_Gan1_GLEAN_10006616 | Candida albicans | Reduced virulence | 95.33 | 98.955 | 98.775 | 107.07 | 104.115 |
| GL_Gan1_GLEAN_10006617 | Ustilago maydis | Unaffected pathogenicity | 271.785 | 134.71 | 247.69 | 213.72 | 163.16 |
| GL_Gan1_GLEAN_10006619 | Candida albicans | Reduced virulence | 184.64 | 99.805 | 111.065 | 109.245 | 107.225 |
| GL_Gan1_GLEAN_10006620 | Septoria lycopersici | Unaffected pathogenicity | 16.335 | 16.975 | 16.045 | 16.63 | 16.275 |
| GL_Gan1_GLEAN_10005164 | Trichoderma virens | Reduced virulence | 48.875 | 194.29 | 182.765 | 166.195 | 208.64 |
| GL_Gan1_GLEAN_10006630 | Fusarium oxysporum | Unaffected pathogenicity | 25.69 | 22.855 | 33.025 | 36.35 | 32.265 |
| GL_Gan1_GLEAN_10006220 | Venturia inaequalis | Resistant to chemical | 87.02 | 834.77 | 516.485 | 619.055 | 829.36 |
| GL_Gan1_GLEAN_10000382 | Cryptococcus neoformans | Reduced virulence | 4.91 | 4.225 | 2.18 | 4.275 | 2.205 |
| GL_Gan1_GLEAN_10000391 | Aspergillus nidulans | Resistant to chemical | 29.78 | 50.29 | 79.665 | 55.945 | 68.84 |
| GL_Gan1_GLEAN_10009059 | Wangiella (Exophiala) dermatitidis | Reduced virulence | 35.37 | 17.605 | 19.98 | 17.91 | 25.31 |
| GL_Gan1_GLEAN_10001101 | Magnaporthe grisea | Reduced virulence | 97.415 | 50.135 | 55.365 | 52.82 | 54.7 |
| GL_Gan1_GLEAN_10001103 | Cercospora nicotianae | Reduced virulence | 0 | 0.37 | 0.435 | 0.37 | 0.53 |
| GL_Gan1_GLEAN_10001108 | Candida albicans | Reduced virulence | 119.975 | 123.99 | 92.37 | 98.585 | 130.62 |
| GL_Gan1_GLEAN_10001109 | Ustilago maydis | Reduced virulence | 5.07 | 29.315 | 23.07 | 20.83 | 29.545 |
| GL_Gan1_GLEAN_10001118 | Alternaria alternata | Loss of pathogenicity | 9.595 | 5.145 | 3.425 | 3.73 | 8.875 |
| GL_Gan1_GLEAN_10001120 | Cercospora nicotianae | Reduced virulence | 7.14 | 3.425 | 0.825 | 1.345 | 5.45 |
| GL_Gan1_GLEAN_10001128 | Cercospora nicotianae | Reduced virulence | 0 | 0.265 | 0.495 | 0.315 | 0.57 |
| GL_Gan1_GLEAN_10000683 | Magnaporthe grisea | Reduced virulence | 47.435 | 24.605 | 43.575 | 42.035 | 30.99 |
| GL_Gan1_GLEAN_10000984 | Candida albicans | Loss of pathogenicity | 41.77 | 32.3 | 27.07 | 31.305 | 24.7 |
| GL_Gan1_GLEAN_10001013 | Botrytis cinerea | Unaffected pathogenicity | 30.775 | 59.885 | 97.875 | 74.4 | 96.85 |
| GL_Gan1_GLEAN_10001014 | Epichloe festucae | Wild-type mutualism | 8.715 | 20.29 | 36.89 | 41.68 | 30.965 |
| GL_Gan1_GLEAN_10000337 | Wangiella (Exophiala) dermatitidis | Reduced virulence | 8.045 | 61.04 | 115.47 | 79.005 | 87.06 |
| GL_Gan1_GLEAN_10001016 | Fusarium oxysporum | Unaffected pathogenicity | 21.68 | 11.68 | 9.005 | 12.235 | 7.32 |
| GL_Gan1_GLEAN_10001017 | Colletotrichum lagenarium | Reduced virulence | 58.16 | 59.635 | 27.665 | 30.805 | 45.065 |
| GL_Gan1_GLEAN_10001018 | Pseudomonas syringae | Unaffected pathogenicity | 19.615 | 32.7 | 18.17 | 21.325 | 19.47 |

**(D)** SSCP

| Gene_ID | amid acid length(aa) | Cys number | Cys (%) | Means-GL0d | Means-GL1d | Means-GL3d | Means-GL5d | Means-GL10d |
| --- | --- | --- | --- | --- | --- | --- | --- | --- |
| GL_Gan1_GLEAN_10000206 | 176 | 4 | 2.27% | 55643.68 | 25390.02 | 13643.25 | 20928.77 | 4537.9 |
| GL_Gan1_GLEAN_10000592 | 194 | 4 | 2.06% | 0 | 0 | 0.8 | 1.065 | 0.525 |
| GL_Gan1_GLEAN_10000630 | 147 | 4 | 2.72% | 930.96 | 339.21 | 959.525 | 568.95 | 384.58 |
| GL_Gan1_GLEAN_10000643 | 97 | 7 | 7.22% | 174.945 | 0 | 0.225 | 0.615 | 0.745 |
| GL_Gan1_GLEAN_10001463 | 137 | 7 | 5.11% | 6.335 | 136.59 | 13.57 | 7.975 | 144.935 |
| GL_Gan1_GLEAN_10001506 | 169 | 6 | 3.55% | 0 | 0.56 | 0 | 0 | 0 |
| GL_Gan1_GLEAN_10001595 | 121 | 6 | 4.96% | 0 | 6.42 | 19.55 | 8.815 | 6.81 |
| GL_Gan1_GLEAN_10001661 | 171 | 9 | 5.26% | 31.345 | 66.965 | 357.47 | 143.41 | 89.125 |
| GL_Gan1_GLEAN_10002000 | 129 | 6 | 4.65% | 0 | 2.975 | 2.195 | 2.38 | 1.085 |
| GL_Gan1_GLEAN_10002046 | 176 | 5 | 2.84% | 624.015 | 1955.405 | 1048.27 | 716.445 | 2113.29 |
| GL_Gan1_GLEAN_10002167 | 146 | 8 | 5.48% | 2.96 | 20.355 | 5.295 | 7.505 | 31.395 |
| GL_Gan1_GLEAN_10002215 | 87 | 5 | 5.75% | 0 | 0 | 0 | 0.235 | 0 |
| GL_Gan1_GLEAN_10002671 | 187 | 13 | 6.95% | 9.065 | 203.855 | 503.075 | 312.74 | 204.36 |
| GL_Gan1_GLEAN_10002725 | 95 | 10 | 10.53% | 0 | 749.705 | 142.18 | 143.835 | 1106.15 |
| GL_Gan1_GLEAN_10002903 | 103 | 6 | 5.83% | 0 | 24.05 | 2.13 | 7.4 | 41.77 |
| GL_Gan1_GLEAN_10003121 | 87 | 6 | 6.90% | 69.71 | 47.39 | 405.135 | 159.995 | 52.27 |
| GL_Gan1_GLEAN_10003649 | 199 | 10 | 5.03% | 87.895 | 406.04 | 678.375 | 468.95 | 428.875 |
| GL_Gan1_GLEAN_10003692 | 153 | 8 | 5.23% | 0 | 0 | 0.235 | 0.625 | 0 |
| GL_Gan1_GLEAN_10004066 | 179 | 7 | 3.91% | 14.925 | 5.78 | 8.44 | 6.605 | 5.015 |
| GL_Gan1_GLEAN_10004237 | 183 | 10 | 5.46% | 0 | 20.44 | 56.355 | 42.15 | 22.51 |
| GL_Gan1_GLEAN_10004277 | 114 | 4 | 3.51% | 164.295 | 13.65 | 6.775 | 6.195 | 5.08 |
| GL_Gan1_GLEAN_10004419 | 140 | 8 | 5.71% | 26.52 | 452.755 | 0.4 | 0.84 | 543.21 |
| GL_Gan1_GLEAN_10004533 | 109 | 8 | 7.34% | 17.455 | 0 | 0.91 | 0.91 | 2.44 |
| GL_Gan1_GLEAN_10004623 | 124 | 6 | 4.84% | 3.535 | 13.25 | 23.6 | 17.3 | 10.13 |
| GL_Gan1_GLEAN_10004739 | 155 | 5 | 3.23% | 0 | 2.45 | 1.14 | 0.85 | 0.45 |
| GL_Gan1_GLEAN_10004856 | 131 | 5 | 3.82% | 0 | 1.47 | 4.43 | 0.73 | 4.79 |
| GL_Gan1_GLEAN_10004859 | 170 | 5 | 2.94% | 5.02 | 1.12 | 0.885 | 1.65 | 0.405 |
| GL_Gan1_GLEAN_10004928 | 97 | 6 | 6.19% | 24.52 | 7.125 | 2.2 | 4.085 | 4.045 |
| GL_Gan1_GLEAN_10005483 | 147 | 4 | 2.72% | 58.245 | 103.74 | 48.405 | 49.4 | 61.07 |
| GL_Gan1_GLEAN_10005638 | 191 | 6 | 3.14% | 0 | 15.56 | 19.94 | 29.205 | 9.34 |
| GL_Gan1_GLEAN_10005676 | 118 | 10 | 8.47% | 0 | 0 | 1.54 | 1 | 0.45 |
| GL_Gan1_GLEAN_10006075 | 169 | 8 | 4.73% | 111.09 | 124.78 | 194.215 | 149.36 | 132.425 |
| GL_Gan1_GLEAN_10006848 | 180 | 4 | 2.22% | 59.35 | 247.935 | 189.56 | 222.655 | 222.31 |
| GL_Gan1_GLEAN_10006894 | 91 | 10 | 10.99% | 0 | 1.1 | 0.475 | 0.88 | 0 |
| GL_Gan1_GLEAN_10007495 | 190 | 4 | 2.11% | 945.75 | 462.365 | 586.095 | 493.83 | 331.53 |
| GL_Gan1_GLEAN_10007598 | 182 | 5 | 2.75% | 93.625 | 387.51 | 581.025 | 546.66 | 395.905 |
| GL_Gan1_GLEAN_10007676 | 136 | 6 | 4.41% | 179.615 | 122.495 | 303.515 | 183.895 | 215.615 |
| GL_Gan1_GLEAN_10007717 | 129 | 4 | 3.10% | 74.865 | 253.44 | 7.6 | 9.555 | 332.77 |
| GL_Gan1_GLEAN_10007781 | 95 | 6 | 6.32% | 94.3 | 57.605 | 119.865 | 92.275 | 77.85 |
| GL_Gan1_GLEAN_10007961 | 156 | 8 | 5.13% | 785.98 | 455.18 | 1127.555 | 668.61 | 662.9 |
| GL_Gan1_GLEAN_10008092 | 164 | 8 | 4.88% | 2422.395 | 1413.87 | 2822.645 | 1951.075 | 2121.695 |
| GL_Gan1_GLEAN_10008113 | 123 | 6 | 4.88% | 40.185 | 60.455 | 57.54 | 57.34 | 31.025 |
| GL_Gan1_GLEAN_10008206 | 121 | 5 | 4.13% | 41.965 | 3.185 | 1.745 | 0.965 | 1.59 |
| GL_Gan1_GLEAN_10008288 | 181 | 8 | 4.42% | 55.295 | 10.825 | 21.82 | 12.375 | 12.945 |
| GL_Gan1_GLEAN_10008420 | 97 | 8 | 8.25% | 139.805 | 119.34 | 247.715 | 159.92 | 104.715 |
| GL_Gan1_GLEAN_10008807 | 174 | 4 | 2.30% | 169.85 | 189.705 | 206.22 | 187.3 | 163.915 |
| GL_Gan1_GLEAN_10008854 | 121 | 8 | 6.61% | 640.845 | 125.97 | 518.72 | 286.375 | 240.845 |
| GL_Gan1_GLEAN_10008855 | 117 | 8 | 6.84% | 727.87 | 327.535 | 1209.36 | 774.495 | 500.61 |
| GL_Gan1_GLEAN_10009049 | 180 | 5 | 2.78% | 0 | 0.53 | 0.115 | 0.63 | 0 |
| GL_Gan1_GLEAN_10009234 | 151 | 6 | 3.97% | 112.335 | 86.41 | 133.315 | 81.75 | 67.92 |
| GL_Gan1_GLEAN_10009245 | 195 | 6 | 3.08% | 27.89 | 1349.46 | 32.185 | 6.7 | 1567.5 |

**(E)** Secondary metabolism (SM)-related genes

| Backbone_gene_id | Gene_id | Expression Means | | | | |
| --- | --- | --- | --- | --- | --- | --- |
|  |  | GL0d | GL1d | GL3d | GL5d | GL10d |
| Cluster:1 |  |  |  |  |  |  |
| GL_Gan1_GLEAN_10000043 | GL_Gan1_GLEAN_10000043 | 0 | 0.08 | 0.12 | 0.11 | 0.105 |
| GL_Gan1_GLEAN_10000043 | GL_Gan1_GLEAN_10000172 | 12.995 | 20.78 | 21.955 | 15.165 | 24.755 |
| GL_Gan1_GLEAN_10000043 | GL_Gan1_GLEAN_10000040 | 84.135 | 5.84 | 19.86 | 16.185 | 12.92 |
| Cluster:2 |  |  |  |  |  |  |
| GL_Gan1_GLEAN_10000051 | GL_Gan1_GLEAN_10001148 | 37.24 | 10.14 | 12.845 | 13.925 | 11.025 |
| GL_Gan1_GLEAN_10000051 | GL_Gan1_GLEAN_10000120 | 0 | 0 | 0 | 0 | 0 |
| GL_Gan1_GLEAN_10000051 | GL_Gan1_GLEAN_10000157 | 3.855 | 0.4 | 0.925 | 0.87 | 0.725 |
| GL_Gan1_GLEAN_10000051 | GL_Gan1_GLEAN_10000147 | 48.01 | 96.265 | 73.43 | 60.355 | 83.555 |
| GL_Gan1_GLEAN_10000051 | GL_Gan1_GLEAN_10000595 | 102.27 | 42.035 | 45.66 | 50.885 | 41.815 |
| GL_Gan1_GLEAN_10000051 | GL_Gan1_GLEAN_10000459 | 124.645 | 129.63 | 218.245 | 194.35 | 115.71 |
| GL_Gan1_GLEAN_10000051 | GL_Gan1_GLEAN_10000185 | 261.3 | 240.525 | 297.55 | 321.13 | 222.055 |
| GL_Gan1_GLEAN_10000051 | GL_Gan1_GLEAN_10000113 | 0 | 8.4 | 7.095 | 11.655 | 13.22 |
| GL_Gan1_GLEAN_10000051 | GL_Gan1_GLEAN_10002730 | 14.69 | 133.115 | 380.215 | 369.11 | 83.36 |
| GL_Gan1_GLEAN_10000051 | GL_Gan1_GLEAN_10000051 | 4.715 | 6.055 | 14.92 | 5.53 | 6.49 |
| Cluster:3 |  |  |  |  |  |  |
| GL_Gan1_GLEAN_10000112 | GL_Gan1_GLEAN_10001021 | 0 | 7.01 | 0.715 | 0.465 | 6.96 |
| GL_Gan1_GLEAN_10000112 | GL_Gan1_GLEAN_10003302 | 14.94 | 47.57 | 69.03 | 55.655 | 42.64 |
| GL_Gan1_GLEAN_10000112 | GL_Gan1_GLEAN_10000349 | 224.09 | 51.37 | 52.795 | 66.87 | 40.09 |
| GL_Gan1_GLEAN_10000112 | GL_Gan1_GLEAN_10001947 | 2.29 | 0.335 | 0.665 | 0.475 | 0.58 |
| GL_Gan1_GLEAN_10000112 | GL_Gan1_GLEAN_10000049 | 2.09 | 104.9 | 78.895 | 44.475 | 94.035 |
| GL_Gan1_GLEAN_10000112 | GL_Gan1_GLEAN_10000214 | 3.015 | 15.555 | 13.66 | 19.565 | 15.49 |
| GL_Gan1_GLEAN_10000112 | GL_Gan1_GLEAN_10000184 | 39.355 | 52.445 | 98.135 | 98.785 | 113.955 |
| GL_Gan1_GLEAN_10000112 | GL_Gan1_GLEAN_10004654 | 0 | 0.495 | 0.215 | 0.1 | 0.265 |
| GL_Gan1_GLEAN_10000112 | GL_Gan1_GLEAN_10000368 | 68.085 | 15.21 | 19.89 | 17.745 | 17.765 |
| GL_Gan1_GLEAN_10000112 | GL_Gan1_GLEAN_10000001 | 0 | 0 | 0 | 0 | 0 |
| GL_Gan1_GLEAN_10000112 | GL_Gan1_GLEAN_10001061 | 25.49 | 123.155 | 777.11 | 748.38 | 100.63 |
| GL_Gan1_GLEAN_10000112 | GL_Gan1_GLEAN_10006645 | 71.07 | 116.73 | 87.545 | 89.185 | 134.43 |
| GL_Gan1_GLEAN_10000112 | GL_Gan1_GLEAN_10000112 | 15.39 | 6.44 | 6.115 | 5.72 | 6.5 |
| Cluster:4 |  |  |  |  |  |  |
| GL_Gan1_GLEAN_10000115 | GL_Gan1_GLEAN_10000982 | 7.41 | 5.535 | 2.46 | 2.775 | 4.415 |
| GL_Gan1_GLEAN_10000115 | GL_Gan1_GLEAN_10005579 | 60.31 | 112.675 | 120.11 | 110.37 | 80.455 |
| GL_Gan1_GLEAN_10000115 | GL_Gan1_GLEAN_10000115 | 4.61 | 36.43 | 80.695 | 68.385 | 62.875 |
| GL_Gan1_GLEAN_10000115 | GL_Gan1_GLEAN_10000099 | 2.555 | 11.675 | 13.985 | 5.94 | 4.745 |
| GL_Gan1_GLEAN_10000115 | GL_Gan1_GLEAN_10000201 | 0 | 3.24 | 2.975 | 2.59 | 2.68 |
| GL_Gan1_GLEAN_10000115 | GL_Gan1_GLEAN_10000597 | 718.83 | 81.355 | 76.215 | 116.81 | 29.93 |
| GL_Gan1_GLEAN_10000115 | GL_Gan1_GLEAN_10008518 | 33.675 | 24 | 37.62 | 33.725 | 32.085 |
| GL_Gan1_GLEAN_10000115 | GL_Gan1_GLEAN_10007566 | 1.54 | 5.74 | 4.645 | 8.14 | 7.825 |
| GL_Gan1_GLEAN_10000115 | GL_Gan1_GLEAN_10000029 | 67.75 | 167.3 | 154.615 | 133.395 | 180.58 |
| GL_Gan1_GLEAN_10000115 | GL_Gan1_GLEAN_10000272 | 192.48 | 268.76 | 187.82 | 213.775 | 284.535 |
| GL_Gan1_GLEAN_10000115 | GL_Gan1_GLEAN_10008016 | 0.47 | 0.825 | 1.83 | 2.32 | 1.54 |
| GL_Gan1_GLEAN_10000115 | GL_Gan1_GLEAN_10000856 | 10.565 | 3.52 | 5.665 | 7.625 | 6.15 |
| GL_Gan1_GLEAN_10000115 | GL_Gan1_GLEAN_10001024 | 21.78 | 26.3 | 27.59 | 28.925 | 30.69 |
| GL_Gan1_GLEAN_10000115 | GL_Gan1_GLEAN_10001130 | 14.61 | 1.21 | 2.34 | 3.175 | 2.5 |
| Cluster:5 |  |  |  |  |  |  |
| GL_Gan1_GLEAN_10000243 | GL_Gan1_GLEAN_10001740 | 69.655 | 35.96 | 38.845 | 41.19 | 25.475 |
| GL_Gan1_GLEAN_10000243 | GL_Gan1_GLEAN_10000243 | 0.265 | 0.17 | 0.145 | 0.055 | 0 |
| Cluster:6 |  |  |  |  |  |  |
| GL_Gan1_GLEAN_10000578 | GL_Gan1_GLEAN_10000578 | 16.95 | 82.615 | 51.2 | 53.24 | 79.895 |
| GL_Gan1_GLEAN_10000578 | GL_Gan1_GLEAN_10002198 | 7.94 | 122.23 | 210.61 | 185.505 | 141.465 |
| GL_Gan1_GLEAN_10000578 | GL_Gan1_GLEAN_10001914 | 0 | 6.625 | 5.405 | 6.03 | 7.185 |
| GL_Gan1_GLEAN_10000578 | GL_Gan1_GLEAN_10001433 | 3.56 | 1.825 | 0.38 | 0.48 | 4.3 |
| GL_Gan1_GLEAN_10000578 | GL_Gan1_GLEAN_10006013 | 2342.325 | 1832.5 | 1105.18 | 1175.91 | 1638.64 |
| GL_Gan1_GLEAN_10000578 | GL_Gan1_GLEAN_10002374 | 79.975 | 3.865 | 3.955 | 5 | 2.585 |
| Cluster:7 |  |  |  |  |  |  |
| GL_Gan1_GLEAN_10000585 | GL_Gan1_GLEAN_10000197 | 34.46 | 90.66 | 66.745 | 61.45 | 103.005 |
| GL_Gan1_GLEAN_10000585 | GL_Gan1_GLEAN_10000498 | 2.215 | 2.12 | 2.155 | 1.41 | 2.26 |
| GL_Gan1_GLEAN_10000585 | GL_Gan1_GLEAN_10003085 | 694.23 | 2023.63 | 2277.485 | 1806.895 | 1554.825 |
| GL_Gan1_GLEAN_10000585 | GL_Gan1_GLEAN_10000585 | 20.475 | 19.255 | 19.68 | 14.04 | 19.51 |
| Cluster:8 |  |  |  |  |  |  |
| GL_Gan1_GLEAN_10001146 | GL_Gan1_GLEAN_10001812 | 15.285 | 22.445 | 21.71 | 21.225 | 15.39 |
| GL_Gan1_GLEAN_10001146 | GL_Gan1_GLEAN_10001595 | 0 | 6.42 | 19.55 | 8.815 | 6.81 |
| GL_Gan1_GLEAN_10001146 | GL_Gan1_GLEAN_10004577 | 25.015 | 9.655 | 15.825 | 21.935 | 12.265 |
| GL_Gan1_GLEAN_10001146 | GL_Gan1_GLEAN_10002557 | 7.96 | 38.905 | 35.37 | 43.855 | 35.735 |
| GL_Gan1_GLEAN_10001146 | GL_Gan1_GLEAN_10004051 | 2997.315 | 3464.8 | 2735.67 | 3313.915 | 2610.925 |
| GL_Gan1_GLEAN_10001146 | GL_Gan1_GLEAN_10001010 | 2.335 | 5.155 | 6.96 | 4.965 | 2.79 |
| GL_Gan1_GLEAN_10001146 | GL_Gan1_GLEAN_10000580 | 42.52 | 18.27 | 23.635 | 19.475 | 23.8 |
| GL_Gan1_GLEAN_10001146 | GL_Gan1_GLEAN_10006015 | 83.81 | 141.09 | 87.995 | 69.27 | 145.725 |
| GL_Gan1_GLEAN_10001146 | GL_Gan1_GLEAN_10003327 | 3.93 | 133.825 | 79.155 | 50.93 | 132.03 |
| GL_Gan1_GLEAN_10001146 | GL_Gan1_GLEAN_10003400 | 0 | 0 | 0.845 | 0.445 | 0.615 |
| GL_Gan1_GLEAN_10001146 | GL_Gan1_GLEAN_10000717 | 24.825 | 81.115 | 48.8 | 42.09 | 51.885 |
| GL_Gan1_GLEAN_10001146 | GL_Gan1_GLEAN_10001146 | 12.625 | 14.855 | 9.07 | 9.32 | 9.01 |
| GL_Gan1_GLEAN_10001146 | GL_Gan1_GLEAN_10001118 | 9.595 | 5.145 | 3.425 | 3.73 | 8.875 |
| GL_Gan1_GLEAN_10001146 | GL_Gan1_GLEAN_10001390 | 1.16 | 3.12 | 3.065 | 2.37 | 3.005 |
| GL_Gan1_GLEAN_10001146 | GL_Gan1_GLEAN_10001295 | 8.605 | 2.165 | 11.97 | 4.91 | 5.18 |
| GL_Gan1_GLEAN_10001146 | GL_Gan1_GLEAN_10005737 | 62.87 | 69.565 | 64.625 | 78.005 | 57.155 |
| GL_Gan1_GLEAN_10001146 | GL_Gan1_GLEAN_10002876 | 0 | 8.425 | 12.59 | 10.56 | 2.795 |
| GL_Gan1_GLEAN_10001146 | GL_Gan1_GLEAN_10002488 | 5.6 | 2.725 | 2.14 | 1.72 | 2.82 |
| GL_Gan1_GLEAN_10001146 | GL_Gan1_GLEAN_10003473 | 0 | 0 | 0.22 | 0.765 | 0 |
| GL_Gan1_GLEAN_10001146 | GL_Gan1_GLEAN_10002201 | 2.1 | 6.25 | 6.98 | 6.515 | 6.67 |
| GL_Gan1_GLEAN_10001146 | GL_Gan1_GLEAN_10001435 | 3.51 | 3.375 | 4.16 | 4.12 | 4.405 |
| GL_Gan1_GLEAN_10001146 | GL_Gan1_GLEAN_10002945 | 0 | 1.785 | 1.42 | 0.725 | 1.935 |
| GL_Gan1_GLEAN_10001146 | GL_Gan1_GLEAN_10000883 | 104.625 | 49.38 | 50.55 | 58.405 | 48.66 |
| Cluster:9 |  |  |  |  |  |  |
| GL_Gan1_GLEAN_10001423 | GL_Gan1_GLEAN_10002933 | 0.87 | 4.4 | 4.56 | 3.8 | 3.03 |
| GL_Gan1_GLEAN_10001423 | GL_Gan1_GLEAN_10001040 | 47.08 | 35.91 | 41.705 | 38.57 | 37.795 |
| GL_Gan1_GLEAN_10001423 | GL_Gan1_GLEAN_10008027 | 9.09 | 22.63 | 18.155 | 20.545 | 21.73 |
| GL_Gan1_GLEAN_10001423 | GL_Gan1_GLEAN_10001799 | 67.615 | 45.315 | 86.49 | 87.66 | 33.715 |
| GL_Gan1_GLEAN_10001423 | GL_Gan1_GLEAN_10003390 | 2.755 | 0.305 | 0.825 | 0.785 | 0.885 |
| GL_Gan1_GLEAN_10001423 | GL_Gan1_GLEAN_10001583 | 5.03 | 8.79 | 10.16 | 10.655 | 7.69 |
| GL_Gan1_GLEAN_10001423 | GL_Gan1_GLEAN_10003314 | 103.055 | 63 | 141.175 | 91.62 | 96.305 |
| GL_Gan1_GLEAN_10001423 | GL_Gan1_GLEAN_10002128 | 23.41 | 31.815 | 33.225 | 34.495 | 30.415 |
| GL_Gan1_GLEAN_10001423 | GL_Gan1_GLEAN_10001423 | 1.535 | 2.945 | 1.465 | 1.095 | 2.06 |
| GL_Gan1_GLEAN_10001423 | GL_Gan1_GLEAN_10000543 | 1124.065 | 116.99 | 113.13 | 106.615 | 95.355 |
| GL_Gan1_GLEAN_10001423 | GL_Gan1_GLEAN_10007177 | 619.07 | 881.76 | 1025.705 | 900.52 | 724.465 |
| GL_Gan1_GLEAN_10001423 | GL_Gan1_GLEAN_10004336 | 139.96 | 71.34 | 11.795 | 40.12 | 29.485 |
| GL_Gan1_GLEAN_10001423 | GL_Gan1_GLEAN_10000526 | 30.34 | 15.87 | 19.005 | 18.455 | 19.52 |
| GL_Gan1_GLEAN_10001423 | GL_Gan1_GLEAN_10002363 | 15.6 | 41.35 | 29.935 | 26.47 | 35.74 |
| GL_Gan1_GLEAN_10001423 | GL_Gan1_GLEAN_10004792 | 18.775 | 39.19 | 36.825 | 33.445 | 45.3 |
| GL_Gan1_GLEAN_10001423 | GL_Gan1_GLEAN_10000471 | 34.54 | 25.765 | 13.31 | 13.975 | 21.56 |
| Cluster:10 |  |  |  |  |  |  |
| GL_Gan1_GLEAN_10001739 | GL_Gan1_GLEAN_10001907 | 35.43 | 15.695 | 18.655 | 20.595 | 16.335 |
| GL_Gan1_GLEAN_10001739 | GL_Gan1_GLEAN_10000092 | 235.035 | 17.01 | 15.57 | 18.44 | 3.32 |
| GL_Gan1_GLEAN_10001739 | GL_Gan1_GLEAN_10002465 | 0 | 0.225 | 0 | 0.35 | 0.12 |
| GL_Gan1_GLEAN_10001739 | GL_Gan1_GLEAN_10001370 | 4.26 | 3.5 | 0.285 | 0.705 | 1.93 |
| GL_Gan1_GLEAN_10001739 | GL_Gan1_GLEAN_10000233 | 2934.385 | 3125.515 | 2682.42 | 3069.895 | 2346.895 |
| GL_Gan1_GLEAN_10001739 | GL_Gan1_GLEAN_10000373 | 217.92 | 373.52 | 387.63 | 437.035 | 424.64 |
| GL_Gan1_GLEAN_10001739 | GL_Gan1_GLEAN_10001739 | 5 | 2.455 | 2.82 | 2.96 | 2.465 |
| Cluster:11 |  |  |  |  |  |  |
| GL_Gan1_GLEAN_10002185 | GL_Gan1_GLEAN_10005586 | 122.715 | 31.09 | 47.075 | 49.27 | 38.9 |
| GL_Gan1_GLEAN_10002185 | GL_Gan1_GLEAN_10000405 | 1.91 | 29.115 | 29.505 | 22.575 | 31.76 |
| GL_Gan1_GLEAN_10002185 | GL_Gan1_GLEAN_10004973 | 0 | 2.71 | 2.325 | 1.085 | 0.985 |
| GL_Gan1_GLEAN_10002185 | GL_Gan1_GLEAN_10000327 | 221.665 | 1238.69 | 1192.58 | 1222.305 | 1336.545 |
| GL_Gan1_GLEAN_10002185 | GL_Gan1_GLEAN_10002927 | 81.315 | 4.36 | 7.37 | 13.635 | 2.18 |
| GL_Gan1_GLEAN_10002185 | GL_Gan1_GLEAN_10000658 | 32.485 | 20.675 | 15.55 | 16.475 | 19.71 |
| GL_Gan1_GLEAN_10002185 | GL_Gan1_GLEAN_10000953 | 76.87 | 39.295 | 79.925 | 87.53 | 59.445 |
| GL_Gan1_GLEAN_10002185 | GL_Gan1_GLEAN_10003645 | 158.57 | 32.345 | 32.835 | 31.13 | 38.565 |
| GL_Gan1_GLEAN_10002185 | GL_Gan1_GLEAN_10002185 | 0.575 | 18.9 | 4.02 | 1.475 | 14.055 |
| GL_Gan1_GLEAN_10002185 | GL_Gan1_GLEAN_10004030 | 0 | 1.65 | 2.49 | 1.69 | 1.09 |
| GL_Gan1_GLEAN_10002185 | GL_Gan1_GLEAN_10000729 | 26.62 | 41.56 | 26.55 | 30.055 | 31.085 |
| GL_Gan1_GLEAN_10002185 | GL_Gan1_GLEAN_10007170 | 33.965 | 69.385 | 46.81 | 45.77 | 60.025 |
| GL_Gan1_GLEAN_10002185 | GL_Gan1_GLEAN_10000518 | 6.61 | 21.345 | 23.435 | 21.23 | 12.285 |
| GL_Gan1_GLEAN_10002185 | GL_Gan1_GLEAN_10000220 | 19.995 | 49.13 | 40.095 | 44.74 | 34.39 |
| GL_Gan1_GLEAN_10002185 | GL_Gan1_GLEAN_10002240 | 8.69 | 0.84 | 6.09 | 2.945 | 2.245 |
| GL_Gan1_GLEAN_10002185 | GL_Gan1_GLEAN_10000305 | 0 | 2.04 | 6.155 | 29.535 | 11.385 |
| GL_Gan1_GLEAN_10002185 | GL_Gan1_GLEAN_10005327 | 1931.7 | 2392.37 | 2356.66 | 2606.6 | 1720.885 |
| GL_Gan1_GLEAN_10002185 | GL_Gan1_GLEAN_10002311 | 6.08 | 12.545 | 8.715 | 6.73 | 10.275 |
| GL_Gan1_GLEAN_10002185 | GL_Gan1_GLEAN_10002008 | 8.08 | 12.57 | 8.105 | 7.835 | 19.945 |
| GL_Gan1_GLEAN_10002185 | GL_Gan1_GLEAN_10001524 | 5.995 | 4.24 | 8.685 | 6.125 | 4.335 |
| Cluster:12 |  |  |  |  |  |  |
| GL_Gan1_GLEAN_10002188 | GL_Gan1_GLEAN_10000365 | 0.64 | 3.215 | 2.14 | 3.94 | 2.57 |
| GL_Gan1_GLEAN_10002188 | GL_Gan1_GLEAN_10000411 | 35.505 | 26.03 | 33.44 | 31.89 | 20.68 |
| GL_Gan1_GLEAN_10002188 | GL_Gan1_GLEAN_10003653 | 0 | 0.19 | 0.045 | 0.075 | 0 |
| GL_Gan1_GLEAN_10002188 | GL_Gan1_GLEAN_10001039 | 0 | 9.425 | 6.685 | 3.91 | 3.48 |
| GL_Gan1_GLEAN_10002188 | GL_Gan1_GLEAN_10000255 | 24.075 | 13 | 12.155 | 16.605 | 15.575 |
| GL_Gan1_GLEAN_10002188 | GL_Gan1_GLEAN_10007356 | 33.05 | 13.79 | 13.965 | 18.37 | 11.605 |
| GL_Gan1_GLEAN_10002188 | GL_Gan1_GLEAN_10001250 | 6.025 | 4.77 | 3.06 | 2.325 | 8.91 |
| GL_Gan1_GLEAN_10002188 | GL_Gan1_GLEAN_10002542 | 16.62 | 20.43 | 19.22 | 12.33 | 22.25 |
| GL_Gan1_GLEAN_10002188 | GL_Gan1_GLEAN_10000996 | 101.345 | 166.805 | 121.805 | 131.36 | 156.695 |
| GL_Gan1_GLEAN_10002188 | GL_Gan1_GLEAN_10002315 | 10.61 | 4.475 | 8.42 | 4.285 | 9.94 |
| GL_Gan1_GLEAN_10002188 | GL_Gan1_GLEAN_10002188 | 24.21 | 247.295 | 51.92 | 19.525 | 265.245 |
| GL_Gan1_GLEAN_10002188 | GL_Gan1_GLEAN_10001641 | 2.375 | 4.895 | 5.985 | 6.53 | 6.595 |
| GL_Gan1_GLEAN_10002188 | GL_Gan1_GLEAN_10001143 | 0 | 0 | 0.31 | 0.19 | 0 |
| GL_Gan1_GLEAN_10002188 | GL_Gan1_GLEAN_10000450 | 8.085 | 60.495 | 99.045 | 104.07 | 134.53 |
| GL_Gan1_GLEAN_10002188 | GL_Gan1_GLEAN_10002127 | 144.945 | 700.47 | 682.445 | 649.26 | 694.305 |
| GL_Gan1_GLEAN_10002188 | GL_Gan1_GLEAN_10000845 | 9.1 | 0.935 | 2.6 | 1.025 | 0 |
| GL_Gan1_GLEAN_10002188 | GL_Gan1_GLEAN_10000586 | 15.025 | 12.235 | 12.34 | 7.725 | 8.475 |
| GL_Gan1_GLEAN_10002188 | GL_Gan1_GLEAN_10005592 | 26.45 | 23.035 | 26.275 | 28.79 | 25.06 |
| GL_Gan1_GLEAN_10002188 | GL_Gan1_GLEAN_10004567 | 6.16 | 6.64 | 10.295 | 9.075 | 6.505 |
| GL_Gan1_GLEAN_10002188 | GL_Gan1_GLEAN_10004230 | 359.045 | 319.845 | 321.195 | 317.075 | 268.345 |
| GL_Gan1_GLEAN_10002188 | GL_Gan1_GLEAN_10000500 | 223.195 | 272.68 | 248.18 | 236.745 | 256.445 |
| GL_Gan1_GLEAN_10002188 | GL_Gan1_GLEAN_10006828 | 0 | 2.26 | 1.74 | 0.24 | 1.83 |
| GL_Gan1_GLEAN_10002188 | GL_Gan1_GLEAN_10000930 | 43.42 | 33.435 | 32.975 | 36.72 | 33.11 |
| GL_Gan1_GLEAN_10002188 | GL_Gan1_GLEAN_10000394 | 43.42 | 33.435 | 32.975 | 36.72 | 33.11 |
| GL_Gan1_GLEAN_10002188 | GL_Gan1_GLEAN_10008026 | 2.83 | 1.875 | 4.36 | 3.56 | 2.27 |
| GL_Gan1_GLEAN_10002188 | GL_Gan1_GLEAN_10007578 | 26.33 | 45.885 | 40.99 | 44.085 | 51.255 |
| GL_Gan1_GLEAN_10002188 | GL_Gan1_GLEAN_10005456 | 0.685 | 0 | 0.185 | 0.06 | 0.085 |
| Cluster:13 |  |  |  |  |  |  |
| GL_Gan1_GLEAN_10003559 | GL_Gan1_GLEAN_10003319 | 1.53 | 9.085 | 6.545 | 6.65 | 19.295 |
| GL_Gan1_GLEAN_10003559 | GL_Gan1_GLEAN_10001255 | 27.075 | 54.485 | 65.735 | 62.31 | 59.665 |
| GL_Gan1_GLEAN_10003559 | GL_Gan1_GLEAN_10000738 | 53.23 | 9.505 | 11.765 | 12.56 | 8.235 |
| GL_Gan1_GLEAN_10003559 | GL_Gan1_GLEAN_10001163 | 174.18 | 162.87 | 75.195 | 87.775 | 103.835 |
| GL_Gan1_GLEAN_10003559 | GL_Gan1_GLEAN_10000614 | 4.89 | 17.75 | 9.225 | 11.325 | 13.575 |
| GL_Gan1_GLEAN_10003559 | GL_Gan1_GLEAN_10002815 | 56.88 | 25.375 | 21.48 | 20.6 | 24.655 |
| GL_Gan1_GLEAN_10003559 | GL_Gan1_GLEAN_10002747 | 59.77 | 37.725 | 33.775 | 34.32 | 44.22 |
| GL_Gan1_GLEAN_10003559 | GL_Gan1_GLEAN_10000550 | 0 | 0.17 | 0 | 0.105 | 0.125 |
| GL_Gan1_GLEAN_10003559 | GL_Gan1_GLEAN_10001333 | 32.27 | 30.755 | 35.515 | 33.285 | 36.44 |
| GL_Gan1_GLEAN_10003559 | GL_Gan1_GLEAN_10003559 | 5.07 | 10.805 | 3.4 | 2.665 | 9.425 |
| GL_Gan1_GLEAN_10003559 | GL_Gan1_GLEAN_10006659 | 16.605 | 20 | 33.945 | 32.52 | 15.975 |
| GL_Gan1_GLEAN_10003559 | GL_Gan1_GLEAN_10009174 | 1.635 | 8.09 | 12.3 | 11.87 | 7.235 |
| GL_Gan1_GLEAN_10003559 | GL_Gan1_GLEAN_10002369 | 0.8 | 0.71 | 0.255 | 0.18 | 0.32 |
| GL_Gan1_GLEAN_10003559 | GL_Gan1_GLEAN_10000816 | 0 | 4.665 | 0.36 | 0.3 | 3.965 |
| GL_Gan1_GLEAN_10003559 | GL_Gan1_GLEAN_10000474 | 91.97 | 124.175 | 110.09 | 145.075 | 112.2 |
| GL_Gan1_GLEAN_10003559 | GL_Gan1_GLEAN_10002131 | 66.535 | 40.055 | 52.19 | 43.195 | 48.35 |
| GL_Gan1_GLEAN_10003559 | GL_Gan1_GLEAN_10000416 | 5.415 | 80.37 | 46.175 | 57.995 | 74.48 |
| GL_Gan1_GLEAN_10003559 | GL_Gan1_GLEAN_10004856 | 0 | 1.47 | 4.43 | 0.73 | 4.79 |
| GL_Gan1_GLEAN_10003559 | GL_Gan1_GLEAN_10000963 | 1.615 | 10.225 | 11.535 | 13.495 | 10.21 |
| GL_Gan1_GLEAN_10003559 | GL_Gan1_GLEAN_10004234 | 42.53 | 29.44 | 37.26 | 35.45 | 32.605 |
| GL_Gan1_GLEAN_10003559 | GL_Gan1_GLEAN_10002938 | 23.595 | 5.075 | 5.19 | 5.73 | 6.08 |
| GL_Gan1_GLEAN_10003559 | GL_Gan1_GLEAN_10007586 | 2.495 | 3.595 | 4.735 | 4.55 | 6.215 |
| GL_Gan1_GLEAN_10003559 | GL_Gan1_GLEAN_10003396 | 7.015 | 14.02 | 8.47 | 7.09 | 7.09 |
| GL_Gan1_GLEAN_10003559 | GL_Gan1_GLEAN_10002684 | 0.74 | 4.575 | 4.97 | 3.83 | 5.08 |
| Cluster:14 |  |  |  |  |  |  |
| GL_Gan1_GLEAN_10003654 | GL_Gan1_GLEAN_10000587 | 62.085 | 223.06 | 82.705 | 96.97 | 184.025 |
| GL_Gan1_GLEAN_10003654 | GL_Gan1_GLEAN_10000961 | 9.885 | 14.2 | 12.14 | 12.315 | 19.71 |
| GL_Gan1_GLEAN_10003654 | GL_Gan1_GLEAN_10007179 | 136.565 | 101.25 | 173.205 | 196 | 125.17 |
| GL_Gan1_GLEAN_10003654 | GL_Gan1_GLEAN_10005458 | 0 | 0 | 0.45 | 0.31 | 0 |
| GL_Gan1_GLEAN_10003654 | GL_Gan1_GLEAN_10006656 | 84.81 | 100.615 | 124.715 | 119.87 | 100.98 |
| GL_Gan1_GLEAN_10003654 | GL_Gan1_GLEAN_10001159 | 29.9 | 251.94 | 565.035 | 525.89 | 523.085 |
| GL_Gan1_GLEAN_10003654 | GL_Gan1_GLEAN_10000705 | 0 | 0 | 0.255 | 0.14 | 0 |
| GL_Gan1_GLEAN_10003654 | GL_Gan1_GLEAN_10000611 | 417.435 | 82.015 | 103.685 | 125.51 | 64.135 |
| GL_Gan1_GLEAN_10003654 | GL_Gan1_GLEAN_10005335 | 0 | 0 | 0 | 0 | 0 |
| GL_Gan1_GLEAN_10003654 | GL_Gan1_GLEAN_10002190 | 18.805 | 21.78 | 43.555 | 26.73 | 28.23 |
| GL_Gan1_GLEAN_10003654 | GL_Gan1_GLEAN_10003654 | 0 | 0.885 | 0.385 | 0.405 | 0.815 |
| Cluster:15 |  |  |  |  |  |  |
| GL_Gan1_GLEAN_10003878 | GL_Gan1_GLEAN_10003212 | 0 | 4.935 | 1.62 | 1.07 | 4.125 |
| GL_Gan1_GLEAN_10003878 | GL_Gan1_GLEAN_10005013 | 7.96 | 16.815 | 10.13 | 11.65 | 16.22 |
| GL_Gan1_GLEAN_10003878 | GL_Gan1_GLEAN_10003878 | 5.015 | 16.79 | 32.54 | 21.135 | 25.41 |
| Cluster:16 |  |  |  |  |  |  |
| GL_Gan1_GLEAN_10004031 | GL_Gan1_GLEAN_10006823 | 46.29 | 41.72 | 29.665 | 24 | 44.795 |
| GL_Gan1_GLEAN_10004031 | GL_Gan1_GLEAN_10001245 | 25.14 | 7.015 | 8.49 | 8.935 | 8.485 |
| GL_Gan1_GLEAN_10004031 | GL_Gan1_GLEAN_10002121 | 1527.785 | 951.185 | 1204.68 | 1140.355 | 601.35 |
| GL_Gan1_GLEAN_10004031 | GL_Gan1_GLEAN_10005721 | 111.805 | 197.075 | 169.47 | 176.25 | 160.58 |
| GL_Gan1_GLEAN_10004031 | GL_Gan1_GLEAN_10001741 | 9.83 | 28.275 | 15.215 | 16.255 | 22.24 |
| GL_Gan1_GLEAN_10004031 | GL_Gan1_GLEAN_10001525 | 0 | 0.425 | 0.68 | 0.935 | 1.035 |
| GL_Gan1_GLEAN_10004031 | GL_Gan1_GLEAN_10002062 | 18.885 | 12.445 | 16.865 | 16.645 | 16.715 |
| GL_Gan1_GLEAN_10004031 | GL_Gan1_GLEAN_10004031 | 1.07 | 4.225 | 3.375 | 3.185 | 3.995 |
| GL_Gan1_GLEAN_10004031 | GL_Gan1_GLEAN_10000990 | 0 | 84.46 | 0.515 | 0.51 | 49.5 |
| Cluster:17 |  |  |  |  |  |  |
| GL_Gan1_GLEAN_10004038 | GL_Gan1_GLEAN_10000775 | 166.07 | 130.08 | 178.02 | 197.48 | 122.76 |
| GL_Gan1_GLEAN_10004038 | GL_Gan1_GLEAN_10005461 | 6.54 | 3.59 | 5.545 | 5.87 | 5.335 |
| GL_Gan1_GLEAN_10004038 | GL_Gan1_GLEAN_10002545 | 8.29 | 63.04 | 29.99 | 31.59 | 64.885 |
| GL_Gan1_GLEAN_10004038 | GL_Gan1_GLEAN_10004852 | 219.155 | 210.835 | 189.505 | 169.165 | 189.66 |
| GL_Gan1_GLEAN_10004038 | GL_Gan1_GLEAN_10002681 | 3.95 | 63.75 | 98.42 | 99.685 | 61.305 |
| GL_Gan1_GLEAN_10004038 | GL_Gan1_GLEAN_10005337 | 57.56 | 30.83 | 48.015 | 44.075 | 43.255 |
| GL_Gan1_GLEAN_10004038 | GL_Gan1_GLEAN_10004038 | 78.82 | 8.2 | 7.775 | 7.83 | 5.48 |
| GL_Gan1_GLEAN_10004038 | GL_Gan1_GLEAN_10003016 | 5.39 | 19.565 | 13.345 | 14.56 | 19.87 |
| GL_Gan1_GLEAN_10004038 | GL_Gan1_GLEAN_10003557 | 234.15 | 695.315 | 1853.94 | 1577.3 | 615.545 |
| GL_Gan1_GLEAN_10004038 | GL_Gan1_GLEAN_10000814 | 0 | 0.42 | 0.095 | 0 | 0.305 |
| GL_Gan1_GLEAN_10004038 | GL_Gan1_GLEAN_10001533 | 208.045 | 197.29 | 145.495 | 175.135 | 203.275 |
| GL_Gan1_GLEAN_10004038 | GL_Gan1_GLEAN_10005199 | 0 | 1.34 | 0.73 | 0.69 | 1.335 |
| GL_Gan1_GLEAN_10004038 | GL_Gan1_GLEAN_10000431 | 48.435 | 19.4 | 19.05 | 19.86 | 17.845 |
| GL_Gan1_GLEAN_10004038 | GL_Gan1_GLEAN_10000547 | 12.475 | 4.405 | 3.08 | 2.835 | 3.4 |
| GL_Gan1_GLEAN_10004038 | GL_Gan1_GLEAN_10000933 | 53.91 | 11.56 | 12.985 | 11.32 | 12.65 |
| GL_Gan1_GLEAN_10004038 | GL_Gan1_GLEAN_10000397 | 53.91 | 11.56 | 12.985 | 11.32 | 12.65 |
| GL_Gan1_GLEAN_10004038 | GL_Gan1_GLEAN_10000571 | 18.83 | 13.52 | 14.775 | 10.23 | 11.945 |
| Cluster:18 |  |  |  |  |  |  |
| GL_Gan1_GLEAN_10004192 | GL_Gan1_GLEAN_10006560 | 4.025 | 8.335 | 13.615 | 10.57 | 8.83 |
| GL_Gan1_GLEAN_10004192 | GL_Gan1_GLEAN_10008075 | 41.55 | 35.7 | 61.6 | 53.425 | 31.875 |
| GL_Gan1_GLEAN_10004192 | GL_Gan1_GLEAN_10003524 | 45.955 | 25.97 | 23.67 | 22.8 | 24.44 |
| GL_Gan1_GLEAN_10004192 | GL_Gan1_GLEAN_10005649 | 73.26 | 109.57 | 109.57 | 113.92 | 93.525 |
| GL_Gan1_GLEAN_10004192 | GL_Gan1_GLEAN_10005941 | 67.56 | 43.66 | 53.235 | 57.83 | 49.67 |
| GL_Gan1_GLEAN_10004192 | GL_Gan1_GLEAN_10004919 | 3.89 | 3.5 | 1.955 | 1.51 | 4.135 |
| GL_Gan1_GLEAN_10004192 | GL_Gan1_GLEAN_10003713 | 0 | 0.2 | 0.55 | 0.195 | 0 |
| GL_Gan1_GLEAN_10004192 | GL_Gan1_GLEAN_10004515 | 11.825 | 21.85 | 12.83 | 16.125 | 25.225 |
| GL_Gan1_GLEAN_10004192 | GL_Gan1_GLEAN_10006716 | 8.02 | 26.525 | 21.675 | 24.895 | 20.27 |
| GL_Gan1_GLEAN_10004192 | GL_Gan1_GLEAN_10003610 | 94.065 | 66.275 | 38.66 | 42.28 | 60.51 |
| GL_Gan1_GLEAN_10004192 | GL_Gan1_GLEAN_10007863 | 176.8 | 41.5 | 53.485 | 58.55 | 44.875 |
| GL_Gan1_GLEAN_10004192 | GL_Gan1_GLEAN_10004192 | 6.05 | 3.095 | 5.48 | 4.47 | 3.695 |
| Cluster:19 |  |  |  |  |  |  |
| GL_Gan1_GLEAN_10004425 | GL_Gan1_GLEAN_10006572 | 30.055 | 2.865 | 7.275 | 3.53 | 5.09 |
| GL_Gan1_GLEAN_10004425 | GL_Gan1_GLEAN_10009253 | 175.235 | 130.37 | 170.48 | 159.3 | 134.755 |
| GL_Gan1_GLEAN_10004425 | GL_Gan1_GLEAN_10005277 | 126.985 | 212.72 | 221.055 | 207.345 | 197.135 |
| GL_Gan1_GLEAN_10004425 | GL_Gan1_GLEAN_10005527 | 0 | 2.105 | 0.735 | 0.635 | 0.875 |
| GL_Gan1_GLEAN_10004425 | GL_Gan1_GLEAN_10003540 | 81.78 | 28.88 | 34.815 | 42.42 | 31.06 |
| GL_Gan1_GLEAN_10004425 | GL_Gan1_GLEAN_10005149 | 100.575 | 47.15 | 90.72 | 112.08 | 69.815 |
| GL_Gan1_GLEAN_10004425 | GL_Gan1_GLEAN_10003898 | 70.785 | 142.22 | 147.82 | 160.105 | 152.285 |
| GL_Gan1_GLEAN_10004425 | GL_Gan1_GLEAN_10006401 | 16.415 | 41.07 | 38.175 | 38.73 | 48.34 |
| GL_Gan1_GLEAN_10004425 | GL_Gan1_GLEAN_10003629 | 14.885 | 25.96 | 21.215 | 22.015 | 25.06 |
| GL_Gan1_GLEAN_10004425 | GL_Gan1_GLEAN_10004006 | 3.18 | 3 | 1.185 | 1.78 | 2.465 |
| GL_Gan1_GLEAN_10004425 | GL_Gan1_GLEAN_10004425 | 4.43 | 10.85 | 6.455 | 4.06 | 6.935 |
| GL_Gan1_GLEAN_10004425 | GL_Gan1_GLEAN_10006889 | 22.665 | 38.355 | 68.485 | 62.23 | 37.385 |
| GL_Gan1_GLEAN_10004425 | GL_Gan1_GLEAN_10004316 | 31.14 | 11.64 | 32.825 | 18.23 | 13.425 |
| GL_Gan1_GLEAN_10004425 | GL_Gan1_GLEAN_10007246 | 19.52 | 5.545 | 14.715 | 12.94 | 10.44 |
| GL_Gan1_GLEAN_10004425 | GL_Gan1_GLEAN_10005037 | 21.445 | 8.05 | 10.875 | 8.64 | 7.53 |
| GL_Gan1_GLEAN_10004425 | GL_Gan1_GLEAN_10003628 | 231.675 | 161.395 | 219.275 | 245.84 | 139.265 |
| GL_Gan1_GLEAN_10004425 | GL_Gan1_GLEAN_10007652 | 64.61 | 20.945 | 19.18 | 19.225 | 22.205 |
| GL_Gan1_GLEAN_10004425 | GL_Gan1_GLEAN_10004935 | 81.085 | 43.765 | 98.225 | 111.68 | 76.985 |
| GL_Gan1_GLEAN_10004425 | GL_Gan1_GLEAN_10004005 | 822.305 | 120.685 | 229.57 | 216.81 | 50.41 |
| GL_Gan1_GLEAN_10004425 | GL_Gan1_GLEAN_10004107 | 0 | 0.8 | 0 | 0 | 0 |
| GL_Gan1_GLEAN_10004425 | GL_Gan1_GLEAN_10007437 | 171.205 | 232.385 | 167.46 | 162.325 | 77.165 |
| Cluster:20 |  |  |  |  |  |  |
| GL_Gan1_GLEAN_10004432 | GL_Gan1_GLEAN_10006899 | 13.25 | 8.265 | 8.57 | 7.745 | 6.965 |
| GL_Gan1_GLEAN_10004432 | GL_Gan1_GLEAN_10006576 | 0 | 0 | 4.925 | 3.73 | 3.09 |
| GL_Gan1_GLEAN_10004432 | GL_Gan1_GLEAN_10005285 | 50.935 | 81.945 | 46.475 | 52.535 | 77.685 |
| GL_Gan1_GLEAN_10004432 | GL_Gan1_GLEAN_10004533 | 17.455 | 0 | 0.91 | 0.91 | 2.44 |
| GL_Gan1_GLEAN_10004432 | GL_Gan1_GLEAN_10004432 | 41.95 | 27.61 | 26.745 | 19.725 | 18.31 |
| GL_Gan1_GLEAN_10004432 | GL_Gan1_GLEAN_10007883 | 0 | 4.175 | 7.01 | 8.035 | 3.135 |
| GL_Gan1_GLEAN_10004432 | GL_Gan1_GLEAN_10004942 | 8.68 | 26.54 | 10.79 | 8.54 | 25.435 |
| GL_Gan1_GLEAN_10004432 | GL_Gan1_GLEAN_10007448 | 17.105 | 15.725 | 18.925 | 19.8 | 23.03 |
| GL_Gan1_GLEAN_10004432 | GL_Gan1_GLEAN_10005411 | 29.22 | 63.74 | 87.5 | 72.055 | 104.23 |
| GL_Gan1_GLEAN_10004432 | GL_Gan1_GLEAN_10006410 | 122.985 | 116.99 | 157.77 | 181.465 | 93.24 |
| GL_Gan1_GLEAN_10004432 | GL_Gan1_GLEAN_10006738 | 36.79 | 44.66 | 61.245 | 45.215 | 44.4 |
| GL_Gan1_GLEAN_10004432 | GL_Gan1_GLEAN_10005532 | 37.655 | 37.03 | 40.905 | 32.61 | 76.815 |
| Cluster:21 |  |  |  |  |  |  |
| GL_Gan1_GLEAN_10004832 | GL_Gan1_GLEAN_10002905 | 20.465 | 1 | 1.5 | 1.67 | 1.44 |
| GL_Gan1_GLEAN_10004832 | GL_Gan1_GLEAN_10003507 | 7.025 | 44.135 | 34.675 | 33.495 | 46.065 |
| GL_Gan1_GLEAN_10004832 | GL_Gan1_GLEAN_10006372 | 76.885 | 68.13 | 110.775 | 112.095 | 68.185 |
| GL_Gan1_GLEAN_10004832 | GL_Gan1_GLEAN_10003272 | 21.845 | 125.75 | 122.345 | 56.135 | 158.49 |
| GL_Gan1_GLEAN_10004832 | GL_Gan1_GLEAN_10005375 | 856.375 | 43.025 | 83.055 | 58.53 | 91.28 |
| GL_Gan1_GLEAN_10004832 | GL_Gan1_GLEAN_10002230 | 0 | 5.4 | 8.57 | 7.22 | 4.93 |
| GL_Gan1_GLEAN_10004832 | GL_Gan1_GLEAN_10003351 | 269.475 | 236.865 | 315.02 | 348.015 | 269.125 |
| GL_Gan1_GLEAN_10004832 | GL_Gan1_GLEAN_10007403 | 66.265 | 15.115 | 32.32 | 35.03 | 24.89 |
| GL_Gan1_GLEAN_10004832 | GL_Gan1_GLEAN_10004832 | 25.205 | 6.135 | 6.65 | 8.45 | 6.66 |
| GL_Gan1_GLEAN_10004832 | GL_Gan1_GLEAN_10008056 | 5.895 | 75.555 | 54.575 | 61.165 | 75.435 |
| GL_Gan1_GLEAN_10004832 | GL_Gan1_GLEAN_10002296 | 39.01 | 39.795 | 23.815 | 22.81 | 58.09 |
| GL_Gan1_GLEAN_10004832 | GL_Gan1_GLEAN_10006200 | 2.235 | 10.755 | 10.405 | 9.78 | 12.89 |
| GL_Gan1_GLEAN_10004832 | GL_Gan1_GLEAN_10006861 | 230.535 | 2070.595 | 4768.035 | 3249.94 | 2616.69 |
| GL_Gan1_GLEAN_10004832 | GL_Gan1_GLEAN_10002840 | 39.17 | 40.3 | 31.105 | 36.53 | 42.65 |
| GL_Gan1_GLEAN_10004832 | GL_Gan1_GLEAN_10007048 | 0.84 | 9.06 | 7.295 | 3.9 | 10.2 |
| GL_Gan1_GLEAN_10004832 | GL_Gan1_GLEAN_10004384 | 2.7 | 0.605 | 1.715 | 1.795 | 0.325 |
| GL_Gan1_GLEAN_10004832 | GL_Gan1_GLEAN_10002787 | 9.835 | 3.605 | 1.48 | 3.525 | 2.46 |
| GL_Gan1_GLEAN_10004832 | GL_Gan1_GLEAN_10008880 | 23.595 | 27.27 | 27.025 | 30.645 | 29.14 |
| GL_Gan1_GLEAN_10004832 | GL_Gan1_GLEAN_10005631 | 1697.83 | 834.235 | 600.955 | 803.33 | 1056.62 |
| Cluster:22 |  |  |  |  |  |  |
| GL_Gan1_GLEAN_10004988 | GL_Gan1_GLEAN_10001507 | 21.075 | 12.215 | 18.475 | 17.655 | 12.545 |
| GL_Gan1_GLEAN_10004988 | GL_Gan1_GLEAN_10006531 | 94.015 | 74.31 | 113.185 | 125.775 | 57.69 |
| GL_Gan1_GLEAN_10004988 | GL_Gan1_GLEAN_10004479 | 3.89 | 39.54 | 20.815 | 19.21 | 60.02 |
| GL_Gan1_GLEAN_10004988 | GL_Gan1_GLEAN_10004365 | 103.035 | 84.385 | 83.72 | 60.315 | 93.46 |
| GL_Gan1_GLEAN_10004988 | GL_Gan1_GLEAN_10002217 | 60.665 | 48.615 | 80.48 | 79.075 | 52.77 |
| GL_Gan1_GLEAN_10004988 | GL_Gan1_GLEAN_10002890 | 23.04 | 64.69 | 33.34 | 34.555 | 64.225 |
| GL_Gan1_GLEAN_10004988 | GL_Gan1_GLEAN_10009198 | 0 | 0.885 | 1.075 | 1.375 | 2.285 |
| GL_Gan1_GLEAN_10004988 | GL_Gan1_GLEAN_10004878 | 3.18 | 15.795 | 27.16 | 13.59 | 20.585 |
| GL_Gan1_GLEAN_10004988 | GL_Gan1_GLEAN_10002275 | 94.09 | 133.51 | 129.865 | 128.955 | 143.97 |
| GL_Gan1_GLEAN_10004988 | GL_Gan1_GLEAN_10001308 | 58.91 | 59.52 | 59.635 | 72.68 | 69.76 |
| GL_Gan1_GLEAN_10004988 | GL_Gan1_GLEAN_10001186 | 1.11 | 12.705 | 5.365 | 8.815 | 12.16 |
| GL_Gan1_GLEAN_10004988 | GL_Gan1_GLEAN_10005755 | 0 | 5.12 | 4.035 | 3.395 | 3.06 |
| GL_Gan1_GLEAN_10004988 | GL_Gan1_GLEAN_10004988 | 0.9 | 14.94 | 5.415 | 6.295 | 15.975 |
| GL_Gan1_GLEAN_10004988 | GL_Gan1_GLEAN_10008048 | 6.605 | 70.525 | 9.42 | 11.915 | 57.87 |
| Cluster:23 |  |  |  |  |  |  |
| GL_Gan1_GLEAN_10005079 | GL_Gan1_GLEAN_10005079 | 4.86 | 5.235 | 4.735 | 2.975 | 6.17 |
| GL_Gan1_GLEAN_10005079 | GL_Gan1_GLEAN_10002063 | 72.33 | 34.65 | 92.575 | 64.625 | 37.905 |
| GL_Gan1_GLEAN_10005079 | GL_Gan1_GLEAN_10001419 | 75.38 | 28.43 | 22.42 | 22.09 | 25.91 |
| GL_Gan1_GLEAN_10005079 | GL_Gan1_GLEAN_10000604 | 28.795 | 28.07 | 18.16 | 17.475 | 30.21 |
| GL_Gan1_GLEAN_10005079 | GL_Gan1_GLEAN_10003836 | 7.31 | 104.405 | 132.685 | 139.01 | 140.77 |
| GL_Gan1_GLEAN_10005079 | GL_Gan1_GLEAN_10001154 | 55.735 | 24.525 | 26.72 | 29.305 | 26.015 |
| GL_Gan1_GLEAN_10005079 | GL_Gan1_GLEAN_10000347 | 15.145 | 2.83 | 1.475 | 2.205 | 2.765 |
| GL_Gan1_GLEAN_10005079 | GL_Gan1_GLEAN_10001200 | 163.64 | 53.71 | 45.94 | 49.145 | 24.085 |
| GL_Gan1_GLEAN_10005079 | GL_Gan1_GLEAN_10002539 | 0 | 4.68 | 3.14 | 3.24 | 12.16 |
| GL_Gan1_GLEAN_10005079 | GL_Gan1_GLEAN_10000483 | 0 | 4.075 | 2.62 | 1.545 | 6.84 |
| Cluster:24 |  |  |  |  |  |  |
| GL_Gan1_GLEAN_10005440 | GL_Gan1_GLEAN_10007489 | 11.88 | 8.935 | 6.285 | 6.34 | 7.9 |
| GL_Gan1_GLEAN_10005440 | GL_Gan1_GLEAN_10009294 | 108.41 | 132.13 | 111.7 | 130.565 | 113.985 |
| GL_Gan1_GLEAN_10005440 | GL_Gan1_GLEAN_10005978 | 151.195 | 36.46 | 49.97 | 53.42 | 49.715 |
| GL_Gan1_GLEAN_10005440 | GL_Gan1_GLEAN_10007121 | 57.615 | 34.53 | 37.175 | 31.425 | 31.9 |
| GL_Gan1_GLEAN_10005440 | GL_Gan1_GLEAN_10005566 | 13.74 | 16.5 | 19.4 | 23.055 | 18.875 |
| GL_Gan1_GLEAN_10005440 | GL_Gan1_GLEAN_10006270 | 7.2 | 4.645 | 3.78 | 3.135 | 2.96 |
| GL_Gan1_GLEAN_10005440 | GL_Gan1_GLEAN_10008370 | 16.025 | 15.305 | 26.32 | 22.18 | 22.76 |
| GL_Gan1_GLEAN_10005440 | GL_Gan1_GLEAN_10006771 | 17.845 | 41.93 | 4.365 | 6.79 | 29.325 |
| GL_Gan1_GLEAN_10005440 | GL_Gan1_GLEAN_10006938 | 97.6 | 179.04 | 131.985 | 138.01 | 177.62 |
| GL_Gan1_GLEAN_10005440 | GL_Gan1_GLEAN_10006116 | 378.14 | 179.235 | 414.33 | 360.945 | 291.835 |
| GL_Gan1_GLEAN_10005440 | GL_Gan1_GLEAN_10005440 | 0.31 | 0.04 | 0.19 | 0.195 | 0.17 |
| GL_Gan1_GLEAN_10005440 | GL_Gan1_GLEAN_10007297 | 6.62 | 16.395 | 23.85 | 22.61 | 9.15 |
| GL_Gan1_GLEAN_10005440 | GL_Gan1_GLEAN_10005849 | 67.77 | 33.54 | 37.51 | 38.4 | 32.645 |
| GL_Gan1_GLEAN_10005440 | GL_Gan1_GLEAN_10007488 | 15.09 | 55.205 | 27.065 | 28.21 | 52.645 |
| GL_Gan1_GLEAN_10005440 | GL_Gan1_GLEAN_10006770 | 58.405 | 362.07 | 262.425 | 246.145 | 329.865 |
| GL_Gan1_GLEAN_10005440 | GL_Gan1_GLEAN_10006455 | 57.53 | 48.615 | 35.175 | 42.155 | 53.535 |
| GL_Gan1_GLEAN_10005440 | GL_Gan1_GLEAN_10005977 | 0 | 2.325 | 0.295 | 0 | 2.66 |
| GL_Gan1_GLEAN_10005440 | GL_Gan1_GLEAN_10007698 | 1541.165 | 523.715 | 611.995 | 613.285 | 636.225 |
| GL_Gan1_GLEAN_10005440 | GL_Gan1_GLEAN_10009293 | 56.145 | 56.715 | 75.2 | 89.4 | 74 |
| GL_Gan1_GLEAN_10005440 | GL_Gan1_GLEAN_10008953 | 0 | 1.67 | 2.99 | 1.935 | 1.92 |
| Cluster:25 |  |  |  |  |  |  |
| GL_Gan1_GLEAN_10005660 | GL_Gan1_GLEAN_10004312 | 0.81 | 6.76 | 10.145 | 4.07 | 7.84 |
| GL_Gan1_GLEAN_10005660 | GL_Gan1_GLEAN_10008092 | 2422.395 | 1413.87 | 2822.645 | 1951.075 | 2121.695 |
| GL_Gan1_GLEAN_10005660 | GL_Gan1_GLEAN_10003378 | 4.2 | 18.915 | 21.55 | 16.94 | 10.72 |
| GL_Gan1_GLEAN_10005660 | GL_Gan1_GLEAN_10005033 | 23.065 | 188.36 | 314.72 | 323.925 | 155.71 |
| GL_Gan1_GLEAN_10005660 | GL_Gan1_GLEAN_10005660 | 5.185 | 5.72 | 3.06 | 3.355 | 7.615 |
| GL_Gan1_GLEAN_10005660 | GL_Gan1_GLEAN_10003624 | 276.19 | 1655.555 | 914.485 | 1753.15 | 1367.99 |
| GL_Gan1_GLEAN_10005660 | GL_Gan1_GLEAN_10005401 | 138.66 | 221.78 | 270.455 | 239.875 | 165.885 |
| GL_Gan1_GLEAN_10005660 | GL_Gan1_GLEAN_10004526 | 8.145 | 14.34 | 16.11 | 13.54 | 14.06 |
| GL_Gan1_GLEAN_10005660 | GL_Gan1_GLEAN_10004633 | 0 | 7.48 | 4.855 | 5.065 | 6.04 |
| GL_Gan1_GLEAN_10005660 | GL_Gan1_GLEAN_10006229 | 59.07 | 53.465 | 36.75 | 38.87 | 44.55 |
| GL_Gan1_GLEAN_10005660 | GL_Gan1_GLEAN_10004200 | 8.79 | 27.62 | 36.77 | 30.55 | 36.215 |
| GL_Gan1_GLEAN_10005660 | GL_Gan1_GLEAN_10006071 | 39.245 | 16.425 | 21.275 | 21.66 | 23.285 |
| GL_Gan1_GLEAN_10005660 | GL_Gan1_GLEAN_10008602 | 4033.665 | 3168.575 | 2767.05 | 3238.315 | 2478.055 |
| GL_Gan1_GLEAN_10005660 | GL_Gan1_GLEAN_10008327 | 13.625 | 25.485 | 35.105 | 35.74 | 58.01 |
| Cluster:26 |  |  |  |  |  |  |
| GL_Gan1_GLEAN_10005700 | GL_Gan1_GLEAN_10008138 | 76.51 | 15.425 | 12.17 | 14.25 | 8.835 |
| GL_Gan1_GLEAN_10005700 | GL_Gan1_GLEAN_10006449 | 0 | 5.875 | 9.835 | 11.475 | 6.335 |
| GL_Gan1_GLEAN_10005700 | GL_Gan1_GLEAN_10005179 | 1259.465 | 1822.955 | 1412.01 | 1149.32 | 1567.065 |
| GL_Gan1_GLEAN_10005700 | GL_Gan1_GLEAN_10005559 | 1.325 | 12.565 | 3.09 | 6.845 | 10.165 |
| GL_Gan1_GLEAN_10005700 | GL_Gan1_GLEAN_10005069 | 229.14 | 181.53 | 245.57 | 198.25 | 148.81 |
| GL_Gan1_GLEAN_10005700 | GL_Gan1_GLEAN_10005700 | 8.4 | 57.2 | 14.405 | 30.39 | 62.67 |
| GL_Gan1_GLEAN_10005700 | GL_Gan1_GLEAN_10005844 | 60.35 | 31.43 | 25.705 | 21.885 | 33.86 |
| GL_Gan1_GLEAN_10005700 | GL_Gan1_GLEAN_10006108 | 12.505 | 5.92 | 3.845 | 6.125 | 2.825 |
| GL_Gan1_GLEAN_10005700 | GL_Gan1_GLEAN_10005322 | 1827.595 | 333.215 | 356.925 | 402.085 | 139.69 |
| GL_Gan1_GLEAN_10005700 | GL_Gan1_GLEAN_10005432 | 20.595 | 10.5 | 6.595 | 6.43 | 8.29 |
| GL_Gan1_GLEAN_10005700 | GL_Gan1_GLEAN_10007908 | 288.445 | 338.155 | 303.405 | 360.605 | 314.19 |
| GL_Gan1_GLEAN_10005700 | GL_Gan1_GLEAN_10009287 | 120.71 | 130.805 | 100.79 | 96.305 | 140.75 |
| GL_Gan1_GLEAN_10005700 | GL_Gan1_GLEAN_10006264 | 7.33 | 6.71 | 4.475 | 8.325 | 5.565 |
| GL_Gan1_GLEAN_10005700 | GL_Gan1_GLEAN_10008645 | 0 | 0.63 | 0.205 | 0.635 | 0.395 |
| GL_Gan1_GLEAN_10005700 | GL_Gan1_GLEAN_10008951 | 288.875 | 31.645 | 49.11 | 42.94 | 26.905 |
| GL_Gan1_GLEAN_10005700 | GL_Gan1_GLEAN_10005973 | 0.775 | 0.505 | 1.065 | 1.505 | 0.28 |
| Cluster:27 |  |  |  |  |  |  |
| GL_Gan1_GLEAN_10005703 | GL_Gan1_GLEAN_10008369 | 25.305 | 8.08 | 10.155 | 9.975 | 9.66 |
| GL_Gan1_GLEAN_10005703 | GL_Gan1_GLEAN_10008141 | 326.745 | 41.29 | 34.27 | 52.11 | 19.99 |
| GL_Gan1_GLEAN_10005703 | GL_Gan1_GLEAN_10008649 | 1460.085 | 64.765 | 61.985 | 55.165 | 24.125 |
| GL_Gan1_GLEAN_10005703 | GL_Gan1_GLEAN_10006114 | 25.78 | 73.2 | 73.05 | 83.425 | 68.545 |
| GL_Gan1_GLEAN_10005703 | GL_Gan1_GLEAN_10007487 | 5.99 | 105 | 6.84 | 6.505 | 9.885 |
| GL_Gan1_GLEAN_10005703 | GL_Gan1_GLEAN_10005703 | 0.54 | 17.74 | 2.63 | 17.475 | 30.45 |
| GL_Gan1_GLEAN_10005703 | GL_Gan1_GLEAN_10007120 | 41.945 | 28.295 | 28.335 | 32.545 | 32.005 |
| GL_Gan1_GLEAN_10005703 | GL_Gan1_GLEAN_10007486 | 53.97 | 29.27 | 21.75 | 25.025 | 28.43 |
| GL_Gan1_GLEAN_10005703 | GL_Gan1_GLEAN_10009291 | 38.41 | 248.035 | 126.425 | 194.72 | 229.045 |
| GL_Gan1_GLEAN_10005703 | GL_Gan1_GLEAN_10005976 | 0 | 7.62 | 6.71 | 10.545 | 7.965 |
| GL_Gan1_GLEAN_10005703 | GL_Gan1_GLEAN_10005564 | 26.465 | 22.595 | 37.07 | 60.69 | 15.715 |
| GL_Gan1_GLEAN_10005703 | GL_Gan1_GLEAN_10007697 | 57.19 | 85.865 | 152.145 | 141.975 | 63.82 |
| GL_Gan1_GLEAN_10005703 | GL_Gan1_GLEAN_10007913 | 18.995 | 20.37 | 24.255 | 23.33 | 22.685 |
| GL_Gan1_GLEAN_10005703 | GL_Gan1_GLEAN_10006453 | 5.07 | 11.135 | 12.73 | 17.545 | 9.665 |
| GL_Gan1_GLEAN_10005703 | GL_Gan1_GLEAN_10006767 | 0 | 10.92 | 7.22 | 9.085 | 9.015 |
| GL_Gan1_GLEAN_10005703 | GL_Gan1_GLEAN_10007295 | 26.44 | 17.48 | 21.495 | 19.72 | 21.14 |
| GL_Gan1_GLEAN_10005703 | GL_Gan1_GLEAN_10006113 | 0 | 0.59 | 1.44 | 0.59 | 0.69 |
| GL_Gan1_GLEAN_10005703 | GL_Gan1_GLEAN_10005437 | 2.37 | 47.61 | 33.36 | 38.835 | 43.97 |
| GL_Gan1_GLEAN_10005703 | GL_Gan1_GLEAN_10005702 | 2.825 | 148.49 | 20.53 | 137.475 | 184.325 |
| Cluster:28 |  |  |  |  |  |  |
| GL_Gan1_GLEAN_10005956 | GL_Gan1_GLEAN_10005956 | 10.565 | 22.245 | 22.775 | 31.175 | 29.95 |
| GL_Gan1_GLEAN_10005956 | GL_Gan1_GLEAN_10004744 | 215.39 | 103.13 | 113.845 | 119.075 | 98.345 |
| GL_Gan1_GLEAN_10005956 | GL_Gan1_GLEAN_10007650 | 69.745 | 60.67 | 53.6 | 56.02 | 66.445 |
| GL_Gan1_GLEAN_10005956 | GL_Gan1_GLEAN_10004635 | 270.31 | 544.305 | 686.425 | 703.415 | 456.025 |
| GL_Gan1_GLEAN_10005956 | GL_Gan1_GLEAN_10004313 | 13.39 | 147.355 | 138.2 | 79.74 | 144.75 |
| GL_Gan1_GLEAN_10005956 | GL_Gan1_GLEAN_10005803 | 96.915 | 80.26 | 83.275 | 89.155 | 74.24 |
| GL_Gan1_GLEAN_10005956 | GL_Gan1_GLEAN_10005402 | 21.255 | 14.395 | 19.8 | 21.355 | 14.945 |
| GL_Gan1_GLEAN_10005956 | GL_Gan1_GLEAN_10004422 | 1.77 | 25.49 | 21.45 | 19.86 | 19.27 |
| Cluster:29 |  |  |  |  |  |  |
| GL_Gan1_GLEAN_10006046 | GL_Gan1_GLEAN_10002790 | 29.095 | 14.465 | 17.005 | 19.19 | 19.23 |
| GL_Gan1_GLEAN_10006046 | GL_Gan1_GLEAN_10007847 | 0 | 2.255 | 1.32 | 2.54 | 1.055 |
| GL_Gan1_GLEAN_10006046 | GL_Gan1_GLEAN_10004835 | 7.725 | 11.16 | 13.065 | 11.26 | 13.84 |
| GL_Gan1_GLEAN_10006046 | GL_Gan1_GLEAN_10003871 | 13.2 | 8.53 | 4.305 | 4.665 | 6.775 |
| GL_Gan1_GLEAN_10006046 | GL_Gan1_GLEAN_10006046 | 0.805 | 3.585 | 0.385 | 0.21 | 2.63 |
| GL_Gan1_GLEAN_10006046 | GL_Gan1_GLEAN_10004287 | 391.64 | 182.475 | 358.37 | 368.12 | 163.305 |
| GL_Gan1_GLEAN_10006046 | GL_Gan1_GLEAN_10006864 | 23.98 | 46.54 | 48.205 | 48.08 | 58.855 |
| GL_Gan1_GLEAN_10006046 | GL_Gan1_GLEAN_10003202 | 1.335 | 1.17 | 2.395 | 3.02 | 1.43 |
| GL_Gan1_GLEAN_10006046 | GL_Gan1_GLEAN_10002176 | 48.48 | 49.62 | 68.985 | 66.48 | 47.565 |
| GL_Gan1_GLEAN_10006046 | GL_Gan1_GLEAN_10005240 | 0 | 48.28 | 24.115 | 26.025 | 43.565 |
| GL_Gan1_GLEAN_10006046 | GL_Gan1_GLEAN_10004389 | 238.57 | 185.38 | 210.315 | 151.24 | 214.81 |
| GL_Gan1_GLEAN_10006046 | GL_Gan1_GLEAN_10006547 | 10.165 | 21.77 | 18.79 | 10.78 | 30.045 |
| Cluster:30 |  |  |  |  |  |  |
| GL_Gan1_GLEAN_10006089 | GL_Gan1_GLEAN_10006089 | 79.5 | 8.36 | 41.76 | 15.62 | 32.495 |
| GL_Gan1_GLEAN_10006089 | GL_Gan1_GLEAN_10007664 | 312.36 | 340.67 | 466.55 | 432.18 | 259.15 |
| GL_Gan1_GLEAN_10006089 | GL_Gan1_GLEAN_10008926 | 11.825 | 49.685 | 67.86 | 69.42 | 52.625 |
| GL_Gan1_GLEAN_10006089 | GL_Gan1_GLEAN_10004761 | 175.395 | 20.335 | 28.1 | 31.155 | 20.235 |
| GL_Gan1_GLEAN_10006089 | GL_Gan1_GLEAN_10006906 | 113.965 | 102.905 | 131.015 | 135.915 | 92.44 |
| GL_Gan1_GLEAN_10006089 | GL_Gan1_GLEAN_10008110 | 2.12 | 0.235 | 0.555 | 0.935 | 1.05 |
| GL_Gan1_GLEAN_10006089 | GL_Gan1_GLEAN_10006245 | 4.26 | 1.19 | 1.33 | 1.82 | 1.13 |
| GL_Gan1_GLEAN_10006089 | GL_Gan1_GLEAN_10007887 | 28.825 | 84.87 | 116.305 | 97.2 | 139.14 |
| GL_Gan1_GLEAN_10006089 | GL_Gan1_GLEAN_10008622 | 22.655 | 22.345 | 12.57 | 12.935 | 24.44 |
| Cluster:31 |  |  |  |  |  |  |
| GL_Gan1_GLEAN_10006137 | GL_Gan1_GLEAN_10006137 | 1.82 | 3.325 | 3.845 | 2.405 | 5.17 |
| GL_Gan1_GLEAN_10006137 | GL_Gan1_GLEAN_10007942 | 0 | 1.81 | 1.45 | 1.83 | 3.17 |
| GL_Gan1_GLEAN_10006137 | GL_Gan1_GLEAN_10008397 | 30.33 | 30.885 | 31.74 | 35.45 | 44.775 |
| GL_Gan1_GLEAN_10006137 | GL_Gan1_GLEAN_10006641 | 95.825 | 94.045 | 98.545 | 109.03 | 93.24 |
| GL_Gan1_GLEAN_10006137 | GL_Gan1_GLEAN_10006966 | 99.975 | 108.69 | 133.03 | 123.52 | 129.705 |
| GL_Gan1_GLEAN_10006137 | GL_Gan1_GLEAN_10007726 | 0.755 | 2.315 | 0.92 | 1.205 | 3.8 |
| GL_Gan1_GLEAN_10006137 | GL_Gan1_GLEAN_10008676 | 0 | 3.385 | 5.325 | 4.4 | 8.43 |
| GL_Gan1_GLEAN_10006137 | GL_Gan1_GLEAN_10007148 | 3.025 | 8.625 | 5.685 | 5.52 | 11.18 |
| GL_Gan1_GLEAN_10006137 | GL_Gan1_GLEAN_10008675 | 0 | 3.87 | 2.845 | 1.295 | 4.275 |
| Cluster:32 |  |  |  |  |  |  |
| GL_Gan1_GLEAN_10006155 | GL_Gan1_GLEAN_10004032 | 14.3 | 0 | 0.37 | 0.345 | 0.25 |
| GL_Gan1_GLEAN_10006155 | GL_Gan1_GLEAN_10002125 | 47.24 | 0.46 | 1.51 | 1.27 | 0.575 |
| GL_Gan1_GLEAN_10006155 | GL_Gan1_GLEAN_10006503 | 267.855 | 233.41 | 281.35 | 269.47 | 174.43 |
| GL_Gan1_GLEAN_10006155 | GL_Gan1_GLEAN_10002678 | 0 | 0 | 0.36 | 0.335 | 0.45 |
| GL_Gan1_GLEAN_10006155 | GL_Gan1_GLEAN_10000212 | 109.19 | 111.31 | 72.34 | 59.71 | 118.12 |
| GL_Gan1_GLEAN_10006155 | GL_Gan1_GLEAN_10005193 | 0 | 2.825 | 2.965 | 3.175 | 2.245 |
| GL_Gan1_GLEAN_10006155 | GL_Gan1_GLEAN_10006155 | 1.39 | 1.33 | 1.22 | 1.19 | 1.35 |
| GL_Gan1_GLEAN_10006155 | GL_Gan1_GLEAN_10006997 | 0 | 14.465 | 5 | 5.12 | 12.25 |
| GL_Gan1_GLEAN_10006155 | GL_Gan1_GLEAN_10001279 | 0 | 0 | 0 | 0 | 0.46 |
| GL_Gan1_GLEAN_10006155 | GL_Gan1_GLEAN_10003239 | 9.3 | 5.205 | 4.72 | 4.925 | 5.525 |
| Cluster:33 |  |  |  |  |  |  |
| GL_Gan1_GLEAN_10008054 | GL_Gan1_GLEAN_10008054 | 12.3 | 19.67 | 8.805 | 6.86 | 205 |
| GL_Gan1_GLEAN_10008054 | GL_Gan1_GLEAN_10004373 | 0 | 1.02 | 1.475 | 0.815 | 0 |
| GL_Gan1_GLEAN_10008054 | GL_Gan1_GLEAN_10004073 | 5.08 | 13.44 | 8.22 | 4.81 | 21.805 |
| Cluster:34 |  |  |  |  |  |  |
| GL_Gan1_GLEAN_10008757 | GL_Gan1_GLEAN_10008757 | 29.875 | 7.995 | 9 | 5.245 | 6.945 |
| GL_Gan1_GLEAN_10008757 | GL_Gan1_GLEAN_10008467 | 147.53 | 56.61 | 82.91 | 79.655 | 77.37 |
| GL_Gan1_GLEAN_10008757 | GL_Gan1_GLEAN_10009066 | 125.76 | 73.3 | 131.53 | 143.255 | 84.06 |
| GL_Gan1_GLEAN_10008757 | GL_Gan1_GLEAN_10008756 | 0 | 2.205 | 4.035 | 2.275 | 0.795 |
| GL_Gan1_GLEAN_10008757 | GL_Gan1_GLEAN_10009065 | 134.915 | 92.83 | 67.225 | 77.155 | 76.975 |
| Cluster:35 |  |  |  |  |  |  |
| GL_Gan1_GLEAN_10008848 | GL_Gan1_GLEAN_10008848 | 60.045 | 13.635 | 10.625 | 14.305 | 11.26 |
| GL_Gan1_GLEAN_10008848 | GL_Gan1_GLEAN_10001084 | 49.825 | 72.345 | 101.54 | 88.705 | 63.01 |
| GL_Gan1_GLEAN_10008848 | GL_Gan1_GLEAN_10003102 | 39.715 | 36.525 | 63.44 | 64.975 | 54.885 |
| GL_Gan1_GLEAN_10008848 | GL_Gan1_GLEAN_10003324 | 140.355 | 338.44 | 192.64 | 174.55 | 353.64 |
| Cluster:36 |  |  |  |  |  |  |
| GL_Gan1_GLEAN_10009124 | GL_Gan1_GLEAN_10009127 | 9.54 | 43.575 | 124.03 | 100.945 | 37.43 |
| GL_Gan1_GLEAN_10009124 | GL_Gan1_GLEAN_10008804 | 15.43 | 7.415 | 11.385 | 7.53 | 8.735 |
| GL_Gan1_GLEAN_10009124 | GL_Gan1_GLEAN_10009126 | 0 | 10.465 | 27.945 | 22.38 | 8.73 |
| GL_Gan1_GLEAN_10009124 | GL_Gan1_GLEAN_10008803 | 11.7 | 39.42 | 26.925 | 26.545 | 45.23 |
| GL_Gan1_GLEAN_10009124 | GL_Gan1_GLEAN_10009125 | 7.03 | 57.55 | 85.89 | 111.675 | 58.885 |
| GL_Gan1_GLEAN_10009124 | GL_Gan1_GLEAN_10008802 | 111.995 | 240.45 | 218.93 | 233.695 | 223.125 |
| GL_Gan1_GLEAN_10009124 | GL_Gan1_GLEAN_10009412 | 0 | 0 | 0 | 0 | 0 |
| GL_Gan1_GLEAN_10009124 | GL_Gan1_GLEAN_10009124 | 10.62 | 16.15 | 30.965 | 28.31 | 26.835 |
| GL_Gan1_GLEAN_10009124 | GL_Gan1_GLEAN_10008801 | 0.175 | 0.08 | 0.055 | 0.165 | 0.045 |
| GL_Gan1_GLEAN_10009124 | GL_Gan1_GLEAN_10009123 | 1.09 | 5.745 | 5.54 | 8.26 | 5.215 |
| GL_Gan1_GLEAN_10009124 | GL_Gan1_GLEAN_10008800 | 0.56 | 9.315 | 21 | 27.57 | 6.75 |
| GL_Gan1_GLEAN_10009124 | GL_Gan1_GLEAN_10009122 | 248.89 | 311.5 | 122.395 | 104.97 | 193.155 |
| GL_Gan1_GLEAN_10009124 | GL_Gan1_GLEAN_10009121 | 0 | 0 | 0.615 | 0.385 | 0 |
| GL_Gan1_GLEAN_10009124 | GL_Gan1_GLEAN_10008799 | 202.745 | 128.155 | 104.63 | 104.545 | 148.115 |
| GL_Gan1_GLEAN_10009124 | GL_Gan1_GLEAN_10008517 | 0 | 0 | 0 | 0 | 0 |
| GL_Gan1_GLEAN_10009124 | GL_Gan1_GLEAN_10009120 | 108.85 | 196.15 | 159.495 | 187.92 | 182.135 |
| GL_Gan1_GLEAN_10009124 | GL_Gan1_GLEAN_10008798 | 8.99 | 70.21 | 9.6 | 24.28 | 54.23 |

**(F)** Speciation specific genes

| Gene_ID | Expression Means | | | | |
| --- | --- | --- | --- | --- | --- |
|  | GL0d | GL1d | GL3d | GL5d | GL10d |
| GL_Gan1_GLEAN_10000001 | 0 | 0 | 0 | 0 | 0 |
| GL_Gan1_GLEAN_10000024 | 0 | 0 | 0 | 0 | 0 |
| GL_Gan1_GLEAN_10000036 | 0 | 0 | 0 | 0 | 0 |
| GL_Gan1_GLEAN_10000072 | 0 | 0 | 0 | 0 | 0 |
| GL_Gan1_GLEAN_10000074 | 0 | 0 | 0 | 0 | 0 |
| GL_Gan1_GLEAN_10000076 | 0 | 0 | 0 | 0 | 0 |
| GL_Gan1_GLEAN_10000077 | 0 | 0 | 0 | 0 | 0 |
| GL_Gan1_GLEAN_10000092 | 235.035 | 17.01 | 15.57 | 18.44 | 3.32 |
| GL_Gan1_GLEAN_10000105 | 0 | 0 | 0 | 0 | 0 |
| GL_Gan1_GLEAN_10000131 | 4.385 | 10.615 | 15.77 | 19.33 | 9.255 |
| GL_Gan1_GLEAN_10000143 | 0 | 0 | 0 | 0 | 0 |
| GL_Gan1_GLEAN_10000240 | 0 | 0 | 0 | 0 | 0 |
| GL_Gan1_GLEAN_10000241 | 0 | 0 | 0 | 0 | 0 |
| GL_Gan1_GLEAN_10000250 | 0 | 0 | 0 | 0 | 0 |
| GL_Gan1_GLEAN_10000266 | 0 | 0 | 0 | 0 | 0 |
| GL_Gan1_GLEAN_10000436 | 0 | 0 | 0.175 | 0.16 | 0 |
| GL_Gan1_GLEAN_10000548 | 0 | 0 | 0.22 | 0.605 | 0 |
| GL_Gan1_GLEAN_10000671 | 0 | 0 | 0 | 0 | 0 |
| GL_Gan1_GLEAN_10000721 | 0 | 0 | 0 | 0 | 0 |
| GL_Gan1_GLEAN_10000894 | 113.96 | 71.055 | 97.595 | 58.1 | 47.615 |
| GL_Gan1_GLEAN_10001144 | 0 | 0 | 0 | 0 | 0 |
| GL_Gan1_GLEAN_10001408 | 0 | 0 | 0 | 0 | 0 |
| GL_Gan1_GLEAN_10001463 | 6.335 | 136.59 | 13.57 | 7.975 | 144.935 |
| GL_Gan1_GLEAN_10001585 | 0 | 0 | 0 | 0 | 0 |
| GL_Gan1_GLEAN_10001834 | 5.39 | 7.165 | 3.41 | 3.65 | 7.49 |
| GL_Gan1_GLEAN_10001878 | 0 | 6.23 | 5.645 | 3.7 | 6.515 |
| GL_Gan1_GLEAN_10002055 | 13.945 | 39.995 | 72.83 | 80.46 | 68.28 |
| GL_Gan1_GLEAN_10002214 | 0 | 5.89 | 5.01 | 6.32 | 5.995 |
| GL_Gan1_GLEAN_10002215 | 0 | 0 | 0 | 0.235 | 0 |
| GL_Gan1_GLEAN_10002220 | 28.715 | 33.335 | 35.515 | 37.72 | 29.67 |
| GL_Gan1_GLEAN_10002333 | 178.51 | 12.88 | 23.325 | 11.16 | 9.115 |
| GL_Gan1_GLEAN_10002515 | 0 | 0 | 0 | 0.27 | 0.69 |
| GL_Gan1_GLEAN_10002799 | 0 | 1.905 | 0.825 | 1.145 | 0.7 |
| GL_Gan1_GLEAN_10002969 | 28.51 | 0 | 0.32 | 0.595 | 0 |
| GL_Gan1_GLEAN_10003054 | 32.995 | 86.775 | 70.155 | 83.935 | 72.8 |
| GL_Gan1_GLEAN_10003078 | 69.525 | 62.635 | 70.95 | 68.52 | 45.575 |
| GL_Gan1_GLEAN_10003179 | 24.265 | 3.105 | 0.54 | 0.255 | 2.02 |
| GL_Gan1_GLEAN_10003337 | 0 | 14.61 | 7.985 | 6.535 | 7.925 |
| GL_Gan1_GLEAN_10003368 | 0 | 0 | 0.24 | 0 | 0 |
| GL_Gan1_GLEAN_10003404 | 102.245 | 37.995 | 31.075 | 27.315 | 26.33 |
| GL_Gan1_GLEAN_10003419 | 21.085 | 0 | 4.185 | 2.99 | 2.865 |
| GL_Gan1_GLEAN_10003740 | 0 | 0 | 0.565 | 0 | 0 |
| GL_Gan1_GLEAN_10003789 | 0 | 0 | 0.17 | 0.185 | 0.49 |
| GL_Gan1_GLEAN_10004046 | 0 | 0 | 0 | 0 | 0 |
| GL_Gan1_GLEAN_10004076 | 0 | 0 | 1.185 | 0 | 0.595 |
| GL_Gan1_GLEAN_10004088 | 0 | 0 | 0 | 0 | 0 |
| GL_Gan1_GLEAN_10004106 | 0 | 0 | 0.42 | 0.215 | 0 |
| GL_Gan1_GLEAN_10004135 | 24.85 | 89.725 | 155.035 | 200.045 | 181.29 |
| GL_Gan1_GLEAN_10004445 | 338.985 | 1050.115 | 1381.95 | 1286.385 | 848.66 |
| GL_Gan1_GLEAN_10004857 | 0 | 3.26 | 3.825 | 0.325 | 2.93 |
| GL_Gan1_GLEAN_10004947 | 0 | 0.945 | 2.12 | 1.515 | 1.88 |
| GL_Gan1_GLEAN_10004973 | 0 | 2.71 | 2.325 | 1.085 | 0.985 |
| GL_Gan1_GLEAN_10005298 | 6.045 | 0 | 1.15 | 0.535 | 0.725 |
| GL_Gan1_GLEAN_10005544 | 0 | 0 | 0 | 0 | 0 |
| GL_Gan1_GLEAN_10005600 | 0 | 3.685 | 2.245 | 1.78 | 1.2 |
| GL_Gan1_GLEAN_10005766 | 9.73 | 1.07 | 8.055 | 5.85 | 3.09 |
| GL_Gan1_GLEAN_10005999 | 0 | 2.745 | 0.685 | 1.1 | 1.485 |
| GL_Gan1_GLEAN_10006048 | 0 | 0 | 0 | 0 | 0 |
| GL_Gan1_GLEAN_10006142 | 10.45 | 15.41 | 13.6 | 18.285 | 21.75 |
| GL_Gan1_GLEAN_10006385 | 0 | 0 | 0 | 0.305 | 1.105 |
| GL_Gan1_GLEAN_10006466 | 0 | 0 | 0 | 0 | 0 |
| GL_Gan1_GLEAN_10006524 | 3.355 | 9.59 | 11.325 | 13.615 | 10.785 |
| GL_Gan1_GLEAN_10006821 | 23.225 | 4.25 | 5.865 | 5.595 | 1.845 |
| GL_Gan1_GLEAN_10006858 | 0 | 0 | 0 | 0 | 0 |
| GL_Gan1_GLEAN_10007109 | 0 | 0.93 | 1.52 | 0.185 | 0 |
| GL_Gan1_GLEAN_10007150 | 18.02 | 1.5 | 2.77 | 1.365 | 0.55 |
| GL_Gan1_GLEAN_10007217 | 0 | 0 | 0.17 | 0 | 0 |
| GL_Gan1_GLEAN_10007287 | 4.115 | 5.475 | 8.84 | 12.08 | 10.785 |
| GL_Gan1_GLEAN_10008031 | 0 | 0 | 1.655 | 0.82 | 1.08 |
| GL_Gan1_GLEAN_10008158 | 0 | 3.13 | 0.23 | 0.425 | 0 |
| GL_Gan1_GLEAN_10008182 | 37.03 | 27.215 | 53.415 | 54.105 | 37.21 |
| GL_Gan1_GLEAN_10008302 | 18.8 | 1.925 | 2.43 | 3.895 | 1.74 |
| GL_Gan1_GLEAN_10008354 | 0 | 0.95 | 0 | 0.755 | 0 |
| GL_Gan1_GLEAN_10008488 | 35.88 | 16.28 | 21.15 | 21.835 | 11.34 |
| GL_Gan1_GLEAN_10008500 | 0 | 35.02 | 11.46 | 17.825 | 39.265 |
| GL_Gan1_GLEAN_10008517 | 0 | 0 | 0 | 0 | 0 |
| GL_Gan1_GLEAN_10008579 | 12.165 | 4.355 | 4.26 | 4.02 | 6.25 |
| GL_Gan1_GLEAN_10008635 | 267.405 | 95.645 | 103.375 | 76.635 | 59.48 |
| GL_Gan1_GLEAN_10008735 | 0 | 0 | 0 | 0 | 0 |
| GL_Gan1_GLEAN_10008755 | 0 | 0 | 0 | 0.095 | 0.345 |
| GL_Gan1_GLEAN_10008764 | 0 | 0 | 0 | 0 | 0 |
| GL_Gan1_GLEAN_10008927 | 11.285 | 2.07 | 1.555 | 3.15 | 3.235 |
| GL_Gan1_GLEAN_10009122 | 248.89 | 311.5 | 122.395 | 104.97 | 193.155 |
| GL_Gan1_GLEAN_10009126 | 0 | 10.465 | 27.945 | 22.38 | 8.73 |
| GL_Gan1_GLEAN_10009131 | 0 | 0 | 0 | 0 | 0 |
