# Supplementary material for: New insights into the evolution of host specificity of three Penicillium species and the pathogenicity of P. Italicum involving the infection of Valencia orange (Citrus sinensis)
Source: Virulence. 2020 Jun 11;11(1):748–68. doi: 10.1080/21505594.2020.1773038 (PMC7549954; doi:10.1080/21505594.2020.1773038)
Supplement: Supplemental Material [file KVIR_A_1773038_SM2584.zip › Figure S1.pdf]

FIG S1

A

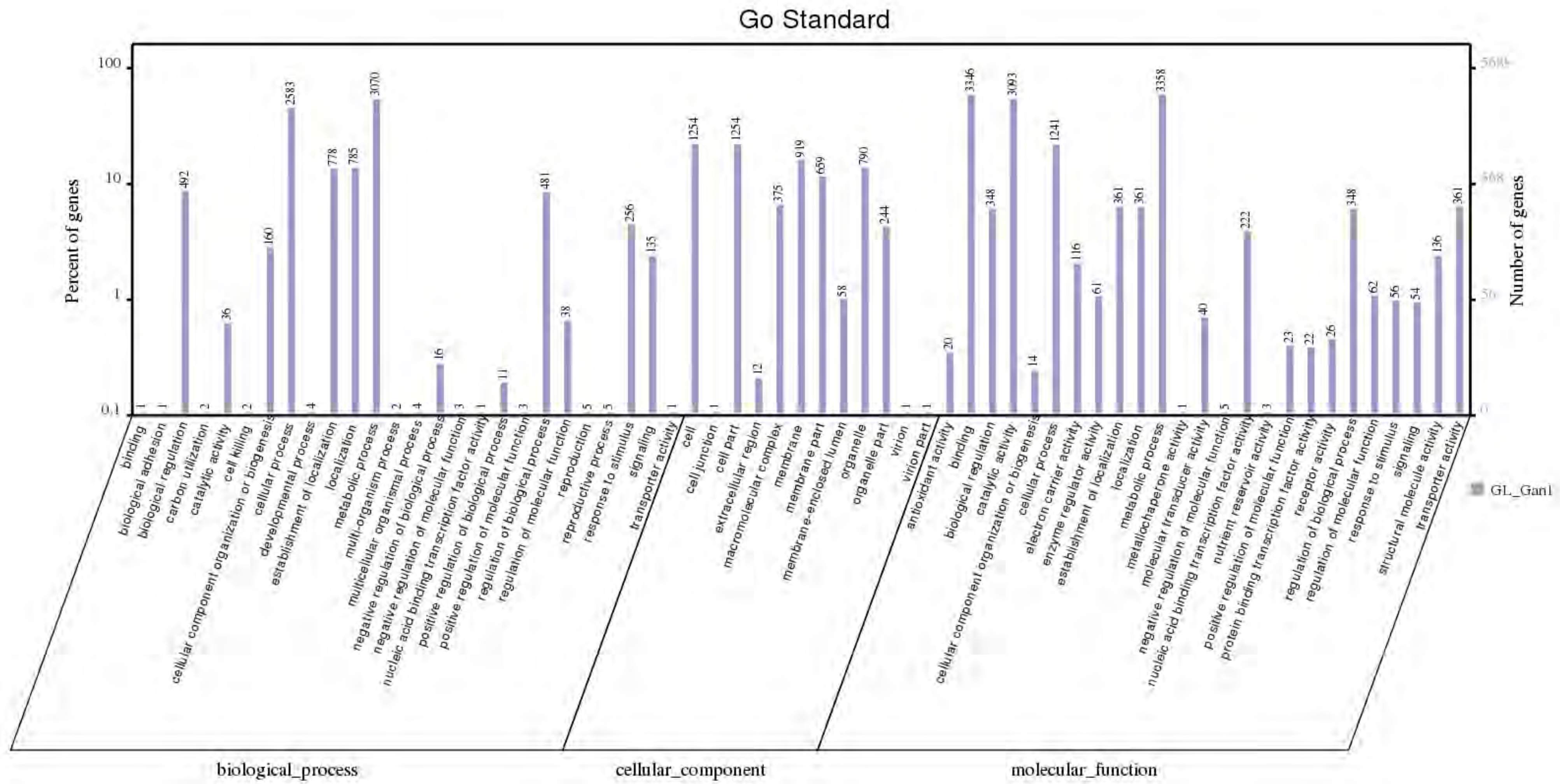

Go Standard
Percent of genes
Number of genes
GL_Gan1
biological_process
cellular_component
molecular_function

B

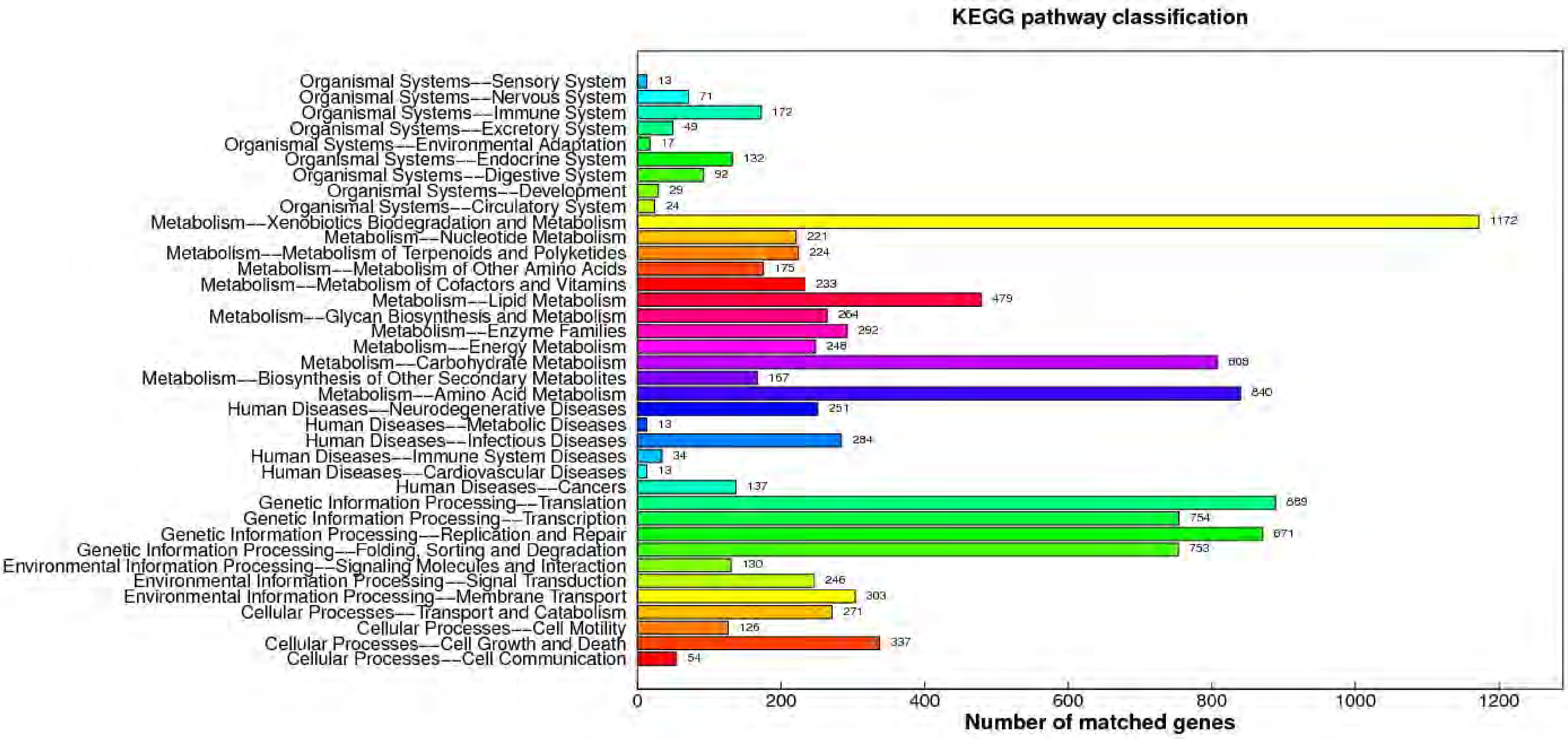
KEGG pathway classification
Organismal Systems--Sensory System 13
Organismal Systems--Nervous System 71
Organismal Systems--Immune System 172
Organismal Systems--Excretory System 49
Organismal Systems--Environmental Adaptation 17
Organismal Systems--Endocrine System 132
Organismal Systems--Digestive System 92
Organismal Systems--Development 29
Organismal Systems--Circulatory System 24
Metabolism--Xenobiotics Biodegradation and Metabolism 1172
Metabolism--Nucleotide Metabolism 221
Metabolism--Metabolism of Terpenoids and Polyketides 224
Metabolism--Metabolism of Other Amino Acids 175
Metabolism--Metabolism of Cofactors and Vitamins 233
Metabolism--Lipid Metabolism 479
Metabolism--Glycan Biosynthesis and Metabolism 264
Metabolism--Enzyme Families 292
Metabolism--Energy Metabolism 248
Metabolism--Carbohydrate Metabolism 808
Metabolism--Biosynthesis of Other Secondary Metabolites 167
Metabolism--Amino Acid Metabolism 840
Human Diseases--Neurodegenerative Diseases 251
Human Diseases--Metabolic Diseases 13
Human Diseases--Infectious Diseases 284
Human Diseases--Immune System Diseases 34
Human Diseases--Cardiovascular Diseases 13
Human Diseases--Cancers 137
Genetic Information Processing--Translation 889
Genetic Information Processing--Transcription 754
Genetic Information Processing--Replication and Repair 871
Genetic Information Processing--Folding, Sorting and Degradation 753
Environmental Information Processing--Signaling Molecules and Interaction 130
Environmental Information Processing--Signal Transduction 246
Environmental Information Processing--Membrane Transport 303
Cellular Processes--Transport and Catabolism 271
Cellular Processes--Cell Motility 126
Cellular Processes--Cell Growth and Death 337
Cellular Processes--Cell Communication 54
0 200 400 600 800 1000 1200
Number of matched genes

C

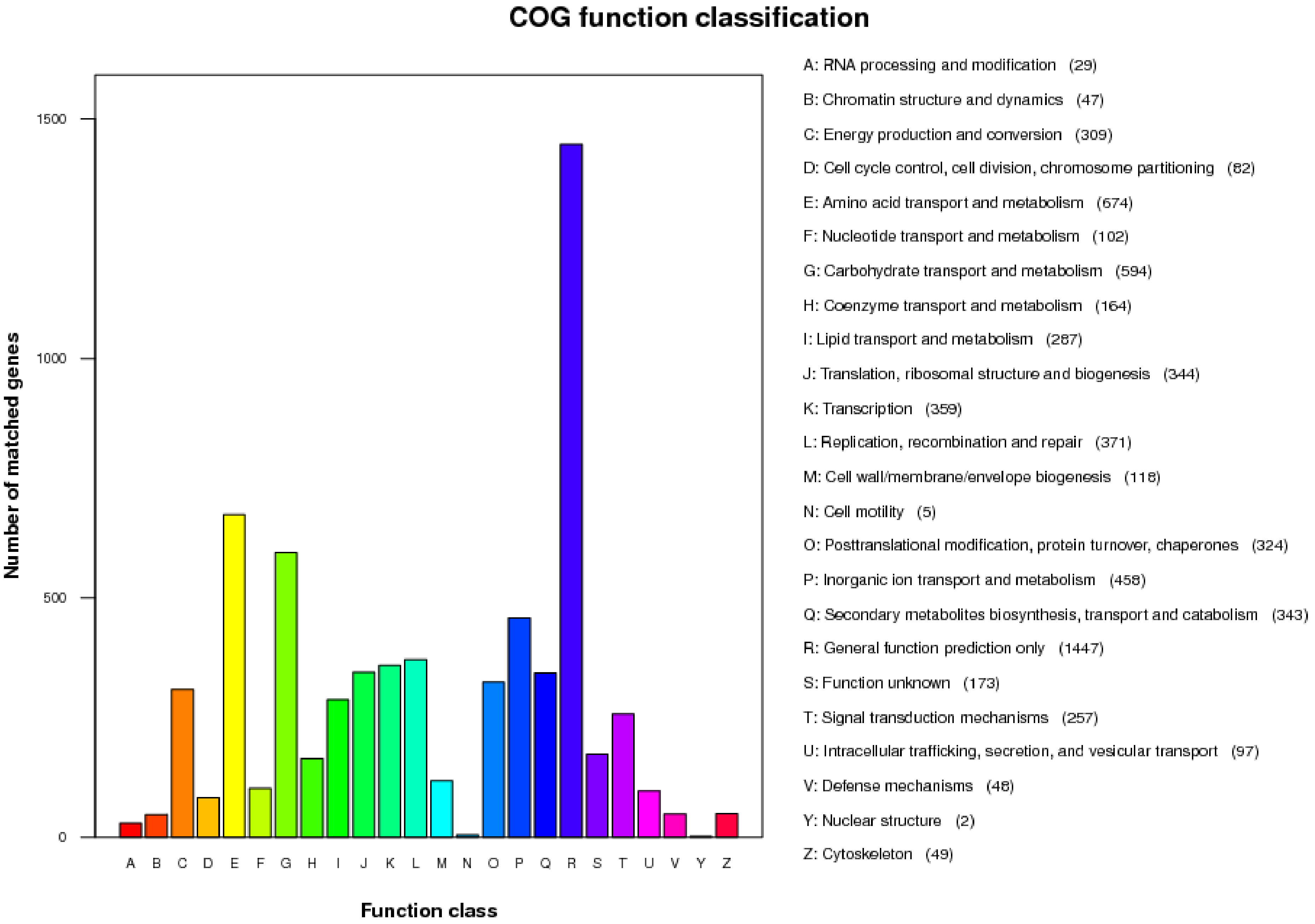
COG function classification
Number of matched genes
0
500
1000
1500
A B C D E F G H I J K L M N O P Q R S T U V Y Z
Function class
A: RNA processing and modification (29)
B: Chromatin structure and dynamics (47)
C: Energy production and conversion (309)
D: Cell cycle control, cell division, chromosome partitioning (82)
E: Amino acid transport and metabolism (674)
F: Nucleotide transport and metabolism (102)
G: Carbohydrate transport and metabolism (594)
H: Coenzyme transport and metabolism (164)
I: Lipid transport and metabolism (287)
J: Translation, ribosomal structure and biogenesis (344)
K: Transcription (359)
L: Replication, recombination and repair (371)
M: Cell wall/membrane/envelope biogenesis (118)
N: Cell motility (5)
O: Posttranslational modification, protein turnover, chaperones (324)
P: Inorganic ion transport and metabolism (458)
Q: Secondary metabolites biosynthesis, transport and catabolism (343)
R: General function prediction only (1447)
S: Function unknown (173)
T: Signal transduction mechanisms (257)
U: Intracellular trafficking, secretion, and vesicular transport (97)
V: Defense mechanisms (48)
Y: Nuclear structure (2)
Z: Cytoskeleton (49)
