# Supplementary material for: New insights into the evolution of host specificity of three Penicillium species and the pathogenicity of P. Italicum involving the infection of Valencia orange (Citrus sinensis)
Source: Virulence. 2020 Jun 11;11(1):748–68. doi: 10.1080/21505594.2020.1773038 (PMC7549954; doi:10.1080/21505594.2020.1773038)
Supplement: Supplemental Material [file KVIR_A_1773038_SM2584.zip › Table S1.docx]

**Table S1** Repeat elements in the *Penicillium* genome

|  | | CMP1 | MD8 | Pd1 | Gan1 | PHI1 | PHI26 |
| --- | --- | --- | --- | --- | --- | --- | --- |
|  | |  |  |  |  |  |  |
| DNA | length | 73,691 | 266,333 | 243,400 | 249,398 | 244,397 | 286,269 |
|  | % in Genome | 0.2370 | 0.8231 | 0.7591 | 0.8036 | 0.8102 | 1.1167 |
| LINE | length | 32,360 | 62,761 | 39,292 | 120,354 | 173,187 | 156,259 |
|  | % in Genome | 0.1041 | 0.1940 | 0.1225 | 0.3878 | 0.5741 | 0.6096 |
| LTR | length | 78,374 | 206,881 | 130,350 | 1,336,262 | 284,021 | 358,670 |
|  | % in Genome | 0.2521 | 0.6394 | 0.4065 | 4.3057 | 0.9416 | 1.3992 |
| SINE | length | 5,151 | 8,941 | 10,091 | 7,049 | 7,825 | 23,022 |
|  | % in Genome | 0.0166 | 0.0276 | 0.0315 | 0.0227 | 0.0259 | 0.0898 |
| Other | length | 0 | 0 | 0 | 0 | 63 | 0 |
|  | % in Genome | 0.0000 | 0.0000 | 0.0000 | 0.0000 | 0.0002 | 0.0000 |
| Unknown | length | 184,141 | 721,433 | 655,151 | 2,049,958 | 1,484,239 | 263,855 |
|  | % in Genome | 0.5923 | 2.2297 | 2.0432 | 6.6054 | 4.9205 | 1.0293 |
| Total | length | 370,855 | 1,191,357 | 1,027,222 | 3,539,147 | 2,122,116 | 1,030,460 |
|  | % in Genome | 1.1930 | 3.682 | 3.2036 | 11.4039 | 7.0352 | 4.0198 |

DNA, DNA transposon; LINE, long interspersed element; LTR, long terminal repeat; SINE, short interspersed elements
