# Supplementary material for: New insights into the evolution of host specificity of three Penicillium species and the pathogenicity of P. Italicum involving the infection of Valencia orange (Citrus sinensis)
Source: Virulence. 2020 Jun 11;11(1):748–68. doi: 10.1080/21505594.2020.1773038 (PMC7549954; doi:10.1080/21505594.2020.1773038)
Supplement: Supplemental Material [file KVIR_A_1773038_SM2584.zip › Table S2.docx]

**Table S2** Details regarding the transcriptome data after mapping to the *Penicillium italicum* genome and the [Valencia orange](http://www.baidu.com/link?url=kHVVlo3thXkUrFrCgTimkoQHEzkfOO8xSGZies5qPeTcuPmUgL2pY0V7MFatpW8-ubOSAuHW9EqLBBz7UDz94xKdXX7bUJ7QwU2XbpJF9qv5OgDPWeSrQY0GWgTt-ikv&wd=&eqid=a3352eeb0000d7b0000000065c9c2b87) genome

| Sample Name | Clean reads | Genome map Rate | Gene map Rate | Expressed Gene |
| --- | --- | --- | --- | --- |
| *Penicillium italicum* | | | | |
| GL_day0A | 42576567 | 2.37% | 1.87% | 7993 |
| GL_day0B | 43428647 | 0.61% | 0.43% | 6840 |
| GL_day1A | 21257317 | 21.92% | 16.94% | 8888 |
| GL_day1B | 22142357 | 19.55% | 16.30% | 8539 |
| GL_day3A | 30391376 | 70.30% | 55.05% | 9145 |
| GL_day3B | 32314471 | 37.97% | 30.61% | 8965 |
| GL_day5A | 41827951 | 57.27% | 43.29% | 9193 |
| GL_day5B | 44244394 | 49.36% | 39.89% | 9040 |
| GL_day10A | 32054085 | 18.97% | 15.75% | 8750 |
| GL_day10B | 32098325 | 26.86% | 20.95% | 8896 |
| [Valencia orange](http://www.baidu.com/link?url=kHVVlo3thXkUrFrCgTimkoQHEzkfOO8xSGZies5qPeTcuPmUgL2pY0V7MFatpW8-ubOSAuHW9EqLBBz7UDz94xKdXX7bUJ7QwU2XbpJF9qv5OgDPWeSrQY0GWgTt-ikv&wd=&eqid=a3352eeb0000d7b0000000065c9c2b87) | | | | |
| GL_day0A | 42576567 | 79.44% | 84.33% | 21896 |

| GL_day0B | 43428647 | 82.82% | 88.03% | 21668 |
| --- | --- | --- | --- | --- |
| GL_day1A | 21257317 | 30.19% | 30.58% | 17990 |
| GL_day1B | 22142357 | 64.49% | 67.92% | 20923 |

| GL_day3A | 30391376 | 12.07% | 12.46% | 17610 |
| --- | --- | --- | --- | --- |
| GL_day3B | 32314471 | 44.14% | 45.59% | 19979 |
| GL_day5A | 41827951 | 21.00% | 21.74% | 18874 |
| GL_day5B | 44244394 | 23.35% | 23.83% | 19147 |
| GL_day10A | 32054085 | 63.43% | 66.77% | 21317 |
| GL_day10B | 32098325 | 38.65% | 39.48% | 19507 |
