# Supplementary material for: New insights into the evolution of host specificity of three Penicillium species and the pathogenicity of P. Italicum involving the infection of Valencia orange (Citrus sinensis)
Source: Virulence. 2020 Jun 11;11(1):748–68. doi: 10.1080/21505594.2020.1773038 (PMC7549954; doi:10.1080/21505594.2020.1773038)
Supplement: Supplemental Material [file KVIR_A_1773038_SM2584.zip › Table S3.docx]

**Table S3** [Valencia orange](http://www.baidu.com/link?url=kHVVlo3thXkUrFrCgTimkoQHEzkfOO8xSGZies5qPeTcuPmUgL2pY0V7MFatpW8-ubOSAuHW9EqLBBz7UDz94xKdXX7bUJ7QwU2XbpJF9qv5OgDPWeSrQY0GWgTt-ikv&wd=&eqid=a3352eeb0000d7b0000000065c9c2b87) *R* genes

| Disease_resistance_domain_Type | Number | ID |
| --- | --- | --- |
| CC | 21 | Cs5g01810,Cs7g10400,Cs1g04140,Cs5g01810,orange1.1t01531,Cs7g14810,Cs7g26130,Cs5g01810,Cs5g28990,Cs7g12380,Cs7g14780,orange1.1t03659,Cs5g28990,Cs1g07720,orange1.1t01364,Cs1g07800,Cs5g28630,Cs1g07910,Cs7g12380,Cs5g28990,Cs4g10850 |
| CC-NBS | 260 | Cs1g07860,Cs3g11010,Cs3g03980,orange1.1t03742,orange1.1t02481,Cs3g06480,Cs1g17000,Cs3g06590,Cs1g09470,Cs1g24520,Cs3g06480,Cs2g26460,Cs1g07240,orange1.1t04603,Cs4g11300,orange1.1t05161,Cs3g07960,orange1.1t04652,Cs3g15220,Cs4g08100,Cs6g02120,Cs1g14090,orange1.1t04094,Cs1g03690,Cs6g02100,Cs1g17140,orange1.1t04588,Cs3g06650,Cs3g12330,orange1.1t04831,orange1.1t01356,orange1.1t03704,Cs8g07740,Cs3g13320,Cs1g23070,Cs6g03920,orange1.1t05048,Cs3g03540,orange1.1t04824,Cs1g16990,Cs3g15220,Cs6g05240,Cs1g07220,Cs1g10960,Cs3g01180,Cs6g06810,Cs1g07160,Cs3g13710,Cs3g13730,Cs3g08110,orange1.1t04656,orange1.1t01926,Cs3g04930,Cs8g09140,Cs1g10490,Cs5g21150,orange1.1t03647,orange1.1t00796,Cs3g02960,Cs3g12700,Cs5g20580,Cs3g13430,Cs3g04930,orange1.1t03481,Cs3g12720,orange1.1t03739,Cs3g13180,Cs3g13430,orange1.1t03534,Cs1g01180,Cs3g13200,orange1.1t04584,Cs7g02220,orange1.1t02498,orange1.1t01898,Cs1g12140,Cs5g21040,orange1.1t03707,Cs3g04930,orange1.1t03640,orange1.1t02473,orange1.1t05295,Cs1g01880,orange1.1t03126,orange1.1t04653,orange1.1t05176,Cs1g16990,Cs4g08110,Cs6g02130,orange1.1t04833,orange1.1t05289,orange1.1t02481,orange1.1t03732,Cs6g05250,Cs3g12710,orange1.1t03734,Cs5g21120,orange1.1t04084,Cs1g18380,Cs2g18480,orange1.1t03736,Cs1g07850,Cs6g14690,orange1.1t04659,Cs3g13060,Cs3g13210,Cs3g13160,orange1.1t01918,Cs3g06500,Cs3g08140,orange1.1t04591,Cs1g18370,Cs5g20560,Cs3g13390,Cs3g11150,Cs1g17000,Cs3g03020,orange1.1t03648,Cs5g21980,Cs8g07890,Cs4g07730,Cs3g11210,orange1.1t02493,Cs8g07700,Cs5g28770,Cs3g11110,orange1.1t02495,Cs6g01040,orange1.1t03486,orange1.1t02750,Cs1g13430,orange1.1t04515,orange1.1t04930,Cs3g13160,Cs4g08110,Cs4g08020,Cs1g14110,orange1.1t04486,Cs5g29510,orange1.1t02477,Cs3g11120,orange1.1t03742,Cs5g21020,Cs2g30580,orange1.1t01435,Cs8g07870,Cs3g06490,Cs3g15220,Cs3g13160,Cs1g01140,Cs3g13690,Cs5g22710,Cs3g11140,orange1.1t04088,Cs3g13210,Cs1g09380,Cs2g18470,orange1.1t04591,orange1.1t03487,orange1.1t03879,orange1.1t04522,orange1.1t04082,Cs1g09330,orange1.1t03874,Cs1g12880,Cs1g12880,Cs1g07160,orange1.1t04537,Cs3g06600,orange1.1t03709,orange1.1t01179,Cs6g02100,orange1.1t04603,Cs1g13410,Cs8g09080,Cs3g15200,orange1.1t05310,Cs1g07180,Cs1g12910,Cs1g09350,Cs5g28770,orange1.1t03487,Cs9g13350,Cs1g15550,orange1.1t04830,orange1.1t01359,Cs1g12210,Cs3g11130,Cs1g13050,Cs3g04020,Cs3g15220,Cs4g07730,Cs6g01020,Cs2g26400,Cs3g11190,Cs1g14090,Cs3g11050,orange1.1t02177,Cs3g11080,Cs1g14090,Cs4g08080,Cs3g06640,Cs3g03570,orange1.1t04211,Cs1g01890,orange1.1t04470,Cs1g13420,Cs1g18380,Cs6g03920,orange1.1t03487,Cs1g05730,orange1.1t04509,Cs2g30290,Cs1g11080,orange1.1t02848,orange1.1t05193,orange1.1t05345,Cs3g04030,Cs9g13120,orange1.1t02504,orange1.1t04078,orange1.1t05207,Cs8g07700,Cs1g12190,orange1.1t04526,Cs3g12760,Cs5g21130,Cs3g13340,Cs3g04930,Cs1g13090,Cs1g01160,Cs1g11430,Cs3g13740,Cs3g04910,orange1.1t01311,orange1.1t04079,Cs3g12340,Cs3g01280,Cs9g11290,Cs3g13250,Cs8g11620,orange1.1t04672,orange1.1t04654,Cs1g12940,Cs1g18380,Cs6g01070,Cs3g15220,orange1.1t04513,Cs3g12850,Cs1g09350,Cs5g21990,orange1.1t04592,Cs3g11160,Cs3g13060,orange1.1t05048,orange1.1t01363,Cs3g13210,Cs6g01080,Cs5g19240,orange1.1t04992 |
| CC-NBS-LRR | 54 | Cs1g10970,Cs9g13230,Cs9g13310,orange1.1t01362,Cs5g28645,Cs1g10460,Cs7g14510,Cs1g10970,Cs5g22030,Cs1g13380,Cs5g28645,Cs9g13250,Cs1g08080,Cs1g12920,Cs1g06720,Cs5g22030,Cs1g10970,Cs4g13040,Cs9g13200,Cs7g16030,Cs5g22000,Cs1g10450,Cs4g10860,Cs5g26190,orange1.1t02842,Cs1g13010,Cs1g10470,Cs1g11320,Cs5g22030,Cs1g10990,Cs7g14540,Cs3g16680,Cs1g08080,Cs3g05280,Cs1g11390,Cs5g26170,Cs7g16030,Cs5g22030,Cs1g11050,Cs1g13480,Cs1g12130,Cs5g22030,Cs5g22030,Cs1g13030,Cs7g16030,Cs4g10810,Cs1g13270,orange1.1t01365,Cs1g13070,Cs1g13460,Cs1g13100,Cs1g11410,Cs9g13300,Cs9g13290 |
| CC-TIR-NBS | 13 | orange1.1t04661,Cs3g05740,orange1.1t04294,orange1.1t04292,Cs3g05740,orange1.1t04301,Cs5g19430,orange1.1t01831,Cs3g05760,orange1.1t02159,Cs3g05770,Cs5g18450,Cs3g05740 |
| LRR | 1 | orange1.1t05191 |
| NBS | 121 | Cs3g05950,Cs3g24020,orange1.1t02175,Cs3g05560,Cs3g08210,Cs5g19510,Cs8g16490,Cs3g08000,Cs7g17260,Cs3g05700,Cs1g12050,orange1.1t01942,Cs1g17180,orange1.1t04582,Cs5g24270,orange1.1t04090,orange1.1t05226,Cs5g22090,Cs3g05520,orange1.1t02748,Cs3g05290,orange1.1t03880,Cs3g06060,Cs5g22330,orange1.1t02169,Cs8g12930,orange1.1t05328,Cs1g11730,Cs4g08140,Cs3g12840,Cs3g06010,orange1.1t04480,Cs5g22330,Cs3g05890,Cs3g05900,orange1.1t04929,Cs5g22010,Cs1g12170,orange1.1t04089,orange1.1t03137,Cs5g18280,orange1.1t03712,Cs5g22330,Cs1g07820,orange1.1t02169,Cs8g16500,orange1.1t01802,orange1.1t04520,Cs5g19410,Cs3g01320,Cs1g09370,orange1.1t02169,Cs5g19240,orange1.1t02518,Cs4g08050,Cs3g07940,Cs3g07940,Cs4g14260,orange1.1t04673,orange1.1t05652,Cs8g12970,orange1.1t01159,orange1.1t01815,Cs1g18980,Cs3g08250,Cs5g18420,Cs3g05870,orange1.1t04538,orange1.1t02478,orange1.1t04443,orange1.1t04083,orange1.1t04933,orange1.1t05262,Cs1g07710,orange1.1t04440,Cs1g17130,Cs5g20260,Cs1g18610,Cs7g26730,Cs5g20430,orange1.1t04568,Cs1g02140,Cs3g11030,Cs3g05940,orange1.1t03713,orange1.1t04517,orange1.1t04945,Cs8g07820,orange1.1t03646,Cs1g08330,Cs3g08040,Cs3g05570,orange1.1t03882,orange1.1t02163,orange1.1t05621,Cs1g13390,Cs4g07610,Cs1g17020,orange1.1t03332,orange1.1t02163,Cs3g05510,Cs3g05830,Cs1g07780,orange1.1t03656,Cs5g22730,Cs5g20390,Cs8g09010,orange1.1t01794,Cs3g13070,Cs1g07790,Cs4g08050,Cs1g14030,Cs3g12810,Cs3g01540,orange1.1t04562,Cs2g30120,Cs3g12320,Cs3g07970,Cs8g13030,orange1.1t02846,Cs1g12150 |
| NBS-LRR | 16 | Cs5g21975,Cs1g13470,Cs2g18460,orange1.1t02132,orange1.1t05257,orange1.1t02132,orange1.1t02132,Cs1g14120,Cs1g11070,Cs2g18460,Cs5g20500,Cs1g13400,Cs5g20470,orange1.1t02132,Cs1g10440,orange1.1t02165 |
| TIR | 3 | Cs8g10570,orange1.1t04662,orange1.1t0525, |
| TIR-NBS | 56 | Cs5g19230,orange1.1t01829,orange1.1t00678,Cs5g22420,orange1.1t01829,orange1.1t01829,orange1.1t01446,Cs2g30970,Cs2g30960,orange1.1t02171,Cs3g05820,orange1.1t04706,orange1.1t01807,Cs2g11020,orange1.1t01815,Cs5g18280,orange1.1t03701,orange1.1t03694,orange1.1t01834,Cs5g20360,orange1.1t00694,Cs1g14810,Cs5g19950,orange1.1t01844,Cs5g17870,Cs5g22400,Cs5g19220,Cs5g19930,Cs5g17280,Cs5g20270,Cs7g26750,Cs5g17280,orange1.1t04571,orange1.1t04750,Cs7g04730,Cs5g19470,orange1.1t04296,orange1.1t01844,Cs2g11000,orange1.1t04573,Cs1g22350,Cs5g19440,Cs5g19450,Cs5g19850,orange1.1t04913,Cs7g26750,orange1.1t02179,Cs5g24260,orange1.1t04291,orange1.1t02179,orange1.1t05139,Cs5g17280,Cs7g26730,Cs5g19460,orange1.1t04445, orange1.1t01829 |
| TIR-NBS-LRR | 24 | Cs5g18480,Cs1g18940,orange1.1t01823,orange1.1t03524,orange1.1t00681,Cs5g22380,orange1.1t03509,Cs5g22460,orange1.1t01822,orange1.1t03516,Cs5g22370,orange1.1t01812,orange1.1t00706,Cs5g19920,Cs5g11840,orange1.1t03510,Cs1g18970,orange1.1t02132,orange1.1t02140,orange1.1t03523,Cs5g23190,orange1.1t03524,orange1.1t03514,orange1.1t03702 |
| other | 58 | Cs7g26130,Cs4g16750,Cs4g04420,Cs6g19410,Cs2g13550,Cs2g30650,Cs9g18650,Cs7g28610,Cs7g26130,Cs1g19850,Cs2g13550,Cs1g13660,Cs1g18460,Cs3g26380,Cs5g33060,Cs7g09730,Cs9g18660,Cs1g02710,Cs7g10200,Cs5g33060,Cs7g11600,Cs4g04420,Cs5g02710,Cs6g19410,Cs7g12380,Cs6g19410,Cs3g26380,Cs1g19860,Cs2g30650,Cs1g13660,Cs1g18450,Cs1g02710,Cs1g13660,Cs3g09820,orange1.1t02845,orange1.1t02189,Cs5g19310,Cs5g19890,orange1.1t01795,Cs1g10910,Cs8g10560,Cs7g14790,orange1.1t01805,orange1.1t04289,Cs6g02150,orange1.1t04298,Cs5g19400,orange1.1t04286,Cs5g19890,Cs1g11040,Cs1g19000,Cs5g22440,Cs5g19880,orange1.1t04663,orange1.1t01837,orange1.1t01840, Cs5g18730,orange1.1t02161 |
| un-annotion | 9 | orange1.1t03654,Cs1g11090,Cs3g05930,orange1.1t05290,Cs1g12200,Cs5g18600,Cs3g05550,Cs3g02990,Cs5g18600 |
| total | 636 |  |
