# Supplementary material for: New insights into the evolution of host specificity of three Penicillium species and the pathogenicity of P. Italicum involving the infection of Valencia orange (Citrus sinensis)
Source: Virulence. 2020 Jun 11;11(1):748–68. doi: 10.1080/21505594.2020.1773038 (PMC7549954; doi:10.1080/21505594.2020.1773038)
Supplement: Supplemental Material [file KVIR_A_1773038_SM2584.zip › Table S4.docx]

**Table S4** Metabolites identified during the metabolome analysis

| Peak | Similarity | R.T. | Count | Mass |
| --- | --- | --- | --- | --- |
| 2-hydroxypyridine | 871.8621 | 6.88739,0 | 29 | 152 |
| lactic acid | 932.2069 | 7.19261,0 | 29 | 117 |
| glycolic acid | 932.8621 | 7.42509,0 | 29 | 147 |
| alanine 1 | 954.3448 | 7.78954,0 | 29 | 116 |
| hydroxylamine | 742.8214 | 7.95161,0 | 28 | 133 |
| sarcosine | 492.6207 | 8.33273,0 | 29 | 86 |
| Lactamide 2 | 386.0556 | 8.33963,0 | 18 | 175 |
| Analyte 104 | 251.3333 | 8.39944,0 | 21 | 125 |
| 3-Hydroxypropionic acid 1 | 886.2759 | 8.3775,0 | 29 | 177 |
| 3-Hydroxypyridine | 896.4828 | 8.49204,0 | 29 | 152 |
| 5-aminovaleric acid lactam | 329.5517 | 8.55187,0 | 29 | 142 |
| 3-hydroxybutyric acid | 813.5714 | 8.58872,0 | 28 | 147 |
| Lactamide 1 | 413.2759 | 8.77256,0 | 29 | 117 |
| N-Methyl-DL-alanine | 486.6071 | 8.80777,0 | 28 | 130 |
| Methyl Phosphate | 815.2222 | 8.90198,0 | 27 | 241 |
| succinate semialdehyde 2 | 534.3077 | 9.08315,0 | 14 | 151 |
| Methylmalonic acid | 610.8095 | 9.22158,0 | 29 | 281 |
| 2-Hydroxyvaleric acid | 707.1035 | 9.28517,0 | 29 | 131 |
| Norleucine 2 | 204.9231 | 9.32267,0 | 20 | 186 |
| valine | 837.4828 | 9.36279,0 | 29 | 144 |
| Acetophenone | 324 | 9.49782,0 | 18 | 227 |
| 4-hydroxybutyrate | 784.5517 | 9.66066,0 | 29 | 147 |
| 2-Butyne-1,4-diol | 587.6667 | 9.81199,0 | 28 | 147 |
| Dihydroxyacetone | 678.0385 | 9.8676,0 | 26 | 174 |
| benzoic acid | 964.2759 | 9.94721,0 | 29 | 179 |
| serine 2 | 965.6552 | 9.97457,0 | 29 | 132 |
| Ethanolamine | 916.8966 | 10.0738,0 | 29 | 86 |
| phosphate | 809.6 | 10.1533,0 | 25 | 205 |
| glycerol | 844.3 | 10.1662,0 | 20 | 117 |
| butyraldehyde 2 | 174 | 10.1909,0 | 29 | 138 |
| 2-Deoxyerythritol | 908.6897 | 10.3709,0 | 29 | 117 |
| threonine 2 | 892.5185 | 10.4776,0 | 28 | 117 |
| proline | 965.069 | 10.5038,0 | 29 | 142 |
| maleic acid | 525.7241 | 10.5501,0 | 29 | 190 |
| glycine 2 | 949.7586 | 10.6084,0 | 29 | 174 |
| succinic acid | 900.069 | 10.7358,0 | 29 | 56 |
| catechol | 359.1429 | 10.7778,0 | 8 | 254 |
| D-Glyceric acid | 936.8966 | 10.9103,0 | 29 | 189 |
| beta-hydroxypyruvate | 473.4118 | 10.9983,0 | 17 | 73 |
| Itaconic acid | 948.4138 | 11.1101,0 | 29 | 147 |
| Citraconic acid 4 | 931.6207 | 11.2003,0 | 29 | 147 |
| fumaric acid | 782.2414 | 11.233,0 | 29 | 245 |
| Pyrrole-2-Carboxylic Acid | 695.8 | 11.2406,0 | 10 | 240 |
| serine 1 | 911.3793 | 11.3197,0 | 29 | 204 |
| 3-Hydroxynorvaline 2 | 372.3333 | 11.3557,0 | 29 | 160 |
| Aminooxyacetic acid | 510.7143 | 11.5989,0 | 29 | 247 |
| L-Allothreonine 1 | 626.3214 | 11.6578,0 | 29 | 217 |
| resorcinol | 345.069 | 11.6746,0 | 29 | 240 |
| Tartronic acid | 469.4074 | 11.7186,0 | 29 | 89 |
| O-acetylserine 1 | 259.2 | 11.8539,0 | 20 | 239 |
| 2-methylfumarate | 740.6552 | 11.8849,0 | 29 | 184 |
| methyl trans-cinnamate | 348.5 | 11.987,0 | 28 | 103 |
| 2-Methylglutaric Acid | 442.7143 | 12.0277,0 | 29 | 215 |
| N-Ethylglycine 1 | 386.92 | 12.116,0 | 29 | 218 |
| beta-Alanine 2 | 795.5 | 12.2163,0 | 24 | 248 |
| aspartic acid 2 | 909.8966 | 12.2136,0 | 29 | 160 |
| Erythrose 2 | 614.6552 | 12.2457,0 | 29 | 201 |
| 6-hydroxy caproic acid | 259.7586 | 12.3075,0 | 29 | 96 |
| 3-hydroxy-L-proline 1 | 294.3182 | 12.3661,0 | 29 | 82 |
| Maleamate 4 | 266.4286 | 12.4464,0 | 29 | 243 |
| L-Threose 2 | 469.3103 | 12.5232,0 | 29 | 350 |
| L-homoserine 1 | 550.9643 | 12.521,0 | 28 | 103 |
| 3-Aminoisobutyric acid 1 | 555.1035 | 12.6239,0 | 29 | 86 |
| Aminomalonic acid | 554.7586 | 12.7363,0 | 29 | 69 |
| L-Malic acid | 811.2857 | 12.9587,0 | 14 | 66 |
| Ethyl cinnamate | 256 | 13.1313,0 | 18 | 58 |
| 4-Hydroxy-6-methyl-2-pyrone | 260.3333 | 13.1718,0 | 8 | 156 |
| Threitol | 905.6207 | 13.1862,0 | 29 | 217 |
| 1,5-Anhydroglucitol | 297.0909 | 13.2581,0 | 17 | 143 |
| salicylic acid | 250 | 13.2983,0 | 19 | 159 |
| aspartic acid 1 | 931.2069 | 13.3679,0 | 29 | 232 |
| oxoproline | 850.8621 | 13.4461,0 | 29 | 156 |
| 4-aminobutyric acid 1 | 823.8214 | 13.4955,0 | 28 | 304 |
| phenylalanine 2 | 610.375 | 13.9377,0 | 8 | 120 |
| 4-Hydroxyphenylethanol | 596.4286 | 14.0417,0 | 23 | 179 |
| (2R,3S)-2-hydroxy-3-isopropylbutanedioic acid | 472.05 | 14.0873,0 | 29 | 234 |
| 3-hydroxy-3-methylglutaric acid | 547.6 | 14.337,0 | 17 | 258 |
| Digitoxose 2 | 552.4828 | 14.3891,0 | 29 | 204 |
| D-erythronolactone 2 | 568.6667 | 14.4641,0 | 29 | 307 |
| tartaric acid | 427.5238 | 14.6612,0 | 23 | 333 |
| 2-mercaptoethanesulfonic acid 2 | 412.3333 | 14.6866,0 | 29 | 229 |
| 4-Hydroxybenzoic acid | 823.5556 | 14.7268,0 | 11 | 223 |
| Lyxose 1 | 917.1035 | 14.9961,0 | 29 | 307 |
| creatine degr | 258.35 | 15.0294,0 | 21 | 245 |
| 2,4-diaminobutyric acid 2 | 427.0357 | 15.0868,0 | 28 | 174 |
| xylose 1 | 714.8929 | 15.1576,0 | 28 | 217 |
| ribose | 673.1154 | 15.3098,0 | 29 | 234 |
| cis-1,2-Dihydronaphthalene-1,2-diol | 470.6957 | 15.4089,0 | 28 | 201 |
| Ribonic acid, gamma-lactone | 573.4828 | 15.4192,0 | 29 | 231 |
| Synephrine 2 | 655.92 | 15.4035,0 | 27 | 267 |
| xylitol | 696.2069 | 15.5049,0 | 29 | 217 |
| Acetol 4 | 450.5556 | 15.6155,0 | 29 | 172 |
| D-Arabitol | 649.4074 | 15.8685,0 | 29 | 205 |
| Diglycerol 2 | 625.4815 | 15.9961,0 | 27 | 333 |
| 3,6-Anhydro-D-galactose 3 | 660.4828 | 16.0129,0 | 29 | 231 |
| D-(glycerol 1-phosphate) | 634.9167 | 16.1259,0 | 24 | 299 |
| Glucose-1-phosphate | 864.4483 | 16.1817,0 | 29 | 232 |
| 4-hydroxy-3-methoxybenzoic acid; | 551.9286 | 16.2765,0 | 28 | 297 |
| 2-deoxy-D-glucose 2 | 452.8148 | 16.4254,0 | 28 | 302 |
| 2-Deoxy-D-galactose 2 | 602.125 | 16.5416,0 | 24 | 437 |
| shikimic acid | 436.3333 | 16.7008,0 | 12 | 204 |
| citric acid | 765.5294 | 16.7313,0 | 17 | 273 |
| alpha-D-glucosamine 1-phosphate | 409.6471 | 16.9432,0 | 17 | 334 |
| Analyte 646 | 522.4 | 17.0338,0 | 23 | 235 |
| Tagatose 1 | 281.0667 | 17.0397,0 | 26 | 316 |
| quinic acid | 793.0345 | 17.1214,0 | 29 | 195 |
| sorbose 1 | 734.7778 | 17.2455,0 | 27 | 363 |
| fructose 2 | 674.6429 | 17.4837,0 | 28 | 52 |
| D-Altrose 1 | 347.4167 | 17.5368,0 | 12 | 317 |
| 2-Keto-L-gulonic acid | 485.5 | 17.598,0 | 16 | 449 |
| mannose 1 | 448.1667 | 17.6173,0 | 12 | 347 |
| L-Gulonolactone | 239.4211 | 17.7948,0 | 29 | 379 |
| D-Talose 2 | 333.65 | 17.8348,0 | 20 | 305 |
| glucose 2 | 671.1539 | 17.9087,0 | 26 | 235 |
| lysine | 568.2308 | 17.9201,0 | 27 | 156 |
| Gluconic lactone 3 | 367.32 | 17.9484,0 | 25 | 275 |
| Sedoheptulose | 688.7857 | 17.9991,0 | 28 | 204 |
| D-galacturonic acid 1 | 631.9565 | 18.0447,0 | 23 | 333 |
| ascorbate | 663.0741 | 18.1079,0 | 27 | 332 |
| 4-hydroxycinnamic acid | 821.4138 | 18.1216,0 | 29 | 249 |
| conduritol b epoxide 2 | 714.0345 | 18.1893,0 | 29 | 265 |
| o-Hydroxyhippuric acid 1 | 135.5 | 18.267,0 | 14 | 523 |
| gly-pro | 244.875 | 18.5432,0 | 17 | 288 |
| gluconic acid 1 | 813.12 | 18.5468,0 | 25 | 333 |
| Galactonic acid | 415.6667 | 18.5637,0 | 20 | 204 |
| N-alpha-Acetyl-L-ornithine 2 | 173.5263 | 18.603,0 | 24 | 214 |
| Saccharic acid | 867.6207 | 18.6562,0 | 29 | 333 |
| gluconic acid 1 | 811.8214 | 18.6688,0 | 29 | 359 |
| mucic acid | 850.8276 | 18.9272,0 | 29 | 333 |
| O-Succinylhomoserine | 158.5714 | 18.9951,0 | 17 | 195 |
| palmitic acid | 919.0345 | 19.033,0 | 29 | 117 |
| mucic acid | 885.5172 | 19.075,0 | 29 | 333 |
| N-Acetyl-D-galactosamine 2 | 485.9655 | 19.24,0 | 29 | 204 |
| N-Acetyl-beta-D-mannosamine 1 | 624.2667 | 19.3003,0 | 15 | 87 |
| myo-inositol | 840.9286 | 19.4521,0 | 28 | 508 |
| ferulic acid | 846.6552 | 19.4961,0 | 29 | 338 |
| phenylacetaldehyde 1 | 315.8571 | 19.5836,0 | 11 | 159 |
| trans-3,5-Dimethoxy-4-hydroxycinnamaldehyde 1 | 363.963 | 19.6932,0 | 29 | 174 |
| Glucoheptonic acid 3 | 632.7222 | 19.7443,0 | 18 | 301 |
| d-Glucoheptose 1 | 673.8333 | 19.8153,0 | 26 | 274 |
| caffeic acid | 705.3 | 19.8522,0 | 20 | 396 |
| noradrenaline | 575.8276 | 20.0274,0 | 29 | 174 |
| Phytol | 781.1304 | 20.1619,0 | 23 | 143 |
| beta-Mannosylglycerate 1 | 777.9583 | 20.4458,0 | 24 | 204 |
| Fructose 2,6-biphosphate degr prod 2 | 478.3214 | 20.4691,0 | 29 | 211 |
| linoleic acid | 777.8182 | 20.5064,0 | 11 | 67 |
| 3-Indolepyruvic acid | 263.4231 | 20.5433,0 | 27 | 301 |
| oleic acid | 762.8333 | 20.5616,0 | 24 | 117 |
| linolenic acid | 563.1923 | 20.577,0 | 26 | 79 |
| Elaidic acid | 649 | 20.617,0 | 17 | 84 |
| 2-aminoethanethiol | 431.9231 | 20.7013,0 | 27 | 174 |
| stearic acid | 919.32 | 20.7995,0 | 29 | 117 |
| trans-sinapinic acid | 789.5862 | 20.8321,0 | 29 | 338 |
| N-2-Fluorenylacetamide 1 | 293.3478 | 21.0393,0 | 28 | 221 |
| alpha-Santonin 1 | 421 | 21.1068,0 | 14 | 245 |
| fructose-6-phosphate | 587 | 21.2441,0 | 25 | 315 |
| glucose-6-phosphate 1 | 668.931 | 21.3489,0 | 29 | 387 |
| Phenyl beta-D-glucopyranoside | 629.1429 | 21.5157,0 | 29 | 73 |
| Purine riboside | 455.5 | 22.0857,0 | 29 | 105 |
| D-erythro-sphingosine 2 | 635.5862 | 22.4397,0 | 29 | 204 |
| 5-Methoxytryptamine 2 | 602.4286 | 22.5695,0 | 14 | 174 |
| cytidine-monophosphate degr prod | 404.8333 | 22.6218,0 | 29 | 105 |
| uridine 1 | 537.2941 | 22.8029,0 | 21 | 257 |
| DL-dihydrosphingosine 1 | 624.3704 | 22.8096,0 | 29 | 321 |
| Neohesperidin | 616.625 | 22.9079,0 | 17 | 257 |
| salicin | 303.5 | 22.9641,0 | 28 | 105 |
| arbutin | 520.3529 | 23.4654,0 | 25 | 259 |
| 1-Monopalmitin | 669.3889 | 23.5754,0 | 18 | 371 |
| sucrose | 644 | 24.053,0 | 11 | 160 |
| prostaglandin A2 3 | 219 | 24.3838,0 | 21 | 406 |
| lactulose 2 | 718.1 | 24.4577,0 | 10 | 82 |
| lactose 1 | 740.931 | 24.5599,0 | 29 | 204 |
| cellobiose 2 | 615.9524 | 24.5678,0 | 22 | 373 |
| 2-Monoolein | 442.6 | 24.5464,0 | 19 | 82 |
| trehalose | 935.6552 | 24.6414,0 | 29 | 191 |
| maltose | 862 | 24.7949,0 | 24 | 160 |
| Sophorose 1 | 863.6296 | 24.8613,0 | 27 | 319 |
| Lactobionic Acid 1 | 680.8696 | 24.9712,0 | 23 | 204 |
| Turanose 2 | 607.8125 | 25.0806,0 | 16 | 307 |
| Gentiobiose 1 | 912.7857 | 25.1681,0 | 28 | 160 |
| Gentiobiose 1 | 876.7931 | 25.2896,0 | 29 | 160 |
| Palatinose | 757.6207 | 25.3607,0 | 29 | 103 |
| kyotorphin 1 | 181.4546 | 25.3882,0 | 14 | 223 |
| melibiose 1 | 873.2069 | 25.5251,0 | 29 | 361 |
| Digalacturonic acid 1 | 682.1875 | 25.6049,0 | 16 | 292 |
| prunin degr. Prod. 1 | 755.8276 | 25.6538,0 | 29 | 332 |
| melibiose 2 | 839.3704 | 25.7447,0 | 28 | 361 |
| naringenin 1 | 421.3704 | 25.8283,0 | 27 | 204 |
| Galactinol 3 | 802.2222 | 25.9901,0 | 27 | 204 |
| hydrocortisone | 409.2857 | 26.347,0 | 29 | 119 |
| Chlorogenic Acid 2 | 597.1724 | 26.5105,0 | 29 | 204 |
| guanosine-5'-monophosphate | 240 | 26.8641,0 | 21 | 295 |
| Tetrahydrocorticosterone 1 | 339 | 27.2625,0 | 17 | 204 |
| 21-hydroxypregnenolone 4 | 345 | 27.2849,0 | 25 | 249 |
| 5-Dihydrocortisol 1 | 491.6 | 27.6618,0 | 23 | 204 |
| trehalose-6-phosphate | 581.3103 | 27.8114,0 | 29 | 361 |
| Cholestan-3beta-ol | 255.5 | 28.1432,0 | 18 | 204 |
| naringin | 646.4828 | 28.7844,0 | 29 | 361 |
| Tricetin | 219.3333 | 28.869,0 | 20 | 342 |
| 1-Kestose | 701.6552 | 29.0222,0 | 29 | 319 |
| raffinose | 761.1035 | 29.1048,0 | 29 | 204 |
| maltotriose 1 | 575 | 30.2579,0 | 22 | 204 |
| Maltotriitol | 510.3333 | 31.2922,0 | 10 | 204 |

For the GC-Quad FiehnLib library, derivatives were designated with increasing numbers according to the retention index (e.g., serine 1, serine 2, and serine 3)
