# Supplementary material for: New insights into the evolution of host specificity of three Penicillium species and the pathogenicity of P. Italicum involving the infection of Valencia orange (Citrus sinensis)
Source: Virulence. 2020 Jun 11;11(1):748–68. doi: 10.1080/21505594.2020.1773038 (PMC7549954; doi:10.1080/21505594.2020.1773038)
Supplement: Supplemental Material [file KVIR_A_1773038_SM2584.zip › Table S5.docx]

**Table S5** Details regarding the qPCR primers

| Gene | Sequence of primer (5’ to 3’) |
| --- | --- |
| *orange1.1t03646.1* | For: CACAACCTTATCAGTGACCTTGCTCAATGG |
|  | Rev: CACAACCTTATCAGTGACCTTGCTCAATGG |
| *orange1.1t04470.1* | For: AAGGGCTGGGTAAGGTGTCACATTTAGAT  Rev: GGCGGTTGTAAGGCTTCAAGAAGTTGT |
| *Cs1g12910.1* | For: AACTTCAGTCGGTTCTCATACGGAATTGC  Rev: CATCACAACCAACTATTCGCAGCCACTT |
| *orange1.1t04515.1* | For: CCGTACAGCAGAAGTCCAGATACTTACCAA  Rev: CAAGGAGCCCAAGCCCAGAAACAATG |
| *Cs3g15210.1* | For: GCGTGAAGAAGAGCATCAAGACATACAAGA  Rev: TGAATTTCCCAAGATGTAATCGTCGGCTTC |
| *Cs3g11060.1* | For: GCCATCCAGCCAATGCCTTGAGTG  Rev: TGAACCCGAGACCAACAAACACCAGTA |
| *orange1.1t04584.1* | For: CAGGTTAGAGGAGTCCACCAACTTGTCAT Rev: GAACTTCGGCAACAAATCGTAGAGAACCAT |
| *orange1.1t03736.1* | For: AGAGATACAGTTCAACCGAGGCAACACT Rev: TCCCACTCCTCCAATTCCATCAATCCC |
| *orange1.1t04573.1* | For: GCTTCAATCTCTGCCACAACTTCCATCTAG Rev: CTGACACTGCCTCAAGGTACTCTCGTAG |
| *Cs3g04030.1* | For: GAGCCGTCTTCATCTTCAGCATCATCATC Rev: TCCTCGTAATCCTGTAATTCCACTCTTCCA |
| *Cs3g11080.1* | For: TCACTTGATGACTTCCTCCAGAGACTTCAC Rev: TGCTTCACTTATTTCTACTGCCATGCCATT |
| *orange1.1t01794.1* | For: GCATACGAACCATACGCCAGTTCTACG |
|  | Rev: GCATACGAACCATACGCCAGTTCTACG |
| *Cs1g01140.1* | For: CTGTCACTGGTTATAGGCATCTCCTGGTT Rev: TTCTTGTCACTTGTGTCCTGTCCTCCAA |
| *Cs3g05430* | For: GGCTTACTCGGTGCTGCTGGAATTG  Rev: TTCACCACCACCTCCAATATCTCCACATT |
| *Orange1.1t05805* | For: TCAAGCAAGGAAAGCGGCAATAGACT  Rev: CATCACATTCAGGCAATTACCTGGCTACTA |
| *Cs5g01775* | For: GGCTTACTCGGTGCTGCTGGAATTG  Rev: CATTGGCTTTGCTTCTTGTGATCCTGACT |
| *Cs5g01780* | For: GGAGACGATCTTGTGTACGCTGAACCA Rev: CAGGCAGGAGAAGCAAAGTGTGGAAC |
| *Cs7g22230* | For: CAGGAGACGATCTTATGTACGCTGAACCA Rev: TGCCTTCGCTGTCATATACATCTTACTTGC |
| *Cs5g01790* | For: CAGGAGACGATCTTATGTACGCTGAACCA Rev: TGCCTTCGCTGTCATATACATCTTACTTGC |
| *LCB1 (NCBI-GeneID: 102615172)* | For: CCATATTGCGGACAGGCTTATCGGATATTC Rev: CTAATTCCCACAGGCAGACGGCATTTATC |
| *LCB2 (NCBI-GeneID: 102617633)* | For: CCTCAGGCAGAATGTGGCTGTTGTG  Rev: GGTCCTCCTTGGTATGCGATGCTGATA |
| *LCB2-like (NCBI-GeneID: 102616935)* | For: ATGGTGGCTACATTGCAGGATCTAAGGAA Rev: AAGGACGAGACTTTGGCAGGGTTGT |
| *G3PDH (C. sinensis XM_006476919.1)* | For: AGTGAGGCTGGAGAAGGA  Rev: CAACAAAGTCGGTGGATA |
| *Actin (C. sinensis CX297817.1)* | For: GGGATTTGTTGTTTCTTCGTGGTCGTA  Rev: GGTTCTTAGTAGCAGTCGGCGATCT |
