# Supplementary figures and images for: New insights into the evolution of host specificity of three Penicillium species and the pathogenicity of P. Italicum involving the infection of Valencia orange (Citrus sinensis)
Source: Virulence. 2020 Jun 11;11(1):748–68. doi: 10.1080/21505594.2020.1773038 (PMC7549954; doi:10.1080/21505594.2020.1773038)

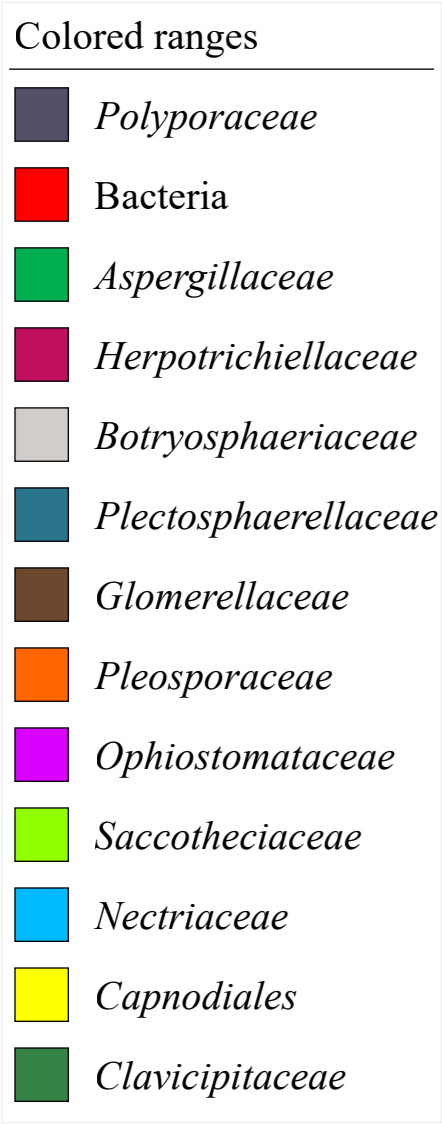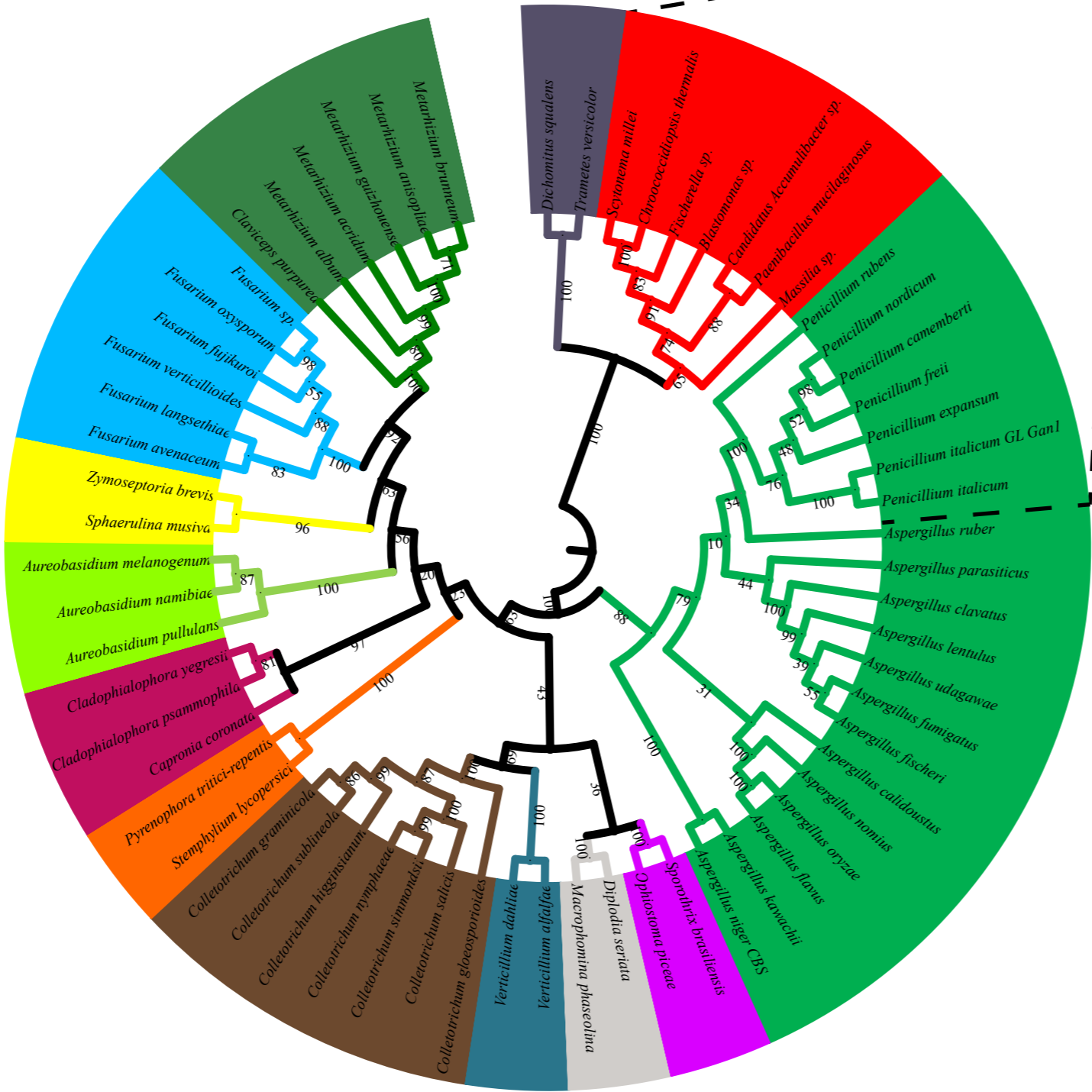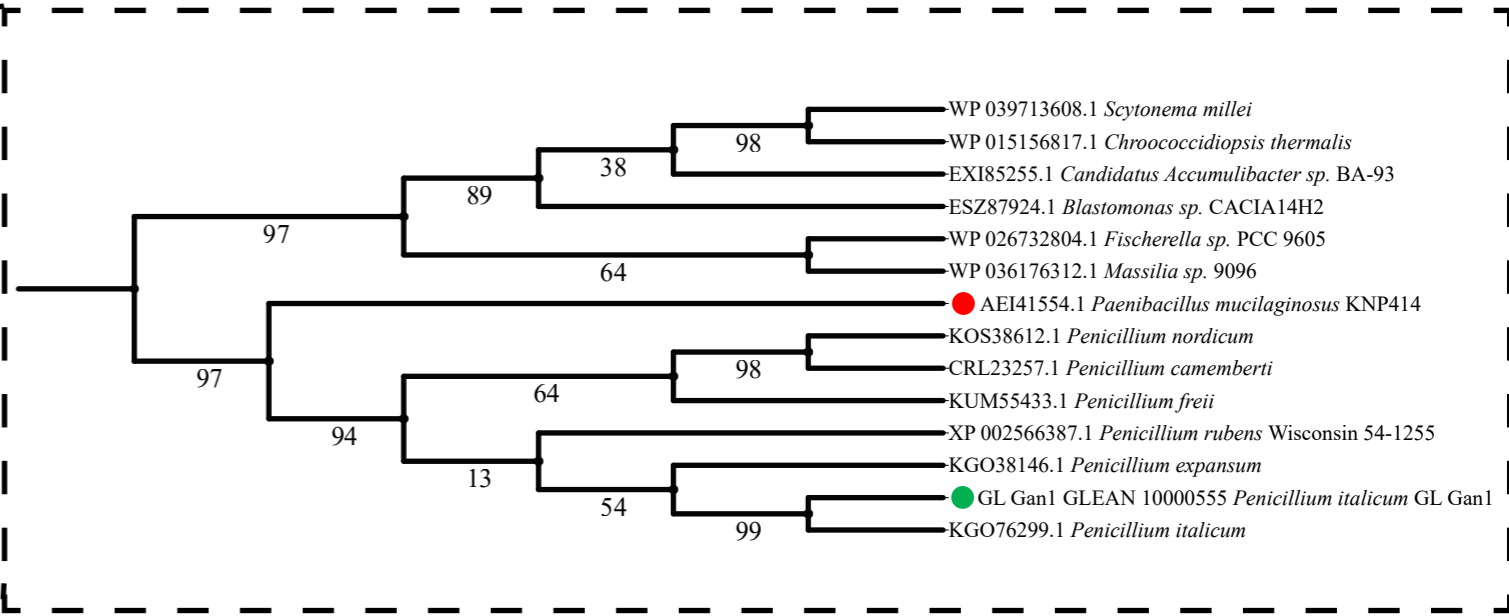

Supplement: Supplemental Material [file KVIR_A_1773038_SM2584.zip › Figure S2.pdf]
